# Molecular insights into chirality transfer from double axially chiral phosphoric acid in a synergistic enantioselective intramolecular amination†

**DOI:** 10.1039/d1sc05749a

**Published:** 2021-12-29

**Authors:** Soumi Tribedi, Raghavan B. Sunoj

**Affiliations:** Department of Chemistry, Indian Institute of Technology Bombay Powai Mumbai 400076 India sunoj@chem.iitb.ac.in

## Abstract

In the most general practice of asymmetric catalysis, a chiral catalyst, typically bearing a center or an axis of chirality, is employed as the chiral source for imparting enantiocontrol over the developing product. Given the current interest toward optically pure compounds, various forms of chiral induction enabled by diverse chiral sources as well as the use of multiple catalysts under one-pot conditions have been in focus. In one such promising development, an achiral *N*-sulfonamide protected 1,6-amino allyl alcohol (NaphSO_2_NHCH_2_C(Ph)_2_CH_2_CH=CHCH_2_OH) was subjected to Tsuji–Trost activation and an intramolecular amination to form important chiral pyrrolidine frameworks. A dual catalytic system comprising Pd(PPh_3_)_4_ and DAPCy (β-cyclohexyl substituted double axially chiral phosphoric acid derived from two homocoupled BINOL backbones with a dynamic central chiral axis) under mild conditions was reported to offer quantitative conversion with an *ee* of 95%. Here, we provide molecular insights into the origin of chiral induction by DAPCy, as obtained through a comprehensive density functional theory (SMD_(toluene)_/B3LYP-D3/6-31G**,Pd(SDD)) investigation. Two key steps in the mechanism are identified to involve a cooperative mode of activation of the Pd-bound allyl alcohol in the form of a Pd-π-allyl moiety at one end of the substrate, followed by an intramolecular nucleophilic addition of *N*-sulfonamide from the other end to yield a pyrrolidine derivative bearing an α-vinyl stereogenic center. (*S*,*R*,*S*)-DAPCy is found to steer the dehydroxylation to yield a Pd-π-allyl intermediate with a suitably poised *si* prochiral face for the nucleophilic addition. In the enantiocontrolled (as well as the turn-over determining step) nucleophilic addition, the chiral catalyst is identified to serve as a chiral phosphate counterion. The chiral induction is facilitated by a series of N–H⋯O, C–H⋯O, C–H⋯π, lone pair (lp)⋯π, O–H⋯O, O–H⋯π, and π⋯π noncovalent interactions, which is noted as more effective in the lower energy C–N bond formation transition state through the *si* prochiral face of the Pd-π-allyl moiety. These insights into the novel dynamic axially double chiral catalyst could be valuable toward exploiting such modes of stereoinduction.

## Abstract 2

The origin of enantiocontrol in an intramolecular amination involving Pd(PPh_3_)_4_ and a double axially chiral phosphoric acid (DAPCy) dual catalytic system is traced to a more effective series of noncovalent interactions in the lower energy C–N bond formation transition state.

## Graphical abstract

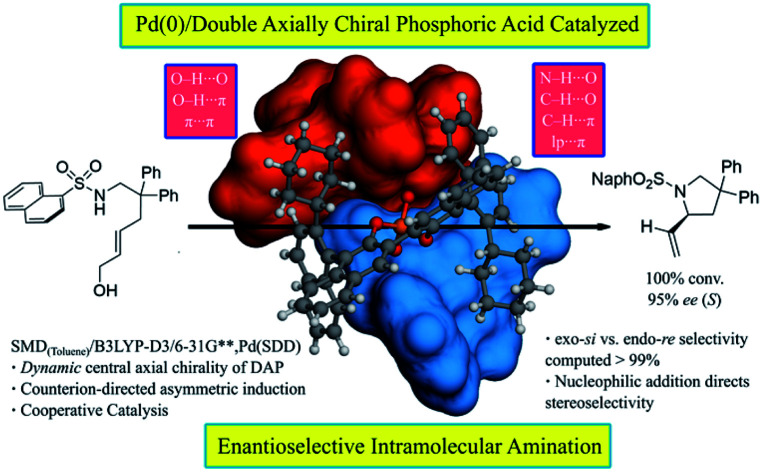

## Introduction

Recent years witnessed impressive activities that demonstrated the versatile use of chiral phosphoric acids (CPAs) in organo- as well as transition metal catalyzed reactions.^1^ While both these forms of catalytic applications of CPAs continue to make concurrent progress,^2^ what became more intriguing is their multi-catalytic cooperative/synergistic action in a plethora of one-pot reactions.^3^ Interesting modulations in reactivity and selectivity could be accomplished when well-established transition metal catalytic protocols, such as a suitably chosen Pd catalyst, are made to work in the presence of catalytic amounts of CPAs. Chiral CPAs are generally known to induce asymmetry by participating as an inner-sphere ligand bound to the transition metal or as a counterion to the transition metal catalyst/substrate.^4^

One of the most widely used families of CPAs is the BINOL-derived axially chiral phosphoric acids. Apart from their inherent axial chirality, the 3,3′-substituents on the BINOL backbone are shown to participate in vital noncovalent interactions with the reaction partners.^5^ In certain reactions, enantioselectivity could even be inverted while maintaining the same catalyst chirality, but by varying the 3,3′-substituents.^6^ As a natural progression, the concept of stereoinduction using BINOL-CPAs has been expanded to encompass double axially chiral frameworks as well.^7,8^ These are newer and promising avenues toward harnessing the potential of CPAs in asymmetric catalysis, where a handful of fascinating examples have been reported in recent years.

**Scheme 1:**
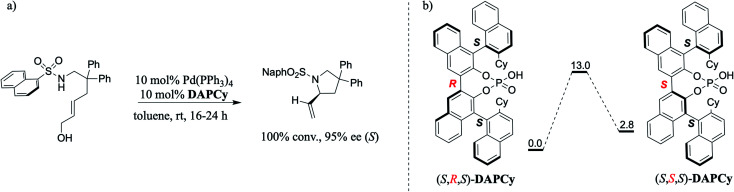
(a) The intramolecular asymmetric amination of achiral *trans*-allyl alcohol catalyzed by (*S*,*R*,*S*) double-axially chiral phosphoric acid (DAPCy) and tetrakis(triphenylphosphine) palladium. (b) The interconversion between (*S*,*R*,*S*)- and (*S*,*S*,*S*)-DAPCy diastereomers through a rotation around the central binaphthyl axis. The relative Gibbs free energies are in kcal mol^−1^.

In one such elegant report, Toste and co-workers demonstrated that a double axially chiral phosphoric acid (henceforth, abbreviated as DAP) could offer quantitative conversion and high enantioselectivities in an intramolecular asymmetric allylic amination of achiral *trans*-allyl alcohol (Scheme 1).^8^ We note that allylic substitution is an important process in enzyme catalysis as well as in CPA catalyzed asymmetric transformations.^9^ Here, the achiral *trans*-allyl alcohol is subjected to a Tsuji–Trost mode of activation to form a Pd-π-allyl moiety at one end of the substrate, which upon an intramolecular amination leads to the desired chiral pyrrolidine framework bearing a stereogenic carbon. To improve the utility of such asymmetric multi-catalytic reactions, compatibility between various catalytic dyads as well as the underlying synergism in their mechanism of action needs to be established. Given that the mechanistic underpinnings as well as the insights into the factors governing the chirality transfer in the DAP family of catalysts remain unclear,^7*d*,8^ we became interested in probing these aspects by using modern computational methods. It shall be noted that a cyclohexyl substituted double axially chiral phosphoric acid (DAPCy) is chosen as the catalyst for the present investigation. In particular, we aim to shed light on (a) the cooperativity between the Pd(0) and DAP catalysts and their respective roles in substrate activation, (b) the mode of participation of the chiral DAPCy catalyst, either as a counterion or a Pd-bound ligand, and (c) how the chiral information gets transferred from the double axially chiral phosphoric acid to the substituted pyrrolidine product bearing one new stereogenic carbon center.

We report important molecular insights into the dual-catalytic asymmetric allylic amination involving a combination of Pd(PPh_3_)_4_ and a double axially chiral phosphoric acid catalyst as the source of chirality (Scheme 1(a)). The discussions are organized into five sections; (a) nature of the active catalyst, (b) overview of the catalytic cycle, (c) configurations of important stationary points, (d) enantiocontrolling step, and (e) origin of enantioselectivity.

## Computational methods

All the computations were performed using the Gaussian 09 (RevisionD.01)^10^ suite of quantum chemical program. All geometries were optimized in a solvent continuum with a dielectric constant of toluene, using the Cramer–Truhlar SMD^11^ solvation model that employs quantum mechanical charge densities of solutes, employing the B3LYP-D3 hybrid density functional theory^12^ with Pople's 6-31G** basis set for all atoms except palladium.^13^ The Stuttgart/Dresden (SDD) ECP basis set with relativistic corrections for the 28 inner shell electrons and explicit basis set for the remaining 18 valence electrons were used for palladium.^14^ All of the stationary points were characterized as minima or a first-order saddle point (transition state) by evaluating the corresponding Hessian indices. The transition states were verified by examining whether they have a unique imaginary frequency representing the desired reaction coordinate. Further verification that these transition states connect to the expected minima (toward the reactant and product) was carried out by using the intrinsic reaction coordinate (IRC) calculation.^15^ Grimme's rigid-rotor-harmonic-oscillator approximation (RRHO) model was employed for obtaining improved estimates of the entropic contribution arising from the low frequency vibrational modes (<100 cm^−1^).^16^ The discussions in this manuscript are presented on the basis of the Gibbs free energies as obtained at the SMD_(toluene)_/B3LYP-D3/6-31G**,Pd(SDD) level of theory.^17^ The noncovalent interactions in the transition states were identified using the topological analyses of electron densities within the Bader's Atoms-in-Molecule (AIM) formalism^18^ by using the AIM2000 program.^19^ Further, the reduced density plots were generated for qualitative graphical analysis of the noncovalent interactions by using the NCIPLOT 3.0.^20^ The activation strain analysis was performed to calculate the relative extent of distortion and interaction energies in the stereocontrolling TSs.^21^ The Shaik-Kozuch energetic span model was applied on the Gibbs free energy profile to identify the turnover determining intermediate (TDI) and the turnover determining transition state (TDTS) pertaining to the catalytic cycle.^22^

## Results and discussion

### (a) Nature of the potential active catalyst

An important aspect pertaining to the axes of chirality in the DAPCy catalyst is given careful consideration first. The computed rotational barrier around the central axis (Scheme 1(b)) is found to be 13.0 kcal mol^−1^, indicating the likelihood of a dynamic equilibrium between the (*S*,*R*,*S*) and (*S*,*S*,*S*) diastereomers of DAPCy under room temperature conditions employed for this reaction.^23^ Whether or not both these diastereomers would partake in the reaction with similar or different catalytic efficiency and/or enantioselectivity remains to be seen. While the overall mechanism is presented in light of the results obtained through a comprehensive investigation by using (*S*,*R*,*S*)-DAPCy as the representative catalyst, both catalyst diastereomers were separately considered for probing their role in the enantiocontrolling step (*vide infra*).

**Fig. 1:**
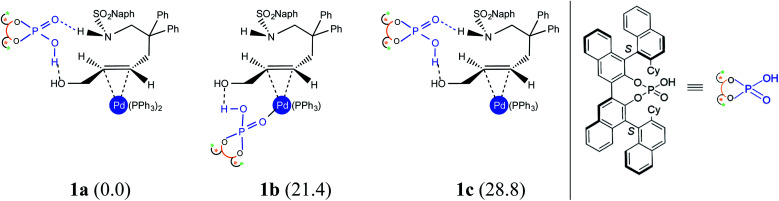
Catalyst–substrate complexes that differ in the mode of DAPCy binding and the number of Pd-bound triphenyl phosphine ligands. The relative free energies (in kcal mol^−1^) are provided in parentheses.

Under one-pot dual catalytic conditions, consisting of Pd(PPh_3_)_4_ as the transition metal precursor and DAPCy as the chiral source, it is prudent to consider different possible ways the substrate may combine with the catalysts in the initial stage of the reaction. Shown in Fig. 1 are three such possibilities that differ in terms of the primary ligands bound to the Pd center as well as in the mode of engagement of DAPCy. The congested coordination environment of Pd(PPh_3_)_4_ is known to make space for the incoming substrate/ligand through ligand dissociation to form Pd(PPh_3_)_2_.^24^ The catalyst–substrate complex 1a, wherein Pd(PPh_3_)_2_ forms a π-olefin complex with the substrate *trans*-allyl alcohol can therefore be considered a highly probable species. In 1a, the immediate coordination sites around Pd are occupied by two strongly coordinating phosphines as well as the substrate. As can be gathered from the Gibbs free energies of formation given in Fig. 1, the progressive removal of the native PPh_3_ ligand, or exchanging them with DAPCy, is not quite favorable.^25^ On the basis of the predicted energetic advantage, we have considered the catalyst–substrate complex 1a as the key species entering the catalytic cycle. In 1a, a relatively stronger hydrogen bonding between the P=O oxygen of DAPCy and the N–H of the sulfonamide group in the substrate is noticed besides several other noncovalent interactions.^26^

### (b) Overview of the catalytic cycle

**Scheme 2:**
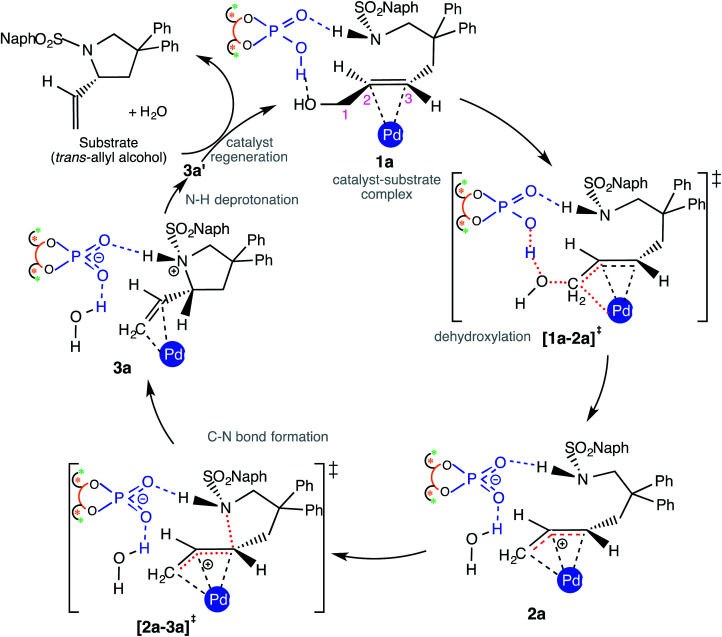
Important catalytic steps in the enantioselective intramolecular allylic amination.

Insofar as the major catalytic events go, the DAPCy catalyzed dehydroxylation in 1a is the first important step imparting an electrophilic character to the allylic end of the substrate. As shown in Scheme 2, DAPCy protonates the allylic hydroxyl group to remove it in the form of water. The transition state for this dehydroxylation is generally denoted as [1a-2a]^‡^ and has been assigned additional stereochemical notations as applicable (*vide infra*).^27^ What appears to be an acid assisted dehydroxylation to form the Pd-π-allyl intermediate presents a number of intriguing mechanistic nuances. The identification of the energetically most preferred dehydroxylation transition state can be intertwined with (i) the conformational possibilities arising due to the possible rotations around various C–C bonds in the substrate^28^ and (ii) the open prochiral face of the allyl alcohol available for the ensuing intramolecular nucleophilic addition, which in turn, depends on which prochiral face is energetically more preferred in its binding with Pd(PPh_3_)_2_. We have carefully considered these aspects in order to locate the most preferred geometry of [1a-2a]^‡^. Dehydroxylation leads to a Pd-π-allyl intermediate (2a) and a DAPCy phosphate counterion, held together through hydrogen bonds and other NCIs. In the intramolecular nucleophilic addition, the nitrogen of the *N*-sulfonamide is added to the C3 carbon of the Pd-π-allyl moiety *via* the ring closing transition state [2a-3a]^‡^ to the protonated variant of the product–catalyst complex 3a. In 3a, the *N*-protonated product is bound to both Pd(PPh_3_)_2_ and DAPCy-ate catalysts primarily through a π-olefin η^2^ interaction and hydrogen bonding, respectively. The regeneration of the neutral DAPCy catalyst can be accomplished through a proton transfer from the nitrogen to the phosphate.^29^ The corresponding neutral intermediate 3a' is the true pyrrolidine product, which upon displacement by a new molecule of allyl alcohol would release the product (and 1a) and complete one full catalytic cycle.

### (c) Configurations of intermediates and transition states

**Fig. 2:**
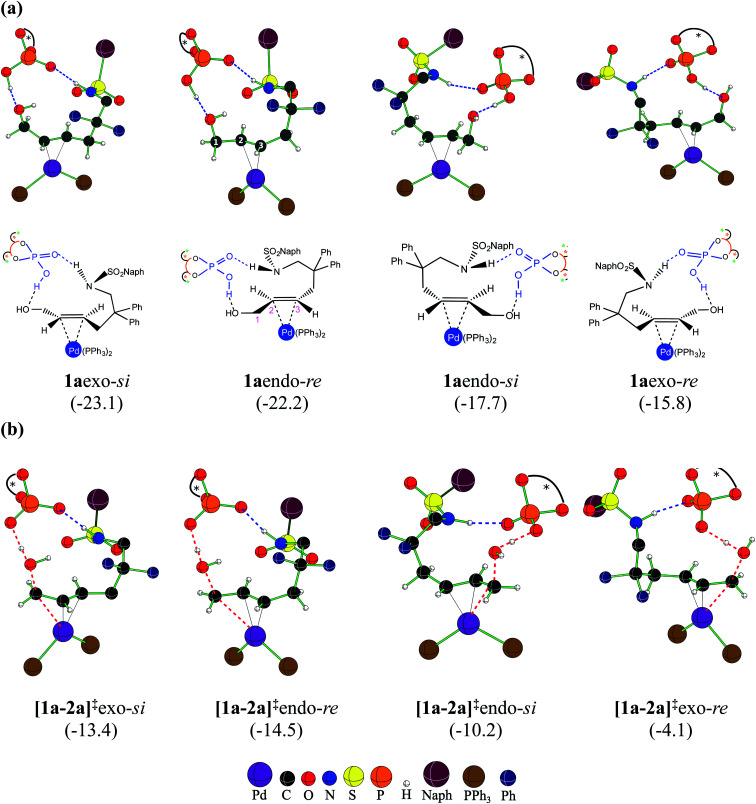
The configurations arising due to different possible binding modes in (a) the catalyst–substrate complex 1a, and (b) the dehydroxylation transition states [1a-2a]^‡^.^33^ Blue dotted lines denote hydrogen bonding interactions while the red dotted lines are the reaction coordinates. The relative free energies in kcal mol^−1^ are given in parentheses.

At this juncture, we wish to highlight certain important stereochemical features of the catalyst–substrate complex 1a as well as the Pd-π-allyl intermediate 2a. Two key binding modes of the achiral *trans*-allyl alcohol to Pd(PPh_3_)_2_ can be thought about. Depending on which face of the π-bond of the allyl alcohol is bound to Pd, the other prochiral face would be available for participation in the intramolecular nucleophilic addition. Herein, we assign the *re* and *si* configurational notations to the exposed prochiral face of the C3 carbon, as depicted in Fig. 2. Within a given configuration (*re* or *si*), when the C2–H bond is pointed towards chiral DAPCy, it is denoted as *endo* whereas *exo* refers to the geometric disposition of C2–H away from the phosphoric acid/phosphate.^30^ Another relevant aspect is the fairly strong interaction (∼−70 kcal mol^−1^) noted between DAPCy and the Pd-bound substrate. The cumulative effect of a good number of noncovalent interactions between the DAPCy catalyst and the allyl alcohol, besides a couple of prominent hydrogen bonding contacts, is found to be responsible for their strong association (Fig. 2). With such binding features between η^2^-π-allyl alcohol-Pd(PPh_3_)_2_ and the DAPCy catalyst, 1aendo and 1aexo configurations should be regarded not interconvertible. However, independent and direct formation of 1aendo-*re* and 1aexo-*si* is likely, given that their Gibbs free energies of formation are very similar. If a Boltzmann population of various configurations of 1a is considered, the two most dominant ones would be 1aexo-*si* and 1aendo-*re* that respectively leaves the *si* and *re* face open for the nucleophilic addition. These energetic details could be viewed as an early thermodynamic impetus, rendering an intrinsic advantage to 1aexo-*si* and 1aendo-*re* configurations for their participation in the ensuing catalytic events as compared to the other 1a species (shown in Fig. 2(a)) formed through alternative modes of substrate binding to the Pd-catalyst in the presence of chiral DAPCy.^31,32^

### (d) Enantiocontrolling step

With the availability of the catalyst–substrate complex, subsequent steps in the catalytic cycle, such as the dehydroxylation and the intramolecular nucleophilic addition, can take place. Enantioselectivity could be determined either in the dehydroxylation or in the ring closing C–N bond formation through the intramolecular nucleophilic addition. If the dehydroxylation is the enantiocontrolling event, it should lead to the preferential formation of a particular diastereomer of 2a, whose open prochiral face only remains accessible for the nucleophilic addition. A given diastereomer of the Pd-π-allyl⋯DAPCy-ate composite intermediate thus formed should as well be able to retain its stereochemical integrity by resisting any π–σ–π interconversion possibilities (note that the catalyst denoted as DAPCy-ate refers to the phosphate formed in the dehydroxylation step through deprotonation of the parent phosphoric acid).^34^ Alternatively, if no energetic preference toward the formation of a particular diastereomer of 2a is discernible, then the nucleophilic addition to one of the prochiral faces of the Pd-π-allyl moiety would decide the enantioselectivity. Taking cognizance of the pivotal role the Pd-π-allyl intermediate 2a is likely to play in steering the stereochemical course of this reaction, we have analyzed its formation and the ensuing reaction in greater detail.

As the first step in this direction, a careful conformational sampling of the transition states for the DAPCy assisted dehydroxylation [1a-2a]^‡^ is undertaken. Different conformers of the Pd-bound allyl alcohol, arising as a result of permissible C–C bond rotations (*e.g.*, C3–C4, C4–C5, C5–C6, C6–N7 and N7–S8 shown in Fig. 1), are separately considered toward identifying the most preferred [1a-2a]^‡^.^35^ Selected sets of [1a-2a]^‡^ conformers, in each of the four key configurations, as depicted in Fig. 2(b), are identified.^28^ With this, it is expected that the substrate assumes a geometry that maximizes the attractive interactions with the chiral phosphoric acid, in the most preferred transition state. A compilation of the relative Gibbs free energies of important dehydroxylation transition states is provided in Table 1 for both the (*S,S,S*)- and (*S,R,S*)-DAPCy catalyst configurations. The energetically most preferred mode of dehydroxylation is found to be *via* the transition state [1a-2a]^‡^endo-*re* for the (*S,R,S*)-DAPCy catalyst. The Gibbs free energies of the dehydroxylation transition state from the other configurations of 1a are found to be higher by 1.1, 4.3 and 10.4 kcal mol^−1^ for exo-*si*, endo-*si*, and exo-*re*, respectively.^36^ Two complementary energetic aspects are worth noting at this stage; (a) the most preferred binding in the catalyst–substrate complex 1a involves the exo-*si* and endo-*re* modes (Fig. 2(a)), indicating a thermodynamic incentive for these configurations and (b) the dehydroxylation TSs from these two configurations also exhibit lower barriers, implying a kinetic advantage for the formation of 2aendo-*re* and 2aexo-*si* intermediates.^37^ The earlier experimental examination^8^ revealed that the dehydroxylation step is unlikely to be the enantiocontrolling step. In addition, no clear energetic preference toward the formation of a particular diastereomer of 2a is discernible (Fig. 2).^38^ All these facts point to the intramolecular nucleophilic addition in the Pd-π-allyl intermediate as holding the key to the enantioselectivity.

**Table 1:** The relative Gibbs free energies (Δ*G*_rel_ in kcal mol^−1^) of the transition states for different stereochemical modes of dehydroxylation and intramolecular nucleophilic addition in the *trans*-Pd-π-allyl intermediate and the corresponding configuration of the product. The energies are with respect to the separated reactants

DAPCy configuration	Dehydroxylation		Nucleophilic addition		Product configuration
	TS	Δ*G*_rel_	TS	Δ*G*_rel_	
(*S,S,S*)	[1a-2a]^‡^endo-*si*	−14.5	[2a-3a]^‡^endo-*si*	−14.4	*S*
	[1a-2a]^‡^endo-*re*	−10.9	[2a-3a]^‡^endo-*re*	−9.4	*R*
	[1a-2a]^‡^exo-*si*	−8.9	[2a-3a]^‡^exo-*si*	−13.7	*S*
	[1a-2a]^‡^exo-*re*	−9.8	[2a-3a]^‡^exo-*re*	−13.7	*R*
(*S,R,S*)	[1a-2a]^‡^endo-*si*	−10.2	[2a-3a]^‡^endo-*si*	−11.6	*S*
	[1a-2a]^‡^endo-*re*	−14.5	[2a-3a]^‡^endo-*re*	−15.2	*R*
	[1a-2a]^‡^exo-*si*	−13.4	[2a-3a]^‡^exo-*si*	−19.2	*S*
	[1a-2a]^‡^exo-*re*	−4.1	[2a-3a]^‡^exo-*re*	−16.1	*R*

### (e) Origin of enantioselectivity

Next, we focused on the intramolecular nucleophilic addition of the suitably poised sulfonamide nitrogen to the exposed prochiral face in the lower energy Pd-π-allyl intermediates such as 2aendo-*re* and 2aexo-*si*. This is one of the most crucial steps resulting in the formation of a new carbon stereogenic center in the cyclized product.^34*b*^ The energetic comparison between the nucleophilic addition transition states [2a-3a]^‡^endo-*re* and [2a-3a]^‡^exo-*si*, respectively, to the *re* face and the *si* face therefore assumes high significance. In these transition states we have retained the water molecules generated through the DAPCy catalyzed dehydroxylation within the system through hydrogen bonding interactions. Although the adventitious water could diffuse away, the transition states with the hydrogen bonded water are found to be of lower energy as compared to the ones devoid of it.^39^ The analysis of the geometry of the nucleophilic addition transition states as well as the IRC calculations suggests that the sulfonamide nitrogen continues to remain in the protonated state, both during and after the C–N bond formation, all the way through the ring closed product. These geometric features would mean that the involvement of the chiral DAPCy catalyst in this step is in the form of a phosphate counterion.^40^ Such molecular insights are valuable in a broader context given that asymmetric chiral counterion directed catalysis (ACDC) is a leading theme in asymmetric catalysis.^4*d*,24*a*,41^

At this point, an intriguing connection between the catalyst configuration and the extent of enantioselectivity predicted using the Gibbs free energies of the nucleophilic addition transition states draws attention. As can be gathered from the Gibbs free energies given in Table 1, the *si* face of the Pd-π-allyl moiety is the most preferred prochiral face for the nucleophilic addition for both the (*S,S,S*)- and (*S,R,S*)-DAPCy configurations. The Gibbs free energy difference (ΔΔ*G*^‡^) between the competing diastereomeric TSs, [2a-3a]^‡^endo-*si* and [2a-3a]^‡^exo-*re*, responsible for the enantioselectivity is as low as 0.7 with (*S,S,S*)-DAPCy. On the other hand, with the (*S,R,S*)-DAPCy catalyst, the ΔΔ*G*^‡^ between [2a-3a]^‡^exo-*si* and [2a-3a]^‡^endo-*re* is 4.0 kcal mol^−1^, which corresponds to a computed *ee* of more than 99% in favor of the *S* enantiomer of the product, closer to the experimental *ee* of 95%. It is therefore most likely that the active diastereomer of the catalyst participating in the enantiocontrolling step is (*S,R,S*)-DAPCy. Taking a cue from the superior predicted enantioselectivity with the (*S,R,S*)-DAPCy catalyst, we endeavored to garner additional support by comparing the overall catalytic efficiencies between these diastereomeric variants of the catalyst.

**Fig. 3:**
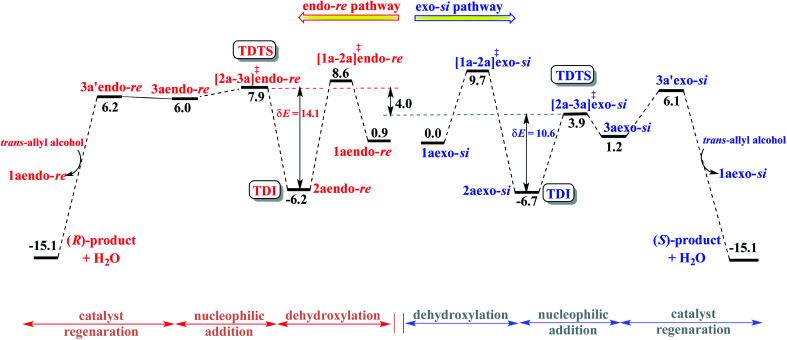
Gibbs free energy profile (in kcal mol^−1^) for enantioselective intramolecular allylic amination of *trans*-allyl alcohol with (*S*,*R*,*S*)-DAPCy and Pd(PPh_3_)_4_ dual catalytic systems. The lowest energy exo-*si* binding mode of the catalyst–substrate complex 1a is considered as the reference point.

Following the recommendation of Shaik and Kozuch,^22^ we have identified the turn-over determining intermediate (TDI) and turn-over determining transition state (TDTS) for the catalytic cycles separately for the (*S,S,S*) and (*S,R,S*) configurations of DAPCy, starting from the respective catalyst–substrate complex 1a (Fig. 3, simplified energy profile diagram). The lowest energy conformer as separately identified in the *endo* and the *exo* configurations of the catalyst–substrate complex 1a, Pd-π-allyl intermediate 2a, and that of the corresponding transition states is employed in the construction of the energy profile diagram and for the calculation of the energetic span (δ*E*). The computed δ*E* for stereochemically distinct catalyst–substrate complexes is provided in Table 2. We have considered δ*E* as the most important quantity for expressing the efficiency of competing catalytic pathways. It is evident that the higher δ*E* found in the case of (*S*,*S*,*S*)-DAPCy renders it less likely to participate as compared to (*S*,*R*,*S*)-DAPCy.

**Table 2:** The energetic span (δ*E*, in kcal mol^−1^) for both the DAPCy configurations beginning from stereochemically different possible binding modes of allyl alcohol in the catalyst–substrate complex

	Endo-*re*	Exo-*si*	Endo-*si*	Exo-*re*
(*S*,*R*,*S*)-DAPCy	14.1	10.6	11.2	13.0
(*S*,*S*,*S*)-DAPCy	15.1	12.3	13.0	12.6

Certain general and significant characteristics of this asymmetric allylic amination reaction that can be observed from Fig. 3 are (a) the TDI and TDTS are respectively 2a and [2a-3a]^‡^ for the (*S,R,S*)-DAPCy catalyst for all configurations of 1a (also true for (*S,S,S*)-DAPCy, not shown here),^42^ (b) the TDI is found prior to the TDTS, thus the difference between the Gibbs free energy of [2a-3a]^‡^ and 2a is the δ*E*, and (c) the product formation is exoergic by more than 15 kcal mol^−1^.^43^ It is conspicuous from δ*E* that the catalytic efficiency for the formation of the *S* enantiomer of the product is notably better than that for the *R* enantiomer, which is in line with the experimentally observed configuration of the major product.

**Fig. 4:**
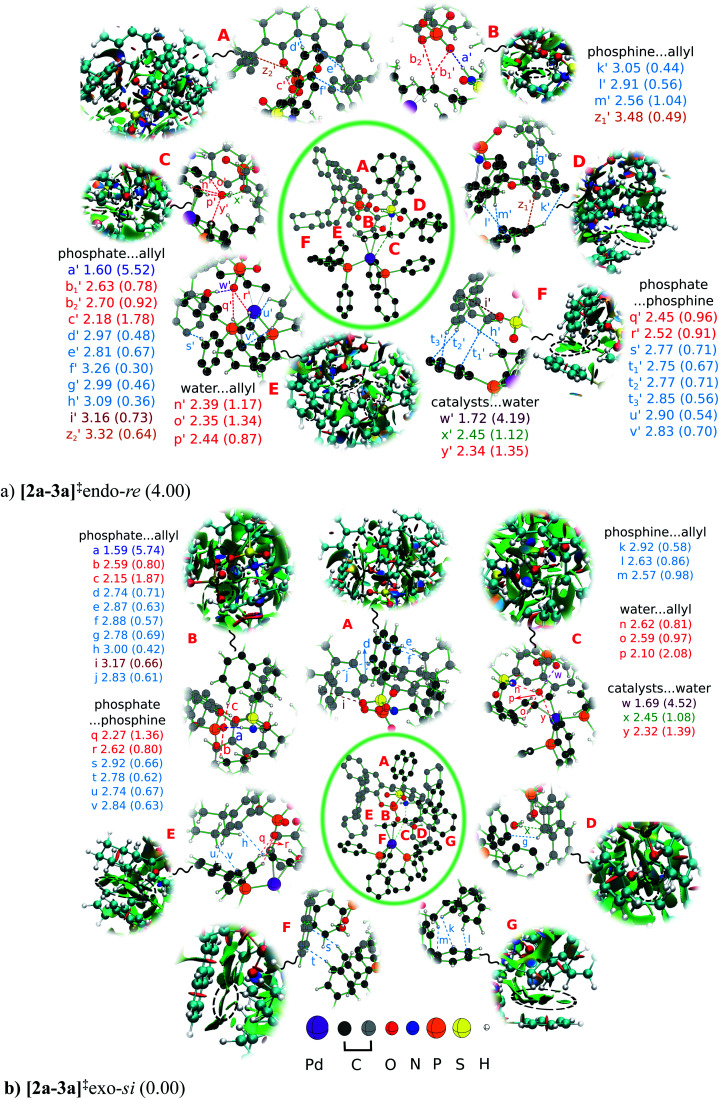
The noncovalent interactions in the enantiocontrolling transition states in the intramolecular nucleophilic addition of the sulfonamide nitrogen to the different prochiral faces of the Pd-π-allyl intermediate with (*S*,*R*,*S*)-DAPCy and Pd(PPh_3_)_4_ dual catalytic systems. The distances (in Å) are provided along with the labels for various types of interactions. The values in parenthesis beside each interaction are the electron density at the bond critical point (*ρ*_bcp_ × 10^−2^ a.u) as obtained through AIM analysis. The color codes for the dotted lines that represent various NCIs are N–H⋯O (dark blue), C–H⋯O (red), C–H⋯π (light blue), lone pair (lp)⋯π (brown), O–H⋯O (purple), O–H⋯π (green), and π⋯π (orange). NCI plots for each region are separately shown alongside the corresponding AIM map, wherein strong and weak attractive interactions are respectively depicted in blue and green leaflets and strong repulsion is in red color. Hydrogen atoms not involved in any significant interaction are omitted for improved clarity.

We have analyzed the enantiocontrolling transition states in greater detail, first by using the activation strain analysis, then by examining the NCI plot for identifying the broad regions of NCIs between the catalysts and substrate, and a more fine grain mapping of the NCIs is also carried out with the help of the atoms in molecule (AIM) topological analysis of the electron density distribution. Each of this analysis helps in deciphering different layers of valuable molecular details as presented below. The activation strain analysis revealed that the stabilizing interaction energies (*i.e.*, between the catalysts and catalyst-substrate) in [2a-3a]^‡^exo-*si* are more than 4 kcal mol^−1^ higher than those in [2a-3a]^‡^endo-*re*. However, the difference in the distortion found in the catalysts and substrate between these two competing transition states is negligible, indicating that the origin of the energetic preference might arise from various interactions.^44^ In a chirality inducing transition state, such as the intramolecular nucleophilic addition to the *si* or *re* prochiral face of the Pd-π-allyl moiety as in the present case, such differential interactions may become critical to high enantioselectivity. To identify the presence of various types of noncovalent interactions (NCIs) in the enantiocontrolling transition states, the bond paths as well as the electron densities at the bond critical points (*ρ*_bcp_) are located using the atoms in molecule formalism. These NCIs, as noted using the bond paths, are shown in Fig. 4.^45^ In general, relatively higher *ρ*_bcp_ values for a given type of interaction imply a stronger interaction.^46^ In the present case, *ρ*_bcp_ is employed as an approximate measure of the strength of individual interactions as well as for comparison of the NCIs between the competing transition states.

In Fig. 4, we combined three levels of molecular insights to make a composite representation that can be understood as described below. At the center of the figure is the full geometry of the transition state inscribed in a green oblate border. As one can imagine, full mapping all the NCIs would impact the clarity and comprehension of the associated discussion. Hence, we have first identified important regions (denoted as A, B,…, G) from within the transition state and placed them in spotlight with a magnified view to show the finer details of the NCIs present in those regions. The relevant parts, carved out of the full NCI plots are shown in the outer rim, primarily to depict the region of attractive interactions. The orientation within a magnified region has been chosen to maximize the clarity and hence may not align with the orientation of the whole transition state geometry shown in the center of the figure. For effective chiral induction, it is expected that the chiral catalyst engages in multi-point contacts with the substrate.^4*a*,17*c*,47^ Since the origin of chiral induction in double axially chiral catalysts such as DAPCy is less known, a systematic analysis of the NCIs in the enantiocontrolling transition states is undertaken to examine whether we could gather useful details that might help rationalize the energetic preference noted in favor of [2a-3a]^‡^exo-*si*.

It can be readily seen from Fig. 4 that both [2a-3a]^‡^exo-*si* and [2a-3a]^‡^endo-*re* are well decorated with several NCIs of the kinds N–H⋯O, C–H⋯O, C–H⋯π, lone pair (lp)⋯π, O–H⋯O, O–H⋯π, and π⋯π. For the ease of discussion, these interactions are classified into phosphate⋯allyl, phosphine⋯allyl, water⋯allyl, phosphate⋯phosphine, and water⋯catalysts, of which the first two belong to the most important catalyst–substrate interaction. Alphabets a, b,…, y denote the NCIs in [2a-3a]^‡^exo-*si* and those in [2a-3a]^‡^endo-*re* are represented using primed letters a′, b′,…, z′. An interaction of similar type found in both the transition states is assigned the same alphabetical notation to the extent possible. Some of these NCIs exhibit very similar *ρ*_bcp_ values in both [2a-3a]^‡^exo-*si* and [2a-3a]^‡^endo-*re* and are unlikely to contribute to the differential stabilization. The focus is therefore placed on the key differences in the pattern of NCI between these transition states.

The highest value of *ρ*_bcp_ is found for the N–H⋯O interaction (denoted as a and a′, respectively, in [2a-3a]^‡^exo-*si* and [2a-3a]^‡^endo-*re*) that serves as the anchoring contact between the chiral catalyst DAPCy-ate and the substrate Pd-π-allyl. As discussed all through the previous sections, the vital difference between the lower energy [2a-3a]^‡^exo-*si* and the higher energy [2a-3a]^‡^endo-*re* transition states presents in the form of the allyl C2–H orientation with respect to the chiral phosphate. Interestingly, in the higher energy [2a-3a]^‡^endo-*re* with C2–H pointed toward the phosphate, two additional C–H⋯O interactions with the phosphate oxygen (b_1_′ and b_2_′) are noticed. These two NCIs are obviously absent in the lower energy [2a-3a]^‡^exo-*si* due to the anti disposition between C2–H and the phosphate. The most important NCIs in these two transition states are of the phosphate⋯allyl type that comprise C–H⋯O (b and c and b_1_′, b_2_′, and c′), C–H⋯π (d, e, f, g, h, and j and d′, e′, f′, g′, and h′), lp(O)⋯π, (i and i′), and π⋯π (z_2_′) interactions. While most of these NCIs are qualitatively similar and common to both the higher and lower energy transition states, efficiency of many of the individual interactions is found to be superior in the lower energy [2a-3a]^‡^exo-*si*, as discernible from the corresponding *ρ*_bcp_ values.^48^ A similar trend of slightly better C–H⋯π interactions (k, l, and m and k′, l′, and m′) in the phosphine⋯allyl region is noticed in the case of [2a-3a]^‡^exo-*si*. The number and strength of NCIs involving the explicit water molecules are found to be very similar in both the transition states and are unlikely to contribute to the differential interaction energies. Interestingly, the number of NCIs identified between the catalysts, *i.e.*, the phosphate⋯phosphine interactions, is more in the higher energy [2a-3a]^‡^endo-*re*. However, the cumulative interaction estimated through the activation strain analysis confirms relatively better interaction in the lower energy [2a-3a]^‡^exo-*si*. Thus, the presence of more efficient interactions between the substrate and catalyst assumes pivotal importance in rendering the cyclization through the *si* face of the Pd-π-allyl intermediate the most favored than through the competing [2a-3a]^‡^endo-*re*.

## Conclusions

A comprehensive computational investigation on the mechanism of a dual catalytic intramolecular asymmetric allylic amination as well as the analysis of the enantiocontrolling step helped us gain valuable molecular insights. The major catalytic steps have been found to involve the cooperative participation of both catalysts, namely Pd(0) and the double axially chiral phosphoric acid (DAPCy), in the activation of allyl alcohol to a Pd-π-allyl intermediate as well as in the intramolecular cyclization to form a pyrrolidine framework with an α-vinyl bearing stereogenic carbon. Interesting binding features of the catalysts to the achiral *N*-sulfonamide protected 1,6-amino allyl alcohol (substrate) have been identified as (a) the presence of certain pivotal hydrogen bonding interactions between DAPCy and the N–H and O–H groups, respectively, of the sulfonamide and the alcoholic ends of the substrate and (b) the difference in the preferred π-olefin face involved in the η^2^-π-allyl coordination to the active catalyst Pd(PPh_3_)_2_ that results in stereochemically distinct *si* and *re* prochiral faces for the intramolecular nucleophilic addition. Among the various binding modes in the catalyst–substrate complex [(η^2^-π-allyl alcohol)Pd(PPh_3_)_2_], both the exo-*si* and endo-*re* configurations (where, exo and endo, respectively, refer to the *anti* and *syn* relative disposition of allyl C2–H with respect to the position of (*S*,*R*,*S*)-DAPCy anchored to the substrate) have been noted as energetically the most likely ones. The exoergic formation of the exo-*si* (−23.1) and endo-*re* (−22.2 kcal mol^−1^) complexes indicates an intrinsic thermodynamic incentive for the participation of such configurations in the catalytic cycle. Furthermore, the barrier for dehydroxylation from the exo-*si* and endo-*re* configurations has as well found to be lower than those from alternative configurations, providing a complementary kinetic advantage toward the formation of the vital Pd-π-allyl intermediate that retains the exo-*si* and endo-*re* configurations. In the enantiocontrolling intramolecular nucleophilic addition, the chiral DAPCy catalyst has been identified to serve as a phosphate counterion. Although the sense of enantioselectivity has been found to be in favor of the *S* enantiomer of the product, the extent of enantioselectivity predicted using the Gibbs free energy difference between the competing transition states for the endo-si and exo-re modes of nucleophilic addition in the case of (*S*,*S*,*S*)-DAPCy is much lower (53%) than the experimental %*ee* of 95. It therefore appears highly likely that the active diastereomer of the catalyst involved in the reaction is (*S*,*R*,*S*)-DAPCy with which the predicted %*ee* is >99. The chiral induction has been facilitated by a series of noncovalent interactions operating between the substrate and the catalysts. The interactions N–H⋯O, C–H⋯O, C–H⋯π, lone pair (lp)⋯π, O–H⋯O, O–H⋯π, and π⋯π, are noted as more efficient in the lower energy C–N bond formation transition state through the *si* prochiral face of the exo-*si* Pd-π-allyl moiety.

The energetic span analysis of the Gibbs free energy profile for the exo-*si* and endo-*re* pathways revealed that the turn-over determining intermediate is the Pd-π-allyl intermediate formed as a result of the dehydroxylation of allyl alcohol and the turn-over determining transition state is the nucleophilic addition, same as the enantiocontrolling transition state. The energetic span in the exo-*si* route toward the major enantiomer with the *S* configuration is 10.6 kcal mol^−1^ as opposed to 14.1 kcal mol^−1^ found in the endo-*re* pathway. Superior catalytic efficiency, as indicated by the energetic span, is also consistent with the quantitative conversion of the reactant obtained under room temperature reaction conditions. The energetic features of the overall catalytic cycle and the molecular insights gathered from the enantiocontrolling transition states of this chiral counterion induced enantioselectivity could serve as a rational framework for developing newer asymmetric catalytic strategies using dynamic double axially chiral phosphoric acid.

## Data availability

The Cartesian coordinates of all the computed geometries are provided in the ESI.†

## Author contributions

S. T. performed all computations. R. B. S. supervised the research. S. T. and R. B. S. wrote the manuscript.

## Conflicts of interest

The authors declare no competing financial interest.

## Supplementary Material

SC-013-D1SC05749A-s001
