# Supplementary material for: Molecular insights into chirality transfer from double axially chiral phosphoric acid in a synergistic enantioselective intramolecular amination
Source: Chem Sci. 2021 Dec 29;13(5):1323–34. doi: 10.1039/d1sc05749a (PMC8809490; doi:10.1039/d1sc05749a)
Supplement: SC-013-D1SC05749A-s001 [file SC-013-D1SC05749A-s001.pdf]

Electronic Supplementary Material (ESI) for Chemical Science.
This journal is © The Royal Society of Chemistry 2021

*Electronic Supplementary Information*

# Molecular Insights on Chirality Transfer from Double Axially Chiral Phosphoric Acid in a Synergistic Enantioselective Intramolecular Amination

Soumi Tribedi and Raghavan B. Sunoj*

Department of Chemistry, Indian Institute of Technology Bombay, Powai, Mumbai 400076, India

sunoj@chem.iitb.ac.in

**Table of Contents**


| | sections | page |
|---|---|---|
| 1 | Rotation Around the Axis of Chirality | S3 |
| 2 | Active Catalytic Species | S4 |
| 3 | Energy Calculation for the Step-by-Step Formation of **1a** | S4-S5 |
| 4 | Alternative Mechanisms for Dehydroxylation | S5-S7 |
| 5 | Analysis of intermediate **1a** | S7-S9 |
| 6 | Noncovalent Interactions in **1a**exo-*si* | S9-S11 |
| 7 | Conformations of Pd-π-Allyl in the Dehydroxylation Transition State | S11-S12 |
| 8 | Analysis of Dehydroxylation Transition States | S12-S14 |
| 9 | The π–σ–π Interconversion | S14-S16 |
| 10 | Alternative Mechanism of Intramolecular Nucleophilic Addition | S16 |
| 11 | Role of *in-situ* generated water molecule | S16-S17 |
| 12 | The Energy Profile Diagram for All The Pathways Considered | S17-S18 |
| 13 | Activation Strain Analysis of Enantiocontrolling TSs | S18-S19 |
| 14 | Espinosa Quantification of Noncovalent Interactions Stereocontrolling TSs | S19-S21 |
| 15 | Calculation of Free Energy Change in Each Step | S22 |
| 16 | Details of the Energetic Span Analysis | S22-S23 |
| 17 | Optimized Cartesian Coordinates of All the Stationary Points | S23-283 |

## 1. Rotation Around the Axis of Chirality

The barrier for rotation around the axis of chirality in **DAPCy** is 13.0 kcal/mol, which could readily be deemed flexible at room temperature. To place this barrier in a better perspective, herein we compare the barrier for rotation in an axially chiral BINOL-derived phosphoric acid **B** (37.5 kcal/mol) and that in a hypothetical chiral phosphoric acid **A** (11.1) bearing an angular type inter-napthyl linkage. It can be noted that **A** has a similar backbone structure as **DAPCy** except for the substituents, which form the outer chiral axes (Figure S1).

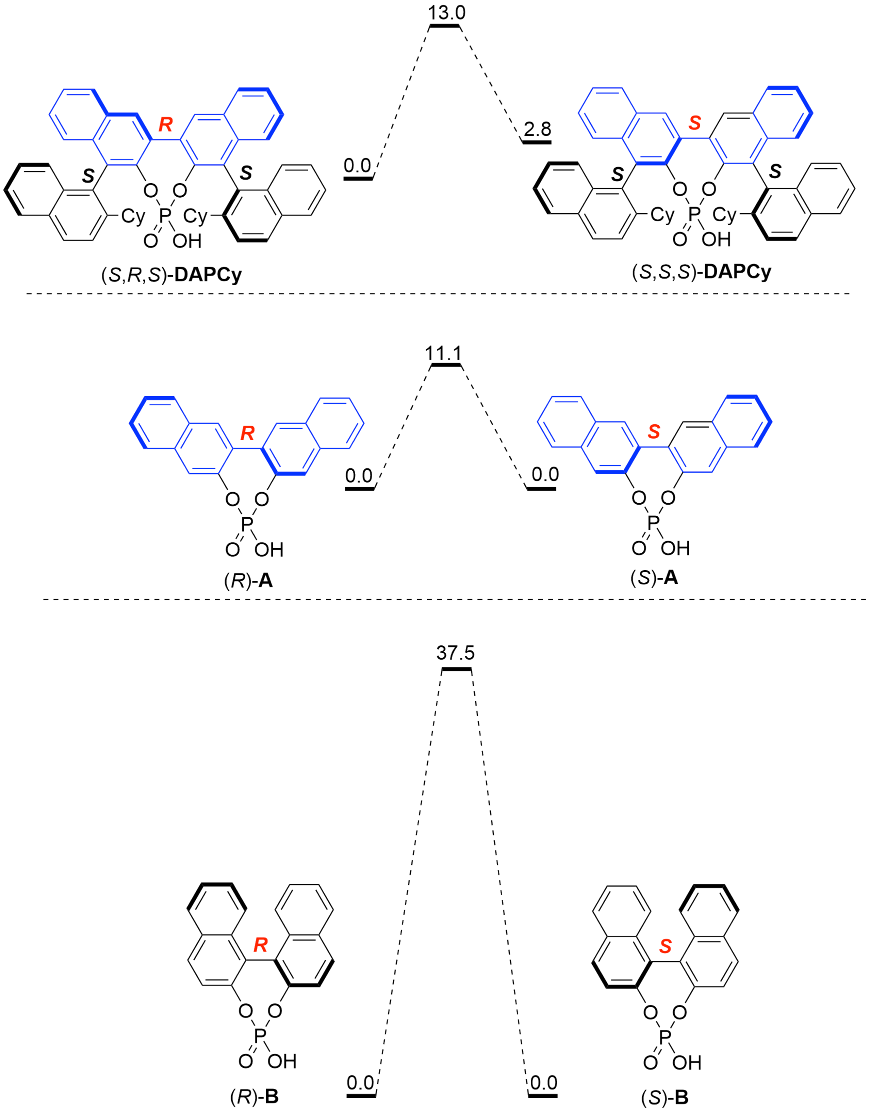


**Figure S1.** The barrier of rotation for axis of chirality in a hypothetical derivative of **DAPCy** (**A**), and a BINOL-derived chiral phosphoric acid (**B**). The relative Gibbs free energies are reported in kcal/mol.

## 2. Active Catalytic Species

Three key possibilities of the catalyst-substrate complexes, denoted as **1a**, **1b**, and **1c** are considered (as shown in Figure 1 in the manuscript), along the lines of our previous work on the dual catalytic Tsuji-Trost reaction involving $Pd(PPh_3)_4$ and chiral phosphoric acid.[1] The species **1a**, consisting of a π-$Pd(PPh_3)_2$ interaction between the substrate and the transition metal catalyst and a set of noncovalent interactions with the phosphoric acid in the outer-sphere, is found to be energetically the most preferred. The **1b** and **1c** modes contain one native triphenylphosphine ligand on the palladium and they differ in the mode of phosphoric acid interaction. In **1b**, the phosphoric acid is coordinated to palladium with a Pd–O distance of 2.48 Å and it is 21.4 kcal/mol higher in energy than the most preferred bis-phosphine complex **1a**. In **1c**, the phosphoric acid is hydrogen bonded to the oxygen of the allyl alcohol and is attached to the palladium-substrate complex via other noncovalent interactions. This mode is also higher by 28.8 kcal/mol than **1a**. Since these three models are stoichiometrically different, the energetic comparison is carried out by careful mass balance by way of including the energy of triphenylphosphine to **1b** and **1c** and applying the standard-state corrections at 1 mol/L concentration and 298.15 K temperature (Table S1).

**Table S1.** The Stoichiometrically Balanced Equations for Calculation of the Gibbs Free Energies for the Formation of **1a**, **1b** and **1c**

| **Reaction** | $\Delta G_r^0$ (kcal/mol) |
|---|---|
| $Pd(PPh_3)_4$ + allyl alcohol + CPA = **1a** + 2$PPh_3$ | -17.7 |
| $Pd(PPh_3)_4$ + allyl alcohol + CPA = **1b** + 3$PPh_3$ | 3.7 |
| $Pd(PPh_3)_4$ + allyl alcohol + CPA = **1c** + 3$PPh_3$ | 11.1 |

## 3. Stoichiometrically Balanced Energies for the Step-by-Step Formation of 1a

(1) Jindal, G.; Sunoj, R. B. *J. Org. Chem.* **2014**, *79*, 7600–7606.

The catalyst-substrate complex **1a** can be formed from $Pd(PPh_3)_4$, **DAPCy**, and the allyl alcohol, by dissociation of two $PPh_3$ ligands from the precatalyst so that the allyl alcohol can bind through a $\eta^2$-$\pi$-bond. The formation of **1a** is found to be quite exoergic ranging from -23.1 till -15.8 kcal/mol for various configurations (exo-*si*, endo-*re*, endo-*si* and exo-*re* configurations of **1a** as shown in Figure 2a in the manuscript). The electronic energy change in the step-by-step process of formation of **1a**endo-*si* from the separated reactants is given below. It may be noted that equations below involve a hypothetical species $Pd(PPh_3)_2$, which is not a minima. Hence, electronic energies are used for stoichiometric balance. However, the data in Figure 2a and in Table S1 are Gibbs free energies, where a comparison of the ΔG of formation of **1a** with that of **1b** and **1c** is provided. Here, the stoichiometric equations for calculating the energy of formation of one of the **1a** configurations (endo-*si*) in a stepwise manner is provided.

$Pd(PPh_3)_4$ + allyl alcohol + CPA (0 kcal/mol) = **1a** $[(PPh_3)_2Pd$-allylOH]…[CPA] ….(1)

(ΔE = -21.4 kcal/mol) : energy of formation of **1a**endo-*si* from separated reactants balanced stoichiometrically

**Step 1:**

$Pd(PPh_3)_4$ = $Pd(PPh_3)_2$ + $2PPh_3$ …. (2) ; ΔE = +85.3 kcal/mol

**Step 2:**

$Pd(PPh_3)_2$ + allyl alcohol = $(PPh_3)_2Pd$-allylOH …. (3) ; ΔE = -44.8 kcal/mol

**Step 3:**

$(PPh_3)_2Pd$-allylOH + CPA = $[(PPh_3)_2Pd$-allylOH]…[CPA] …. (4) ; ΔE = -70.2 kcal/mol

From equations (2), (3) and (4), equation (1) is obtained,

$Pd(PPh_3)_4$ + allyl alcohol + CPA (0 kcal/mol) = **1a** $[(PPh_3)_2Pd$-allylOH]…[CPA]

Where, the total additive change ΔE = -70.2 – 44.8 + 85.3 = -29.7 kcal/mol

## 4. Alternative Mechanisms for Dehydroxylation

The higher energy catalyst-substrate complexes such as **1b** and **1c** are considered in the dehydroxylation step. In **1b**, the **DAPCy** bound to the Pd protonates the O of the alcohol and subsequently assists the elimination of $H_2O$ with the formation of the corresponding $\eta^3$-allyl intermediate **2b** via **[1b-2b]**$^‡$ (Scheme S1).

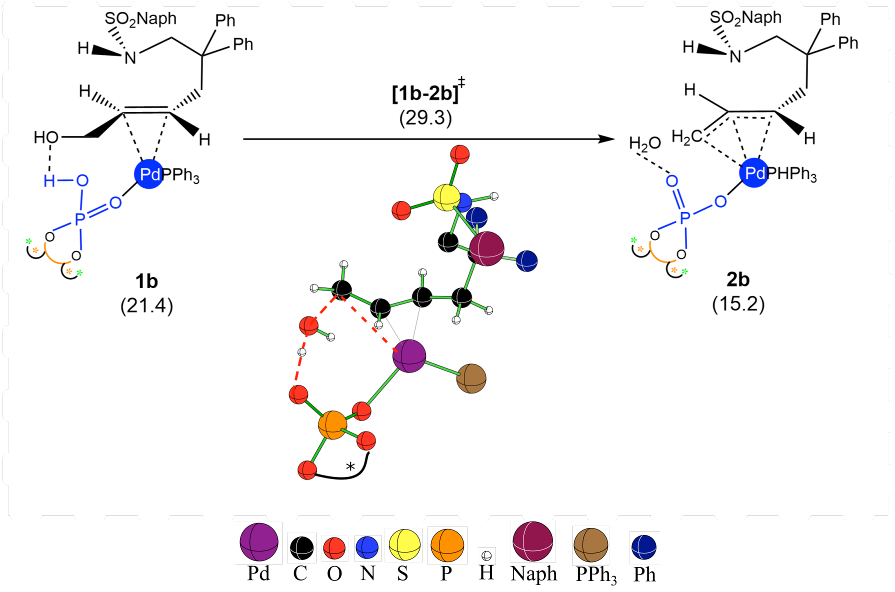


**Scheme S1.** The dehydroxylation from **1b** by the Pd-bound **DAPCy** to form **2b** via **[1b-2b]**‡. The relative free energies with respect to **1a** is given in parentheses in kcal/mol.

The **DAPCy** in **1c** is in the outersphere and it protonates the hydroxyl group of the substrate with a concomittant elimination of water to form **2c**, where a counterion **DAPCy-ate** remains bound to the Pd-π-allyl intermediate (Scheme S2).

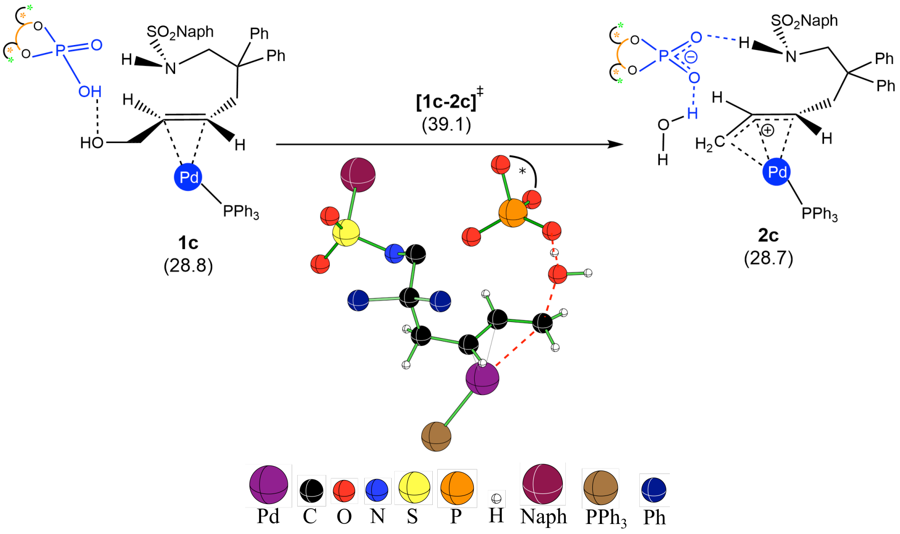


**Scheme S2.** The dehydroxylation from **1c** by noncovalently bound **DAPCy** to form **2c** via **[1c-2c]**‡. The relative free energies with respect to **1a** is given in parentheses in kcal/mol.

Another mechanism of dehydroxylation in the absence of chiral **DAPCy** is exploredparticipate in an autocatalytic manner. The faster dehydroxylation in this fashion in the absence of **DAPCy** would lead to equal proportions of *re* and *si* **2a** intermediates. One may propose

the involvement of chiral catalyst only in the second step (intramolecular nucleophilic addition in **2a**) which is deemed the enantiocontrolling step. An important aspect to be noted here is that the interaction energy between **DAPCy** and $\eta^2$-$\pi$-allyl alcohol-Pd($PPh_3$)$_2$ in **1a** is considerably large (Table S2). It is therefore unlikely that the hydronium ion can displace the regenerated **DAPCy** from the product-catalyst complex at the end of the catalytic cycle so as to sustain it through promoting dehydroxylation of the next molecule of allyl alcohol Thus, this alternative mechanism unlikely to operate.

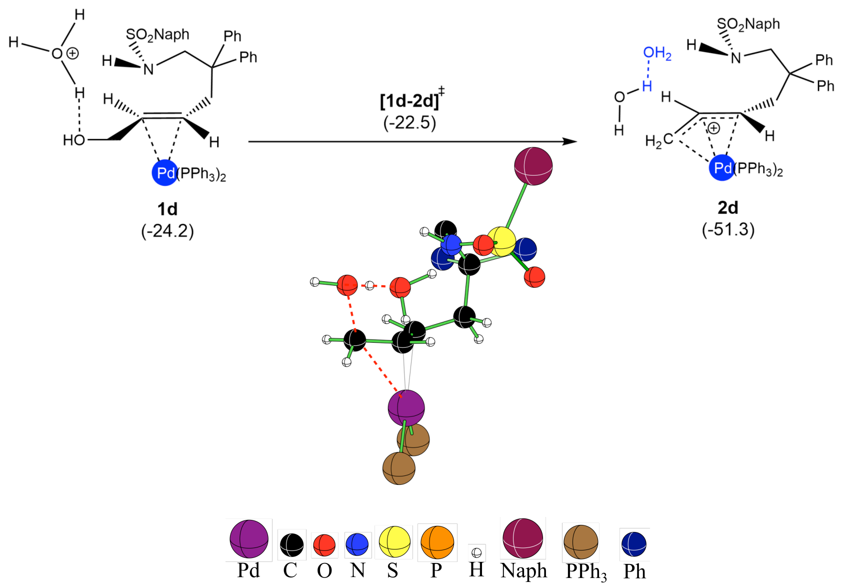


**Scheme S3.** The dehydroxylation from **1d** by hydronium ion to form **2d** via **[1d-2d]**$^{\ddagger}$. The relative free energies with respect to the corresponding **1a** is given in parentheses in kcal/mol.

## 5. Analysis of 1a

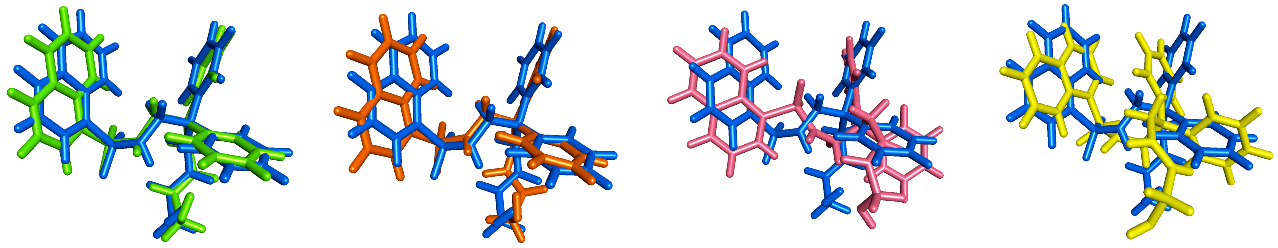

| **1a**endo-*re* | **1a**exo-*si* | **1a**endo-*si* | **1a**exo-*re* |
|---|---|---|---|
| RMSD = 0.70 | 1.09 | 3.12 | 3.56 |
| $\Delta E_{dis}$ = 10.5 | 9.1 | 17.2 | 9.6 |

**Figure S2.** Superimposed geometries of the distorted allyl alcohols taken from the respective **1a** intermediates overlaid on the minimum energy geometry of the *trans*-allyl alcohol (blue). RMSDs

of all atoms are in angstrom unit. Distortion energies are given in kcal/mol.

**Table S2.** The Relative Gibbs Free Energies, Interaction Energies Between **DAPCy** and Pd-alcohol, and, Binding Energies Between $Pd(PPh_3)_2$ and allyl alcohol in the **1a** intermediates

| | **ΔΔG** | **$\Delta E_{int}$ [DAPCy…Pd-alcohol]** | **$\Delta E_{bind}$ [$Pd(PPh_3)_2$…alcohol]** |
|---|---|---|---|
| **1a**endo-*re* | 0.9 | -67.2 | -53.4 |
| **1a**exo-*si* | 0.0 | -68.3 | -50.9 |
| **1a**endo-*si* | 5.4 | -70.2 | -52.6 |
| **1a**exo-*re* | 7.3 | -59.1 | -55.7 |

The catalyst-substrate configurations **1a**endo-*re* and **1a**exo-*si* are both thermodynamically stable and are quite close in energy (differing only by 0.9 kcal/mol). In contrast, the other two configurations **1a**endo-*si* and **1a**exo-*re* are higher by 5.4 and 7.3 kcal/mol respectively (Table S2). In order to understand the origin of these energy differences between these configurations, the geometries of these complexes were subjected to the *activation strain*-like analysis. It is interesting to note that the interaction between **DAPCy** and Pd-alcohol, which is solely controlled by NCIs is much higher in all the configurations, than that between Pd and alcohol which is a $\eta^2$-π bond. The interaction between **DAPCy** and Pd-alcohol is lowest in **1a**exo-*re*, (by 8.1 kcal/mol from the next lower) which explains its highest relative Gibbs free energy among the **1a** intermediates. **1a**endo-*si*, however has the highest interaction (-70.2 kcal/mol) but has higher Gibbs free energies than **1a**endo-*re* and **1a**exo-*si*. Analyzing the distortion energies, we find that the alcohol geometries are closer to the optimized minimum in **1a**endo-*re*, **1a**exo-*si* and **1a**exo-*re* while it is more distorted in **1a**endo-*si* (Figure S2). Further careful inspection reveal a particular intramolecular C–H···O interaction in both the lower energy configurations (**1a**endo-*re* and **1a**exo-*si*) between an oxygen atom of the $–NSO_2Ar$ group and the C2–H or C3–H, which is missing in the other two (Figure S3).

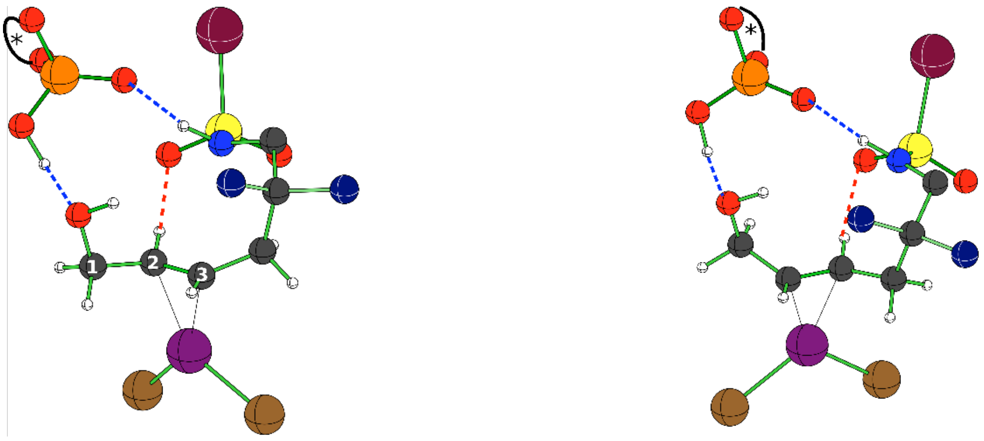


**1a**endo-*re* **1a**exo-*si*

**Figure S3.** The geometries of **1a**endo-*re* and **1a**exo-*si* with the important intramolecular C–H···O interaction marked in red dotted lines.

## 6. Noncovalent Interactions in 1aexo-*si*

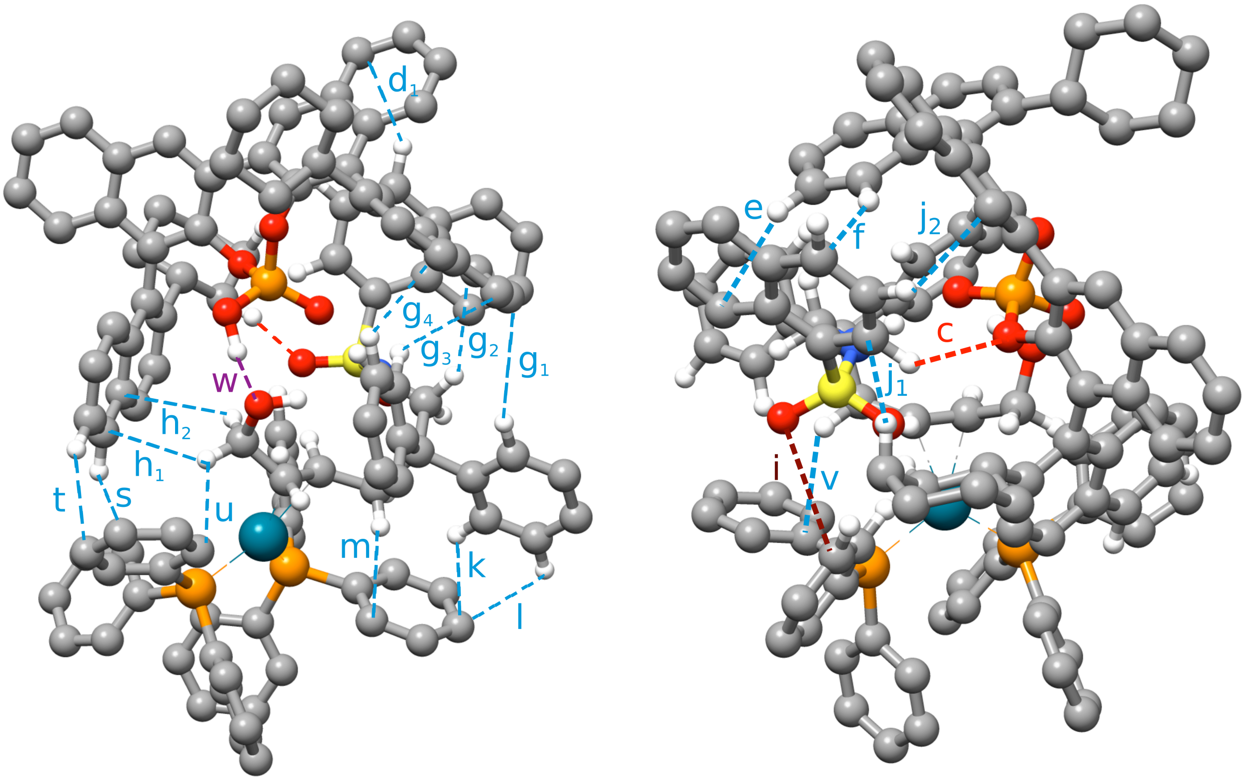

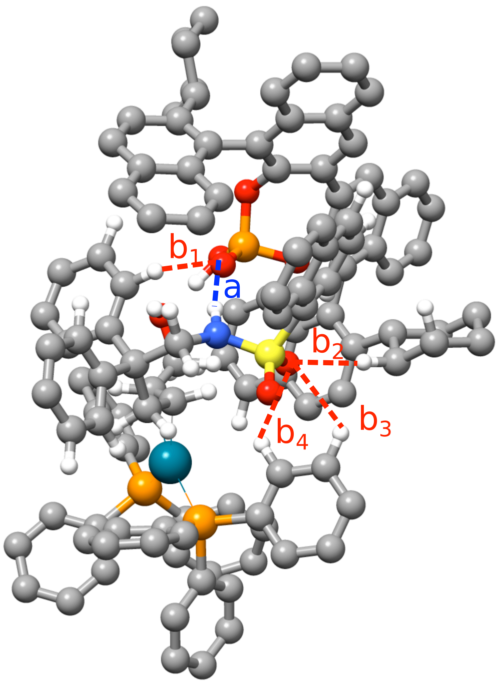


**Figure S4.** The noncovalent interactions as obtained from AIM analysis of **1a**exo-*si* in three different orientations, marked with dotted lines and labelled. Color codes: red (C–H···O); blue (N–H···O); sky (C–H···π); purple (O–H···O).

**Table S3.** The Distances and Electron Densities of the (3,-1) Bond Critical Points Identified Through AIM Analysis of **1a**exo-*si*

| type | label | distance (Å) | $\rho \times 10^{-2}$ |
|---|---|---|---|
| | **Phosphate…Allyl** | | |
| C–H···O | $b_1$ | 2.46 | 1.05 |
| | $b_2$ | 2.31 | 1.34 |
| | c | 2.39 | 1.12 |
| N–H···O | a | 1.79 | 3.36 |
| C–H···π | $g_1$ | 3.24 | 0.28 |
| | $g_2$ | 2.79 | 0.67 |
| | $g_3$ | 3.09 | 0.47 |
| | $g_4$ | 2.79 | 0.67 |
| | $h_1$ | 3.05 | 0.46 |
| | $h_2$ | 3.08 | 0.38 |
| | e | 2.67 | 0.85 |
| | f | 2.85 | 0.59 |
| | $d_1$ | 3.30 | 0.27 |
| | $j_1$ | 3.08 | 0.39 |
| | $j_2$ | 2.70 | 0.78 |
| O–H···O | w | 1.46 | 0.86 |
| | **Phosphine…Allyl** | | |

| | | | |
|---|---|---|---|
| C–H···π | u | 2.66 | 0.79 |
| | m | 3.04 | 0.44 |
| | v | 2.92 | 0.55 |
| | k | 2.77 | 0.70 |
| | l | 3.00 | 0.44 |
| C–H···O | $b_3$ | 2.58 | 0.83 |
| | $b_4$ | 2.54 | 0.90 |
| lp(O)···π | i | 3.45 | 0.43 |
| **Phosphate…Phosphine** | | | |
| C–H···π | s | 2.73 | 0.68 |
| | t | 2.82 | 0.60 |

## 7. Conformations of Pd-Allyl in the Dehydroxylation Transition State

The transition state for the first step in the mechanistic cycle, i.e., dehydroxylation, is optimized at various conformational variations of the substrate. The dihedral angles of the *trans* substrate that are considered in the conformational sampling of the dehydroxylation transition state responsible for the formation of the *endo* intermediate (shown as a representative example) is given in Figure S5 and the relative free energies of such conformations are given in Table S3.

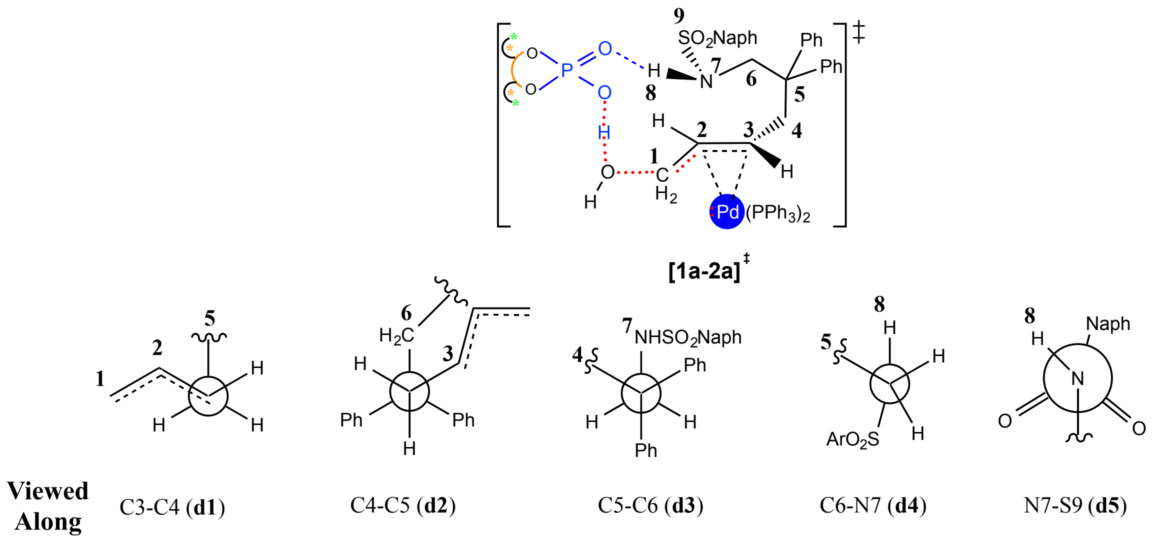


**Figure S5**. A schematic representation of the key dihedral angles considered for conformational sampling of the dehydroxylation transition state leading to the *endo* Pd-π-allyl intermediate.

We note that the Gibbs free energies of these conformers are more sensitive to two key dihedral angles (**d2** and **d5**, in Figure S5). This is expected, since relatively bulkier groups are involved in these. In the case of **d2**, **3**, which is Pd-η3-allyl part and **6**, which is the –

$CH_2NHSO_2$Naph tend to produce lower energy conformations when kept farther apart (Table S4). On the other hand, the naphthyl group and the rest of the substrate chain attached to N, tend to remain anti to each other on changing the dihedral **d5**.

**Table S4**. Relative Gibbs Free Energies (in kcal/mol) with respect to the Lowest Energy Dehydroxylation Transition States (See Figure S5 for details of the dihedral angles)

| | d1 | d2 | d3 | d4 | d5 | ΔΔG |
|---|---|---|---|---|---|---|
| *re*-face | | | | | | |
| | 52.5 | -100.5 | 43.2 | 77.7 | 50.9 | -3.8 |
| | 80.3 | -101.5 | 47.7 | 90.8 | 56.9 | -4.2 |
| endo-*re* | 77.1 | -101.6 | 47.1 | 98.8 | 67.0 | -3.6 |
| | 92.4 | 35.3 | -61.0 | -48.2 | 143.5 | 27.6[a] |
| | 91.4 | -80.4 | 169.8 | -113 | -143.5 | 15.3 |
| exo-*re* | 20.4 | 71.6 | 48.4 | -104.5 | 94.4 | 6.2 |
| | 94.4 | -87.7 | 52.3 | 40.9 | 79.0 | 6.5 |
| *si*-face | | | | | | |
| exo-*si* | -80.3 | -79.0 | 52.2 | 34.4 | 125.5 | 15.6 |
| | -97.9 | -90.5 | 57.9 | 37.1 | 150.7 | 12.6 |
| | -80.5 | -74.5 | 51.9 | 80.8 | 61.2 | -3.2 |
| endo-*si* | -89.3 | 85.3 | -174.7 | 84.3 | -129.5 | 11.9 |
| | -91.4 | 89.0 | -48.7 | -52.2 | 77.6 | 7.8 |
| | -106.8 | 85.6 | -56.1 | 88.4 | 69.2 | 3.5 |
| | -111.6 | 83.6 | -54.1 | 79.6 | -161.9 | 6.0 |
| | -87.3 | 94.1 | -45.2 | -118.2 | 38.1 | 16.0 |
| | -98.5 | 75.7 | -71.5 | 121.1 | 32.7 | 7.9 |
| | -79.0 | 75.4 | 42.1 | -132.1 | -107.5 | 1.2 |
| | -75.6 | -178 | -172.7 | -65.2 | 151.3 | 19.8 |
| | -109.2 | -50.1 | 84.6 | -13.5 | 56.5 | 10.2 |
| | -83.1 | 74.5 | 46.9 | -117.6 | -30.9 | **0.0** |

[a] one of the $PPh_3$ decoordinates from the Pd center

## 8. Analysis of Dehydroxylation Transition States

**Table S5.** The Distortion Energies of Various Fragments in the Dehydroxylation Transition States Calculated with respect to the Corresponding Fragments in the Lowest Energy **[1a-2a]**$^{\ddagger}$endo-*re*

| Fragments | | **[1a-2a]**$^{\ddagger}$exo-*si* | **[1a-2a]**$^{\ddagger}$exo-*re* | **[1a-2a]**$^{\ddagger}$endo-*si* |
|---|---|---|---|---|
| **DAPCy-ate** | 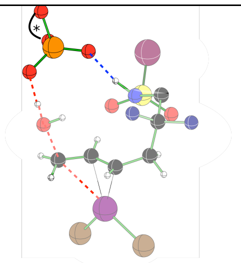 | 0.0 | 1.7 | 3.2 |

| | | | | |
|---|---|---|---|---|
| [Pd-allyl alcohol-H]$^+$ | 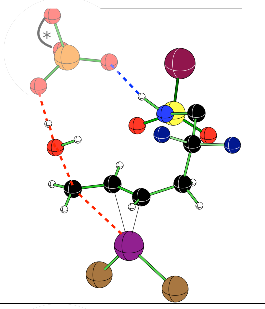 | -2.0 | 13.2 | 16.0 |
| [allyl alcohol-H]$^+$ | 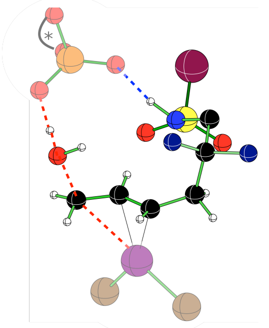 | -1.3 | 11.3 | 16.4 |

The relative free energies of the dehydroxylation TSs suggest that the exo-*re* pathway ($\Delta G^{\ddagger}$ = -4.1 kcal/mol) is quite unlikely as compared to that of the other configurations. To rationalize the potential origin of the higher energy of the exo-*re* configuration, we have examined the geometries and other electronic structure features of the relevant TSs. The relative free energies of the dehydroxylation **[1a-2a]**$^{\ddagger}$ in the exo-*si* (1.1), endo-*si* (4.3), and exo-*re* (10.4) configurations are higher as compared to **[1a-2a]**$^{\ddagger}$endo-*re* (0.0) (Table 1 in the main manuscript). Like the catalyst-substrate complex **1a**, configurations endo-*re* and exo-*si* are energetically more feasible, whereas the other two configurations **1a**endo-*si* and **1a**exo-*re* are higher by 4.3 and 10.4 kcal/mol respectively.

The interaction energies between **DAPCy-ate** and [Pd-allyl alcohol-H]$^+$ fragments (where the proton involved in the reaction coordinate is considered as fully transferred to the alcohol and hence is part of the Pd-allyl alcohol fragment) are found to be -102.0, -98.4, -116.2 and, -107.5 kcal/mol respectively in **[1a-2a]**$^{\ddagger}$endo-*re*, exo-*si*, endo-*si* and, exo-*re*. It is interesting to note that the endo-*si* and exo-*re* exhibit better interaction making the trend opposite to that of the relative free energies. The distortion energies with respect to the most preferred **[1a-2a]**$^{\ddagger}$endo-*re* (Table S5) indicate the presence of higher distortions in [allyl alcohol-H]$^+$ and [Pd-allyl alcohol-H]$^+$ fragments in the endo-*si* and exo-*re* configurations of **[1a-2a]**$^{\ddagger}$ whereas the corresponding distortions in endo-

*re* and exo-*si* are found to be reasonable. This lead us to infer that to maximize the NCIs between **DAPCy-ate** and [Pd-allyl alcohol-H]$^{+}$, the allyl alcohol assumes highly distorted conformations in the higher energy **[1a-2a]**$^{\ddagger}$endo-*si* and **[1a-2a]**$^{\ddagger}$exo-*re*.

This can also be gleaned from the root mean square deviation (RMSD) calculated with respect to the geometry of the [allyl alcohol-H]$^{+}$ fragment as seen in the lowest energy **[1a-2a]**$^{\ddagger}$endo-*re* (Figure S6). While both of the higher energy endo-*si* and exo-*re* configurations bear high distortions, the stabilizing interaction energy in endo-*si* considerably offsets the destabilizing distortion. The net effect of the lower interaction energy and high distortion in the exo-*re* configuration therefore renders it the least preferred.

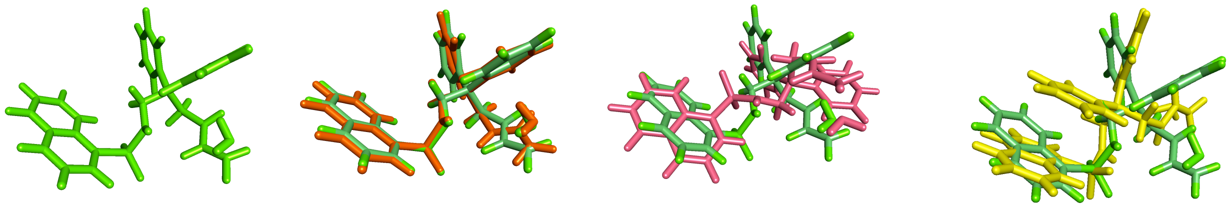

| **[1a-2a]**$^{\ddagger}$endo-*re* | **[1a-2a]**$^{\ddagger}$exo-*si* | **[1a-2a]**$^{\ddagger}$endo-*si* | **[1a-2a]**$^{\ddagger}$exo-*re* |
|---|---|---|---|
| RMSD = 0.00 | 0.64 | 4.75 | 4.43 |

**Figure S6.** Superimposed geometries of the protonated allyl alcohol taken from the respective transition states and the corresponding images overlaid on the lowest energy **[1a-2a]**$^{\ddagger}$endo-*re* (green). RMSDs of all atoms are in angstrom unit.

### 9. The π–σ–π Interconversion

(a) **2a'**exo-*re* ↔ **2a'**endo-*si*

An interconversion from *re* to *si* from the Pd-π-allyl intermediate **2a** is considered via a π–σ–π TS **[re-si]**$^{\ddagger}$. In this TS, the C1 is bonded to the Pd by a σ-bond and the reaction coordinate features a rotation along the C1-C2 bond, the IRC (intrinsic reaction coordinate) of which is connected to intermediates **2a'** (also σ-bonded) where either the *re* or *si* face is exposed for subsequent nucleophilic addition. Each of the **2a'**exo-*re* and **2a'**endo-*si* intermediates are further connected to the corresponding **2a** (Scheme S4).

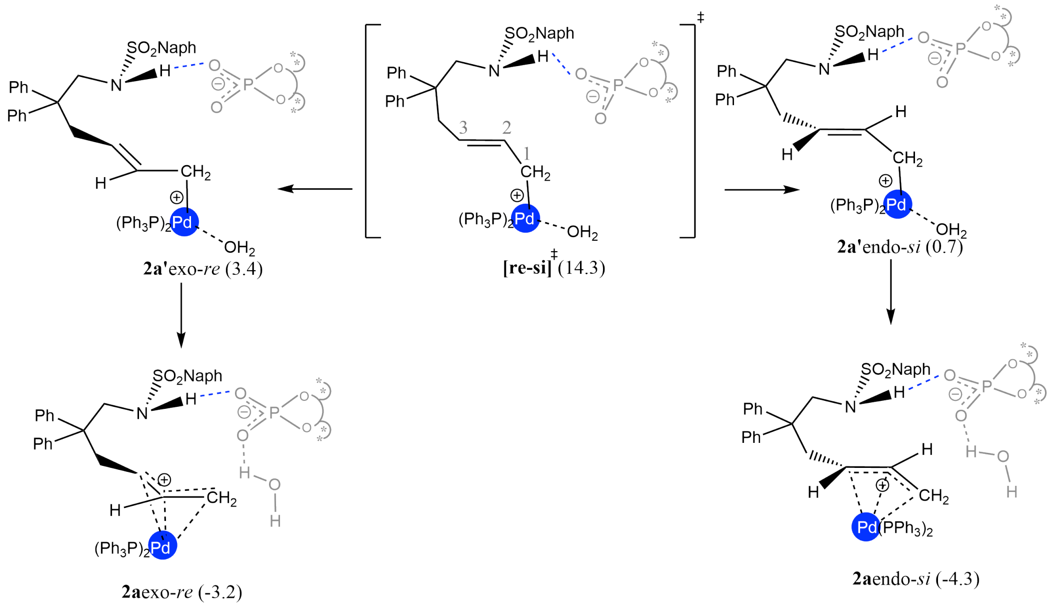


**Scheme S4.** The interconversion of **2a'**exo-*re* to **2a'**endo-*si* via π–σ–π transition state. Relative Gibbs free energies are in kcal/mol with respect to **1a**exo-*si*.

(b) **2a''***si*(*trans*) ↔ **2a''***si*(*cis*)

The dehydroxylation of *trans*-allyl alcohol leads to intermediate **2a** where an s-*trans* disposition is maintained across the C2-C3 bond (Scheme S5). A σ-bond rotation across the C2-C3 bond is considered from intermediate **2a''***si*(*trans*) via transition state ***si*[*trans-cis*]**‡, where C3 is bound to the Pd by a σ bond, making the *si* prochiral face available for the nucleophilic addition. A rotation of this sort can result in an s-*cis* intermediate **2a''***si*(*cis*), where the prochiral face remains the same. Similar TS for the *re* face is also located (Scheme S6).

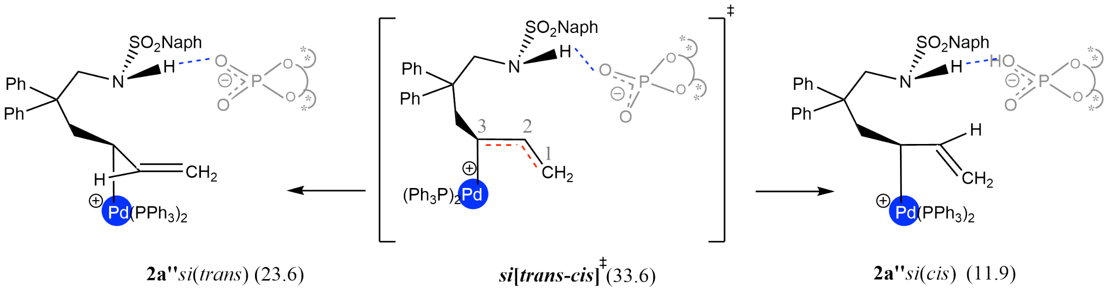


**Scheme S5.** The interconversion of s-*trans* intermediate **2a''***si*(*trans*) to s-*cis* **2a''***si*(*cis*) via a σ-rotation transition state. Relative Gibbs free energies are in kcal/mol with respect to **1a**exo-*si*.

(c) **2a''***re*(*trans*) ↔ **2a''***re*(*cis*)

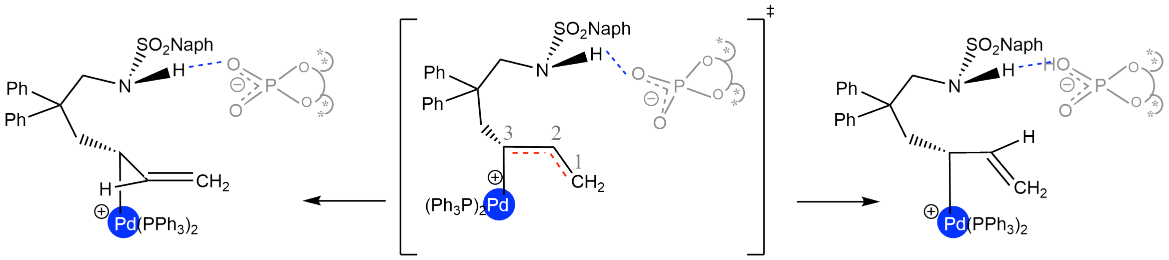


**2a''**$re$($trans$) (24.3) **$re$[$trans$-$cis$]**$^{‡}$(30.4) **2a''**$re$($cis$) (27.2)

**Scheme S6.** The interconversion of s-*trans* intermediate **2a''***re*(*trans*) to s-*cis* **2a''***re*(*cis*) via a σ-rotation transition state. Relative Gibbs free energies are in kcal/mol with respect to **1a**exo-*si*.

## 10. Alternative Mechanism of Intramolecular Nucleophilic Addition

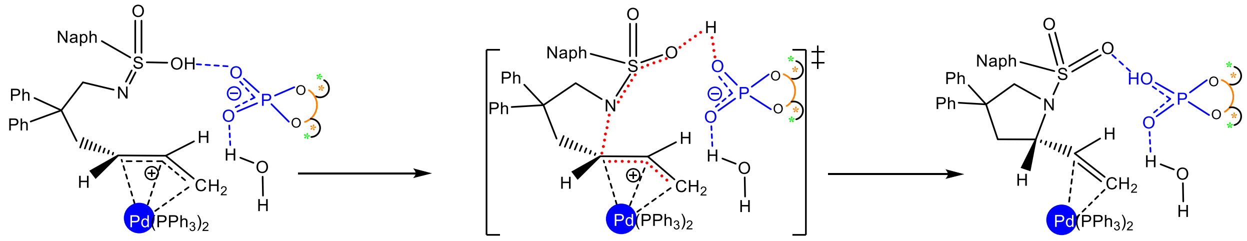


**2a*** **[2a*-3a*]**$^{‡}$ **3a***

**Scheme S7.** The alternative nucleophilic addition pathway where the N–H is abstracted by the **DAPCy-ate** prior to cyclization.

## 11. Role of *in-situ* generated water molecule

After the dehydroxylation, the eliminated water molecule stays hydrogen-bonded to the rest of the system. There is no apparent role for the water molecule other than in the activation of the *trans*-allyl alcohol to Pd-π-allyl. However, the elementary step barrier for the most favored intramolecular nucleophilic transition state is found to be lowered by 6.4 kcal/mol when the hydrogen-bonded water was retained. Owing to this kinetic advantage, we propose the TS models including the hydrogen-bonded water molecule.

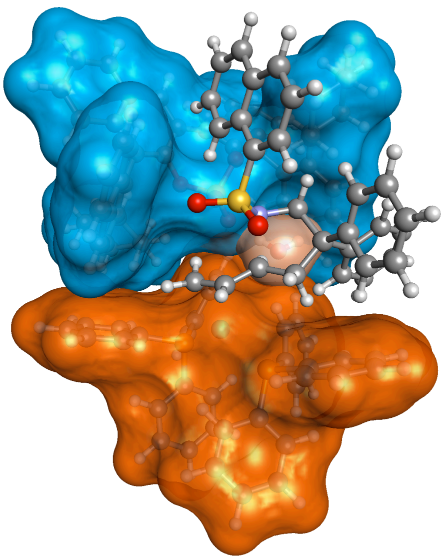

**[2a-3a]**$^{‡}$exo-*si* (-19.2)

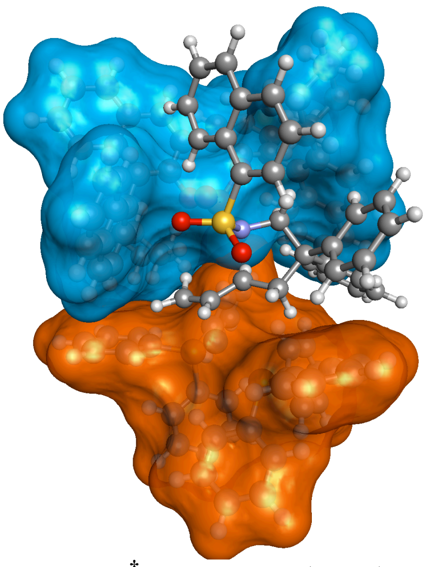

**[2a-3a]**$^{‡}$exo-*si-no-w* (-12.8)

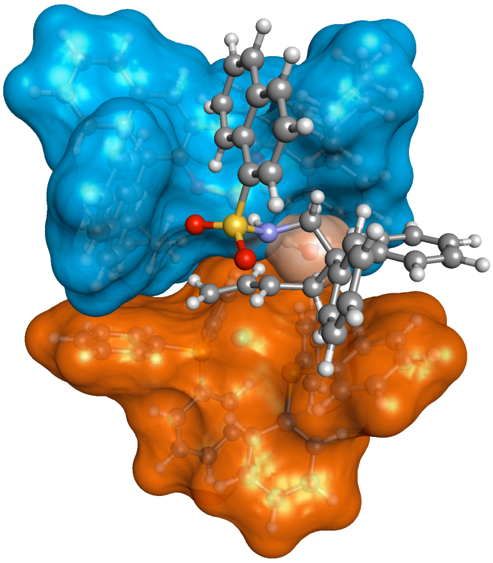

**[2a-3a]**$^{‡}$endo-*re* (-15.2)

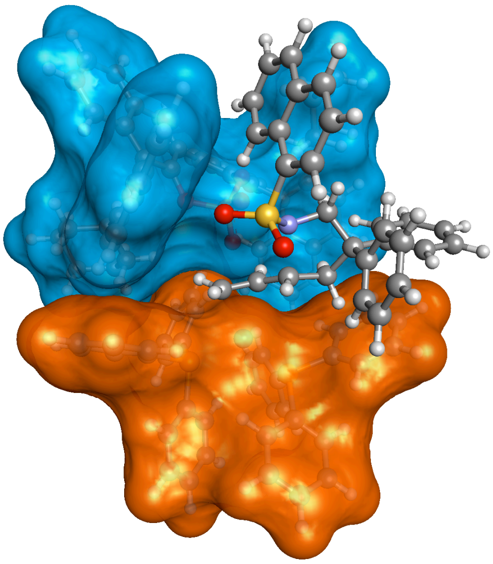

**[2a-3a]**$^{‡}$endo-*re-no-w* (-7.2)

**Figure S7.** The space-filling model of the diastereomeric transition states for the nucleophilic addition in the presence and absence of water molecule. Relative Gibbs free energies are given in kcal/mol. Color codes: red: $Pd(PPh_3)_2$; blue: **DAPCy-ate**; pink: water.

## 12. The Energy Profile Diagram for All The Pathways Considered

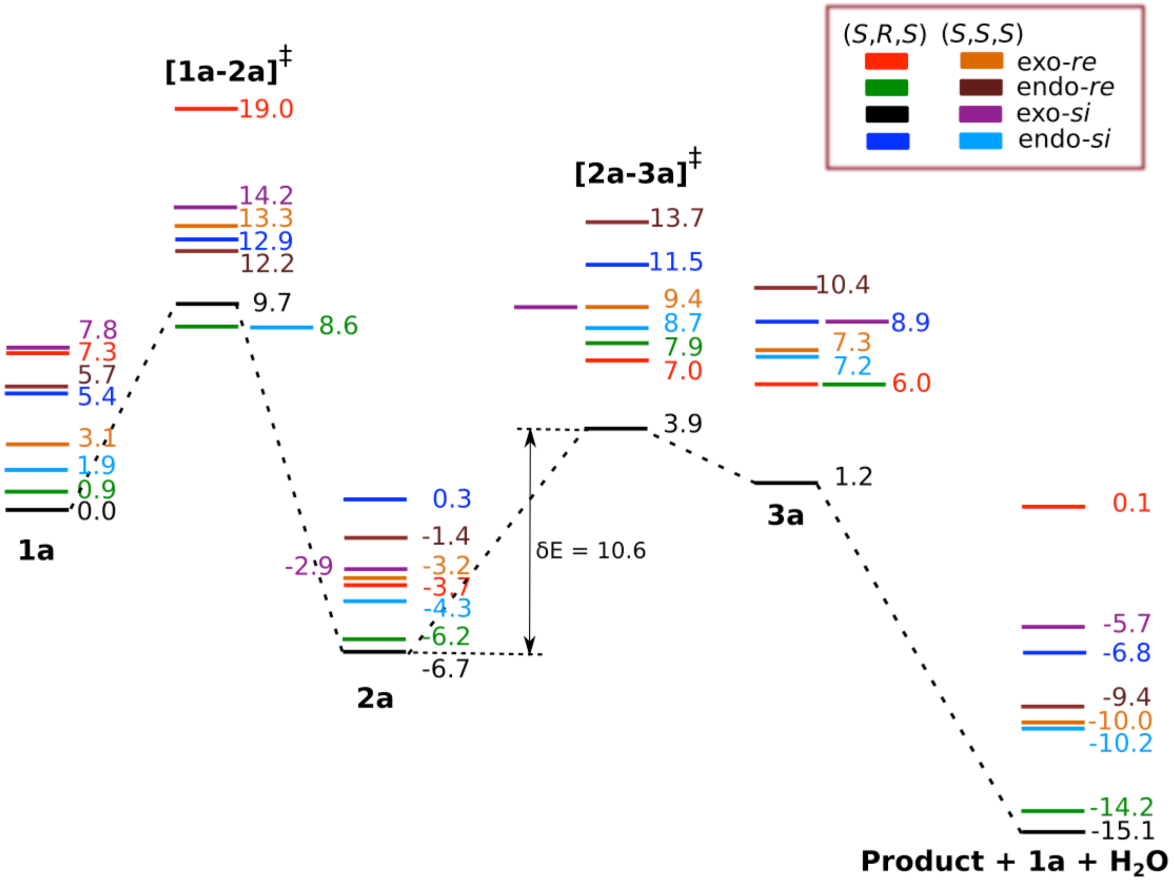


**Figure S8.** Gibbs free energy profile for all the pathways of allylic amination catalyzed by (*S*,*S*,*S*) or (*S*,*R*,*S*) diastereomers of **DAPCy**. Energies are in kcal/mol.

## 13. Activation Strain Analysis of Enantiocontrolling TSs

The distortion energies are calculated by subtracting the energies of particular fragments in the higher energy **[2a-3a]**‡endo-*re* from the corresponding energies of the same fragments in the lower energy **[2a-3a]**‡exo-*si*. This is so calculated as one is only concerned about how much more or less distorted these TSs are with respect to each other. As can be noticed from the data given in Table S6, the distortion of various fragments of **[2a-3a]**‡endo-*re* very similar to that in **[2a-3a]**‡exo-*si*. The most distorted fragment (by 2.4 kcal/mol) is F2 in approach 2, which is the Pd-π-allyl and water combined. Water has better interactions with **DAPCy-ate** than with Pd-π-allyl in both TSs (from Espinosa formulation, interactions in endo-*re*: [water...Pd-π-allyl] = -25.2 kcal/mol and [water...**DAPCy-ate**] = -27.7 kcal/mol, and exo-*si*: [water...Pd-π-allyl] = -28.0 kcal/mol and [water...**DAPCy-ate**] = -29.3).

**Table S6.** The Activation Strain Analysis on the Diastereomeric TSs ($\Delta\Delta E_x = \Delta E_x$((*S,R,S*)-**[2a-3a]**$^{\ddagger}$endo-*re*) – $\Delta E_x$((*S,R,S*)-**[2a-3a]**$^{\ddagger}$exo-*si*)) in kcal/mol)

| | $\Delta\Delta E_{dis}$(F1) | $\Delta\Delta E_{dis}$(F2) | $\Delta\Delta E_{dis}$(F3) | $\Delta\Delta E_{int}$ |
|---|---|---|---|---|
| **Approach 1**<br>F1 = Phosphate + water<br>F2 = Pd-π-allyl | -0.8 | -0.1 | -- | 5.0 |
| **Approach 2**<br>F1 = Phosphate<br>F2 = Pd-π-allyl + water | -0.1 | 2.4 | -- | 1.9 |
| **Approach 3**<br>F1 = Phosphate<br>F2 = Pd-π-allyl<br>F3 = water | -0.1 | -0.1 | -0.1 | 4.5 |

The Figure S9 shown below depicts individual distortion of fragments (a) **DAPCy**-ate and (b) Pd-π-allyl in the two TSs.

(a) (b)

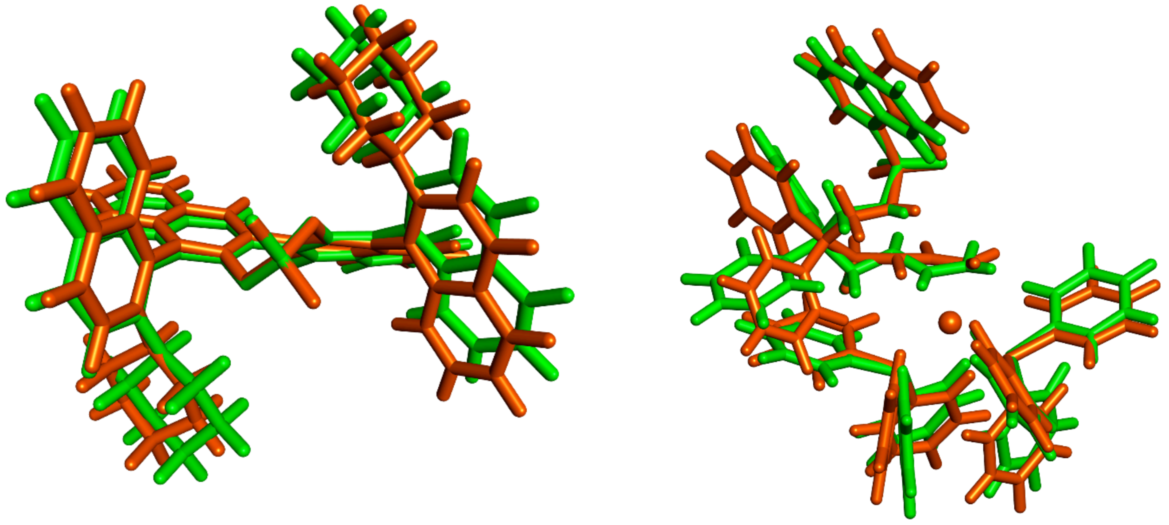

**Figure S9.** The superimposed images of the geometries of the fragments (a) **DAPCy**-ate, and (b) Pd–π–allyl as present in **[2a-3a]**$^{\ddagger}$endo-*re* (green) and **[2a-3a]**$^{\ddagger}$exo-*si* (red).

## 14. Espinosa Quantification of Noncovalent Interactions Stereocontrolling TSs

Espinosa's formulation is employed that relies on the electron density at the bond critical point ($\rho_{bcp}$), the corresponding Laplacian of the electron density ($\nabla^2\rho$), and kinetic energy (G) toward the quantification of the overall strength of the NCIs (Table S7).[2]

**Table S7.** Estimated Strengths of the Important Noncovalent Interactions in the exo-*si* and endo-*re* Nucleophilic Addition Transition States. The Topological Quantities are Derived from the Atoms In Molecule (AIM) Analysis

| | | | | | | |
|---|---|---|---|---|---|---|
| | | | **[2a-3a]**$^‡$exo-*si* | | | |
| | | ρ | $\nabla^2\rho$ | G | $V=1/4\nabla^2\rho-2G$ | E=(1/2)V (kcal/mol) |
| N–H···O | a | 0.057 | -0.042 | 0.045 | -0.101 | -31.8 |
| C–H···O | b | 0.008 | -0.007 | 0.006 | -0.002 | -4.3 |
| | c | 0.019 | -0.014 | 0.014 | -0.032 | -10.1 |
| | q | 0.014 | -0.013 | 0.011 | -0.026 | -8.1 |
| | r | 0.008 | -0.008 | 0.006 | -0.015 | -4.7 |
| | y | 0.014 | -0.011 | 0.010 | -0.003 | -7.3 |
| | n | 0.008 | -0.007 | 0.006 | -0.013 | -4.2 |
| | o | 0.010 | -0.010 | 0.008 | -0.018 | -5.7 |
| | p | 0.021 | -0.015 | 0.015 | -0.034 | -10.7 |
| C–H···π | d | 0.007 | -0.005 | 0.004 | -0.010 | -3.2 |
| | e | 0.006 | -0.004 | 0.003 | -0.009 | -2.7 |
| | f | 0.006 | -0.005 | 0.004 | -0.008 | -2.6 |
| | g | 0.007 | -0.005 | 0.004 | -0.010 | -3.0 |
| | h | 0.004 | -0.003 | 0.002 | -0.006 | -1.8 |
| | j | 0.006 | -0.005 | 0.004 | -0.009 | -2.8 |
| | k | 0.006 | -0.005 | 0.004 | -0.009 | -2.7 |
| | l | 0.009 | -0.006 | 0.005 | -0.012 | -3.8 |
| | m | 0.010 | -0.008 | 0.006 | -0.015 | -4.6 |
| | s | 0.007 | -0.005 | 0.004 | -0.009 | -2.9 |
| | t | 0.006 | -0.005 | 0.004 | -0.009 | -2.7 |
| | u | 0.007 | -0.005 | 0.004 | -0.009 | -3.0 |
| | v | 0.006 | -0.005 | 0.004 | -0.008 | -2.6 |
| lp(O)···π | i | 0.007 | -0.006 | 0.005 | -0.011 | -3.5 |
| O–H···O | w | 0.045 | -0.034 | 0.035 | -0.078 | -24.4 |
| O–H···π | x | 0.011 | -0.008 | 0.007 | -0.016 | -4.9 |
| | | | **[2a-3a]**$^‡$endo-*re* | | | |
| N–H···O | a' | 0.055 | -0.042 | 0.044 | -0.099 | -31.0 |
| C–H···O | $b_1$' | 0.008 | -0.007 | 0.006 | -0.014 | -4.3 |
| | $b_2$' | 0.009 | -0.008 | -0.008 | -0.016 | -5.1 |
| | c' | 0.018 | -0.015 | 0.014 | -0.032 | -10.0 |
| | q' | 0.010 | -0.009 | 0.008 | -0.018 | -5.6 |

(2) (a) Espinosa, E.; Alkorta, I.; Elguero, J.; Molins, E. *J. Chem. Phys.* **2002**, *117*, 5529–5542. (b) Unnikrishnan, A. Sunoj, R. B. *Chem. Sci.* **2019**, *10*, 3826–3835.

| | | | | | | |
|---|---|---|---|---|---|---|
| | r' | 0.009 | -0.008 | 0.007 | -0.017 | -5.2 |
| | y' | 0.014 | -0.010 | 0.010 | -0.022 | -6.8 |
| | n' | 0.012 | -0.009 | 0.009 | -0.020 | -6.3 |
| | o' | 0.013 | -0.009 | 0.010 | -0.021 | -6.7 |
| | p' | 0.009 | -0.009 | 0.007 | -0.017 | -5.4 |
| C–H···π | d' | 0.005 | -0.004 | 0.003 | -0.006 | -2.0 |
| | e' | 0.007 | -0.005 | 0.004 | -0.009 | -2.9 |
| | f' | 0.003 | -0.002 | 0.002 | -0.004 | -1.3 |
| | g' | 0.005 | -0.004 | 0.003 | -0.006 | -2.0 |
| | h' | 0.004 | -0.003 | 0.002 | -0.005 | -1.5 |
| | k' | 0.004 | -0.003 | 0.003 | -0.006 | -2.0 |
| | l' | 0.006 | -0.004 | 0.003 | -0.008 | -2.4 |
| | m' | 0.010 | -0.009 | 0.007 | -0.016 | -5.2 |
| | s' | 0.007 | -0.006 | 0.004 | -0.010 | -3.3 |
| | $t_1$' | 0.007 | -0.005 | 0.004 | -0.009 | -2.9 |
| | $t_2$' | 0.007 | -0.005 | 0.004 | -0.009 | -2.9 |
| | $t_3$' | 0.006 | -0.004 | 0.003 | -0.008 | -2.5 |
| | u' | 0.005 | -0.004 | 0.003 | -0.008 | -2.5 |
| | v' | 0.007 | -0.005 | 0.004 | -0.009 | -3.0 |
| lp(O)···π | i' | 0.007 | -0.006 | 0.006 | -0.013 | -4.0 |
| O–H···O | w' | 0.042 | -0.032 | 0.032 | -0.071 | -22.4 |
| O–H···π | x' | 0.011 | -0.008 | 0.007 | -0.017 | -5.3 |
| π···π | $z_1$' | 0.005 | -0.003 | 0.003 | -0.006 | -2.0 |
| | $z_2$' | 0.006 | -0.005 | 0.004 | -0.010 | -3.0 |

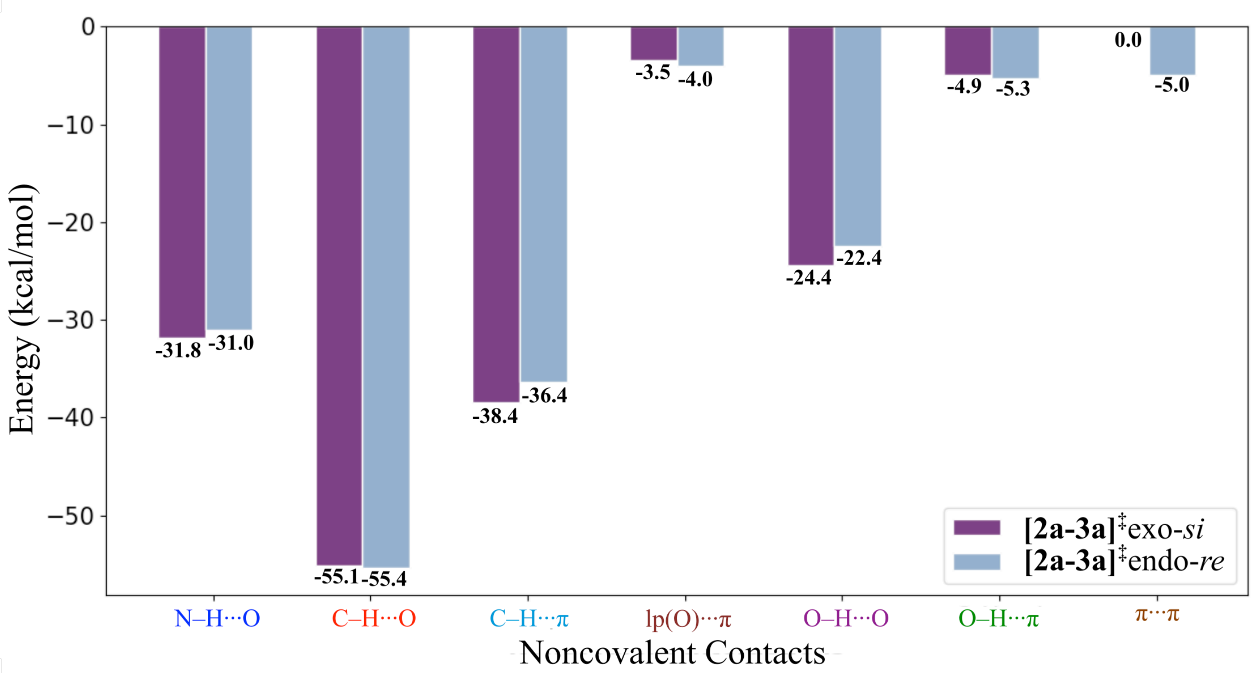


**Figure S10.** Plot of cumulative noncovalent interaction energies in the diastereomeric transition states **[2a-3a]**‡exo-*si* and **[2a-3a]**‡endo-*re* as obtained from Espinosa's formalism

## 15. Calculation of Free Energy Change in Each Step

The equations at each step used for calculation of free energy values as given in Figure 3 are shown below for exo-*si*:

(1) $Pd(PPh_3)_4$ + *trans*-allyl alcohol + **DAPCy** → **1a**exo-*si* (ΔG = 0 kcal/mol)

(2) **1a**exo-*si* → **[1a-2a]**$^{‡}$exo-*si* (ΔG = 9.7 kcal/mol)

(3) **[1a-2a]**$^{‡}$exo-*si* → **2a**exo-*si* (ΔG = -6.7 kcal/mol)

(4) **2a**exo-*si* → **[2a-3a]**$^{‡}$exo-*si* (ΔG = 3.9 kcal/mol)

(5) **[2a-3a]**$^{‡}$exo-*si* → **3a**exo-*si* (ΔG = 1.2 kcal/mol)

(6) **3a**exo-*si* → **3a'**exo-*si* (ΔG = 6.1 kcal/mol)

(7) **3a'**exo-*si* + *trans*-allyl alcohol → (*S*)-product + $H_2O$ + **1a**exo-*si* (ΔG = -15.1 kcal/mol)

## 16. Details of the Energetic Span Analysis

The energetic span (δ*E*) is determined from the turn-over determining transition state (TDTS) and the turn-over determining intermediate (TDI) using the following equation:

$\delta E = E_{(TDTS)} - E_{(TDI)}$, if TDTS appears after TDI ….(1)

$\delta E = E_{(TDTS)} - E_{(TDI)} + \Delta G_{rxn}$, if TDTS appears before TDI ….(2)

where $\Delta G_{rxn}$ is the Gibbs free energy of reaction.

**Table S8.** Calculation of Energetic Span (δE, in kcal/mol) using Different Likely Combinations of TDI and TDTS for Intramolecular Asymmetric Amination Catalyzed by $Pd(PPh_3)_4$ and (*S*,*R*,*S*)- or (*S*,*S*,*S*)-**DAPCy**

| (*S*,*R*,*S*)-**DAPCy** | | | | | |
|---|---|---|---|---|---|
| exo-*si* | | | endo-*re* | | |
| **TDI** | **[1a-2a]**$^{‡}$ | **[2a-3a]**$^{‡}$ | **TDI** | **[1a-2a]**$^{‡}$ | **[2a-3a]**$^{‡}$ |
| **1a** | 9.7 | 3.9 | **1a** | 7.7 | 7.0 |
| **2a** | 1.3 | 10.6 | **2a** | -0.3 | 14.1 |
| **3a** | -6.7 | -12.5 | **3a** | -12.5 | -13.2 |
| exo-*re* | | | endo-*si* | | |
| **TDI** | **[1a-2a]**$^{‡}$ | **[2a-3a]**$^{‡}$ | **TDI** | **[1a-2a]**$^{‡}$ | **[2a-3a]**$^{‡}$ |
| **1a** | 5.8 | -6.2 | **1a** | 7.5 | 6.1 |
| **2a** | -0.2 | 13.0 | **2a** | -6.0 | 11.2 |
| **3a** | -12.2 | -24.2 | **3a** | -14.6 | -16.0 |
| (*S*,*S*,*S*)-**DAPCy** | | | | | |

| exo-*si* | | | endo-*re* | | |
|---|---|---|---|---|---|
| **TDI** | **[1a-2a]‡** | **[2a-3a]‡** | **TDI** | **[1a-2a]‡** | **[2a-3a]‡** |
| **1a** | 6.3 | 1.5 | **1a** | 6.5 | 8.0 |
| **2a** | 1.9 | 12.3 | **2a** | -1.5 | 15.1 |
| **3a** | -9.8 | -14.6 | **3a** | -13.2 | -11.8 |
| exo-*re* | | | endo-*si* | | |
| **TDI** | **[1a-2a]‡** | **[2a-3a]‡** | **TDI** | **[1a-2a]‡** | **[2a-3a]‡** |
| **1a** | 10.1 | 6.3 | **1a** | 6.6 | 6.7 |
| **2a** | 1.4 | 12.6 | **2a** | -2.2 | 13.0 |
| **3a** | -9.2 | -13.0 | **3a** | -13.7 | -13.6 |

## 17. Cartesian Coordinates of the Optimized Geometries of Various Stationary Points

----------------------------------------------------------------------------------------------------------------

$Pd(PPh_3)_4$

----------------------------------------------------------------------------------------------------------------

Number of imaginary frequencies : 0
The smallest frequencies are : 13.9853 20.9721 24.8416 cm(-1)

Electronic energy : HF=-4273.5448117
Zero-point correction= 1.105049 (Hartree/Particle)
Thermal correction to Energy= 1.174820
Thermal correction to Enthalpy= 1.175764
Thermal correction to Gibbs Free Energy= 0.994333
Sum of electronic and zero-point Energies= -4272.439762
Sum of electronic and thermal Energies= -4272.369992
Sum of electronic and thermal Enthalpies= -4272.369048
Sum of electronic and thermal Free Energies= -4272.550479

............................................................

Cartesian Coordinates

............................................................

----------------------------------------------------------------------------------------------------------------

*Trans*-allyl alcohol

----------------------------------------------------------------------------------------------------------------

Number of imaginary frequencies : 0
The smallest frequencies are : 10.1475 22.3072 31.1657 cm(-1)

Electronic energy : HF=-1761.8698509
Zero-point correction= 0.490849 (Hartree/Particle)
Thermal correction to Energy= 0.519603
Thermal correction to Enthalpy= 0.520548
Thermal correction to Gibbs Free Energy= 0.429371
Sum of electronic and zero-point Energies= -1761.379002
Sum of electronic and thermal Energies= -1761.350247
Sum of electronic and thermal Enthalpies= -1761.349303
Sum of electronic and thermal Free Energies= -1761.440480

............................................................

Cartesian Coordinates

............................................................

| | | | |
|---|---|---|---|
| 6 | 2.656805 | -1.228416 | -1.563263 |
| 6 | 2.125005 | -2.429739 | -1.807469 |
| 1 | 3.675425 | -1.195364 | -1.175999 |
| 1 | 1.111463 | -2.528263 | -2.186725 |
| 6 | 2.807984 | -3.685403 | -1.330900 |
| 6 | 1.956688 | 0.099423 | -1.660120 |
| 6 | 1.623042 | 0.729246 | -0.248344 |

| | | | |
|---|---|---|---|
| 1 | 2.604922 | 0.806265 | -2.190128 |
| 1 | 1.031513 | -0.002947 | -2.233704 |
| 6 | 0.232497 | 0.246694 | 0.272346 |
| 1 | -0.522367 | 0.785936 | -0.297044 |
| 1 | 0.119553 | 0.571986 | 1.312219 |
| 6 | 1.437888 | 2.257471 | -0.351123 |
| 6 | 0.946503 | 2.853781 | -1.523082 |
| 6 | 1.631097 | 3.074524 | 0.772989 |
| 6 | 0.682474 | 4.223574 | -1.575942 |
| 1 | 0.756221 | 2.251168 | -2.404747 |
| 6 | 1.365781 | 4.444028 | 0.723156 |
| 1 | 2.000691 | 2.636814 | 1.694652 |
| 6 | 0.893391 | 5.026512 | -0.453756 |
| 1 | 0.309631 | 4.661347 | -2.497934 |
| 1 | 1.529320 | 5.054054 | 1.607321 |
| 1 | 0.689360 | 6.092679 | -0.495525 |
| 6 | 2.762375 | 0.379768 | 0.720557 |
| 6 | 4.032578 | 0.942231 | 0.508529 |
| 6 | 2.620880 | -0.544931 | 1.764514 |
| 6 | 5.123744 | 0.583842 | 1.297252 |
| 1 | 4.162632 | 1.669704 | -0.287535 |
| 6 | 3.715628 | -0.909782 | 2.557579 |
| 1 | 1.658050 | -0.994751 | 1.983309 |
| 6 | 4.971535 | -0.351457 | 2.325168 |
| 1 | 6.094394 | 1.034375 | 1.109287 |
| 1 | 3.577277 | -1.629351 | 3.359840 |
| 1 | 5.821042 | -0.635324 | 2.939497 |
| 7 | -0.087082 | -1.183797 | 0.179525 |
| 1 | 0.676776 | -1.855167 | 0.163017 |
| 16 | -1.284822 | -1.693540 | -0.876752 |
| 8 | -1.294901 | -0.879112 | -2.105320 |
| 8 | -1.101476 | -3.146586 | -0.986022 |
| 8 | 2.539548 | -3.884982 | 0.069555 |
| 1 | 2.919192 | -3.121524 | 0.530191 |
| 1 | 2.420322 | -4.574471 | -1.835463 |
| 1 | 3.892356 | -3.636711 | -1.516046 |
| 6 | -2.800303 | -1.368501 | 0.054086 |
| 6 | -3.468866 | -0.099039 | 0.068563 |
| 6 | -3.238687 | -2.429410 | 0.820997 |
| 6 | -3.119576 | 1.032150 | -0.724218 |
| 6 | -4.592217 | 0.028712 | 0.961189 |
| 6 | -4.361132 | -2.289639 | 1.664257 |
| 1 | -2.711419 | -3.375441 | 0.773205 |
| 6 | -3.804903 | 2.221103 | -0.605799 |
| 1 | -2.321443 | 0.948032 | -1.448094 |
| 6 | -5.276601 | 1.271596 | 1.050356 |
| 6 | -5.012504 | -1.081612 | 1.740183 |
| 1 | -4.693876 | -3.137512 | 2.254503 |
| 6 | -4.888940 | 2.351242 | 0.292487 |
| 1 | -3.509911 | 3.067206 | -1.219558 |
| 1 | -6.117991 | 1.349438 | 1.733888 |
| 1 | -5.867774 | -0.958076 | 2.399290 |
| 1 | -5.416996 | 3.296746 | 0.372777 |

---

(*S*,*R*,*S*)-**DAPCy**

---

Number of imaginary frequencies : 0
The smallest frequencies are : 10.3715 12.7152 18.5416 cm(-1)

Electronic energy : HF=-2651.2726756
Zero-point correction= 0.841791 (Hartree/Particle)

Thermal correction to Energy= 0.887453
Thermal correction to Enthalpy= 0.888397
Thermal correction to Gibbs Free Energy= 0.760577
Sum of electronic and zero-point Energies= -2650.430885
Sum of electronic and thermal Energies= -2650.385222
Sum of electronic and thermal Enthalpies= -2650.384278
Sum of electronic and thermal Free Energies= -2650.512098

.............................................................

Cartesian Coordinates

.............................................................

| | | | |
|---|---|---|---|
| 1 | 1.219286 | 1.628887 | -2.578270 |
| 6 | 1.548945 | 0.132856 | 0.569880 |
| 6 | 2.849472 | 0.592931 | 0.578797 |
| 6 | 3.354805 | 1.196377 | 1.773453 |
| 6 | 4.684040 | 1.690695 | 1.868152 |
| 1 | 5.337551 | 1.606308 | 1.006749 |
| 6 | 5.137703 | 2.269990 | 3.032803 |
| 1 | 6.156137 | 2.643342 | 3.088919 |
| 6 | 4.287009 | 2.385374 | 4.159486 |
| 1 | 4.658683 | 2.846327 | 5.069966 |
| 6 | 2.995308 | 1.913995 | 4.100439 |
| 1 | 2.336713 | 1.996351 | 4.961201 |
| 6 | 2.495574 | 1.306620 | 2.915766 |
| 6 | 1.172748 | 0.804382 | 2.842908 |
| 1 | 0.527263 | 0.901978 | 3.710883 |
| 6 | 0.679780 | 0.208742 | 1.696022 |
| 6 | -0.685055 | -0.371691 | 1.661277 |
| 6 | -1.163109 | -1.111742 | 2.727440 |
| 1 | -0.511661 | -1.305072 | 3.574736 |
| 6 | -2.476436 | -1.642176 | 2.741819 |
| 6 | -2.960515 | -2.396646 | 3.845563 |
| 1 | -2.297914 | -2.568927 | 4.689844 |
| 6 | -4.242747 | -2.896553 | 3.848252 |
| 1 | -4.603221 | -3.470169 | 4.697197 |
| 6 | -5.097984 | -2.663522 | 2.743525 |
| 1 | -6.109091 | -3.060109 | 2.754693 |
| 6 | -4.658479 | -1.940994 | 1.656034 |
| 1 | -5.315937 | -1.766899 | 0.811493 |
| 6 | -3.338992 | -1.413556 | 1.620404 |
| 6 | -2.849063 | -0.661747 | 0.505000 |
| 6 | -1.559492 | -0.175960 | 0.551788 |
| 6 | 3.709348 | 0.418992 | -0.634495 |
| 6 | 3.878330 | 1.523005 | -1.535761 |
| 6 | 3.272213 | 2.793087 | -1.313593 |
| 1 | 2.660327 | 2.937938 | -0.430854 |
| 6 | 3.438795 | 3.826726 | -2.210377 |
| 1 | 2.961120 | 4.783968 | -2.022306 |
| 6 | 4.226257 | 3.651934 | -3.374098 |
| 1 | 4.348609 | 4.474926 | -4.072244 |
| 6 | 4.832740 | 2.439758 | -3.615820 |
| 1 | 5.439685 | 2.293490 | -4.505784 |
| 6 | 4.677409 | 1.352509 | -2.714077 |
| 6 | 5.287249 | 0.092569 | -2.948535 |
| 1 | 5.892695 | -0.040942 | -3.841446 |
| 6 | 5.114092 | -0.942497 | -2.064455 |
| 1 | 5.586980 | -1.899167 | -2.264516 |
| 6 | 4.322008 | -0.803041 | -0.890212 |
| 6 | -3.717728 | -0.381893 | -0.680571 |
| 6 | -3.851598 | -1.392175 | -1.689441 |
| 6 | -3.167298 | -2.637240 | -1.619325 |
| 1 | -2.509581 | -2.831726 | -0.779409 |
| 6 | -3.319388 | -3.586386 | -2.605525 |

| | | | |
|---|---|---|---|
| 1 | -2.781920 | -4.527758 | -2.535255 |
| 6 | -4.163952 | -3.343051 | -3.714995 |
| 1 | -4.274012 | -4.100333 | -4.486098 |
| 6 | -4.836050 | -2.146082 | -3.817309 |
| 1 | -5.481334 | -1.945390 | -4.669088 |
| 6 | -4.697811 | -1.145259 | -2.818565 |
| 6 | -5.369908 | 0.101583 | -2.907990 |
| 1 | -6.009672 | 0.293561 | -3.765885 |
| 6 | -5.211036 | 1.054691 | -1.933047 |
| 1 | -5.727297 | 2.005456 | -2.026714 |
| 6 | -4.382838 | 0.832984 | -0.797350 |
| 8 | 0.526269 | 1.634632 | -1.899316 |
| 8 | -0.375645 | -0.730966 | -2.645376 |
| 8 | 1.099239 | -0.531142 | -0.573712 |
| 8 | -1.139709 | 0.655830 | -0.489922 |
| 15 | -0.008509 | 0.167280 | -1.537216 |
| 6 | -4.236634 | 1.934977 | 0.236214 |
| 6 | -3.517996 | 3.173912 | -0.346745 |
| 6 | -5.582581 | 2.341210 | 0.875922 |
| 1 | -3.606206 | 1.558520 | 1.048939 |
| 6 | -3.322604 | 4.256531 | 0.724078 |
| 1 | -4.113723 | 3.583069 | -1.174486 |
| 1 | -2.554299 | 2.868905 | -0.767391 |
| 6 | -5.382248 | 3.424540 | 1.946224 |
| 1 | -6.261711 | 2.721522 | 0.100952 |
| 1 | -6.064657 | 1.457311 | 1.310774 |
| 6 | -4.656323 | 4.651993 | 1.375087 |
| 1 | -2.835401 | 5.135913 | 0.285352 |
| 1 | -2.641350 | 3.874391 | 1.497935 |
| 1 | -6.350183 | 3.716359 | 2.371874 |
| 1 | -4.788952 | 3.006733 | 2.772116 |
| 1 | -4.491585 | 5.397590 | 2.162676 |
| 1 | -5.297368 | 5.128635 | 0.619332 |
| 6 | 4.165256 | -1.995647 | 0.036046 |
| 6 | 3.425904 | -3.166577 | -0.650663 |
| 6 | 5.516085 | -2.469565 | 0.617940 |
| 1 | 3.549631 | -1.688117 | 0.887841 |
| 6 | 3.238647 | -4.346509 | 0.313844 |
| 1 | 4.003239 | -3.500298 | -1.523822 |
| 1 | 2.458447 | -2.815197 | -1.024127 |
| 6 | 5.320945 | -3.650077 | 1.580903 |
| 1 | 6.183631 | -2.776653 | -0.198637 |
| 1 | 6.008646 | -1.634231 | 1.130062 |
| 6 | 4.579320 | -4.810906 | 0.902023 |
| 1 | 2.738526 | -5.175585 | -0.201153 |
| 1 | 2.572038 | -4.038095 | 1.131979 |
| 1 | 6.292241 | -3.987340 | 1.962929 |
| 1 | 4.741400 | -3.310944 | 2.451423 |
| 1 | 4.421204 | -5.630044 | 1.614146 |
| 1 | 5.206734 | -5.213359 | 0.093495 |

---

(*S*,*S*,*S*)-**DAPCy**

---

Number of imaginary frequencies : 0
The smallest frequencies are : 9.5453 12.2184 19.6812 cm(-1)

Electronic energy : HF=-2651.2690299
Zero-point correction= 0.842417 (Hartree/Particle)
Thermal correction to Energy= 0.887886
Thermal correction to Enthalpy= 0.888830
Thermal correction to Gibbs Free Energy= 0.762077

Sum of electronic and zero-point Energies= -2650.426613
Sum of electronic and thermal Energies= -2650.381144
Sum of electronic and thermal Enthalpies= -2650.380200
Sum of electronic and thermal Free Energies= -2650.506953

..........................................................

Cartesian Coordinates

..........................................................

| | | | |
|---|---|---|---|
| 1 | 1.430253 | -0.062507 | -2.760477 |
| 6 | 1.471355 | 0.437234 | 0.889786 |
| 6 | 2.852156 | 0.516071 | 0.875838 |
| 6 | 3.569033 | 0.015954 | 2.010570 |
| 6 | 4.972774 | 0.182816 | 2.153544 |
| 1 | 5.511555 | 0.737446 | 1.394043 |
| 6 | 5.638758 | -0.332450 | 3.243899 |
| 1 | 6.711360 | -0.189588 | 3.337829 |
| 6 | 4.935479 | -1.042227 | 4.247594 |
| 1 | 5.474455 | -1.451112 | 5.097189 |
| 6 | 3.571464 | -1.197602 | 4.153132 |
| 1 | 3.020020 | -1.723683 | 4.928054 |
| 6 | 2.851916 | -0.662515 | 3.049140 |
| 6 | 1.443976 | -0.778101 | 2.959358 |
| 1 | 0.914353 | -1.323070 | 3.734929 |
| 6 | 0.729351 | -0.212981 | 1.917585 |
| 6 | -0.753610 | -0.276966 | 1.903448 |
| 6 | -1.465474 | -0.101260 | 3.076983 |
| 1 | -0.932824 | 0.161614 | 3.985980 |
| 6 | -2.870419 | -0.260203 | 3.133899 |
| 6 | -3.583981 | -0.116707 | 4.355714 |
| 1 | -3.031454 | 0.147962 | 5.253544 |
| 6 | -4.943951 | -0.320439 | 4.404520 |
| 1 | -5.479392 | -0.212371 | 5.343237 |
| 6 | -5.648025 | -0.685595 | 3.231241 |
| 1 | -6.717919 | -0.866067 | 3.280903 |
| 6 | -4.987517 | -0.817547 | 2.029249 |
| 1 | -5.529413 | -1.106124 | 1.136130 |
| 6 | -3.587267 | -0.591764 | 1.938911 |
| 6 | -2.874079 | -0.697882 | 0.700668 |
| 6 | -1.499531 | -0.561356 | 0.720027 |
| 6 | 3.557609 | 1.102620 | -0.308773 |
| 6 | 3.473145 | 2.519099 | -0.537138 |
| 6 | 2.812704 | 3.400637 | 0.363839 |
| 1 | 2.356625 | 2.995656 | 1.260204 |
| 6 | 2.747228 | 4.753803 | 0.117389 |
| 1 | 2.236680 | 5.405186 | 0.820996 |
| 6 | 3.337346 | 5.304617 | -1.045433 |
| 1 | 3.274227 | 6.373214 | -1.229724 |
| 6 | 3.990936 | 4.483439 | -1.935278 |
| 1 | 4.453491 | 4.893460 | -2.829648 |
| 6 | 4.080308 | 3.084005 | -1.705120 |
| 6 | 4.763027 | 2.223013 | -2.602097 |
| 1 | 5.221139 | 2.646028 | -3.492750 |
| 6 | 4.856773 | 0.878227 | -2.344761 |
| 1 | 5.399437 | 0.242543 | -3.037885 |
| 6 | 4.263751 | 0.283984 | -1.193732 |
| 6 | -3.596068 | -0.979890 | -0.580833 |
| 6 | -3.549302 | -2.311323 | -1.116778 |
| 6 | -2.843858 | -3.370024 | -0.479551 |
| 1 | -2.320437 | -3.172341 | 0.449408 |
| 6 | -2.816608 | -4.636045 | -1.021856 |
| 1 | -2.269202 | -5.427107 | -0.516939 |
| 6 | -3.494837 | -4.916009 | -2.232254 |
| 1 | -3.463662 | -5.918096 | -2.650531 |

| | | | |
|---|---|---|---|
| 6 | -4.193221 | -3.917215 | -2.872155 |
| 1 | -4.721940 | -4.119812 | -3.800399 |
| 6 | -4.241912 | -2.602186 | -2.336531 |
| 6 | -4.966873 | -1.561991 | -2.973554 |
| 1 | -5.496850 | -1.779599 | -3.897606 |
| 6 | -5.001760 | -0.301488 | -2.433461 |
| 1 | -5.564431 | 0.477636 | -2.938132 |
| 6 | -4.314862 | 0.022345 | -1.228907 |
| 8 | 0.989234 | -0.566803 | -2.058634 |
| 8 | -0.836969 | 1.308970 | -2.070642 |
| 8 | 0.765734 | 1.121330 | -0.100945 |
| 8 | -0.802434 | -0.829827 | -0.464513 |
| 15 | -0.055323 | 0.354967 | -1.263669 |
| 6 | -4.406050 | 1.446233 | -0.702136 |
| 6 | -5.838135 | 1.804259 | -0.238778 |
| 6 | -3.911819 | 2.495865 | -1.724367 |
| 1 | -3.754202 | 1.528506 | 0.174473 |
| 6 | -5.893835 | 3.218900 | 0.356181 |
| 1 | -6.520393 | 1.742001 | -1.098178 |
| 1 | -6.189635 | 1.074859 | 0.497400 |
| 6 | -3.958610 | 3.909213 | -1.125628 |
| 1 | -4.551355 | 2.466802 | -2.617524 |
| 1 | -2.897728 | 2.247057 | -2.043860 |
| 6 | -5.367458 | 4.268943 | -0.632499 |
| 1 | -6.920915 | 3.458187 | 0.658954 |
| 1 | -5.283680 | 3.243195 | 1.270518 |
| 1 | -3.614988 | 4.640446 | -1.867523 |
| 1 | -3.253572 | 3.964725 | -0.283716 |
| 1 | -5.371169 | 5.263768 | -0.169791 |
| 1 | -6.047144 | 4.320982 | -1.495697 |
| 6 | 4.466487 | -1.211231 | -0.979062 |
| 6 | 5.956312 | -1.543215 | -0.716207 |
| 6 | 3.948855 | -2.082625 | -2.147119 |
| 1 | 3.904566 | -1.513482 | -0.090299 |
| 6 | 6.155008 | -3.035207 | -0.415802 |
| 1 | 6.547180 | -1.268570 | -1.600892 |
| 1 | 6.335747 | -0.940565 | 0.113612 |
| 6 | 4.146065 | -3.578372 | -1.855495 |
| 1 | 4.485809 | -1.821878 | -3.069460 |
| 1 | 2.890007 | -1.879554 | -2.319315 |
| 6 | 5.613070 | -3.911971 | -1.552918 |
| 1 | 7.218248 | -3.243754 | -0.244332 |
| 1 | 5.631428 | -3.282542 | 0.518602 |
| 1 | 3.785234 | -4.172999 | -2.703502 |
| 1 | 3.525623 | -3.857111 | -0.991984 |
| 1 | 5.719602 | -4.974400 | -1.301903 |
| 1 | 6.215652 | -3.740129 | -2.456753 |

---

$PPh_3$

---

Number of imaginary frequencies : 0
The smallest frequencies are : 23.9400 30.7428 41.9399 cm(-1)

Electronic energy : HF=-1036.3544004
Zero-point correction= 0.274253 (Hartree/Particle)
Thermal correction to Energy= 0.290120
Thermal correction to Enthalpy= 0.291065
Thermal correction to Gibbs Free Energy= 0.228163
Sum of electronic and zero-point Energies= -1036.080147
Sum of electronic and thermal Energies= -1036.064280
Sum of electronic and thermal Enthalpies= -1036.063336

Sum of electronic and thermal Free Energies= -1036.126237

.............................................................

Cartesian Coordinates

.............................................................

| | | | |
|---|---|---|---|
| 15 | -0.001698 | -0.001275 | -1.245636 |
| 6 | 0.648103 | 1.529890 | -0.433203 |
| 6 | 1.773650 | 2.134511 | -1.018443 |
| 6 | 2.337807 | 3.282163 | -0.460879 |
| 6 | 1.773678 | 3.854268 | 0.682201 |
| 6 | 0.647344 | 3.269572 | 1.264987 |
| 6 | 0.089373 | 2.113481 | 0.714489 |
| 6 | -1.651015 | -0.202640 | -0.428984 |
| 6 | -2.743999 | 0.444886 | -1.029742 |
| 6 | -4.019875 | 0.356818 | -0.472259 |
| 6 | -4.226145 | -0.394921 | 0.687198 |
| 6 | -3.149626 | -1.052063 | 1.286745 |
| 6 | -1.870014 | -0.955583 | 0.735258 |
| 1 | 2.211400 | 1.702654 | -1.915472 |
| 1 | -0.782434 | 1.665104 | 1.180376 |
| 1 | 3.211008 | 3.734174 | -0.923274 |
| 1 | 0.202377 | 3.711202 | 2.152671 |
| 1 | 2.206529 | 4.752854 | 1.112998 |
| 1 | -2.594399 | 1.021671 | -1.939529 |
| 1 | -1.040845 | -1.467136 | 1.213819 |
| 1 | -4.853310 | 0.866883 | -0.947483 |
| 1 | -3.303917 | -1.640131 | 2.187508 |
| 1 | -5.220465 | -0.471258 | 1.118340 |
| 6 | 0.999987 | -1.327017 | -0.429724 |
| 6 | 1.774591 | -1.134751 | 0.724920 |
| 6 | 0.974011 | -2.601651 | -1.020919 |
| 6 | 2.499439 | -2.193605 | 1.276493 |
| 1 | 1.812354 | -0.157390 | 1.195666 |
| 6 | 1.688871 | -3.661660 | -0.462673 |
| 1 | 0.389974 | -2.764619 | -1.923735 |
| 6 | 2.456493 | -3.458859 | 0.687008 |
| 1 | 3.096043 | -2.028951 | 2.169790 |
| 1 | 1.654310 | -4.641885 | -0.930188 |
| 1 | 3.020810 | -4.281083 | 1.118213 |

---

**[**(S,R,S)-(S,S,S)**DAPCy]**‡

---

Number of imaginary frequencies : 1
The smallest frequencies are : -66.6172 14.0983 18.4367 cm(-1)

Electronic energy : HF=-2651.2526146
Zero-point correction= 0.841593 (Hartree/Particle)
Thermal correction to Energy= 0.886589
Thermal correction to Enthalpy= 0.887533
Thermal correction to Gibbs Free Energy= 0.763124
Sum of electronic and zero-point Energies= -2650.411022
Sum of electronic and thermal Energies= -2650.366026
Sum of electronic and thermal Enthalpies= -2650.365081
Sum of electronic and thermal Free Energies= -2650.489490

.............................................................

Cartesian Coordinates

.............................................................

| | | | |
|---|---|---|---|
| 6 | -4.635779 | -3.089535 | 3.546800 |
| 6 | -3.287723 | -2.823283 | 3.497292 |
| 6 | -2.733390 | -2.093506 | 2.407177 |
| 6 | -3.591779 | -1.643782 | 1.361666 |
| 6 | -4.982512 | -1.931460 | 1.439454 |

| | | | |
|---|---|---|---|
| 6 | -5.489001 | -2.636920 | 2.507979 |
| 1 | -0.780873 | -2.162945 | 3.186135 |
| 1 | -5.052767 | -3.645500 | 4.381375 |
| 1 | -2.626814 | -3.163798 | 4.290358 |
| 6 | -1.355720 | -1.799258 | 2.347466 |
| 6 | -3.004675 | -0.909191 | 0.295019 |
| 1 | -5.637705 | -1.588576 | 0.646879 |
| 1 | -6.552669 | -2.851108 | 2.557379 |
| 6 | -1.641191 | -0.657632 | 0.286145 |
| 6 | -0.732597 | -1.088382 | 1.322005 |
| 6 | -3.865399 | -0.407046 | -0.824359 |
| 6 | -4.224234 | -1.317042 | -1.872347 |
| 6 | -4.338923 | 0.900807 | -0.824568 |
| 6 | -3.742228 | -2.655458 | -1.921426 |
| 6 | -5.099274 | -0.873902 | -2.917255 |
| 6 | -5.218393 | 1.308916 | -1.865660 |
| 6 | -4.107711 | -3.505260 | -2.942052 |
| 1 | -3.074333 | -3.004218 | -1.141195 |
| 6 | -5.457472 | -1.775276 | -3.955740 |
| 6 | -5.588869 | 0.456824 | -2.876858 |
| 1 | -5.608136 | 2.321835 | -1.855994 |
| 6 | -4.974845 | -3.064329 | -3.970908 |
| 1 | -3.726402 | -4.522430 | -2.960010 |
| 1 | -6.122253 | -1.425043 | -4.741622 |
| 1 | -6.262301 | 0.794004 | -3.661022 |
| 1 | -5.255433 | -3.744984 | -4.769732 |
| 6 | 0.765791 | -0.843556 | 1.433722 |
| 6 | 1.440485 | -1.353169 | 2.542226 |
| 6 | 1.617555 | -0.117479 | 0.526068 |
| 6 | 2.815448 | -1.172213 | 2.801715 |
| 1 | 0.912531 | -1.930773 | 3.286178 |
| 6 | 2.974368 | 0.088904 | 0.721481 |
| 6 | 3.425007 | -1.719025 | 3.966725 |
| 6 | 3.612253 | -0.423828 | 1.885765 |
| 6 | 4.763232 | -1.522430 | 4.215136 |
| 1 | 2.813287 | -2.292965 | 4.658171 |
| 6 | 4.989948 | -0.225111 | 2.174495 |
| 6 | 5.549783 | -0.763533 | 3.311871 |
| 1 | 5.221118 | -1.942530 | 5.105848 |
| 1 | 5.592509 | 0.359879 | 1.489300 |
| 1 | 6.603096 | -0.602958 | 3.522976 |
| 6 | 3.763076 | 0.836533 | -0.310667 |
| 6 | 3.656748 | 2.267123 | -0.372888 |
| 6 | 4.588439 | 0.150209 | -1.195546 |
| 6 | 2.797320 | 3.008911 | 0.484629 |
| 6 | 4.424776 | 2.986689 | -1.345717 |
| 6 | 5.348960 | 0.891878 | -2.143828 |
| 6 | 2.694407 | 4.379188 | 0.376542 |
| 1 | 2.197500 | 2.486298 | 1.220215 |
| 6 | 4.307055 | 4.400499 | -1.422533 |
| 6 | 5.277065 | 2.259753 | -2.217938 |
| 1 | 5.995172 | 0.354219 | -2.831378 |
| 6 | 3.457861 | 5.086111 | -0.582649 |
| 1 | 2.017973 | 4.918641 | 1.033149 |
| 1 | 4.897891 | 4.931457 | -2.165018 |
| 1 | 5.864678 | 2.803114 | -2.953828 |
| 1 | 3.370582 | 6.166623 | -0.654217 |
| 8 | 1.165988 | 0.416010 | -0.675455 |
| 8 | -1.250676 | 0.053820 | -0.846344 |
| 15 | -0.203936 | 1.262996 | -0.721209 |
| 8 | -0.225747 | 1.792283 | -2.225637 |
| 8 | -0.411113 | 2.238192 | 0.369109 |

| | | | |
|---|---|---|---|
| 1 | 0.050515 | 2.721480 | -2.265421 |
| 6 | -3.971313 | 1.879612 | 0.277581 |
| 6 | -3.570613 | 3.277475 | -0.239048 |
| 6 | -5.101531 | 2.001316 | 1.325954 |
| 1 | -3.095611 | 1.486636 | 0.802760 |
| 6 | -3.135040 | 4.180669 | 0.923469 |
| 1 | -4.418267 | 3.745821 | -0.756940 |
| 1 | -2.762292 | 3.183567 | -0.972012 |
| 6 | -4.684251 | 2.917373 | 2.485672 |
| 1 | -6.001519 | 2.402801 | 0.838229 |
| 1 | -5.362535 | 1.005046 | 1.701667 |
| 6 | -4.241988 | 4.299271 | 1.981612 |
| 1 | -2.864635 | 5.174335 | 0.544549 |
| 1 | -2.229376 | 3.755343 | 1.372544 |
| 1 | -5.507712 | 3.016023 | 3.203897 |
| 1 | -3.850052 | 2.446544 | 3.024691 |
| 1 | -3.902434 | 4.918666 | 2.820959 |
| 1 | -5.108608 | 4.814166 | 1.541476 |
| 6 | 4.676296 | -1.366329 | -1.214140 |
| 6 | 4.144563 | -1.954641 | -2.541386 |
| 6 | 6.102257 | -1.887505 | -0.927411 |
| 1 | 4.031502 | -1.756388 | -0.420432 |
| 6 | 4.187090 | -3.489798 | -2.526601 |
| 1 | 4.752176 | -1.581685 | -3.377028 |
| 1 | 3.122049 | -1.599772 | -2.714376 |
| 6 | 6.139771 | -3.422340 | -0.912188 |
| 1 | 6.792490 | -1.515380 | -1.696983 |
| 1 | 6.453054 | -1.490943 | 0.031712 |
| 6 | 5.599751 | -4.011390 | -2.223140 |
| 1 | 3.831125 | -3.884229 | -3.486208 |
| 1 | 3.493186 | -3.859567 | -1.758091 |
| 1 | 7.163456 | -3.770921 | -0.729035 |
| 1 | 5.529785 | -3.785820 | -0.073128 |
| 1 | 5.599755 | -5.107338 | -2.177658 |
| 1 | 6.271610 | -3.730691 | -3.047216 |

---

**[(*R*)-(*S*)A]**$^{‡}$

---

Number of imaginary frequencies : 1
The smallest frequencies are : -118.9565 24.5941 44.3090 cm(-1)

Electronic energy : HF=-1412.3628655
Zero-point correction= 0.282256 (Hartree/Particle)
Thermal correction to Energy= 0.300671
Thermal correction to Enthalpy= 0.301615
Thermal correction to Gibbs Free Energy= 0.235312
Sum of electronic and zero-point Energies= -1412.080609
Sum of electronic and thermal Energies= -1412.062195
Sum of electronic and thermal Enthalpies= -1412.061251
Sum of electronic and thermal Free Energies= -1412.127553

............................................................

Cartesian Coordinates

............................................................

| | | | |
|---|---|---|---|
| 6 | -4.800395 | -2.984091 | 0.174258 |
| 6 | -3.430502 | -2.859024 | 0.162333 |
| 6 | -2.824633 | -1.580275 | 0.003940 |
| 6 | -3.660176 | -0.433498 | -0.142319 |
| 6 | -5.071997 | -0.589634 | -0.126456 |
| 6 | -5.627798 | -1.839390 | 0.029031 |
| 1 | -0.857678 | -2.327023 | 0.106145 |
| 1 | -5.257272 | -3.961857 | 0.295552 |

| | | | |
|---|---|---|---|
| 1 | -2.794682 | -3.733521 | 0.274597 |
| 6 | -1.423236 | -1.413959 | -0.011036 |
| 6 | -3.018022 | 0.812290 | -0.287228 |
| 1 | -5.701150 | 0.289279 | -0.237309 |
| 1 | -6.707586 | -1.956035 | 0.041292 |
| 6 | -1.646485 | 0.932036 | -0.299327 |
| 6 | -0.758000 | -0.195097 | -0.163326 |
| 6 | 0.757893 | -0.195215 | -0.163459 |
| 6 | 1.423069 | -1.414113 | -0.011186 |
| 6 | 1.646408 | 0.931836 | -0.299684 |
| 6 | 2.824470 | -1.580443 | 0.003845 |
| 1 | 0.857493 | -2.327182 | 0.105881 |
| 6 | 3.017935 | 0.812103 | -0.287538 |
| 6 | 3.430308 | -2.859191 | 0.162356 |
| 6 | 3.660047 | -0.433688 | -0.142426 |
| 6 | 4.800199 | -2.984278 | 0.174416 |
| 1 | 2.794465 | -3.733670 | 0.274628 |
| 6 | 5.071864 | -0.589849 | -0.126441 |
| 6 | 5.627634 | -1.839603 | 0.029186 |
| 1 | 5.257048 | -3.962044 | 0.295826 |
| 1 | 5.701041 | 0.289045 | -0.237304 |
| 1 | 6.707418 | -1.956264 | 0.041558 |
| 8 | 1.236118 | 2.246398 | -0.511170 |
| 8 | -1.236347 | 2.246730 | -0.510353 |
| 15 | 0.000240 | 2.899907 | 0.284622 |
| 8 | 0.000279 | 4.368869 | -0.344797 |
| 8 | 0.000689 | 2.791066 | 1.757314 |
| 1 | -0.000029 | 5.034452 | 0.360048 |
| 1 | -3.602211 | 1.720216 | -0.395670 |
| 1 | 3.602141 | 1.719998 | -0.396147 |

---

(*R*)-**A**

---

Number of imaginary frequencies : 0
The smallest frequencies are : 46.0003 48.3112 53.1244 cm(-1)

Electronic energy : HF=-1412.3823196
Zero-point correction= 0.283738 (Hartree/Particle)
Thermal correction to Energy= 0.302289
Thermal correction to Enthalpy= 0.303233
Thermal correction to Gibbs Free Energy= 0.237276
Sum of electronic and zero-point Energies= -1412.098582
Sum of electronic and thermal Energies= -1412.080031
Sum of electronic and thermal Enthalpies= -1412.079087
Sum of electronic and thermal Free Energies= -1412.145044

..........................................................

Cartesian Coordinates

..........................................................

| | | | |
|---|---|---|---|
| 6 | -5.004244 | -2.693583 | -0.513328 |
| 6 | -3.632396 | -2.687784 | -0.630251 |
| 6 | -2.874865 | -1.555994 | -0.221827 |
| 6 | -3.563067 | -0.416695 | 0.309967 |
| 6 | -4.978813 | -0.453588 | 0.418394 |
| 6 | -5.682659 | -1.567142 | 0.016678 |
| 1 | -0.946211 | -2.372516 | -0.755814 |
| 1 | -5.573791 | -3.563459 | -0.827327 |
| 1 | -3.108890 | -3.548933 | -1.037286 |
| 6 | -1.461913 | -1.515890 | -0.331354 |
| 6 | -2.803042 | 0.713688 | 0.705780 |
| 1 | -5.496222 | 0.412104 | 0.823081 |
| 1 | -6.765237 | -1.585657 | 0.102857 |

| | | | |
|---|---|---|---|
| 6 | -1.437947 | 0.706697 | 0.581998 |
| 6 | -0.722734 | -0.412646 | 0.060810 |
| 6 | 0.755306 | -0.403230 | -0.041484 |
| 6 | 1.509731 | -1.501904 | 0.333665 |
| 6 | 1.454929 | 0.731958 | -0.547800 |
| 6 | 2.922909 | -1.520684 | 0.220079 |
| 1 | 1.006706 | -2.371179 | 0.747483 |
| 6 | 2.819061 | 0.761441 | -0.675506 |
| 6 | 3.696740 | -2.647787 | 0.610556 |
| 6 | 3.594566 | -0.364622 | -0.297106 |
| 6 | 5.068233 | -2.633136 | 0.490473 |
| 1 | 3.186065 | -3.521701 | 1.006513 |
| 6 | 5.010392 | -0.380388 | -0.408578 |
| 6 | 5.730096 | -1.489988 | -0.024453 |
| 1 | 5.650348 | -3.499542 | 0.790756 |
| 1 | 5.514979 | 0.498502 | -0.800639 |
| 1 | 6.812680 | -1.492228 | -0.112453 |
| 8 | 0.719531 | 1.829710 | -1.013607 |
| 8 | -0.726444 | 1.808818 | 1.068466 |
| 15 | 0.028779 | 2.824798 | 0.058211 |
| 8 | -1.105486 | 3.446222 | -0.894930 |
| 8 | 0.874195 | 3.793933 | 0.780138 |
| 1 | -1.210680 | 4.393140 | -0.715477 |
| 1 | -3.293341 | 1.588035 | 1.121009 |
| 1 | 3.295857 | 1.649662 | -1.076674 |

---

(*S*)-**A**

---

Number of imaginary frequencies : 0
The smallest frequencies are : 46.0015 48.3137 53.1281 cm(-1)

Electronic energy : HF=-1412.3823196
Zero-point correction= 0.283738 (Hartree/Particle)
Thermal correction to Energy= 0.302289
Thermal correction to Enthalpy= 0.303233
Thermal correction to Gibbs Free Energy= 0.237277
Sum of electronic and zero-point Energies= -1412.098582
Sum of electronic and thermal Energies= -1412.080031
Sum of electronic and thermal Enthalpies= -1412.079087
Sum of electronic and thermal Free Energies= -1412.145043

............................................................

Cartesian Coordinates

............................................................

| | | | |
|---|---|---|---|
| 6 | -5.068246 | -2.633122 | 0.490468 |
| 6 | -3.696752 | -2.647778 | 0.610548 |
| 6 | -2.922919 | -1.520676 | 0.220076 |
| 6 | -3.594573 | -0.364607 | -0.297098 |
| 6 | -5.010399 | -0.380367 | -0.408564 |
| 6 | -5.730106 | -1.489967 | -0.024446 |
| 1 | -1.006716 | -2.371180 | 0.747471 |
| 1 | -5.650363 | -3.499528 | 0.790746 |
| 1 | -3.186080 | -3.521697 | 1.006497 |
| 6 | -1.509740 | -1.501902 | 0.333658 |
| 6 | -2.819065 | 0.761454 | -0.675497 |
| 1 | -5.514984 | 0.498528 | -0.800618 |
| 1 | -6.812691 | -1.492202 | -0.112443 |
| 6 | -1.454932 | 0.731963 | -0.547798 |
| 6 | -0.755312 | -0.403228 | -0.041488 |
| 6 | 0.722728 | -0.412649 | 0.060808 |
| 6 | 1.461906 | -1.515893 | -0.331356 |
| 6 | 1.437938 | 0.706688 | 0.582010 |

| | | | |
|---|---|---|---|
| 6 | 2.874858 | -1.555998 | -0.221828 |
| 1 | 0.946205 | -2.372516 | -0.755825 |
| 6 | 2.803033 | 0.713677 | 0.705797 |
| 6 | 3.632392 | -2.687783 | -0.630261 |
| 6 | 3.563059 | -0.416702 | 0.309976 |
| 6 | 5.004239 | -2.693582 | -0.513334 |
| 1 | 3.108888 | -3.548930 | -1.037304 |
| 6 | 4.978804 | -0.453595 | 0.418407 |
| 6 | 5.682653 | -1.567146 | 0.016684 |
| 1 | 5.573788 | -3.563455 | -0.827338 |
| 1 | 5.496212 | 0.412093 | 0.823104 |
| 1 | 6.765230 | -1.585661 | 0.102865 |
| 8 | 0.726428 | 1.808802 | 1.068479 |
| 8 | -0.719532 | 1.829714 | -1.013605 |
| 15 | -0.028759 | 2.824794 | 0.058203 |
| 8 | 1.105508 | 3.446166 | -0.894969 |
| 8 | -0.874136 | 3.793967 | 0.780125 |
| 1 | 1.210895 | 4.393035 | -0.715372 |
| 1 | -3.295859 | 1.649679 | -1.076659 |
| 1 | 3.293332 | 1.588020 | 1.121035 |

---

**[**(*R*)-(*S*)**B]**‡

---

Number of imaginary frequencies : 1
The smallest frequencies are : -55.8978 32.1932 66.8974 cm(-1)

Electronic energy : HF=-1412.3174519
Zero-point correction= 0.283284 (Hartree/Particle)
Thermal correction to Energy= 0.300954
Thermal correction to Enthalpy= 0.301899
Thermal correction to Gibbs Free Energy= 0.238542
Sum of electronic and zero-point Energies= -1412.034168
Sum of electronic and thermal Energies= -1412.016498
Sum of electronic and thermal Enthalpies= -1412.015553
Sum of electronic and thermal Free Energies= -1412.078910

............................................................

Cartesian Coordinates

............................................................

| | | | |
|---|---|---|---|
| 6 | 3.089018 | -1.068416 | -0.014109 |
| 6 | 3.575565 | 0.211789 | 0.349705 |
| 6 | 1.674837 | -1.353246 | 0.036563 |
| 6 | 2.694554 | 1.150073 | 0.798020 |
| 6 | 1.304504 | 0.915574 | 0.702332 |
| 6 | 0.713483 | -0.248478 | 0.184125 |
| 6 | -0.756437 | -0.149023 | -0.213939 |
| 6 | -1.863882 | -1.108898 | -0.059966 |
| 6 | -1.190973 | 1.093039 | -0.698519 |
| 6 | -3.212661 | -0.614176 | 0.081982 |
| 6 | -2.532019 | 1.539879 | -0.717476 |
| 6 | -3.517503 | 0.735885 | -0.226497 |
| 8 | 0.545189 | 2.012308 | 1.144604 |
| 15 | 0.149404 | 3.106617 | 0.018252 |
| 8 | -0.281922 | 2.069043 | -1.146693 |
| 1 | -2.724569 | 2.549902 | -1.059374 |
| 1 | -4.539838 | 1.090612 | -0.138293 |
| 1 | 3.017796 | 2.113706 | 1.172660 |
| 1 | 4.643687 | 0.406243 | 0.332278 |
| 6 | -4.277389 | -1.496314 | 0.408914 |
| 1 | -5.263457 | -1.066307 | 0.563083 |
| 6 | 4.026377 | -2.094294 | -0.311019 |
| 1 | 5.074435 | -1.816832 | -0.388382 |

| | | | |
|---|---|---|---|
| 6 | 3.639613 | -3.407279 | -0.438410 |
| 6 | 2.291769 | -3.728613 | -0.189100 |
| 6 | -4.091097 | -2.857462 | 0.467452 |
| 1 | -4.913984 | -3.523468 | 0.708251 |
| 6 | -2.829946 | -3.369569 | 0.108436 |
| 1 | 1.979244 | -4.768177 | -0.145986 |
| 1 | 4.365901 | -4.184888 | -0.654298 |
| 6 | 1.360034 | -2.737339 | 0.047275 |
| 1 | -2.688850 | -4.441062 | -0.001546 |
| 6 | -1.770947 | -2.522692 | -0.154637 |
| 1 | -0.879535 | -2.960896 | -0.559849 |
| 1 | 0.383069 | -3.044837 | 0.361886 |
| 8 | -0.803078 | 4.126678 | 0.496011 |
| 8 | 1.511516 | 3.675871 | -0.614186 |
| 1 | 1.569990 | 4.632922 | -0.468908 |

---

(*S*)-**B**

---

Number of imaginary frequencies : 0
The smallest frequencies are : 47.9870   56.4468   68.1014  cm(-1)

Electronic energy :        HF=-1412.377115
Zero-point correction=                          0.283918 (Hartree/Particle)
Thermal correction to Energy=                   0.302374
Thermal correction to Enthalpy=                 0.303319
Thermal correction to Gibbs Free Energy=        0.238056
Sum of electronic and zero-point Energies=         -1412.093197
Sum of electronic and thermal Energies=            -1412.074741
Sum of electronic and thermal Enthalpies=          -1412.073796
Sum of electronic and thermal Free Energies=       -1412.139059

Cartesian Coordinates

| | | | |
|---|---|---|---|
| 6 | -0.330486 | -3.140539 | 0.192906 |
| 6 | 0.856289 | -3.336137 | 0.948301 |
| 6 | -0.696177 | -1.816083 | -0.223812 |
| 6 | 1.671797 | -2.275689 | 1.255578 |
| 6 | 1.299037 | -0.978060 | 0.851753 |
| 6 | 0.123388 | -0.700697 | 0.172237 |
| 6 | -0.218329 | 0.709869 | -0.161759 |
| 6 | -1.461273 | 1.325021 | 0.224048 |
| 6 | 0.706537 | 1.498342 | -0.827347 |
| 6 | -1.735977 | 2.672963 | -0.187738 |
| 6 | 0.451459 | 2.824998 | -1.227395 |
| 6 | -0.762209 | 3.393196 | -0.929896 |
| 8 | 2.168775 | 0.061409 | 1.201222 |
| 15 | 3.035046 | 0.790187 | 0.038230 |
| 8 | 1.959518 | 0.966310 | -1.161833 |
| 1 | 1.225814 | 3.364006 | -1.761710 |
| 1 | -0.979655 | 4.412271 | -1.236552 |
| 1 | 2.598796 | -2.401546 | 1.803877 |
| 1 | 1.123665 | -4.341711 | 1.260221 |
| 6 | -2.969133 | 3.276702 | 0.180312 |
| 1 | -3.165979 | 4.294199 | -0.147094 |
| 6 | -1.147966 | -4.241022 | -0.183347 |
| 1 | -0.862210 | -5.235817 | 0.148605 |
| 6 | -2.268092 | -4.057892 | -0.960636 |
| 6 | -2.608816 | -2.759087 | -1.406846 |
| 6 | -3.888717 | 2.599022 | 0.946777 |
| 1 | -4.825184 | 3.072836 | 1.225809 |
| 6 | -3.603543 | 1.285357 | 1.388373 |

| | | | |
|---|---|---|---|
| 1 | -3.478898 | -2.619388 | -2.041820 |
| 1 | -2.883336 | -4.906665 | -1.244484 |
| 6 | -1.844798 | -1.669672 | -1.050784 |
| 1 | -4.319067 | 0.761744 | 2.015753 |
| 6 | -2.424039 | 0.666051 | 1.038763 |
| 1 | -2.219136 | -0.335865 | 1.395458 |
| 1 | -2.116671 | -0.685257 | -1.411243 |
| 8 | 3.743715 | 1.982672 | 0.539025 |
| 8 | 3.952274 | -0.350233 | -0.624971 |
| 1 | 4.888200 | -0.172734 | -0.444744 |

---

(*R*)-**B**

---

Number of imaginary frequencies : 0
The smallest frequencies are : 49.5381 56.6055 65.7479 cm(-1)

Electronic energy : HF=-1412.3762841
Zero-point correction= 0.283907 (Hartree/Particle)
Thermal correction to Energy= 0.302319
Thermal correction to Enthalpy= 0.303263
Thermal correction to Gibbs Free Energy= 0.238113
Sum of electronic and zero-point Energies= -1412.092377
Sum of electronic and thermal Energies= -1412.073965
Sum of electronic and thermal Enthalpies= -1412.073021
Sum of electronic and thermal Free Energies= -1412.138171

Cartesian Coordinates

| | | | |
|---|---|---|---|
| 6 | -1.235940 | 2.155474 | -1.028232 |
| 6 | -0.094953 | 1.930068 | -0.208175 |
| 6 | 0.325646 | 0.612871 | 0.192611 |
| 6 | -0.436310 | -0.621106 | -0.147418 |
| 6 | -1.810349 | -0.823037 | 0.229484 |
| 6 | -2.526601 | 0.098046 | 1.043608 |
| 6 | -3.843246 | -0.125516 | 1.380575 |
| 6 | -4.519040 | -1.282587 | 0.925762 |
| 6 | -3.849357 | -2.208726 | 0.160083 |
| 6 | -2.486447 | -2.016528 | -0.195158 |
| 6 | -1.778327 | -2.998951 | -0.937454 |
| 6 | -0.446703 | -2.833186 | -1.226752 |
| 6 | 0.203743 | -1.652695 | -0.817102 |
| 6 | -1.610277 | 3.430227 | -1.391551 |
| 6 | -0.864079 | 4.553261 | -0.961891 |
| 6 | 0.260635 | 4.371997 | -0.191134 |
| 6 | 0.681916 | 3.069802 | 0.193751 |
| 6 | 1.871795 | 2.879651 | 0.945617 |
| 6 | 2.302519 | 1.615575 | 1.265730 |
| 6 | 1.528101 | 0.503187 | 0.875298 |
| 8 | 2.021350 | -0.758847 | 1.222799 |
| 8 | 1.557974 | -1.527257 | -1.153598 |
| 1 | -1.813697 | 1.308308 | -1.376771 |
| 1 | -2.022012 | 0.983352 | 1.410939 |
| 1 | -4.365847 | 0.590948 | 2.007677 |
| 1 | -5.558964 | -1.442671 | 1.194635 |
| 1 | -4.349869 | -3.112396 | -0.177598 |
| 1 | -2.298992 | -3.898411 | -1.253112 |
| 1 | 0.125460 | -3.582188 | -1.762808 |
| 1 | -2.484369 | 3.574160 | -2.019945 |
| 1 | -1.174331 | 5.552688 | -1.252074 |
| 1 | 0.853708 | 5.224342 | 0.129872 |
| 1 | 2.449588 | 3.748210 | 1.248572 |

| | | | |
|---|---|---|---|
| 1 | 3.219216 | 1.440698 | 1.818430 |
| 15 | 2.661898 | -1.673517 | 0.038515 |
| 8 | 3.880559 | -0.844600 | -0.592991 |
| 1 | 3.595662 | -0.025107 | -1.028094 |
| 8 | 3.022570 | -3.020065 | 0.503369 |

---

(*S*,*R*,*S*)-**1a**endo-*re*

---

Number of imaginary frequencies : 0
The smallest frequencies are : 8.8165 12.9652 14.4348 cm(-1)

Electronic energy : HF=-6614.0203631
Zero-point correction= 1.889974 (Hartree/Particle)
Thermal correction to Energy= 2.000924
Thermal correction to Enthalpy= 2.001868
Thermal correction to Gibbs Free Energy= 1.733381
Sum of electronic and zero-point Energies= -6612.130389
Sum of electronic and thermal Energies= -6612.019439
Sum of electronic and thermal Enthalpies= -6612.018495
Sum of electronic and thermal Free Energies= -6612.286982

Cartesian Coordinates

| | | | |
|---|---|---|---|
| 6 | 5.620407 | -1.584731 | 2.117655 |
| 6 | 6.556983 | -1.562223 | 1.068657 |
| 6 | 7.909110 | -1.779047 | 1.366938 |
| 6 | 8.314016 | -2.017700 | 2.682851 |
| 6 | 7.374885 | -2.048186 | 3.715899 |
| 6 | 6.024130 | -1.833127 | 3.429683 |
| 15 | 5.905029 | -1.204254 | -0.625270 |
| 6 | 7.396289 | -0.744913 | -1.617779 |
| 6 | 8.362006 | -1.689672 | -2.007880 |
| 6 | 9.479160 | -1.297146 | -2.744962 |
| 6 | 9.644779 | 0.042344 | -3.109500 |
| 6 | 8.684144 | 0.984447 | -2.740446 |
| 6 | 7.564541 | 0.590839 | -2.004851 |
| 46 | 4.010609 | 0.209803 | -0.540860 |
| 6 | 2.418391 | -1.278454 | -0.894845 |
| 6 | 1.789875 | -0.024875 | -0.805272 |
| 15 | 4.589130 | 2.415961 | 0.057931 |
| 6 | 5.582479 | -2.896967 | -1.303947 |
| 6 | 5.189955 | -2.987878 | -2.651202 |
| 6 | 4.894108 | -4.223222 | -3.223396 |
| 6 | 4.952968 | -5.388088 | -2.449962 |
| 6 | 5.321710 | -5.305922 | -1.107463 |
| 6 | 5.646373 | -4.069799 | -0.539413 |
| 6 | 6.379214 | 2.701735 | 0.455166 |
| 6 | 6.957827 | 1.848874 | 1.410178 |
| 6 | 8.313860 | 1.936739 | 1.716385 |
| 6 | 9.119214 | 2.877908 | 1.067879 |
| 6 | 8.553929 | 3.734403 | 0.123056 |
| 6 | 7.191250 | 3.650989 | -0.180951 |
| 6 | 4.297948 | 3.686308 | -1.253857 |
| 6 | 4.326343 | 3.244786 | -2.586620 |
| 6 | 4.217719 | 4.153834 | -3.640977 |
| 6 | 4.066164 | 5.517170 | -3.376249 |
| 6 | 4.014614 | 5.964843 | -2.053376 |
| 6 | 4.131340 | 5.056607 | -0.998831 |
| 1 | 2.615414 | -1.663057 | -1.894967 |
| 1 | 5.119848 | -2.085407 | -3.253436 |
| 1 | 5.938861 | -4.023390 | 0.504408 |

| | | | |
|---|---|---|---|
| 1 | 4.602396 | -4.276419 | -4.268685 |
| 1 | 5.350477 | -6.201118 | -0.493260 |
| 1 | 4.702416 | -6.349005 | -2.889974 |
| 1 | 8.236267 | -2.734638 | -1.740566 |
| 1 | 6.820157 | 1.324819 | -1.725308 |
| 1 | 10.218438 | -2.037534 | -3.038244 |
| 1 | 8.800723 | 2.026945 | -3.022224 |
| 1 | 10.515445 | 0.346098 | -3.684217 |
| 1 | 8.654037 | -1.745632 | 0.579597 |
| 1 | 4.575172 | -1.381415 | 1.905875 |
| 1 | 9.367121 | -2.176067 | 2.899175 |
| 1 | 5.285566 | -1.841657 | 4.226382 |
| 1 | 7.693813 | -2.230357 | 4.738335 |
| 1 | 6.346356 | 1.099942 | 1.902475 |
| 1 | 6.770572 | 4.321622 | -0.922639 |
| 1 | 8.740922 | 1.258656 | 2.449384 |
| 1 | 9.171743 | 4.470951 | -0.383916 |
| 1 | 10.179576 | 2.939860 | 1.296278 |
| 1 | 4.430926 | 2.182235 | -2.792101 |
| 1 | 4.095744 | 5.418041 | 0.023633 |
| 1 | 4.241572 | 3.795832 | -4.666420 |
| 1 | 3.879865 | 7.021649 | -1.840914 |
| 1 | 3.973509 | 6.225272 | -4.194713 |
| 1 | 1.248409 | 0.231286 | 0.098971 |
| 6 | 1.307362 | 0.659877 | -2.050874 |
| 6 | 2.424263 | -2.343966 | 0.190015 |
| 6 | 1.218705 | -3.370453 | 0.222461 |
| 1 | 3.335261 | -2.926657 | 0.058459 |
| 1 | 2.479682 | -1.865981 | 1.171640 |
| 6 | 0.205663 | -2.929620 | 1.323109 |
| 1 | 0.686321 | -3.112940 | 2.284138 |
| 1 | -0.694782 | -3.550125 | 1.279146 |
| 6 | 1.693830 | -4.758446 | 0.701790 |
| 6 | 2.738472 | -4.887880 | 1.631358 |
| 6 | 1.023563 | -5.924344 | 0.302216 |
| 6 | 3.121774 | -6.139750 | 2.115753 |
| 1 | 3.264823 | -4.008243 | 1.986499 |
| 6 | 1.402204 | -7.177826 | 0.787186 |
| 1 | 0.200165 | -5.849590 | -0.399964 |
| 6 | 2.458810 | -7.293241 | 1.691917 |
| 1 | 3.940850 | -6.210098 | 2.826692 |
| 1 | 0.869237 | -8.064657 | 0.454173 |
| 1 | 2.758390 | -8.268187 | 2.066433 |
| 6 | 0.607939 | -3.473262 | -1.178199 |
| 6 | 1.418715 | -3.944960 | -2.226525 |
| 6 | -0.695486 | -3.072739 | -1.488797 |
| 6 | 0.959085 | -3.974428 | -3.540310 |
| 1 | 2.426326 | -4.280839 | -2.005624 |
| 6 | -1.157475 | -3.084961 | -2.812784 |
| 1 | -1.383706 | -2.744452 | -0.723472 |
| 6 | -0.331711 | -3.527876 | -3.844340 |
| 1 | 1.613304 | -4.337232 | -4.328679 |
| 1 | -2.170264 | -2.756113 | -3.021046 |
| 1 | -0.690131 | -3.534676 | -4.869855 |
| 7 | -0.122025 | -1.509531 | 1.263904 |
| 1 | -0.839116 | -1.215130 | 0.592420 |
| 16 | -0.223876 | -0.568100 | 2.615998 |
| 8 | 0.782702 | -1.040797 | 3.581177 |
| 1 | 2.019880 | 0.552342 | -2.876867 |
| 1 | 1.109665 | 1.720916 | -1.895199 |
| 8 | -0.163245 | 0.821795 | 2.135047 |
| 8 | 0.029991 | 0.095040 | -2.513142 |

| | | | |
|---|---|---|---|
| 1 | -1.313412 | 0.651353 | -2.291627 |
| 6 | 3.797983 | 3.088036 | 1.587088 |
| 6 | 4.367305 | 4.135445 | 2.334339 |
| 6 | 2.611868 | 2.496155 | 2.044425 |
| 6 | 3.750511 | 4.588812 | 3.500185 |
| 1 | 5.302753 | 4.585198 | 2.015073 |
| 6 | 2.004123 | 2.940428 | 3.221167 |
| 1 | 2.157527 | 1.682391 | 1.492789 |
| 6 | 2.568890 | 3.989181 | 3.947868 |
| 1 | 4.200073 | 5.400170 | 4.066436 |
| 1 | 1.093001 | 2.455195 | 3.551143 |
| 1 | 2.097390 | 4.333510 | 4.864554 |
| 1 | 0.036115 | -0.861718 | -2.342568 |
| 6 | -1.893420 | -0.806610 | 3.280838 |
| 6 | -2.268208 | -1.955502 | 4.052423 |
| 6 | -2.831361 | 0.108787 | 2.850444 |
| 6 | -1.367776 | -2.948034 | 4.532841 |
| 6 | -3.668842 | -2.116493 | 4.345881 |
| 6 | -4.198272 | -0.059944 | 3.161405 |
| 1 | -2.524054 | 0.944084 | 2.238317 |
| 6 | -1.827059 | -4.044080 | 5.231219 |
| 1 | -0.307465 | -2.828975 | 4.359656 |
| 6 | -4.102134 | -3.258196 | 5.072551 |
| 6 | -4.607513 | -1.153342 | 3.886191 |
| 1 | -4.918783 | 0.661825 | 2.792174 |
| 6 | -3.205151 | -4.208814 | 5.502327 |
| 1 | -1.117521 | -4.788517 | 5.580708 |
| 1 | -5.164342 | -3.368468 | 5.274376 |
| 1 | -5.660553 | -1.307262 | 4.106010 |
| 1 | -3.549921 | -5.080733 | 6.050335 |
| 6 | -4.274408 | 2.569299 | -0.209791 |
| 6 | -3.797119 | 3.853413 | -0.367075 |
| 6 | -4.737325 | 4.905049 | -0.607304 |
| 6 | -4.327253 | 6.250092 | -0.816892 |
| 1 | -3.268105 | 6.481874 | -0.796565 |
| 6 | -5.255486 | 7.242154 | -1.044882 |
| 1 | -4.924819 | 8.264419 | -1.204315 |
| 6 | -6.639217 | 6.940755 | -1.076748 |
| 1 | -7.359291 | 7.733561 | -1.257519 |
| 6 | -7.068510 | 5.647853 | -0.881149 |
| 1 | -8.128391 | 5.407805 | -0.906175 |
| 6 | -6.137714 | 4.599559 | -0.642564 |
| 6 | -6.563341 | 3.263109 | -0.440190 |
| 1 | -7.625598 | 3.038429 | -0.475462 |
| 6 | -5.661424 | 2.238516 | -0.222200 |
| 6 | -6.122296 | 0.851226 | 0.030142 |
| 6 | -7.172584 | 0.589648 | 0.889193 |
| 1 | -7.648872 | 1.411786 | 1.415660 |
| 6 | -7.631126 | -0.731261 | 1.120406 |
| 6 | -8.701372 | -0.993141 | 2.019575 |
| 1 | -9.167316 | -0.155046 | 2.531535 |
| 6 | -9.141448 | -2.278967 | 2.236732 |
| 1 | -9.959284 | -2.467668 | 2.926144 |
| 6 | -8.531663 | -3.360056 | 1.555177 |
| 1 | -8.888365 | -4.371827 | 1.725111 |
| 6 | -7.491097 | -3.140883 | 0.679369 |
| 1 | -7.029311 | -3.975111 | 0.164260 |
| 6 | -7.001262 | -1.827347 | 0.442671 |
| 6 | -5.903049 | -1.567158 | -0.442546 |
| 6 | -5.501610 | -0.258126 | -0.613443 |
| 6 | -2.326618 | 4.113841 | -0.264395 |
| 6 | -1.552680 | 4.165368 | -1.468323 |

| | | | |
|---|---|---|---|
| 6 | -2.129077 | 4.000689 | -2.759564 |
| 1 | -3.197400 | 3.836343 | -2.842939 |
| 6 | -1.348604 | 4.021959 | -3.893731 |
| 1 | -1.810210 | 3.883765 | -4.867499 |
| 6 | 0.052460 | 4.207562 | -3.800498 |
| 1 | 0.660890 | 4.213942 | -4.700692 |
| 6 | 0.642340 | 4.376697 | -2.568763 |
| 1 | 1.713354 | 4.515356 | -2.483384 |
| 6 | -0.137051 | 4.365201 | -1.380597 |
| 6 | 0.451140 | 4.529939 | -0.101951 |
| 1 | 1.524885 | 4.673247 | -0.030664 |
| 6 | -0.318963 | 4.489259 | 1.034214 |
| 1 | 0.165695 | 4.601837 | 1.996002 |
| 6 | -1.720596 | 4.262179 | 0.981994 |
| 6 | -5.240823 | -2.677800 | -1.199505 |
| 6 | -4.381814 | -3.591235 | -0.496987 |
| 6 | -4.054720 | -3.434078 | 0.879569 |
| 1 | -4.436431 | -2.578612 | 1.423750 |
| 6 | -3.245447 | -4.343716 | 1.524718 |
| 1 | -3.011891 | -4.206903 | 2.573920 |
| 6 | -2.700731 | -5.449958 | 0.828413 |
| 1 | -2.048001 | -6.145357 | 1.347807 |
| 6 | -2.978442 | -5.620277 | -0.508365 |
| 1 | -2.552861 | -6.453450 | -1.061938 |
| 6 | -3.820941 | -4.707923 | -1.198667 |
| 6 | -4.104708 | -4.861849 | -2.579534 |
| 1 | -3.667896 | -5.700467 | -3.115613 |
| 6 | -4.899965 | -3.955214 | -3.233426 |
| 1 | -5.093051 | -4.083104 | -4.294288 |
| 6 | -5.487737 | -2.847355 | -2.559883 |
| 8 | -2.306702 | 0.938112 | -2.195002 |
| 8 | -2.361011 | -0.743036 | -0.192295 |
| 8 | -3.355881 | 1.567725 | 0.102958 |
| 8 | -4.487000 | 0.015900 | -1.533005 |
| 15 | -3.016989 | 0.361272 | -0.933635 |
| 6 | -6.351437 | -1.892166 | -3.364082 |
| 6 | -5.526470 | -1.152484 | -4.443084 |
| 6 | -7.577137 | -2.584694 | -4.000110 |
| 1 | -6.744002 | -1.127402 | -2.687123 |
| 6 | -6.395854 | -0.146865 | -5.210496 |
| 1 | -5.109746 | -1.884395 | -5.149117 |
| 1 | -4.680839 | -0.645554 | -3.968761 |
| 6 | -8.443040 | -1.577822 | -4.772875 |
| 1 | -7.241873 | -3.373524 | -4.687093 |
| 1 | -8.167247 | -3.078050 | -3.218272 |
| 6 | -7.623610 | -0.827062 | -5.833179 |
| 1 | -5.799865 | 0.351132 | -5.984888 |
| 1 | -6.728387 | 0.639058 | -4.517281 |
| 1 | -9.291843 | -2.092773 | -5.239469 |
| 1 | -8.867406 | -0.851943 | -4.064365 |
| 1 | -8.251804 | -0.088345 | -6.345965 |
| 1 | -7.288240 | -1.540950 | -6.599701 |
| 6 | -2.530486 | 4.193335 | 2.268883 |
| 6 | -1.772027 | 3.592127 | 3.469151 |
| 6 | -3.110022 | 5.572717 | 2.662526 |
| 1 | -3.388415 | 3.535996 | 2.082055 |
| 6 | -2.701906 | 3.445152 | 4.681648 |
| 1 | -0.939191 | 4.250554 | 3.750173 |
| 1 | -1.331677 | 2.630798 | 3.193453 |
| 6 | -4.030743 | 5.456655 | 3.886373 |
| 1 | -2.276327 | 6.254729 | 2.883191 |
| 1 | -3.657510 | 6.005366 | 1.819459 |

| | | | |
|---|---|---|---|
| 6 | -3.319249 | 4.795406 | 5.076246 |
| 1 | -2.150007 | 3.016214 | 5.526881 |
| 1 | -3.501955 | 2.730649 | 4.440717 |
| 1 | -4.408498 | 6.447054 | 4.169325 |
| 1 | -4.908517 | 4.854404 | 3.609675 |
| 1 | -4.015923 | 4.669694 | 5.914460 |
| 1 | -2.519492 | 5.462423 | 5.429682 |

---------------------------------------------------------------------------------------------------------------------

(*S*,*R*,*S*)-**1a**exo-*si*

---------------------------------------------------------------------------------------------------------------------

Number of imaginary frequencies : 0
The smallest frequencies are : 7.3219 10.8779 13.2252 cm(-1)

Electronic energy : HF=-6614.0212447
Zero-point correction= 1.889791 (Hartree/Particle)
Thermal correction to Energy= 2.000693
Thermal correction to Enthalpy= 2.001637
Thermal correction to Gibbs Free Energy= 1.732156
Sum of electronic and zero-point Energies= -6612.131454
Sum of electronic and thermal Energies= -6612.020552
Sum of electronic and thermal Enthalpies= -6612.019608
Sum of electronic and thermal Free Energies= -6612.289089

.............................................................

Cartesian Coordinates

.............................................................

| | | | |
|---|---|---|---|
| 6 | 5.757418 | 3.096932 | -0.308613 |
| 6 | 5.252347 | 2.518538 | -1.488075 |
| 6 | 4.979745 | 3.346034 | -2.585734 |
| 6 | 5.237192 | 4.718439 | -2.516686 |
| 6 | 5.777389 | 5.277139 | -1.357073 |
| 6 | 6.029629 | 4.463224 | -0.248330 |
| 15 | 4.996385 | 0.687537 | -1.483840 |
| 6 | 4.181006 | 0.316673 | -3.097067 |
| 6 | 4.880202 | 0.358633 | -4.317122 |
| 6 | 4.225551 | 0.075598 | -5.515132 |
| 6 | 2.865270 | -0.250785 | -5.510961 |
| 6 | 2.164550 | -0.293092 | -4.306168 |
| 6 | 2.820283 | -0.017768 | -3.104027 |
| 46 | 3.860028 | 0.047417 | 0.487212 |
| 6 | 1.925701 | 1.204319 | 0.411110 |
| 6 | 2.020479 | 0.472457 | 1.606042 |
| 15 | 5.192331 | -1.358047 | 1.823920 |
| 6 | 6.712418 | 0.068341 | -1.821041 |
| 6 | 6.870710 | -1.314853 | -2.015046 |
| 6 | 8.136780 | -1.875190 | -2.170965 |
| 6 | 9.272654 | -1.060227 | -2.139698 |
| 6 | 9.126437 | 0.317152 | -1.970040 |
| 6 | 7.855802 | 0.879802 | -1.813876 |
| 6 | 6.717784 | -0.527505 | 2.463088 |
| 6 | 7.737343 | -0.219519 | 1.545068 |
| 6 | 8.858744 | 0.505902 | 1.943307 |
| 6 | 8.979645 | 0.945358 | 3.264536 |
| 6 | 7.974147 | 0.642316 | 4.183787 |
| 6 | 6.852203 | -0.091595 | 3.789341 |
| 6 | 4.447428 | -2.086588 | 3.357182 |
| 6 | 3.649270 | -1.253470 | 4.159821 |
| 6 | 3.038567 | -1.742327 | 5.314581 |
| 6 | 3.206068 | -3.079482 | 5.681921 |
| 6 | 3.994079 | -3.918187 | 4.891140 |
| 6 | 4.611807 | -3.426993 | 3.738170 |
| 1 | 1.321196 | 0.760038 | -0.370146 |

| | | | |
|---|---|---|---|
| 1 | 5.999212 | -1.959580 | -2.029388 |
| 1 | 7.763948 | 1.951322 | -1.676092 |
| 1 | 8.233915 | -2.949023 | -2.301069 |
| 1 | 10.001811 | 0.961260 | -1.958021 |
| 1 | 10.261641 | -1.496018 | -2.250570 |
| 1 | 5.936866 | 0.608623 | -4.327608 |
| 1 | 2.262516 | -0.059351 | -2.176407 |
| 1 | 4.776188 | 0.110428 | -6.451477 |
| 1 | 1.107157 | -0.527494 | -4.283048 |
| 1 | 2.355513 | -0.468170 | -6.445862 |
| 1 | 4.568364 | 2.923486 | -3.496337 |
| 1 | 5.945106 | 2.469264 | 0.559180 |
| 1 | 5.018180 | 5.348432 | -3.374324 |
| 1 | 6.428718 | 4.893654 | 0.665820 |
| 1 | 5.988572 | 6.341710 | -1.310917 |
| 1 | 7.658626 | -0.553202 | 0.517857 |
| 1 | 6.086933 | -0.324185 | 4.522297 |
| 1 | 9.633064 | 0.728517 | 1.214602 |
| 1 | 8.061445 | 0.971619 | 5.215764 |
| 1 | 9.850852 | 1.515579 | 3.574775 |
| 1 | 3.496482 | -0.219219 | 3.870057 |
| 1 | 5.218105 | -4.094976 | 3.136082 |
| 1 | 2.420329 | -1.081886 | 5.915915 |
| 1 | 4.129897 | -4.960030 | 5.168887 |
| 1 | 2.721435 | -3.465811 | 6.574097 |
| 1 | 2.298093 | 0.991036 | 2.521555 |
| 6 | 1.178849 | -0.752221 | 1.819112 |
| 6 | 2.144685 | 2.700423 | 0.287084 |
| 6 | 0.919762 | 3.630525 | 0.625553 |
| 1 | 2.977892 | 3.002481 | 0.928199 |
| 1 | 2.457256 | 2.914147 | -0.738567 |
| 6 | -0.146571 | 3.563989 | -0.512304 |
| 1 | 0.308999 | 3.997232 | -1.403668 |
| 1 | -1.006526 | 4.186965 | -0.245191 |
| 6 | 1.336024 | 5.118041 | 0.637507 |
| 6 | 2.490231 | 5.584425 | -0.005095 |
| 6 | 0.483415 | 6.064365 | 1.232627 |
| 6 | 2.790447 | 6.948849 | -0.037408 |
| 1 | 3.170725 | 4.894841 | -0.487259 |
| 6 | 0.777561 | 7.426698 | 1.194656 |
| 1 | -0.417168 | 5.725959 | 1.736122 |
| 6 | 1.938062 | 7.876908 | 0.559986 |
| 1 | 3.697473 | 7.278533 | -0.536339 |
| 1 | 0.101676 | 8.136031 | 1.665243 |
| 1 | 2.173343 | 8.937404 | 0.533136 |
| 6 | 0.367952 | 3.266488 | 2.011953 |
| 6 | 1.228529 | 3.354796 | 3.121538 |
| 6 | -0.949551 | 2.853023 | 2.242537 |
| 6 | 0.805797 | 3.000427 | 4.399802 |
| 1 | 2.245166 | 3.708281 | 2.977161 |
| 6 | -1.376334 | 2.480541 | 3.525471 |
| 1 | -1.676960 | 2.814901 | 1.443517 |
| 6 | -0.500145 | 2.543898 | 4.606305 |
| 1 | 1.497282 | 3.075796 | 5.234894 |
| 1 | -2.398696 | 2.145093 | 3.663996 |
| 1 | -0.830008 | 2.249828 | 5.598631 |
| 7 | -0.584030 | 2.221046 | -0.857177 |
| 1 | -1.157678 | 1.665677 | -0.216741 |
| 16 | -0.561713 | 1.577245 | -2.364922 |
| 8 | 0.318858 | 2.402977 | -3.206491 |
| 1 | 0.844515 | -1.171181 | 0.863414 |
| 1 | 1.682028 | -1.534518 | 2.385962 |

| | | | |
|---|---|---|---|
| 8 | -0.272162 | 0.142414 | -2.205820 |
| 8 | -0.000211 | -0.408412 | 2.629977 |
| 1 | -1.191456 | -1.187358 | 2.321519 |
| 6 | 5.843102 | -2.851849 | 0.959902 |
| 6 | 7.069496 | -3.465054 | 1.254144 |
| 6 | 5.031066 | -3.404966 | -0.041573 |
| 6 | 7.472087 | -4.606385 | 0.557057 |
| 1 | 7.715812 | -3.047656 | 2.019900 |
| 6 | 5.424272 | -4.554696 | -0.727003 |
| 1 | 4.099634 | -2.907055 | -0.294174 |
| 6 | 6.650314 | -5.156077 | -0.430520 |
| 1 | 8.428265 | -5.067954 | 0.787780 |
| 1 | 4.781997 | -4.971133 | -1.498158 |
| 1 | 6.965468 | -6.045775 | -0.968461 |
| 1 | -0.259012 | 0.501973 | 2.399261 |
| 6 | -2.267533 | 1.682149 | -2.957634 |
| 6 | -2.851013 | 2.913009 | -3.403794 |
| 6 | -3.024426 | 0.539462 | -2.804098 |
| 6 | -2.137884 | 4.130363 | -3.595855 |
| 6 | -4.269189 | 2.913540 | -3.649093 |
| 6 | -4.408632 | 0.553176 | -3.084638 |
| 1 | -2.561473 | -0.363207 | -2.430092 |
| 6 | -2.793987 | 5.282092 | -3.974861 |
| 1 | -1.066073 | 4.146335 | -3.454162 |
| 6 | -4.908410 | 4.121758 | -4.036859 |
| 6 | -5.019963 | 1.718183 | -3.481247 |
| 1 | -4.984879 | -0.353991 | -2.946226 |
| 6 | -4.191434 | 5.285670 | -4.191540 |
| 1 | -2.226831 | 6.198520 | -4.110185 |
| 1 | -5.982189 | 4.106716 | -4.203908 |
| 1 | -6.091873 | 1.745854 | -3.657457 |
| 1 | -4.692610 | 6.204031 | -4.482927 |
| 6 | -3.626664 | -2.840414 | -0.479939 |
| 6 | -2.914768 | -4.005148 | -0.677417 |
| 6 | -3.637020 | -5.237741 | -0.768786 |
| 6 | -2.978862 | -6.488954 | -0.920225 |
| 1 | -1.895710 | -6.507384 | -0.967189 |
| 6 | -3.700527 | -7.659684 | -1.000480 |
| 1 | -3.181181 | -8.607015 | -1.112640 |
| 6 | -5.115350 | -7.640219 | -0.935208 |
| 1 | -5.670697 | -8.571379 | -1.000143 |
| 6 | -5.782592 | -6.445574 | -0.788671 |
| 1 | -6.868092 | -6.421496 | -0.736512 |
| 6 | -5.069066 | -5.218660 | -0.701373 |
| 6 | -5.741844 | -3.980229 | -0.555398 |
| 1 | -6.827256 | -3.972980 | -0.512699 |
| 6 | -5.052559 | -2.787420 | -0.445557 |
| 6 | -5.769353 | -1.491253 | -0.357020 |
| 6 | -6.839376 | -1.203570 | -1.182484 |
| 1 | -7.154953 | -1.934102 | -1.921917 |
| 6 | -7.509745 | 0.043739 | -1.119583 |
| 6 | -8.583078 | 0.347992 | -2.001445 |
| 1 | -8.890393 | -0.402590 | -2.725182 |
| 6 | -9.221005 | 1.566491 | -1.942559 |
| 1 | -10.038150 | 1.788977 | -2.622614 |
| 6 | -8.816733 | 2.531440 | -0.988199 |
| 1 | -9.328881 | 3.488149 | -0.941883 |
| 6 | -7.780972 | 2.268207 | -0.118871 |
| 1 | -7.476675 | 3.013967 | 0.606305 |
| 6 | -7.088375 | 1.027622 | -0.164084 |
| 6 | -5.986257 | 0.733612 | 0.704438 |
| 6 | -5.367754 | -0.492773 | 0.575168 |

| | | | |
|---|---|---|---|
| 6 | -1.426891 | -3.956715 | -0.845903 |
| 6 | -0.587173 | -4.196932 | 0.289756 |
| 6 | -1.112904 | -4.457310 | 1.586353 |
| 1 | -2.187611 | -4.475335 | 1.724782 |
| 6 | -0.278684 | -4.651065 | 2.664491 |
| 1 | -0.703069 | -4.830612 | 3.648292 |
| 6 | 1.127495 | -4.605327 | 2.505617 |
| 1 | 1.776291 | -4.741271 | 3.365096 |
| 6 | 1.671202 | -4.371286 | 1.263038 |
| 1 | 2.748540 | -4.335717 | 1.134061 |
| 6 | 0.837137 | -4.159503 | 0.130961 |
| 6 | 1.366957 | -3.896194 | -1.157878 |
| 1 | 2.443610 | -3.875035 | -1.293154 |
| 6 | 0.537789 | -3.662444 | -2.227760 |
| 1 | 0.978120 | -3.447775 | -3.195113 |
| 6 | -0.877611 | -3.676997 | -2.096308 |
| 6 | -5.540951 | 1.699923 | 1.759712 |
| 6 | -4.826692 | 2.885162 | 1.372428 |
| 6 | -4.455477 | 3.152076 | 0.024654 |
| 1 | -4.692335 | 2.427769 | -0.745647 |
| 6 | -3.794139 | 4.313092 | -0.311697 |
| 1 | -3.527269 | 4.499989 | -1.344284 |
| 6 | -3.448310 | 5.263036 | 0.679490 |
| 1 | -2.916798 | 6.167190 | 0.396729 |
| 6 | -3.769663 | 5.025784 | 1.996381 |
| 1 | -3.494281 | 5.736359 | 2.771580 |
| 6 | -4.463195 | 3.844645 | 2.373587 |
| 6 | -4.791419 | 3.579061 | 3.727804 |
| 1 | -4.506416 | 4.300790 | 4.489046 |
| 6 | -5.444609 | 2.423122 | 4.073175 |
| 1 | -5.677500 | 2.233984 | 5.116710 |
| 6 | -5.836039 | 1.461145 | 3.099671 |
| 8 | -2.102268 | -1.617200 | 2.039644 |
| 8 | -2.279583 | 0.572858 | 0.641207 |
| 8 | -2.914006 | -1.641037 | -0.437695 |
| 8 | -4.351407 | -0.824805 | 1.469623 |
| 15 | -2.818247 | -0.776956 | 0.937900 |
| 6 | -6.555798 | 0.210990 | 3.574442 |
| 6 | -5.662348 | -0.646937 | 4.499452 |
| 6 | -7.902625 | 0.526738 | 4.262527 |
| 1 | -6.791049 | -0.405773 | 2.701919 |
| 6 | -6.382901 | -1.933456 | 4.926789 |
| 1 | -5.398695 | -0.066128 | 5.394061 |
| 1 | -4.727299 | -0.883595 | 3.982975 |
| 6 | -8.619615 | -0.761521 | 4.694716 |
| 1 | -7.728264 | 1.157028 | 5.145120 |
| 1 | -8.535616 | 1.108050 | 3.581085 |
| 6 | -7.729002 | -1.626177 | 5.599199 |
| 1 | -5.742815 | -2.516233 | 5.600304 |
| 1 | -6.553733 | -2.558762 | 4.038757 |
| 1 | -9.558778 | -0.515356 | 5.205311 |
| 1 | -8.891366 | -1.338017 | 3.798860 |
| 1 | -8.246073 | -2.556505 | 5.864520 |
| 1 | -7.544689 | -1.089019 | 6.540949 |
| 6 | -1.757672 | -3.415706 | -3.311022 |
| 6 | -1.195358 | -2.362366 | -4.285818 |
| 6 | -2.081996 | -4.716104 | -4.084282 |
| 1 | -2.713118 | -3.021017 | -2.945320 |
| 6 | -2.195247 | -2.070788 | -5.413988 |
| 1 | -0.264434 | -2.730928 | -4.736999 |
| 1 | -0.945373 | -1.449971 | -3.740528 |
| 6 | -3.071355 | -4.443882 | -5.227968 |

| | | | |
|---|---|---|---|
| 1 | -1.146052 | -5.127829 | -4.488258 |
| 1 | -2.490534 | -5.468936 | -3.403058 |
| 6 | -2.559295 | -3.351834 | -6.178911 |
| 1 | -1.778959 | -1.321017 | -6.097837 |
| 1 | -3.105278 | -1.626592 | -4.986141 |
| 1 | -3.271541 | -5.369869 | -5.781168 |
| 1 | -4.030022 | -4.124802 | -4.793549 |
| 1 | -3.307400 | -3.139315 | -6.952775 |
| 1 | -1.664527 | -3.722566 | -6.699670 |

---

(*S*,*R*,*S*)-**1a**exo-*re*

---

Number of imaginary frequencies : 0
The smallest frequencies are : 10.5339 13.5955 16.3175 cm(-1)

Electronic energy : HF=-6614.0115659
Zero-point correction= 1.890161 (Hartree/Particle)
Thermal correction to Energy= 2.000810
Thermal correction to Enthalpy= 2.001754
Thermal correction to Gibbs Free Energy= 1.736268
Sum of electronic and zero-point Energies= -6612.121405
Sum of electronic and thermal Energies= -6612.010756
Sum of electronic and thermal Enthalpies= -6612.009812
Sum of electronic and thermal Free Energies= -6612.275298

.............................................................

Cartesian Coordinates

.............................................................

| | | | |
|---|---|---|---|
| 6 | 5.144499 | 3.114247 | 1.158275 |
| 6 | 5.464507 | 1.845671 | 0.651730 |
| 6 | 6.276519 | 1.760407 | -0.494303 |
| 6 | 6.775612 | 2.913522 | -1.096962 |
| 6 | 6.440087 | 4.173914 | -0.593357 |
| 6 | 5.620018 | 4.269146 | 0.530677 |
| 15 | 4.699037 | 0.303396 | 1.320046 |
| 6 | 6.139172 | -0.803478 | 1.656994 |
| 6 | 7.481444 | -0.397592 | 1.653124 |
| 6 | 8.502951 | -1.333161 | 1.840142 |
| 6 | 8.197983 | -2.681341 | 2.040221 |
| 6 | 6.862141 | -3.091735 | 2.069592 |
| 6 | 5.844804 | -2.158013 | 1.879841 |
| 46 | 3.103843 | -0.434792 | -0.249712 |
| 6 | 1.192574 | 0.758410 | 0.030269 |
| 6 | 1.110860 | -0.048452 | -1.110849 |
| 15 | 4.072983 | -1.988159 | -1.740590 |
| 6 | 4.204856 | 0.832170 | 3.021571 |
| 6 | 2.858988 | 1.137960 | 3.262136 |
| 6 | 2.454049 | 1.644448 | 4.499416 |
| 6 | 3.389750 | 1.825554 | 5.518090 |
| 6 | 4.732241 | 1.500303 | 5.296674 |
| 6 | 5.139832 | 1.014097 | 4.054783 |
| 6 | 5.700511 | -1.507547 | -2.475980 |
| 6 | 5.813854 | -0.861866 | -3.715938 |
| 6 | 7.049441 | -0.380623 | -4.155991 |
| 6 | 8.191614 | -0.540616 | -3.369484 |
| 6 | 8.091607 | -1.194933 | -2.138631 |
| 6 | 6.858646 | -1.670344 | -1.692701 |
| 6 | 4.421685 | -3.667465 | -1.062771 |
| 6 | 3.711496 | -4.054359 | 0.080879 |
| 6 | 3.899474 | -5.315822 | 0.648573 |
| 6 | 4.812018 | -6.205279 | 0.076490 |
| 6 | 5.525518 | -5.830223 | -1.065815 |

| | | | |
|---|---|---|---|
| 6 | 5.328741 | -4.571635 | -1.636797 |
| 1 | 0.704074 | 0.363520 | 0.921987 |
| 1 | 2.126488 | 0.974168 | 2.481594 |
| 1 | 6.186301 | 0.776526 | 3.888356 |
| 1 | 1.409360 | 1.885278 | 4.659936 |
| 1 | 5.463386 | 1.632235 | 6.089753 |
| 1 | 3.075131 | 2.213720 | 6.483142 |
| 1 | 7.735594 | 0.643516 | 1.484480 |
| 1 | 4.811486 | -2.486152 | 1.870338 |
| 1 | 9.539178 | -1.006154 | 1.824194 |
| 1 | 6.609852 | -4.138507 | 2.212840 |
| 1 | 8.995248 | -3.407699 | 2.170616 |
| 1 | 6.516663 | 0.791575 | -0.917588 |
| 1 | 4.517585 | 3.207570 | 2.038676 |
| 1 | 7.411434 | 2.824246 | -1.973408 |
| 1 | 5.338504 | 5.243084 | 0.919028 |
| 1 | 6.809055 | 5.073021 | -1.078141 |
| 1 | 4.942202 | -0.726724 | -4.345373 |
| 1 | 6.799874 | -2.165387 | -0.729397 |
| 1 | 7.115753 | 0.118128 | -5.119228 |
| 1 | 8.970259 | -1.329518 | -1.514387 |
| 1 | 9.150766 | -0.164404 | -3.713744 |
| 1 | 3.023809 | -3.342443 | 0.525977 |
| 1 | 5.886934 | -4.290351 | -2.524497 |
| 1 | 3.344141 | -5.596682 | 1.539161 |
| 1 | 6.234962 | -6.520008 | -1.514368 |
| 1 | 4.968388 | -7.184935 | 0.519217 |
| 1 | 1.272226 | 0.381835 | -2.092408 |
| 6 | 0.307340 | -1.309146 | -1.130328 |
| 6 | 1.454236 | 2.264353 | 0.100897 |
| 6 | 1.103123 | 3.155076 | -1.121284 |
| 1 | 0.862576 | 2.643399 | 0.938967 |
| 1 | 2.495161 | 2.449636 | 0.365679 |
| 6 | -0.417315 | 3.027078 | -1.425563 |
| 1 | -0.613116 | 2.043772 | -1.856285 |
| 1 | -0.732748 | 3.768712 | -2.160129 |
| 6 | 1.925590 | 2.770560 | -2.371679 |
| 6 | 3.311217 | 2.572834 | -2.260089 |
| 6 | 1.351996 | 2.653993 | -3.647184 |
| 6 | 4.090308 | 2.259831 | -3.373135 |
| 1 | 3.794824 | 2.670086 | -1.296640 |
| 6 | 2.131937 | 2.350085 | -4.767669 |
| 1 | 0.286864 | 2.785650 | -3.787918 |
| 6 | 3.505806 | 2.149866 | -4.637687 |
| 1 | 5.154429 | 2.095539 | -3.244342 |
| 1 | 1.657792 | 2.273305 | -5.742907 |
| 1 | 4.112732 | 1.907849 | -5.505873 |
| 6 | 1.453958 | 4.634207 | -0.812264 |
| 6 | 1.203472 | 5.612358 | -1.789829 |
| 6 | 2.054272 | 5.052112 | 0.382463 |
| 6 | 1.540383 | 6.947990 | -1.587461 |
| 1 | 0.751371 | 5.322313 | -2.733042 |
| 6 | 2.405859 | 6.389944 | 0.586030 |
| 1 | 2.251397 | 4.347589 | 1.179617 |
| 6 | 2.152759 | 7.345451 | -0.395291 |
| 1 | 1.330262 | 7.678061 | -2.364340 |
| 1 | 2.862205 | 6.680486 | 1.528125 |
| 1 | 2.423309 | 8.385676 | -0.235729 |
| 7 | -1.268052 | 3.118127 | -0.236962 |
| 1 | -1.833454 | 2.283771 | -0.090788 |
| 16 | -2.154995 | 4.479423 | 0.129286 |
| 8 | -2.063211 | 5.445877 | -0.977000 |

| | | | |
|---|---|---|---|
| 1 | 0.122058 | -1.695618 | -0.123031 |
| 1 | 0.778553 | -2.085816 | -1.733883 |
| 8 | -3.465585 | 4.005489 | 0.601474 |
| 8 | -0.982495 | -1.003689 | -1.777330 |
| 6 | 2.976302 | -2.360073 | -3.186457 |
| 6 | 2.454225 | -1.273920 | -3.911980 |
| 6 | 2.509901 | -3.650627 | -3.482519 |
| 6 | 1.510624 | -1.474286 | -4.917870 |
| 1 | 2.764522 | -0.262839 | -3.671996 |
| 6 | 1.546155 | -3.846838 | -4.476645 |
| 1 | 2.883958 | -4.507180 | -2.932153 |
| 6 | 1.045688 | -2.762322 | -5.199363 |
| 1 | 1.125898 | -0.617458 | -5.463036 |
| 1 | 1.190568 | -4.852507 | -4.685147 |
| 1 | 0.297121 | -2.917421 | -5.971444 |
| 1 | -1.421133 | -1.830037 | -2.027531 |
| 6 | -1.320304 | 5.201132 | 1.552310 |
| 6 | -1.181190 | 4.463992 | 2.773255 |
| 6 | -0.874247 | 6.496378 | 1.429903 |
| 6 | -1.637154 | 3.131893 | 2.966913 |
| 6 | -0.529250 | 5.127485 | 3.868355 |
| 6 | -0.248609 | 7.137943 | 2.524138 |
| 1 | -0.980706 | 7.009949 | 0.481906 |
| 6 | -1.457786 | 2.488991 | 4.171518 |
| 1 | -2.160639 | 2.602351 | 2.186223 |
| 6 | -0.365032 | 4.435940 | 5.099125 |
| 6 | -0.075939 | 6.466397 | 3.710436 |
| 1 | 0.103504 | 8.158216 | 2.410009 |
| 6 | -0.821421 | 3.145323 | 5.251342 |
| 1 | -1.816457 | 1.470568 | 4.271594 |
| 1 | 0.124312 | 4.950783 | 5.922077 |
| 1 | 0.410077 | 6.952762 | 4.552578 |
| 1 | -0.695727 | 2.629695 | 6.199584 |
| 6 | -5.352230 | -0.847585 | -0.543453 |
| 6 | -5.698799 | -0.246709 | -1.736839 |
| 6 | -6.482979 | -0.996939 | -2.674473 |
| 6 | -6.929666 | -0.437967 | -3.902850 |
| 1 | -6.673903 | 0.590098 | -4.133381 |
| 6 | -7.680457 | -1.183075 | -4.785561 |
| 1 | -8.017460 | -0.737368 | -5.717040 |
| 6 | -8.017948 | -2.526525 | -4.490432 |
| 1 | -8.607625 | -3.102541 | -5.197752 |
| 6 | -7.600288 | -3.097934 | -3.310086 |
| 1 | -7.854415 | -4.128093 | -3.073666 |
| 6 | -6.831014 | -2.354281 | -2.373682 |
| 6 | -6.369765 | -2.936399 | -1.167229 |
| 1 | -6.572736 | -3.986889 | -0.980078 |
| 6 | -5.637062 | -2.212671 | -0.245168 |
| 6 | -5.094803 | -2.875179 | 0.966159 |
| 6 | -5.847128 | -3.775305 | 1.697811 |
| 1 | -6.897372 | -3.909738 | 1.455480 |
| 6 | -5.284682 | -4.533999 | 2.754302 |
| 6 | -6.063319 | -5.455720 | 3.506548 |
| 1 | -7.121330 | -5.553038 | 3.277188 |
| 6 | -5.490491 | -6.213567 | 4.502638 |
| 1 | -6.095166 | -6.914566 | 5.070645 |
| 6 | -4.109286 | -6.084667 | 4.789414 |
| 1 | -3.665495 | -6.688715 | 5.575550 |
| 6 | -3.326662 | -5.198813 | 4.081730 |
| 1 | -2.269699 | -5.099706 | 4.304224 |
| 6 | -3.889451 | -4.394939 | 3.054109 |
| 6 | -3.107829 | -3.456105 | 2.310140 |

| | | | |
|---|---|---|---|
| 6 | -3.726482 | -2.704163 | 1.334070 |
| 6 | -5.205109 | 1.124353 | -2.076329 |
| 6 | -4.115213 | 1.223473 | -3.009014 |
| 6 | -3.472124 | 0.083457 | -3.568496 |
| 1 | -3.810168 | -0.906477 | -3.282844 |
| 6 | -2.424678 | 0.214154 | -4.454048 |
| 1 | -1.942473 | -0.672564 | -4.854839 |
| 6 | -1.963871 | 1.495513 | -4.836560 |
| 1 | -1.142113 | 1.587131 | -5.540916 |
| 6 | -2.555468 | 2.621409 | -4.307033 |
| 1 | -2.204656 | 3.612498 | -4.584484 |
| 6 | -3.632835 | 2.518616 | -3.386069 |
| 6 | -4.235478 | 3.667470 | -2.811358 |
| 1 | -3.848825 | 4.649723 | -3.064576 |
| 6 | -5.257330 | 3.538685 | -1.907009 |
| 1 | -5.676367 | 4.429169 | -1.451837 |
| 6 | -5.763749 | 2.266933 | -1.518752 |
| 6 | -1.627600 | -3.342070 | 2.489338 |
| 6 | -1.089035 | -2.477660 | 3.497155 |
| 6 | -1.915233 | -1.772256 | 4.414364 |
| 1 | -2.991912 | -1.857640 | 4.327638 |
| 6 | -1.362995 | -0.984276 | 5.400148 |
| 1 | -2.011654 | -0.462320 | 6.098005 |
| 6 | 0.041488 | -0.835979 | 5.502400 |
| 1 | 0.464963 | -0.201258 | 6.275173 |
| 6 | 0.870087 | -1.486081 | 4.615993 |
| 1 | 1.947613 | -1.365335 | 4.677836 |
| 6 | 0.332456 | -2.324566 | 3.603565 |
| 6 | 1.164626 | -3.031860 | 2.696864 |
| 1 | 2.241691 | -2.897495 | 2.758339 |
| 6 | 0.616916 | -3.879639 | 1.765447 |
| 1 | 1.262416 | -4.422627 | 1.086858 |
| 6 | -0.787162 | -4.063658 | 1.648629 |
| 8 | -2.365808 | 0.434368 | -0.311770 |
| 8 | -2.971284 | 0.315488 | 2.212848 |
| 8 | -4.773530 | -0.070720 | 0.456046 |
| 8 | -2.944061 | -1.805251 | 0.598856 |
| 15 | -3.203445 | -0.204824 | 0.851448 |
| 6 | -1.315567 | -5.006174 | 0.577733 |
| 6 | -1.147967 | -4.395853 | -0.832068 |
| 6 | -0.674921 | -6.410418 | 0.635803 |
| 1 | -2.389882 | -5.143620 | 0.739090 |
| 6 | -1.692915 | -5.315675 | -1.932016 |
| 1 | -0.082993 | -4.201677 | -1.013542 |
| 1 | -1.661485 | -3.429036 | -0.848434 |
| 6 | -1.238068 | -7.325590 | -0.462105 |
| 1 | 0.412329 | -6.331982 | 0.505331 |
| 1 | -0.843879 | -6.848519 | 1.626671 |
| 6 | -1.052154 | -6.708480 | -1.856290 |
| 1 | -1.515778 | -4.865528 | -2.917036 |
| 1 | -2.782367 | -5.408729 | -1.818035 |
| 1 | -0.755365 | -8.309159 | -0.413191 |
| 1 | -2.309582 | -7.491072 | -0.280707 |
| 1 | -1.474658 | -7.366457 | -2.625158 |
| 1 | 0.022976 | -6.619800 | -2.071182 |
| 6 | -6.881124 | 2.202497 | -0.495294 |
| 6 | -6.449502 | 2.801266 | 0.862691 |
| 6 | -8.180306 | 2.871597 | -0.996824 |
| 1 | -7.121111 | 1.148329 | -0.317931 |
| 6 | -7.572423 | 2.675088 | 1.900539 |
| 1 | -6.186551 | 3.858004 | 0.733753 |
| 1 | -5.540836 | 2.303673 | 1.208915 |

| | | | |
|---|---|---|---|
| 6 | -9.301605 | 2.754645 | 0.046994 |
| 1 | -7.988743 | 3.932455 | -1.208030 |
| 1 | -8.489729 | 2.412531 | -1.944148 |
| 6 | -8.870780 | 3.329229 | 1.405059 |
| 1 | -7.256178 | 3.124292 | 2.849796 |
| 1 | -7.757226 | 1.610238 | 2.104552 |
| 1 | -10.206248 | 3.259931 | -0.313806 |
| 1 | -9.563114 | 1.693900 | 0.171896 |
| 1 | -9.672779 | 3.201020 | 2.142971 |
| 1 | -8.709092 | 4.411956 | 1.301442 |
| 1 | -1.817004 | -0.189514 | -0.944648 |

---

(*S*,*R*,*S*)-**1a**endo-*si*

---

Number of imaginary frequencies : 0
The smallest frequencies are : 11.9957 15.5297 16.8420 cm(-1)

Electronic energy : HF=-6614.0126371
Zero-point correction= 1.889104 (Hartree/Particle)
Thermal correction to Energy= 2.000121
Thermal correction to Enthalpy= 2.001065
Thermal correction to Gibbs Free Energy= 1.733838
Sum of electronic and zero-point Energies= -6612.123533
Sum of electronic and thermal Energies= -6612.012516
Sum of electronic and thermal Enthalpies= -6612.011572
Sum of electronic and thermal Free Energies= -6612.278799

............................................................

Cartesian Coordinates

............................................................

| | | | |
|---|---|---|---|
| 6 | -3.282306 | 5.147217 | -0.385064 |
| 6 | -3.761146 | 3.837589 | -0.215335 |
| 6 | -4.462889 | 3.521013 | 0.955934 |
| 6 | -4.701288 | 4.492839 | 1.929326 |
| 6 | -4.229402 | 5.794506 | 1.748474 |
| 6 | -3.517234 | 6.118737 | 0.589746 |
| 15 | -3.510006 | 2.514583 | -1.483298 |
| 6 | -4.780364 | 2.897181 | -2.771941 |
| 6 | -5.508308 | 4.093285 | -2.828970 |
| 6 | -6.484999 | 4.282191 | -3.810740 |
| 6 | -6.742126 | 3.282484 | -4.750554 |
| 6 | -6.017180 | 2.087849 | -4.706302 |
| 6 | -5.048882 | 1.895155 | -3.721384 |
| 46 | -3.598829 | 0.250436 | -0.858604 |
| 6 | -2.485408 | -1.676666 | -0.925036 |
| 6 | -1.626762 | -0.637690 | -1.317993 |
| 15 | -5.610286 | -0.307725 | 0.248783 |
| 6 | -6.636571 | -1.703117 | -0.400385 |
| 6 | -5.986769 | -2.747985 | -1.076209 |
| 6 | -6.707747 | -3.840550 | -1.558450 |
| 6 | -8.091938 | -3.899572 | -1.382743 |
| 6 | -8.750141 | -2.861492 | -0.719450 |
| 6 | -8.028830 | -1.770633 | -0.229759 |
| 6 | -1.939066 | 3.078582 | -2.271354 |
| 6 | -1.867457 | 3.680364 | -3.535765 |
| 6 | -0.631131 | 4.068854 | -4.061945 |
| 6 | 0.537431 | 3.878526 | -3.323529 |
| 6 | 0.471295 | 3.286451 | -2.059127 |
| 6 | -0.754976 | 2.873998 | -1.543205 |
| 6 | -5.230175 | -0.827228 | 1.983699 |
| 6 | -6.000843 | -1.753756 | 2.701460 |
| 6 | -5.652690 | -2.098769 | 4.009894 |

| | | | |
|---|---|---|---|
| 6 | -4.529251 | -1.529221 | 4.613041 |
| 6 | -3.747944 | -0.613783 | 3.902869 |
| 6 | -4.097706 | -0.264657 | 2.598671 |
| 6 | -6.881963 | 1.013809 | 0.487316 |
| 6 | -7.294969 | 1.722103 | -0.653772 |
| 6 | -8.198047 | 2.777906 | -0.546391 |
| 6 | -8.694478 | 3.151300 | 0.705960 |
| 6 | -8.292894 | 2.452345 | 1.845177 |
| 6 | -7.394837 | 1.386201 | 1.738177 |
| 1 | -2.908357 | -2.266511 | -1.738497 |
| 1 | -2.772574 | 3.852388 | -4.109722 |
| 1 | -0.793515 | 2.391975 | -0.571012 |
| 1 | -0.587452 | 4.529007 | -5.045804 |
| 1 | 1.371451 | 3.147857 | -1.474754 |
| 1 | 1.497963 | 4.187451 | -3.726040 |
| 1 | -5.326309 | 4.876245 | -2.100654 |
| 1 | -4.508978 | 0.952905 | -3.668187 |
| 1 | -7.046553 | 5.212199 | -3.836887 |
| 1 | -6.215155 | 1.301479 | -5.429467 |
| 1 | -7.505013 | 3.429875 | -5.509908 |
| 1 | -4.820975 | 2.510812 | 1.107939 |
| 1 | -2.718544 | 5.405890 | -1.276098 |
| 1 | -5.249954 | 4.227824 | 2.828440 |
| 1 | -3.141605 | 7.128225 | 0.445620 |
| 1 | -4.407655 | 6.550879 | 2.507681 |
| 1 | -6.870221 | -2.211873 | 2.240208 |
| 1 | -3.470974 | 0.424858 | 2.039063 |
| 1 | -6.254484 | -2.822577 | 4.552631 |
| 1 | -2.842881 | -0.211109 | 4.344015 |
| 1 | -4.248274 | -1.813757 | 5.622912 |
| 1 | -4.914767 | -2.704977 | -1.226144 |
| 1 | -8.551801 | -0.970705 | 0.284899 |
| 1 | -6.182373 | -4.638144 | -2.075270 |
| 1 | -9.827312 | -2.899067 | -0.581008 |
| 1 | -8.655475 | -4.746979 | -1.763402 |
| 1 | -6.892645 | 1.458478 | -1.626834 |
| 1 | -7.089910 | 0.851671 | 2.632044 |
| 1 | -8.496566 | 3.318780 | -1.439546 |
| 1 | -8.676282 | 2.735016 | 2.821987 |
| 1 | -9.386974 | 3.984013 | 0.792373 |
| 1 | -0.923910 | -0.213219 | -0.604617 |
| 6 | -1.215534 | -0.491476 | -2.750962 |
| 8 | 0.057689 | -1.218703 | -2.937191 |
| 6 | -2.465358 | -2.390760 | 0.407372 |
| 6 | -1.561442 | -3.666631 | 0.458213 |
| 1 | -2.156567 | -1.714826 | 1.207292 |
| 1 | -3.482691 | -2.709275 | 0.645581 |
| 6 | -0.055353 | -3.244575 | 0.499899 |
| 1 | 0.232611 | -2.789826 | -0.449615 |
| 1 | 0.570882 | -4.127433 | 0.654950 |
| 6 | -1.886029 | -4.499875 | -0.797929 |
| 6 | -3.093092 | -5.222224 | -0.826310 |
| 6 | -1.115164 | -4.478133 | -1.968288 |
| 6 | -3.512581 | -5.897319 | -1.971033 |
| 1 | -3.712217 | -5.253144 | 0.064523 |
| 6 | -1.536259 | -5.152747 | -3.119461 |
| 1 | -0.191462 | -3.915441 | -2.016779 |
| 6 | -2.733715 | -5.865156 | -3.131152 |
| 1 | -4.446491 | -6.452865 | -1.953702 |
| 1 | -0.922600 | -5.108575 | -4.014538 |
| 1 | -3.056445 | -6.388353 | -4.027030 |
| 6 | -1.868312 | -4.512413 | 1.708970 |

| | | | |
|---|---|---|---|
| 6 | -1.317984 | -5.801232 | 1.808499 |
| 6 | -2.659051 | -4.052599 | 2.766124 |
| 6 | -1.547570 | -6.602266 | 2.922121 |
| 1 | -0.706791 | -6.182034 | 0.994633 |
| 6 | -2.893666 | -4.855090 | 3.886569 |
| 1 | -3.084897 | -3.059448 | 2.749142 |
| 6 | -2.342341 | -6.130594 | 3.971666 |
| 1 | -1.107500 | -7.594819 | 2.973073 |
| 1 | -3.508999 | -4.466409 | 4.693180 |
| 1 | -2.524381 | -6.752652 | 4.844069 |
| 7 | 0.282928 | -2.220863 | 1.485929 |
| 1 | 0.372949 | -1.261003 | 1.155088 |
| 16 | 0.384415 | -2.414686 | 3.117404 |
| 8 | -0.748031 | -1.741792 | 3.787136 |
| 1 | 0.139565 | -1.521878 | -3.856394 |
| 1 | 1.179308 | -0.301910 | -2.635306 |
| 1 | -1.959451 | -0.932590 | -3.422362 |
| 1 | -1.045186 | 0.550489 | -3.037038 |
| 8 | 0.626663 | -3.837748 | 3.391824 |
| 6 | 1.880080 | -1.477444 | 3.530820 |
| 6 | 2.082102 | -0.906059 | 4.832808 |
| 6 | 2.859339 | -1.416018 | 2.563852 |
| 6 | 1.142415 | -0.941715 | 5.902655 |
| 6 | 3.332210 | -0.228681 | 5.060476 |
| 6 | 4.092647 | -0.783091 | 2.821457 |
| 1 | 2.670704 | -1.828293 | 1.584449 |
| 6 | 1.415787 | -0.328351 | 7.105776 |
| 1 | 0.196079 | -1.444146 | 5.759966 |
| 6 | 3.571500 | 0.401316 | 6.311549 |
| 6 | 4.319139 | -0.192840 | 4.040754 |
| 1 | 4.842026 | -0.749617 | 2.041415 |
| 6 | 2.634307 | 0.357651 | 7.316404 |
| 1 | 0.679003 | -0.369919 | 7.903052 |
| 1 | 4.514889 | 0.922071 | 6.452934 |
| 1 | 5.253618 | 0.322820 | 4.239005 |
| 1 | 2.826073 | 0.843817 | 8.268643 |
| 6 | 4.695037 | -0.757683 | -1.370030 |
| 6 | 4.970605 | -1.698762 | -2.340922 |
| 6 | 6.224330 | -1.622038 | -3.033191 |
| 6 | 6.568299 | -2.525481 | -4.074592 |
| 1 | 5.858242 | -3.294070 | -4.357842 |
| 6 | 7.783375 | -2.431121 | -4.717527 |
| 1 | 8.028034 | -3.128708 | -5.513285 |
| 6 | 8.717531 | -1.432353 | -4.350593 |
| 1 | 9.671549 | -1.370686 | -4.865957 |
| 6 | 8.415726 | -0.543871 | -3.343788 |
| 1 | 9.126105 | 0.225944 | -3.053808 |
| 6 | 7.170731 | -0.613571 | -2.661126 |
| 6 | 6.849119 | 0.287579 | -1.616220 |
| 1 | 7.571715 | 1.049359 | -1.338524 |
| 6 | 5.634359 | 0.237251 | -0.960397 |
| 6 | 5.343447 | 1.154991 | 0.169033 |
| 6 | 6.285645 | 1.378762 | 1.155963 |
| 1 | 7.236322 | 0.855210 | 1.113161 |
| 6 | 6.021916 | 2.232945 | 2.255027 |
| 6 | 6.969099 | 2.411747 | 3.300405 |
| 1 | 7.920668 | 1.890177 | 3.236842 |
| 6 | 6.684421 | 3.221924 | 4.376900 |
| 1 | 7.412574 | 3.347253 | 5.173062 |
| 6 | 5.440794 | 3.896458 | 4.449186 |
| 1 | 5.222544 | 4.530080 | 5.303823 |
| 6 | 4.506286 | 3.753335 | 3.446697 |

| | | | |
|---|---|---|---|
| 1 | 3.553775 | 4.268083 | 3.505840 |
| 6 | 4.763542 | 2.914660 | 2.329289 |
| 6 | 3.802953 | 2.731830 | 1.283415 |
| 6 | 4.097086 | 1.838640 | 0.274499 |
| 6 | 3.978029 | -2.766674 | -2.689858 |
| 6 | 3.098545 | -2.511255 | -3.799846 |
| 6 | 3.145369 | -1.297001 | -4.543206 |
| 1 | 3.861803 | -0.535168 | -4.263295 |
| 6 | 2.278257 | -1.062082 | -5.586984 |
| 1 | 2.327218 | -0.120902 | -6.126182 |
| 6 | 1.313282 | -2.032814 | -5.955221 |
| 1 | 0.634213 | -1.836887 | -6.780052 |
| 6 | 1.250323 | -3.228361 | -5.269907 |
| 1 | 0.523847 | -3.986750 | -5.549188 |
| 6 | 2.134197 | -3.496632 | -4.188241 |
| 6 | 2.082904 | -4.715505 | -3.468011 |
| 1 | 1.357316 | -5.472159 | -3.750175 |
| 6 | 2.939859 | -4.936069 | -2.421118 |
| 1 | 2.881299 | -5.876248 | -1.879464 |
| 6 | 3.914438 | -3.981260 | -2.006499 |
| 6 | 2.569905 | 3.579672 | 1.208197 |
| 6 | 1.347941 | 3.150682 | 1.822892 |
| 6 | 1.237084 | 1.934372 | 2.551686 |
| 1 | 2.109842 | 1.302240 | 2.662825 |
| 6 | 0.032054 | 1.541004 | 3.092407 |
| 1 | -0.042357 | 0.603833 | 3.631530 |
| 6 | -1.123776 | 2.343315 | 2.935708 |
| 1 | -2.069577 | 2.015200 | 3.357699 |
| 6 | -1.049502 | 3.537996 | 2.255871 |
| 1 | -1.928416 | 4.161971 | 2.131821 |
| 6 | 0.179971 | 3.972347 | 1.692878 |
| 6 | 0.275380 | 5.194438 | 0.979700 |
| 1 | -0.616620 | 5.804730 | 0.872449 |
| 6 | 1.463244 | 5.585785 | 0.415736 |
| 1 | 1.507492 | 6.516200 | -0.141690 |
| 6 | 2.635927 | 4.787617 | 0.517344 |
| 8 | 1.936545 | 0.359470 | -2.387625 |
| 8 | 1.268626 | 0.087996 | 0.131930 |
| 8 | 3.484853 | -0.838740 | -0.680064 |
| 8 | 3.181166 | 1.683001 | -0.769682 |
| 15 | 2.331359 | 0.301451 | -0.877505 |
| 6 | 4.753882 | -4.395338 | -0.798232 |
| 6 | 6.276264 | -4.157083 | -0.865467 |
| 6 | 4.190540 | -3.802446 | 0.512397 |
| 1 | 4.629765 | -5.484648 | -0.720941 |
| 6 | 6.972167 | -4.777972 | 0.356284 |
| 1 | 6.489854 | -3.085193 | -0.872116 |
| 1 | 6.685611 | -4.566358 | -1.796006 |
| 6 | 4.889468 | -4.385792 | 1.746966 |
| 1 | 4.333709 | -2.719361 | 0.490373 |
| 1 | 3.109903 | -3.975747 | 0.568308 |
| 6 | 6.411392 | -4.201989 | 1.665692 |
| 1 | 8.053477 | -4.604450 | 0.294558 |
| 1 | 6.827261 | -5.867912 | 0.347819 |
| 1 | 4.490491 | -3.913782 | 2.651944 |
| 1 | 4.656289 | -5.457325 | 1.821790 |
| 1 | 6.899601 | -4.668249 | 2.530034 |
| 1 | 6.646322 | -3.128401 | 1.712990 |
| 6 | 3.906186 | 5.250127 | -0.178796 |
| 6 | 3.794123 | 5.091615 | -1.713347 |
| 6 | 4.314293 | 6.691698 | 0.192206 |
| 1 | 4.730295 | 4.604795 | 0.141993 |

| | | | |
|---|---|---|---|
| 6 | 5.091641 | 5.514135 | -2.417452 |
| 1 | 2.957283 | 5.703483 | -2.077138 |
| 1 | 3.552404 | 4.050548 | -1.953619 |
| 6 | 5.619549 | 7.096077 | -0.509107 |
| 1 | 3.522471 | 7.394067 | -0.099358 |
| 1 | 4.421450 | 6.772229 | 1.280612 |
| 6 | 5.504832 | 6.942049 | -2.032837 |
| 1 | 4.974043 | 5.428952 | -3.504832 |
| 1 | 5.893589 | 4.817108 | -2.134048 |
| 1 | 5.883187 | 8.128213 | -0.247803 |
| 1 | 6.437751 | 6.459904 | -0.142007 |
| 1 | 6.452970 | 7.208085 | -2.516067 |
| 1 | 4.750674 | 7.648474 | -2.409132 |

---

(*S*,*S*,*S*)-**1a**endo-*re*

---

Number of imaginary frequencies : 0
The smallest frequencies are : 10.9652 14.7375 15.6363 cm(-1)

Electronic energy : HF=-6614.0095518
Zero-point correction= 1.890070 (Hartree/Particle)
Thermal correction to Energy= 2.000685
Thermal correction to Enthalpy= 2.001629
Thermal correction to Gibbs Free Energy= 1.736954
Sum of electronic and zero-point Energies= -6612.119481
Sum of electronic and thermal Energies= -6612.008867
Sum of electronic and thermal Enthalpies= -6612.007922
Sum of electronic and thermal Free Energies= -6612.272598

Cartesian Coordinates

| | | | |
|---|---|---|---|
| 6 | -3.110315 | -5.138168 | -0.178197 |
| 6 | -3.397205 | -3.768038 | -0.070344 |
| 6 | -3.881093 | -3.273495 | 1.148797 |
| 6 | -4.084248 | -4.126308 | 2.235468 |
| 6 | -3.807138 | -5.489158 | 2.114234 |
| 6 | -3.323269 | -5.993812 | 0.903414 |
| 15 | -3.138905 | -2.574789 | -1.457724 |
| 6 | -4.414234 | -3.032845 | -2.717308 |
| 6 | -5.185319 | -4.202352 | -2.686557 |
| 6 | -6.162176 | -4.433318 | -3.659818 |
| 6 | -6.380851 | -3.500824 | -4.675084 |
| 6 | -5.613908 | -2.332523 | -4.717582 |
| 6 | -4.642342 | -2.100117 | -3.745035 |
| 46 | -3.300634 | -0.274169 | -0.975298 |
| 6 | -2.343440 | 1.715222 | -1.126061 |
| 6 | -1.379698 | 0.723711 | -1.382149 |
| 15 | -5.438801 | 0.248659 | -0.098625 |
| 6 | -6.494330 | 1.395486 | -1.092223 |
| 6 | -5.854646 | 2.408481 | -1.824684 |
| 6 | -6.595987 | 3.329871 | -2.563991 |
| 6 | -7.989845 | 3.243498 | -2.593874 |
| 6 | -8.636219 | 2.236515 | -1.873441 |
| 6 | -7.895682 | 1.320092 | -1.123352 |
| 6 | -1.586972 | -3.231054 | -2.225569 |
| 6 | -1.582294 | -4.137351 | -3.297541 |
| 6 | -0.375040 | -4.603916 | -3.825061 |
| 6 | 0.839225 | -4.170729 | -3.290207 |
| 6 | 0.845487 | -3.268743 | -2.224032 |
| 6 | -0.358282 | -2.798991 | -1.698691 |
| 6 | -5.300561 | 1.110092 | 1.530812 |

| | | | |
|---|---|---|---|
| 6 | -6.150833 | 2.149312 | 1.934548 |
| 6 | -5.992902 | 2.743361 | 3.189368 |
| 6 | -4.993534 | 2.301039 | 4.058325 |
| 6 | -4.138504 | 1.268096 | 3.664080 |
| 6 | -4.287601 | 0.683766 | 2.406305 |
| 6 | -6.602370 | -1.134283 | 0.293775 |
| 6 | -7.000850 | -1.976800 | -0.758059 |
| 6 | -7.806220 | -3.087280 | -0.511655 |
| 6 | -8.217032 | -3.383223 | 0.790892 |
| 6 | -7.828911 | -2.551550 | 1.841826 |
| 6 | -7.032534 | -1.429972 | 1.595652 |
| 1 | -2.754354 | 2.197299 | -2.013327 |
| 1 | -2.519823 | -4.481557 | -3.723008 |
| 1 | -0.345897 | -2.090223 | -0.876716 |
| 1 | -0.387407 | -5.306965 | -4.654013 |
| 1 | 1.783801 | -2.932866 | -1.801819 |
| 1 | 1.778951 | -4.539572 | -3.690549 |
| 1 | -5.043373 | -4.928795 | -1.894069 |
| 1 | -4.069621 | -1.176034 | -3.763076 |
| 1 | -6.756113 | -5.342369 | -3.617921 |
| 1 | -5.780396 | -1.596924 | -5.499561 |
| 1 | -7.145537 | -3.678931 | -5.425838 |
| 1 | -4.095342 | -2.215755 | 1.243564 |
| 1 | -2.703317 | -5.533787 | -1.103803 |
| 1 | -4.458496 | -3.723152 | 3.172024 |
| 1 | -3.099374 | -7.052639 | 0.805946 |
| 1 | -3.962536 | -6.155366 | 2.958117 |
| 1 | -6.934700 | 2.501314 | 1.271355 |
| 1 | -3.601775 | -0.096761 | 2.089783 |
| 1 | -6.651183 | 3.555317 | 3.485579 |
| 1 | -3.341272 | 0.937023 | 4.321607 |
| 1 | -4.868430 | 2.768391 | 5.030781 |
| 1 | -4.773560 | 2.475097 | -1.813397 |
| 1 | -8.410965 | 0.546405 | -0.563364 |
| 1 | -6.079061 | 4.108083 | -3.118440 |
| 1 | -9.720294 | 2.163939 | -1.891060 |
| 1 | -8.569326 | 3.955210 | -3.175372 |
| 1 | -6.672868 | -1.770809 | -1.771621 |
| 1 | -6.742879 | -0.790912 | 2.423107 |
| 1 | -8.097719 | -3.727806 | -1.338578 |
| 1 | -8.146367 | -2.771032 | 2.857655 |
| 1 | -8.833152 | -4.256993 | 0.984162 |
| 1 | -0.694949 | 0.427003 | -0.590389 |
| 6 | -0.845315 | 0.537090 | -2.766580 |
| 8 | 0.402544 | 1.333687 | -2.852928 |
| 6 | -2.424157 | 2.565021 | 0.119212 |
| 6 | -1.526615 | 3.846771 | 0.061168 |
| 1 | -2.149894 | 1.985840 | 1.003521 |
| 1 | -3.459453 | 2.885605 | 0.260213 |
| 6 | -0.031524 | 3.434323 | 0.253869 |
| 1 | 0.285951 | 2.797701 | -0.573663 |
| 1 | 0.603415 | 4.323740 | 0.258282 |
| 6 | -1.766936 | 4.502458 | -1.311205 |
| 6 | -2.998752 | 5.141817 | -1.540166 |
| 6 | -0.875624 | 4.400582 | -2.386989 |
| 6 | -3.329152 | 5.658051 | -2.791719 |
| 1 | -3.707257 | 5.232770 | -0.722633 |
| 6 | -1.202429 | 4.922969 | -3.643444 |
| 1 | 0.069997 | 3.889335 | -2.270444 |
| 6 | -2.427680 | 5.552992 | -3.855472 |
| 1 | -4.288026 | 6.149837 | -2.933673 |
| 1 | -0.493180 | 4.828300 | -4.461695 |

| | | | |
|---|---|---|---|
| 1 | -2.679042 | 5.956747 | -4.832391 |
| 6 | -1.906004 | 4.848786 | 1.165114 |
| 6 | -1.404978 | 6.160037 | 1.098199 |
| 6 | -2.716285 | 4.509195 | 2.252080 |
| 6 | -1.713267 | 7.102198 | 2.073959 |
| 1 | -0.768776 | 6.442606 | 0.264121 |
| 6 | -3.029715 | 5.453162 | 3.234481 |
| 1 | -3.101638 | 3.504775 | 2.360648 |
| 6 | -2.534627 | 6.751872 | 3.150372 |
| 1 | -1.311620 | 8.109297 | 1.998250 |
| 1 | -3.660538 | 5.158031 | 4.068427 |
| 1 | -2.778289 | 7.484557 | 3.915162 |
| 7 | 0.202943 | 2.634229 | 1.455105 |
| 1 | 0.492298 | 1.666637 | 1.317837 |
| 16 | 0.528680 | 3.269988 | 2.952522 |
| 8 | -0.560447 | 2.943524 | 3.887619 |
| 1 | 0.616684 | 1.548838 | -3.773549 |
| 1 | 1.443559 | 0.454922 | -2.298414 |
| 1 | -1.546461 | 0.914217 | -3.517131 |
| 1 | -0.588382 | -0.500740 | -2.997705 |
| 8 | 0.899481 | 4.682061 | 2.770387 |
| 6 | 2.009982 | 2.340527 | 3.445748 |
| 6 | 2.191098 | 1.735137 | 4.735518 |
| 6 | 3.012672 | 2.323466 | 2.497723 |
| 6 | 1.249896 | 1.759981 | 5.805183 |
| 6 | 3.436335 | 1.045168 | 4.958915 |
| 6 | 4.242899 | 1.688072 | 2.758594 |
| 1 | 2.853782 | 2.782699 | 1.529698 |
| 6 | 1.513762 | 1.121706 | 6.997305 |
| 1 | 0.316575 | 2.285014 | 5.667898 |
| 6 | 3.658651 | 0.375575 | 6.193368 |
| 6 | 4.441044 | 1.049650 | 3.958115 |
| 1 | 5.012501 | 1.685697 | 1.995889 |
| 6 | 2.719222 | 0.409553 | 7.196184 |
| 1 | 0.774847 | 1.158279 | 7.792851 |
| 1 | 4.593096 | -0.163460 | 6.326292 |
| 1 | 5.371722 | 0.528229 | 4.157767 |
| 1 | 2.898843 | -0.104836 | 8.135802 |
| 6 | 4.937589 | 0.663491 | -0.630519 |
| 6 | 5.400821 | 1.453634 | -1.662030 |
| 6 | 6.798525 | 1.438373 | -1.959643 |
| 6 | 7.378735 | 2.296513 | -2.932338 |
| 1 | 6.741707 | 3.002949 | -3.452984 |
| 6 | 8.727932 | 2.239794 | -3.206118 |
| 1 | 9.156791 | 2.907146 | -3.947977 |
| 6 | 9.562454 | 1.318477 | -2.526924 |
| 1 | 10.622919 | 1.280986 | -2.758271 |
| 6 | 9.032162 | 0.478467 | -1.574353 |
| 1 | 9.666922 | -0.227801 | -1.045524 |
| 6 | 7.646386 | 0.519421 | -1.257363 |
| 6 | 7.082995 | -0.346809 | -0.287241 |
| 1 | 7.722237 | -1.085432 | 0.187191 |
| 6 | 5.743488 | -0.286469 | 0.057425 |
| 6 | 5.179937 | -1.206004 | 1.078104 |
| 6 | 5.887690 | -1.496083 | 2.229536 |
| 1 | 6.816043 | -0.968798 | 2.428986 |
| 6 | 5.430210 | -2.450791 | 3.168469 |
| 6 | 6.148917 | -2.712230 | 4.367587 |
| 1 | 7.051064 | -2.140134 | 4.569682 |
| 6 | 5.716988 | -3.674342 | 5.251940 |
| 1 | 6.270790 | -3.864710 | 6.166699 |
| 6 | 4.556564 | -4.432168 | 4.961351 |

| | | | |
|---|---|---|---|
| 1 | 4.231618 | -5.204162 | 5.652766 |
| 6 | 3.837800 | -4.201355 | 3.808821 |
| 1 | 2.955500 | -4.790926 | 3.589067 |
| 6 | 4.232422 | -3.188036 | 2.893496 |
| 6 | 3.479543 | -2.888271 | 1.711331 |
| 6 | 3.940785 | -1.888463 | 0.878429 |
| 6 | 4.422365 | 2.263058 | -2.451874 |
| 6 | 3.915737 | 3.476415 | -1.865409 |
| 6 | 4.325453 | 3.926189 | -0.578897 |
| 1 | 5.063656 | 3.353299 | -0.029139 |
| 6 | 3.790234 | 5.064585 | -0.015577 |
| 1 | 4.098286 | 5.370638 | 0.979624 |
| 6 | 2.829553 | 5.832107 | -0.716365 |
| 1 | 2.408510 | 6.720598 | -0.255778 |
| 6 | 2.427958 | 5.441243 | -1.973828 |
| 1 | 1.684264 | 6.014916 | -2.519335 |
| 6 | 2.950574 | 4.262495 | -2.570951 |
| 6 | 2.525477 | 3.819212 | -3.850098 |
| 1 | 1.805593 | 4.416185 | -4.403358 |
| 6 | 3.014848 | 2.651798 | -4.377995 |
| 1 | 2.675756 | 2.328631 | -5.360143 |
| 6 | 3.963815 | 1.829445 | -3.694349 |
| 6 | 2.312669 | -3.742808 | 1.314133 |
| 6 | 2.577132 | -4.808138 | 0.383082 |
| 6 | 3.846856 | -5.001633 | -0.231780 |
| 1 | 4.667702 | -4.341179 | 0.023610 |
| 6 | 4.048843 | -6.007672 | -1.152034 |
| 1 | 5.025508 | -6.126515 | -1.613159 |
| 6 | 2.996538 | -6.886525 | -1.503154 |
| 1 | 3.167346 | -7.672810 | -2.232995 |
| 6 | 1.760437 | -6.737978 | -0.916206 |
| 1 | 0.939533 | -7.399940 | -1.180267 |
| 6 | 1.520146 | -5.707282 | 0.030508 |
| 6 | 0.245427 | -5.527067 | 0.622809 |
| 1 | -0.557395 | -6.208287 | 0.361142 |
| 6 | 0.013891 | -4.490950 | 1.489932 |
| 1 | -0.979936 | -4.363441 | 1.906007 |
| 6 | 1.039079 | -3.567876 | 1.846480 |
| 8 | 2.100492 | -0.262823 | -1.949149 |
| 8 | 1.349051 | 0.093397 | 0.534281 |
| 8 | 3.634666 | 0.868618 | -0.190768 |
| 8 | 3.212742 | -1.623553 | -0.286447 |
| 15 | 2.441089 | -0.201714 | -0.425613 |
| 6 | 4.360368 | 0.565505 | -4.468818 |
| 6 | 4.831424 | -0.692747 | -3.710721 |
| 6 | 5.377517 | 0.902803 | -5.589663 |
| 1 | 3.434461 | 0.261292 | -4.980652 |
| 6 | 4.969652 | -1.877797 | -4.680003 |
| 1 | 5.806981 | -0.519829 | -3.249791 |
| 1 | 4.131039 | -0.941012 | -2.913713 |
| 6 | 5.563534 | -0.280133 | -6.551716 |
| 1 | 6.338304 | 1.157822 | -5.126415 |
| 1 | 5.048443 | 1.790457 | -6.143179 |
| 6 | 5.963692 | -1.560910 | -5.805930 |
| 1 | 5.290328 | -2.768076 | -4.125570 |
| 1 | 3.986477 | -2.116432 | -5.111283 |
| 1 | 6.313391 | -0.029037 | -7.311945 |
| 1 | 4.619547 | -0.455885 | -7.087626 |
| 1 | 6.036822 | -2.402024 | -6.506291 |
| 1 | 6.963507 | -1.424299 | -5.369406 |
| 6 | 0.696291 | -2.425942 | 2.784435 |
| 6 | 0.429316 | -2.902766 | 4.231560 |

| | | | |
|---|---|---|---|
| 6 | -0.515152 | -1.601362 | 2.293010 |
| 1 | 1.552927 | -1.743294 | 2.813335 |
| 6 | 0.153894 | -1.713558 | 5.165404 |
| 1 | -0.432735 | -3.585399 | 4.227608 |
| 1 | 1.285429 | -3.474389 | 4.604800 |
| 6 | -0.721980 | -0.386107 | 3.201756 |
| 1 | -1.418673 | -2.224950 | 2.304674 |
| 1 | -0.354591 | -1.281020 | 1.263856 |
| 6 | -0.983874 | -0.819899 | 4.648862 |
| 1 | -0.069905 | -2.074401 | 6.177469 |
| 1 | 1.066892 | -1.111383 | 5.245710 |
| 1 | -1.531128 | 0.248076 | 2.829911 |
| 1 | 0.182582 | 0.230727 | 3.178082 |
| 1 | -1.090181 | 0.059563 | 5.294022 |
| 1 | -1.933941 | -1.373710 | 4.696094 |

---

(*S*,*S*,*S*)-**1a**exo-*si*

---

Number of imaginary frequencies : 0
The smallest frequencies are : 9.4287 13.0029 14.7659 cm(-1)

Electronic energy : HF=-6614.0073204
Zero-point correction= 1.891315 (Hartree/Particle)
Thermal correction to Energy= 2.001688
Thermal correction to Enthalpy= 2.002632
Thermal correction to Gibbs Free Energy= 1.737569
Sum of electronic and zero-point Energies= -6612.116006
Sum of electronic and thermal Energies= -6612.005632
Sum of electronic and thermal Enthalpies= -6612.004688
Sum of electronic and thermal Free Energies= -6612.269751

Cartesian Coordinates

| | | | |
|---|---|---|---|
| 6 | -4.214254 | 4.001064 | -0.018043 |
| 6 | -4.753675 | 2.727593 | -0.245821 |
| 6 | -5.431338 | 2.486583 | -1.456350 |
| 6 | -5.578106 | 3.500407 | -2.401529 |
| 6 | -5.026695 | 4.764861 | -2.168215 |
| 6 | -4.343278 | 5.009729 | -0.976922 |
| 15 | -4.424328 | 1.277427 | 0.852727 |
| 6 | -6.097326 | 0.826617 | 1.494681 |
| 6 | -7.262027 | 1.565477 | 1.246144 |
| 6 | -8.499684 | 1.111045 | 1.710237 |
| 6 | -8.589142 | -0.083949 | 2.426446 |
| 6 | -7.429598 | -0.818218 | 2.694995 |
| 6 | -6.195547 | -0.364364 | 2.232981 |
| 46 | -3.181918 | -0.204061 | -0.518393 |
| 6 | -1.147848 | 0.764218 | -0.659589 |
| 6 | -1.282648 | -0.182253 | -1.684156 |
| 15 | -4.634547 | -1.836950 | -1.376260 |
| 6 | -3.650705 | 2.015999 | 2.354670 |
| 6 | -2.327010 | 1.674408 | 2.660806 |
| 6 | -1.687065 | 2.238867 | 3.766307 |
| 6 | -2.367636 | 3.146224 | 4.578290 |
| 6 | -3.694842 | 3.480747 | 4.291541 |
| 6 | -4.335252 | 2.915910 | 3.188819 |
| 6 | -6.175414 | -1.134759 | -2.130868 |
| 6 | -6.221234 | -0.768264 | -3.485564 |
| 6 | -7.321331 | -0.080379 | -4.000149 |
| 6 | -8.395656 | 0.253492 | -3.172289 |
| 6 | -8.362477 | -0.111920 | -1.825291 |

| | | | |
|---|---|---|---|
| 6 | -7.262466 | -0.796134 | -1.306462 |
| 6 | -5.284048 | -3.020325 | -0.118055 |
| 6 | -4.570584 | -3.135445 | 1.083602 |
| 6 | -4.994238 | -4.010505 | 2.085619 |
| 6 | -6.149481 | -4.773147 | 1.901181 |
| 6 | -6.876188 | -4.657814 | 0.712619 |
| 6 | -6.445911 | -3.789825 | -0.292241 |
| 1 | -0.552685 | 0.450979 | 0.198026 |
| 1 | -1.800747 | 0.965201 | 2.030221 |
| 1 | -5.367149 | 3.176714 | 2.971076 |
| 1 | -0.663239 | 1.964873 | 3.988501 |
| 1 | -4.230844 | 4.181026 | 4.926602 |
| 1 | -1.862420 | 3.588698 | 5.431024 |
| 1 | -7.209725 | 2.485970 | 0.674496 |
| 1 | -5.303188 | -0.950589 | 2.426035 |
| 1 | -9.395432 | 1.690982 | 1.504464 |
| 1 | -7.487130 | -1.751701 | 3.247383 |
| 1 | -9.554258 | -0.440892 | 2.774875 |
| 1 | -5.836738 | 1.502070 | -1.662812 |
| 1 | -3.658487 | 4.206528 | 0.889435 |
| 1 | -6.110533 | 3.294508 | -3.325989 |
| 1 | -3.882249 | 5.976152 | -0.796996 |
| 1 | -5.119598 | 5.548720 | -2.914676 |
| 1 | -5.398968 | -1.016686 | -4.147975 |
| 1 | -7.249852 | -1.053355 | -0.253697 |
| 1 | -7.336872 | 0.192884 | -5.051770 |
| 1 | -9.185195 | 0.145621 | -1.165098 |
| 1 | -9.249189 | 0.792395 | -3.573650 |
| 1 | -3.692837 | -2.512455 | 1.231373 |
| 1 | -7.023081 | -3.699777 | -1.207521 |
| 1 | -4.431604 | -4.084602 | 3.012240 |
| 1 | -7.779989 | -5.243011 | 0.567639 |
| 1 | -6.487015 | -5.449205 | 2.681769 |
| 1 | -1.544477 | 0.143550 | -2.688930 |
| 6 | -0.643042 | -1.532569 | -1.592431 |
| 6 | -1.366369 | 2.261576 | -0.774157 |
| 6 | -0.276568 | 3.111290 | -1.511501 |
| 1 | -1.465722 | 2.638535 | 0.242670 |
| 1 | -2.314177 | 2.473187 | -1.264178 |
| 6 | 1.130149 | 2.769493 | -0.958345 |
| 1 | 1.387720 | 1.749712 | -1.230961 |
| 1 | 1.870971 | 3.431202 | -1.410694 |
| 6 | -0.356532 | 2.750027 | -3.005796 |
| 6 | -1.579563 | 2.909707 | -3.685591 |
| 6 | 0.717870 | 2.210973 | -3.725023 |
| 6 | -1.730012 | 2.514925 | -5.012765 |
| 1 | -2.425526 | 3.353858 | -3.170133 |
| 6 | 0.569699 | 1.807877 | -5.056769 |
| 1 | 1.687490 | 2.086437 | -3.260224 |
| 6 | -0.654899 | 1.948410 | -5.705832 |
| 1 | -2.688923 | 2.650721 | -5.506376 |
| 1 | 1.419694 | 1.376116 | -5.577913 |
| 1 | -0.771171 | 1.632460 | -6.739015 |
| 6 | -0.552721 | 4.610233 | -1.269153 |
| 6 | -0.428976 | 5.557498 | -2.295821 |
| 6 | -0.870093 | 5.085728 | 0.015194 |
| 6 | -0.642602 | 6.917573 | -2.061549 |
| 1 | -0.165402 | 5.232646 | -3.296365 |
| 6 | -1.082992 | 6.443394 | 0.254332 |
| 1 | -0.930120 | 4.398404 | 0.848502 |
| 6 | -0.978065 | 7.369694 | -0.785109 |
| 1 | -0.543431 | 7.623547 | -2.882022 |

| | | | |
|---|---|---|---|
| 1 | -1.327920 | 6.772797 | 1.260900 |
| 1 | -1.146911 | 8.427344 | -0.601245 |
| 7 | 1.200545 | 2.867984 | 0.515148 |
| 1 | 1.246939 | 1.945157 | 0.952833 |
| 16 | 2.408926 | 3.851762 | 1.151568 |
| 8 | 2.440869 | 5.071441 | 0.335030 |
| 1 | -0.402044 | -1.789817 | -0.556103 |
| 1 | -1.278602 | -2.314090 | -2.006024 |
| 8 | 3.652302 | 3.099539 | 1.374671 |
| 8 | 0.602016 | -1.551106 | -2.388568 |
| 1 | 1.644429 | -0.759249 | -1.760788 |
| 6 | -3.997511 | -2.938293 | -2.727550 |
| 6 | -3.206546 | -2.342778 | -3.726639 |
| 6 | -4.229018 | -4.320895 | -2.792820 |
| 6 | -2.682800 | -3.101793 | -4.773559 |
| 1 | -2.987695 | -1.280668 | -3.673015 |
| 6 | -3.694346 | -5.082983 | -3.835034 |
| 1 | -4.822994 | -4.812197 | -2.030033 |
| 6 | -2.925137 | -4.476588 | -4.830760 |
| 1 | -2.074181 | -2.619985 | -5.533473 |
| 1 | -3.881746 | -6.152836 | -3.866903 |
| 1 | -2.510479 | -5.071790 | -5.639436 |
| 1 | 0.885569 | -2.475728 | -2.481755 |
| 6 | 1.726271 | 4.259972 | 2.768463 |
| 6 | 1.690776 | 3.305071 | 3.834431 |
| 6 | 1.272526 | 5.550832 | 2.929089 |
| 6 | 2.107524 | 1.948469 | 3.721625 |
| 6 | 1.157663 | 3.753161 | 5.092762 |
| 6 | 0.756414 | 5.972760 | 4.175445 |
| 1 | 1.315292 | 6.235533 | 2.090107 |
| 6 | 1.989526 | 1.085564 | 4.789361 |
| 1 | 2.527724 | 1.577653 | 2.797504 |
| 6 | 1.061306 | 2.834014 | 6.172847 |
| 6 | 0.706387 | 5.093960 | 5.231340 |
| 1 | 0.405095 | 6.993725 | 4.288896 |
| 6 | 1.462677 | 1.525627 | 6.026376 |
| 1 | 2.303962 | 0.053296 | 4.670363 |
| 1 | 0.653922 | 3.184539 | 7.117711 |
| 1 | 0.312308 | 5.411561 | 6.193387 |
| 1 | 1.373109 | 0.828869 | 6.854640 |
| 6 | 5.303550 | -0.594394 | 0.249249 |
| 6 | 6.147697 | 0.064148 | -0.624032 |
| 6 | 7.487448 | -0.418302 | -0.772674 |
| 6 | 8.451207 | 0.258183 | -1.570707 |
| 1 | 8.160526 | 1.169281 | -2.081376 |
| 6 | 9.734320 | -0.228686 | -1.689847 |
| 1 | 10.458697 | 0.303793 | -2.299355 |
| 6 | 10.119223 | -1.418474 | -1.023998 |
| 1 | 11.133531 | -1.791979 | -1.130561 |
| 6 | 9.210878 | -2.095267 | -0.242824 |
| 1 | 9.496873 | -3.007722 | 0.274063 |
| 6 | 7.881916 | -1.612480 | -0.088753 |
| 6 | 6.931705 | -2.301440 | 0.702213 |
| 1 | 7.216212 | -3.250820 | 1.145692 |
| 6 | 5.648300 | -1.819431 | 0.897164 |
| 6 | 4.681115 | -2.644110 | 1.670589 |
| 6 | 5.077919 | -3.357243 | 2.787556 |
| 1 | 6.054392 | -3.165507 | 3.222346 |
| 6 | 4.264704 | -4.368907 | 3.355573 |
| 6 | 4.696733 | -5.112317 | 4.488723 |
| 1 | 5.643578 | -4.849122 | 4.953129 |
| 6 | 3.941398 | -6.151516 | 4.982072 |

| | | | |
|---|---|---|---|
| 1 | 4.282204 | -6.713200 | 5.846828 |
| 6 | 2.724258 | -6.502915 | 4.349715 |
| 1 | 2.143934 | -7.340040 | 4.726806 |
| 6 | 2.272388 | -5.795482 | 3.257306 |
| 1 | 1.344493 | -6.075393 | 2.772907 |
| 6 | 3.008264 | -4.693717 | 2.743841 |
| 6 | 2.540790 | -3.896848 | 1.647112 |
| 6 | 3.363739 | -2.889985 | 1.178874 |
| 6 | 5.665076 | 1.259933 | -1.387060 |
| 6 | 5.893286 | 2.560958 | -0.836280 |
| 6 | 6.595001 | 2.759273 | 0.384656 |
| 1 | 6.970293 | 1.896491 | 0.923905 |
| 6 | 6.793666 | 4.023271 | 0.889409 |
| 1 | 7.330837 | 4.152428 | 1.825032 |
| 6 | 6.284200 | 5.157591 | 0.213163 |
| 1 | 6.429133 | 6.148209 | 0.634950 |
| 6 | 5.597688 | 5.002070 | -0.967865 |
| 1 | 5.189018 | 5.863986 | -1.488275 |
| 6 | 5.399692 | 3.712405 | -1.529032 |
| 6 | 4.721640 | 3.524574 | -2.758715 |
| 1 | 4.338519 | 4.394332 | -3.286847 |
| 6 | 4.552807 | 2.264996 | -3.284852 |
| 1 | 4.042497 | 2.158506 | -4.235671 |
| 6 | 5.010744 | 1.100115 | -2.610177 |
| 6 | 1.237912 | -4.165088 | 0.957822 |
| 6 | 1.264755 | -4.595258 | -0.419531 |
| 6 | 2.473046 | -4.797755 | -1.145879 |
| 1 | 3.423174 | -4.699421 | -0.634680 |
| 6 | 2.461974 | -5.092681 | -2.492386 |
| 1 | 3.401707 | -5.221816 | -3.021389 |
| 6 | 1.237757 | -5.220323 | -3.192807 |
| 1 | 1.241247 | -5.429194 | -4.258586 |
| 6 | 0.047211 | -5.091436 | -2.511672 |
| 1 | -0.902286 | -5.198232 | -3.028103 |
| 6 | 0.031016 | -4.794136 | -1.120829 |
| 6 | -1.191061 | -4.643546 | -0.416180 |
| 1 | -2.127197 | -4.807436 | -0.941965 |
| 6 | -1.186448 | -4.254120 | 0.896544 |
| 1 | -2.131244 | -4.128731 | 1.408487 |
| 6 | 0.017672 | -3.971006 | 1.602378 |
| 8 | 2.400242 | -0.205338 | -1.302587 |
| 8 | 1.637825 | -0.140189 | 1.197766 |
| 8 | 4.099295 | 0.022973 | 0.580715 |
| 8 | 2.925845 | -2.165910 | 0.075647 |
| 15 | 2.652916 | -0.565860 | 0.196816 |
| 6 | -0.106730 | -3.400949 | 3.006473 |
| 6 | -0.913876 | -4.317469 | 3.954815 |
| 6 | -0.725220 | -1.980776 | 2.976226 |
| 1 | 0.895734 | -3.298194 | 3.432920 |
| 6 | -1.011015 | -3.720969 | 5.365661 |
| 1 | -1.928631 | -4.456335 | 3.557964 |
| 1 | -0.455404 | -5.311141 | 3.994712 |
| 6 | -0.839829 | -1.393011 | 4.389474 |
| 1 | -1.723337 | -2.027481 | 2.518413 |
| 1 | -0.113567 | -1.330002 | 2.343882 |
| 6 | -1.623865 | -2.314275 | 5.332535 |
| 1 | -1.599256 | -4.383680 | 6.012595 |
| 1 | -0.003606 | -3.668189 | 5.802539 |
| 1 | -1.310691 | -0.406518 | 4.344800 |
| 1 | 0.169536 | -1.237521 | 4.793372 |
| 1 | -1.658168 | -1.887055 | 6.342291 |
| 1 | -2.666111 | -2.381853 | 4.985975 |

| | | | |
|---|---|---|---|
| 6 | 4.788209 | -0.277405 | -3.225243 |
| 6 | 6.015557 | -0.795295 | -4.013749 |
| 6 | 3.549113 | -0.388510 | -4.136834 |
| 1 | 4.626865 | -0.974712 | -2.396228 |
| 6 | 5.806845 | -2.255861 | -4.445697 |
| 1 | 6.165034 | -0.158229 | -4.897305 |
| 1 | 6.923391 | -0.714327 | -3.412201 |
| 6 | 3.302449 | -1.851854 | -4.527898 |
| 1 | 3.709839 | 0.195514 | -5.053754 |
| 1 | 2.668752 | 0.026316 | -3.646729 |
| 6 | 4.517517 | -2.439168 | -5.260988 |
| 1 | 6.673256 | -2.607585 | -5.019379 |
| 1 | 5.754300 | -2.880318 | -3.542099 |
| 1 | 2.402408 | -1.931290 | -5.149755 |
| 1 | 3.108228 | -2.437559 | -3.618179 |
| 1 | 4.356275 | -3.501199 | -5.485961 |
| 1 | 4.629390 | -1.928218 | -6.228272 |

---

(*S*,*S*,*S*)-**1a**exo-*re*

---

Number of imaginary frequencies : 0
The smallest frequencies are : 9.2013 11.8039 13.4790 cm(-1)

Electronic energy : HF=-6614.0130064
Zero-point correction= 1.890486 (Hartree/Particle)
Thermal correction to Energy= 2.001237
Thermal correction to Enthalpy= 2.002181
Thermal correction to Gibbs Free Energy= 1.733976
Sum of electronic and zero-point Energies= -6612.122521
Sum of electronic and thermal Energies= -6612.011769
Sum of electronic and thermal Enthalpies= -6612.010825
Sum of electronic and thermal Free Energies= -6612.279030

.............................................................
Cartesian Coordinates
.............................................................

| | | | |
|---|---|---|---|
| 6 | 5.598541 | -3.275988 | 0.540662 |
| 6 | 5.266254 | -3.009895 | -0.800355 |
| 6 | 5.138473 | -4.085092 | -1.690795 |
| 6 | 5.354742 | -5.395566 | -1.253698 |
| 6 | 5.708481 | -5.646644 | 0.072972 |
| 6 | 5.825531 | -4.582186 | 0.971824 |
| 15 | 5.012051 | -1.240747 | -1.281824 |
| 6 | 4.342695 | -1.362072 | -2.995893 |
| 6 | 5.169802 | -1.475945 | -4.127096 |
| 6 | 4.610515 | -1.634384 | -5.395270 |
| 6 | 3.221947 | -1.696761 | -5.546846 |
| 6 | 2.395363 | -1.586462 | -4.427990 |
| 6 | 2.951807 | -1.403348 | -3.160672 |
| 46 | 3.799057 | -0.132205 | 0.401378 |
| 6 | 1.802404 | -1.177049 | 0.543818 |
| 6 | 1.938834 | -0.222282 | 1.563481 |
| 15 | 5.020538 | 1.565264 | 1.486269 |
| 6 | 6.743511 | -0.669275 | -1.600026 |
| 6 | 6.936219 | 0.706317 | -1.808514 |
| 6 | 8.215578 | 1.224072 | -2.001522 |
| 6 | 9.325793 | 0.374760 | -1.976971 |
| 6 | 9.145040 | -0.994459 | -1.775130 |
| 6 | 7.860805 | -1.516440 | -1.592998 |
| 6 | 6.629107 | 1.175661 | 2.307974 |
| 6 | 7.406561 | 0.128454 | 1.791685 |
| 6 | 8.646315 | -0.181443 | 2.352275 |

| | | | |
|---|---|---|---|
| 6 | 9.120339 | 0.544791 | 3.446409 |
| 6 | 8.350306 | 1.584233 | 3.975015 |
| 6 | 7.113514 | 1.899777 | 3.410074 |
| 6 | 4.094505 | 2.392693 | 2.857573 |
| 6 | 3.646764 | 1.583136 | 3.917047 |
| 6 | 2.847595 | 2.111595 | 4.927774 |
| 6 | 2.479047 | 3.460730 | 4.898170 |
| 6 | 2.927307 | 4.275245 | 3.858226 |
| 6 | 3.731631 | 3.746370 | 2.843314 |
| 1 | 1.228211 | -0.861984 | -0.319455 |
| 1 | 6.082387 | 1.375747 | -1.797354 |
| 1 | 7.732542 | -2.582938 | -1.438761 |
| 1 | 8.343483 | 2.292368 | -2.149223 |
| 1 | 10.002846 | -1.661481 | -1.759379 |
| 1 | 10.325213 | 0.778994 | -2.111435 |
| 1 | 6.249185 | -1.447210 | -4.016798 |
| 1 | 2.297579 | -1.298292 | -2.304716 |
| 1 | 5.259273 | -1.717613 | -6.263195 |
| 1 | 1.317863 | -1.634515 | -4.525002 |
| 1 | 2.787635 | -1.829818 | -6.534197 |
| 1 | 4.872364 | -3.903782 | -2.727013 |
| 1 | 5.674938 | -2.453728 | 1.247725 |
| 1 | 5.249106 | -6.218529 | -1.955195 |
| 1 | 6.081848 | -4.770798 | 2.010571 |
| 1 | 5.882079 | -6.664919 | 0.409040 |
| 1 | 7.046244 | -0.437408 | 0.942208 |
| 1 | 6.523920 | 2.710037 | 3.827690 |
| 1 | 9.236737 | -0.990393 | 1.931471 |
| 1 | 8.712334 | 2.151840 | 4.827922 |
| 1 | 10.082882 | 0.301862 | 3.887968 |
| 1 | 3.910599 | 0.529070 | 3.936197 |
| 1 | 4.067338 | 4.391398 | 2.038254 |
| 1 | 2.497755 | 1.467341 | 5.729513 |
| 1 | 2.650371 | 5.325321 | 3.829597 |
| 1 | 1.843116 | 3.870148 | 5.677716 |
| 1 | 2.210004 | -0.536189 | 2.568832 |
| 6 | 1.158577 | 1.056735 | 1.511050 |
| 6 | 1.953057 | -2.675431 | 0.720751 |
| 6 | 0.726250 | -3.460419 | 1.316789 |
| 1 | 2.812073 | -2.883253 | 1.364135 |
| 1 | 2.184268 | -3.110558 | -0.255227 |
| 6 | -0.416746 | -3.601103 | 0.258706 |
| 1 | -0.047494 | -4.267769 | -0.521442 |
| 1 | -1.278223 | -4.095083 | 0.721143 |
| 6 | 1.115091 | -4.925864 | 1.619722 |
| 6 | 2.227360 | -5.550730 | 1.037663 |
| 6 | 0.280100 | -5.701571 | 2.442484 |
| 6 | 2.500008 | -6.899327 | 1.281366 |
| 1 | 2.900059 | -5.000103 | 0.391935 |
| 6 | 0.546886 | -7.049076 | 2.680964 |
| 1 | -0.583377 | -5.238489 | 2.910960 |
| 6 | 1.663652 | -7.656057 | 2.101354 |
| 1 | 3.374449 | -7.352078 | 0.822765 |
| 1 | -0.115554 | -7.622827 | 3.323755 |
| 1 | 1.878760 | -8.704438 | 2.289177 |
| 6 | 0.276218 | -2.791994 | 2.623217 |
| 6 | 1.185099 | -2.716555 | 3.694067 |
| 6 | -0.997143 | -2.242588 | 2.814539 |
| 6 | 0.848402 | -2.085021 | 4.888945 |
| 1 | 2.171061 | -3.158935 | 3.585500 |
| 6 | -1.342091 | -1.603942 | 4.014077 |
| 1 | -1.751127 | -2.295994 | 2.043460 |

| | | | |
|---|---|---|---|
| 6 | -0.418036 | -1.513563 | 5.052309 |
| 1 | 1.576637 | -2.037668 | 5.694399 |
| 1 | -2.336606 | -1.181114 | 4.118997 |
| 1 | -0.680058 | -1.011030 | 5.978863 |
| 7 | -0.849220 | -2.375431 | -0.396769 |
| 1 | -1.346710 | -1.633020 | 0.098197 |
| 16 | -0.635611 | -2.042216 | -1.994249 |
| 8 | 0.304591 | -3.040236 | -2.531889 |
| 1 | 0.848151 | 1.288429 | 0.485590 |
| 1 | 1.700136 | 1.908369 | 1.919617 |
| 8 | -0.308076 | -0.613918 | -2.096025 |
| 8 | -0.039399 | 0.944930 | 2.358854 |
| 1 | -1.161806 | 1.698846 | 1.859760 |
| 6 | 5.428211 | 2.977732 | 0.370164 |
| 6 | 6.611169 | 3.725437 | 0.434566 |
| 6 | 4.480625 | 3.286217 | -0.620591 |
| 6 | 6.840848 | 4.759481 | -0.477790 |
| 1 | 7.359700 | 3.498496 | 1.186737 |
| 6 | 4.703278 | 4.327811 | -1.520665 |
| 1 | 3.576331 | 2.688607 | -0.688748 |
| 6 | 5.889987 | 5.064294 | -1.454043 |
| 1 | 7.766169 | 5.326661 | -0.423957 |
| 1 | 3.956870 | 4.555544 | -2.276175 |
| 1 | 6.072696 | 5.868309 | -2.161390 |
| 1 | -0.336692 | 0.017671 | 2.331384 |
| 6 | -2.256830 | -2.239601 | -2.779226 |
| 6 | -2.741734 | -3.476570 | -3.317682 |
| 6 | -3.046540 | -1.108526 | -2.737357 |
| 6 | -2.009917 | -4.696996 | -3.365036 |
| 6 | -4.089881 | -3.478722 | -3.825667 |
| 6 | -4.370835 | -1.138115 | -3.224911 |
| 1 | -2.644203 | -0.191847 | -2.328454 |
| 6 | -2.578379 | -5.844358 | -3.874240 |
| 1 | -0.990898 | -4.716357 | -3.005401 |
| 6 | -4.635542 | -4.680578 | -4.352574 |
| 6 | -4.877491 | -2.297683 | -3.761908 |
| 1 | -4.978956 | -0.241009 | -3.163802 |
| 6 | -3.900920 | -5.843214 | -4.374895 |
| 1 | -1.998395 | -6.762656 | -3.893408 |
| 1 | -5.654688 | -4.663089 | -4.730092 |
| 1 | -5.897282 | -2.333095 | -4.136210 |
| 1 | -4.331050 | -6.757002 | -4.774466 |
| 6 | -3.576578 | 2.747078 | -1.291505 |
| 6 | -2.763967 | 3.668297 | -1.923699 |
| 6 | -3.371399 | 4.835041 | -2.490341 |
| 6 | -2.631070 | 5.783620 | -3.247556 |
| 1 | -1.576235 | 5.604264 | -3.420500 |
| 6 | -3.240220 | 6.905783 | -3.765002 |
| 1 | -2.657214 | 7.618265 | -4.341384 |
| 6 | -4.621869 | 7.136642 | -3.556218 |
| 1 | -5.087137 | 8.029507 | -3.963663 |
| 6 | -5.373804 | 6.223063 | -2.853651 |
| 1 | -6.438924 | 6.381044 | -2.704964 |
| 6 | -4.778452 | 5.046807 | -2.319831 |
| 6 | -5.551772 | 4.064010 | -1.656198 |
| 1 | -6.618908 | 4.228808 | -1.540359 |
| 6 | -4.989110 | 2.898515 | -1.166562 |
| 6 | -5.855420 | 1.833802 | -0.597838 |
| 6 | -7.053376 | 1.513742 | -1.211862 |
| 1 | -7.315131 | 1.994697 | -2.149691 |
| 6 | -7.957665 | 0.582288 | -0.646739 |
| 6 | -9.197596 | 0.281854 | -1.274678 |

| | | | |
|---|---|---|---|
| 1 | -9.430561 | 0.761999 | -2.221668 |
| 6 | -10.090535 | -0.588244 | -0.692330 |
| 1 | -11.035684 | -0.808673 | -1.179830 |
| 6 | -9.783941 | -1.186760 | 0.553825 |
| 1 | -10.501754 | -1.855535 | 1.019845 |
| 6 | -8.585815 | -0.924667 | 1.181833 |
| 1 | -8.361180 | -1.377179 | 2.140453 |
| 6 | -7.628646 | -0.053247 | 0.595083 |
| 6 | -6.357363 | 0.211161 | 1.202709 |
| 6 | -5.522380 | 1.134446 | 0.599473 |
| 6 | -1.302603 | 3.395801 | -2.113960 |
| 6 | -0.927118 | 2.540015 | -3.205053 |
| 6 | -1.881385 | 1.944366 | -4.075717 |
| 1 | -2.936361 | 2.141369 | -3.919956 |
| 6 | -1.487189 | 1.124131 | -5.110041 |
| 1 | -2.236690 | 0.671990 | -5.753544 |
| 6 | -0.116565 | 0.858509 | -5.335416 |
| 1 | 0.182128 | 0.205309 | -6.150194 |
| 6 | 0.834246 | 1.424587 | -4.517516 |
| 1 | 1.888725 | 1.217194 | -4.672292 |
| 6 | 0.458310 | 2.271361 | -3.443387 |
| 6 | 1.423831 | 2.873781 | -2.597519 |
| 1 | 2.475859 | 2.667011 | -2.775438 |
| 6 | 1.037039 | 3.703202 | -1.573927 |
| 1 | 1.793832 | 4.162659 | -0.948483 |
| 6 | -0.333372 | 3.979219 | -1.301591 |
| 6 | -5.943089 | -0.451459 | 2.482029 |
| 6 | -5.913263 | 0.337518 | 3.684166 |
| 6 | -6.312757 | 1.702925 | 3.718026 |
| 1 | -6.668014 | 2.173445 | 2.808168 |
| 6 | -6.258308 | 2.434292 | 4.884003 |
| 1 | -6.567271 | 3.475804 | 4.880786 |
| 6 | -5.800309 | 1.843346 | 6.085571 |
| 1 | -5.756427 | 2.432818 | 6.996957 |
| 6 | -5.417568 | 0.521467 | 6.093411 |
| 1 | -5.069678 | 0.051105 | 7.009900 |
| 6 | -5.468195 | -0.259985 | 4.907981 |
| 6 | -5.091236 | -1.628148 | 4.902575 |
| 1 | -4.749836 | -2.084399 | 5.828445 |
| 6 | -5.157382 | -2.365347 | 3.747657 |
| 1 | -4.865495 | -3.410726 | 3.768212 |
| 6 | -5.581573 | -1.797110 | 2.512484 |
| 8 | -2.004571 | 2.160551 | 1.439090 |
| 8 | -2.474409 | -0.303040 | 0.769902 |
| 8 | -3.002010 | 1.546137 | -0.879549 |
| 8 | -4.353631 | 1.492729 | 1.263939 |
| 15 | -2.888206 | 1.122043 | 0.681317 |
| 6 | -5.593877 | -2.688453 | 1.281897 |
| 6 | -6.586786 | -3.867482 | 1.411048 |
| 6 | -4.184949 | -3.222095 | 0.939822 |
| 1 | -5.914646 | -2.088650 | 0.424050 |
| 6 | -6.615091 | -4.712607 | 0.129095 |
| 1 | -6.289564 | -4.502602 | 2.256975 |
| 1 | -7.589816 | -3.491910 | 1.637305 |
| 6 | -4.213434 | -4.076199 | -0.330747 |
| 1 | -3.798588 | -3.816390 | 1.779217 |
| 1 | -3.513897 | -2.372506 | 0.804023 |
| 6 | -5.216068 | -5.232216 | -0.230004 |
| 1 | -7.318620 | -5.546578 | 0.245435 |
| 1 | -6.994016 | -4.093623 | -0.696913 |
| 1 | -3.212486 | -4.457935 | -0.557288 |
| 1 | -4.493164 | -3.432593 | -1.171298 |

| | | | |
|---|---|---|---|
| 1 | -5.243819 | -5.789820 | -1.173731 |
| 1 | -4.883084 | -5.937316 | 0.545773 |
| 6 | -0.675497 | 4.916194 | -0.152076 |
| 6 | -0.431579 | 6.399356 | -0.521548 |
| 6 | 0.091880 | 4.594758 | 1.149461 |
| 1 | -1.743081 | 4.801268 | 0.067807 |
| 6 | -0.847634 | 7.339757 | 0.620004 |
| 1 | 0.636396 | 6.535873 | -0.745247 |
| 1 | -0.980759 | 6.657378 | -1.430996 |
| 6 | -0.355479 | 5.508554 | 2.297209 |
| 1 | 1.169685 | 4.732309 | 0.992620 |
| 1 | -0.054354 | 3.550281 | 1.418147 |
| 6 | -0.145542 | 6.986395 | 1.939289 |
| 1 | -0.639901 | 8.380608 | 0.341606 |
| 1 | -1.935273 | 7.263974 | 0.759888 |
| 1 | 0.194288 | 5.250428 | 3.209729 |
| 1 | -1.418404 | 5.327176 | 2.508588 |
| 1 | -0.504810 | 7.635311 | 2.747690 |
| 1 | 0.932555 | 7.179901 | 1.835381 |

---

(*S*,*S*,*S*)-**1a**endo-*si*

---

Number of imaginary frequencies : 0
The smallest frequencies are : 10.1311 12.9496 15.8195 cm(-1)

Electronic energy : HF=-6614.0152556
Zero-point correction= 1.890447 (Hartree/Particle)
Thermal correction to Energy= 2.001015
Thermal correction to Enthalpy= 2.001959
Thermal correction to Gibbs Free Energy= 1.735712
Sum of electronic and zero-point Energies= -6612.124809
Sum of electronic and thermal Energies= -6612.014240
Sum of electronic and thermal Enthalpies= -6612.013296
Sum of electronic and thermal Free Energies= -6612.279544

............................................................
Cartesian Coordinates
............................................................

| | | | |
|---|---|---|---|
| 6 | -5.543218 | -1.522430 | 1.934016 |
| 6 | -6.466741 | -1.510962 | 0.872948 |
| 6 | -7.833103 | -1.625981 | 1.165755 |
| 6 | -8.264958 | -1.742488 | 2.489496 |
| 6 | -7.340060 | -1.749455 | 3.536010 |
| 6 | -5.975410 | -1.644216 | 3.254414 |
| 15 | -5.784708 | -1.247940 | -0.825997 |
| 6 | -7.234994 | -0.752004 | -1.855823 |
| 6 | -8.235700 | -1.660559 | -2.241575 |
| 6 | -9.316937 | -1.235657 | -3.013735 |
| 6 | -9.409482 | 0.099920 | -3.416696 |
| 6 | -8.413193 | 1.005802 | -3.050463 |
| 6 | -7.329650 | 0.580838 | -2.279406 |
| 46 | -3.881076 | 0.143411 | -0.734446 |
| 6 | -2.245603 | -1.259244 | -1.249792 |
| 6 | -1.667724 | -0.001832 | -1.001473 |
| 15 | -4.455806 | 2.211991 | 0.250784 |
| 6 | -5.506671 | -2.970836 | -1.438442 |
| 6 | -5.018878 | -3.116247 | -2.749153 |
| 6 | -4.730863 | -4.378711 | -3.263776 |
| 6 | -4.899614 | -5.517345 | -2.468125 |
| 6 | -5.375846 | -5.381847 | -1.164773 |
| 6 | -5.686156 | -4.117767 | -0.653764 |
| 6 | -6.250753 | 2.674523 | 0.258730 |

| | | | |
|---|---|---|---|
| 6 | -7.136738 | 1.872574 | 0.999755 |
| 6 | -8.511652 | 2.087118 | 0.939001 |
| 6 | -9.030083 | 3.105379 | 0.133737 |
| 6 | -8.159526 | 3.911600 | -0.600195 |
| 6 | -6.778682 | 3.700553 | -0.538158 |
| 6 | -3.703841 | 3.751853 | -0.443813 |
| 6 | -3.336125 | 3.730044 | -1.798222 |
| 6 | -2.862182 | 4.884412 | -2.426054 |
| 6 | -2.733167 | 6.071101 | -1.700528 |
| 6 | -3.078450 | 6.097729 | -0.346452 |
| 6 | -3.566922 | 4.948094 | 0.276664 |
| 1 | -2.404581 | -1.529667 | -2.293904 |
| 1 | -4.856373 | -2.234017 | -3.363389 |
| 1 | -6.056011 | -4.028559 | 0.362644 |
| 1 | -4.359258 | -4.473660 | -4.280512 |
| 1 | -5.497653 | -6.259293 | -0.536886 |
| 1 | -4.651446 | -6.499638 | -2.859134 |
| 1 | -8.164940 | -2.702738 | -1.943973 |
| 1 | -6.555738 | 1.285645 | -1.999506 |
| 1 | -10.084591 | -1.947513 | -3.304801 |
| 1 | -8.474947 | 2.044696 | -3.360464 |
| 1 | -10.251654 | 0.428816 | -4.019427 |
| 1 | -8.566713 | -1.600358 | 0.367213 |
| 1 | -4.483302 | -1.403694 | 1.728942 |
| 1 | -9.327938 | -1.819353 | 2.702119 |
| 1 | -5.245619 | -1.633088 | 4.058924 |
| 1 | -7.680431 | -1.828634 | 4.564687 |
| 1 | -6.749872 | 1.072803 | 1.621723 |
| 1 | -6.117497 | 4.337504 | -1.116668 |
| 1 | -9.176694 | 1.449296 | 1.514163 |
| 1 | -8.551170 | 4.710593 | -1.224274 |
| 1 | -10.102866 | 3.267453 | 0.079434 |
| 1 | -3.423242 | 2.802125 | -2.357435 |
| 1 | -3.834954 | 4.984427 | 1.327311 |
| 1 | -2.583652 | 4.853061 | -3.475464 |
| 1 | -2.964698 | 7.013814 | 0.226118 |
| 1 | -2.355078 | 6.967009 | -2.184215 |
| 1 | -1.165997 | 0.184239 | -0.059039 |
| 6 | -1.161934 | 0.828033 | -2.141233 |
| 6 | -2.228837 | -2.442892 | -0.296735 |
| 6 | -0.987601 | -3.431803 | -0.373553 |
| 1 | -3.119873 | -3.035682 | -0.502472 |
| 1 | -2.306500 | -2.085933 | 0.733059 |
| 6 | 0.007639 | -3.105251 | 0.785350 |
| 1 | -0.464742 | -3.437359 | 1.709199 |
| 1 | 0.926568 | -3.689796 | 0.668819 |
| 6 | -1.438664 | -4.875850 | -0.064482 |
| 6 | -2.420969 | -5.115178 | 0.911630 |
| 6 | -0.819614 | -5.987228 | -0.654317 |
| 6 | -2.793207 | -6.413530 | 1.259570 |
| 1 | -2.904338 | -4.281218 | 1.409856 |
| 6 | -1.191353 | -7.289155 | -0.309871 |
| 1 | -0.045133 | -5.836656 | -1.398760 |
| 6 | -2.184315 | -7.509701 | 0.644925 |
| 1 | -3.562241 | -6.565985 | 2.012277 |
| 1 | -0.699253 | -8.130962 | -0.789842 |
| 1 | -2.475765 | -8.521742 | 0.911987 |
| 6 | -0.358846 | -3.336487 | -1.766656 |
| 6 | -1.120256 | -3.742207 | -2.878051 |
| 6 | 0.910544 | -2.797959 | -2.011144 |
| 6 | -0.642844 | -3.596549 | -4.178534 |
| 1 | -2.105072 | -4.166550 | -2.712717 |

| | | | |
|---|---|---|---|
| 6 | 1.392729 | -2.640663 | -3.318582 |
| 1 | 1.555760 | -2.482154 | -1.204106 |
| 6 | 0.617291 | -3.033925 | -4.408139 |
| 1 | -1.259005 | -3.915946 | -5.015059 |
| 1 | 2.376988 | -2.211262 | -3.471591 |
| 1 | 0.991303 | -2.907996 | -5.420170 |
| 7 | 0.314268 | -1.688065 | 0.920637 |
| 1 | 1.132632 | -1.310497 | 0.430993 |
| 16 | 0.044196 | -0.834291 | 2.306747 |
| 8 | -1.161268 | -1.394985 | 2.947864 |
| 1 | -1.815937 | 0.769643 | -3.018990 |
| 1 | -1.030765 | 1.874608 | -1.864732 |
| 8 | 0.056579 | 0.577958 | 1.906609 |
| 8 | 0.172490 | 0.382228 | -2.575416 |
| 1 | 1.365435 | 0.902167 | -1.947792 |
| 6 | -4.068298 | 2.233525 | 2.058787 |
| 6 | -4.734750 | 3.070773 | 2.971305 |
| 6 | -3.111582 | 1.328765 | 2.543927 |
| 6 | -4.444342 | 3.005199 | 4.334325 |
| 1 | -5.499276 | 3.757370 | 2.620603 |
| 6 | -2.832124 | 1.253640 | 3.909943 |
| 1 | -2.580578 | 0.676099 | 1.862217 |
| 6 | -3.497783 | 2.090854 | 4.806952 |
| 1 | -4.967359 | 3.657747 | 5.028245 |
| 1 | -2.097872 | 0.533690 | 4.253814 |
| 1 | -3.283137 | 2.031177 | 5.870623 |
| 1 | 0.176555 | -0.590394 | -2.597092 |
| 6 | 1.477067 | -1.073547 | 3.386487 |
| 6 | 1.637113 | -2.207031 | 4.250793 |
| 6 | 2.472054 | -0.129312 | 3.236820 |
| 6 | 0.674128 | -3.238214 | 4.441541 |
| 6 | 2.883971 | -2.310702 | 4.961996 |
| 6 | 3.686805 | -0.251913 | 3.945362 |
| 1 | 2.324082 | 0.714642 | 2.576622 |
| 6 | 0.942494 | -4.317865 | 5.255426 |
| 1 | -0.286634 | -3.164427 | 3.951717 |
| 6 | 3.118238 | -3.433268 | 5.800611 |
| 6 | 3.885147 | -1.316414 | 4.790612 |
| 1 | 4.453270 | 0.503609 | 3.808106 |
| 6 | 2.174429 | -4.424702 | 5.940388 |
| 1 | 0.192242 | -5.094379 | 5.374171 |
| 1 | 4.068885 | -3.497508 | 6.323653 |
| 1 | 4.821001 | -1.424591 | 5.332810 |
| 1 | 2.370226 | -5.283588 | 6.575555 |
| 6 | 4.349798 | 2.726486 | 0.314202 |
| 6 | 3.732605 | 3.943441 | 0.535057 |
| 6 | 4.531648 | 5.129200 | 0.471727 |
| 6 | 4.004905 | 6.410479 | 0.786841 |
| 1 | 2.974343 | 6.480465 | 1.114798 |
| 6 | 4.789510 | 7.539093 | 0.696036 |
| 1 | 4.369701 | 8.510046 | 0.942700 |
| 6 | 6.143174 | 7.443432 | 0.290007 |
| 1 | 6.748357 | 8.342275 | 0.215443 |
| 6 | 6.692459 | 6.213701 | 0.006470 |
| 1 | 7.735375 | 6.128961 | -0.288172 |
| 6 | 5.912601 | 5.028167 | 0.101681 |
| 6 | 6.475550 | 3.750696 | -0.136015 |
| 1 | 7.521104 | 3.684182 | -0.422182 |
| 6 | 5.731064 | 2.591212 | -0.010422 |
| 6 | 6.376544 | 1.263062 | -0.171338 |
| 6 | 7.621128 | 1.022035 | 0.380184 |
| 1 | 8.086704 | 1.785213 | 0.996658 |

| | | | |
|---|---|---|---|
| 6 | 8.315180 | -0.191067 | 0.155928 |
| 6 | 9.607982 | -0.413825 | 0.704647 |
| 1 | 10.045790 | 0.356856 | 1.333902 |
| 6 | 10.297296 | -1.574277 | 0.436832 |
| 1 | 11.285470 | -1.733324 | 0.858670 |
| 6 | 9.723890 | -2.558109 | -0.404556 |
| 1 | 10.281631 | -3.462092 | -0.631886 |
| 6 | 8.467271 | -2.377510 | -0.940493 |
| 1 | 8.039469 | -3.130936 | -1.591308 |
| 6 | 7.714887 | -1.204009 | -0.661068 |
| 6 | 6.392719 | -0.997526 | -1.177883 |
| 6 | 5.774389 | 0.214153 | -0.929177 |
| 6 | 2.282979 | 3.976680 | 0.909122 |
| 6 | 1.937718 | 3.622021 | 2.257775 |
| 6 | 2.919311 | 3.373257 | 3.258657 |
| 1 | 3.970106 | 3.450621 | 3.000300 |
| 6 | 2.555432 | 3.049431 | 4.547846 |
| 1 | 3.322705 | 2.867609 | 5.295313 |
| 6 | 1.190231 | 2.942115 | 4.905018 |
| 1 | 0.916438 | 2.668883 | 5.920195 |
| 6 | 0.216140 | 3.182001 | 3.962798 |
| 1 | -0.835548 | 3.100308 | 4.218284 |
| 6 | 0.559972 | 3.535623 | 2.631970 |
| 6 | -0.429651 | 3.804406 | 1.653180 |
| 1 | -1.477060 | 3.701038 | 1.918891 |
| 6 | -0.071236 | 4.199088 | 0.389566 |
| 1 | -0.847504 | 4.427053 | -0.330511 |
| 6 | 1.291602 | 4.311503 | -0.010091 |
| 6 | 5.727052 | -2.044129 | -2.019856 |
| 6 | 5.649096 | -1.829535 | -3.440017 |
| 6 | 6.151454 | -0.656079 | -4.069292 |
| 1 | 6.630292 | 0.105863 | -3.464929 |
| 6 | 6.036396 | -0.470094 | -5.429392 |
| 1 | 6.424186 | 0.437781 | -5.882799 |
| 6 | 5.415281 | -1.448361 | -6.241696 |
| 1 | 5.325437 | -1.285462 | -7.311936 |
| 6 | 4.932866 | -2.603989 | -5.670529 |
| 1 | 4.460684 | -3.368968 | -6.282231 |
| 6 | 5.042814 | -2.827353 | -4.271451 |
| 6 | 4.560057 | -4.017489 | -3.668668 |
| 1 | 4.099450 | -4.776716 | -4.295417 |
| 6 | 4.654335 | -4.199369 | -2.313103 |
| 1 | 4.267959 | -5.113176 | -1.873000 |
| 6 | 5.226681 | -3.216559 | -1.455700 |
| 8 | 2.253930 | 1.353429 | -1.635715 |
| 8 | 2.711956 | -0.717702 | -0.109725 |
| 8 | 3.612312 | 1.562732 | 0.538303 |
| 8 | 4.553397 | 0.474769 | -1.547508 |
| 15 | 3.189461 | 0.572687 | -0.671278 |
| 6 | 5.240745 | -3.495528 | 0.039525 |
| 6 | 6.137719 | -4.699147 | 0.413347 |
| 6 | 3.814620 | -3.717145 | 0.595689 |
| 1 | 5.646598 | -2.614887 | 0.549165 |
| 6 | 6.159920 | -4.929727 | 1.932433 |
| 1 | 5.757815 | -5.601791 | -0.085293 |
| 1 | 7.155668 | -4.539128 | 0.045199 |
| 6 | 3.835770 | -3.951215 | 2.110143 |
| 1 | 3.355947 | -4.583735 | 0.099721 |
| 1 | 3.206404 | -2.842738 | 0.363746 |
| 6 | 4.745320 | -5.123566 | 2.495888 |
| 1 | 6.791340 | -5.795218 | 2.169568 |
| 1 | 6.625677 | -4.059213 | 2.416490 |

| | | | |
|---|---|---|---|
| 1 | 2.818620 | -4.119242 | 2.480804 |
| 1 | 4.192736 | -3.037825 | 2.600740 |
| 1 | 4.777098 | -5.235033 | 3.586690 |
| 1 | 4.322323 | -6.056768 | 2.095900 |
| 6 | 1.592243 | 4.869567 | -1.395136 |
| 6 | 1.229906 | 6.376287 | -1.450546 |
| 6 | 0.868031 | 4.135321 | -2.545569 |
| 1 | 2.668486 | 4.778952 | -1.580381 |
| 6 | 1.568484 | 6.997384 | -2.812721 |
| 1 | 0.154236 | 6.483647 | -1.257109 |
| 1 | 1.747599 | 6.918825 | -0.654349 |
| 6 | 1.209078 | 4.749230 | -3.911448 |
| 1 | -0.215714 | 4.194614 | -2.391992 |
| 1 | 1.140285 | 3.081446 | -2.536581 |
| 6 | 0.877428 | 6.246583 | -3.958951 |
| 1 | 1.284144 | 8.057376 | -2.821716 |
| 1 | 2.657306 | 6.964004 | -2.960713 |
| 1 | 0.671380 | 4.212577 | -4.703182 |
| 1 | 2.280763 | 4.608686 | -4.112179 |
| 1 | 1.164761 | 6.675034 | -4.927164 |
| 1 | -0.210823 | 6.375613 | -3.867210 |

---

(*S*,*R*,*S*)-**[1a-2a]**$^{‡}$endo-*re*

---

Number of imaginary frequencies : 1
The smallest frequencies are : -235.2595 8.3896 14.5500 cm(-1)

Electronic energy : HF=-6614.0075724
Zero-point correction= 1.888479 (Hartree/Particle)
Thermal correction to Energy= 1.998951
Thermal correction to Enthalpy= 1.999896
Thermal correction to Gibbs Free Energy= 1.734648
Sum of electronic and zero-point Energies= -6612.119094
Sum of electronic and thermal Energies= -6612.008621
Sum of electronic and thermal Enthalpies= -6612.007677
Sum of electronic and thermal Free Energies= -6612.272924

............................................................

Cartesian Coordinates

............................................................

| | | | |
|---|---|---|---|
| 6 | 5.301607 | -1.371518 | 2.288161 |
| 6 | 6.319382 | -1.411635 | 1.318146 |
| 6 | 7.652843 | -1.508224 | 1.738014 |
| 6 | 7.961041 | -1.564424 | 3.099463 |
| 6 | 6.943978 | -1.527855 | 4.055632 |
| 6 | 5.611001 | -1.434164 | 3.646615 |
| 15 | 5.794338 | -1.284429 | -0.445631 |
| 6 | 7.310825 | -0.880663 | -1.415890 |
| 6 | 8.367299 | -1.799110 | -1.551486 |
| 6 | 9.498964 | -1.468878 | -2.295520 |
| 6 | 9.585348 | -0.224339 | -2.927067 |
| 6 | 8.531702 | 0.683124 | -2.818246 |
| 6 | 7.399239 | 0.353812 | -2.070920 |
| 46 | 3.927162 | 0.135147 | -0.636253 |
| 6 | 2.394501 | -1.371107 | -1.039453 |
| 6 | 1.695984 | -0.134734 | -0.961634 |
| 15 | 4.636925 | 2.350547 | -0.076060 |
| 6 | 5.548741 | -3.043644 | -0.950561 |
| 6 | 5.136312 | -3.280920 | -2.272864 |
| 6 | 4.892991 | -4.579558 | -2.715474 |
| 6 | 5.038353 | -5.659120 | -1.837572 |
| 6 | 5.445468 | -5.431006 | -0.523613 |

| | | | |
|---|---|---|---|
| 6 | 5.706539 | -4.130817 | -0.081162 |
| 6 | 6.430891 | 2.607247 | 0.301983 |
| 6 | 6.972273 | 1.917128 | 1.399520 |
| 6 | 8.331485 | 2.006496 | 1.693752 |
| 6 | 9.174370 | 2.783738 | 0.894828 |
| 6 | 8.644615 | 3.478553 | -0.192534 |
| 6 | 7.281141 | 3.394678 | -0.486998 |
| 6 | 4.356494 | 3.608264 | -1.395992 |
| 6 | 4.345618 | 3.155202 | -2.724437 |
| 6 | 4.255071 | 4.060165 | -3.784232 |
| 6 | 4.162202 | 5.429735 | -3.526404 |
| 6 | 4.152980 | 5.889180 | -2.206328 |
| 6 | 4.252779 | 4.985603 | -1.146974 |
| 1 | 2.638904 | -1.726538 | -2.039662 |
| 1 | 4.997220 | -2.443737 | -2.952595 |
| 1 | 6.017941 | -3.966152 | 0.945253 |
| 1 | 4.574755 | -4.749089 | -3.740379 |
| 1 | 5.547949 | -6.263073 | 0.166153 |
| 1 | 4.825393 | -6.669131 | -2.174692 |
| 1 | 8.298688 | -2.777670 | -1.085631 |
| 1 | 6.580271 | 1.057810 | -1.996277 |
| 1 | 10.309876 | -2.185742 | -2.389517 |
| 1 | 8.586337 | 1.648779 | -3.312264 |
| 1 | 10.467076 | 0.030404 | -3.508367 |
| 1 | 8.456706 | -1.514118 | 1.010614 |
| 1 | 4.264598 | -1.268775 | 1.982756 |
| 1 | 8.999878 | -1.629643 | 3.411199 |
| 1 | 4.810945 | -1.394869 | 4.380169 |
| 1 | 7.188187 | -1.564769 | 5.113450 |
| 1 | 6.331214 | 1.306461 | 2.026796 |
| 1 | 6.888091 | 3.941550 | -1.337438 |
| 1 | 8.729305 | 1.458905 | 2.542776 |
| 1 | 9.290473 | 4.089659 | -0.817134 |
| 1 | 10.235204 | 2.847421 | 1.120030 |
| 1 | 4.406378 | 2.088366 | -2.924673 |
| 1 | 4.249207 | 5.355032 | -0.126938 |
| 1 | 4.246547 | 3.694811 | -4.807139 |
| 1 | 4.064035 | 6.951870 | -2.000301 |
| 1 | 4.082582 | 6.135084 | -4.348391 |
| 1 | 1.124837 | 0.109814 | -0.074663 |
| 6 | 1.508346 | 0.692677 | -2.105080 |
| 6 | 2.306275 | -2.471292 | 0.003296 |
| 6 | 1.048951 | -3.433192 | -0.044985 |
| 1 | 3.193951 | -3.089334 | -0.119945 |
| 1 | 2.349118 | -2.033227 | 1.003515 |
| 6 | 0.038700 | -2.988218 | 1.057111 |
| 1 | 0.486212 | -3.253467 | 2.014981 |
| 1 | -0.893949 | -3.550116 | 0.965546 |
| 6 | 1.442244 | -4.864377 | 0.380464 |
| 6 | 2.420712 | -5.079170 | 1.364491 |
| 6 | 0.753795 | -5.982049 | -0.113931 |
| 6 | 2.723569 | -6.365535 | 1.813103 |
| 1 | 2.955940 | -4.237819 | 1.792188 |
| 6 | 1.053881 | -7.270690 | 0.333098 |
| 1 | -0.025028 | -5.843196 | -0.855338 |
| 6 | 2.045280 | -7.470355 | 1.294995 |
| 1 | 3.491015 | -6.501299 | 2.570685 |
| 1 | 0.507974 | -8.118511 | -0.072460 |
| 1 | 2.281488 | -8.472632 | 1.641645 |
| 6 | 0.461781 | -3.438997 | -1.460271 |
| 6 | 1.268538 | -3.897262 | -2.518147 |
| 6 | -0.822817 | -2.979642 | -1.768088 |

| | | | |
|---|---|---|---|
| 6 | 0.823421 | -3.864928 | -3.837473 |
| 1 | 2.260617 | -4.277930 | -2.296676 |
| 6 | -1.273292 | -2.938445 | -3.095910 |
| 1 | -1.503092 | -2.635324 | -1.002106 |
| 6 | -0.451763 | -3.372376 | -4.135948 |
| 1 | 1.472460 | -4.220883 | -4.633492 |
| 1 | -2.273171 | -2.569767 | -3.300089 |
| 1 | -0.801411 | -3.334535 | -5.163703 |
| 7 | -0.214016 | -1.551638 | 1.057521 |
| 1 | -1.004019 | -1.214570 | 0.474778 |
| 16 | -0.162048 | -0.667381 | 2.448863 |
| 8 | 0.898476 | -1.220678 | 3.311553 |
| 1 | 2.126095 | 0.534143 | -2.982441 |
| 1 | 1.162707 | 1.710857 | -1.962860 |
| 8 | -0.065116 | 0.738812 | 2.020096 |
| 8 | -0.042517 | 0.136463 | -3.052880 |
| 1 | -0.911477 | 0.527033 | -2.665092 |
| 6 | 3.856344 | 2.999163 | 1.461872 |
| 6 | 4.421166 | 4.054176 | 2.202245 |
| 6 | 2.692484 | 2.379324 | 1.939142 |
| 6 | 3.820646 | 4.485953 | 3.384096 |
| 1 | 5.342039 | 4.522777 | 1.868661 |
| 6 | 2.103326 | 2.801697 | 3.132933 |
| 1 | 2.236500 | 1.562915 | 1.391748 |
| 6 | 2.663285 | 3.856390 | 3.854186 |
| 1 | 4.264643 | 5.303354 | 3.945668 |
| 1 | 1.207129 | 2.298783 | 3.475739 |
| 1 | 2.205159 | 4.184725 | 4.783249 |
| 1 | -0.112093 | -0.818351 | -2.877040 |
| 6 | -1.786061 | -0.856934 | 3.229440 |
| 6 | -2.145088 | -1.995835 | 4.023201 |
| 6 | -2.724350 | 0.078787 | 2.844407 |
| 6 | -1.246549 | -3.014884 | 4.448594 |
| 6 | -3.529816 | -2.117672 | 4.399709 |
| 6 | -4.074322 | -0.053406 | 3.235651 |
| 1 | -2.437474 | 0.898907 | 2.202141 |
| 6 | -1.694154 | -4.098457 | 5.173504 |
| 1 | -0.196084 | -2.926640 | 4.208942 |
| 6 | -3.950423 | -3.246399 | 5.153758 |
| 6 | -4.468277 | -1.131732 | 3.990545 |
| 1 | -4.796376 | 0.682318 | 2.898980 |
| 6 | -3.057075 | -4.223136 | 5.529249 |
| 1 | -0.986892 | -4.864696 | 5.477814 |
| 1 | -5.001280 | -3.326325 | 5.419283 |
| 1 | -5.510868 | -1.258207 | 4.269957 |
| 1 | -3.393231 | -5.085354 | 6.097664 |
| 6 | -4.169091 | 2.617499 | -0.129778 |
| 6 | -3.667355 | 3.894001 | -0.290733 |
| 6 | -4.582346 | 4.976696 | -0.483838 |
| 6 | -4.141663 | 6.312705 | -0.692820 |
| 1 | -3.076069 | 6.513813 | -0.709485 |
| 6 | -5.047188 | 7.335100 | -0.874961 |
| 1 | -4.691581 | 8.349040 | -1.035075 |
| 6 | -6.439530 | 7.075506 | -0.858981 |
| 1 | -7.142203 | 7.891075 | -1.004236 |
| 6 | -6.898647 | 5.792789 | -0.662213 |
| 1 | -7.965502 | 5.583681 | -0.651077 |
| 6 | -5.991941 | 4.714437 | -0.470330 |
| 6 | -6.448562 | 3.388250 | -0.266815 |
| 1 | -7.517811 | 3.195249 | -0.264122 |
| 6 | -5.569550 | 2.335896 | -0.095951 |
| 6 | -6.060583 | 0.958592 | 0.155388 |

| | | | |
|---|---|---|---|
| 6 | -7.081674 | 0.712011 | 1.052532 |
| 1 | -7.513643 | 1.537503 | 1.611651 |
| 6 | -7.568807 | -0.599760 | 1.278761 |
| 6 | -8.611849 | -0.849025 | 2.212703 |
| 1 | -9.033916 | -0.007445 | 2.756497 |
| 6 | -9.082398 | -2.125286 | 2.423428 |
| 1 | -9.879813 | -2.303354 | 3.139291 |
| 6 | -8.530545 | -3.209422 | 1.698732 |
| 1 | -8.910963 | -4.213873 | 1.861651 |
| 6 | -7.516345 | -3.002448 | 0.789536 |
| 1 | -7.099755 | -3.839539 | 0.241416 |
| 6 | -6.994867 | -1.699614 | 0.557993 |
| 6 | -5.922576 | -1.453181 | -0.361828 |
| 6 | -5.492554 | -0.151408 | -0.538559 |
| 6 | -2.189002 | 4.121491 | -0.226115 |
| 6 | -1.438803 | 4.155022 | -1.445180 |
| 6 | -2.042914 | 3.967983 | -2.719875 |
| 1 | -3.109759 | 3.786475 | -2.772839 |
| 6 | -1.289488 | 3.979585 | -3.871931 |
| 1 | -1.771577 | 3.820434 | -4.832320 |
| 6 | 0.111949 | 4.175189 | -3.812814 |
| 1 | 0.699166 | 4.175952 | -4.727281 |
| 6 | 0.729534 | 4.362782 | -2.596972 |
| 1 | 1.800655 | 4.514170 | -2.538978 |
| 6 | -0.022282 | 4.360606 | -1.390677 |
| 6 | 0.592904 | 4.544254 | -0.126902 |
| 1 | 1.666631 | 4.698807 | -0.081038 |
| 6 | -0.152616 | 4.509028 | 1.025876 |
| 1 | 0.349323 | 4.636143 | 1.977284 |
| 6 | -1.554582 | 4.277951 | 1.004598 |
| 6 | -5.299490 | -2.568680 | -1.144404 |
| 6 | -4.461694 | -3.517757 | -0.462937 |
| 6 | -4.137858 | -3.401424 | 0.918553 |
| 1 | -4.502329 | -2.549031 | 1.478263 |
| 6 | -3.354032 | -4.344037 | 1.547189 |
| 1 | -3.122289 | -4.235441 | 2.600522 |
| 6 | -2.827935 | -5.444054 | 0.826492 |
| 1 | -2.192017 | -6.165816 | 1.330699 |
| 6 | -3.099158 | -5.573917 | -0.516234 |
| 1 | -2.687695 | -6.402708 | -1.087170 |
| 6 | -3.919035 | -4.627750 | -1.188994 |
| 6 | -4.200697 | -4.743565 | -2.573869 |
| 1 | -3.779021 | -5.577620 | -3.129152 |
| 6 | -4.979959 | -3.808053 | -3.206165 |
| 1 | -5.175045 | -3.908652 | -4.269722 |
| 6 | -5.547239 | -2.704561 | -2.508546 |
| 8 | -2.271956 | 0.958052 | -2.189729 |
| 8 | -2.460068 | -0.761281 | -0.206320 |
| 8 | -3.283690 | 1.591402 | 0.136266 |
| 8 | -4.530526 | 0.117578 | -1.493071 |
| 15 | -2.985343 | 0.406711 | -0.983536 |
| 6 | -6.393939 | -1.718416 | -3.293271 |
| 6 | -5.563657 | -0.982957 | -4.370970 |
| 6 | -7.640602 | -2.376133 | -3.925742 |
| 1 | -6.762037 | -0.954502 | -2.602294 |
| 6 | -6.419311 | 0.050955 | -5.115954 |
| 1 | -5.170889 | -1.713994 | -5.091940 |
| 1 | -4.704574 | -0.499585 | -3.897343 |
| 6 | -8.492507 | -1.341707 | -4.677320 |
| 1 | -7.329200 | -3.163408 | -4.625854 |
| 1 | -8.233838 | -2.866710 | -3.144411 |
| 6 | -7.667234 | -0.594406 | -5.735689 |

| | | | |
|---|---|---|---|
| 1 | -5.820658 | 0.547455 | -5.889425 |
| 1 | -6.727550 | 0.833656 | -4.408290 |
| 1 | -9.356513 | -1.832565 | -5.142461 |
| 1 | -8.894721 | -0.616334 | -3.955643 |
| 1 | -8.284607 | 0.163798 | -6.233271 |
| 1 | -7.354019 | -1.305546 | -6.514213 |
| 6 | -2.334952 | 4.217617 | 2.309053 |
| 6 | -1.577284 | 3.543007 | 3.470259 |
| 6 | -2.829733 | 5.614195 | 2.752666 |
| 1 | -3.230375 | 3.611910 | 2.127373 |
| 6 | -2.477504 | 3.416535 | 4.707280 |
| 1 | -0.699036 | 4.144508 | 3.740936 |
| 1 | -1.202454 | 2.565580 | 3.156203 |
| 6 | -3.720661 | 5.513859 | 3.999673 |
| 1 | -1.956312 | 6.247605 | 2.965308 |
| 1 | -3.377401 | 6.093879 | 1.935293 |
| 6 | -3.009646 | 4.787647 | 5.151519 |
| 1 | -1.925563 | 2.937638 | 5.525482 |
| 1 | -3.321798 | 2.752231 | 4.474268 |
| 1 | -4.039836 | 6.514217 | 4.317825 |
| 1 | -4.634779 | 4.963029 | 3.734667 |
| 1 | -3.686371 | 4.678020 | 6.008206 |
| 1 | -2.165416 | 5.403405 | 5.494714 |

---

(*S*,*R*,*S*)-**[1a-2a]**‡exo-*si*

---

Number of imaginary frequencies : 1
The smallest frequencies are : -208.2331 9.7734 11.8844 cm(-1)

Electronic energy : HF=-6614.0048982
Zero-point correction= 1.888248 (Hartree/Particle)
Thermal correction to Energy= 1.998960
Thermal correction to Enthalpy= 1.999904
Thermal correction to Gibbs Free Energy= 1.732415
Sum of electronic and zero-point Energies= -6612.116650
Sum of electronic and thermal Energies= -6612.005938
Sum of electronic and thermal Enthalpies= -6612.004994
Sum of electronic and thermal Free Energies= -6612.272483

.............................................................

Cartesian Coordinates

.............................................................

| | | | |
|---|---|---|---|
| 6 | 5.628876 | 3.175777 | -0.282855 |
| 6 | 5.109710 | 2.578293 | -1.445948 |
| 6 | 4.815017 | 3.385292 | -2.552855 |
| 6 | 5.071039 | 4.758775 | -2.510199 |
| 6 | 5.625281 | 5.337844 | -1.367249 |
| 6 | 5.894781 | 4.544299 | -0.248280 |
| 15 | 4.892773 | 0.747735 | -1.423479 |
| 6 | 4.032958 | 0.295827 | -2.985192 |
| 6 | 4.666720 | 0.430975 | -4.235349 |
| 6 | 4.007435 | 0.050874 | -5.401888 |
| 6 | 2.712703 | -0.476130 | -5.334895 |
| 6 | 2.080407 | -0.614617 | -4.101116 |
| 6 | 2.737990 | -0.232412 | -2.929177 |
| 46 | 3.827595 | 0.059588 | 0.573907 |
| 6 | 1.971020 | 1.245153 | 0.497253 |
| 6 | 2.100233 | 0.641186 | 1.777342 |
| 15 | 5.340804 | -1.354152 | 1.752958 |
| 6 | 6.605646 | 0.155443 | -1.811193 |
| 6 | 6.768295 | -1.204277 | -2.127911 |
| 6 | 8.032911 | -1.732499 | -2.378911 |

| | | | |
|---|---|---|---|
| 6 | 9.160257 | -0.906242 | -2.328581 |
| 6 | 9.006714 | 0.449256 | -2.036620 |
| 6 | 7.739205 | 0.978304 | -1.776496 |
| 6 | 6.921902 | -0.510600 | 2.217857 |
| 6 | 7.974801 | -0.453256 | 1.289705 |
| 6 | 9.132489 | 0.271901 | 1.572924 |
| 6 | 9.257761 | 0.958796 | 2.781472 |
| 6 | 8.216841 | 0.907745 | 3.710580 |
| 6 | 7.059081 | 0.179101 | 3.433399 |
| 6 | 4.713240 | -2.006107 | 3.367391 |
| 6 | 4.057029 | -1.112239 | 4.232755 |
| 6 | 3.529439 | -1.548694 | 5.447385 |
| 6 | 3.632957 | -2.893459 | 5.811352 |
| 6 | 4.273551 | -3.791529 | 4.956506 |
| 6 | 4.813076 | -3.353247 | 3.744092 |
| 1 | 1.331727 | 0.720948 | -0.203818 |
| 1 | 5.902595 | -1.854337 | -2.179801 |
| 1 | 7.644497 | 2.032932 | -1.544786 |
| 1 | 8.135971 | -2.788974 | -2.608433 |
| 1 | 9.874102 | 1.103048 | -2.008027 |
| 1 | 10.147359 | -1.316104 | -2.522651 |
| 1 | 5.677729 | 0.823030 | -4.292175 |
| 1 | 2.228918 | -0.342295 | -1.979562 |
| 1 | 4.503870 | 0.161469 | -6.362082 |
| 1 | 1.070993 | -0.999636 | -4.029863 |
| 1 | 2.199210 | -0.772815 | -6.245437 |
| 1 | 4.385546 | 2.948377 | -3.447870 |
| 1 | 5.835408 | 2.563221 | 0.591622 |
| 1 | 4.835598 | 5.374152 | -3.373772 |
| 1 | 6.304313 | 4.991659 | 0.652825 |
| 1 | 5.831576 | 6.403840 | -1.340673 |
| 1 | 7.899973 | -0.980781 | 0.347036 |
| 1 | 6.273034 | 0.142017 | 4.178979 |
| 1 | 9.931795 | 0.298821 | 0.838684 |
| 1 | 8.305318 | 1.429442 | 4.659556 |
| 1 | 10.158984 | 1.524643 | 2.999858 |
| 1 | 3.937251 | -0.072442 | 3.942296 |
| 1 | 5.300349 | -4.070030 | 3.092731 |
| 1 | 3.020918 | -0.842673 | 6.097372 |
| 1 | 4.353452 | -4.840368 | 5.228407 |
| 1 | 3.208751 | -3.239120 | 6.749427 |
| 1 | 2.467709 | 1.217128 | 2.622882 |
| 6 | 1.601596 | -0.659970 | 2.030089 |
| 6 | 2.097650 | 2.734993 | 0.263917 |
| 6 | 0.832653 | 3.613471 | 0.588042 |
| 1 | 2.938239 | 3.122034 | 0.846816 |
| 1 | 2.350950 | 2.893110 | -0.787089 |
| 6 | -0.247966 | 3.419376 | -0.521331 |
| 1 | 0.162514 | 3.852779 | -1.434106 |
| 1 | -1.147031 | 3.986903 | -0.262987 |
| 6 | 1.167157 | 5.118741 | 0.504973 |
| 6 | 2.287912 | 5.609428 | -0.176255 |
| 6 | 0.265238 | 6.049562 | 1.049689 |
| 6 | 2.510027 | 6.984136 | -0.295095 |
| 1 | 3.002507 | 4.931670 | -0.623983 |
| 6 | 0.480765 | 7.421194 | 0.925083 |
| 1 | -0.613216 | 5.691729 | 1.577740 |
| 6 | 1.609915 | 7.897106 | 0.253177 |
| 1 | 3.393290 | 7.333560 | -0.822684 |
| 1 | -0.232351 | 8.118396 | 1.357148 |
| 1 | 1.783637 | 8.965586 | 0.159301 |
| 6 | 0.332636 | 3.306500 | 2.007015 |

| | | | |
|---|---|---|---|
| 6 | 1.200537 | 3.538339 | 3.089899 |
| 6 | -0.951141 | 2.827427 | 2.290842 |
| 6 | 0.816778 | 3.265627 | 4.401052 |
| 1 | 2.189385 | 3.946704 | 2.899825 |
| 6 | -1.341307 | 2.547300 | 3.608292 |
| 1 | -1.678849 | 2.654031 | 1.511817 |
| 6 | -0.458418 | 2.756608 | 4.666907 |
| 1 | 1.511463 | 3.452873 | 5.215694 |
| 1 | -2.341138 | 2.166067 | 3.788991 |
| 1 | -0.760529 | 2.532559 | 5.685900 |
| 7 | -0.579371 | 2.030684 | -0.797158 |
| 1 | -1.238681 | 1.514588 | -0.188106 |
| 16 | -0.539347 | 1.369271 | -2.299716 |
| 8 | 0.400501 | 2.151196 | -3.122235 |
| 1 | 1.130758 | -1.213222 | 1.223024 |
| 1 | 1.986830 | -1.251537 | 2.845661 |
| 8 | -0.297629 | -0.069099 | -2.098366 |
| 8 | -0.060062 | -0.409490 | 3.108827 |
| 1 | -0.835944 | -0.923037 | 2.696744 |
| 6 | 5.875270 | -2.867820 | 0.858091 |
| 6 | 7.045551 | -3.580463 | 1.162963 |
| 6 | 5.035135 | -3.340419 | -0.160059 |
| 6 | 7.365620 | -4.740753 | 0.456697 |
| 1 | 7.709509 | -3.225412 | 1.944935 |
| 6 | 5.347888 | -4.508476 | -0.857086 |
| 1 | 4.141409 | -2.776621 | -0.411160 |
| 6 | 6.516893 | -5.208442 | -0.551040 |
| 1 | 8.276635 | -5.282077 | 0.695635 |
| 1 | 4.684363 | -4.864849 | -1.639779 |
| 1 | 6.767638 | -6.114010 | -1.096046 |
| 1 | -0.293873 | 0.522581 | 2.961660 |
| 6 | -2.214720 | 1.514282 | -2.969747 |
| 6 | -2.725737 | 2.731858 | -3.528371 |
| 6 | -3.027801 | 0.419895 | -2.761270 |
| 6 | -1.961034 | 3.910522 | -3.759099 |
| 6 | -4.128220 | 2.763649 | -3.852357 |
| 6 | -4.395500 | 0.464460 | -3.109994 |
| 1 | -2.625566 | -0.465644 | -2.289608 |
| 6 | -2.552968 | 5.055519 | -4.247810 |
| 1 | -0.898596 | 3.899764 | -3.559925 |
| 6 | -4.699771 | 3.961788 | -4.359180 |
| 6 | -4.934745 | 1.613631 | -3.635502 |
| 1 | -5.017392 | -0.402848 | -2.919968 |
| 6 | -3.934571 | 5.089527 | -4.547266 |
| 1 | -1.946411 | 5.942446 | -4.407513 |
| 1 | -5.762459 | 3.969967 | -4.586869 |
| 1 | -5.993777 | 1.667164 | -3.873159 |
| 1 | -4.385457 | 6.001602 | -4.927306 |
| 6 | -3.645674 | -2.835932 | -0.376198 |
| 6 | -2.917997 | -3.999939 | -0.533125 |
| 6 | -3.616548 | -5.248412 | -0.569782 |
| 6 | -2.937701 | -6.493841 | -0.674997 |
| 1 | -1.854331 | -6.494858 | -0.725151 |
| 6 | -3.637480 | -7.680378 | -0.706172 |
| 1 | -3.100708 | -8.621755 | -0.782670 |
| 6 | -5.052064 | -7.684235 | -0.635864 |
| 1 | -5.591244 | -8.626871 | -0.661938 |
| 6 | -5.739300 | -6.496004 | -0.533093 |
| 1 | -6.825037 | -6.489329 | -0.476811 |
| 6 | -5.048931 | -5.253532 | -0.496317 |
| 6 | -5.744124 | -4.022222 | -0.395842 |
| 1 | -6.829630 | -4.033672 | -0.348862 |

| | | | |
|---|---|---|---|
| 6 | -5.075048 | -2.815327 | -0.333501 |
| 6 | -5.807904 | -1.524786 | -0.286092 |
| 6 | -6.885036 | -1.275136 | -1.114105 |
| 1 | -7.199397 | -2.032943 | -1.826663 |
| 6 | -7.572057 | -0.034748 | -1.082583 |
| 6 | -8.663813 | 0.226696 | -1.955284 |
| 1 | -8.972252 | -0.551743 | -2.648853 |
| 6 | -9.319020 | 1.437274 | -1.926033 |
| 1 | -10.150920 | 1.625407 | -2.598776 |
| 6 | -8.912120 | 2.438660 | -1.011250 |
| 1 | -9.437286 | 3.389329 | -0.986718 |
| 6 | -7.857298 | 2.218142 | -0.152815 |
| 1 | -7.551193 | 2.991176 | 0.542845 |
| 6 | -7.147889 | 0.985786 | -0.166839 |
| 6 | -6.031384 | 0.733057 | 0.694892 |
| 6 | -5.405018 | -0.496686 | 0.617616 |
| 6 | -1.434717 | -3.931712 | -0.743381 |
| 6 | -0.550080 | -4.093076 | 0.372363 |
| 6 | -1.025505 | -4.288166 | 1.699066 |
| 1 | -2.093815 | -4.295065 | 1.875990 |
| 6 | -0.151677 | -4.420371 | 2.754858 |
| 1 | -0.539561 | -4.548115 | 3.761390 |
| 6 | 1.247500 | -4.369169 | 2.543694 |
| 1 | 1.928489 | -4.459704 | 3.385037 |
| 6 | 1.744019 | -4.192766 | 1.271703 |
| 1 | 2.815534 | -4.160450 | 1.100628 |
| 6 | 0.867526 | -4.047861 | 0.160544 |
| 6 | 1.349168 | -3.850055 | -1.159059 |
| 1 | 2.420388 | -3.825575 | -1.333456 |
| 6 | 0.478610 | -3.689715 | -2.209332 |
| 1 | 0.878948 | -3.527186 | -3.204435 |
| 6 | -0.930314 | -3.718373 | -2.025441 |
| 6 | -5.559464 | 1.748035 | 1.691621 |
| 6 | -4.857583 | 2.914181 | 1.229466 |
| 6 | -4.547318 | 3.130088 | -0.143180 |
| 1 | -4.821458 | 2.378542 | -0.873451 |
| 6 | -3.894691 | 4.271975 | -0.553534 |
| 1 | -3.674465 | 4.418472 | -1.604288 |
| 6 | -3.492424 | 5.252844 | 0.385699 |
| 1 | -2.964724 | 6.139722 | 0.046387 |
| 6 | -3.753271 | 5.065613 | 1.724007 |
| 1 | -3.436869 | 5.801064 | 2.459682 |
| 6 | -4.440448 | 3.906614 | 2.176084 |
| 6 | -4.710928 | 3.697152 | 3.552441 |
| 1 | -4.386156 | 4.444335 | 4.272318 |
| 6 | -5.360534 | 2.562679 | 3.968938 |
| 1 | -5.550975 | 2.416297 | 5.027909 |
| 6 | -5.798057 | 1.566162 | 3.051778 |
| 8 | -2.088529 | -1.544603 | 2.070094 |
| 8 | -2.427502 | 0.623444 | 0.619927 |
| 8 | -2.973633 | -1.630141 | -0.385933 |
| 8 | -4.407673 | -0.798508 | 1.518790 |
| 15 | -2.831149 | -0.760794 | 1.021458 |
| 6 | -6.505516 | 0.342543 | 3.606275 |
| 6 | -5.589700 | -0.478536 | 4.542962 |
| 6 | -7.830167 | 0.697686 | 4.318263 |
| 1 | -6.768566 | -0.311811 | 2.770147 |
| 6 | -6.304840 | -1.739637 | 5.047962 |
| 1 | -5.297857 | 0.139177 | 5.404112 |
| 1 | -4.672833 | -0.744915 | 4.009468 |
| 6 | -8.541382 | -0.565263 | 4.827626 |
| 1 | -7.627845 | 1.365659 | 5.167071 |

| | | | |
|---|---|---|---|
| 1 | -8.480083 | 1.251938 | 3.630098 |
| 6 | -7.629719 | -1.394958 | 5.743869 |
| 1 | -5.648727 | -2.296113 | 5.728453 |
| 1 | -6.503479 | -2.402893 | 4.194041 |
| 1 | -9.464753 | -0.291275 | 5.353089 |
| 1 | -8.840972 | -1.179070 | 3.966038 |
| 1 | -8.144019 | -2.309383 | 6.064760 |
| 1 | -7.416236 | -0.817534 | 6.655444 |
| 6 | -1.853162 | -3.558338 | -3.224131 |
| 6 | -1.389770 | -2.509827 | -4.255541 |
| 6 | -2.105775 | -4.908414 | -3.935169 |
| 1 | -2.823133 | -3.215343 | -2.847468 |
| 6 | -2.443588 | -2.333054 | -5.357931 |
| 1 | -0.450934 | -2.836427 | -4.723745 |
| 1 | -1.183145 | -1.562063 | -3.753423 |
| 6 | -3.144503 | -4.755071 | -5.055571 |
| 1 | -1.154923 | -5.271272 | -4.351756 |
| 1 | -2.440293 | -5.657290 | -3.210538 |
| 6 | -2.741738 | -3.665700 | -6.061844 |
| 1 | -2.105489 | -1.583698 | -6.084095 |
| 1 | -3.367564 | -1.937425 | -4.912828 |
| 1 | -3.292045 | -5.713517 | -5.568734 |
| 1 | -4.110839 | -4.489668 | -4.602964 |
| 1 | -3.527657 | -3.534444 | -6.816200 |
| 1 | -1.840581 | -3.992579 | -6.601116 |

---

(*S*,*R*,*S*)-**[1a-2a]**$^{\ddagger}$exo-*re*

---

Number of imaginary frequencies : 1
The smallest frequencies are : -341.6365 12.2491 14.0794 cm(-1)

Electronic energy : HF=-6613.9921508
Zero-point correction= 1.888993 (Hartree/Particle)
Thermal correction to Energy= 1.999222
Thermal correction to Enthalpy= 2.000166
Thermal correction to Gibbs Free Energy= 1.736301
Sum of electronic and zero-point Energies= -6612.103157
Sum of electronic and thermal Energies= -6611.992929
Sum of electronic and thermal Enthalpies= -6611.991984
Sum of electronic and thermal Free Energies= -6612.255850

............................................................
Cartesian Coordinates
............................................................

| | | | |
|---|---|---|---|
| 6 | 5.100120 | 3.227021 | 0.144953 |
| 6 | 5.476569 | 1.894608 | -0.084127 |
| 6 | 6.212057 | 1.589446 | -1.243500 |
| 6 | 6.571919 | 2.595057 | -2.138581 |
| 6 | 6.178978 | 3.917233 | -1.909400 |
| 6 | 5.440448 | 4.228204 | -0.768290 |
| 15 | 4.848812 | 0.519705 | 0.976195 |
| 6 | 6.350276 | -0.443331 | 1.444723 |
| 6 | 7.664455 | 0.024417 | 1.305795 |
| 6 | 8.743294 | -0.789271 | 1.660093 |
| 6 | 8.522737 | -2.075394 | 2.158324 |
| 6 | 7.215114 | -2.545084 | 2.311890 |
| 6 | 6.138389 | -1.733742 | 1.955553 |
| 46 | 3.215641 | -0.673138 | -0.256772 |
| 6 | 1.293767 | 0.358690 | 0.114042 |
| 6 | 1.118618 | -0.568205 | -0.938755 |
| 15 | 4.366921 | -2.360480 | -1.487231 |
| 6 | 4.456647 | 1.415236 | 2.547061 |

| | | | |
|---|---|---|---|
| 6 | 3.123779 | 1.566751 | 2.947589 |
| 6 | 2.796697 | 2.340350 | 4.064206 |
| 6 | 3.805626 | 2.955181 | 4.804967 |
| 6 | 5.143129 | 2.788238 | 4.431306 |
| 6 | 5.467012 | 2.027986 | 3.308138 |
| 6 | 5.980416 | -1.892972 | -2.258593 |
| 6 | 6.021390 | -1.305339 | -3.533462 |
| 6 | 7.220410 | -0.811178 | -4.049571 |
| 6 | 8.398726 | -0.897770 | -3.304229 |
| 6 | 8.368382 | -1.484300 | -2.037495 |
| 6 | 7.170342 | -1.972966 | -1.514632 |
| 6 | 4.749762 | -3.891984 | -0.537645 |
| 6 | 3.995710 | -4.142739 | 0.619185 |
| 6 | 4.232826 | -5.281822 | 1.390026 |
| 6 | 5.228710 | -6.186078 | 1.012929 |
| 6 | 5.979413 | -5.949883 | -0.141349 |
| 6 | 5.740023 | -4.812680 | -0.914708 |
| 1 | 0.997320 | -0.008460 | 1.096836 |
| 1 | 2.337419 | 1.076487 | 2.392710 |
| 1 | 6.507708 | 1.917700 | 3.018426 |
| 1 | 1.756823 | 2.466076 | 4.345693 |
| 1 | 5.934146 | 3.257420 | 5.009865 |
| 1 | 3.551174 | 3.559842 | 5.670955 |
| 1 | 7.849859 | 1.014310 | 0.901866 |
| 1 | 5.126049 | -2.113236 | 2.048238 |
| 1 | 9.757389 | -0.417884 | 1.540646 |
| 1 | 7.032043 | -3.547602 | 2.687415 |
| 1 | 9.364517 | -2.708898 | 2.423184 |
| 1 | 6.505477 | 0.567721 | -1.447921 |
| 1 | 4.522771 | 3.489945 | 1.023291 |
| 1 | 7.147600 | 2.337437 | -3.022957 |
| 1 | 5.104119 | 5.244916 | -0.591276 |
| 1 | 6.439398 | 4.696100 | -2.620183 |
| 1 | 5.117766 | -1.225429 | -4.127485 |
| 1 | 7.166992 | -2.408195 | -0.522262 |
| 1 | 7.231573 | -0.360787 | -5.038284 |
| 1 | 9.274014 | -1.553324 | -1.442307 |
| 1 | 9.330714 | -0.511905 | -3.707082 |
| 1 | 3.236044 | -3.427620 | 0.920661 |
| 1 | 6.332776 | -4.636723 | -1.806685 |
| 1 | 3.650845 | -5.458589 | 2.290332 |
| 1 | 6.752846 | -6.651205 | -0.441290 |
| 1 | 5.419898 | -7.069180 | 1.615822 |
| 1 | 1.072884 | -0.244811 | -1.971035 |
| 6 | 0.866342 | -1.963743 | -0.678370 |
| 6 | 1.394096 | 1.886580 | 0.063258 |
| 6 | 0.870276 | 2.736172 | -1.126118 |
| 1 | 0.881412 | 2.258956 | 0.953932 |
| 1 | 2.439584 | 2.159729 | 0.198812 |
| 6 | -0.685605 | 2.739502 | -1.123218 |
| 1 | -1.053314 | 1.748428 | -1.394414 |
| 1 | -1.065040 | 3.455332 | -1.854343 |
| 6 | 1.425894 | 2.218289 | -2.465653 |
| 6 | 2.818267 | 2.094970 | -2.618449 |
| 6 | 0.614788 | 1.887111 | -3.560195 |
| 6 | 3.381253 | 1.655899 | -3.814579 |
| 1 | 3.473428 | 2.358834 | -1.797446 |
| 6 | 1.179374 | 1.453557 | -4.766043 |
| 1 | -0.464954 | 1.949951 | -3.492684 |
| 6 | 2.561795 | 1.335851 | -4.901734 |
| 1 | 4.460337 | 1.564607 | -3.889514 |
| 1 | 0.528132 | 1.212775 | -5.601540 |

| | | | |
|---|---|---|---|
| 1 | 2.996116 | 0.995920 | -5.837926 |
| 6 | 1.360365 | 4.197512 | -0.954933 |
| 6 | 1.205522 | 5.092844 | -2.027024 |
| 6 | 1.952612 | 4.685046 | 0.216585 |
| 6 | 1.645354 | 6.411024 | -1.944083 |
| 1 | 0.743750 | 4.745263 | -2.946283 |
| 6 | 2.410342 | 6.004146 | 0.300364 |
| 1 | 2.062139 | 4.053410 | 1.088806 |
| 6 | 2.263239 | 6.873703 | -0.777822 |
| 1 | 1.510925 | 7.076585 | -2.792427 |
| 1 | 2.863595 | 6.348962 | 1.225250 |
| 1 | 2.615799 | 7.899429 | -0.711575 |
| 7 | -1.243244 | 3.043895 | 0.196774 |
| 1 | -1.626452 | 2.233448 | 0.704035 |
| 16 | -2.028738 | 4.445988 | 0.572503 |
| 8 | -1.979533 | 5.342355 | -0.592881 |
| 1 | 0.954151 | -2.305043 | 0.347649 |
| 1 | 1.183490 | -2.674971 | -1.428669 |
| 8 | -3.317535 | 4.106016 | 1.196158 |
| 8 | -0.879694 | -2.402738 | -0.911498 |
| 1 | -1.382421 | -1.507581 | -1.097845 |
| 6 | 3.379170 | -2.973203 | -2.928350 |
| 6 | 2.675396 | -2.017199 | -3.681183 |
| 6 | 3.257322 | -4.327632 | -3.272154 |
| 6 | 1.886638 | -2.402360 | -4.764372 |
| 1 | 2.734533 | -0.967960 | -3.414026 |
| 6 | 2.451933 | -4.712619 | -4.347120 |
| 1 | 3.779926 | -5.087884 | -2.701448 |
| 6 | 1.768706 | -3.753786 | -5.097871 |
| 1 | 1.354611 | -1.643212 | -5.329361 |
| 1 | 2.360254 | -5.766128 | -4.596707 |
| 1 | 1.143926 | -4.058678 | -5.932714 |
| 1 | -1.240903 | -2.698857 | -0.057375 |
| 6 | -1.048998 | 5.190952 | 1.892276 |
| 6 | -0.860513 | 4.522567 | 3.146874 |
| 6 | -0.539283 | 6.445981 | 1.656745 |
| 6 | -1.382573 | 3.237028 | 3.467521 |
| 6 | -0.089832 | 5.211528 | 4.145739 |
| 6 | 0.208898 | 7.112105 | 2.655476 |
| 1 | -0.695946 | 6.908138 | 0.689377 |
| 6 | -1.159414 | 2.675744 | 4.707313 |
| 1 | -1.985142 | 2.688411 | 2.756478 |
| 6 | 0.122846 | 4.596702 | 5.409448 |
| 6 | 0.433522 | 6.504788 | 3.866752 |
| 1 | 0.609476 | 8.099706 | 2.448925 |
| 6 | -0.402446 | 3.355394 | 5.690525 |
| 1 | -1.583981 | 1.701719 | 4.931992 |
| 1 | 0.702772 | 5.133343 | 6.156194 |
| 1 | 1.011142 | 7.007841 | 4.638073 |
| 1 | -0.240874 | 2.897925 | 6.662433 |
| 6 | -5.406132 | -0.213587 | -0.408849 |
| 6 | -5.880562 | -0.122019 | -1.702820 |
| 6 | -7.117431 | -0.760874 | -2.028593 |
| 6 | -7.687620 | -0.683820 | -3.328004 |
| 1 | -7.160637 | -0.127276 | -4.095822 |
| 6 | -8.889822 | -1.296229 | -3.607792 |
| 1 | -9.314679 | -1.222444 | -4.604911 |
| 6 | -9.577355 | -2.024325 | -2.606129 |
| 1 | -10.523839 | -2.502778 | -2.841233 |
| 6 | -9.044413 | -2.129113 | -1.340852 |
| 1 | -9.561299 | -2.693417 | -0.568596 |
| 6 | -7.808549 | -1.505930 | -1.016807 |

| | | | |
|---|---|---|---|
| 6 | -7.212459 | -1.655931 | 0.261219 |
| 1 | -7.685439 | -2.313909 | 0.984837 |
| 6 | -6.015928 | -1.040885 | 0.582549 |
| 6 | -5.319748 | -1.382142 | 1.851173 |
| 6 | -5.986883 | -1.531823 | 3.052107 |
| 1 | -7.026934 | -1.226611 | 3.125595 |
| 6 | -5.355769 | -2.095964 | 4.190992 |
| 6 | -6.043725 | -2.236150 | 5.427203 |
| 1 | -7.059738 | -1.857137 | 5.502640 |
| 6 | -5.441880 | -2.842412 | 6.506965 |
| 1 | -5.978817 | -2.942270 | 7.445870 |
| 6 | -4.121556 | -3.342457 | 6.395730 |
| 1 | -3.655657 | -3.823401 | 7.251185 |
| 6 | -3.424261 | -3.222071 | 5.213385 |
| 1 | -2.411493 | -3.602697 | 5.133613 |
| 6 | -4.010972 | -2.587772 | 4.085127 |
| 6 | -3.313129 | -2.424048 | 2.848726 |
| 6 | -3.946727 | -1.775701 | 1.810544 |
| 6 | -5.025910 | 0.531938 | -2.744455 |
| 6 | -4.062638 | -0.297516 | -3.412469 |
| 6 | -3.946607 | -1.693233 | -3.160182 |
| 1 | -4.626082 | -2.161653 | -2.457477 |
| 6 | -2.966148 | -2.447714 | -3.763992 |
| 1 | -2.883098 | -3.506729 | -3.537371 |
| 6 | -2.053283 | -1.851397 | -4.666423 |
| 1 | -1.275628 | -2.455019 | -5.125547 |
| 6 | -2.151700 | -0.508062 | -4.950611 |
| 1 | -1.461565 | -0.040416 | -5.648058 |
| 6 | -3.145945 | 0.299238 | -4.335440 |
| 6 | -3.236750 | 1.694543 | -4.580903 |
| 1 | -2.541029 | 2.153038 | -5.280081 |
| 6 | -4.177707 | 2.459789 | -3.936982 |
| 1 | -4.216130 | 3.528432 | -4.124855 |
| 6 | -5.091399 | 1.894098 | -3.002956 |
| 6 | -1.991558 | -3.082056 | 2.587350 |
| 6 | -0.765896 | -2.469411 | 3.009132 |
| 6 | -0.727905 | -1.174491 | 3.598693 |
| 1 | -1.650093 | -0.611269 | 3.681551 |
| 6 | 0.466202 | -0.620648 | 4.006202 |
| 1 | 0.478303 | 0.375505 | 4.436084 |
| 6 | 1.682452 | -1.331242 | 3.856376 |
| 1 | 2.612392 | -0.882168 | 4.189149 |
| 6 | 1.684579 | -2.573609 | 3.264206 |
| 1 | 2.615125 | -3.119359 | 3.133444 |
| 6 | 0.473359 | -3.166110 | 2.813853 |
| 6 | 0.455746 | -4.415573 | 2.140479 |
| 1 | 1.392935 | -4.935805 | 1.965500 |
| 6 | -0.726190 | -4.951028 | 1.690310 |
| 1 | -0.712718 | -5.894540 | 1.153372 |
| 6 | -1.975889 | -4.308210 | 1.918704 |
| 8 | -2.091113 | -0.232897 | -1.099160 |
| 8 | -2.208882 | 0.681035 | 1.355359 |
| 8 | -4.327526 | 0.566604 | -0.035762 |
| 8 | -3.239963 | -1.615962 | 0.631587 |
| 15 | -2.832288 | -0.057799 | 0.208581 |
| 6 | -3.243083 | -4.961816 | 1.391816 |
| 6 | -3.303683 | -4.916089 | -0.153744 |
| 6 | -3.446647 | -6.405793 | 1.898030 |
| 1 | -4.098738 | -4.386640 | 1.755626 |
| 6 | -4.627052 | -5.495382 | -0.671554 |
| 1 | -2.461266 | -5.488934 | -0.566703 |
| 1 | -3.185324 | -3.884496 | -0.497124 |

| | | | |
|---|---|---|---|
| 6 | -4.775283 | -6.979674 | 1.381889 |
| 1 | -2.621599 | -7.045312 | 1.556235 |
| 1 | -3.423502 | -6.417324 | 2.994364 |
| 6 | -4.857409 | -6.921879 | -0.151162 |
| 1 | -4.640310 | -5.481079 | -1.768238 |
| 1 | -5.451080 | -4.846779 | -0.341330 |
| 1 | -4.900519 | -8.011546 | 1.732074 |
| 1 | -5.605269 | -6.399620 | 1.809883 |
| 1 | -5.827578 | -7.301654 | -0.494040 |
| 1 | -4.091246 | -7.586327 | -0.576644 |
| 6 | -6.041415 | 2.801966 | -2.246213 |
| 6 | -5.257022 | 3.661705 | -1.231519 |
| 6 | -6.916456 | 3.690719 | -3.156345 |
| 1 | -6.725869 | 2.167452 | -1.670227 |
| 6 | -6.185563 | 4.563064 | -0.411436 |
| 1 | -4.527760 | 4.284700 | -1.767056 |
| 1 | -4.682214 | 3.013597 | -0.568705 |
| 6 | -7.849373 | 4.583510 | -2.322281 |
| 1 | -6.276999 | 4.330089 | -3.779872 |
| 1 | -7.498627 | 3.062700 | -3.842602 |
| 6 | -7.061529 | 5.438792 | -1.317995 |
| 1 | -5.584689 | 5.174690 | 0.268828 |
| 1 | -6.832328 | 3.934700 | 0.218563 |
| 1 | -8.448869 | 5.220905 | -2.984583 |
| 1 | -8.557975 | 3.946203 | -1.773510 |
| 1 | -7.749280 | 6.049440 | -0.719212 |
| 1 | -6.418595 | 6.139408 | -1.870805 |

---

(*S*,*R*,*S*)-**[1a-2a]**$^{‡}$endo-*si*

---

Number of imaginary frequencies : 1
The smallest frequencies are : -298.8412 11.7187 15.6685 cm(-1)

Electronic energy : HF=-6613.9996265
Zero-point correction= 1.887562 (Hartree/Particle)
Thermal correction to Energy= 1.998352
Thermal correction to Enthalpy= 1.999297
Thermal correction to Gibbs Free Energy= 1.732915
Sum of electronic and zero-point Energies= -6612.112064
Sum of electronic and thermal Energies= -6612.001274
Sum of electronic and thermal Enthalpies= -6612.000330
Sum of electronic and thermal Free Energies= -6612.266711

......................................................

Cartesian Coordinates

......................................................

| | | | |
|---|---|---|---|
| 6 | -3.419009 | 5.124939 | -0.395887 |
| 6 | -3.870470 | 3.806428 | -0.218451 |
| 6 | -4.556056 | 3.477201 | 0.958202 |
| 6 | -4.808279 | 4.446698 | 1.930182 |
| 6 | -4.366195 | 5.757439 | 1.741648 |
| 6 | -3.668566 | 6.093587 | 0.577548 |
| 15 | -3.591674 | 2.501212 | -1.492155 |
| 6 | -4.861551 | 2.843103 | -2.786629 |
| 6 | -5.617225 | 4.021295 | -2.847895 |
| 6 | -6.585307 | 4.189110 | -3.841630 |
| 6 | -6.806209 | 3.185762 | -4.786788 |
| 6 | -6.053566 | 2.008690 | -4.736966 |
| 6 | -5.092601 | 1.837103 | -3.741185 |
| 46 | -3.557540 | 0.203617 | -0.873329 |
| 6 | -2.513546 | -1.696402 | -0.835645 |
| 6 | -1.580956 | -0.692451 | -1.208297 |

| | | | |
|---|---|---|---|
| 15 | -5.551462 | -0.385447 | 0.244131 |
| 6 | -6.540607 | -1.806824 | -0.401994 |
| 6 | -5.882974 | -2.841905 | -1.084343 |
| 6 | -6.591039 | -3.950532 | -1.548911 |
| 6 | -7.969831 | -4.036401 | -1.347324 |
| 6 | -8.636316 | -3.008142 | -0.677062 |
| 6 | -7.928743 | -1.900543 | -0.206511 |
| 6 | -2.022275 | 3.072815 | -2.270822 |
| 6 | -1.959071 | 3.692588 | -3.526742 |
| 6 | -0.723117 | 4.075397 | -4.057658 |
| 6 | 0.450336 | 3.856336 | -3.335047 |
| 6 | 0.391686 | 3.248697 | -2.077691 |
| 6 | -0.835434 | 2.850779 | -1.552608 |
| 6 | -5.157682 | -0.862122 | 1.983037 |
| 6 | -5.909989 | -1.795933 | 2.710419 |
| 6 | -5.556783 | -2.114189 | 4.024086 |
| 6 | -4.446182 | -1.512745 | 4.619710 |
| 6 | -3.685175 | -0.587943 | 3.899891 |
| 6 | -4.041518 | -0.261989 | 2.591736 |
| 6 | -6.847458 | 0.913962 | 0.443193 |
| 6 | -7.295191 | 1.567925 | -0.716048 |
| 6 | -8.231642 | 2.596728 | -0.632344 |
| 6 | -8.728177 | 2.992641 | 0.612632 |
| 6 | -8.294092 | 2.343626 | 1.769503 |
| 6 | -7.361397 | 1.306062 | 1.687698 |
| 1 | -2.918159 | -2.274371 | -1.666772 |
| 1 | -2.867353 | 3.878845 | -4.091100 |
| 1 | -0.867305 | 2.356110 | -0.586478 |
| 1 | -0.682955 | 4.548567 | -5.035326 |
| 1 | 1.295807 | 3.079272 | -1.508316 |
| 1 | 1.410863 | 4.152820 | -3.746001 |
| 1 | -5.463507 | 4.805548 | -2.114512 |
| 1 | -4.528707 | 0.909096 | -3.688506 |
| 1 | -7.168430 | 5.105383 | -3.872877 |
| 1 | -6.222827 | 1.220579 | -5.465316 |
| 1 | -7.562237 | 3.317076 | -5.555727 |
| 1 | -4.893076 | 2.461909 | 1.117970 |
| 1 | -2.864058 | 5.391965 | -1.289856 |
| 1 | -5.343277 | 4.172570 | 2.834644 |
| 1 | -3.313852 | 7.109733 | 0.428881 |
| 1 | -4.554906 | 6.511915 | 2.500030 |
| 1 | -6.765990 | -2.282175 | 2.253286 |
| 1 | -3.429122 | 0.435816 | 2.026913 |
| 1 | -6.142375 | -2.844615 | 4.575273 |
| 1 | -2.789436 | -0.161727 | 4.337192 |
| 1 | -4.157809 | -1.780318 | 5.631936 |
| 1 | -4.815920 | -2.785889 | -1.253515 |
| 1 | -8.459573 | -1.109074 | 0.312622 |
| 1 | -6.058367 | -4.740458 | -2.069978 |
| 1 | -9.709594 | -3.066008 | -0.518322 |
| 1 | -8.522951 | -4.897121 | -1.712636 |
| 1 | -6.899836 | 1.282449 | -1.685420 |
| 1 | -7.030077 | 0.810733 | 2.594430 |
| 1 | -8.557114 | 3.097934 | -1.538822 |
| 1 | -8.679107 | 2.643379 | 2.740332 |
| 1 | -9.447945 | 3.803563 | 0.679840 |
| 1 | -0.880732 | -0.277080 | -0.488721 |
| 6 | -1.353393 | -0.384281 | -2.580061 |
| 8 | 0.126022 | -1.436851 | -3.079416 |
| 6 | -2.481407 | -2.437998 | 0.477185 |
| 6 | -1.503793 | -3.657186 | 0.525835 |
| 1 | -2.221333 | -1.761934 | 1.292346 |

| | | | |
|---|---|---|---|
| 1 | -3.484997 | -2.817422 | 0.681470 |
| 6 | -0.030186 | -3.139624 | 0.624582 |
| 1 | 0.271386 | -2.667095 | -0.311409 |
| 1 | 0.640494 | -3.984374 | 0.807389 |
| 6 | -1.748183 | -4.480685 | -0.753763 |
| 6 | -2.929982 | -5.240606 | -0.838673 |
| 6 | -0.935662 | -4.410815 | -1.893205 |
| 6 | -3.289568 | -5.900374 | -2.012025 |
| 1 | -3.576073 | -5.312758 | 0.030685 |
| 6 | -1.298423 | -5.068600 | -3.074079 |
| 1 | -0.029092 | -3.819596 | -1.901335 |
| 6 | -2.473266 | -5.814494 | -3.143859 |
| 1 | -4.204952 | -6.485776 | -2.039816 |
| 1 | -0.656314 | -4.979862 | -3.945006 |
| 1 | -2.750143 | -6.323937 | -4.062841 |
| 6 | -1.794257 | -4.533060 | 1.759854 |
| 6 | -1.210134 | -5.807894 | 1.842503 |
| 6 | -2.595333 | -4.107781 | 2.824054 |
| 6 | -1.424255 | -6.631445 | 2.943209 |
| 1 | -0.583366 | -6.158599 | 1.027123 |
| 6 | -2.814407 | -4.932910 | 3.931306 |
| 1 | -3.044430 | -3.124637 | 2.821728 |
| 6 | -2.234128 | -6.196780 | 3.997192 |
| 1 | -0.958710 | -7.612850 | 2.980791 |
| 1 | -3.438907 | -4.571272 | 4.743623 |
| 1 | -2.403678 | -6.837007 | 4.858803 |
| 7 | 0.159334 | -2.093578 | 1.626097 |
| 1 | 0.470629 | -1.183565 | 1.270419 |
| 16 | 0.411536 | -2.360172 | 3.236307 |
| 8 | -0.698038 | -1.759474 | 4.004602 |
| 1 | 0.205812 | -1.555488 | -4.041563 |
| 1 | 0.898059 | -0.802737 | -2.799707 |
| 1 | -2.036083 | -0.789947 | -3.321317 |
| 1 | -0.886899 | 0.560411 | -2.832287 |
| 8 | 0.710785 | -3.786843 | 3.430951 |
| 6 | 1.910565 | -1.404695 | 3.587598 |
| 6 | 2.160835 | -0.793287 | 4.862442 |
| 6 | 2.858712 | -1.387539 | 2.587451 |
| 6 | 1.256824 | -0.778193 | 5.963230 |
| 6 | 3.427067 | -0.126921 | 5.025992 |
| 6 | 4.107182 | -0.764164 | 2.782276 |
| 1 | 2.639433 | -1.823900 | 1.624379 |
| 6 | 1.577753 | -0.126887 | 7.134233 |
| 1 | 0.302028 | -1.275443 | 5.868609 |
| 6 | 3.715597 | 0.542781 | 6.245840 |
| 6 | 4.381259 | -0.141014 | 3.975235 |
| 1 | 4.827255 | -0.763995 | 1.974464 |
| 6 | 2.811378 | 0.548524 | 7.281282 |
| 1 | 0.867057 | -0.130333 | 7.956027 |
| 1 | 4.669473 | 1.055165 | 6.337756 |
| 1 | 5.328913 | 0.364996 | 4.127951 |
| 1 | 3.040772 | 1.065349 | 8.208770 |
| 6 | 4.685828 | -0.726046 | -1.360733 |
| 6 | 4.997236 | -1.654670 | -2.335186 |
| 6 | 6.253750 | -1.546029 | -3.018619 |
| 6 | 6.635589 | -2.445556 | -4.051107 |
| 1 | 5.952787 | -3.237864 | -4.335858 |
| 6 | 7.852833 | -2.319727 | -4.684875 |
| 1 | 8.124691 | -3.016217 | -5.472935 |
| 6 | 8.753367 | -1.290260 | -4.319021 |
| 1 | 9.709341 | -1.202894 | -4.827230 |
| 6 | 8.415407 | -0.405039 | -3.320799 |

| | | | |
|---|---|---|---|
| 1 | 9.099445 | 0.388244 | -3.030088 |
| 6 | 7.168168 | -0.508491 | -2.646592 |
| 6 | 6.811708 | 0.388055 | -1.609423 |
| 1 | 7.507707 | 1.175511 | -1.334633 |
| 6 | 5.596833 | 0.300031 | -0.958055 |
| 6 | 5.273865 | 1.215669 | 0.164308 |
| 6 | 6.215425 | 1.485562 | 1.140000 |
| 1 | 7.182420 | 0.992308 | 1.098000 |
| 6 | 5.937612 | 2.355281 | 2.222884 |
| 6 | 6.889368 | 2.589004 | 3.253008 |
| 1 | 7.855510 | 2.094317 | 3.191624 |
| 6 | 6.592789 | 3.418721 | 4.311422 |
| 1 | 7.325389 | 3.587071 | 5.095603 |
| 6 | 5.329799 | 4.056885 | 4.379507 |
| 1 | 5.100764 | 4.705922 | 5.219808 |
| 6 | 4.390008 | 3.859143 | 3.391217 |
| 1 | 3.423217 | 4.346974 | 3.448613 |
| 6 | 4.659860 | 2.999819 | 2.291838 |
| 6 | 3.698469 | 2.764452 | 1.257676 |
| 6 | 4.001999 | 1.859135 | 0.259174 |
| 6 | 4.046992 | -2.754432 | -2.702462 |
| 6 | 3.197533 | -2.534172 | -3.843913 |
| 6 | 3.198693 | -1.304844 | -4.562794 |
| 1 | 3.839119 | -0.499531 | -4.227673 |
| 6 | 2.374899 | -1.110642 | -5.649572 |
| 1 | 2.388315 | -0.158210 | -6.171054 |
| 6 | 1.499680 | -2.137481 | -6.084319 |
| 1 | 0.858378 | -1.975044 | -6.946163 |
| 6 | 1.473945 | -3.342639 | -5.413917 |
| 1 | 0.813608 | -4.142303 | -5.739623 |
| 6 | 2.312410 | -3.568644 | -4.288360 |
| 6 | 2.292969 | -4.795015 | -3.578692 |
| 1 | 1.625865 | -5.588552 | -3.902575 |
| 6 | 3.108611 | -4.976550 | -2.492451 |
| 1 | 3.074393 | -5.921754 | -1.957103 |
| 6 | 4.014737 | -3.976126 | -2.030692 |
| 6 | 2.451254 | 3.589782 | 1.171976 |
| 6 | 1.237635 | 3.153323 | 1.796470 |
| 6 | 1.147039 | 1.940208 | 2.533319 |
| 1 | 2.024849 | 1.312760 | 2.626190 |
| 6 | -0.044431 | 1.543904 | 3.100654 |
| 1 | -0.101384 | 0.612208 | 3.651802 |
| 6 | -1.208030 | 2.338640 | 2.959113 |
| 1 | -2.142380 | 2.012043 | 3.407167 |
| 6 | -1.155281 | 3.525524 | 2.263343 |
| 1 | -2.040451 | 4.142868 | 2.150028 |
| 6 | 0.060986 | 3.963597 | 1.674782 |
| 6 | 0.136476 | 5.179043 | 0.948134 |
| 1 | -0.762027 | 5.780660 | 0.844692 |
| 6 | 1.315216 | 5.575815 | 0.368555 |
| 1 | 1.344308 | 6.500649 | -0.199222 |
| 6 | 2.498336 | 4.793595 | 0.471765 |
| 8 | 1.840171 | 0.225938 | -2.365194 |
| 8 | 1.291743 | 0.026500 | 0.194394 |
| 8 | 3.496962 | -0.843611 | -0.661237 |
| 8 | 3.097431 | 1.668745 | -0.771390 |
| 15 | 2.269266 | 0.243706 | -0.915733 |
| 6 | 4.804452 | -4.356872 | -0.778454 |
| 6 | 6.288631 | -3.947810 | -0.694696 |
| 6 | 4.056832 | -3.885740 | 0.489858 |
| 1 | 4.799215 | -5.456221 | -0.753846 |
| 6 | 6.935135 | -4.524869 | 0.574875 |

| | | | |
|---|---|---|---|
| 1 | 6.378906 | -2.859512 | -0.658010 |
| 1 | 6.825226 | -4.278195 | -1.591138 |
| 6 | 4.704343 | -4.418671 | 1.773377 |
| 1 | 4.062938 | -2.793775 | 0.496023 |
| 1 | 3.005394 | -4.191791 | 0.438015 |
| 6 | 6.194321 | -4.055789 | 1.836815 |
| 1 | 7.990557 | -4.229190 | 0.619491 |
| 1 | 6.918160 | -5.623625 | 0.531488 |
| 1 | 4.171871 | -4.023037 | 2.645645 |
| 1 | 4.595670 | -5.512131 | 1.807061 |
| 1 | 6.654893 | -4.486065 | 2.734492 |
| 1 | 6.292580 | -2.964127 | 1.925818 |
| 6 | 3.761501 | 5.265949 | -0.231084 |
| 6 | 3.660010 | 5.068234 | -1.761571 |
| 6 | 4.141754 | 6.723025 | 0.106669 |
| 1 | 4.595527 | 4.643602 | 0.107628 |
| 6 | 4.953993 | 5.495765 | -2.469059 |
| 1 | 2.814581 | 5.657128 | -2.143592 |
| 1 | 3.439073 | 4.017318 | -1.976429 |
| 6 | 5.444473 | 7.132181 | -0.596700 |
| 1 | 3.340621 | 7.405043 | -0.207526 |
| 1 | 4.240680 | 6.832324 | 1.193354 |
| 6 | 5.341686 | 6.939166 | -2.116911 |
| 1 | 4.844183 | 5.382366 | -3.554747 |
| 1 | 5.765183 | 4.818766 | -2.164680 |
| 1 | 5.689537 | 8.174737 | -0.359332 |
| 1 | 6.270724 | 6.518841 | -0.209552 |
| 1 | 6.288345 | 7.209143 | -2.601028 |
| 1 | 4.578381 | 7.624007 | -2.514455 |

---

(*S*,*S*,*S*)-**[1a-2a]**$^{‡}$endo-*re*

---

Number of imaginary frequencies : 1
The smallest frequencies are : -346.7453 10.1613 11.9239 cm(-1)

Electronic energy : HF=-6613.9973723
Zero-point correction= 1.888089 (Hartree/Particle)
Thermal correction to Energy= 1.998369
Thermal correction to Enthalpy= 1.999313
Thermal correction to Gibbs Free Energy= 1.735126
Sum of electronic and zero-point Energies= -6612.109283
Sum of electronic and thermal Energies= -6611.999003
Sum of electronic and thermal Enthalpies= -6611.998059
Sum of electronic and thermal Free Energies= -6612.262246

Cartesian Coordinates

| | | | |
|---|---|---|---|
| 6 | -3.260053 | -5.074562 | -0.279374 |
| 6 | -3.493130 | -3.696518 | -0.143501 |
| 6 | -3.922240 | -3.200438 | 1.094773 |
| 6 | -4.128521 | -4.060948 | 2.174638 |
| 6 | -3.911395 | -5.431603 | 2.025139 |
| 6 | -3.480203 | -5.936997 | 0.794732 |
| 15 | -3.213694 | -2.506945 | -1.523008 |
| 6 | -4.500252 | -2.900563 | -2.787518 |
| 6 | -5.292705 | -4.055683 | -2.783363 |
| 6 | -6.268865 | -4.247895 | -3.765037 |
| 6 | -6.465843 | -3.290169 | -4.761032 |
| 6 | -5.678028 | -2.135314 | -4.775970 |
| 6 | -4.706019 | -1.941718 | -3.795328 |
| 46 | -3.249164 | -0.184564 | -0.994200 |

| | | | |
|---|---|---|---|
| 6 | -2.302527 | 1.764555 | -1.006864 |
| 6 | -1.312888 | 0.783084 | -1.278505 |
| 15 | -5.370062 | 0.366313 | -0.108037 |
| 6 | -6.380502 | 1.563356 | -1.085280 |
| 6 | -5.714084 | 2.601878 | -1.755022 |
| 6 | -6.428437 | 3.563706 | -2.468403 |
| 6 | -7.821928 | 3.495201 | -2.532700 |
| 6 | -8.494824 | 2.464928 | -1.872386 |
| 6 | -7.781739 | 1.506267 | -1.149156 |
| 6 | -1.673827 | -3.170755 | -2.298483 |
| 6 | -1.686557 | -4.029648 | -3.408134 |
| 6 | -0.485988 | -4.494680 | -3.952171 |
| 6 | 0.734371 | -4.104389 | -3.398733 |
| 6 | 0.756843 | -3.250961 | -2.292956 |
| 6 | -0.439991 | -2.791055 | -1.744275 |
| 6 | -5.223401 | 1.169759 | 1.545187 |
| 6 | -6.075627 | 2.195133 | 1.978960 |
| 6 | -5.925462 | 2.741171 | 3.255950 |
| 6 | -4.932716 | 2.264573 | 4.114544 |
| 6 | -4.076407 | 1.245172 | 3.689446 |
| 6 | -4.217786 | 0.707902 | 2.410039 |
| 6 | -6.557066 | -1.008644 | 0.225151 |
| 6 | -7.012935 | -1.774774 | -0.860705 |
| 6 | -7.852728 | -2.867964 | -0.653115 |
| 6 | -8.239979 | -3.221594 | 0.641779 |
| 6 | -7.795021 | -2.464396 | 1.725937 |
| 6 | -6.963773 | -1.360850 | 1.520641 |
| 1 | -2.691827 | 2.271462 | -1.889872 |
| 1 | -2.629250 | -4.334218 | -3.851704 |
| 1 | -0.414620 | -2.125624 | -0.887250 |
| 1 | -0.508718 | -5.160361 | -4.811049 |
| 1 | 1.698496 | -2.940343 | -1.859396 |
| 1 | 1.667949 | -4.466864 | -3.818428 |
| 1 | -5.169528 | -4.800574 | -2.005191 |
| 1 | -4.115655 | -1.028836 | -3.795034 |
| 1 | -6.879719 | -5.146292 | -3.744427 |
| 1 | -5.828000 | -1.381004 | -5.543289 |
| 1 | -7.230088 | -3.438951 | -5.518523 |
| 1 | -4.088251 | -2.136966 | 1.213343 |
| 1 | -2.886213 | -5.470197 | -1.218892 |
| 1 | -4.455315 | -3.657371 | 3.128493 |
| 1 | -3.299463 | -7.001832 | 0.677223 |
| 1 | -4.069416 | -6.103957 | 2.863543 |
| 1 | -6.853616 | 2.572932 | 1.323216 |
| 1 | -3.530226 | -0.061104 | 2.070799 |
| 1 | -6.584433 | 3.542683 | 3.577488 |
| 1 | -3.282950 | 0.889576 | 4.338420 |
| 1 | -4.814434 | 2.694687 | 5.104705 |
| 1 | -4.634430 | 2.662827 | -1.714254 |
| 1 | -8.318940 | 0.715446 | -0.636345 |
| 1 | -5.889808 | 4.360189 | -2.973651 |
| 1 | -9.578877 | 2.406342 | -1.915634 |
| 1 | -8.380459 | 4.239540 | -3.093260 |
| 1 | -6.709413 | -1.521246 | -1.870889 |
| 1 | -6.631059 | -0.779777 | 2.373830 |
| 1 | -8.189797 | -3.449404 | -1.505664 |
| 1 | -8.094999 | -2.728111 | 2.736354 |
| 1 | -8.884595 | -4.080933 | 0.803419 |
| 1 | -0.636079 | 0.441320 | -0.499800 |
| 6 | -0.976583 | 0.448697 | -2.625742 |
| 8 | 0.495516 | 1.498345 | -3.010200 |
| 6 | -2.344768 | 2.603318 | 0.245349 |

| | | | |
|---|---|---|---|
| 6 | -1.396818 | 3.848388 | 0.206870 |
| 1 | -2.092337 | 2.002438 | 1.121043 |
| 1 | -3.366739 | 2.963145 | 0.386743 |
| 6 | 0.082835 | 3.376268 | 0.385715 |
| 1 | 0.386450 | 2.766442 | -0.466839 |
| 1 | 0.746400 | 4.243926 | 0.424225 |
| 6 | -1.621428 | 4.546512 | -1.147801 |
| 6 | -2.835513 | 5.227351 | -1.353472 |
| 6 | -0.735916 | 4.449231 | -2.228474 |
| 6 | -3.156231 | 5.785385 | -2.589427 |
| 1 | -3.535510 | 5.320656 | -0.528713 |
| 6 | -1.055270 | 5.010424 | -3.470434 |
| 1 | 0.198382 | 3.913586 | -2.133020 |
| 6 | -2.263429 | 5.678950 | -3.660656 |
| 1 | -4.099786 | 6.310560 | -2.713356 |
| 1 | -0.352390 | 4.912276 | -4.293532 |
| 1 | -2.507427 | 6.113887 | -4.626010 |
| 6 | -1.733874 | 4.837515 | 1.336609 |
| 6 | -1.161913 | 6.120647 | 1.306688 |
| 6 | -2.569232 | 4.514863 | 2.409412 |
| 6 | -1.422011 | 7.050901 | 2.307213 |
| 1 | -0.505389 | 6.389182 | 0.483942 |
| 6 | -2.836933 | 5.448095 | 3.415556 |
| 1 | -3.007227 | 3.529780 | 2.491518 |
| 6 | -2.268444 | 6.718226 | 3.369863 |
| 1 | -0.963730 | 8.035322 | 2.261350 |
| 1 | -3.486967 | 5.166378 | 4.239412 |
| 1 | -2.474173 | 7.442010 | 4.153991 |
| 7 | 0.278492 | 2.515575 | 1.551623 |
| 1 | 0.624528 | 1.570290 | 1.358676 |
| 16 | 0.623085 | 3.100208 | 3.070595 |
| 8 | -0.490541 | 2.793456 | 3.984612 |
| 1 | 0.707805 | 1.510116 | -3.957397 |
| 1 | 1.213305 | 0.884115 | -2.553922 |
| 1 | -1.625774 | 0.815065 | -3.416690 |
| 1 | -0.485677 | -0.496792 | -2.822487 |
| 8 | 1.048777 | 4.503552 | 2.939374 |
| 6 | 2.058910 | 2.097021 | 3.547075 |
| 6 | 2.208760 | 1.462329 | 4.826501 |
| 6 | 3.058603 | 2.044982 | 2.597884 |
| 6 | 1.268758 | 1.514698 | 5.896174 |
| 6 | 3.417193 | 0.706500 | 5.036350 |
| 6 | 4.253381 | 1.340922 | 2.844819 |
| 1 | 2.924681 | 2.523924 | 1.636326 |
| 6 | 1.499740 | 0.843062 | 7.076957 |
| 1 | 0.362615 | 2.087733 | 5.768008 |
| 6 | 3.606106 | 0.006828 | 6.259464 |
| 6 | 4.419084 | 0.672831 | 4.032716 |
| 1 | 5.017722 | 1.309254 | 2.078337 |
| 6 | 2.668673 | 0.069337 | 7.262971 |
| 1 | 0.763399 | 0.902240 | 7.873583 |
| 1 | 4.511796 | -0.581543 | 6.381139 |
| 1 | 5.319033 | 0.096124 | 4.220624 |
| 1 | 2.822551 | -0.468953 | 8.193752 |
| 6 | 4.891995 | 0.586859 | -0.661008 |
| 6 | 5.376111 | 1.433934 | -1.639761 |
| 6 | 6.770038 | 1.396502 | -1.954950 |
| 6 | 7.373372 | 2.315327 | -2.856416 |
| 1 | 6.757765 | 3.085255 | -3.308341 |
| 6 | 8.718139 | 2.239871 | -3.147496 |
| 1 | 9.162701 | 2.955195 | -3.833524 |
| 6 | 9.527391 | 1.237332 | -2.559026 |

| | | | |
|---|---|---|---|
| 1 | 10.584408 | 1.185077 | -2.803441 |
| 6 | 8.975918 | 0.338455 | -1.674579 |
| 1 | 9.590835 | -0.429125 | -1.211472 |
| 6 | 7.595255 | 0.398569 | -1.339160 |
| 6 | 7.015466 | -0.511760 | -0.420916 |
| 1 | 7.637219 | -1.297325 | -0.001331 |
| 6 | 5.685080 | -0.426794 | -0.048772 |
| 6 | 5.116937 | -1.369038 | 0.948804 |
| 6 | 5.844574 | -1.715374 | 2.071902 |
| 1 | 6.792196 | -1.220496 | 2.264810 |
| 6 | 5.383268 | -2.685069 | 2.992999 |
| 6 | 6.119023 | -3.000803 | 4.168353 |
| 1 | 7.041506 | -2.460249 | 4.366319 |
| 6 | 5.678625 | -3.974609 | 5.035807 |
| 1 | 6.246069 | -4.206095 | 5.932645 |
| 6 | 4.490642 | -4.690209 | 4.749433 |
| 1 | 4.157767 | -5.471651 | 5.426529 |
| 6 | 3.755266 | -4.406360 | 3.619223 |
| 1 | 2.851706 | -4.964413 | 3.403205 |
| 6 | 4.158702 | -3.378569 | 2.723184 |
| 6 | 3.389481 | -3.021181 | 1.568189 |
| 6 | 3.850238 | -2.004142 | 0.751873 |
| 6 | 4.432091 | 2.351225 | -2.349938 |
| 6 | 3.980338 | 3.528389 | -1.655161 |
| 6 | 4.389317 | 3.828983 | -0.325397 |
| 1 | 5.086943 | 3.166295 | 0.173740 |
| 6 | 3.899710 | 4.930117 | 0.342498 |
| 1 | 4.202721 | 5.118542 | 1.367906 |
| 6 | 2.987953 | 5.809646 | -0.289266 |
| 1 | 2.600252 | 6.665413 | 0.254961 |
| 6 | 2.590908 | 5.566658 | -1.584726 |
| 1 | 1.885054 | 6.227351 | -2.080630 |
| 6 | 3.070073 | 4.430584 | -2.291895 |
| 6 | 2.653555 | 4.144869 | -3.616813 |
| 1 | 1.978232 | 4.833586 | -4.116857 |
| 6 | 3.084552 | 3.006509 | -4.248042 |
| 1 | 2.744006 | 2.799870 | -5.260880 |
| 6 | 3.967234 | 2.066093 | -3.633107 |
| 6 | 2.200290 | -3.845309 | 1.172592 |
| 6 | 2.427948 | -4.891808 | 0.210990 |
| 6 | 3.680895 | -5.085597 | -0.437099 |
| 1 | 4.511828 | -4.434980 | -0.190263 |
| 6 | 3.853327 | -6.077941 | -1.377832 |
| 1 | 4.818438 | -6.197799 | -1.862436 |
| 6 | 2.785564 | -6.942081 | -1.718792 |
| 1 | 2.933285 | -7.718686 | -2.464064 |
| 6 | 1.563882 | -6.791011 | -1.102961 |
| 1 | 0.731197 | -7.441527 | -1.359199 |
| 6 | 1.354341 | -5.774449 | -0.133960 |
| 6 | 0.095299 | -5.593146 | 0.490478 |
| 1 | -0.721020 | -6.260416 | 0.233469 |
| 6 | -0.102574 | -4.574026 | 1.385762 |
| 1 | -1.084779 | -4.444435 | 1.828372 |
| 6 | 0.942782 | -3.673306 | 1.741840 |
| 8 | 1.972910 | -0.162190 | -1.960191 |
| 8 | 1.352609 | 0.029935 | 0.580063 |
| 8 | 3.613620 | 0.795334 | -0.186180 |
| 8 | 3.116045 | -1.685007 | -0.379364 |
| 15 | 2.360531 | -0.222499 | -0.496601 |
| 6 | 4.293479 | 0.849727 | -4.509495 |
| 6 | 4.639935 | -0.497860 | -3.844650 |
| 6 | 5.361257 | 1.196007 | -5.578535 |

| | | | |
|---|---|---|---|
| 1 | 3.361342 | 0.662145 | -5.066063 |
| 6 | 4.723739 | -1.609398 | -4.902298 |
| 1 | 5.607270 | -0.437143 | -3.339893 |
| 1 | 3.889568 | -0.744679 | -3.093192 |
| 6 | 5.485639 | 0.080401 | -6.626998 |
| 1 | 6.326223 | 1.339711 | -5.078818 |
| 1 | 5.112884 | 2.146812 | -6.065854 |
| 6 | 5.770967 | -1.280378 | -5.975617 |
| 1 | 4.962361 | -2.562141 | -4.414006 |
| 1 | 3.740059 | -1.740641 | -5.377204 |
| 1 | 6.272608 | 0.332182 | -7.348744 |
| 1 | 4.546449 | 0.017042 | -7.196049 |
| 1 | 5.805261 | -2.067887 | -6.738814 |
| 1 | 6.764485 | -1.251153 | -5.505756 |
| 6 | 0.637558 | -2.557386 | 2.722755 |
| 6 | 0.359042 | -3.079613 | 4.152040 |
| 6 | -0.548516 | -1.681166 | 2.262079 |
| 1 | 1.513249 | -1.901072 | 2.771498 |
| 6 | 0.113746 | -1.919462 | 5.129661 |
| 1 | -0.521401 | -3.738073 | 4.125727 |
| 1 | 1.200287 | -3.687329 | 4.501999 |
| 6 | -0.722518 | -0.498170 | 3.218172 |
| 1 | -1.468776 | -2.280204 | 2.243507 |
| 1 | -0.364771 | -1.321638 | 1.249886 |
| 6 | -1.001872 | -0.980748 | 4.646181 |
| 1 | -0.118605 | -2.311577 | 6.128143 |
| 1 | 1.040388 | -1.342044 | 5.230694 |
| 1 | -1.506888 | 0.178878 | 2.870442 |
| 1 | 0.201110 | 0.088233 | 3.218090 |
| 1 | -1.090040 | -0.124974 | 5.325132 |
| 1 | -1.965059 | -1.513760 | 4.669440 |

---

(*S*,*S*,*S*)-**[1a-2a]**‡exo-*si*

---

Number of imaginary frequencies : 1
The smallest frequencies are : -321.8704 9.2174 13.1574 cm(-1)

Electronic energy : HF=-6613.9963426
Zero-point correction= 1.889907 (Hartree/Particle)
Thermal correction to Energy= 1.999944
Thermal correction to Enthalpy= 2.000889
Thermal correction to Gibbs Free Energy= 1.737404
Sum of electronic and zero-point Energies= -6612.106436
Sum of electronic and thermal Energies= -6611.996398
Sum of electronic and thermal Enthalpies= -6611.995454
Sum of electronic and thermal Free Energies= -6612.258938

Cartesian Coordinates

| | | | |
|---|---|---|---|
| 6 | -4.068396 | 4.102602 | -0.124660 |
| 6 | -4.636771 | 2.835332 | -0.312278 |
| 6 | -5.374226 | 2.588914 | -1.485401 |
| 6 | -5.548692 | 3.592853 | -2.436264 |
| 6 | -4.968781 | 4.851390 | -2.244519 |
| 6 | -4.227221 | 5.101175 | -1.089327 |
| 15 | -4.297184 | 1.400494 | 0.798415 |
| 6 | -5.950679 | 0.994597 | 1.511865 |
| 6 | -7.111179 | 1.743680 | 1.276033 |
| 6 | -8.333569 | 1.339881 | 1.819508 |
| 6 | -8.412076 | 0.184988 | 2.599778 |
| 6 | -7.255079 | -0.557465 | 2.855591 |

| | | | |
|---|---|---|---|
| 6 | -6.034817 | -0.150772 | 2.319554 |
| 46 | -3.140215 | -0.181108 | -0.554198 |
| 6 | -1.128595 | 0.815799 | -0.722399 |
| 6 | -1.273752 | -0.077989 | -1.807800 |
| 15 | -4.754208 | -1.757044 | -1.305334 |
| 6 | -3.451728 | 2.123645 | 2.263488 |
| 6 | -2.163648 | 1.687805 | 2.599154 |
| 6 | -1.493638 | 2.241190 | 3.692628 |
| 6 | -2.109685 | 3.228335 | 4.461500 |
| 6 | -3.402407 | 3.656868 | 4.144934 |
| 6 | -4.073488 | 3.104210 | 3.055622 |
| 6 | -6.286791 | -0.979434 | -1.997415 |
| 6 | -6.358857 | -0.595792 | -3.346537 |
| 6 | -7.455690 | 0.123081 | -3.823636 |
| 6 | -8.500109 | 0.469603 | -2.963327 |
| 6 | -8.439894 | 0.087692 | -1.622441 |
| 6 | -7.342172 | -0.626435 | -1.139787 |
| 6 | -5.405363 | -2.890752 | -0.009363 |
| 6 | -4.686833 | -2.980399 | 1.190040 |
| 6 | -5.119035 | -3.814733 | 2.222488 |
| 6 | -6.287298 | -4.562950 | 2.068149 |
| 6 | -7.021091 | -4.471186 | 0.881415 |
| 6 | -6.584626 | -3.641463 | -0.151852 |
| 1 | -0.529989 | 0.444794 | 0.110056 |
| 1 | -1.683546 | 0.917497 | 2.005244 |
| 1 | -5.078953 | 3.436820 | 2.813880 |
| 1 | -0.495430 | 1.899027 | 3.935771 |
| 1 | -3.887295 | 4.420613 | 4.746711 |
| 1 | -1.580785 | 3.662015 | 5.303531 |
| 1 | -7.069951 | 2.632211 | 0.655600 |
| 1 | -5.142850 | -0.735001 | 2.517080 |
| 1 | -9.226438 | 1.927795 | 1.624934 |
| 1 | -7.302864 | -1.458661 | 3.459804 |
| 1 | -9.366471 | -0.133600 | 3.009281 |
| 1 | -5.809833 | 1.610771 | -1.655792 |
| 1 | -3.468169 | 4.311201 | 0.752564 |
| 1 | -6.127517 | 3.384564 | -3.331622 |
| 1 | -3.746969 | 6.063543 | -0.940320 |
| 1 | -5.086877 | 5.627676 | -2.995207 |
| 1 | -5.562666 | -0.857147 | -4.034819 |
| 1 | -7.309319 | -0.896937 | -0.090773 |
| 1 | -7.492984 | 0.408861 | -4.871203 |
| 1 | -9.240253 | 0.353465 | -0.938772 |
| 1 | -9.352401 | 1.030763 | -3.335523 |
| 1 | -3.795720 | -2.372267 | 1.312898 |
| 1 | -7.169740 | -3.565231 | -1.063282 |
| 1 | -4.550479 | -3.869160 | 3.146660 |
| 1 | -7.935924 | -5.044450 | 0.761461 |
| 1 | -6.630557 | -5.209371 | 2.870854 |
| 1 | -1.575901 | 0.286344 | -2.786525 |
| 6 | -0.879194 | -1.450099 | -1.692248 |
| 6 | -1.279818 | 2.318973 | -0.786660 |
| 6 | -0.156747 | 3.147412 | -1.495527 |
| 1 | -1.360529 | 2.659007 | 0.243638 |
| 1 | -2.216443 | 2.582713 | -1.272382 |
| 6 | 1.233834 | 2.733989 | -0.954148 |
| 1 | 1.449920 | 1.707721 | -1.242177 |
| 1 | 2.000234 | 3.370575 | -1.400310 |
| 6 | -0.255035 | 2.833749 | -2.999405 |
| 6 | -1.459936 | 3.099365 | -3.678039 |
| 6 | 0.778212 | 2.226412 | -3.724241 |
| 6 | -1.634804 | 2.743173 | -5.013196 |

| | | | |
|---|---|---|---|
| 1 | -2.270506 | 3.597416 | -3.154279 |
| 6 | 0.605369 | 1.863039 | -5.064391 |
| 1 | 1.730049 | 2.013809 | -3.255470 |
| 6 | -0.602002 | 2.110831 | -5.714234 |
| 1 | -2.578079 | 2.962153 | -5.507136 |
| 1 | 1.422226 | 1.376937 | -5.590524 |
| 1 | -0.737131 | 1.825709 | -6.754053 |
| 6 | -0.380642 | 4.646548 | -1.206197 |
| 6 | -0.168094 | 5.619715 | -2.193260 |
| 6 | -0.728655 | 5.094305 | 0.079796 |
| 6 | -0.327992 | 6.979875 | -1.921391 |
| 1 | 0.125667 | 5.314353 | -3.191723 |
| 6 | -0.894769 | 6.451957 | 0.354977 |
| 1 | -0.845448 | 4.387184 | 0.890561 |
| 6 | -0.702620 | 7.405477 | -0.646567 |
| 1 | -0.157514 | 7.706529 | -2.711508 |
| 1 | -1.166751 | 6.759976 | 1.361374 |
| 1 | -0.832009 | 8.463320 | -0.434168 |
| 7 | 1.308554 | 2.799310 | 0.521980 |
| 1 | 1.374925 | 1.858950 | 0.927243 |
| 16 | 2.528454 | 3.768077 | 1.158708 |
| 8 | 2.551757 | 5.005576 | 0.367456 |
| 1 | -0.577226 | -1.811474 | -0.713744 |
| 1 | -1.357363 | -2.185590 | -2.323747 |
| 8 | 3.771497 | 3.008445 | 1.347859 |
| 8 | 0.750081 | -1.666800 | -2.560245 |
| 1 | 1.423003 | -1.043554 | -2.071957 |
| 6 | -4.222453 | -2.871630 | -2.686920 |
| 6 | -3.452092 | -2.300959 | -3.716031 |
| 6 | -4.534346 | -4.237420 | -2.759954 |
| 6 | -3.024048 | -3.068574 | -4.799592 |
| 1 | -3.174844 | -1.252058 | -3.659137 |
| 6 | -4.096173 | -5.007749 | -3.840517 |
| 1 | -5.112878 | -4.710244 | -1.974215 |
| 6 | -3.345991 | -4.426441 | -4.864882 |
| 1 | -2.428267 | -2.607771 | -5.582113 |
| 1 | -4.342021 | -6.065305 | -3.878083 |
| 1 | -3.005510 | -5.028716 | -5.702150 |
| 1 | 1.061865 | -2.571430 | -2.385276 |
| 6 | 1.884667 | 4.146612 | 2.798855 |
| 6 | 1.871742 | 3.167836 | 3.843669 |
| 6 | 1.452839 | 5.438768 | 3.002720 |
| 6 | 2.267625 | 1.809131 | 3.686157 |
| 6 | 1.384298 | 3.593002 | 5.128130 |
| 6 | 0.984362 | 5.838955 | 4.274907 |
| 1 | 1.478539 | 6.141248 | 2.177795 |
| 6 | 2.168547 | 0.922972 | 4.736542 |
| 1 | 2.651439 | 1.450456 | 2.741602 |
| 6 | 1.305505 | 2.649363 | 6.188311 |
| 6 | 0.958099 | 4.936885 | 5.312094 |
| 1 | 0.651277 | 6.861609 | 4.423537 |
| 6 | 1.683558 | 1.339645 | 5.998458 |
| 1 | 2.465998 | -0.109745 | 4.583065 |
| 1 | 0.931021 | 2.982736 | 7.152886 |
| 1 | 0.601267 | 5.237584 | 6.294096 |
| 1 | 1.608620 | 0.624542 | 6.812609 |
| 6 | 5.258003 | -0.770170 | 0.233958 |
| 6 | 6.145548 | -0.109443 | -0.597969 |
| 6 | 7.464207 | -0.642161 | -0.760212 |
| 6 | 8.470808 | 0.038068 | -1.501202 |
| 1 | 8.229333 | 0.993090 | -1.954278 |
| 6 | 9.732364 | -0.498212 | -1.636722 |

| | | | |
|---|---|---|---|
| 1 | 10.489010 | 0.039360 | -2.201147 |
| 6 | 10.053034 | -1.745791 | -1.046704 |
| 1 | 11.050385 | -2.159146 | -1.166796 |
| 6 | 9.103222 | -2.427247 | -0.320834 |
| 1 | 9.340138 | -3.383269 | 0.139492 |
| 6 | 7.796162 | -1.893446 | -0.148742 |
| 6 | 6.806648 | -2.580104 | 0.593686 |
| 1 | 7.041768 | -3.565718 | 0.984398 |
| 6 | 5.547979 | -2.045075 | 0.809813 |
| 6 | 4.541093 | -2.849761 | 1.549677 |
| 6 | 4.914619 | -3.627639 | 2.630707 |
| 1 | 5.905532 | -3.503556 | 3.057547 |
| 6 | 4.062722 | -4.622265 | 3.169070 |
| 6 | 4.476764 | -5.440050 | 4.256475 |
| 1 | 5.444246 | -5.246562 | 4.712838 |
| 6 | 3.680574 | -6.465175 | 4.713806 |
| 1 | 4.008538 | -7.085049 | 5.543220 |
| 6 | 2.437801 | -6.725181 | 4.087391 |
| 1 | 1.824298 | -7.552777 | 4.432227 |
| 6 | 2.002302 | -5.941733 | 3.040618 |
| 1 | 1.054116 | -6.155751 | 2.561515 |
| 6 | 2.781871 | -4.851186 | 2.566987 |
| 6 | 2.340167 | -3.978121 | 1.518907 |
| 6 | 3.203483 | -2.995579 | 1.063982 |
| 6 | 5.727506 | 1.140507 | -1.309652 |
| 6 | 6.015469 | 2.405155 | -0.706489 |
| 6 | 6.720818 | 2.517730 | 0.523696 |
| 1 | 7.043547 | 1.614477 | 1.029850 |
| 6 | 6.988755 | 3.748703 | 1.075914 |
| 1 | 7.528827 | 3.812554 | 2.016739 |
| 6 | 6.549047 | 4.934747 | 0.439905 |
| 1 | 6.751265 | 5.899590 | 0.896841 |
| 6 | 5.857667 | 4.862028 | -0.746646 |
| 1 | 5.501842 | 5.764461 | -1.236668 |
| 6 | 5.587695 | 3.607169 | -1.354982 |
| 6 | 4.903518 | 3.502915 | -2.591305 |
| 1 | 4.564475 | 4.411464 | -3.083203 |
| 6 | 4.676672 | 2.275249 | -3.169214 |
| 1 | 4.164096 | 2.231062 | -4.123964 |
| 6 | 5.080878 | 1.063641 | -2.545092 |
| 6 | 0.997787 | -4.145250 | 0.876507 |
| 6 | 0.926318 | -4.562755 | -0.502668 |
| 6 | 2.083777 | -4.786814 | -1.303704 |
| 1 | 3.064947 | -4.699115 | -0.853819 |
| 6 | 1.981464 | -5.089769 | -2.646072 |
| 1 | 2.883653 | -5.232618 | -3.233673 |
| 6 | 0.712920 | -5.210891 | -3.264591 |
| 1 | 0.645519 | -5.433059 | -4.325439 |
| 6 | -0.428643 | -5.046719 | -2.513029 |
| 1 | -1.409918 | -5.140293 | -2.968490 |
| 6 | -0.352528 | -4.725730 | -1.130282 |
| 6 | -1.525169 | -4.523359 | -0.358505 |
| 1 | -2.496864 | -4.657835 | -0.824704 |
| 6 | -1.427319 | -4.130206 | 0.949812 |
| 1 | -2.333351 | -3.974269 | 1.518529 |
| 6 | -0.175823 | -3.903787 | 1.588105 |
| 8 | 2.372075 | -0.202763 | -1.346618 |
| 8 | 1.625762 | -0.157794 | 1.157308 |
| 8 | 4.089427 | -0.132992 | 0.604349 |
| 8 | 2.789830 | -2.227134 | -0.002917 |
| 15 | 2.602266 | -0.585792 | 0.101740 |
| 6 | -0.200508 | -3.354937 | 3.006217 |

| | | | |
|---|---|---|---|
| 6 | -0.993634 | -4.258376 | 3.978903 |
| 6 | -0.757060 | -1.908834 | 3.034199 |
| 1 | 0.826424 | -3.301548 | 3.379241 |
| 6 | -0.996765 | -3.688262 | 5.403126 |
| 1 | -2.032668 | -4.350364 | 3.633625 |
| 1 | -0.571921 | -5.268699 | 3.975978 |
| 6 | -0.787041 | -1.349809 | 4.463545 |
| 1 | -1.775205 | -1.902878 | 2.618072 |
| 1 | -0.140435 | -1.270734 | 2.392410 |
| 6 | -1.559651 | -2.261014 | 5.425316 |
| 1 | -1.575127 | -4.342628 | 6.067313 |
| 1 | 0.032549 | -3.678833 | 5.788610 |
| 1 | -1.221258 | -0.345714 | 4.461460 |
| 1 | 0.244817 | -1.240312 | 4.821814 |
| 1 | -1.531212 | -1.852536 | 6.443053 |
| 1 | -2.619063 | -2.286012 | 5.127963 |
| 6 | 4.835492 | -0.274204 | -3.234119 |
| 6 | 6.067569 | -0.760856 | -4.036119 |
| 6 | 3.606469 | -0.317998 | -4.162766 |
| 1 | 4.650245 | -1.009680 | -2.444503 |
| 6 | 5.851857 | -2.190005 | -4.557768 |
| 1 | 6.234463 | -0.074671 | -4.879114 |
| 1 | 6.968216 | -0.725106 | -3.420155 |
| 6 | 3.355621 | -1.751218 | -4.652713 |
| 1 | 3.775957 | 0.325511 | -5.038022 |
| 1 | 2.729154 | 0.060422 | -3.641026 |
| 6 | 4.574828 | -2.305668 | -5.403460 |
| 1 | 6.724411 | -2.516848 | -5.137333 |
| 1 | 5.777041 | -2.867344 | -3.694758 |
| 1 | 2.463174 | -1.782225 | -5.289657 |
| 1 | 3.138655 | -2.389237 | -3.785008 |
| 1 | 4.404678 | -3.349707 | -5.697182 |
| 1 | 4.709708 | -1.736287 | -6.334758 |

---

(*S*,*S*,*S*)-**[1a-2a]**$^{\ddagger}$exo-*re*

---

Number of imaginary frequencies : 1
The smallest frequencies are : -250.7884 9.1633 11.8609 cm(-1)

Electronic energy : HF=-6613.9958513
Zero-point correction= 1.889104 (Hartree/Particle)
Thermal correction to Energy= 1.999587
Thermal correction to Enthalpy= 2.000531
Thermal correction to Gibbs Free Energy= 1.733988
Sum of electronic and zero-point Energies= -6612.106748
Sum of electronic and thermal Energies= -6611.996264
Sum of electronic and thermal Enthalpies= -6611.995320
Sum of electronic and thermal Free Energies= -6612.261863

Cartesian Coordinates

| | | | |
|---|---|---|---|
| 6 | 5.602358 | -3.156560 | 0.692199 |
| 6 | 5.189837 | -2.963468 | -0.639059 |
| 6 | 5.018720 | -4.081842 | -1.466025 |
| 6 | 5.270420 | -5.366474 | -0.975466 |
| 6 | 5.691612 | -5.548538 | 0.342725 |
| 6 | 5.856710 | -4.439041 | 1.176858 |
| 15 | 4.867043 | -1.230071 | -1.182085 |
| 6 | 4.141483 | -1.360313 | -2.865739 |
| 6 | 4.913823 | -1.775376 | -3.968327 |
| 6 | 4.337551 | -1.866437 | -5.232852 |

| | | | |
|---|---|---|---|
| 6 | 2.987837 | -1.541282 | -5.412895 |
| 6 | 2.220760 | -1.126180 | -4.326662 |
| 6 | 2.797270 | -1.028288 | -3.057258 |
| 46 | 3.649518 | -0.153891 | 0.527471 |
| 6 | 1.767206 | -1.319466 | 0.600229 |
| 6 | 1.904287 | -0.539825 | 1.777909 |
| 15 | 5.068455 | 1.511210 | 1.493249 |
| 6 | 6.549082 | -0.600151 | -1.613804 |
| 6 | 6.626255 | 0.685172 | -2.177666 |
| 6 | 7.858064 | 1.238157 | -2.520851 |
| 6 | 9.034870 | 0.511571 | -2.309163 |
| 6 | 8.965757 | -0.770210 | -1.762223 |
| 6 | 7.730310 | -1.324870 | -1.414089 |
| 6 | 6.703505 | 0.915426 | 2.128679 |
| 6 | 7.789103 | 0.779744 | 1.247635 |
| 6 | 8.995981 | 0.232490 | 1.684394 |
| 6 | 9.139886 | -0.199137 | 3.003498 |
| 6 | 8.067527 | -0.068382 | 3.887783 |
| 6 | 6.861162 | 0.485531 | 3.456820 |
| 6 | 4.340938 | 2.386759 | 2.953131 |
| 6 | 3.759838 | 1.611685 | 3.973597 |
| 6 | 3.143947 | 2.216055 | 5.068723 |
| 6 | 3.077210 | 3.609233 | 5.152064 |
| 6 | 3.637754 | 4.388089 | 4.138860 |
| 6 | 4.270460 | 3.784040 | 3.049028 |
| 1 | 1.157740 | -0.879155 | -0.181795 |
| 1 | 5.720084 | 1.258215 | -2.344618 |
| 1 | 7.694428 | -2.320564 | -0.984947 |
| 1 | 7.898198 | 2.237639 | -2.943884 |
| 1 | 9.874264 | -1.344067 | -1.601409 |
| 1 | 9.996722 | 0.942107 | -2.572828 |
| 1 | 5.963510 | -2.020228 | -3.836476 |
| 1 | 2.189384 | -0.697692 | -2.224898 |
| 1 | 4.939950 | -2.188250 | -6.078078 |
| 1 | 1.175695 | -0.872386 | -4.446623 |
| 1 | 2.538883 | -1.613720 | -6.399857 |
| 1 | 4.683269 | -3.957345 | -2.489916 |
| 1 | 5.722330 | -2.298386 | 1.348756 |
| 1 | 5.129289 | -6.224505 | -1.626701 |
| 1 | 6.176142 | -4.572246 | 2.206585 |
| 1 | 5.880452 | -6.548358 | 0.722529 |
| 1 | 7.701528 | 1.108934 | 0.220354 |
| 1 | 6.052802 | 0.593641 | 4.170469 |
| 1 | 9.818821 | 0.141325 | 0.982097 |
| 1 | 8.168363 | -0.389960 | 4.920736 |
| 1 | 10.079043 | -0.628245 | 3.340994 |
| 1 | 3.756647 | 0.528270 | 3.890829 |
| 1 | 4.697498 | 4.408434 | 2.272263 |
| 1 | 2.694099 | 1.599061 | 5.841009 |
| 1 | 3.580860 | 5.471635 | 4.190561 |
| 1 | 2.580103 | 4.082540 | 5.993561 |
| 1 | 2.255051 | -0.990695 | 2.702486 |
| 6 | 1.421772 | 0.794309 | 1.827253 |
| 6 | 1.853423 | -2.829750 | 0.582910 |
| 6 | 0.587751 | -3.617654 | 1.093556 |
| 1 | 2.706689 | -3.148680 | 1.188221 |
| 1 | 2.055496 | -3.148360 | -0.443837 |
| 6 | -0.553424 | -3.587906 | 0.022443 |
| 1 | -0.232730 | -4.234572 | -0.794758 |
| 1 | -1.460146 | -4.031059 | 0.445920 |
| 6 | 0.908412 | -5.121712 | 1.242418 |
| 6 | 2.019322 | -5.725500 | 0.638098 |

| | | | |
|---|---|---|---|
| 6 | 0.010282 | -5.946284 | 1.942143 |
| 6 | 2.234073 | -7.102236 | 0.744403 |
| 1 | 2.736146 | -5.136311 | 0.080660 |
| 6 | 0.217568 | -7.321303 | 2.040945 |
| 1 | -0.854375 | -5.501776 | 2.426181 |
| 6 | 1.336045 | -7.907838 | 1.443426 |
| 1 | 3.111181 | -7.539075 | 0.274705 |
| 1 | -0.493038 | -7.933511 | 2.589789 |
| 1 | 1.504004 | -8.978259 | 1.524113 |
| 6 | 0.153888 | -3.076000 | 2.463067 |
| 6 | 1.029562 | -3.215790 | 3.554218 |
| 6 | -1.079012 | -2.453699 | 2.692831 |
| 6 | 0.697001 | -2.732961 | 4.818645 |
| 1 | 1.980632 | -3.720322 | 3.408074 |
| 6 | -1.422976 | -1.973446 | 3.964224 |
| 1 | -1.798068 | -2.318284 | 1.898660 |
| 6 | -0.533396 | -2.103083 | 5.029900 |
| 1 | 1.396177 | -2.855118 | 5.641766 |
| 1 | -2.388250 | -1.497044 | 4.104024 |
| 1 | -0.796939 | -1.723887 | 6.012957 |
| 7 | -0.853606 | -2.284313 | -0.546070 |
| 1 | -1.414565 | -1.574685 | -0.051604 |
| 16 | -0.621348 | -1.915133 | -2.131507 |
| 8 | 0.290369 | -2.928848 | -2.689707 |
| 1 | 0.980893 | 1.211044 | 0.926717 |
| 1 | 1.834558 | 1.502606 | 2.527473 |
| 8 | -0.237495 | -0.495228 | -2.177422 |
| 8 | -0.209815 | 0.783192 | 2.892022 |
| 1 | -0.884083 | 1.313261 | 2.334857 |
| 6 | 5.475872 | 2.885040 | 0.341779 |
| 6 | 6.602206 | 3.711290 | 0.475917 |
| 6 | 4.568634 | 3.131194 | -0.700716 |
| 6 | 6.822152 | 4.752507 | -0.427563 |
| 1 | 7.311084 | 3.538667 | 1.279487 |
| 6 | 4.781083 | 4.183094 | -1.593215 |
| 1 | 3.700210 | 2.487956 | -0.814880 |
| 6 | 5.912869 | 4.991000 | -1.461596 |
| 1 | 7.701723 | 5.380938 | -0.320054 |
| 1 | 4.065481 | 4.367927 | -2.388576 |
| 1 | 6.084818 | 5.804646 | -2.160460 |
| 1 | -0.513911 | -0.139887 | 2.843795 |
| 6 | -2.233096 | -2.022713 | -2.949677 |
| 6 | -2.752317 | -3.223272 | -3.538007 |
| 6 | -2.982227 | -0.865973 | -2.879195 |
| 6 | -2.064816 | -4.467652 | -3.617447 |
| 6 | -4.090408 | -3.159015 | -4.067343 |
| 6 | -4.296569 | -0.830910 | -3.392725 |
| 1 | -2.564459 | 0.020132 | -2.421884 |
| 6 | -2.667434 | -5.576716 | -4.170744 |
| 1 | -1.052324 | -4.536199 | -3.245686 |
| 6 | -4.672174 | -4.322479 | -4.639599 |
| 6 | -4.834479 | -1.951533 | -3.978576 |
| 1 | -4.872521 | 0.085105 | -3.309643 |
| 6 | -3.981631 | -5.511118 | -4.688363 |
| 1 | -2.120580 | -6.514503 | -4.211840 |
| 1 | -5.684041 | -4.254505 | -5.030758 |
| 1 | -5.847757 | -1.936920 | -4.371741 |
| 1 | -4.439794 | -6.395411 | -5.121772 |
| 6 | -3.517112 | 2.873760 | -1.146234 |
| 6 | -2.693743 | 3.822132 | -1.725951 |
| 6 | -3.265575 | 5.069712 | -2.136881 |
| 6 | -2.514812 | 6.059945 | -2.828818 |

| | | | |
|---|---|---|---|
| 1 | -1.478034 | 5.855268 | -3.070351 |
| 6 | -3.089842 | 7.256451 | -3.198254 |
| 1 | -2.498140 | 7.998418 | -3.726985 |
| 6 | -4.447364 | 7.526422 | -2.899008 |
| 1 | -4.886070 | 8.476764 | -3.189364 |
| 6 | -5.209581 | 6.578985 | -2.254407 |
| 1 | -6.257503 | 6.768152 | -2.035465 |
| 6 | -4.650305 | 5.328386 | -1.873043 |
| 6 | -5.439366 | 4.320140 | -1.268268 |
| 1 | -6.489182 | 4.521983 | -1.075891 |
| 6 | -4.912001 | 3.088146 | -0.927549 |
| 6 | -5.797245 | 2.014746 | -0.406971 |
| 6 | -7.038138 | 1.802480 | -0.979129 |
| 1 | -7.322829 | 2.372072 | -1.859330 |
| 6 | -7.959839 | 0.871365 | -0.442038 |
| 6 | -9.243643 | 0.679257 | -1.022263 |
| 1 | -9.499437 | 1.242361 | -1.916450 |
| 6 | -10.149890 | -0.191544 | -0.461142 |
| 1 | -11.128507 | -0.329107 | -0.912035 |
| 6 | -9.811222 | -0.898730 | 0.717838 |
| 1 | -10.537294 | -1.567606 | 1.171262 |
| 6 | -8.571323 | -0.741476 | 1.298097 |
| 1 | -8.323399 | -1.277100 | 2.206979 |
| 6 | -7.599579 | 0.125899 | 0.727808 |
| 6 | -6.288406 | 0.283733 | 1.284916 |
| 6 | -5.429581 | 1.210630 | 0.716427 |
| 6 | -1.260551 | 3.508274 | -2.032266 |
| 6 | -0.988205 | 2.778111 | -3.239514 |
| 6 | -2.021624 | 2.316458 | -4.102026 |
| 1 | -3.054901 | 2.512815 | -3.839324 |
| 6 | -1.731800 | 1.626956 | -5.258993 |
| 1 | -2.540570 | 1.274571 | -5.892906 |
| 6 | -0.389901 | 1.370943 | -5.626498 |
| 1 | -0.172758 | 0.829211 | -6.542940 |
| 6 | 0.635076 | 1.807363 | -4.817822 |
| 1 | 1.669690 | 1.610799 | -5.084016 |
| 6 | 0.366908 | 2.505949 | -3.612148 |
| 6 | 1.409522 | 2.962717 | -2.766463 |
| 1 | 2.439959 | 2.748775 | -3.040559 |
| 6 | 1.120896 | 3.666852 | -1.623957 |
| 1 | 1.932867 | 4.025404 | -1.002114 |
| 6 | -0.216376 | 3.954237 | -1.226099 |
| 6 | -5.863716 | -0.496334 | 2.491960 |
| 6 | -5.765861 | 0.187647 | 3.753179 |
| 6 | -6.066840 | 1.570646 | 3.900744 |
| 1 | -6.386956 | 2.136579 | 3.033683 |
| 6 | -5.957794 | 2.199777 | 5.121053 |
| 1 | -6.190659 | 3.257726 | 5.203751 |
| 6 | -5.542090 | 1.482144 | 6.267872 |
| 1 | -5.455968 | 1.991414 | 7.223657 |
| 6 | -5.251448 | 0.140912 | 6.165646 |
| 1 | -4.935593 | -0.424952 | 7.038920 |
| 6 | -5.358046 | -0.536085 | 4.920770 |
| 6 | -5.072650 | -1.920832 | 4.800378 |
| 1 | -4.760451 | -2.474371 | 5.682694 |
| 6 | -5.185716 | -2.552259 | 3.587595 |
| 1 | -4.957862 | -3.611498 | 3.517408 |
| 6 | -5.582417 | -1.858732 | 2.408755 |
| 8 | -1.801082 | 2.034621 | 1.378888 |
| 8 | -2.518067 | -0.362116 | 0.610128 |
| 8 | -3.006388 | 1.616460 | -0.884136 |
| 8 | -4.230042 | 1.468450 | 1.340931 |

| | | | |
|---|---|---|---|
| 15 | -2.764988 | 1.115398 | 0.669462 |
| 6 | -5.658371 | -2.643095 | 1.108809 |
| 6 | -6.702374 | -3.783198 | 1.159487 |
| 6 | -4.280055 | -3.207297 | 0.696906 |
| 1 | -5.965020 | -1.960249 | 0.310087 |
| 6 | -6.787489 | -4.518806 | -0.186097 |
| 1 | -6.423927 | -4.498890 | 1.945509 |
| 1 | -7.683945 | -3.381715 | 1.431466 |
| 6 | -4.365722 | -3.954819 | -0.637332 |
| 1 | -3.909358 | -3.884796 | 1.479053 |
| 1 | -3.576739 | -2.376929 | 0.617029 |
| 6 | -5.419221 | -5.068885 | -0.612129 |
| 1 | -7.527006 | -5.327275 | -0.125883 |
| 1 | -7.148822 | -3.817454 | -0.951823 |
| 1 | -3.387085 | -4.364270 | -0.909779 |
| 1 | -4.624344 | -3.234124 | -1.420960 |
| 1 | -5.486123 | -5.546517 | -1.597083 |
| 1 | -5.107381 | -5.848965 | 0.098278 |
| 6 | -0.436360 | 4.765576 | 0.042854 |
| 6 | -0.132547 | 6.267468 | -0.173188 |
| 6 | 0.389938 | 4.250436 | 1.242081 |
| 1 | -1.491218 | 4.669113 | 0.321796 |
| 6 | -0.414227 | 7.085395 | 1.096621 |
| 1 | 0.924842 | 6.377327 | -0.456059 |
| 1 | -0.724868 | 6.660060 | -1.004416 |
| 6 | 0.086640 | 5.049886 | 2.515376 |
| 1 | 1.464024 | 4.332442 | 1.025435 |
| 1 | 0.159591 | 3.199752 | 1.398124 |
| 6 | 0.359733 | 6.545745 | 2.308281 |
| 1 | -0.169376 | 8.141202 | 0.924114 |
| 1 | -1.491650 | 7.043823 | 1.310240 |
| 1 | 0.685896 | 4.661859 | 3.347912 |
| 1 | -0.967227 | 4.899835 | 2.786350 |
| 1 | 0.101484 | 7.115295 | 3.210059 |
| 1 | 1.437342 | 6.692352 | 2.140140 |

---

(*S*,*S*,*S*)-**[1a-2a]**$^{\ddagger}$endo-*si*

---

Number of imaginary frequencies : 1
The smallest frequencies are : -263.1646 10.5696 11.8795 cm(-1)

Electronic energy : HF=-6614.0029339
Zero-point correction= 1.888623 (Hartree/Particle)
Thermal correction to Energy= 1.999084
Thermal correction to Enthalpy= 2.000028
Thermal correction to Gibbs Free Energy= 1.733849
Sum of electronic and zero-point Energies= -6612.114311
Sum of electronic and thermal Energies= -6612.003850
Sum of electronic and thermal Enthalpies= -6612.002906
Sum of electronic and thermal Free Energies= -6612.269085

.............................................................

Cartesian Coordinates

.............................................................

| | | | |
|---|---|---|---|
| 6 | -5.508430 | -1.398583 | 2.001091 |
| 6 | -6.387791 | -1.527202 | 0.912149 |
| 6 | -7.755526 | -1.710172 | 1.161463 |
| 6 | -8.232449 | -1.755825 | 2.473055 |
| 6 | -7.351445 | -1.622057 | 3.549186 |
| 6 | -5.986275 | -1.447374 | 3.310766 |
| 15 | -5.658057 | -1.364303 | -0.773955 |
| 6 | -7.047056 | -0.896994 | -1.892216 |

| | | | |
|---|---|---|---|
| 6 | -8.066074 | -1.804492 | -2.231261 |
| 6 | -9.109707 | -1.413075 | -3.069143 |
| 6 | -9.143377 | -0.115940 | -3.589363 |
| 6 | -8.124190 | 0.783910 | -3.276432 |
| 6 | -7.079001 | 0.393406 | -2.437264 |
| 46 | -3.780868 | 0.056060 | -0.741927 |
| 6 | -2.228511 | -1.363715 | -1.236829 |
| 6 | -1.586530 | -0.111775 | -1.023059 |
| 15 | -4.532228 | 2.135341 | 0.187987 |
| 6 | -5.364052 | -3.108152 | -1.299841 |
| 6 | -4.867641 | -3.317773 | -2.598116 |
| 6 | -4.562359 | -4.603614 | -3.039907 |
| 6 | -4.733909 | -5.698415 | -2.186054 |
| 6 | -5.227612 | -5.497990 | -0.897579 |
| 6 | -5.546687 | -4.210719 | -0.455235 |
| 6 | -6.339715 | 2.525006 | 0.115755 |
| 6 | -7.231263 | 1.759325 | 0.886080 |
| 6 | -8.608415 | 1.932137 | 0.758967 |
| 6 | -9.119917 | 2.871071 | -0.140111 |
| 6 | -8.242410 | 3.644366 | -0.901304 |
| 6 | -6.861526 | 3.475450 | -0.774534 |
| 6 | -3.809156 | 3.695312 | -0.482649 |
| 6 | -3.418519 | 3.709208 | -1.829884 |
| 6 | -2.971707 | 4.889343 | -2.428322 |
| 6 | -2.898165 | 6.066115 | -1.679744 |
| 6 | -3.272208 | 6.058170 | -0.333144 |
| 6 | -3.730780 | 4.881995 | 0.261692 |
| 1 | -2.415786 | -1.637437 | -2.274625 |
| 1 | -4.713447 | -2.469462 | -3.260162 |
| 1 | -5.927435 | -4.068373 | 0.550962 |
| 1 | -4.179242 | -4.750973 | -4.045878 |
| 1 | -5.354984 | -6.342517 | -0.227322 |
| 1 | -4.474123 | -6.698105 | -2.520904 |
| 1 | -8.037249 | -2.820921 | -1.850065 |
| 1 | -6.286778 | 1.093232 | -2.201116 |
| 1 | -9.893045 | -2.122423 | -3.321138 |
| 1 | -8.139264 | 1.792058 | -3.679474 |
| 1 | -9.957048 | 0.186880 | -4.242434 |
| 1 | -8.456024 | -1.790604 | 0.337760 |
| 1 | -4.450952 | -1.226378 | 1.826106 |
| 1 | -9.295870 | -1.887712 | 2.652871 |
| 1 | -5.292712 | -1.325775 | 4.137376 |
| 1 | -7.728112 | -1.646214 | 4.567793 |
| 1 | -6.848296 | 1.030535 | 1.590689 |
| 1 | -6.194845 | 4.085864 | -1.374628 |
| 1 | -9.279157 | 1.326057 | 1.360993 |
| 1 | -8.629765 | 4.384641 | -1.596070 |
| 1 | -10.193416 | 3.000638 | -0.243841 |
| 1 | -3.468425 | 2.791919 | -2.409705 |
| 1 | -4.021810 | 4.889206 | 1.306857 |
| 1 | -2.670198 | 4.886195 | -3.471534 |
| 1 | -3.202522 | 6.967591 | 0.256447 |
| 1 | -2.540484 | 6.982027 | -2.140437 |
| 1 | -1.045653 | 0.085555 | -0.106070 |
| 6 | -1.417377 | 0.820201 | -2.083253 |
| 6 | -2.122387 | -2.547805 | -0.293063 |
| 6 | -0.811374 | -3.438326 | -0.386718 |
| 1 | -2.972977 | -3.194084 | -0.504017 |
| 1 | -2.223051 | -2.207076 | 0.740149 |
| 6 | 0.137066 | -3.072896 | 0.799796 |
| 1 | -0.334099 | -3.453862 | 1.705741 |
| 1 | 1.090027 | -3.599083 | 0.689846 |

| | | | |
|---|---|---|---|
| 6 | -1.146319 | -4.923227 | -0.130228 |
| 6 | -2.118764 | -5.278637 | 0.819413 |
| 6 | -0.415429 | -5.955757 | -0.735684 |
| 6 | -2.375670 | -6.615292 | 1.125200 |
| 1 | -2.683115 | -4.506482 | 1.331778 |
| 6 | -0.671896 | -7.295254 | -0.433939 |
| 1 | 0.358580 | -5.713260 | -1.455899 |
| 6 | -1.657896 | -7.632935 | 0.493544 |
| 1 | -3.138574 | -6.859379 | 1.859768 |
| 1 | -0.094480 | -8.074245 | -0.924690 |
| 1 | -1.859045 | -8.674655 | 0.727734 |
| 6 | -0.172122 | -3.253869 | -1.767324 |
| 6 | -0.892928 | -3.670396 | -2.902666 |
| 6 | 1.069532 | -2.643427 | -1.972262 |
| 6 | -0.403179 | -3.464910 | -4.190587 |
| 1 | -1.854667 | -4.155116 | -2.764842 |
| 6 | 1.567173 | -2.432132 | -3.265887 |
| 1 | 1.678364 | -2.300695 | -1.148127 |
| 6 | 0.832163 | -2.835134 | -4.380803 |
| 1 | -0.985995 | -3.794488 | -5.046876 |
| 1 | 2.530610 | -1.949222 | -3.387976 |
| 1 | 1.220385 | -2.666175 | -5.381019 |
| 7 | 0.358457 | -1.640812 | 0.957682 |
| 1 | 1.230567 | -1.246515 | 0.557379 |
| 16 | 0.001041 | -0.848225 | 2.363328 |
| 8 | -1.193747 | -1.485615 | 2.954642 |
| 1 | -1.994237 | 0.700934 | -2.995060 |
| 1 | -1.143375 | 1.839155 | -1.836746 |
| 8 | -0.063408 | 0.575667 | 2.008551 |
| 8 | 0.213888 | 0.482182 | -2.992783 |
| 1 | 0.960651 | 0.753460 | -2.340753 |
| 6 | -4.184588 | 2.157120 | 1.998292 |
| 6 | -4.931525 | 2.923349 | 2.910089 |
| 6 | -3.153136 | 1.338095 | 2.481666 |
| 6 | -4.650483 | 2.864277 | 4.275258 |
| 1 | -5.745293 | 3.549201 | 2.557704 |
| 6 | -2.878959 | 1.273210 | 3.848846 |
| 1 | -2.554544 | 0.748770 | 1.798234 |
| 6 | -3.629643 | 2.033418 | 4.747511 |
| 1 | -5.236027 | 3.459195 | 4.970776 |
| 1 | -2.085247 | 0.620152 | 4.192927 |
| 1 | -3.421634 | 1.980113 | 5.812720 |
| 1 | 0.276781 | -0.487638 | -3.048223 |
| 6 | 1.423268 | -1.046372 | 3.461706 |
| 6 | 1.618853 | -2.189423 | 4.306059 |
| 6 | 2.387403 | -0.068253 | 3.328446 |
| 6 | 0.691778 | -3.256732 | 4.474821 |
| 6 | 2.864530 | -2.261291 | 5.023262 |
| 6 | 3.602472 | -0.162247 | 4.040763 |
| 1 | 2.221742 | 0.775476 | 2.672248 |
| 6 | 0.991916 | -4.338180 | 5.275330 |
| 1 | -0.267768 | -3.208905 | 3.979260 |
| 6 | 3.131211 | -3.386264 | 5.848778 |
| 6 | 3.832504 | -1.231732 | 4.871161 |
| 1 | 4.345464 | 0.617660 | 3.913469 |
| 6 | 2.221692 | -4.411818 | 5.968317 |
| 1 | 0.268512 | -5.142211 | 5.377319 |
| 1 | 4.080054 | -3.424728 | 6.377671 |
| 1 | 4.769804 | -1.317778 | 5.414862 |
| 1 | 2.442646 | -5.271922 | 6.593611 |
| 6 | 4.232797 | 2.797244 | 0.359047 |
| 6 | 3.607193 | 4.011184 | 0.588632 |

| | | | |
|---|---|---|---|
| 6 | 4.372367 | 5.213943 | 0.459864 |
| 6 | 3.832651 | 6.491765 | 0.770963 |
| 1 | 2.818587 | 6.548744 | 1.149875 |
| 6 | 4.582301 | 7.636352 | 0.608845 |
| 1 | 4.151424 | 8.603111 | 0.853730 |
| 6 | 5.913328 | 7.562813 | 0.130265 |
| 1 | 6.491270 | 8.473279 | -0.000393 |
| 6 | 6.475147 | 6.338739 | -0.153964 |
| 1 | 7.501650 | 6.270315 | -0.505637 |
| 6 | 5.732192 | 5.137871 | 0.014505 |
| 6 | 6.308363 | 3.868190 | -0.232043 |
| 1 | 7.336626 | 3.818944 | -0.578531 |
| 6 | 5.599006 | 2.695834 | -0.042103 |
| 6 | 6.268874 | 1.381954 | -0.219603 |
| 6 | 7.548305 | 1.190008 | 0.266830 |
| 1 | 8.018036 | 1.975507 | 0.851841 |
| 6 | 8.275155 | 0.002578 | 0.012484 |
| 6 | 9.603256 | -0.169443 | 0.490191 |
| 1 | 10.044560 | 0.619920 | 1.093627 |
| 6 | 10.322063 | -1.303043 | 0.186421 |
| 1 | 11.337340 | -1.422845 | 0.553560 |
| 6 | 9.741186 | -2.309407 | -0.622301 |
| 1 | 10.319424 | -3.192024 | -0.880900 |
| 6 | 8.450960 | -2.177763 | -1.088544 |
| 1 | 8.019421 | -2.949265 | -1.715333 |
| 6 | 7.668419 | -1.033750 | -0.769149 |
| 6 | 6.313400 | -0.877599 | -1.213768 |
| 6 | 5.653559 | 0.309077 | -0.938843 |
| 6 | 2.168317 | 4.033596 | 1.000884 |
| 6 | 1.842102 | 3.679073 | 2.353504 |
| 6 | 2.839090 | 3.423461 | 3.336667 |
| 1 | 3.884785 | 3.496232 | 3.057272 |
| 6 | 2.496473 | 3.095220 | 4.630414 |
| 1 | 3.275512 | 2.905136 | 5.363404 |
| 6 | 1.137051 | 2.990441 | 5.010156 |
| 1 | 0.880164 | 2.713158 | 6.028743 |
| 6 | 0.147766 | 3.239737 | 4.086204 |
| 1 | -0.900094 | 3.162017 | 4.359919 |
| 6 | 0.469650 | 3.597295 | 2.750741 |
| 6 | -0.535512 | 3.879848 | 1.791187 |
| 1 | -1.579443 | 3.788873 | 2.076338 |
| 6 | -0.194469 | 4.268739 | 0.520908 |
| 1 | -0.979956 | 4.502939 | -0.187742 |
| 6 | 1.161982 | 4.368724 | 0.098988 |
| 6 | 5.662292 | -1.961079 | -2.019721 |
| 6 | 5.540815 | -1.780175 | -3.441294 |
| 6 | 5.939185 | -0.582341 | -4.098953 |
| 1 | 6.362645 | 0.225238 | -3.513273 |
| 6 | 5.787466 | -0.429751 | -5.459548 |
| 1 | 6.092371 | 0.498733 | -5.934154 |
| 6 | 5.234229 | -1.469152 | -6.244922 |
| 1 | 5.116004 | -1.334001 | -7.316414 |
| 6 | 4.852053 | -2.648099 | -5.645912 |
| 1 | 4.430368 | -3.458063 | -6.236415 |
| 6 | 4.996450 | -2.835383 | -4.244607 |
| 6 | 4.602392 | -4.041939 | -3.611158 |
| 1 | 4.188764 | -4.844492 | -4.216587 |
| 6 | 4.721197 | -4.185162 | -2.252691 |
| 1 | 4.400453 | -5.111735 | -1.786641 |
| 6 | 5.243883 | -3.150378 | -1.425276 |
| 8 | 2.008503 | 1.253624 | -1.411438 |
| 8 | 2.791711 | -0.709873 | 0.143990 |

| | | | |
|---|---|---|---|
| 8 | 3.551170 | 1.629138 | 0.641904 |
| 8 | 4.404664 | 0.520952 | -1.486977 |
| 15 | 3.050267 | 0.599598 | -0.542934 |
| 6 | 5.300183 | -3.390810 | 0.075220 |
| 6 | 6.277110 | -4.523486 | 0.467320 |
| 6 | 3.899701 | -3.684010 | 0.659827 |
| 1 | 5.653844 | -2.471288 | 0.553716 |
| 6 | 6.337515 | -4.701851 | 1.992393 |
| 1 | 5.946308 | -5.463789 | 0.003803 |
| 1 | 7.277002 | -4.311572 | 0.076416 |
| 6 | 3.957241 | -3.865846 | 2.180534 |
| 1 | 3.487496 | -4.592920 | 0.199025 |
| 1 | 3.241662 | -2.852093 | 0.411474 |
| 6 | 4.947108 | -4.963897 | 2.588214 |
| 1 | 7.025970 | -5.517611 | 2.247171 |
| 1 | 6.754974 | -3.787491 | 2.437948 |
| 1 | 2.958823 | -4.088222 | 2.573531 |
| 1 | 4.260179 | -2.914792 | 2.635060 |
| 1 | 5.004419 | -5.036010 | 3.681435 |
| 1 | 4.578125 | -5.935789 | 2.228119 |
| 6 | 1.441571 | 4.892728 | -1.302314 |
| 6 | 1.017257 | 6.376533 | -1.424275 |
| 6 | 0.764190 | 4.065426 | -2.416056 |
| 1 | 2.520772 | 4.843572 | -1.481764 |
| 6 | 1.331880 | 6.944682 | -2.815177 |
| 1 | -0.061693 | 6.453425 | -1.235616 |
| 1 | 1.515215 | 6.974825 | -0.655062 |
| 6 | 1.075516 | 4.632383 | -3.808681 |
| 1 | -0.322718 | 4.071747 | -2.264765 |
| 1 | 1.103599 | 3.033372 | -2.341135 |
| 6 | 0.676294 | 6.109783 | -3.924250 |
| 1 | 1.003378 | 7.990190 | -2.877163 |
| 1 | 2.421412 | 6.949063 | -2.961037 |
| 1 | 0.565497 | 4.035026 | -4.575139 |
| 1 | 2.152836 | 4.533202 | -4.003542 |
| 1 | 0.944735 | 6.506079 | -4.911671 |
| 1 | -0.416967 | 6.194650 | -3.837138 |

---

(*S*,*R*,*S*)-**2a**endo-*re*

---

Number of imaginary frequencies : 0
The smallest frequencies are : 9.0746 12.0909 15.2051 cm(-1)

Electronic energy : HF=-6614.0307685
Zero-point correction= 1.888886 (Hartree/Particle)
Thermal correction to Energy= 2.000348
Thermal correction to Enthalpy= 2.001292
Thermal correction to Gibbs Free Energy= 1.734618
Sum of electronic and zero-point Energies= -6612.141883
Sum of electronic and thermal Energies= -6612.030421
Sum of electronic and thermal Enthalpies= -6612.029477
Sum of electronic and thermal Free Energies= -6612.296151

.............................................................
Cartesian Coordinates
.............................................................

| | | | |
|---|---|---|---|
| 6 | 5.862285 | -1.648368 | 2.195761 |
| 6 | 6.465889 | -1.508058 | 0.931765 |
| 6 | 7.857446 | -1.379840 | 0.855180 |
| 6 | 8.631050 | -1.411640 | 2.017115 |
| 6 | 8.028535 | -1.573095 | 3.264795 |
| 6 | 6.639045 | -1.688187 | 3.352867 |

| | | | |
|---|---|---|---|
| 15 | 5.322819 | -1.523616 | -0.510961 |
| 6 | 6.236573 | -1.026610 | -2.026347 |
| 6 | 7.410931 | -1.678526 | -2.440719 |
| 6 | 8.059209 | -1.282358 | -3.609605 |
| 6 | 7.526532 | -0.256352 | -4.397119 |
| 6 | 6.332373 | 0.359384 | -4.021087 |
| 6 | 5.689817 | -0.027483 | -2.842917 |
| 46 | 3.507097 | -0.117205 | 0.052663 |
| 6 | 1.752191 | -1.555002 | -0.051783 |
| 6 | 1.505877 | -0.559167 | 0.909193 |
| 15 | 4.658042 | 1.949196 | 0.281076 |
| 6 | 5.098441 | -3.323003 | -0.861028 |
| 6 | 4.544671 | -3.687242 | -2.100713 |
| 6 | 4.354828 | -5.030028 | -2.422799 |
| 6 | 4.709550 | -6.027614 | -1.511276 |
| 6 | 5.256657 | -5.673182 | -0.278482 |
| 6 | 5.449748 | -4.328887 | 0.049595 |
| 6 | 6.491123 | 1.949305 | 0.530327 |
| 6 | 7.030280 | 1.995524 | 1.825833 |
| 6 | 8.411640 | 1.979989 | 2.019456 |
| 6 | 9.276609 | 1.915192 | 0.925985 |
| 6 | 8.749935 | 1.853945 | -0.365284 |
| 6 | 7.369046 | 1.867882 | -0.563288 |
| 6 | 4.421754 | 2.989768 | -1.217750 |
| 6 | 3.409803 | 2.627348 | -2.122533 |
| 6 | 3.267920 | 3.306245 | -3.333061 |
| 6 | 4.127120 | 4.359806 | -3.650723 |
| 6 | 5.117112 | 4.748146 | -2.743491 |
| 6 | 5.268027 | 4.066550 | -1.534828 |
| 1 | 1.529797 | -1.287988 | -1.083048 |
| 1 | 4.261491 | -2.920218 | -2.816128 |
| 1 | 5.881413 | -4.072670 | 1.010972 |
| 1 | 3.921965 | -5.296867 | -3.382445 |
| 1 | 5.534125 | -6.442673 | 0.436158 |
| 1 | 4.546581 | -7.072240 | -1.756612 |
| 1 | 7.809780 | -2.505757 | -1.861908 |
| 1 | 4.762195 | 0.455090 | -2.559913 |
| 1 | 8.973474 | -1.783843 | -3.913778 |
| 1 | 5.896116 | 1.142394 | -4.634207 |
| 1 | 8.033044 | 0.046676 | -5.309016 |
| 1 | 8.344007 | -1.221992 | -0.098775 |
| 1 | 4.780617 | -1.726221 | 2.272380 |
| 1 | 9.708378 | -1.297291 | 1.942951 |
| 1 | 6.157841 | -1.803294 | 4.319836 |
| 1 | 8.635612 | -1.597360 | 4.165168 |
| 1 | 6.376675 | 2.052924 | 2.689360 |
| 1 | 6.982616 | 1.832247 | -1.573922 |
| 1 | 8.808801 | 2.017775 | 3.029493 |
| 1 | 9.410442 | 1.798421 | -1.226019 |
| 1 | 10.351982 | 1.908524 | 1.079538 |
| 1 | 2.734921 | 1.804208 | -1.914030 |
| 1 | 6.069472 | 4.350255 | -0.860420 |
| 1 | 2.476108 | 3.006865 | -4.011468 |
| 1 | 5.782844 | 5.573064 | -2.981099 |
| 1 | 4.019429 | 4.885062 | -4.595612 |
| 1 | 1.479437 | -0.816934 | 1.962959 |
| 6 | 1.539563 | 0.791896 | 0.519416 |
| 6 | 1.802439 | -3.031063 | 0.259523 |
| 6 | 0.509326 | -3.828019 | -0.158698 |
| 1 | 2.639916 | -3.472279 | -0.282075 |
| 1 | 1.989120 | -3.176867 | 1.328117 |
| 6 | -0.554566 | -3.732499 | 0.980517 |

| | | | |
|---|---|---|---|
| 1 | -0.232168 | -4.405507 | 1.775667 |
| 1 | -1.518926 | -4.095649 | 0.611610 |
| 6 | 0.787487 | -5.339553 | -0.272663 |
| 6 | 1.796425 | -5.968725 | 0.468972 |
| 6 | -0.062183 | -6.145274 | -1.047719 |
| 6 | 1.964506 | -7.354600 | 0.422868 |
| 1 | 2.468329 | -5.383200 | 1.085981 |
| 6 | 0.101306 | -7.529973 | -1.092855 |
| 1 | -0.859116 | -5.680747 | -1.618450 |
| 6 | 1.119716 | -8.142759 | -0.359218 |
| 1 | 2.760249 | -7.816144 | 1.001487 |
| 1 | -0.569113 | -8.129411 | -1.703263 |
| 1 | 1.250717 | -9.220814 | -0.394414 |
| 6 | 0.028241 | -3.285087 | -1.512591 |
| 6 | 0.871363 | -3.423220 | -2.630565 |
| 6 | -1.175589 | -2.598540 | -1.676946 |
| 6 | 0.533377 | -2.865763 | -3.861954 |
| 1 | 1.801589 | -3.971236 | -2.525209 |
| 6 | -1.516307 | -2.027355 | -2.907672 |
| 1 | -1.863876 | -2.467416 | -0.855850 |
| 6 | -0.666766 | -2.155694 | -4.004813 |
| 1 | 1.202369 | -2.984841 | -4.710497 |
| 1 | -2.449480 | -1.483320 | -2.994606 |
| 1 | -0.933495 | -1.710285 | -4.958984 |
| 7 | -0.653951 | -2.400650 | 1.563272 |
| 1 | -1.117252 | -1.649224 | 1.020081 |
| 16 | -0.879225 | -2.177452 | 3.183500 |
| 8 | -0.290987 | -3.328839 | 3.890717 |
| 1 | 1.222631 | 1.062721 | -0.482367 |
| 1 | 1.464055 | 1.569289 | 1.269290 |
| 8 | -0.370515 | -0.831945 | 3.495908 |
| 8 | 0.901111 | 0.574444 | -2.724165 |
| 1 | 0.056665 | 0.896158 | -2.329629 |
| 6 | 4.102302 | 2.918776 | 1.747836 |
| 6 | 4.287571 | 4.306375 | 1.838041 |
| 6 | 3.557984 | 2.230203 | 2.842193 |
| 6 | 3.918188 | 4.992440 | 2.995341 |
| 1 | 4.708534 | 4.857694 | 1.004154 |
| 6 | 3.199505 | 2.916570 | 4.004501 |
| 1 | 3.404824 | 1.156816 | 2.784491 |
| 6 | 3.375416 | 4.299617 | 4.081309 |
| 1 | 4.048962 | 6.069312 | 3.046152 |
| 1 | 2.769914 | 2.371016 | 4.839131 |
| 1 | 3.083723 | 4.836386 | 4.979156 |
| 1 | 0.670393 | -0.308582 | -3.041907 |
| 6 | -2.671964 | -2.115737 | 3.414941 |
| 6 | -3.483758 | -3.273298 | 3.655901 |
| 6 | -3.233299 | -0.879029 | 3.163528 |
| 6 | -2.990502 | -4.582103 | 3.928190 |
| 6 | -4.911338 | -3.089567 | 3.604643 |
| 6 | -4.634007 | -0.718805 | 3.146973 |
| 1 | -2.598362 | -0.031885 | 2.939792 |
| 6 | -3.854502 | -5.641050 | 4.109664 |
| 1 | -1.923245 | -4.739833 | 3.995654 |
| 6 | -5.767456 | -4.208835 | 3.788176 |
| 6 | -5.454501 | -1.802317 | 3.349501 |
| 1 | -5.050448 | 0.258225 | 2.935149 |
| 6 | -5.255327 | -5.461623 | 4.032529 |
| 1 | -3.450032 | -6.628821 | 4.312166 |
| 1 | -6.840612 | -4.050366 | 3.721522 |
| 1 | -6.533704 | -1.691725 | 3.297316 |
| 1 | -5.919819 | -6.310194 | 4.166841 |

| | | | |
|---|---|---|---|
| 6 | -3.504522 | 3.046269 | 0.411901 |
| 6 | -2.933567 | 4.303879 | 0.332661 |
| 6 | -3.786312 | 5.454651 | 0.298247 |
| 6 | -3.275608 | 6.782404 | 0.287559 |
| 1 | -2.201816 | 6.930755 | 0.309733 |
| 6 | -4.124383 | 7.867061 | 0.254117 |
| 1 | -3.713947 | 8.873010 | 0.250390 |
| 6 | -5.528560 | 7.683920 | 0.222678 |
| 1 | -6.185798 | 8.548376 | 0.192020 |
| 6 | -6.055305 | 6.412460 | 0.233501 |
| 1 | -7.131575 | 6.259534 | 0.212326 |
| 6 | -5.207277 | 5.271895 | 0.277734 |
| 6 | -5.733863 | 3.957682 | 0.284408 |
| 1 | -6.809546 | 3.826655 | 0.208712 |
| 6 | -4.916990 | 2.844116 | 0.360813 |
| 6 | -5.513135 | 1.486652 | 0.354926 |
| 6 | -6.671599 | 1.226587 | 1.062429 |
| 1 | -7.075114 | 1.989147 | 1.722680 |
| 6 | -7.349562 | -0.010952 | 0.956292 |
| 6 | -8.516852 | -0.279637 | 1.722898 |
| 1 | -8.870015 | 0.481405 | 2.414434 |
| 6 | -9.187595 | -1.474869 | 1.594141 |
| 1 | -10.075991 | -1.672248 | 2.187281 |
| 6 | -8.723234 | -2.444829 | 0.672710 |
| 1 | -9.262164 | -3.381470 | 0.560991 |
| 6 | -7.597027 | -2.212303 | -0.086439 |
| 1 | -7.257135 | -2.965292 | -0.786935 |
| 6 | -6.857823 | -1.003272 | 0.045645 |
| 6 | -5.648329 | -0.747150 | -0.682962 |
| 6 | -4.977985 | 0.447450 | -0.471287 |
| 6 | -1.449882 | 4.450685 | 0.237172 |
| 6 | -0.900193 | 4.837244 | -1.033080 |
| 6 | -1.699039 | 4.990061 | -2.201493 |
| 1 | -2.755013 | 4.751376 | -2.154096 |
| 6 | -1.148798 | 5.417874 | -3.389375 |
| 1 | -1.779118 | 5.519110 | -4.268603 |
| 6 | 0.230539 | 5.724045 | -3.474455 |
| 1 | 0.650890 | 6.076572 | -4.412639 |
| 6 | 1.036800 | 5.558440 | -2.371348 |
| 1 | 2.099752 | 5.769965 | -2.425647 |
| 6 | 0.503526 | 5.091428 | -1.141162 |
| 6 | 1.327824 | 4.861126 | -0.009863 |
| 1 | 2.398279 | 5.020726 | -0.097782 |
| 6 | 0.783224 | 4.434694 | 1.175547 |
| 1 | 1.433523 | 4.253921 | 2.023778 |
| 6 | -0.619118 | 4.246260 | 1.331773 |
| 6 | -5.172653 | -1.705306 | -1.732778 |
| 6 | -4.665304 | -2.990708 | -1.343033 |
| 6 | -4.482366 | -3.352174 | 0.021767 |
| 1 | -4.701387 | -2.624377 | 0.793447 |
| 6 | -4.021066 | -4.602754 | 0.369009 |
| 1 | -3.895439 | -4.858866 | 1.414358 |
| 6 | -3.690806 | -5.552764 | -0.628021 |
| 1 | -3.309950 | -6.527498 | -0.337315 |
| 6 | -3.832858 | -5.228441 | -1.957816 |
| 1 | -3.569749 | -5.943357 | -2.733780 |
| 6 | -4.324497 | -3.953747 | -2.348350 |
| 6 | -4.471950 | -3.598408 | -3.712432 |
| 1 | -4.207231 | -4.323376 | -4.477945 |
| 6 | -4.921322 | -2.349501 | -4.060576 |
| 1 | -5.008644 | -2.089685 | -5.111345 |
| 6 | -5.278487 | -1.376737 | -3.085342 |

| | | | |
|---|---|---|---|
| 8 | -1.446001 | 1.354333 | -1.562528 |
| 8 | -2.043304 | -0.416390 | 0.305834 |
| 8 | -2.696690 | 1.962317 | 0.658502 |
| 8 | -3.848558 | 0.706230 | -1.210652 |
| 15 | -2.344108 | 0.815600 | -0.493101 |
| 6 | -5.735751 | -0.011998 | -3.569328 |
| 6 | -4.597030 | 0.742505 | -4.295798 |
| 6 | -6.996431 | -0.068114 | -4.459345 |
| 1 | -6.004127 | 0.589041 | -2.697297 |
| 6 | -5.044778 | 2.156929 | -4.689033 |
| 1 | -4.312295 | 0.187174 | -5.201080 |
| 1 | -3.717718 | 0.789903 | -3.648301 |
| 6 | -7.444182 | 1.346823 | -4.859501 |
| 1 | -6.789233 | -0.652980 | -5.365917 |
| 1 | -7.802287 | -0.587625 | -3.926485 |
| 6 | -6.314404 | 2.123765 | -5.553042 |
| 1 | -4.234613 | 2.674257 | -5.217434 |
| 1 | -5.238090 | 2.733883 | -3.773374 |
| 1 | -8.326742 | 1.293794 | -5.509206 |
| 1 | -7.751323 | 1.891494 | -3.955224 |
| 1 | -6.645081 | 3.142898 | -5.789438 |
| 1 | -6.082211 | 1.638131 | -6.512256 |
| 6 | -1.155750 | 3.861138 | 2.697838 |
| 6 | -0.627892 | 2.491654 | 3.176497 |
| 6 | -0.865076 | 4.952209 | 3.754647 |
| 1 | -2.244398 | 3.773082 | 2.623572 |
| 6 | -1.212213 | 2.118268 | 4.545935 |
| 1 | 0.466650 | 2.531093 | 3.257528 |
| 1 | -0.867040 | 1.723998 | 2.436547 |
| 6 | -1.441633 | 4.573391 | 5.126999 |
| 1 | 0.221202 | 5.091619 | 3.844375 |
| 1 | -1.278427 | 5.910497 | 3.416162 |
| 6 | -0.927095 | 3.203936 | 5.592768 |
| 1 | -0.815548 | 1.149566 | 4.862124 |
| 1 | -2.300523 | 1.991413 | 4.448098 |
| 1 | -1.198441 | 5.348700 | 5.864859 |
| 1 | -2.538408 | 4.539426 | 5.057550 |
| 1 | -1.376322 | 2.936099 | 6.557405 |
| 1 | 0.158852 | 3.267054 | 5.759334 |

---

(*S*,*R*,*S*)-**2a**exo-*si*

---

Number of imaginary frequencies : 0
The smallest frequencies are : 11.5987 15.4681 17.0253 cm(-1)

Electronic energy : HF=-6614.0295753
Zero-point correction= 1.889283 (Hartree/Particle)
Thermal correction to Energy= 2.000357
Thermal correction to Enthalpy= 2.001301
Thermal correction to Gibbs Free Energy= 1.737967
Sum of electronic and zero-point Energies= -6612.140293
Sum of electronic and thermal Energies= -6612.029219
Sum of electronic and thermal Enthalpies= -6612.028274
Sum of electronic and thermal Free Energies= -6612.291608

........................................................

Cartesian Coordinates

........................................................

| | | | |
|---|---|---|---|
| 6 | -3.913722 | 0.461901 | 2.516027 |
| 6 | -5.160782 | 0.581513 | 1.878183 |
| 6 | -6.332993 | 0.464042 | 2.641670 |
| 6 | -6.255560 | 0.225128 | 4.014718 |

| | | | |
|---|---|---|---|
| 6 | -5.011715 | 0.106880 | 4.642239 |
| 6 | -3.841981 | 0.229009 | 3.890827 |
| 15 | -5.119547 | 0.851150 | 0.056237 |
| 6 | -6.746503 | 0.374120 | -0.667823 |
| 6 | -7.935881 | 1.020726 | -0.286720 |
| 6 | -9.144821 | 0.693918 | -0.897293 |
| 6 | -9.179941 | -0.261098 | -1.917653 |
| 6 | -8.000043 | -0.881368 | -2.326814 |
| 6 | -6.789778 | -0.563785 | -1.706773 |
| 46 | -3.221949 | -0.298922 | -0.803061 |
| 6 | -1.245559 | -0.842356 | -1.543409 |
| 6 | -1.453633 | 0.499303 | -1.946228 |
| 6 | -1.654430 | 1.491637 | -0.984897 |
| 15 | -3.748390 | -2.568486 | -0.312886 |
| 6 | -5.510686 | -3.044569 | -0.047959 |
| 6 | -6.160418 | -2.598148 | 1.114088 |
| 6 | -7.512877 | -2.865019 | 1.315240 |
| 6 | -8.235116 | -3.588785 | 0.363282 |
| 6 | -7.591606 | -4.053729 | -0.784047 |
| 6 | -6.237175 | -3.784174 | -0.991491 |
| 6 | -5.283294 | 2.679688 | -0.129388 |
| 6 | -5.381308 | 3.197766 | -1.432524 |
| 6 | -5.592386 | 4.559866 | -1.635900 |
| 6 | -5.692160 | 5.424600 | -0.540533 |
| 6 | -5.574408 | 4.919921 | 0.754193 |
| 6 | -5.376261 | 3.552184 | 0.961599 |
| 6 | -3.232951 | -3.756162 | -1.621519 |
| 6 | -2.897836 | -5.086565 | -1.330951 |
| 6 | -2.617888 | -5.982654 | -2.363817 |
| 6 | -2.682113 | -5.563712 | -3.695362 |
| 6 | -3.015599 | -4.240030 | -3.991911 |
| 6 | -3.279605 | -3.337923 | -2.959673 |
| 6 | -2.947395 | -3.122734 | 1.244790 |
| 6 | -1.800129 | -2.455417 | 1.691471 |
| 6 | -1.163841 | -2.842965 | 2.869932 |
| 6 | -1.679353 | -3.897160 | 3.622757 |
| 6 | -2.821974 | -4.575407 | 3.185921 |
| 6 | -3.454805 | -4.194442 | 2.003038 |
| 1 | -1.342662 | 1.267210 | 0.038177 |
| 1 | -0.684500 | -1.028620 | -0.633256 |
| 1 | -1.148608 | -1.614458 | -2.299784 |
| 1 | -5.302889 | 2.531632 | -2.287982 |
| 1 | -5.295797 | 3.169491 | 1.973168 |
| 1 | -5.666925 | 4.948415 | -2.647203 |
| 1 | -5.634896 | 5.587558 | 1.608490 |
| 1 | -5.848708 | 6.487478 | -0.699102 |
| 1 | -7.912900 | 1.803113 | 0.465326 |
| 1 | -5.875555 | -1.045489 | -2.034332 |
| 1 | -10.056703 | 1.197420 | -0.589139 |
| 1 | -8.016299 | -1.616174 | -3.126069 |
| 1 | -10.121773 | -0.508970 | -2.398775 |
| 1 | -7.306475 | 0.524305 | 2.169759 |
| 1 | -2.980993 | 0.536547 | 1.964435 |
| 1 | -7.169877 | 0.123508 | 4.592809 |
| 1 | -2.865360 | 0.125399 | 4.353477 |
| 1 | -4.957844 | -0.088754 | 5.709617 |
| 1 | -2.848658 | -5.423704 | -0.301111 |
| 1 | -3.516935 | -2.303027 | -3.192875 |
| 1 | -2.346067 | -7.007087 | -2.126735 |
| 1 | -3.058413 | -3.905920 | -5.024399 |
| 1 | -2.462476 | -6.262154 | -4.497379 |
| 1 | -5.608938 | -2.048134 | 1.867086 |

| | | | |
|---|---|---|---|
| 1 | -5.753881 | -4.149534 | -1.891136 |
| 1 | -7.999670 | -2.505030 | 2.216894 |
| 1 | -8.142868 | -4.626326 | -1.524588 |
| 1 | -9.290642 | -3.792873 | 0.517768 |
| 1 | -1.386386 | -1.628741 | 1.135480 |
| 1 | -4.347860 | -4.719809 | 1.681214 |
| 1 | -0.270153 | -2.314720 | 3.176585 |
| 1 | -3.223936 | -5.400812 | 3.766944 |
| 1 | -1.189410 | -4.191857 | 4.546160 |
| 1 | -1.598319 | 0.738676 | -2.998589 |
| 1 | 1.260713 | 1.480300 | -1.603404 |
| 6 | 0.600315 | 3.249752 | -0.689643 |
| 6 | -0.813683 | 3.915971 | -0.656611 |
| 1 | 0.638781 | 2.550499 | 0.147504 |
| 1 | 1.383614 | 3.986572 | -0.495058 |
| 7 | 0.906473 | 2.416990 | -1.847842 |
| 16 | 1.357849 | 2.927754 | -3.341146 |
| 8 | 0.405783 | 3.951764 | -3.795591 |
| 8 | 1.544249 | 1.704804 | -4.131211 |
| 6 | -1.845574 | 2.945839 | -1.333806 |
| 1 | -2.852042 | 3.269438 | -1.063903 |
| 1 | -1.748030 | 3.073024 | -2.414890 |
| 6 | -0.891980 | 5.291610 | -1.355836 |
| 6 | -2.148453 | 5.868897 | -1.593970 |
| 6 | 0.239066 | 6.050514 | -1.681868 |
| 6 | -2.271743 | 7.137290 | -2.160035 |
| 1 | -3.049079 | 5.332682 | -1.322284 |
| 6 | 0.122871 | 7.324199 | -2.241692 |
| 1 | 1.233511 | 5.655416 | -1.520693 |
| 6 | -1.133772 | 7.875286 | -2.490500 |
| 1 | -3.261828 | 7.550367 | -2.335551 |
| 1 | 1.023507 | 7.881090 | -2.487215 |
| 1 | -1.225203 | 8.864351 | -2.930718 |
| 6 | -1.102523 | 4.183024 | 0.840038 |
| 6 | -2.089861 | 3.506561 | 1.563548 |
| 6 | -0.323258 | 5.129430 | 1.530071 |
| 6 | -2.302354 | 3.764301 | 2.920487 |
| 1 | -2.707509 | 2.763696 | 1.082350 |
| 6 | -0.529197 | 5.391011 | 2.883953 |
| 1 | 0.437837 | 5.688896 | 0.995882 |
| 6 | -1.526193 | 4.709643 | 3.588723 |
| 1 | -3.072491 | 3.209554 | 3.449285 |
| 1 | 0.084395 | 6.134241 | 3.386062 |
| 1 | -1.694260 | 4.917201 | 4.641937 |
| 6 | 2.965343 | 3.745545 | -3.176120 |
| 6 | 4.122978 | 3.042722 | -2.704635 |
| 6 | 3.027423 | 5.073964 | -3.542237 |
| 6 | 4.128456 | 1.681098 | -2.296797 |
| 6 | 5.351007 | 3.786210 | -2.611936 |
| 6 | 4.244899 | 5.786921 | -3.449289 |
| 1 | 2.129277 | 5.565695 | -3.896868 |
| 6 | 5.275567 | 1.099295 | -1.801461 |
| 1 | 3.238889 | 1.074449 | -2.371514 |
| 6 | 6.518401 | 3.145177 | -2.115983 |
| 6 | 5.377534 | 5.155080 | -2.993246 |
| 1 | 4.273691 | 6.833060 | -3.738859 |
| 6 | 6.482225 | 1.829837 | -1.711271 |
| 1 | 5.244959 | 0.066487 | -1.472663 |
| 1 | 7.438723 | 3.720200 | -2.052679 |
| 1 | 6.315878 | 5.698023 | -2.912737 |
| 1 | 7.374722 | 1.349472 | -1.320444 |
| 6 | 3.983214 | -2.364792 | -0.515665 |

| | | | |
|---|---|---|---|
| 6 | 4.020296 | -2.857630 | -1.811356 |
| 6 | 5.295294 | -3.195192 | -2.375606 |
| 6 | 5.426183 | -3.811866 | -3.651280 |
| 1 | 4.529934 | -4.053895 | -4.210345 |
| 6 | 6.666195 | -4.100314 | -4.176089 |
| 1 | 6.741458 | -4.574524 | -5.150575 |
| 6 | 7.845465 | -3.783550 | -3.458162 |
| 1 | 8.817187 | -4.008860 | -3.888345 |
| 6 | 7.755410 | -3.199476 | -2.215888 |
| 1 | 8.653320 | -2.963707 | -1.649882 |
| 6 | 6.489424 | -2.905171 | -1.637046 |
| 6 | 6.386015 | -2.342599 | -0.341869 |
| 1 | 7.298232 | -2.110227 | 0.200224 |
| 6 | 5.160349 | -2.102198 | 0.253821 |
| 6 | 5.079043 | -1.654864 | 1.668028 |
| 6 | 5.946816 | -2.177572 | 2.610465 |
| 1 | 6.669076 | -2.931088 | 2.310306 |
| 6 | 5.911197 | -1.778341 | 3.968222 |
| 6 | 6.838313 | -2.290486 | 4.917430 |
| 1 | 7.592969 | -2.996053 | 4.578861 |
| 6 | 6.788754 | -1.898929 | 6.235797 |
| 1 | 7.504581 | -2.293560 | 6.951223 |
| 6 | 5.803310 | -0.975020 | 6.661758 |
| 1 | 5.772648 | -0.664199 | 7.702307 |
| 6 | 4.884296 | -0.468314 | 5.769218 |
| 1 | 4.133439 | 0.238787 | 6.103664 |
| 6 | 4.906655 | -0.852301 | 4.399627 |
| 6 | 3.966119 | -0.347761 | 3.443812 |
| 6 | 4.109024 | -0.701611 | 2.111762 |
| 6 | 2.757343 | -2.984192 | -2.609704 |
| 6 | 2.608117 | -2.141500 | -3.771561 |
| 6 | 3.587170 | -1.187236 | -4.168708 |
| 1 | 4.467953 | -1.042068 | -3.558258 |
| 6 | 3.430075 | -0.427849 | -5.305351 |
| 1 | 4.185275 | 0.307887 | -5.565044 |
| 6 | 2.282800 | -0.571837 | -6.117687 |
| 1 | 2.168088 | 0.040822 | -7.006948 |
| 6 | 1.302288 | -1.464131 | -5.754777 |
| 1 | 0.402414 | -1.573790 | -6.355842 |
| 6 | 1.432503 | -2.256593 | -4.582749 |
| 6 | 0.419672 | -3.166398 | -4.189307 |
| 1 | -0.480168 | -3.251379 | -4.792752 |
| 6 | 0.569957 | -3.933146 | -3.062731 |
| 1 | -0.223858 | -4.615724 | -2.781121 |
| 6 | 1.741950 | -3.870739 | -2.257088 |
| 6 | 2.806453 | 0.493442 | 3.880103 |
| 6 | 1.756903 | -0.151273 | 4.627975 |
| 6 | 1.771737 | -1.541979 | 4.933964 |
| 1 | 2.582097 | -2.156967 | 4.562448 |
| 6 | 0.771243 | -2.119692 | 5.685254 |
| 1 | 0.813277 | -3.182344 | 5.907346 |
| 6 | -0.316784 | -1.346897 | 6.156025 |
| 1 | -1.102986 | -1.816235 | 6.740442 |
| 6 | -0.377470 | -0.004522 | 5.857552 |
| 1 | -1.209779 | 0.603168 | 6.204786 |
| 6 | 0.644197 | 0.620963 | 5.093319 |
| 6 | 0.586652 | 2.000740 | 4.766572 |
| 1 | -0.266279 | 2.588481 | 5.093285 |
| 6 | 1.586857 | 2.584083 | 4.029583 |
| 1 | 1.509273 | 3.637328 | 3.781700 |
| 6 | 2.721955 | 1.848298 | 3.581259 |
| 8 | 1.826510 | 0.024834 | -0.831168 |

| | | | |
|---|---|---|---|
| 8 | 0.966718 | -1.088595 | 1.385877 |
| 8 | 2.760632 | -2.236107 | 0.099428 |
| 8 | 3.354343 | -0.041444 | 1.167990 |
| 15 | 2.070488 | -0.771146 | 0.413072 |
| 6 | 3.792331 | 2.584973 | 2.797129 |
| 6 | 3.219105 | 3.196403 | 1.501780 |
| 6 | 4.496244 | 3.664851 | 3.650587 |
| 1 | 4.560013 | 1.865218 | 2.497914 |
| 6 | 4.283613 | 3.959416 | 0.707381 |
| 1 | 2.395950 | 3.878196 | 1.751149 |
| 1 | 2.802608 | 2.393229 | 0.890268 |
| 6 | 5.573314 | 4.400667 | 2.839633 |
| 1 | 3.754434 | 4.391290 | 4.010762 |
| 1 | 4.936356 | 3.198523 | 4.540759 |
| 6 | 4.985249 | 5.021416 | 1.564180 |
| 1 | 3.829707 | 4.423065 | -0.175747 |
| 1 | 5.026046 | 3.247414 | 0.328081 |
| 1 | 6.050247 | 5.170518 | 3.459003 |
| 1 | 6.361566 | 3.686980 | 2.560624 |
| 1 | 5.771426 | 5.518462 | 0.982104 |
| 1 | 4.261152 | 5.800700 | 1.845541 |
| 6 | 1.826502 | -4.816646 | -1.070944 |
| 6 | 0.680319 | -4.588709 | -0.058965 |
| 6 | 1.856118 | -6.294053 | -1.528011 |
| 1 | 2.765689 | -4.633902 | -0.541582 |
| 6 | 0.784930 | -5.548721 | 1.133513 |
| 1 | -0.283334 | -4.748672 | -0.558108 |
| 1 | 0.694737 | -3.553976 | 0.289537 |
| 6 | 1.959638 | -7.250317 | -0.330729 |
| 1 | 0.941971 | -6.521387 | -2.093823 |
| 1 | 2.696394 | -6.448075 | -2.216268 |
| 6 | 0.823702 | -7.013239 | 0.675099 |
| 1 | -0.054488 | -5.381695 | 1.817393 |
| 1 | 1.699358 | -5.319180 | 1.699420 |
| 1 | 1.955928 | -8.291455 | -0.677881 |
| 1 | 2.923497 | -7.090710 | 0.173488 |
| 1 | 0.930919 | -7.685106 | 1.536195 |
| 1 | -0.135728 | -7.264737 | 0.198206 |
| 1 | -0.089694 | 0.134907 | 1.830315 |
| 8 | -0.770944 | 0.836293 | 1.969399 |
| 1 | -0.355600 | 1.502875 | 2.530089 |

---

(*S*,*R*,*S*)-**2a**exo-*re*

---

Number of imaginary frequencies : 0
The smallest frequencies are : 10.5573 11.8018 14.0344 cm(-1)

Electronic energy : HF=-6614.0276054
Zero-point correction= 1.889241 (Hartree/Particle)
Thermal correction to Energy= 2.000457
Thermal correction to Enthalpy= 2.001401
Thermal correction to Gibbs Free Energy= 1.735606
Sum of electronic and zero-point Energies= -6612.138365
Sum of electronic and thermal Energies= -6612.027148
Sum of electronic and thermal Enthalpies= -6612.026204
Sum of electronic and thermal Free Energies= -6612.291999

Cartesian Coordinates

| | | | |
|---|---|---|---|
| 6 | 6.778264 | -0.232343 | -1.229853 |
| 6 | 6.739164 | 0.385425 | 0.029914 |

| | | | |
|---|---|---|---|
| 6 | 7.908552 | 0.407904 | 0.803814 |
| 6 | 9.082477 | -0.178128 | 0.328272 |
| 6 | 9.108172 | -0.795518 | -0.924828 |
| 6 | 7.951982 | -0.817355 | -1.706403 |
| 15 | 5.118583 | 1.100644 | 0.578782 |
| 6 | 5.237424 | 1.278488 | 2.402566 |
| 6 | 6.158946 | 2.131100 | 3.040147 |
| 6 | 6.196515 | 2.216828 | 4.430874 |
| 6 | 5.315460 | 1.454155 | 5.204389 |
| 6 | 4.398191 | 0.609415 | 4.580468 |
| 6 | 4.355514 | 0.521874 | 3.186798 |
| 46 | 3.313989 | -0.182263 | -0.348790 |
| 6 | 1.752444 | -0.777672 | -1.729417 |
| 6 | 1.976447 | 0.600687 | -1.992526 |
| 6 | 1.702538 | 1.537410 | -1.000200 |
| 15 | 4.007794 | -2.411663 | 0.089823 |
| 6 | 5.014177 | -2.613342 | 1.624823 |
| 6 | 6.404849 | -2.423473 | 1.622071 |
| 6 | 7.128612 | -2.479383 | 2.814117 |
| 6 | 6.477092 | -2.723946 | 4.023459 |
| 6 | 5.094447 | -2.919496 | 4.034174 |
| 6 | 4.366710 | -2.865021 | 2.846115 |
| 6 | 5.278183 | 2.826233 | -0.079990 |
| 6 | 5.030780 | 3.971759 | 0.692194 |
| 6 | 5.107617 | 5.245493 | 0.123327 |
| 6 | 5.430104 | 5.396022 | -1.224896 |
| 6 | 5.664317 | 4.262657 | -2.007095 |
| 6 | 5.585167 | 2.990780 | -1.441741 |
| 6 | 2.660109 | -3.640914 | 0.387155 |
| 6 | 2.868748 | -5.021859 | 0.234276 |
| 6 | 1.856339 | -5.924415 | 0.558442 |
| 6 | 0.632456 | -5.465394 | 1.055490 |
| 6 | 0.420315 | -4.095421 | 1.209869 |
| 6 | 1.425899 | -3.188042 | 0.868090 |
| 6 | 5.023590 | -3.168286 | -1.249658 |
| 6 | 4.847200 | -2.702682 | -2.561693 |
| 6 | 5.562621 | -3.264897 | -3.619657 |
| 6 | 6.469612 | -4.299832 | -3.380661 |
| 6 | 6.655241 | -4.769597 | -2.078665 |
| 6 | 5.937322 | -4.210261 | -1.020346 |
| 1 | 1.093956 | 1.196407 | -0.168747 |
| 1 | 1.991299 | -1.499420 | -2.501662 |
| 1 | 0.927298 | -1.050508 | -1.077283 |
| 1 | 4.777060 | 3.879476 | 1.741356 |
| 1 | 5.771584 | 2.124344 | -2.067675 |
| 1 | 4.898263 | 6.117915 | 0.734750 |
| 1 | 5.908626 | 4.366274 | -3.060538 |
| 1 | 5.484342 | 6.386769 | -1.665604 |
| 1 | 6.835696 | 2.741257 | 2.450518 |
| 1 | 3.627401 | -0.125872 | 2.714158 |
| 1 | 6.910021 | 2.881090 | 4.910281 |
| 1 | 3.707560 | 0.015465 | 5.171543 |
| 1 | 5.344795 | 1.523885 | 6.288218 |
| 1 | 7.904971 | 0.846682 | 1.793956 |
| 1 | 5.875808 | -0.286480 | -1.828752 |
| 1 | 9.976405 | -0.161596 | 0.945576 |
| 1 | 7.949197 | -1.310813 | -2.673574 |
| 1 | 10.020730 | -1.261291 | -1.285700 |
| 1 | 3.811097 | -5.398087 | -0.148858 |
| 1 | 1.237146 | -2.132928 | 0.969821 |
| 1 | 2.021198 | -6.989051 | 0.418053 |
| 1 | -0.534407 | -3.719715 | 1.562747 |

| | | | |
|---|---|---|---|
| 1 | -0.157406 | -6.170741 | 1.292298 |
| 1 | 6.929671 | -2.238321 | 0.693111 |
| 1 | 3.292742 | -3.013810 | 2.873729 |
| 1 | 8.203766 | -2.327172 | 2.790638 |
| 1 | 4.576502 | -3.112601 | 4.969330 |
| 1 | 7.041549 | -2.763028 | 4.950752 |
| 1 | 4.154751 | -1.890846 | -2.752737 |
| 1 | 6.099948 | -4.580944 | -0.013824 |
| 1 | 5.413642 | -2.889290 | -4.627960 |
| 1 | 7.360255 | -5.572606 | -1.883468 |
| 1 | 7.029923 | -4.735454 | -4.202977 |
| 1 | 2.464078 | 0.921043 | -2.912107 |
| 1 | -1.394927 | 1.821648 | -1.293494 |
| 6 | -0.508813 | 3.789576 | -1.347233 |
| 6 | 0.774708 | 3.872559 | -0.461887 |
| 1 | -0.328622 | 4.447322 | -2.194780 |
| 1 | -1.373667 | 4.189502 | -0.807571 |
| 7 | -0.782752 | 2.451554 | -1.853385 |
| 16 | -0.937481 | 2.168930 | -3.470657 |
| 8 | -0.868022 | 0.715617 | -3.655505 |
| 8 | 0.025720 | 3.037216 | -4.174630 |
| 6 | 1.903442 | 3.023579 | -1.136144 |
| 1 | 1.965440 | 3.271415 | -2.200360 |
| 1 | 2.855383 | 3.311437 | -0.684868 |
| 6 | 0.576316 | 3.375492 | 0.976949 |
| 6 | 1.638822 | 3.516059 | 1.886650 |
| 6 | -0.596220 | 2.760768 | 1.424146 |
| 6 | 1.538227 | 3.052977 | 3.197908 |
| 1 | 2.548957 | 4.004580 | 1.559188 |
| 6 | -0.698181 | 2.280662 | 2.733976 |
| 1 | -1.445251 | 2.619140 | 0.770064 |
| 6 | 0.360606 | 2.428261 | 3.628045 |
| 1 | 2.378813 | 3.166879 | 3.876612 |
| 1 | -1.607107 | 1.777151 | 3.034975 |
| 1 | 0.279394 | 2.045589 | 4.641258 |
| 6 | 1.113910 | 5.374809 | -0.439278 |
| 6 | 2.024754 | 5.954728 | -1.332292 |
| 6 | 0.404371 | 6.225433 | 0.424876 |
| 6 | 2.235877 | 7.335988 | -1.346897 |
| 1 | 2.588906 | 5.335483 | -2.018708 |
| 6 | 0.609160 | 7.604664 | 0.408680 |
| 1 | -0.311631 | 5.798395 | 1.120174 |
| 6 | 1.531009 | 8.167985 | -0.476954 |
| 1 | 2.955448 | 7.758658 | -2.043197 |
| 1 | 0.048669 | 8.238861 | 1.090305 |
| 1 | 1.695970 | 9.241836 | -0.488977 |
| 6 | -2.629114 | 2.676760 | -3.894510 |
| 6 | -3.019149 | 4.049207 | -4.053253 |
| 6 | -3.561261 | 1.659134 | -3.901521 |
| 6 | -2.124490 | 5.155739 | -4.108762 |
| 6 | -4.427996 | 4.321353 | -4.170427 |
| 6 | -4.935607 | 1.946239 | -4.058679 |
| 1 | -3.231506 | 0.635589 | -3.770493 |
| 6 | -2.594314 | 6.447181 | -4.208100 |
| 1 | -1.059454 | 4.972559 | -4.099543 |
| 6 | -4.874588 | 5.666963 | -4.274244 |
| 6 | -5.358841 | 3.248346 | -4.175467 |
| 1 | -5.652573 | 1.132672 | -4.062616 |
| 6 | -3.981319 | 6.713036 | -4.278785 |
| 1 | -1.885893 | 7.269929 | -4.241592 |
| 1 | -5.943164 | 5.851388 | -4.349281 |
| 1 | -6.416938 | 3.478748 | -4.268994 |

| | | | |
|---|---|---|---|
| 1 | -4.335053 | 7.737350 | -4.351970 |
| 6 | -4.000940 | -0.930711 | 1.903083 |
| 6 | -4.878444 | -0.002451 | 2.434622 |
| 6 | -6.237845 | -0.386471 | 2.675961 |
| 6 | -7.205127 | 0.524949 | 3.181688 |
| 1 | -6.906438 | 1.547689 | 3.383729 |
| 6 | -8.504257 | 0.123897 | 3.407128 |
| 1 | -9.229853 | 0.836388 | 3.789344 |
| 6 | -8.904461 | -1.207915 | 3.140521 |
| 1 | -9.931972 | -1.509741 | 3.322650 |
| 6 | -7.992456 | -2.114171 | 2.648362 |
| 1 | -8.290241 | -3.137844 | 2.435195 |
| 6 | -6.645226 | -1.732293 | 2.404386 |
| 6 | -5.698151 | -2.646458 | 1.879999 |
| 1 | -6.023408 | -3.654594 | 1.639822 |
| 6 | -4.392437 | -2.275595 | 1.615387 |
| 6 | -3.490991 | -3.255112 | 0.951336 |
| 6 | -3.460645 | -4.583452 | 1.330115 |
| 1 | -3.935847 | -4.886011 | 2.259191 |
| 6 | -2.876857 | -5.574050 | 0.501232 |
| 6 | -2.837982 | -6.938490 | 0.900590 |
| 1 | -3.207880 | -7.200930 | 1.888672 |
| 6 | -2.353601 | -7.910301 | 0.054022 |
| 1 | -2.327855 | -8.949222 | 0.370216 |
| 6 | -1.903586 | -7.555531 | -1.240897 |
| 1 | -1.539391 | -8.327104 | -1.913390 |
| 6 | -1.926158 | -6.242459 | -1.657574 |
| 1 | -1.578938 | -5.984840 | -2.651013 |
| 6 | -2.381379 | -5.206185 | -0.795768 |
| 6 | -2.363932 | -3.823933 | -1.173068 |
| 6 | -2.824265 | -2.889171 | -0.257316 |
| 6 | -4.409519 | 1.363357 | 2.835449 |
| 6 | -4.405328 | 2.431439 | 1.878470 |
| 6 | -4.811438 | 2.249941 | 0.528130 |
| 1 | -5.142962 | 1.270257 | 0.206308 |
| 6 | -4.740530 | 3.281685 | -0.380364 |
| 1 | -5.026736 | 3.109880 | -1.412741 |
| 6 | -4.260542 | 4.554733 | 0.011488 |
| 1 | -4.193128 | 5.353972 | -0.721514 |
| 6 | -3.880122 | 4.774158 | 1.317534 |
| 1 | -3.516981 | 5.749602 | 1.632518 |
| 6 | -3.951726 | 3.731263 | 2.280588 |
| 6 | -3.565173 | 3.932528 | 3.630464 |
| 1 | -3.224710 | 4.918043 | 3.938483 |
| 6 | -3.595895 | 2.895970 | 4.530370 |
| 1 | -3.272574 | 3.069904 | 5.552019 |
| 6 | -4.006373 | 1.587928 | 4.149853 |
| 6 | -2.059871 | -3.366621 | -2.569982 |
| 6 | -0.732270 | -3.436386 | -3.115861 |
| 6 | 0.364280 | -4.006794 | -2.409421 |
| 1 | 0.214880 | -4.364391 | -1.400018 |
| 6 | 1.607623 | -4.122530 | -2.993030 |
| 1 | 2.419128 | -4.576836 | -2.436206 |
| 6 | 1.841295 | -3.636280 | -4.301034 |
| 1 | 2.830672 | -3.726018 | -4.739860 |
| 6 | 0.813636 | -3.048448 | -5.002704 |
| 1 | 0.975488 | -2.665169 | -6.007340 |
| 6 | -0.489234 | -2.950715 | -4.443893 |
| 6 | -1.565970 | -2.403835 | -5.184169 |
| 1 | -1.377145 | -2.015036 | -6.181377 |
| 6 | -2.831522 | -2.386562 | -4.657401 |
| 1 | -3.648974 | -1.988410 | -5.251102 |

| | | | |
|---|---|---|---|
| 6 | -3.108039 | -2.869658 | -3.349499 |
| 8 | -2.447168 | 0.878397 | -0.404980 |
| 8 | -0.562244 | -0.773926 | 0.405702 |
| 8 | -2.674407 | -0.592722 | 1.740303 |
| 8 | -2.835417 | -1.565123 | -0.622511 |
| 15 | -2.009101 | -0.409289 | 0.225963 |
| 6 | -4.553099 | -2.832940 | -2.874199 |
| 6 | -5.126729 | -1.400867 | -2.838834 |
| 6 | -5.469500 | -3.755472 | -3.709379 |
| 1 | -4.594947 | -3.212607 | -1.851501 |
| 6 | -6.557553 | -1.389247 | -2.283792 |
| 1 | -5.135957 | -0.995759 | -3.859419 |
| 1 | -4.475468 | -0.761337 | -2.238524 |
| 6 | -6.904918 | -3.745516 | -3.159061 |
| 1 | -5.476697 | -3.422833 | -4.756640 |
| 1 | -5.066227 | -4.775386 | -3.705702 |
| 6 | -7.476137 | -2.320825 | -3.088479 |
| 1 | -6.953282 | -0.365617 | -2.284801 |
| 1 | -6.537215 | -1.711415 | -1.233407 |
| 1 | -7.549367 | -4.384956 | -3.775138 |
| 1 | -6.902247 | -4.182254 | -2.149887 |
| 1 | -8.483369 | -2.336303 | -2.654054 |
| 1 | -7.580017 | -1.926739 | -4.110082 |
| 6 | -3.908740 | 0.452606 | 5.154924 |
| 6 | -2.436901 | 0.008847 | 5.332694 |
| 6 | -4.550808 | 0.762464 | 6.522536 |
| 1 | -4.445859 | -0.409515 | 4.746830 |
| 6 | -2.322436 | -1.184735 | 6.291006 |
| 1 | -1.854753 | 0.855068 | 5.725396 |
| 1 | -2.013481 | -0.243698 | 4.354398 |
| 6 | -4.442760 | -0.444253 | 7.467076 |
| 1 | -4.051150 | 1.621835 | 6.989394 |
| 1 | -5.600693 | 1.045925 | 6.381793 |
| 6 | -2.982970 | -0.887926 | 7.645430 |
| 1 | -1.268555 | -1.456269 | 6.428461 |
| 1 | -2.812706 | -2.055491 | 5.832706 |
| 1 | -4.889730 | -0.201949 | 8.439172 |
| 1 | -5.026237 | -1.278675 | 7.052498 |
| 1 | -2.929046 | -1.767739 | 8.298387 |
| 1 | -2.424000 | -0.086719 | 8.150895 |
| 1 | 0.668768 | -0.295339 | 1.515409 |
| 8 | 1.511412 | -0.145517 | 2.003334 |
| 1 | 1.492372 | 0.788254 | 2.248648 |

---

(*S*,*R*,*S*)-**2a**endo-*si*

---

Number of imaginary frequencies : 0
The smallest frequencies are : 3.0772 10.9972 16.1447 cm(-1)

Electronic energy : HF=-6614.0195857
Zero-point correction= 1.888622 (Hartree/Particle)
Thermal correction to Energy= 2.000251
Thermal correction to Enthalpy= 2.001196
Thermal correction to Gibbs Free Energy= 1.732860
Sum of electronic and zero-point Energies= -6612.130964
Sum of electronic and thermal Energies= -6612.019334
Sum of electronic and thermal Enthalpies= -6612.018390
Sum of electronic and thermal Free Energies= -6612.286726

............................................................
Cartesian Coordinates
............................................................

| | | | |
|---|---|---|---|
| 6 | 6.850472 | -0.643188 | -1.250804 |
| 6 | 6.880408 | 0.102166 | -0.062539 |
| 6 | 8.063218 | 0.122882 | 0.691073 |
| 6 | 9.181143 | -0.597134 | 0.267852 |
| 6 | 9.137515 | -1.343075 | -0.913126 |
| 6 | 7.969201 | -1.360473 | -1.676065 |
| 15 | 5.320680 | 0.971304 | 0.421599 |
| 6 | 5.448379 | 1.315986 | 2.218885 |
| 6 | 6.434974 | 2.151843 | 2.775391 |
| 6 | 6.477180 | 2.374421 | 4.150651 |
| 6 | 5.533882 | 1.770338 | 4.988014 |
| 6 | 4.548316 | 0.947665 | 4.443366 |
| 6 | 4.501595 | 0.722408 | 3.065532 |
| 46 | 3.380121 | -0.246307 | -0.268422 |
| 6 | 1.663459 | -0.876576 | -1.428561 |
| 6 | 1.453509 | 0.503140 | -1.189008 |
| 6 | 2.338117 | 1.452572 | -1.683422 |
| 15 | 3.919456 | -2.476894 | 0.388983 |
| 6 | 4.885984 | -2.562529 | 1.959314 |
| 6 | 6.278928 | -2.391522 | 1.975077 |
| 6 | 6.972617 | -2.337335 | 3.184607 |
| 6 | 6.287396 | -2.450206 | 4.394696 |
| 6 | 4.902260 | -2.627672 | 4.387696 |
| 6 | 4.204414 | -2.682647 | 3.181692 |
| 6 | 5.568810 | 2.614627 | -0.395013 |
| 6 | 5.359580 | 3.834201 | 0.266888 |
| 6 | 5.474251 | 5.046010 | -0.419416 |
| 6 | 5.799113 | 5.058856 | -1.775333 |
| 6 | 6.003632 | 3.850297 | -2.446165 |
| 6 | 5.886828 | 2.640214 | -1.763837 |
| 6 | 2.561182 | -3.686613 | 0.732057 |
| 6 | 2.743585 | -5.070520 | 0.566199 |
| 6 | 1.727990 | -5.959445 | 0.918581 |
| 6 | 0.524863 | -5.486226 | 1.450311 |
| 6 | 0.338006 | -4.114076 | 1.615399 |
| 6 | 1.347727 | -3.220768 | 1.251817 |
| 6 | 4.934847 | -3.355590 | -0.876578 |
| 6 | 4.700318 | -3.061438 | -2.229583 |
| 6 | 5.402531 | -3.725314 | -3.235894 |
| 6 | 6.354863 | -4.692149 | -2.904077 |
| 6 | 6.591689 | -4.996839 | -1.562444 |
| 6 | 5.883948 | -4.338497 | -0.554940 |
| 1 | 3.004985 | 1.124534 | -2.482350 |
| 1 | 0.964624 | -1.575519 | -0.989813 |
| 1 | 2.108467 | -1.187148 | -2.370745 |
| 1 | 5.105083 | 3.849802 | 1.320093 |
| 1 | 6.049354 | 1.713153 | -2.304344 |
| 1 | 5.292930 | 5.977456 | 0.108069 |
| 1 | 6.253286 | 3.847493 | -3.503355 |
| 1 | 5.884858 | 6.001468 | -2.307674 |
| 1 | 7.157149 | 2.645854 | 2.133051 |
| 1 | 3.723093 | 0.090402 | 2.654808 |
| 1 | 7.241837 | 3.023661 | 4.567526 |
| 1 | 3.807410 | 0.478304 | 5.083166 |
| 1 | 5.566703 | 1.947478 | 6.059368 |
| 1 | 8.110409 | 0.663042 | 1.628630 |
| 1 | 5.933849 | -0.692975 | -1.827984 |
| 1 | 10.085720 | -0.582511 | 0.869409 |
| 1 | 7.911478 | -1.951778 | -2.584623 |
| 1 | 10.006086 | -1.912778 | -1.230735 |
| 1 | 3.665967 | -5.461014 | 0.150824 |
| 1 | 1.180656 | -2.164883 | 1.365510 |

| | | | |
|---|---|---|---|
| 1 | 1.874585 | -7.025584 | 0.769328 |
| 1 | -0.602456 | -3.725138 | 1.991203 |
| 1 | -0.268921 | -6.179702 | 1.707986 |
| 1 | 6.829467 | -2.305738 | 1.047731 |
| 1 | 3.127866 | -2.810295 | 3.196915 |
| 1 | 8.050225 | -2.201508 | 3.173841 |
| 1 | 4.358363 | -2.719817 | 5.323500 |
| 1 | 6.827403 | -2.401318 | 5.335904 |
| 1 | 3.970383 | -2.305968 | -2.496975 |
| 1 | 6.077762 | -4.591480 | 0.481677 |
| 1 | 5.209106 | -3.481587 | -4.276668 |
| 1 | 7.327246 | -5.750259 | -1.295541 |
| 1 | 6.906761 | -5.205675 | -3.686086 |
| 1 | 0.685586 | 0.786557 | -0.483579 |
| 1 | -1.191685 | 1.721282 | -1.639441 |
| 6 | -0.196479 | 3.637145 | -1.804033 |
| 6 | 1.085365 | 3.707000 | -0.907455 |
| 1 | 0.026787 | 4.238348 | -2.684552 |
| 1 | -1.034591 | 4.118740 | -1.290725 |
| 7 | -0.588196 | 2.310213 | -2.245427 |
| 16 | -0.570745 | 1.832314 | -3.816005 |
| 8 | -0.256996 | 0.399023 | -3.853162 |
| 8 | 0.284499 | 2.775622 | -4.563061 |
| 6 | 2.263257 | 2.961916 | -1.605379 |
| 1 | 2.268073 | 3.282699 | -2.654844 |
| 1 | 3.207709 | 3.325130 | -1.192279 |
| 6 | 0.875291 | 3.235345 | 0.537283 |
| 6 | 1.943220 | 3.351484 | 1.443559 |
| 6 | -0.358763 | 2.800551 | 1.029771 |
| 6 | 1.780342 | 3.062684 | 2.798281 |
| 1 | 2.900551 | 3.711018 | 1.085816 |
| 6 | -0.523090 | 2.488016 | 2.382987 |
| 1 | -1.214676 | 2.682372 | 0.381502 |
| 6 | 0.536319 | 2.632163 | 3.276647 |
| 1 | 2.623665 | 3.161875 | 3.475383 |
| 1 | -1.486276 | 2.125771 | 2.717063 |
| 1 | 0.404415 | 2.388048 | 4.326531 |
| 6 | 1.371377 | 5.232071 | -0.853180 |
| 6 | 2.294496 | 5.869080 | -1.694109 |
| 6 | 0.597641 | 6.039058 | -0.000287 |
| 6 | 2.455731 | 7.257650 | -1.666333 |
| 1 | 2.907800 | 5.294229 | -2.376200 |
| 6 | 0.750541 | 7.424364 | 0.023093 |
| 1 | -0.126762 | 5.573783 | 0.660694 |
| 6 | 1.686817 | 8.043109 | -0.808510 |
| 1 | 3.186268 | 7.722394 | -2.323422 |
| 1 | 0.139058 | 8.019621 | 0.696010 |
| 1 | 1.812822 | 9.122060 | -0.788760 |
| 6 | -2.284191 | 1.990445 | -4.382777 |
| 6 | -2.912212 | 3.265355 | -4.575134 |
| 6 | -2.988020 | 0.813209 | -4.520395 |
| 6 | -2.248571 | 4.520338 | -4.487065 |
| 6 | -4.318375 | 3.267930 | -4.875403 |
| 6 | -4.358183 | 0.837528 | -4.866247 |
| 1 | -2.490344 | -0.134466 | -4.350673 |
| 6 | -2.939872 | 5.703207 | -4.636595 |
| 1 | -1.180126 | 4.543912 | -4.330242 |
| 6 | -4.998674 | 4.506802 | -5.021895 |
| 6 | -5.011379 | 2.035556 | -5.023226 |
| 1 | -4.887272 | -0.100443 | -4.990391 |
| 6 | -4.330058 | 5.702918 | -4.895210 |
| 1 | -2.406526 | 6.646431 | -4.560447 |

| | | | |
|---|---|---|---|
| 1 | -6.063902 | 4.489250 | -5.237281 |
| 1 | -6.071049 | 2.060377 | -5.263964 |
| 1 | -4.862173 | 6.643386 | -5.004569 |
| 6 | -4.025623 | -0.487132 | 2.026716 |
| 6 | -4.870346 | 0.528854 | 2.442929 |
| 6 | -6.193866 | 0.208363 | 2.880799 |
| 6 | -7.113718 | 1.201835 | 3.316317 |
| 1 | -6.806382 | 2.241581 | 3.300768 |
| 6 | -8.375513 | 0.855897 | 3.749048 |
| 1 | -9.063913 | 1.629572 | 4.077581 |
| 6 | -8.786598 | -0.499329 | 3.763512 |
| 1 | -9.784833 | -0.757589 | 4.105167 |
| 6 | -7.922798 | -1.484006 | 3.339642 |
| 1 | -8.230357 | -2.526777 | 3.340806 |
| 6 | -6.612700 | -1.161196 | 2.892402 |
| 6 | -5.717428 | -2.158750 | 2.433696 |
| 1 | -6.064080 | -3.187205 | 2.388990 |
| 6 | -4.438478 | -1.857983 | 1.998439 |
| 6 | -3.609005 | -2.950654 | 1.420668 |
| 6 | -3.580964 | -4.218232 | 1.972257 |
| 1 | -3.996575 | -4.380982 | 2.962881 |
| 6 | -3.070441 | -5.328343 | 1.252334 |
| 6 | -3.036641 | -6.629115 | 1.826179 |
| 1 | -3.353932 | -6.749535 | 2.859081 |
| 6 | -2.621590 | -7.717097 | 1.090656 |
| 1 | -2.601835 | -8.705565 | 1.540676 |
| 6 | -2.228131 | -7.548894 | -0.259572 |
| 1 | -1.909352 | -8.411418 | -0.837948 |
| 6 | -2.246119 | -6.301812 | -0.845255 |
| 1 | -1.938108 | -6.180730 | -1.877600 |
| 6 | -2.646137 | -5.153138 | -0.108659 |
| 6 | -2.639251 | -3.837889 | -0.671545 |
| 6 | -3.022221 | -2.776814 | 0.130873 |
| 6 | -4.380459 | 1.943841 | 2.447873 |
| 6 | -4.342795 | 2.670783 | 1.209835 |
| 6 | -4.800378 | 2.117111 | -0.016735 |
| 1 | -5.203448 | 1.111277 | -0.021416 |
| 6 | -4.705809 | 2.820438 | -1.195919 |
| 1 | -5.034029 | 2.364978 | -2.124860 |
| 6 | -4.144783 | 4.118927 | -1.215480 |
| 1 | -4.057660 | 4.653234 | -2.156158 |
| 6 | -3.711921 | 4.697478 | -0.041828 |
| 1 | -3.286635 | 5.698536 | -0.043659 |
| 6 | -3.809638 | 4.001248 | 1.193863 |
| 6 | -3.385985 | 4.581005 | 2.418191 |
| 1 | -2.984790 | 5.591737 | 2.410450 |
| 6 | -3.470399 | 3.877007 | 3.593676 |
| 1 | -3.129031 | 4.337465 | 4.516087 |
| 6 | -3.953294 | 2.538308 | 3.631231 |
| 6 | -2.435207 | -3.586064 | -2.137363 |
| 6 | -1.135286 | -3.669148 | -2.739824 |
| 6 | 0.026256 | -4.035205 | -2.005124 |
| 1 | -0.061687 | -4.239003 | -0.946558 |
| 6 | 1.256118 | -4.132624 | -2.619698 |
| 1 | 2.122614 | -4.422792 | -2.036672 |
| 6 | 1.400478 | -3.844864 | -3.997179 |
| 1 | 2.379025 | -3.919384 | -4.463812 |
| 6 | 0.300432 | -3.475077 | -4.737347 |
| 1 | 0.395460 | -3.246313 | -5.795956 |
| 6 | -0.985185 | -3.390842 | -4.138905 |
| 6 | -2.132933 | -3.043704 | -4.895618 |
| 1 | -2.020443 | -2.823457 | -5.954280 |

| | | | |
|---|---|---|---|
| 6 | -3.368625 | -2.992449 | -4.300014 |
| 1 | -4.237937 | -2.737376 | -4.899226 |
| 6 | -3.548589 | -3.259509 | -2.914762 |
| 8 | -2.188347 | 0.840065 | -0.603463 |
| 8 | -0.644587 | -0.941187 | 0.567778 |
| 8 | -2.721101 | -0.166461 | 1.742652 |
| 8 | -3.002021 | -1.512717 | -0.408507 |
| 15 | -1.999527 | -0.371555 | 0.256269 |
| 6 | -4.955240 | -3.162315 | -2.345149 |
| 6 | -5.520923 | -1.726778 | -2.428429 |
| 6 | -5.940978 | -4.159325 | -2.992762 |
| 1 | -4.917096 | -3.423302 | -1.285125 |
| 6 | -6.892823 | -1.634102 | -1.747190 |
| 1 | -5.631236 | -1.445646 | -3.483586 |
| 1 | -4.811829 | -1.025301 | -1.981570 |
| 6 | -7.321768 | -4.070100 | -2.321993 |
| 1 | -6.041598 | -3.942587 | -4.065248 |
| 1 | -5.543204 | -5.178216 | -2.912963 |
| 6 | -7.882123 | -2.639442 | -2.355663 |
| 1 | -7.285522 | -0.613016 | -1.829627 |
| 1 | -6.777372 | -1.837337 | -0.673348 |
| 1 | -8.020961 | -4.764631 | -2.804168 |
| 1 | -7.228966 | -4.394338 | -1.275457 |
| 1 | -8.844633 | -2.597345 | -1.830913 |
| 1 | -8.081262 | -2.357650 | -3.400116 |
| 6 | -3.929962 | 1.780451 | 4.948483 |
| 6 | -2.484119 | 1.516550 | 5.430283 |
| 6 | -4.753360 | 2.475487 | 6.053933 |
| 1 | -4.386833 | 0.799584 | 4.783543 |
| 6 | -2.464813 | 0.718403 | 6.741916 |
| 1 | -1.968896 | 2.476140 | 5.577212 |
| 1 | -1.934161 | 0.976149 | 4.652013 |
| 6 | -4.731928 | 1.665850 | 7.358591 |
| 1 | -4.346111 | 3.477528 | 6.246076 |
| 1 | -5.784246 | 2.613573 | 5.707846 |
| 6 | -3.294684 | 1.407613 | 7.834415 |
| 1 | -1.430540 | 0.574818 | 7.078445 |
| 1 | -2.875184 | -0.284086 | 6.555072 |
| 1 | -5.304065 | 2.188213 | 8.135318 |
| 1 | -5.235828 | 0.703533 | 7.190487 |
| 1 | -3.298188 | 0.803595 | 8.750118 |
| 1 | -2.823547 | 2.367428 | 8.092465 |
| 1 | 0.660993 | -0.335088 | 1.607944 |
| 8 | 1.491070 | -0.158961 | 2.106398 |
| 1 | 1.557785 | 0.803695 | 2.127035 |

---

(*S*,*S*,*S*)-**2a**endo-*re*

---

Number of imaginary frequencies : 0
The smallest frequencies are : 12.3367 14.5637 19.6888 cm(-1)

Electronic energy : HF=-6614.0214246
Zero-point correction= 1.890544 (Hartree/Particle)
Thermal correction to Energy= 2.001655
Thermal correction to Enthalpy= 2.002599
Thermal correction to Gibbs Free Energy= 1.738433
Sum of electronic and zero-point Energies= -6612.130881
Sum of electronic and thermal Energies= -6612.019769
Sum of electronic and thermal Enthalpies= -6612.018825
Sum of electronic and thermal Free Energies= -6612.282991
............................................................

Cartesian Coordinates

.............................................................

| | | | |
|---|---|---|---|
| 6 | -3.688730 | -4.874811 | -0.526675 |
| 6 | -3.739675 | -3.485530 | -0.320893 |
| 6 | -4.003255 | -2.998628 | 0.964253 |
| 6 | -4.241465 | -3.877207 | 2.022904 |
| 6 | -4.218790 | -5.254853 | 1.804246 |
| 6 | -3.942156 | -5.752141 | 0.526676 |
| 15 | -3.403198 | -2.309858 | -1.692844 |
| 6 | -4.772681 | -2.499441 | -2.907989 |
| 6 | -5.666717 | -3.577846 | -2.919079 |
| 6 | -6.704436 | -3.623234 | -3.852877 |
| 6 | -6.860171 | -2.595131 | -4.784178 |
| 6 | -5.968830 | -1.518517 | -4.784660 |
| 6 | -4.932948 | -1.470486 | -3.852548 |
| 46 | -3.147420 | -0.035797 | -1.080185 |
| 6 | -2.160423 | 2.019851 | -0.904801 |
| 6 | -1.207668 | 1.022150 | -1.173734 |
| 15 | -5.203953 | 0.571376 | -0.040436 |
| 6 | -6.177539 | 1.748216 | -1.071420 |
| 6 | -5.552956 | 2.455590 | -2.108548 |
| 6 | -6.267902 | 3.382832 | -2.868849 |
| 6 | -7.620462 | 3.605159 | -2.608349 |
| 6 | -8.258262 | 2.892190 | -1.589271 |
| 6 | -7.544913 | 1.967539 | -0.827059 |
| 6 | -1.948696 | -3.078424 | -2.520503 |
| 6 | -2.049139 | -3.767649 | -3.737044 |
| 6 | -0.902817 | -4.300618 | -4.334778 |
| 6 | 0.345828 | -4.136111 | -3.734111 |
| 6 | 0.452679 | -3.450033 | -2.521659 |
| 6 | -0.689642 | -2.937654 | -1.911422 |
| 6 | -4.984151 | 1.384912 | 1.593020 |
| 6 | -5.771246 | 2.454158 | 2.042657 |
| 6 | -5.569747 | 2.981471 | 3.320050 |
| 6 | -4.587308 | 2.448631 | 4.157234 |
| 6 | -3.789533 | 1.391524 | 3.711786 |
| 6 | -3.988362 | 0.865770 | 2.435436 |
| 6 | -6.448973 | -0.741284 | 0.321423 |
| 6 | -7.073002 | -1.386640 | -0.758057 |
| 6 | -7.975275 | -2.425127 | -0.533499 |
| 6 | -8.264930 | -2.834922 | 0.769998 |
| 6 | -7.655175 | -2.192099 | 1.848066 |
| 6 | -6.753828 | -1.148695 | 1.627887 |
| 1 | -2.603282 | 2.503628 | -1.772269 |
| 1 | -3.012382 | -3.886965 | -4.222982 |
| 1 | -0.588336 | -2.408303 | -0.969648 |
| 1 | -0.990290 | -4.834830 | -5.277004 |
| 1 | 1.417214 | -3.299309 | -2.053265 |
| 1 | 1.237058 | -4.540506 | -4.204230 |
| 1 | -5.576856 | -4.371359 | -2.186454 |
| 1 | -4.253537 | -0.622352 | -3.848212 |
| 1 | -7.395616 | -4.461362 | -3.844834 |
| 1 | -6.083481 | -0.713822 | -5.505047 |
| 1 | -7.672310 | -2.630281 | -5.504578 |
| 1 | -4.009442 | -1.931261 | 1.140630 |
| 1 | -3.431039 | -5.268549 | -1.505116 |
| 1 | -4.436712 | -3.481741 | 3.015065 |
| 1 | -3.906484 | -6.824063 | 0.354985 |
| 1 | -4.402878 | -5.940515 | 2.626117 |
| 1 | -6.534261 | 2.882263 | 1.401902 |
| 1 | -3.350266 | 0.062530 | 2.080558 |
| 1 | -6.177304 | 3.816559 | 3.656287 |

| | | | |
|---|---|---|---|
| 1 | -2.990776 | 1.005331 | 4.335175 |
| 1 | -4.426017 | 2.869917 | 5.144907 |
| 1 | -4.507964 | 2.279467 | -2.328647 |
| 1 | -8.052815 | 1.412552 | -0.044574 |
| 1 | -5.763341 | 3.926787 | -3.662023 |
| 1 | -9.313047 | 3.054799 | -1.387294 |
| 1 | -8.178299 | 4.325706 | -3.199494 |
| 1 | -6.859697 | -1.077602 | -1.775707 |
| 1 | -6.288228 | -0.659858 | 2.476639 |
| 1 | -8.443457 | -2.915405 | -1.381329 |
| 1 | -7.879268 | -2.499792 | 2.865447 |
| 1 | -8.963867 | -3.648051 | 0.943789 |
| 1 | -0.506877 | 0.693540 | -0.411452 |
| 6 | -1.346536 | 0.242468 | -2.329600 |
| 8 | 0.940053 | 2.174989 | -2.756897 |
| 6 | -2.167512 | 2.836055 | 0.358023 |
| 6 | -1.135299 | 4.010424 | 0.336887 |
| 1 | -1.950258 | 2.207905 | 1.223299 |
| 1 | -3.168305 | 3.247418 | 0.503123 |
| 6 | 0.312844 | 3.422386 | 0.472856 |
| 1 | 0.593056 | 2.902910 | -0.447539 |
| 1 | 1.022580 | 4.239937 | 0.619700 |
| 6 | -1.310169 | 4.788137 | -0.983189 |
| 6 | -2.561393 | 5.373056 | -1.252956 |
| 6 | -0.299853 | 4.932689 | -1.939726 |
| 6 | -2.801998 | 6.053299 | -2.444249 |
| 1 | -3.353060 | 5.302896 | -0.512213 |
| 6 | -0.535699 | 5.623559 | -3.133325 |
| 1 | 0.673214 | 4.490720 | -1.782978 |
| 6 | -1.784673 | 6.182003 | -3.396100 |
| 1 | -3.780548 | 6.490688 | -2.625544 |
| 1 | 0.265191 | 5.708404 | -3.862990 |
| 1 | -1.966140 | 6.714114 | -4.326016 |
| 6 | -1.392259 | 4.991535 | 1.496735 |
| 6 | -0.734971 | 6.233251 | 1.488152 |
| 6 | -2.232620 | 4.699349 | 2.573736 |
| 6 | -0.915089 | 7.152000 | 2.516817 |
| 1 | -0.077136 | 6.479811 | 0.659727 |
| 6 | -2.419415 | 5.621248 | 3.608162 |
| 1 | -2.732015 | 3.743491 | 2.639277 |
| 6 | -1.765063 | 6.849981 | 3.585704 |
| 1 | -0.393403 | 8.104969 | 2.486217 |
| 1 | -3.074456 | 5.364048 | 4.436337 |
| 1 | -1.908826 | 7.565571 | 4.390799 |
| 7 | 0.456197 | 2.429633 | 1.537442 |
| 1 | 0.693627 | 1.467821 | 1.271103 |
| 16 | 0.681415 | 2.813818 | 3.133202 |
| 8 | -0.514714 | 2.435953 | 3.911824 |
| 1 | 1.754518 | 2.653764 | -2.952237 |
| 1 | 1.239491 | 1.261583 | -2.556891 |
| 1 | -1.835527 | 0.658916 | -3.209978 |
| 1 | -0.648624 | -0.563083 | -2.511623 |
| 8 | 1.142869 | 4.209446 | 3.211650 |
| 6 | 2.038470 | 1.707354 | 3.609715 |
| 6 | 2.132846 | 1.100945 | 4.908316 |
| 6 | 3.021467 | 1.523657 | 2.660989 |
| 6 | 1.204208 | 1.278598 | 5.973531 |
| 6 | 3.257052 | 0.231169 | 5.139735 |
| 6 | 4.139271 | 0.708286 | 2.928003 |
| 1 | 2.932530 | 1.977863 | 1.684591 |
| 6 | 1.363609 | 0.616082 | 7.171540 |
| 1 | 0.357512 | 1.934234 | 5.829839 |

| | | | |
|---|---|---|---|
| 6 | 3.376030 | -0.448464 | 6.382391 |
| 6 | 4.242439 | 0.059100 | 4.132603 |
| 1 | 4.890327 | 0.576888 | 2.159865 |
| 6 | 2.448008 | -0.266596 | 7.380686 |
| 1 | 0.636892 | 0.770397 | 7.964285 |
| 1 | 4.218977 | -1.120344 | 6.522643 |
| 1 | 5.076199 | -0.607323 | 4.331239 |
| 1 | 2.546416 | -0.792487 | 8.325961 |
| 6 | 4.824919 | 0.284687 | -0.720714 |
| 6 | 5.434932 | 1.232038 | -1.528097 |
| 6 | 6.837258 | 1.126230 | -1.792512 |
| 6 | 7.559766 | 2.118693 | -2.511497 |
| 1 | 7.035051 | 3.001976 | -2.856646 |
| 6 | 8.905806 | 1.970095 | -2.765762 |
| 1 | 9.438771 | 2.742619 | -3.312753 |
| 6 | 9.602054 | 0.819733 | -2.320669 |
| 1 | 10.661108 | 0.712347 | -2.536620 |
| 6 | 8.937064 | -0.150818 | -1.606994 |
| 1 | 9.463445 | -1.031210 | -1.246847 |
| 6 | 7.552388 | -0.017776 | -1.310969 |
| 6 | 6.867313 | -0.979814 | -0.531750 |
| 1 | 7.414794 | -1.848866 | -0.178832 |
| 6 | 5.533512 | -0.836570 | -0.189358 |
| 6 | 4.917095 | -1.805010 | 0.754960 |
| 6 | 5.637616 | -2.227626 | 1.857763 |
| 1 | 6.603823 | -1.777327 | 2.065884 |
| 6 | 5.147837 | -3.218808 | 2.739204 |
| 6 | 5.878899 | -3.611657 | 3.894036 |
| 1 | 6.821661 | -3.113413 | 4.107179 |
| 6 | 5.408695 | -4.605396 | 4.721988 |
| 1 | 5.972910 | -4.896130 | 5.603503 |
| 6 | 4.192555 | -5.262467 | 4.413975 |
| 1 | 3.835105 | -6.059975 | 5.059211 |
| 6 | 3.460194 | -4.901838 | 3.303582 |
| 1 | 2.533720 | -5.415201 | 3.073563 |
| 6 | 3.896041 | -3.851227 | 2.448962 |
| 6 | 3.131593 | -3.407656 | 1.321224 |
| 6 | 3.621286 | -2.370528 | 0.543983 |
| 6 | 4.651081 | 2.376613 | -2.090936 |
| 6 | 4.314649 | 3.462783 | -1.205176 |
| 6 | 4.637017 | 3.442148 | 0.180515 |
| 1 | 5.189573 | 2.600177 | 0.580071 |
| 6 | 4.238793 | 4.455813 | 1.024791 |
| 1 | 4.464185 | 4.393992 | 2.084842 |
| 6 | 3.514225 | 5.565346 | 0.528722 |
| 1 | 3.185472 | 6.343587 | 1.210449 |
| 6 | 3.221152 | 5.641783 | -0.814242 |
| 1 | 2.668348 | 6.488436 | -1.213087 |
| 6 | 3.610893 | 4.605392 | -1.704900 |
| 6 | 3.299901 | 4.656026 | -3.088087 |
| 1 | 2.783402 | 5.526375 | -3.484425 |
| 6 | 3.621585 | 3.603614 | -3.907899 |
| 1 | 3.346089 | 3.645138 | -4.958572 |
| 6 | 4.277736 | 2.424506 | -3.433638 |
| 6 | 1.891591 | -4.153728 | 0.927749 |
| 6 | 2.030897 | -5.190312 | -0.059345 |
| 6 | 3.254094 | -5.442454 | -0.742377 |
| 1 | 4.125704 | -4.843787 | -0.504148 |
| 6 | 3.345296 | -6.423884 | -1.705578 |
| 1 | 4.289139 | -6.589446 | -2.217622 |
| 6 | 2.220076 | -7.216356 | -2.036044 |
| 1 | 2.304121 | -7.985275 | -2.798934 |

| | | | |
|---|---|---|---|
| 6 | 1.023466 | -7.005832 | -1.388861 |
| 1 | 0.148731 | -7.602328 | -1.636434 |
| 6 | 0.896820 | -5.998288 | -0.396379 |
| 6 | -0.335107 | -5.751482 | 0.259869 |
| 1 | -1.198883 | -6.358195 | 0.005469 |
| 6 | -0.447130 | -4.743807 | 1.182978 |
| 1 | -1.407806 | -4.560614 | 1.652840 |
| 6 | 0.662777 | -3.923195 | 1.536277 |
| 8 | 1.783638 | -0.329394 | -2.074385 |
| 8 | 1.279482 | -0.165950 | 0.498022 |
| 8 | 3.533893 | 0.513825 | -0.312571 |
| 8 | 2.895275 | -1.962638 | -0.554194 |
| 15 | 2.219104 | -0.446528 | -0.641878 |
| 6 | 4.464127 | 1.341402 | -4.501324 |
| 6 | 4.499704 | -0.134222 | -4.059493 |
| 6 | 5.662821 | 1.643136 | -5.435348 |
| 1 | 3.562975 | 1.431233 | -5.127346 |
| 6 | 4.468595 | -1.057657 | -5.286833 |
| 1 | 5.415624 | -0.343107 | -3.499922 |
| 1 | 3.653342 | -0.347881 | -3.403133 |
| 6 | 5.664490 | 0.707812 | -6.654272 |
| 1 | 6.594141 | 1.512764 | -4.872236 |
| 1 | 5.633861 | 2.690309 | -5.762031 |
| 6 | 5.642903 | -0.769112 | -6.233277 |
| 1 | 4.489624 | -2.104283 | -4.959245 |
| 1 | 3.520343 | -0.918592 | -5.826249 |
| 1 | 6.540138 | 0.914388 | -7.282493 |
| 1 | 4.778187 | 0.919976 | -7.269798 |
| 1 | 5.595910 | -1.415993 | -7.118289 |
| 1 | 6.583858 | -1.007520 | -5.716908 |
| 6 | 0.463081 | -2.819001 | 2.554146 |
| 6 | 0.172793 | -3.355684 | 3.975774 |
| 6 | -0.656282 | -1.838552 | 2.142702 |
| 1 | 1.388011 | -2.234119 | 2.601687 |
| 6 | 0.037921 | -2.208700 | 4.991065 |
| 1 | -0.757512 | -3.942034 | 3.952568 |
| 1 | 0.969845 | -4.038097 | 4.290070 |
| 6 | -0.695183 | -0.672204 | 3.129474 |
| 1 | -1.622264 | -2.359865 | 2.138703 |
| 1 | -0.464960 | -1.468406 | 1.135074 |
| 6 | -0.997676 | -1.162725 | 4.549385 |
| 1 | -0.217274 | -2.610357 | 5.979976 |
| 1 | 1.010635 | -1.713063 | 5.099460 |
| 1 | -1.405297 | 0.095623 | 2.817539 |
| 1 | 0.284600 | -0.187725 | 3.119552 |
| 1 | -0.999869 | -0.320307 | 5.249030 |
| 1 | -2.004720 | -1.608327 | 4.574345 |

---

(*S*,*S*,*S*)-**2a**exo-*si*

---

Number of imaginary frequencies : 0
The smallest frequencies are : 14.5457 16.1661 18.7971 cm(-1)

Electronic energy : HF=-6614.0240408
Zero-point correction= 1.890620 (Hartree/Particle)
Thermal correction to Energy= 2.001948
Thermal correction to Enthalpy= 2.002892
Thermal correction to Gibbs Free Energy= 1.739594
Sum of electronic and zero-point Energies= -6612.133421
Sum of electronic and thermal Energies= -6612.022093
Sum of electronic and thermal Enthalpies= -6612.021149

Sum of electronic and thermal Free Energies= -6612.284447

............................................................

Cartesian Coordinates

............................................................

| | | | |
|---|---|---|---|
| 6 | -4.244260 | 4.204326 | -0.148662 |
| 6 | -4.640577 | 2.915523 | -0.533081 |
| 6 | -5.108788 | 2.707321 | -1.843261 |
| 6 | -5.193956 | 3.771174 | -2.739401 |
| 6 | -4.793466 | 5.052617 | -2.349519 |
| 6 | -4.316427 | 5.264973 | -1.055532 |
| 15 | -4.456385 | 1.430582 | 0.541373 |
| 6 | -6.181569 | 0.953032 | 0.972475 |
| 6 | -7.303425 | 1.715200 | 0.622634 |
| 6 | -8.582312 | 1.290377 | 0.991168 |
| 6 | -8.752004 | 0.104560 | 1.707820 |
| 6 | -7.634300 | -0.651915 | 2.074016 |
| 6 | -6.357036 | -0.227458 | 1.711868 |
| 46 | -3.025348 | -0.100860 | -0.608444 |
| 6 | -1.107771 | 1.122843 | -0.257240 |
| 6 | -1.042681 | 0.414454 | -1.468812 |
| 15 | -4.511314 | -1.702627 | -1.552329 |
| 6 | -3.871082 | 2.049009 | 2.173649 |
| 6 | -2.672392 | 1.575296 | 2.719884 |
| 6 | -2.216397 | 2.048724 | 3.951816 |
| 6 | -2.962289 | 2.995646 | 4.652217 |
| 6 | -4.174599 | 3.456629 | 4.129267 |
| 6 | -4.632444 | 2.980965 | 2.901436 |
| 6 | -5.920318 | -0.893022 | -2.438258 |
| 6 | -5.752861 | -0.448430 | -3.761547 |
| 6 | -6.759656 | 0.274391 | -4.401330 |
| 6 | -7.951610 | 0.564981 | -3.733271 |
| 6 | -8.129348 | 0.121626 | -2.423097 |
| 6 | -7.122341 | -0.597431 | -1.776376 |
| 6 | -5.283150 | -2.848435 | -0.343051 |
| 6 | -4.614912 | -3.063036 | 0.871269 |
| 6 | -5.122005 | -3.955509 | 1.816285 |
| 6 | -6.313716 | -4.637147 | 1.560156 |
| 6 | -6.990202 | -4.427319 | 0.355404 |
| 6 | -6.476997 | -3.543487 | -0.595052 |
| 1 | -0.742714 | 0.558797 | 0.595990 |
| 1 | -2.087094 | 0.840608 | 2.181995 |
| 1 | -5.580073 | 3.335646 | 2.506597 |
| 1 | -1.280639 | 1.680802 | 4.355389 |
| 1 | -4.764518 | 4.185068 | 4.678273 |
| 1 | -2.599767 | 3.368512 | 5.605118 |
| 1 | -7.186820 | 2.629643 | 0.050930 |
| 1 | -5.496341 | -0.825073 | 1.992748 |
| 1 | -9.446328 | 1.886809 | 0.711867 |
| 1 | -7.755587 | -1.576525 | 2.630265 |
| 1 | -9.748681 | -0.227137 | 1.984290 |
| 1 | -5.407630 | 1.715494 | -2.165018 |
| 1 | -3.855789 | 4.385990 | 0.846282 |
| 1 | -5.562206 | 3.593323 | -3.745481 |
| 1 | -3.982576 | 6.251352 | -0.747603 |
| 1 | -4.844021 | 5.877725 | -3.054113 |
| 1 | -4.840674 | -0.665860 | -4.304649 |
| 1 | -7.282558 | -0.919991 | -0.755600 |
| 1 | -6.610688 | 0.606306 | -5.424783 |
| 1 | -9.048142 | 0.340398 | -1.888112 |
| 1 | -8.734765 | 1.128757 | -4.231686 |
| 1 | -3.702374 | -2.515275 | 1.082032 |
| 1 | -7.009019 | -3.392207 | -1.528745 |

| | | | |
|---|---|---|---|
| 1 | -4.589768 | -4.110913 | 2.750183 |
| 1 | -7.917179 | -4.955611 | 0.151936 |
| 1 | -6.714762 | -5.328641 | 2.295487 |
| 1 | -1.094795 | 0.921470 | -2.427981 |
| 6 | -1.157382 | -0.985060 | -1.388157 |
| 6 | -1.128886 | 2.614917 | -0.065287 |
| 6 | 0.119359 | 3.410673 | -0.559283 |
| 1 | -1.227232 | 2.775723 | 1.004832 |
| 1 | -2.000161 | 3.057111 | -0.544953 |
| 6 | 1.421567 | 2.746806 | -0.034480 |
| 1 | 1.611205 | 1.842458 | -0.605048 |
| 1 | 2.271808 | 3.412034 | -0.195372 |
| 6 | 0.082493 | 3.409501 | -2.103602 |
| 6 | -0.981403 | 4.076741 | -2.739804 |
| 6 | 0.993892 | 2.714006 | -2.908079 |
| 6 | -1.147256 | 4.028939 | -4.120886 |
| 1 | -1.694492 | 4.636448 | -2.141717 |
| 6 | 0.831595 | 2.669690 | -4.298161 |
| 1 | 1.825136 | 2.168076 | -2.482883 |
| 6 | -0.238485 | 3.316626 | -4.911561 |
| 1 | -1.985345 | 4.547586 | -4.579044 |
| 1 | 1.552880 | 2.115824 | -4.890999 |
| 1 | -0.363077 | 3.273776 | -5.990266 |
| 6 | 0.049974 | 4.863384 | -0.043406 |
| 6 | 0.914032 | 5.812847 | -0.611262 |
| 6 | -0.798583 | 5.289215 | 0.986302 |
| 6 | 0.929794 | 7.134072 | -0.173454 |
| 1 | 1.590326 | 5.507097 | -1.402483 |
| 6 | -0.790662 | 6.616904 | 1.425980 |
| 1 | -1.474121 | 4.596410 | 1.472539 |
| 6 | 0.071499 | 7.547137 | 0.849203 |
| 1 | 1.615109 | 7.842405 | -0.631437 |
| 1 | -1.461382 | 6.916443 | 2.227470 |
| 1 | 0.079490 | 8.578348 | 1.191681 |
| 7 | 1.323919 | 2.327115 | 1.380766 |
| 1 | 1.379270 | 1.300129 | 1.438570 |
| 16 | 2.372044 | 3.036121 | 2.477848 |
| 8 | 2.292273 | 4.488884 | 2.276574 |
| 1 | -0.819371 | -1.481320 | -0.481513 |
| 1 | -1.133544 | -1.589231 | -2.283196 |
| 8 | 3.704130 | 2.406397 | 2.502482 |
| 8 | 0.701684 | -1.979552 | -3.712649 |
| 1 | 1.139286 | -1.418441 | -3.037898 |
| 6 | -3.801886 | -2.817167 | -2.847887 |
| 6 | -2.919622 | -2.275163 | -3.797504 |
| 6 | -4.109570 | -4.184544 | -2.916211 |
| 6 | -2.351202 | -3.077570 | -4.785670 |
| 1 | -2.652530 | -1.224578 | -3.757848 |
| 6 | -3.547321 | -4.985608 | -3.912725 |
| 1 | -4.777071 | -4.636241 | -2.191567 |
| 6 | -2.666397 | -4.436520 | -4.846648 |
| 1 | -1.639038 | -2.642929 | -5.478709 |
| 1 | -3.792549 | -6.043257 | -3.949224 |
| 1 | -2.220111 | -5.065398 | -5.611600 |
| 1 | 0.987999 | -2.874466 | -3.488543 |
| 6 | 1.565712 | 2.611818 | 4.036108 |
| 6 | 1.637383 | 1.291742 | 4.588253 |
| 6 | 0.953630 | 3.650289 | 4.702659 |
| 6 | 2.234734 | 0.173430 | 3.940947 |
| 6 | 1.062699 | 1.097988 | 5.893639 |
| 6 | 0.378852 | 3.435609 | 5.976446 |
| 1 | 0.928422 | 4.632719 | 4.244342 |

| | | | |
|---|---|---|---|
| 6 | 2.286627 | -1.052774 | 4.565573 |
| 1 | 2.664614 | 0.274192 | 2.953941 |
| 6 | 1.140414 | -0.183073 | 6.505776 |
| 6 | 0.437443 | 2.189936 | 6.556844 |
| 1 | -0.095501 | 4.264033 | 6.493563 |
| 6 | 1.751229 | -1.234917 | 5.862263 |
| 1 | 2.743972 | -1.888934 | 4.051512 |
| 1 | 0.709616 | -0.312365 | 7.495335 |
| 1 | 0.009764 | 2.021631 | 7.541912 |
| 1 | 1.813102 | -2.210392 | 6.336029 |
| 6 | 4.904537 | -0.112033 | 0.017888 |
| 6 | 5.728628 | 0.663965 | -0.778658 |
| 6 | 7.145567 | 0.483679 | -0.703602 |
| 6 | 8.059752 | 1.304978 | -1.421354 |
| 1 | 7.669840 | 2.102227 | -2.044334 |
| 6 | 9.418902 | 1.101209 | -1.324853 |
| 1 | 10.100653 | 1.743824 | -1.874949 |
| 6 | 9.937797 | 0.060885 | -0.515098 |
| 1 | 11.011707 | -0.090326 | -0.452358 |
| 6 | 9.080222 | -0.753869 | 0.188115 |
| 1 | 9.466298 | -1.556816 | 0.811378 |
| 6 | 7.672936 | -0.562787 | 0.119570 |
| 6 | 6.775487 | -1.410138 | 0.810826 |
| 1 | 7.181267 | -2.259398 | 1.352802 |
| 6 | 5.405307 | -1.214910 | 0.785464 |
| 6 | 4.529538 | -2.206985 | 1.464430 |
| 6 | 4.890113 | -2.784060 | 2.669849 |
| 1 | 5.747621 | -2.397598 | 3.212571 |
| 6 | 4.176399 | -3.879596 | 3.215973 |
| 6 | 4.558631 | -4.468588 | 4.453393 |
| 1 | 5.401044 | -4.042028 | 4.992019 |
| 6 | 3.886064 | -5.558939 | 4.956298 |
| 1 | 4.188776 | -5.999012 | 5.902104 |
| 6 | 2.809112 | -6.123146 | 4.228620 |
| 1 | 2.301969 | -7.003003 | 4.614573 |
| 6 | 2.404980 | -5.567459 | 3.034653 |
| 1 | 1.588418 | -6.006125 | 2.472703 |
| 6 | 3.047147 | -4.413625 | 2.508742 |
| 6 | 2.606021 | -3.764360 | 1.313131 |
| 6 | 3.352515 | -2.701922 | 0.825668 |
| 6 | 5.151152 | 1.631854 | -1.767241 |
| 6 | 4.969466 | 3.003755 | -1.403941 |
| 6 | 5.156886 | 3.461520 | -0.071020 |
| 1 | 5.426453 | 2.750091 | 0.699120 |
| 6 | 4.923626 | 4.776293 | 0.265429 |
| 1 | 5.017988 | 5.089904 | 1.299314 |
| 6 | 4.519017 | 5.710316 | -0.718471 |
| 1 | 4.335313 | 6.742674 | -0.435635 |
| 6 | 4.334662 | 5.302826 | -2.020917 |
| 1 | 4.010007 | 6.008198 | -2.782458 |
| 6 | 4.539158 | 3.946759 | -2.394345 |
| 6 | 4.307788 | 3.486358 | -3.714360 |
| 1 | 3.989228 | 4.197562 | -4.472255 |
| 6 | 4.442445 | 2.154418 | -4.023664 |
| 1 | 4.228758 | 1.822927 | -5.034250 |
| 6 | 4.843138 | 1.192637 | -3.055570 |
| 6 | 1.389190 | -4.185114 | 0.544848 |
| 6 | 1.572633 | -4.690311 | -0.793620 |
| 6 | 2.858134 | -4.953886 | -1.345094 |
| 1 | 3.737054 | -4.807972 | -0.728122 |
| 6 | 3.008848 | -5.374909 | -2.647862 |
| 1 | 4.004069 | -5.557835 | -3.042244 |

| | | | |
|---|---|---|---|
| 6 | 1.879044 | -5.556682 | -3.481566 |
| 1 | 2.011602 | -5.867041 | -4.513976 |
| 6 | 0.616650 | -5.344093 | -2.973816 |
| 1 | -0.264218 | -5.485402 | -3.593924 |
| 6 | 0.433129 | -4.922434 | -1.627843 |
| 6 | -0.861843 | -4.713708 | -1.090511 |
| 1 | -1.730059 | -4.886616 | -1.718778 |
| 6 | -1.011645 | -4.294877 | 0.205549 |
| 1 | -2.013552 | -4.155871 | 0.596523 |
| 6 | 0.100726 | -4.003787 | 1.048400 |
| 8 | 1.933234 | -0.341587 | -1.798399 |
| 8 | 1.300882 | -0.508356 | 0.754757 |
| 8 | 3.586385 | 0.256285 | 0.113246 |
| 8 | 2.984858 | -2.148752 | -0.381237 |
| 15 | 2.288764 | -0.649411 | -0.373857 |
| 6 | -0.205231 | -3.494201 | 2.450591 |
| 6 | -0.974693 | -4.554045 | 3.275426 |
| 6 | -0.985555 | -2.157566 | 2.433150 |
| 1 | 0.736043 | -3.296371 | 2.970363 |
| 6 | -1.272353 | -4.059917 | 4.696582 |
| 1 | -1.923087 | -4.789422 | 2.772695 |
| 1 | -0.404375 | -5.487544 | 3.311232 |
| 6 | -1.340696 | -1.680663 | 3.848613 |
| 1 | -1.907199 | -2.281605 | 1.850096 |
| 1 | -0.375969 | -1.411093 | 1.918735 |
| 6 | -2.071952 | -2.751689 | 4.666745 |
| 1 | -1.813719 | -4.831980 | 5.257994 |
| 1 | -0.322951 | -3.892607 | 5.224166 |
| 1 | -1.950854 | -0.773838 | 3.796756 |
| 1 | -0.417961 | -1.405886 | 4.368462 |
| 1 | -2.255974 | -2.389191 | 5.685860 |
| 1 | -3.059824 | -2.943305 | 4.219967 |
| 6 | 4.912004 | -0.279811 | -3.432628 |
| 6 | 6.291909 | -0.707579 | -3.982052 |
| 6 | 3.813444 | -0.728769 | -4.416051 |
| 1 | 4.744677 | -0.850972 | -2.513926 |
| 6 | 6.346995 | -2.231782 | -4.174812 |
| 1 | 6.466881 | -0.200667 | -4.942553 |
| 1 | 7.089273 | -0.386056 | -3.306478 |
| 6 | 3.838565 | -2.254436 | -4.576300 |
| 1 | 3.976157 | -0.268297 | -5.401102 |
| 1 | 2.836372 | -0.409031 | -4.053928 |
| 6 | 5.208217 | -2.738679 | -5.074558 |
| 1 | 7.320420 | -2.526227 | -4.587322 |
| 1 | 6.266805 | -2.710149 | -3.187940 |
| 1 | 3.039353 | -2.571952 | -5.255170 |
| 1 | 3.614744 | -2.714127 | -3.605342 |
| 1 | 5.231949 | -3.834927 | -5.134045 |
| 1 | 5.368943 | -2.367099 | -6.097553 |

---

(*S*,*S*,*S*)-**2a**exo-*re*

---

Number of imaginary frequencies : 0
The smallest frequencies are : 15.2399 19.5429 21.8015 cm(-1)

Electronic energy : HF=-6614.0229857
Zero-point correction= 1.889226 (Hartree/Particle)
Thermal correction to Energy= 2.000656
Thermal correction to Enthalpy= 2.001600
Thermal correction to Gibbs Free Energy= 1.737741
Sum of electronic and zero-point Energies= -6612.133760

Sum of electronic and thermal Energies= -6612.022330
Sum of electronic and thermal Enthalpies= -6612.021386
Sum of electronic and thermal Free Energies= -6612.285244

.............................................................

Cartesian Coordinates

.............................................................

| | | | |
|---|---|---|---|
| 6 | 3.339830 | -0.529815 | 3.040298 |
| 6 | 4.565386 | 0.010831 | 2.614707 |
| 6 | 5.695354 | -0.145234 | 3.428935 |
| 6 | 5.591923 | -0.811680 | 4.650505 |
| 6 | 4.364528 | -1.325766 | 5.075385 |
| 6 | 3.236863 | -1.191324 | 4.264296 |
| 15 | 4.520819 | 0.972452 | 1.046160 |
| 6 | 6.207369 | 1.067947 | 0.308807 |
| 6 | 7.306221 | 1.588058 | 1.013866 |
| 6 | 8.562629 | 1.656937 | 0.413146 |
| 6 | 8.734158 | 1.240611 | -0.910533 |
| 6 | 7.638797 | 0.772604 | -1.637088 |
| 6 | 6.383982 | 0.690736 | -1.030028 |
| 46 | 2.998815 | -0.029686 | -0.470884 |
| 6 | 1.388262 | -0.541967 | -1.881052 |
| 6 | 1.504380 | 0.864464 | -1.866254 |
| 6 | 1.364112 | 1.553091 | -0.649319 |
| 15 | 3.992566 | -2.192186 | -0.590926 |
| 6 | 5.298176 | -2.664481 | 0.629922 |
| 6 | 4.962206 | -3.328111 | 1.820105 |
| 6 | 5.949609 | -3.647188 | 2.752257 |
| 6 | 7.282122 | -3.305642 | 2.515769 |
| 6 | 7.624359 | -2.639611 | 1.337911 |
| 6 | 6.640981 | -2.322319 | 0.401226 |
| 6 | 4.311583 | 2.703612 | 1.639916 |
| 6 | 4.763245 | 3.772509 | 0.847246 |
| 6 | 4.586811 | 5.087544 | 1.272721 |
| 6 | 3.948613 | 5.354621 | 2.486353 |
| 6 | 3.482799 | 4.299387 | 3.269938 |
| 6 | 3.665620 | 2.980145 | 2.852095 |
| 6 | 4.807219 | -2.532471 | -2.205453 |
| 6 | 5.658258 | -3.637121 | -2.388237 |
| 6 | 6.276614 | -3.856684 | -3.618626 |
| 6 | 6.071051 | -2.967246 | -4.677138 |
| 6 | 5.236225 | -1.862849 | -4.502916 |
| 6 | 4.603249 | -1.650469 | -3.276419 |
| 6 | 2.723000 | -3.491188 | -0.323770 |
| 6 | 1.713483 | -3.217846 | 0.609762 |
| 6 | 0.778557 | -4.195680 | 0.943864 |
| 6 | 0.815608 | -5.441820 | 0.318690 |
| 6 | 1.813085 | -5.718354 | -0.621280 |
| 6 | 2.773349 | -4.754764 | -0.930754 |
| 1 | 0.735740 | 1.070334 | 0.101565 |
| 1 | 0.714553 | -1.052764 | -1.200831 |
| 1 | 1.614088 | -1.085836 | -2.790940 |
| 1 | 5.265406 | 3.579399 | -0.095976 |
| 1 | 3.307228 | 2.170266 | 3.475541 |
| 1 | 4.942207 | 5.904789 | 0.651743 |
| 1 | 2.971175 | 4.497156 | 4.207018 |
| 1 | 3.799232 | 6.379343 | 2.808882 |
| 1 | 7.176636 | 1.962565 | 2.023733 |
| 1 | 5.537860 | 0.324944 | -1.600673 |
| 1 | 9.405799 | 2.048839 | 0.974583 |
| 1 | 7.754049 | 0.464959 | -2.672488 |
| 1 | 9.713569 | 1.298934 | -1.376529 |
| 1 | 6.661586 | 0.225501 | 3.111709 |

| | | | |
|---|---|---|---|
| 1 | 2.453157 | -0.440660 | 2.420592 |
| 1 | 6.478168 | -0.935583 | 5.266450 |
| 1 | 2.271834 | -1.597435 | 4.546315 |
| 1 | 4.291827 | -1.841114 | 6.029139 |
| 1 | 5.852153 | -4.316485 | -1.564496 |
| 1 | 3.950714 | -0.793396 | -3.150141 |
| 1 | 6.927816 | -4.716342 | -3.747166 |
| 1 | 5.070373 | -1.166746 | -5.319800 |
| 1 | 6.562597 | -3.133995 | -5.631283 |
| 1 | 3.933082 | -3.597621 | 2.027261 |
| 1 | 6.928245 | -1.817168 | -0.512403 |
| 1 | 5.670729 | -4.157701 | 3.668911 |
| 1 | 8.656783 | -2.365078 | 1.140922 |
| 1 | 8.047261 | -3.554265 | 3.245733 |
| 1 | 1.645460 | -2.250511 | 1.093811 |
| 1 | 3.547954 | -4.987450 | -1.652385 |
| 1 | 0.028826 | -3.955135 | 1.686366 |
| 1 | 1.844373 | -6.685010 | -1.115831 |
| 1 | 0.064959 | -6.191006 | 0.552354 |
| 1 | 1.908027 | 1.373608 | -2.738137 |
| 1 | -1.426557 | 1.631436 | -1.846907 |
| 6 | -0.881975 | 3.433708 | -0.974135 |
| 6 | 0.540619 | 4.070858 | -0.871152 |
| 1 | -1.075621 | 2.803754 | -0.103420 |
| 1 | -1.611367 | 4.246686 | -0.935595 |
| 7 | -1.119662 | 2.570600 | -2.138805 |
| 16 | -2.080518 | 3.052188 | -3.429309 |
| 8 | -1.267760 | 3.746506 | -4.434512 |
| 8 | -2.826526 | 1.851990 | -3.838824 |
| 6 | 1.629855 | 3.019336 | -0.411554 |
| 1 | 1.738031 | 3.128856 | 0.667780 |
| 1 | 2.595537 | 3.315863 | -0.823739 |
| 6 | 0.858796 | 4.737944 | -2.219415 |
| 6 | 1.837003 | 4.280125 | -3.106718 |
| 6 | 0.115466 | 5.868882 | -2.599068 |
| 6 | 2.069131 | 4.923349 | -4.326128 |
| 1 | 2.440165 | 3.414923 | -2.859621 |
| 6 | 0.346765 | 6.518605 | -3.807381 |
| 1 | -0.652015 | 6.250610 | -1.933519 |
| 6 | 1.327982 | 6.047269 | -4.682027 |
| 1 | 2.833660 | 4.538877 | -4.996200 |
| 1 | -0.243682 | 7.393163 | -4.067675 |
| 1 | 1.509008 | 6.549760 | -5.628112 |
| 6 | 0.519560 | 5.144262 | 0.244757 |
| 6 | -0.322881 | 5.026164 | 1.362198 |
| 6 | 1.399585 | 6.236155 | 0.205735 |
| 6 | -0.296118 | 5.970368 | 2.390920 |
| 1 | -1.012106 | 4.195813 | 1.448947 |
| 6 | 1.422028 | 7.185973 | 1.227182 |
| 1 | 2.068888 | 6.350798 | -0.639550 |
| 6 | 0.572326 | 7.060189 | 2.327348 |
| 1 | -0.964767 | 5.851566 | 3.239286 |
| 1 | 2.109752 | 8.025046 | 1.161289 |
| 1 | 0.586725 | 7.800542 | 3.122450 |
| 6 | -3.256032 | 4.269276 | -2.779315 |
| 6 | -4.188275 | 3.940922 | -1.742658 |
| 6 | -3.225218 | 5.522053 | -3.354427 |
| 6 | -4.250638 | 2.677675 | -1.093713 |
| 6 | -5.087165 | 4.977771 | -1.313616 |
| 6 | -4.127798 | 6.524216 | -2.931454 |
| 1 | -2.493645 | 5.728683 | -4.126144 |
| 6 | -5.149114 | 2.454516 | -0.072820 |

| | | | |
|---|---|---|---|
| 1 | -3.592112 | 1.875094 | -1.388652 |
| 6 | -6.012956 | 4.703226 | -0.270903 |
| 6 | -5.035225 | 6.255424 | -1.933565 |
| 1 | -4.093586 | 7.504209 | -3.397736 |
| 6 | -6.042636 | 3.470494 | 0.339567 |
| 1 | -5.167389 | 1.487813 | 0.419776 |
| 1 | -6.694827 | 5.491532 | 0.038283 |
| 1 | -5.727557 | 7.023791 | -1.598527 |
| 1 | -6.749629 | 3.266721 | 1.136201 |
| 6 | -2.037544 | -3.221111 | -0.610550 |
| 6 | -2.217913 | -3.683079 | -1.902214 |
| 6 | -2.781497 | -4.979795 | -2.108095 |
| 6 | -2.946047 | -5.537464 | -3.406088 |
| 1 | -2.632478 | -4.957416 | -4.267009 |
| 6 | -3.483277 | -6.795611 | -3.570580 |
| 1 | -3.594581 | -7.208188 | -4.569358 |
| 6 | -3.892924 | -7.557534 | -2.448481 |
| 1 | -4.316655 | -8.547166 | -2.593693 |
| 6 | -3.760949 | -7.039793 | -1.179771 |
| 1 | -4.082802 | -7.611951 | -0.313025 |
| 6 | -3.206214 | -5.746719 | -0.975296 |
| 6 | -3.120574 | -5.167160 | 0.313728 |
| 1 | -3.552122 | -5.704077 | 1.153481 |
| 6 | -2.559036 | -3.918698 | 0.522937 |
| 6 | -2.623603 | -3.315528 | 1.877904 |
| 6 | -2.430571 | -4.083353 | 3.013158 |
| 1 | -2.062952 | -5.100561 | 2.912129 |
| 6 | -2.727494 | -3.591845 | 4.309147 |
| 6 | -2.516966 | -4.382977 | 5.472749 |
| 1 | -2.069449 | -5.367032 | 5.358384 |
| 6 | -2.883663 | -3.920111 | 6.715560 |
| 1 | -2.720096 | -4.532701 | 7.597404 |
| 6 | -3.488503 | -2.645821 | 6.846456 |
| 1 | -3.793629 | -2.294791 | 7.828040 |
| 6 | -3.693265 | -1.849060 | 5.741388 |
| 1 | -4.157502 | -0.875355 | 5.848406 |
| 6 | -3.298968 | -2.282624 | 4.445272 |
| 6 | -3.437130 | -1.458342 | 3.283035 |
| 6 | -3.050683 | -1.960668 | 2.054330 |
| 6 | -1.953452 | -2.769068 | -3.056610 |
| 6 | -0.677941 | -2.756310 | -3.703737 |
| 6 | 0.385349 | -3.614573 | -3.307724 |
| 1 | 0.239674 | -4.278881 | -2.462600 |
| 6 | 1.588835 | -3.606881 | -3.979284 |
| 1 | 2.387795 | -4.270689 | -3.666799 |
| 6 | 1.801436 | -2.725550 | -5.067511 |
| 1 | 2.756711 | -2.730434 | -5.582452 |
| 6 | 0.801808 | -1.862489 | -5.458431 |
| 1 | 0.955594 | -1.176533 | -6.288233 |
| 6 | -0.457752 | -1.858960 | -4.800149 |
| 6 | -1.512913 | -1.002064 | -5.204287 |
| 1 | -1.342710 | -0.297243 | -6.013731 |
| 6 | -2.732955 | -1.054846 | -4.580040 |
| 1 | -3.518537 | -0.379874 | -4.896411 |
| 6 | -2.984750 | -1.944061 | -3.500242 |
| 6 | -4.085261 | -0.111546 | 3.378434 |
| 6 | -5.486071 | -0.024110 | 3.068897 |
| 6 | -6.234577 | -1.129022 | 2.573769 |
| 1 | -5.738927 | -2.082644 | 2.432451 |
| 6 | -7.569767 | -1.002498 | 2.257145 |
| 1 | -8.113672 | -1.857356 | 1.865514 |
| 6 | -8.238479 | 0.231484 | 2.437007 |

| | | | |
|---|---|---|---|
| 1 | -9.292048 | 0.317686 | 2.187091 |
| 6 | -7.549359 | 1.317106 | 2.929010 |
| 1 | -8.054028 | 2.268745 | 3.079043 |
| 6 | -6.166882 | 1.224501 | 3.245071 |
| 6 | -5.431677 | 2.342165 | 3.719202 |
| 1 | -5.946523 | 3.288999 | 3.861017 |
| 6 | -4.088263 | 2.236074 | 3.976019 |
| 1 | -3.542014 | 3.108254 | 4.321888 |
| 6 | -3.383219 | 1.011515 | 3.798798 |
| 8 | -2.102806 | 0.322737 | -0.836592 |
| 8 | -0.685835 | -0.317352 | 1.256192 |
| 8 | -1.264432 | -2.100448 | -0.426702 |
| 8 | -3.161289 | -1.140677 | 0.949470 |
| 15 | -1.739827 | -0.656568 | 0.231673 |
| 6 | -1.889878 | 0.969833 | 4.058882 |
| 6 | -1.535625 | 1.072605 | 5.558135 |
| 6 | -1.134645 | 2.054421 | 3.261367 |
| 1 | -1.513084 | 0.007823 | 3.704905 |
| 6 | -0.018657 | 0.947364 | 5.764024 |
| 1 | -1.888652 | 2.036112 | 5.954284 |
| 1 | -2.055625 | 0.288892 | 6.118797 |
| 6 | 0.379525 | 1.950255 | 3.474822 |
| 1 | -1.474695 | 3.050737 | 3.575598 |
| 1 | -1.370131 | 1.941939 | 2.199955 |
| 6 | 0.745125 | 2.012553 | 4.964272 |
| 1 | 0.226770 | 1.017652 | 6.831448 |
| 1 | 0.297962 | -0.046421 | 5.420777 |
| 1 | 0.885850 | 2.750917 | 2.924610 |
| 1 | 0.721172 | 0.998707 | 3.054055 |
| 1 | 1.825213 | 1.878596 | 5.102358 |
| 1 | 0.497273 | 3.010070 | 5.356977 |
| 6 | -4.382199 | -1.996175 | -2.901921 |
| 6 | -5.395458 | -2.598464 | -3.902596 |
| 6 | -4.883292 | -0.633097 | -2.379631 |
| 1 | -4.358596 | -2.668973 | -2.038984 |
| 6 | -6.790826 | -2.723552 | -3.272438 |
| 1 | -5.449815 | -1.956209 | -4.792760 |
| 1 | -5.042455 | -3.580252 | -4.240752 |
| 6 | -6.275721 | -0.764651 | -1.747697 |
| 1 | -4.916774 | 0.096623 | -3.197959 |
| 1 | -4.167458 | -0.247663 | -1.654065 |
| 6 | -7.287873 | -1.376079 | -2.726996 |
| 1 | -7.500638 | -3.126321 | -4.006323 |
| 1 | -6.744085 | -3.451561 | -2.449476 |
| 1 | -6.620133 | 0.217905 | -1.403851 |
| 1 | -6.205824 | -1.397150 | -0.850828 |
| 1 | -8.266190 | -1.498394 | -2.244462 |
| 1 | -7.435661 | -0.682590 | -3.567513 |
| 1 | -0.086474 | -1.295830 | 2.529586 |
| 8 | 0.283984 | -1.838033 | 3.265379 |
| 1 | -0.485823 | -2.272100 | 3.652298 |

---

(*S*,*S*,*S*)-**2a**endo-*si*

---

Number of imaginary frequencies : 0
The smallest frequencies are : 13.2659 13.9570 17.4557 cm(-1)

Electronic energy : HF=-6614.0243374
Zero-point correction= 1.889596 (Hartree/Particle)
Thermal correction to Energy= 2.001137
Thermal correction to Enthalpy= 2.002081

Thermal correction to Gibbs Free Energy= 1.736526
Sum of electronic and zero-point Energies= -6612.134742
Sum of electronic and thermal Energies= -6612.023200
Sum of electronic and thermal Enthalpies= -6612.022256
Sum of electronic and thermal Free Energies= -6612.287812

............................................................

Cartesian Coordinates

............................................................

| | | | |
|---|---|---|---|
| 6 | -3.405205 | 0.101125 | 2.732243 |
| 6 | -4.624980 | -0.444034 | 2.299222 |
| 6 | -5.720456 | -0.442943 | 3.174306 |
| 6 | -5.590976 | 0.090028 | 4.457106 |
| 6 | -4.370804 | 0.624631 | 4.881278 |
| 6 | -3.274619 | 0.631292 | 4.016576 |
| 15 | -4.646360 | -1.169809 | 0.609476 |
| 6 | -6.379766 | -1.188977 | -0.018578 |
| 6 | -7.390758 | -1.930783 | 0.618365 |
| 6 | -8.683538 | -1.954945 | 0.098236 |
| 6 | -8.980079 | -1.266219 | -1.081905 |
| 6 | -7.975741 | -0.559651 | -1.742283 |
| 6 | -6.685218 | -0.520067 | -1.210707 |
| 46 | -3.157921 | 0.087459 | -0.736109 |
| 6 | -1.615982 | 0.853174 | -2.119397 |
| 6 | -1.051456 | -0.178758 | -1.350716 |
| 6 | -1.581706 | -1.477953 | -1.391934 |
| 15 | -4.147074 | 2.252060 | -0.526780 |
| 6 | -5.715766 | 2.506475 | 0.412064 |
| 6 | -5.680006 | 2.645624 | 1.808820 |
| 6 | -6.862457 | 2.791714 | 2.533891 |
| 6 | -8.095500 | 2.790502 | 1.879453 |
| 6 | -8.140212 | 2.650144 | 0.491276 |
| 6 | -6.959643 | 2.514298 | -0.238661 |
| 6 | -4.415626 | -2.974129 | 0.922899 |
| 6 | -4.662673 | -3.873245 | -0.128597 |
| 6 | -4.567945 | -5.248005 | 0.079334 |
| 6 | -4.224403 | -5.743248 | 1.341526 |
| 6 | -3.956370 | -4.856076 | 2.384195 |
| 6 | -4.049523 | -3.476869 | 2.178463 |
| 6 | -4.584322 | 2.942085 | -2.183580 |
| 6 | -4.953351 | 4.282561 | -2.379864 |
| 6 | -5.362338 | 4.728413 | -3.636143 |
| 6 | -5.421093 | 3.840827 | -4.715004 |
| 6 | -5.061411 | 2.505620 | -4.530956 |
| 6 | -4.643882 | 2.062373 | -3.274080 |
| 6 | -2.998994 | 3.404289 | 0.334763 |
| 6 | -1.746226 | 2.937461 | 0.752822 |
| 6 | -0.874148 | 3.770911 | 1.456123 |
| 6 | -1.233201 | 5.088985 | 1.725821 |
| 6 | -2.487731 | 5.564026 | 1.326595 |
| 6 | -3.372862 | 4.723270 | 0.655888 |
| 1 | -2.072785 | -1.788002 | -2.311916 |
| 1 | -2.076938 | 0.626873 | -3.078176 |
| 1 | -1.259102 | 1.869876 | -2.003154 |
| 1 | -4.951531 | -3.498161 | -1.106903 |
| 1 | -3.847646 | -2.799890 | 3.000391 |
| 1 | -4.757983 | -5.931195 | -0.742741 |
| 1 | -3.670643 | -5.229658 | 3.362888 |
| 1 | -4.153472 | -6.814701 | 1.503689 |
| 1 | -7.163922 | -2.513229 | 1.505458 |
| 1 | -5.910322 | 0.032971 | -1.728590 |
| 1 | -9.456571 | -2.524737 | 0.605672 |
| 1 | -8.191414 | -0.033311 | -2.667767 |

| | | | |
|---|---|---|---|
| 1 | -9.986407 | -1.294245 | -1.489729 |
| 1 | -6.681637 | -0.826615 | 2.855165 |
| 1 | -2.545338 | 0.119818 | 2.070484 |
| 1 | -6.448887 | 0.093464 | 5.123665 |
| 1 | -2.310880 | 1.034940 | 4.307426 |
| 1 | -4.277345 | 1.037255 | 5.881984 |
| 1 | -4.926723 | 4.986042 | -1.557055 |
| 1 | -4.356693 | 1.024216 | -3.142638 |
| 1 | -5.636909 | 5.770401 | -3.771893 |
| 1 | -5.096135 | 1.808115 | -5.362714 |
| 1 | -5.741798 | 4.190673 | -5.691835 |
| 1 | -4.732175 | 2.635909 | 2.335688 |
| 1 | -7.012802 | 2.417916 | -1.317042 |
| 1 | -6.813928 | 2.894144 | 3.613514 |
| 1 | -9.093977 | 2.647910 | -0.028545 |
| 1 | -9.015462 | 2.900242 | 2.446743 |
| 1 | -1.440310 | 1.918475 | 0.552097 |
| 1 | -4.365919 | 5.087725 | 0.417038 |
| 1 | 0.067652 | 3.364128 | 1.796587 |
| 1 | -2.784580 | 6.583645 | 1.555715 |
| 1 | -0.547113 | 5.741615 | 2.258447 |
| 1 | -0.361883 | 0.061564 | -0.547068 |
| 1 | 1.543768 | -1.010987 | -2.198571 |
| 6 | 1.222117 | -2.902580 | -1.448408 |
| 6 | -0.086745 | -3.613019 | -1.024207 |
| 1 | 1.610543 | -2.358146 | -0.584976 |
| 1 | 1.969696 | -3.653821 | -1.702442 |
| 7 | 1.133634 | -1.898776 | -2.515319 |
| 16 | 1.636812 | -2.186942 | -4.084880 |
| 8 | 0.532553 | -2.778471 | -4.852806 |
| 8 | 2.224374 | -0.918293 | -4.546230 |
| 6 | -1.101834 | -2.560661 | -0.454229 |
| 1 | -0.622703 | -2.085373 | 0.405338 |
| 1 | -1.967805 | -3.100214 | -0.068064 |
| 6 | -0.656090 | -4.480860 | -2.162605 |
| 6 | -2.002238 | -4.459989 | -2.547436 |
| 6 | 0.183931 | -5.412954 | -2.793425 |
| 6 | -2.483062 | -5.305657 | -3.551867 |
| 1 | -2.703241 | -3.798578 | -2.055990 |
| 6 | -0.288527 | -6.257567 | -3.794321 |
| 1 | 1.222246 | -5.492937 | -2.490834 |
| 6 | -1.627879 | -6.203192 | -4.187669 |
| 1 | -3.532750 | -5.259662 | -3.831201 |
| 1 | 0.392373 | -6.960954 | -4.265914 |
| 1 | -1.999115 | -6.858259 | -4.970797 |
| 6 | 0.253159 | -4.595369 | 0.131715 |
| 6 | 1.554711 | -4.834346 | 0.597096 |
| 6 | -0.792295 | -5.321632 | 0.724322 |
| 6 | 1.796147 | -5.768343 | 1.610067 |
| 1 | 2.405554 | -4.296597 | 0.198614 |
| 6 | -0.558484 | -6.230969 | 1.752185 |
| 1 | -1.805371 | -5.187146 | 0.365108 |
| 6 | 0.744356 | -6.465192 | 2.200793 |
| 1 | 2.817109 | -5.932779 | 1.941929 |
| 1 | -1.396130 | -6.763950 | 2.193337 |
| 1 | 0.934219 | -7.182224 | 2.994707 |
| 6 | 2.938433 | -3.448593 | -4.004725 |
| 6 | 4.083588 | -3.314777 | -3.154458 |
| 6 | 2.755856 | -4.562818 | -4.796102 |
| 6 | 4.323266 | -2.200394 | -2.306391 |
| 6 | 5.007249 | -4.416137 | -3.111533 |
| 6 | 3.704934 | -5.609952 | -4.786998 |

| | | | |
|---|---|---|---|
| 1 | 1.861174 | -4.638331 | -5.402162 |
| 6 | 5.380085 | -2.199765 | -1.422837 |
| 1 | 3.668484 | -1.343638 | -2.325020 |
| 6 | 6.105499 | -4.365359 | -2.209806 |
| 6 | 4.797125 | -5.541350 | -3.953465 |
| 1 | 3.549375 | -6.475354 | -5.424028 |
| 6 | 6.280400 | -3.289461 | -1.369311 |
| 1 | 5.513401 | -1.352822 | -0.758286 |
| 1 | 6.796954 | -5.204079 | -2.187642 |
| 1 | 5.514984 | -6.356991 | -3.920192 |
| 1 | 7.103342 | -3.267328 | -0.661742 |
| 6 | 2.368160 | 3.296595 | 0.227690 |
| 6 | 2.563701 | 4.072642 | -0.900709 |
| 6 | 3.181532 | 5.354421 | -0.766793 |
| 6 | 3.345946 | 6.234104 | -1.871743 |
| 1 | 2.983380 | 5.921372 | -2.844710 |
| 6 | 3.946470 | 7.463954 | -1.711640 |
| 1 | 4.059354 | 8.124890 | -2.566359 |
| 6 | 4.419232 | 7.875189 | -0.441008 |
| 1 | 4.896048 | 8.844989 | -0.330914 |
| 6 | 4.276688 | 7.045736 | 0.648307 |
| 1 | 4.639123 | 7.351737 | 1.626676 |
| 6 | 3.652053 | 5.774804 | 0.519510 |
| 6 | 3.523272 | 4.892539 | 1.619499 |
| 1 | 3.961354 | 5.179500 | 2.571069 |
| 6 | 2.892760 | 3.665412 | 1.504923 |
| 6 | 2.860134 | 2.748152 | 2.672344 |
| 6 | 2.625588 | 3.228925 | 3.948669 |
| 1 | 2.319683 | 4.262907 | 4.080512 |
| 6 | 2.811595 | 2.418991 | 5.096634 |
| 6 | 2.602279 | 2.932991 | 6.406455 |
| 1 | 2.245262 | 3.954250 | 6.512560 |
| 6 | 2.863011 | 2.163018 | 7.516377 |
| 1 | 2.705494 | 2.566763 | 8.512309 |
| 6 | 3.354619 | 0.844062 | 7.360527 |
| 1 | 3.581629 | 0.247898 | 8.239741 |
| 6 | 3.551855 | 0.312090 | 6.105104 |
| 1 | 3.935126 | -0.695198 | 5.994219 |
| 6 | 3.265423 | 1.067696 | 4.935199 |
| 6 | 3.420732 | 0.532397 | 3.614251 |
| 6 | 3.203291 | 1.365782 | 2.530167 |
| 6 | 2.205231 | 3.517261 | -2.242221 |
| 6 | 0.883754 | 3.693026 | -2.759366 |
| 6 | -0.108033 | 4.443159 | -2.068508 |
| 1 | 0.133801 | 4.871657 | -1.102285 |
| 6 | -1.361800 | 4.628024 | -2.610197 |
| 1 | -2.104970 | 5.201154 | -2.065275 |
| 6 | -1.694880 | 4.059985 | -3.863709 |
| 1 | -2.687561 | 4.207798 | -4.273810 |
| 6 | -0.763535 | 3.311219 | -4.548060 |
| 1 | -1.013065 | 2.862643 | -5.506828 |
| 6 | 0.542028 | 3.111388 | -4.023990 |
| 6 | 1.524464 | 2.356097 | -4.713993 |
| 1 | 1.259406 | 1.872782 | -5.649972 |
| 6 | 2.792169 | 2.226116 | -4.204788 |
| 1 | 3.520677 | 1.634758 | -4.747899 |
| 6 | 3.166650 | 2.817134 | -2.966807 |
| 6 | 3.879353 | -0.878813 | 3.404606 |
| 6 | 5.223290 | -1.096451 | 2.935915 |
| 6 | 6.109292 | -0.031219 | 2.607609 |
| 1 | 5.777419 | 0.992441 | 2.735371 |
| 6 | 7.376633 | -0.275413 | 2.124836 |

| | | | |
|---|---|---|---|
| 1 | 8.025386 | 0.558459 | 1.871948 |
| 6 | 7.840432 | -1.601849 | 1.954402 |
| 1 | 8.842072 | -1.781413 | 1.573947 |
| 6 | 7.018643 | -2.657565 | 2.279845 |
| 1 | 7.361711 | -3.682659 | 2.162170 |
| 6 | 5.703185 | -2.436471 | 2.770092 |
| 6 | 4.845628 | -3.516073 | 3.104085 |
| 1 | 5.214219 | -4.533429 | 2.995661 |
| 6 | 3.568009 | -3.280389 | 3.542052 |
| 1 | 2.920067 | -4.120113 | 3.769258 |
| 6 | 3.047573 | -1.961758 | 3.682597 |
| 8 | 2.517219 | 0.026448 | -0.972329 |
| 8 | 0.957048 | -0.038261 | 1.120138 |
| 8 | 1.561821 | 2.185553 | 0.131116 |
| 8 | 3.406783 | 0.863137 | 1.259481 |
| 15 | 2.060334 | 0.614703 | 0.324240 |
| 6 | 1.584762 | -1.808105 | 4.064289 |
| 6 | 1.255705 | -2.314158 | 5.486992 |
| 6 | 0.676021 | -2.531001 | 3.044830 |
| 1 | 1.322956 | -0.747388 | 4.020703 |
| 6 | -0.230502 | -2.097778 | 5.816002 |
| 1 | 1.493647 | -3.385400 | 5.556249 |
| 1 | 1.877616 | -1.802765 | 6.228537 |
| 6 | -0.803323 | -2.297408 | 3.360321 |
| 1 | 0.878326 | -3.607170 | 3.062159 |
| 1 | 0.913592 | -2.169831 | 2.044048 |
| 6 | -1.150832 | -2.762884 | 4.780364 |
| 1 | -0.450297 | -2.474538 | 6.823280 |
| 1 | -0.433083 | -1.018115 | 5.830925 |
| 1 | -1.422651 | -2.821837 | 2.625066 |
| 1 | -1.017700 | -1.228097 | 3.271562 |
| 1 | -2.200592 | -2.539319 | 5.013506 |
| 1 | -1.036877 | -3.855581 | 4.840274 |
| 6 | 4.611095 | 2.716837 | -2.501357 |
| 6 | 5.528063 | 3.624040 | -3.355975 |
| 6 | 5.163173 | 1.277735 | -2.467767 |
| 1 | 4.662768 | 3.090569 | -1.473242 |
| 6 | 6.974938 | 3.598058 | -2.842005 |
| 1 | 5.499710 | 3.278985 | -4.399359 |
| 1 | 5.145025 | 4.650635 | -3.351121 |
| 6 | 6.608885 | 1.253923 | -1.950478 |
| 1 | 5.134869 | 0.837029 | -3.472876 |
| 1 | 4.514864 | 0.670046 | -1.836341 |
| 6 | 7.524653 | 2.165724 | -2.779576 |
| 1 | 7.611953 | 4.226436 | -3.477440 |
| 1 | 7.000940 | 4.041066 | -1.835793 |
| 1 | 6.988517 | 0.225414 | -1.958536 |
| 1 | 6.618859 | 1.580913 | -0.900458 |
| 1 | 8.542768 | 2.165340 | -2.369757 |
| 1 | 7.596546 | 1.765711 | -3.801368 |
| 1 | 0.198891 | 0.729052 | 2.488869 |
| 8 | -0.209287 | 1.177724 | 3.264749 |
| 1 | 0.530736 | 1.624757 | 3.692692 |

---

(*S*,*R*,*S*)-**[2a-3a]**$^{\ddagger}$endo-*re*

---

Number of imaginary frequencies : 1
The smallest frequencies are : -343.6904 12.1029 17.3108 cm(-1)

Electronic energy : HF=-6614.0094573
Zero-point correction= 1.888230 (Hartree/Particle)

Thermal correction to Energy= 1.998840
Thermal correction to Enthalpy= 1.999785
Thermal correction to Gibbs Free Energy= 1.737733
Sum of electronic and zero-point Energies= -6612.121227
Sum of electronic and thermal Energies= -6612.010617
Sum of electronic and thermal Enthalpies= -6612.009673
Sum of electronic and thermal Free Energies= -6612.271725

..............................................................

Cartesian Coordinates

..............................................................

| | | | |
|---|---|---|---|
| 6 | -3.632398 | 0.097907 | 2.779263 |
| 6 | -4.895184 | 0.298251 | 2.193962 |
| 6 | -6.044810 | -0.031026 | 2.927939 |
| 6 | -5.932213 | -0.557849 | 4.216709 |
| 6 | -4.673969 | -0.754330 | 4.790318 |
| 6 | -3.525982 | -0.417504 | 4.071130 |
| 15 | -4.905780 | 0.911629 | 0.451329 |
| 6 | -6.653658 | 0.787175 | -0.126752 |
| 6 | -7.694393 | 1.516499 | 0.475031 |
| 6 | -9.005232 | 1.387018 | 0.017964 |
| 6 | -9.292053 | 0.541955 | -1.058500 |
| 6 | -8.262340 | -0.164280 | -1.680558 |
| 6 | -6.950518 | -0.040453 | -1.217926 |
| 46 | -3.286760 | -0.299360 | -0.809372 |
| 6 | -1.381585 | -0.576358 | -1.836008 |
| 6 | -1.236943 | 0.557416 | -1.011374 |
| 6 | -1.239061 | 1.924377 | -1.470143 |
| 15 | -3.890076 | -2.578729 | -0.590416 |
| 6 | -5.667590 | -3.052510 | -0.418896 |
| 6 | -6.353207 | -2.705764 | 0.757873 |
| 6 | -7.723284 | -2.929044 | 0.876088 |
| 6 | -8.438043 | -3.492942 | -0.183550 |
| 6 | -7.767354 | -3.838280 | -1.357949 |
| 6 | -6.391966 | -3.622904 | -1.476413 |
| 6 | -4.748435 | 2.746360 | 0.648403 |
| 6 | -5.172894 | 3.573473 | -0.407176 |
| 6 | -4.977771 | 4.953693 | -0.351989 |
| 6 | -4.342550 | 5.529040 | 0.751309 |
| 6 | -3.911036 | 4.715646 | 1.799617 |
| 6 | -4.115941 | 3.335141 | 1.752992 |
| 6 | -3.326824 | -3.744769 | -1.904577 |
| 6 | -3.028271 | -5.091708 | -1.650102 |
| 6 | -2.693375 | -5.949654 | -2.699013 |
| 6 | -2.670629 | -5.476521 | -4.014334 |
| 6 | -2.967739 | -4.137053 | -4.275757 |
| 6 | -3.279271 | -3.272206 | -3.224125 |
| 6 | -3.123400 | -3.201074 | 0.971712 |
| 6 | -1.939685 | -2.591961 | 1.419700 |
| 6 | -1.347354 | -2.978026 | 2.622037 |
| 6 | -1.934582 | -3.978648 | 3.397464 |
| 6 | -3.100311 | -4.608702 | 2.953742 |
| 6 | -3.690175 | -4.226953 | 1.747528 |
| 1 | -1.585225 | 2.100805 | -2.486020 |
| 1 | -1.532577 | -0.448157 | -2.907217 |
| 1 | -0.896251 | -1.507397 | -1.561761 |
| 1 | -5.662588 | 3.138116 | -1.273969 |
| 1 | -3.761519 | 2.718756 | 2.571725 |
| 1 | -5.309888 | 5.577580 | -1.177118 |
| 1 | -3.393213 | 5.153372 | 2.647395 |
| 1 | -4.169210 | 6.599861 | 0.786889 |
| 1 | -7.476581 | 2.191960 | 1.296976 |
| 1 | -6.152220 | -0.594938 | -1.698837 |

| | | | |
|---|---|---|---|
| 1 | -9.801600 | 1.950104 | 0.496772 |
| 1 | -8.476051 | -0.820499 | -2.518684 |
| 1 | -10.314047 | 0.442443 | -1.413698 |
| 1 | -7.029260 | 0.093106 | 2.491453 |
| 1 | -2.722187 | 0.348214 | 2.241097 |
| 1 | -6.831172 | -0.819641 | 4.768526 |
| 1 | -2.541075 | -0.571159 | 4.498958 |
| 1 | -4.589205 | -1.172467 | 5.789839 |
| 1 | -3.050857 | -5.469045 | -0.632669 |
| 1 | -3.483822 | -2.223155 | -3.421536 |
| 1 | -2.451851 | -6.987798 | -2.489242 |
| 1 | -2.945127 | -3.762004 | -5.294923 |
| 1 | -2.411574 | -6.145908 | -4.829515 |
| 1 | -5.812839 | -2.259487 | 1.584170 |
| 1 | -5.887690 | -3.902965 | -2.395618 |
| 1 | -8.232738 | -2.652302 | 1.794897 |
| 1 | -8.312078 | -4.282994 | -2.186497 |
| 1 | -9.507705 | -3.660132 | -0.095213 |
| 1 | -1.465911 | -1.804124 | 0.847311 |
| 1 | -4.604351 | -4.714393 | 1.425223 |
| 1 | -0.434170 | -2.483478 | 2.932904 |
| 1 | -3.557026 | -5.395890 | 3.547635 |
| 1 | -1.483836 | -4.270453 | 4.342002 |
| 1 | -0.814773 | 0.413234 | -0.023126 |
| 1 | 1.085220 | 1.514967 | -1.317675 |
| 6 | 0.807633 | 3.548770 | -0.855037 |
| 6 | -0.568502 | 4.239516 | -0.654669 |
| 1 | 1.175506 | 3.154052 | 0.090795 |
| 1 | 1.573558 | 4.213271 | -1.256038 |
| 7 | 0.606811 | 2.365452 | -1.724556 |
| 16 | 1.035339 | 2.416503 | -3.431782 |
| 8 | 0.002747 | 3.198267 | -4.113864 |
| 8 | 1.234635 | 1.011071 | -3.769859 |
| 6 | -1.509064 | 3.021086 | -0.465208 |
| 1 | -1.366840 | 2.604681 | 0.532676 |
| 1 | -2.551477 | 3.325682 | -0.528738 |
| 6 | -0.909718 | 5.091643 | -1.887943 |
| 6 | -2.128983 | 4.987188 | -2.565958 |
| 6 | 0.009322 | 6.052208 | -2.341608 |
| 6 | -2.413546 | 5.793368 | -3.670150 |
| 1 | -2.874346 | 4.270807 | -2.244596 |
| 6 | -0.271549 | 6.864867 | -3.438749 |
| 1 | 0.953247 | 6.183453 | -1.821154 |
| 6 | -1.485987 | 6.733514 | -4.115883 |
| 1 | -3.364672 | 5.678851 | -4.182924 |
| 1 | 0.459851 | 7.599935 | -3.764278 |
| 1 | -1.706392 | 7.358720 | -4.976506 |
| 6 | -0.575593 | 5.089961 | 0.636198 |
| 6 | -0.241008 | 4.494004 | 1.866391 |
| 6 | -0.981296 | 6.431181 | 0.649303 |
| 6 | -0.313631 | 5.217486 | 3.056606 |
| 1 | 0.036146 | 3.445448 | 1.914883 |
| 6 | -1.043919 | 7.158821 | 1.840948 |
| 1 | -1.270162 | 6.918681 | -0.273383 |
| 6 | -0.711695 | 6.556342 | 3.053100 |
| 1 | -0.070614 | 4.723923 | 3.992316 |
| 1 | -1.363890 | 8.197054 | 1.815114 |
| 1 | -0.766903 | 7.116953 | 3.982157 |
| 6 | 2.575370 | 3.347463 | -3.493668 |
| 6 | 3.801383 | 2.848786 | -2.940912 |
| 6 | 2.492229 | 4.584618 | -4.102151 |
| 6 | 3.953071 | 1.587247 | -2.309416 |

| | | | |
|---|---|---|---|
| 6 | 4.948202 | 3.714556 | -3.026972 |
| 6 | 3.634823 | 5.409917 | -4.185560 |
| 1 | 1.544221 | 4.922930 | -4.500850 |
| 6 | 5.168498 | 1.208856 | -1.779375 |
| 1 | 3.121804 | 0.904079 | -2.233308 |
| 6 | 6.187564 | 3.281270 | -2.483918 |
| 6 | 4.829910 | 4.983683 | -3.654648 |
| 1 | 3.556033 | 6.379817 | -4.666261 |
| 6 | 6.297115 | 2.054480 | -1.868932 |
| 1 | 5.249659 | 0.246130 | -1.284564 |
| 1 | 7.046718 | 3.942188 | -2.561028 |
| 1 | 5.710601 | 5.618575 | -3.707395 |
| 1 | 7.246726 | 1.733883 | -1.450777 |
| 6 | 3.818501 | -2.326577 | -0.485203 |
| 6 | 3.900469 | -2.791973 | -1.791669 |
| 6 | 5.178124 | -3.215539 | -2.290732 |
| 6 | 5.335712 | -3.828000 | -3.566064 |
| 1 | 4.457602 | -4.003865 | -4.175873 |
| 6 | 6.579309 | -4.199470 | -4.026378 |
| 1 | 6.673434 | -4.670050 | -5.000954 |
| 6 | 7.737459 | -3.973252 | -3.242815 |
| 1 | 8.713028 | -4.262708 | -3.622859 |
| 6 | 7.619752 | -3.401942 | -1.997024 |
| 1 | 8.498405 | -3.240108 | -1.377396 |
| 6 | 6.346538 | -3.027900 | -1.483829 |
| 6 | 6.204629 | -2.506828 | -0.176604 |
| 1 | 7.095466 | -2.371294 | 0.429944 |
| 6 | 4.965872 | -2.205957 | 0.362907 |
| 6 | 4.855609 | -1.859275 | 1.802760 |
| 6 | 5.631016 | -2.527057 | 2.734338 |
| 1 | 6.281566 | -3.332911 | 2.407781 |
| 6 | 5.596295 | -2.202309 | 4.111778 |
| 6 | 6.431129 | -2.867831 | 5.051327 |
| 1 | 7.103512 | -3.642658 | 4.691664 |
| 6 | 6.396064 | -2.535479 | 6.386197 |
| 1 | 7.040870 | -3.047116 | 7.094978 |
| 6 | 5.519816 | -1.518527 | 6.837705 |
| 1 | 5.502889 | -1.252926 | 7.890951 |
| 6 | 4.688980 | -0.864285 | 5.954899 |
| 1 | 4.022803 | -0.087024 | 6.311511 |
| 6 | 4.695269 | -1.186857 | 4.569484 |
| 6 | 3.838784 | -0.536433 | 3.621644 |
| 6 | 3.968053 | -0.847425 | 2.279855 |
| 6 | 2.681356 | -2.856671 | -2.660438 |
| 6 | 2.630186 | -2.020386 | -3.834552 |
| 6 | 3.677077 | -1.127595 | -4.198167 |
| 1 | 4.531769 | -1.015924 | -3.544458 |
| 6 | 3.625272 | -0.395476 | -5.363147 |
| 1 | 4.441048 | 0.279777 | -5.606294 |
| 6 | 2.515666 | -0.500217 | -6.232271 |
| 1 | 2.485407 | 0.083470 | -7.148099 |
| 6 | 1.466934 | -1.324255 | -5.896969 |
| 1 | 0.594179 | -1.399264 | -6.541393 |
| 6 | 1.493247 | -2.093599 | -4.703371 |
| 6 | 0.417759 | -2.945531 | -4.347722 |
| 1 | -0.460576 | -2.984712 | -4.985950 |
| 6 | 0.484531 | -3.723083 | -3.220418 |
| 1 | -0.349258 | -4.371865 | -2.976488 |
| 6 | 1.625545 | -3.717497 | -2.369750 |
| 6 | 2.787714 | 0.418281 | 4.094190 |
| 6 | 1.671950 | -0.132308 | 4.818596 |
| 6 | 1.477380 | -1.534394 | 4.970942 |

| | | | |
|---|---|---|---|
| 1 | 2.170076 | -2.217897 | 4.495443 |
| 6 | 0.416152 | -2.032311 | 5.693845 |
| 1 | 0.292226 | -3.106490 | 5.795000 |
| 6 | -0.517275 | -1.158191 | 6.301146 |
| 1 | -1.348978 | -1.564518 | 6.869639 |
| 6 | -0.376812 | 0.203184 | 6.152241 |
| 1 | -1.098817 | 0.885344 | 6.593994 |
| 6 | 0.706746 | 0.746715 | 5.409410 |
| 6 | 0.854690 | 2.147003 | 5.217144 |
| 1 | 0.126985 | 2.820350 | 5.663443 |
| 6 | 1.892738 | 2.639069 | 4.464726 |
| 1 | 1.973997 | 3.709461 | 4.308909 |
| 6 | 2.880925 | 1.790572 | 3.889541 |
| 8 | 1.813678 | 0.284470 | -0.610308 |
| 8 | 0.834505 | -0.985863 | 1.464006 |
| 8 | 2.569037 | -2.125150 | 0.060621 |
| 8 | 3.291292 | -0.082564 | 1.356992 |
| 15 | 1.975823 | -0.664921 | 0.540672 |
| 6 | 3.980376 | 2.428891 | 3.059666 |
| 6 | 3.393865 | 3.099638 | 1.795512 |
| 6 | 4.835115 | 3.432108 | 3.864691 |
| 1 | 4.656065 | 1.640277 | 2.715876 |
| 6 | 4.490257 | 3.735639 | 0.932860 |
| 1 | 2.668929 | 3.869998 | 2.091385 |
| 1 | 2.853454 | 2.344323 | 1.219524 |
| 6 | 5.932694 | 4.056332 | 2.988802 |
| 1 | 4.195630 | 4.232827 | 4.260272 |
| 1 | 5.277149 | 2.925588 | 4.731442 |
| 6 | 5.340793 | 4.725675 | 1.739260 |
| 1 | 4.042840 | 4.233375 | 0.063802 |
| 1 | 5.133022 | 2.943154 | 0.531604 |
| 1 | 6.512287 | 4.781143 | 3.573857 |
| 1 | 6.635268 | 3.269834 | 2.677549 |
| 1 | 6.141317 | 5.138109 | 1.112504 |
| 1 | 4.713585 | 5.574188 | 2.049484 |
| 6 | 1.656228 | -4.711920 | -1.222113 |
| 6 | 0.486080 | -4.522177 | -0.234129 |
| 6 | 1.686826 | -6.166271 | -1.749816 |
| 1 | 2.581244 | -4.566582 | -0.656941 |
| 6 | 0.555975 | -5.536959 | 0.915244 |
| 1 | -0.466226 | -4.649363 | -0.762539 |
| 1 | 0.502176 | -3.504302 | 0.160565 |
| 6 | 1.758260 | -7.177888 | -0.596373 |
| 1 | 0.783117 | -6.357428 | -2.344448 |
| 1 | 2.541198 | -6.293226 | -2.426322 |
| 6 | 0.600998 | -6.979663 | 0.392804 |
| 1 | -0.300045 | -5.398904 | 1.583616 |
| 1 | 1.457083 | -5.337946 | 1.513514 |
| 1 | 1.756274 | -8.201707 | -0.991522 |
| 1 | 2.711935 | -7.048143 | -0.064740 |
| 1 | 0.685889 | -7.688803 | 1.226171 |
| 1 | -0.347784 | -7.205737 | -0.115787 |
| 1 | -0.036807 | 0.388339 | 2.028796 |
| 8 | -0.539010 | 1.200773 | 2.273332 |
| 1 | -0.277092 | 1.382900 | 3.184300 |

---

(*S*,*R*,*S*)-**[2a-3a]**$^‡$exo-*si*

---

Number of imaginary frequencies : 1
The smallest frequencies are : -325.7967 12.9637 15.6376 cm(-1)

Electronic energy : HF=-6614.0161065
Zero-point correction= 1.888435 (Hartree/Particle)
Thermal correction to Energy= 1.998766
Thermal correction to Enthalpy= 1.999710
Thermal correction to Gibbs Free Energy= 1.737322
Sum of electronic and zero-point Energies= -6612.127671
Sum of electronic and thermal Energies= -6612.017340
Sum of electronic and thermal Enthalpies= -6612.016396
Sum of electronic and thermal Free Energies= -6612.278784

..............................................................

Cartesian Coordinates

..............................................................

| | | | |
|---|---|---|---|
| 6 | -3.677269 | 0.762833 | 2.380669 |
| 6 | -4.983649 | 0.827092 | 1.864950 |
| 6 | -6.066567 | 0.786567 | 2.756309 |
| 6 | -5.843301 | 0.682091 | 4.131659 |
| 6 | -4.540417 | 0.625062 | 4.634482 |
| 6 | -3.456625 | 0.669133 | 3.755050 |
| 15 | -5.123852 | 0.908760 | 0.024741 |
| 6 | -6.872288 | 0.512384 | -0.413160 |
| 6 | -7.950755 | 1.311255 | 0.005080 |
| 6 | -9.255188 | 0.989021 | -0.367410 |
| 6 | -9.498402 | -0.121693 | -1.180893 |
| 6 | -8.431181 | -0.900740 | -1.628461 |
| 6 | -7.125554 | -0.582109 | -1.249993 |
| 46 | -3.396204 | -0.436214 | -0.917883 |
| 6 | -1.527373 | -0.742424 | -1.945458 |
| 6 | -1.823132 | 0.632970 | -2.141042 |
| 6 | -1.293194 | 1.642351 | -1.263525 |
| 15 | -3.804395 | -2.630695 | -0.128834 |
| 6 | -5.557188 | -3.080054 | 0.258563 |
| 6 | -6.168762 | -2.474790 | 1.369881 |
| 6 | -7.509928 | -2.712467 | 1.660424 |
| 6 | -8.267338 | -3.555399 | 0.842887 |
| 6 | -7.668300 | -4.164635 | -0.260275 |
| 6 | -6.321186 | -3.931466 | -0.551553 |
| 6 | -5.158702 | 2.727232 | -0.312678 |
| 6 | -5.296525 | 3.132072 | -1.652306 |
| 6 | -5.361222 | 4.483981 | -1.980544 |
| 6 | -5.271118 | 5.454829 | -0.975746 |
| 6 | -5.111449 | 5.062507 | 0.352832 |
| 6 | -5.062444 | 3.704993 | 0.685309 |
| 6 | -3.325365 | -3.995150 | -1.277039 |
| 6 | -2.960475 | -5.273477 | -0.828507 |
| 6 | -2.687535 | -6.291451 | -1.744140 |
| 6 | -2.785752 | -6.048764 | -3.116900 |
| 6 | -3.151700 | -4.779899 | -3.572017 |
| 6 | -3.410765 | -3.757173 | -2.657032 |
| 6 | -2.959641 | -3.014078 | 1.464529 |
| 6 | -1.790798 | -2.315282 | 1.796853 |
| 6 | -1.145804 | -2.552504 | 3.012262 |
| 6 | -1.665846 | -3.485619 | 3.908892 |
| 6 | -2.821338 | -4.200729 | 3.582110 |
| 6 | -3.465133 | -3.967679 | 2.367153 |
| 1 | -1.002250 | 1.301652 | -0.270475 |
| 1 | -0.752082 | -1.020056 | -1.234630 |
| 1 | -1.615652 | -1.423450 | -2.786234 |
| 1 | -5.353059 | 2.382757 | -2.438109 |
| 1 | -4.945005 | 3.413467 | 1.723293 |
| 1 | -5.470162 | 4.782352 | -3.019416 |
| 1 | -5.019405 | 5.808970 | 1.136391 |
| 1 | -5.310654 | 6.509734 | -1.231973 |

| | | | |
|---|---|---|---|
| 1 | -7.769214 | 2.196849 | 0.607092 |
| 1 | -6.297961 | -1.189203 | -1.598285 |
| 1 | -10.080653 | 1.610964 | -0.032137 |
| 1 | -8.609867 | -1.762064 | -2.264807 |
| 1 | -10.515177 | -0.370583 | -1.471773 |
| 1 | -7.084268 | 0.808403 | 2.383008 |
| 1 | -2.810384 | 0.766436 | 1.727393 |
| 1 | -6.691013 | 0.639342 | 4.810216 |
| 1 | -2.435554 | 0.607098 | 4.117595 |
| 1 | -4.372811 | 0.535865 | 5.704428 |
| 1 | -2.882842 | -5.475472 | 0.234662 |
| 1 | -3.679786 | -2.766259 | -3.013120 |
| 1 | -2.395661 | -7.274208 | -1.384858 |
| 1 | -3.224275 | -4.581624 | -4.637653 |
| 1 | -2.569987 | -6.841661 | -3.827103 |
| 1 | -5.591670 | -1.820425 | 2.013326 |
| 1 | -5.871009 | -4.417523 | -1.410719 |
| 1 | -7.964029 | -2.232230 | 2.522605 |
| 1 | -8.246701 | -4.827831 | -0.897908 |
| 1 | -9.315125 | -3.735897 | 1.065357 |
| 1 | -1.372804 | -1.581930 | 1.117006 |
| 1 | -4.370954 | -4.516266 | 2.130584 |
| 1 | -0.239117 | -2.006503 | 3.236136 |
| 1 | -3.225748 | -4.936144 | 4.272472 |
| 1 | -1.166635 | -3.658681 | 4.858296 |
| 1 | -2.222621 | 0.971404 | -3.095063 |
| 1 | 1.048700 | 1.199908 | -1.291105 |
| 6 | 0.671483 | 3.226943 | -0.769486 |
| 6 | -0.652373 | 4.060761 | -0.788490 |
| 1 | 0.792273 | 2.786438 | 0.220803 |
| 1 | 1.566873 | 3.809696 | -0.981057 |
| 7 | 0.560326 | 2.061472 | -1.665237 |
| 16 | 0.899646 | 2.205224 | -3.366738 |
| 8 | -0.132319 | 3.084478 | -3.926336 |
| 8 | 1.028566 | 0.824286 | -3.820981 |
| 6 | -1.738655 | 3.079880 | -1.348456 |
| 1 | -2.680896 | 3.191072 | -0.814095 |
| 1 | -1.936184 | 3.316718 | -2.392924 |
| 6 | -0.601271 | 5.324985 | -1.666023 |
| 6 | -1.813070 | 5.935240 | -2.028231 |
| 6 | 0.585567 | 5.959653 | -2.049291 |
| 6 | -1.838173 | 7.114897 | -2.768132 |
| 1 | -2.750779 | 5.487917 | -1.719716 |
| 6 | 0.566342 | 7.145488 | -2.790982 |
| 1 | 1.548687 | 5.543472 | -1.780003 |
| 6 | -0.644729 | 7.727677 | -3.159218 |
| 1 | -2.793601 | 7.558110 | -3.036433 |
| 1 | 1.506489 | 7.609373 | -3.077092 |
| 1 | -0.660872 | 8.647706 | -3.736577 |
| 6 | -0.920038 | 4.508382 | 0.669971 |
| 6 | -1.779277 | 3.798436 | 1.518043 |
| 6 | -0.248235 | 5.620849 | 1.201437 |
| 6 | -1.987215 | 4.199303 | 2.838712 |
| 1 | -2.289527 | 2.914386 | 1.166070 |
| 6 | -0.446445 | 6.021731 | 2.524201 |
| 1 | 0.422094 | 6.196804 | 0.572312 |
| 6 | -1.328143 | 5.318747 | 3.348203 |
| 1 | -2.661352 | 3.621591 | 3.465264 |
| 1 | 0.080651 | 6.892550 | 2.904581 |
| 1 | -1.493358 | 5.635835 | 4.374244 |
| 6 | 2.479539 | 3.066000 | -3.514572 |
| 6 | 3.718240 | 2.492814 | -3.075149 |

| | | | |
|---|---|---|---|
| 6 | 2.426938 | 4.283850 | -4.163909 |
| 6 | 3.841169 | 1.253978 | -2.394571 |
| 6 | 4.913599 | 3.251115 | -3.336877 |
| 6 | 3.612843 | 5.010262 | -4.408543 |
| 1 | 1.471293 | 4.682213 | -4.480195 |
| 6 | 5.076036 | 0.800769 | -1.980674 |
| 1 | 2.975735 | 0.637957 | -2.199565 |
| 6 | 6.169253 | 2.740196 | -2.912590 |
| 6 | 4.824797 | 4.502974 | -4.002414 |
| 1 | 3.553999 | 5.966168 | -4.919193 |
| 6 | 6.250986 | 1.539597 | -2.244509 |
| 1 | 5.139311 | -0.140404 | -1.446693 |
| 1 | 7.062949 | 3.322284 | -3.121071 |
| 1 | 5.741304 | 5.057285 | -4.187416 |
| 1 | 7.213069 | 1.157649 | -1.915934 |
| 6 | 3.930603 | -2.489414 | -0.334949 |
| 6 | 3.919542 | -3.098387 | -1.582093 |
| 6 | 5.169531 | -3.530345 | -2.139910 |
| 6 | 5.249931 | -4.260142 | -3.359191 |
| 1 | 4.333815 | -4.513168 | -3.879958 |
| 6 | 6.466437 | -4.646540 | -3.876267 |
| 1 | 6.502591 | -5.205948 | -4.806775 |
| 6 | 7.671916 | -4.322147 | -3.207011 |
| 1 | 8.624367 | -4.629660 | -3.629259 |
| 6 | 7.630834 | -3.627827 | -2.020258 |
| 1 | 8.547993 | -3.382910 | -1.490213 |
| 6 | 6.389976 | -3.224496 | -1.453391 |
| 6 | 6.337648 | -2.534446 | -0.218869 |
| 1 | 7.270548 | -2.282289 | 0.277014 |
| 6 | 5.136920 | -2.188258 | 0.374527 |
| 6 | 5.117484 | -1.566636 | 1.723552 |
| 6 | 5.989737 | -2.000568 | 2.706066 |
| 1 | 6.669249 | -2.820433 | 2.492298 |
| 6 | 6.010176 | -1.422222 | 3.998491 |
| 6 | 6.937137 | -1.850352 | 4.988455 |
| 1 | 7.648258 | -2.631681 | 4.731972 |
| 6 | 6.940724 | -1.285636 | 6.243468 |
| 1 | 7.655902 | -1.617615 | 6.990585 |
| 6 | 6.009509 | -0.267514 | 6.563396 |
| 1 | 6.019884 | 0.177624 | 7.554333 |
| 6 | 5.090722 | 0.161631 | 5.630893 |
| 1 | 4.379578 | 0.939586 | 5.885869 |
| 6 | 5.060862 | -0.398938 | 4.323896 |
| 6 | 4.118642 | 0.020530 | 3.330029 |
| 6 | 4.205474 | -0.519001 | 2.058605 |
| 6 | 2.635377 | -3.263908 | -2.338572 |
| 6 | 2.475626 | -2.526031 | -3.568722 |
| 6 | 3.465447 | -1.638929 | -4.077888 |
| 1 | 4.369682 | -1.467709 | -3.510113 |
| 6 | 3.295179 | -0.981383 | -5.275710 |
| 1 | 4.067083 | -0.302007 | -5.626568 |
| 6 | 2.120352 | -1.164882 | -6.038878 |
| 1 | 1.995205 | -0.636688 | -6.979903 |
| 6 | 1.128353 | -1.991959 | -5.567278 |
| 1 | 0.207060 | -2.127511 | -6.128992 |
| 6 | 1.274150 | -2.680320 | -4.333850 |
| 6 | 0.250002 | -3.522656 | -3.833475 |
| 1 | -0.670858 | -3.633393 | -4.399625 |
| 6 | 0.414282 | -4.193270 | -2.649549 |
| 1 | -0.387800 | -4.826536 | -2.287904 |
| 6 | 1.611532 | -4.093049 | -1.885783 |
| 6 | 3.008927 | 0.963970 | 3.676147 |

| | | | |
|---|---|---|---|
| 6 | 1.934316 | 0.457841 | 4.489141 |
| 6 | 1.869926 | -0.896807 | 4.920966 |
| 1 | 2.636430 | -1.590833 | 4.599614 |
| 6 | 0.840289 | -1.341897 | 5.720064 |
| 1 | 0.815819 | -2.381693 | 6.033331 |
| 6 | -0.193425 | -0.463258 | 6.124890 |
| 1 | -1.002183 | -0.830274 | 6.750204 |
| 6 | -0.174239 | 0.848704 | 5.709594 |
| 1 | -0.965269 | 1.535152 | 6.001684 |
| 6 | 0.875981 | 1.336664 | 4.884963 |
| 6 | 0.892492 | 2.677572 | 4.419287 |
| 1 | 0.083496 | 3.345644 | 4.696627 |
| 6 | 1.908798 | 3.123233 | 3.610209 |
| 1 | 1.883762 | 4.148186 | 3.252956 |
| 6 | 2.998366 | 2.283176 | 3.239369 |
| 8 | 1.636410 | -0.155313 | -0.714248 |
| 8 | 1.108379 | -0.992688 | 1.710891 |
| 8 | 2.728512 | -2.284334 | 0.304981 |
| 8 | 3.424704 | 0.014596 | 1.050717 |
| 15 | 2.066057 | -0.803712 | 0.572474 |
| 6 | 4.120886 | 2.876722 | 2.407315 |
| 6 | 3.622893 | 3.370773 | 1.032457 |
| 6 | 4.846787 | 4.009617 | 3.168175 |
| 1 | 4.860826 | 2.092354 | 2.216772 |
| 6 | 4.754364 | 3.997638 | 0.207764 |
| 1 | 2.826145 | 4.112724 | 1.178203 |
| 1 | 3.186998 | 2.525723 | 0.492737 |
| 6 | 5.981488 | 4.617358 | 2.331158 |
| 1 | 4.126088 | 4.799064 | 3.421775 |
| 1 | 5.233768 | 3.621031 | 4.118022 |
| 6 | 5.464939 | 5.120013 | 0.975310 |
| 1 | 4.361291 | 4.374049 | -0.743801 |
| 1 | 5.480775 | 3.217535 | -0.051022 |
| 1 | 6.462561 | 5.432227 | 2.886219 |
| 1 | 6.753388 | 3.853327 | 2.161301 |
| 1 | 6.290888 | 5.525412 | 0.377733 |
| 1 | 4.761255 | 5.948876 | 1.142496 |
| 6 | 1.717296 | -4.943847 | -0.631403 |
| 6 | 0.607880 | -4.620051 | 0.394439 |
| 6 | 1.711144 | -6.453461 | -0.971262 |
| 1 | 2.674968 | -4.736812 | -0.145294 |
| 6 | 0.738827 | -5.482452 | 1.656023 |
| 1 | -0.374352 | -4.800034 | -0.058180 |
| 1 | 0.646969 | -3.561819 | 0.658311 |
| 6 | 1.841829 | -7.313040 | 0.294347 |
| 1 | 0.775728 | -6.708581 | -1.488111 |
| 1 | 2.526361 | -6.676733 | -1.670585 |
| 6 | 0.745492 | -6.979626 | 1.316873 |
| 1 | -0.077448 | -5.248287 | 2.347600 |
| 1 | 1.673008 | -5.222104 | 2.174579 |
| 1 | 1.811425 | -8.378179 | 0.031317 |
| 1 | 2.825209 | -7.131012 | 0.751308 |
| 1 | 0.877066 | -7.580640 | 2.225589 |
| 1 | -0.233005 | -7.255895 | 0.896529 |
| 1 | 0.057902 | 0.329846 | 1.829429 |
| 8 | -0.520555 | 1.127241 | 1.767373 |
| 1 | -0.179039 | 1.750154 | 2.420975 |

---

(*S*,*R*,*S*)-**[2a-3a]**‡exo-*re*

---

Number of imaginary frequencies : 1

The smallest frequencies are : -275.7137 10.1934 14.1091 cm(-1)

Electronic energy : HF=-6614.0104044
Zero-point correction= 1.888424 (Hartree/Particle)
Thermal correction to Energy= 1.999110
Thermal correction to Enthalpy= 2.000055
Thermal correction to Gibbs Free Energy= 1.735497
Sum of electronic and zero-point Energies= -6612.121980
Sum of electronic and thermal Energies= -6612.011294
Sum of electronic and thermal Enthalpies= -6612.010350
Sum of electronic and thermal Free Energies= -6612.274907

Cartesian Coordinates

| | | | |
|---|---|---|---|
| 6 | 6.905608 | -0.469507 | -1.120234 |
| 6 | 6.823321 | 0.271726 | 0.070299 |
| 6 | 7.951543 | 0.330555 | 0.901308 |
| 6 | 9.125215 | -0.340237 | 0.552672 |
| 6 | 9.193378 | -1.077411 | -0.632187 |
| 6 | 8.079896 | -1.136550 | -1.471829 |
| 15 | 5.176297 | 1.030918 | 0.470860 |
| 6 | 5.177871 | 1.312756 | 2.290620 |
| 6 | 6.073706 | 2.170096 | 2.957071 |
| 6 | 6.001560 | 2.332098 | 4.339924 |
| 6 | 5.030684 | 1.645398 | 5.076608 |
| 6 | 4.131055 | 0.804007 | 4.422934 |
| 6 | 4.201432 | 0.638540 | 3.037354 |
| 46 | 3.431346 | -0.318918 | -0.435015 |
| 6 | 1.788365 | -0.673959 | -1.801732 |
| 6 | 2.049143 | 0.713201 | -1.897375 |
| 6 | 1.333128 | 1.682497 | -1.092133 |
| 15 | 3.949998 | -2.518822 | 0.297841 |
| 6 | 4.858929 | -2.630537 | 1.905159 |
| 6 | 6.241079 | -2.388238 | 1.966310 |
| 6 | 6.898841 | -2.329943 | 3.195180 |
| 6 | 6.188874 | -2.509976 | 4.383543 |
| 6 | 4.815710 | -2.756813 | 4.332676 |
| 6 | 4.154472 | -2.817385 | 3.105607 |
| 6 | 5.390079 | 2.734582 | -0.235961 |
| 6 | 5.051075 | 3.901640 | 0.467384 |
| 6 | 5.151537 | 5.156489 | -0.139854 |
| 6 | 5.578081 | 5.266787 | -1.463619 |
| 6 | 5.899039 | 4.110140 | -2.180213 |
| 6 | 5.805747 | 2.858667 | -1.573279 |
| 6 | 2.548095 | -3.685605 | 0.603534 |
| 6 | 2.676157 | -5.082774 | 0.540807 |
| 6 | 1.586917 | -5.900368 | 0.844164 |
| 6 | 0.366331 | -5.338545 | 1.233187 |
| 6 | 0.234350 | -3.950999 | 1.300359 |
| 6 | 1.317599 | -3.131379 | 0.974693 |
| 6 | 5.024002 | -3.439106 | -0.892235 |
| 6 | 4.919773 | -3.107150 | -2.252770 |
| 6 | 5.685579 | -3.773633 | -3.210373 |
| 6 | 6.574638 | -4.778358 | -2.820338 |
| 6 | 6.687325 | -5.116088 | -1.469983 |
| 6 | 5.915869 | -4.454452 | -0.512475 |
| 1 | 0.934112 | 1.275016 | -0.166881 |
| 1 | 2.066170 | -1.311840 | -2.634442 |
| 1 | 0.936556 | -1.020034 | -1.225641 |
| 1 | 4.706473 | 3.838300 | 1.493008 |
| 1 | 6.064202 | 1.972804 | -2.145652 |
| 1 | 4.883507 | 6.045910 | 0.423138 |

| | | | |
|---|---|---|---|
| 1 | 6.227236 | 4.181897 | -3.213531 |
| 1 | 5.654544 | 6.242339 | -1.935391 |
| 1 | 6.813430 | 2.730375 | 2.393685 |
| 1 | 3.480275 | 0.001655 | 2.537501 |
| 1 | 6.698535 | 2.997753 | 4.841822 |
| 1 | 3.366219 | 0.274115 | 4.982511 |
| 1 | 4.975061 | 1.774264 | 6.154206 |
| 1 | 7.911816 | 0.860893 | 1.844862 |
| 1 | 6.028504 | -0.561643 | -1.753197 |
| 1 | 9.984374 | -0.295954 | 1.216640 |
| 1 | 8.107879 | -1.725589 | -2.383759 |
| 1 | 10.103572 | -1.610332 | -0.892501 |
| 1 | 3.616189 | -5.535947 | 0.242823 |
| 1 | 1.202228 | -2.060023 | 0.997233 |
| 1 | 1.689035 | -6.979971 | 0.773815 |
| 1 | -0.713657 | -3.495699 | 1.567499 |
| 1 | -0.481193 | -5.979771 | 1.454509 |
| 1 | 6.810020 | -2.248006 | 1.055295 |
| 1 | 3.085343 | -3.000038 | 3.086890 |
| 1 | 7.967531 | -2.136156 | 3.217838 |
| 1 | 4.252248 | -2.900449 | 5.250609 |
| 1 | 6.700388 | -2.456970 | 5.340540 |
| 1 | 4.237738 | -2.319347 | -2.555893 |
| 1 | 6.018931 | -4.722527 | 0.533887 |
| 1 | 5.592698 | -3.502047 | -4.258430 |
| 1 | 7.376642 | -5.896116 | -1.158710 |
| 1 | 7.177197 | -5.293344 | -3.563369 |
| 1 | 2.587772 | 1.105720 | -2.757584 |
| 1 | -1.089202 | 1.559930 | -1.231746 |
| 6 | -0.510761 | 3.578298 | -1.359788 |
| 6 | 0.671657 | 3.963687 | -0.431888 |
| 1 | -0.431967 | 4.194757 | -2.253979 |
| 1 | -1.497015 | 3.737682 | -0.920816 |
| 7 | -0.353069 | 2.147648 | -1.736615 |
| 16 | -0.575482 | 1.814494 | -3.447761 |
| 8 | -0.643131 | 0.360861 | -3.548581 |
| 8 | 0.467099 | 2.575448 | -4.144685 |
| 6 | 1.819975 | 3.109846 | -1.016100 |
| 1 | 2.084467 | 3.441461 | -2.025031 |
| 1 | 2.718332 | 3.161340 | -0.402339 |
| 6 | 0.485439 | 3.570473 | 1.042524 |
| 6 | 1.512636 | 3.895330 | 1.945551 |
| 6 | -0.612761 | 2.847834 | 1.516552 |
| 6 | 1.452507 | 3.495394 | 3.278922 |
| 1 | 2.367658 | 4.464570 | 1.594109 |
| 6 | -0.673436 | 2.440424 | 2.853375 |
| 1 | -1.429347 | 2.566728 | 0.864275 |
| 6 | 0.353803 | 2.760726 | 3.739031 |
| 1 | 2.267170 | 3.742988 | 3.953379 |
| 1 | -1.521747 | 1.854573 | 3.182353 |
| 1 | 0.307823 | 2.429400 | 4.772118 |
| 6 | 0.851857 | 5.479415 | -0.539356 |
| 6 | 1.945994 | 6.077639 | -1.171779 |
| 6 | -0.158992 | 6.304994 | -0.019140 |
| 6 | 2.030821 | 7.469711 | -1.279663 |
| 1 | 2.747694 | 5.470270 | -1.573838 |
| 6 | -0.080618 | 7.690913 | -0.136463 |
| 1 | -1.008532 | 5.851695 | 0.484816 |
| 6 | 1.018882 | 8.280904 | -0.767606 |
| 1 | 2.893935 | 7.915846 | -1.766697 |
| 1 | -0.874742 | 8.311865 | 0.269449 |
| 1 | 1.085265 | 9.361803 | -0.854583 |

| | | | |
|---|---|---|---|
| 6 | -2.217915 | 2.491593 | -3.756893 |
| 6 | -2.468146 | 3.822807 | -4.234209 |
| 6 | -3.241368 | 1.626497 | -3.415291 |
| 6 | -1.477270 | 4.768523 | -4.622561 |
| 6 | -3.849486 | 4.219500 | -4.326540 |
| 6 | -4.584967 | 2.038021 | -3.528334 |
| 1 | -3.011561 | 0.637786 | -3.041190 |
| 6 | -1.832045 | 6.031675 | -5.044337 |
| 1 | -0.433793 | 4.487931 | -4.596421 |
| 6 | -4.173239 | 5.530099 | -4.770623 |
| 6 | -4.877633 | 3.307126 | -3.969160 |
| 1 | -5.375087 | 1.350469 | -3.249406 |
| 6 | -3.188036 | 6.423889 | -5.118410 |
| 1 | -1.053621 | 6.733742 | -5.328353 |
| 1 | -5.221473 | 5.810980 | -4.828139 |
| 1 | -5.910637 | 3.635642 | -4.047503 |
| 1 | -3.446595 | 7.424154 | -5.452535 |
| 6 | -4.100058 | -0.742409 | 1.764938 |
| 6 | -4.902430 | 0.211454 | 2.364302 |
| 6 | -6.283713 | -0.092188 | 2.604175 |
| 6 | -7.177621 | 0.847490 | 3.187800 |
| 1 | -6.807257 | 1.831605 | 3.451625 |
| 6 | -8.498055 | 0.521961 | 3.412677 |
| 1 | -9.165916 | 1.255318 | 3.855705 |
| 6 | -8.994453 | -0.758334 | 3.068065 |
| 1 | -10.037313 | -1.000916 | 3.250933 |
| 6 | -8.154799 | -1.690809 | 2.501996 |
| 1 | -8.524824 | -2.676857 | 2.232586 |
| 6 | -6.787592 | -1.387524 | 2.259827 |
| 6 | -5.907950 | -2.339268 | 1.687789 |
| 1 | -6.297166 | -3.316947 | 1.418359 |
| 6 | -4.580705 | -2.047975 | 1.434305 |
| 6 | -3.727764 | -3.081674 | 0.789918 |
| 6 | -3.762020 | -4.402012 | 1.194932 |
| 1 | -4.281841 | -4.667432 | 2.111163 |
| 6 | -3.162601 | -5.426644 | 0.420291 |
| 6 | -3.171620 | -6.780729 | 0.854755 |
| 1 | -3.601658 | -7.012096 | 1.826070 |
| 6 | -2.653987 | -7.780611 | 0.062473 |
| 1 | -2.662998 | -8.810991 | 0.406060 |
| 6 | -2.120176 | -7.466861 | -1.210965 |
| 1 | -1.725510 | -8.260849 | -1.838472 |
| 6 | -2.096855 | -6.165233 | -1.661681 |
| 1 | -1.681770 | -5.936843 | -2.636118 |
| 6 | -2.588092 | -5.101979 | -0.855086 |
| 6 | -2.522874 | -3.730714 | -1.266659 |
| 6 | -3.003450 | -2.766218 | -0.396764 |
| 6 | -4.339573 | 1.501458 | 2.881621 |
| 6 | -4.237856 | 2.650343 | 2.029326 |
| 6 | -4.552386 | 2.603847 | 0.643462 |
| 1 | -4.884556 | 1.668055 | 0.211286 |
| 6 | -4.390157 | 3.711229 | -0.159597 |
| 1 | -4.611620 | 3.642253 | -1.219217 |
| 6 | -3.912787 | 4.929960 | 0.380419 |
| 1 | -3.779231 | 5.791639 | -0.268073 |
| 6 | -3.616504 | 5.017243 | 1.723247 |
| 1 | -3.248864 | 5.947025 | 2.150406 |
| 6 | -3.772365 | 3.892021 | 2.576831 |
| 6 | -3.451181 | 3.954595 | 3.956913 |
| 1 | -3.102081 | 4.895949 | 4.373248 |
| 6 | -3.543775 | 2.836114 | 4.746901 |
| 1 | -3.260869 | 2.897246 | 5.793368 |

| | | | |
|---|---|---|---|
| 6 | -3.974939 | 1.585031 | 4.223866 |
| 6 | -2.129134 | -3.321656 | -2.655511 |
| 6 | -0.766952 | -3.401636 | -3.103227 |
| 6 | 0.276665 | -3.931124 | -2.294679 |
| 1 | 0.055620 | -4.245493 | -1.284094 |
| 6 | 1.558151 | -4.064077 | -2.783075 |
| 1 | 2.330354 | -4.483413 | -2.148890 |
| 6 | 1.879756 | -3.638947 | -4.093439 |
| 1 | 2.897810 | -3.744052 | -4.456964 |
| 6 | 0.903462 | -3.088655 | -4.892306 |
| 1 | 1.135998 | -2.749732 | -5.898992 |
| 6 | -0.434940 | -2.970132 | -4.430001 |
| 6 | -1.460368 | -2.454040 | -5.260558 |
| 1 | -1.204170 | -2.101320 | -6.256383 |
| 6 | -2.759395 | -2.420419 | -4.822260 |
| 1 | -3.534668 | -2.044426 | -5.483442 |
| 6 | -3.125244 | -2.868921 | -3.523446 |
| 8 | -2.361407 | 0.964679 | -0.538101 |
| 8 | -0.628535 | -0.798368 | 0.301959 |
| 8 | -2.755320 | -0.479300 | 1.601712 |
| 8 | -2.941600 | -1.438509 | -0.771071 |
| 15 | -2.049371 | -0.364837 | 0.108508 |
| 6 | -4.602044 | -2.863266 | -3.155253 |
| 6 | -5.200334 | -1.440909 | -3.109655 |
| 6 | -5.437642 | -3.771896 | -4.085259 |
| 1 | -4.710386 | -3.278362 | -2.150064 |
| 6 | -6.675491 | -1.468793 | -2.683850 |
| 1 | -5.115216 | -0.975086 | -4.101172 |
| 1 | -4.618169 | -0.833924 | -2.412057 |
| 6 | -6.911881 | -3.798574 | -3.652907 |
| 1 | -5.369986 | -3.409634 | -5.119879 |
| 1 | -5.018121 | -4.785024 | -4.078725 |
| 6 | -7.507283 | -2.383981 | -3.593641 |
| 1 | -7.089776 | -0.452222 | -2.685404 |
| 1 | -6.739717 | -1.826767 | -1.646547 |
| 1 | -7.493475 | -4.429655 | -4.336224 |
| 1 | -6.985101 | -4.262104 | -2.658424 |
| 1 | -8.548361 | -2.422513 | -3.249953 |
| 1 | -7.526280 | -1.960313 | -4.608328 |
| 6 | -3.952419 | 0.361844 | 5.123018 |
| 6 | -2.496915 | -0.058675 | 5.438338 |
| 6 | -4.767161 | 0.527751 | 6.422650 |
| 1 | -4.404413 | -0.470574 | 4.575746 |
| 6 | -2.458687 | -1.335767 | 6.288918 |
| 1 | -1.994019 | 0.756440 | 5.977964 |
| 1 | -1.947289 | -0.207001 | 4.501878 |
| 6 | -4.726306 | -0.756216 | 7.265162 |
| 1 | -4.363319 | 1.359419 | 7.015970 |
| 1 | -5.802553 | 0.789212 | 6.173431 |
| 6 | -3.282664 | -1.180367 | 7.575590 |
| 1 | -1.420612 | -1.597798 | 6.526543 |
| 1 | -2.864641 | -2.169065 | 5.697964 |
| 1 | -5.291597 | -0.614499 | 8.194433 |
| 1 | -5.227915 | -1.562722 | 6.711665 |
| 1 | -3.275970 | -2.115876 | 8.148175 |
| 1 | -2.813625 | -0.417552 | 8.214118 |
| 1 | 0.498772 | -0.282190 | 1.590255 |
| 8 | 1.231175 | -0.086469 | 2.210804 |
| 1 | 1.216439 | 0.872907 | 2.310152 |

---

(*S*,*R*,*S*)-**[2a-3a]**$^{‡}$endo-*si*

---

Number of imaginary frequencies : 1
The smallest frequencies are : -329.4020 7.4598 12.9282 cm(-1)

Electronic energy : HF=-6614.0017194
Zero-point correction= 1.887858 (Hartree/Particle)
Thermal correction to Energy= 1.999173
Thermal correction to Enthalpy= 2.000118
Thermal correction to Gibbs Free Energy= 1.733237
Sum of electronic and zero-point Energies= -6612.113862
Sum of electronic and thermal Energies= -6612.002546
Sum of electronic and thermal Enthalpies= -6612.001602
Sum of electronic and thermal Free Energies= -6612.268483
............................................................
Cartesian Coordinates
............................................................

| | | | |
|---|---|---|---|
| 6 | 6.861357 | -0.967884 | -1.266709 |
| 6 | 7.045647 | -0.146561 | -0.142202 |
| 6 | 8.296662 | -0.137910 | 0.489938 |
| 6 | 9.333564 | -0.942216 | 0.013102 |
| 6 | 9.137565 | -1.761543 | -1.101757 |
| 6 | 7.898618 | -1.768549 | -1.746267 |
| 15 | 5.569019 | 0.798263 | 0.452107 |
| 6 | 5.896174 | 1.165725 | 2.225749 |
| 6 | 6.889147 | 2.062021 | 2.658897 |
| 6 | 7.096796 | 2.281437 | 4.020229 |
| 6 | 6.312160 | 1.614716 | 4.966192 |
| 6 | 5.314393 | 0.734976 | 4.545658 |
| 6 | 5.103584 | 0.515485 | 3.183026 |
| 46 | 3.572009 | -0.408617 | 0.021115 |
| 6 | 1.607736 | -0.706528 | -0.747669 |
| 6 | 1.789745 | 0.700246 | -0.697505 |
| 6 | 2.024073 | 1.430216 | -1.913281 |
| 15 | 3.990286 | -2.635801 | 0.707498 |
| 6 | 5.191189 | -2.865742 | 2.092875 |
| 6 | 6.573874 | -2.795412 | 1.854679 |
| 6 | 7.478296 | -2.821536 | 2.916167 |
| 6 | 7.019413 | -2.911429 | 4.231757 |
| 6 | 5.647189 | -2.986460 | 4.477893 |
| 6 | 4.738867 | -2.966152 | 3.418463 |
| 6 | 5.809206 | 2.445605 | -0.359096 |
| 6 | 5.325926 | 3.614412 | 0.254148 |
| 6 | 5.445171 | 4.851989 | -0.380342 |
| 6 | 6.033288 | 4.941918 | -1.644656 |
| 6 | 6.492817 | 3.782972 | -2.273196 |
| 6 | 6.383371 | 2.545195 | -1.636166 |
| 6 | 2.501409 | -3.548467 | 1.309668 |
| 6 | 2.307856 | -4.925398 | 1.112445 |
| 6 | 1.147352 | -5.544279 | 1.579418 |
| 6 | 0.172002 | -4.803447 | 2.252884 |
| 6 | 0.363664 | -3.437108 | 2.461076 |
| 6 | 1.519876 | -2.811662 | 1.990145 |
| 6 | 4.645953 | -3.688705 | -0.656947 |
| 6 | 4.325812 | -3.325253 | -1.975428 |
| 6 | 4.772609 | -4.091232 | -3.053162 |
| 6 | 5.551425 | -5.228955 | -2.826722 |
| 6 | 5.877516 | -5.597266 | -1.519416 |
| 6 | 5.427346 | -4.834175 | -0.440360 |
| 1 | 2.407402 | 0.815497 | -2.722157 |
| 1 | 0.948977 | -1.181259 | -0.033917 |
| 1 | 1.638926 | -1.187343 | -1.723296 |
| 1 | 4.860072 | 3.559258 | 1.232635 |

| | | | |
|---|---|---|---|
| 1 | 6.761361 | 1.657766 | -2.134075 |
| 1 | 5.072108 | 5.746059 | 0.111185 |
| 1 | 6.948940 | 3.840220 | -3.257650 |
| 1 | 6.129473 | 5.906235 | -2.134975 |
| 1 | 7.489273 | 2.600309 | 1.931611 |
| 1 | 4.319553 | -0.161024 | 2.860280 |
| 1 | 7.867912 | 2.975544 | 4.342878 |
| 1 | 4.695331 | 0.217925 | 5.272539 |
| 1 | 6.475021 | 1.788013 | 6.026328 |
| 1 | 8.459068 | 0.467149 | 1.374745 |
| 1 | 5.887316 | -1.009161 | -1.743925 |
| 1 | 10.293794 | -0.936361 | 0.521630 |
| 1 | 7.725476 | -2.413926 | -2.602368 |
| 1 | 9.943436 | -2.395456 | -1.460789 |
| 1 | 3.045425 | -5.512130 | 0.574695 |
| 1 | 1.637812 | -1.743941 | 2.143638 |
| 1 | 0.995416 | -6.605398 | 1.401833 |
| 1 | -0.400610 | -2.840109 | 2.946722 |
| 1 | -0.739295 | -5.285444 | 2.593192 |
| 1 | 6.948995 | -2.723758 | 0.840815 |
| 1 | 3.675857 | -3.022731 | 3.628602 |
| 1 | 8.542876 | -2.764006 | 2.708558 |
| 1 | 5.278410 | -3.061834 | 5.497125 |
| 1 | 7.725331 | -2.923677 | 5.057397 |
| 1 | 3.728371 | -2.435726 | -2.152605 |
| 1 | 5.691571 | -5.126636 | 0.570795 |
| 1 | 4.516656 | -3.794858 | -4.066561 |
| 1 | 6.482932 | -6.480763 | -1.337108 |
| 1 | 5.903962 | -5.824245 | -3.664310 |
| 1 | 1.411330 | 1.252269 | 0.154592 |
| 1 | -0.406287 | 1.253050 | -1.953530 |
| 6 | 0.088018 | 3.204065 | -2.627732 |
| 6 | 1.184549 | 3.801887 | -1.699924 |
| 1 | 0.277213 | 3.570205 | -3.636165 |
| 1 | -0.927727 | 3.480861 | -2.340051 |
| 7 | 0.241069 | 1.730805 | -2.636237 |
| 16 | 0.031100 | 0.915017 | -4.184694 |
| 8 | 0.711192 | -0.370541 | -4.012797 |
| 8 | 0.500734 | 1.850665 | -5.213463 |
| 6 | 2.409053 | 2.890109 | -1.990691 |
| 1 | 2.768610 | 3.091571 | -3.003729 |
| 1 | 3.228552 | 3.090187 | -1.298878 |
| 6 | 0.847964 | 3.754451 | -0.201116 |
| 6 | 1.801169 | 4.251291 | 0.704224 |
| 6 | -0.325229 | 3.194729 | 0.308709 |
| 6 | 1.607232 | 4.154423 | 2.079696 |
| 1 | 2.706402 | 4.711265 | 0.322456 |
| 6 | -0.518164 | 3.086240 | 1.689219 |
| 1 | -1.095542 | 2.796356 | -0.337660 |
| 6 | 0.443560 | 3.560343 | 2.580220 |
| 1 | 2.361662 | 4.539835 | 2.760330 |
| 1 | -1.425355 | 2.620719 | 2.050366 |
| 1 | 0.286290 | 3.467329 | 3.650403 |
| 6 | 1.398578 | 5.264802 | -2.109544 |
| 6 | 2.613806 | 5.759142 | -2.595548 |
| 6 | 0.314588 | 6.149732 | -1.993152 |
| 6 | 2.739640 | 7.102991 | -2.962881 |
| 1 | 3.478620 | 5.112065 | -2.677832 |
| 6 | 0.435804 | 7.485640 | -2.368283 |
| 1 | -0.625831 | 5.784435 | -1.593570 |
| 6 | 1.653025 | 7.969884 | -2.856963 |
| 1 | 3.693516 | 7.468084 | -3.334194 |

| | | | |
|---|---|---|---|
| 1 | -0.417867 | 8.151545 | -2.273830 |
| 1 | 1.752248 | 9.012357 | -3.145975 |
| 6 | -1.730964 | 0.625994 | -4.276717 |
| 6 | -2.669401 | 1.616385 | -4.721784 |
| 6 | -2.129648 | -0.594904 | -3.770723 |
| 6 | -2.341295 | 2.904602 | -5.227473 |
| 6 | -4.060672 | 1.257561 | -4.638493 |
| 6 | -3.498478 | -0.917073 | -3.697251 |
| 1 | -1.396225 | -1.307786 | -3.416332 |
| 6 | -3.328844 | 3.785618 | -5.615596 |
| 1 | -1.304701 | 3.193117 | -5.329197 |
| 6 | -5.047415 | 2.187930 | -5.062481 |
| 6 | -4.438260 | -0.010169 | -4.119395 |
| 1 | -3.785863 | -1.872971 | -3.285583 |
| 6 | -4.694598 | 3.430675 | -5.536591 |
| 1 | -3.048572 | 4.764015 | -5.994999 |
| 1 | -6.092646 | 1.899327 | -4.992133 |
| 1 | -5.496436 | -0.248311 | -4.050613 |
| 1 | -5.458098 | 4.136497 | -5.849320 |
| 6 | -4.160968 | 0.512495 | 1.661637 |
| 6 | -4.838496 | 1.684801 | 1.967073 |
| 6 | -6.187137 | 1.627247 | 2.441433 |
| 6 | -6.952772 | 2.794604 | 2.719306 |
| 1 | -6.504808 | 3.768417 | 2.558594 |
| 6 | -8.248588 | 2.697699 | 3.178167 |
| 1 | -8.817231 | 3.601530 | 3.378499 |
| 6 | -8.849519 | 1.432190 | 3.387639 |
| 1 | -9.871920 | 1.372469 | 3.749513 |
| 6 | -8.136497 | 0.284137 | 3.127293 |
| 1 | -8.586053 | -0.693792 | 3.281120 |
| 6 | -6.797120 | 0.351002 | 2.655849 |
| 6 | -6.042036 | -0.815219 | 2.394160 |
| 1 | -6.506319 | -1.783289 | 2.557920 |
| 6 | -4.743194 | -0.771859 | 1.915619 |
| 6 | -4.059331 | -2.070176 | 1.677309 |
| 6 | -4.138737 | -3.099492 | 2.595534 |
| 1 | -4.554099 | -2.907270 | 3.580644 |
| 6 | -3.694834 | -4.408147 | 2.276651 |
| 6 | -3.707180 | -5.457877 | 3.234432 |
| 1 | -4.026375 | -5.232692 | 4.248916 |
| 6 | -3.321938 | -6.735026 | 2.889380 |
| 1 | -3.330897 | -7.527353 | 3.632456 |
| 6 | -2.915998 | -7.018002 | 1.562526 |
| 1 | -2.612812 | -8.026662 | 1.296349 |
| 6 | -2.894980 | -6.022649 | 0.609198 |
| 1 | -2.569702 | -6.241207 | -0.402041 |
| 6 | -3.267305 | -4.691113 | 0.937594 |
| 6 | -3.196405 | -3.625087 | -0.015896 |
| 6 | -3.472403 | -2.342472 | 0.408524 |
| 6 | -4.133128 | 3.003300 | 1.909368 |
| 6 | -3.916863 | 3.658465 | 0.652667 |
| 6 | -4.236340 | 3.048720 | -0.592173 |
| 1 | -4.660878 | 2.051049 | -0.598470 |
| 6 | -3.989742 | 3.694340 | -1.783989 |
| 1 | -4.225465 | 3.205276 | -2.721371 |
| 6 | -3.425202 | 4.992859 | -1.796253 |
| 1 | -3.241865 | 5.489886 | -2.744784 |
| 6 | -3.108240 | 5.614791 | -0.608575 |
| 1 | -2.675913 | 6.612394 | -0.602240 |
| 6 | -3.326902 | 4.964838 | 0.635392 |
| 6 | -2.950580 | 5.564515 | 1.864259 |
| 1 | -2.495183 | 6.551471 | 1.850574 |

| | | | |
|---|---|---|---|
| 6 | -3.119452 | 4.891922 | 3.048083 |
| 1 | -2.785375 | 5.350288 | 3.973975 |
| 6 | -3.708410 | 3.597961 | 3.096131 |
| 6 | -2.995246 | -3.919861 | -1.469548 |
| 6 | -1.687766 | -3.951065 | -2.047008 |
| 6 | -0.517778 | -3.680347 | -1.284904 |
| 1 | -0.613583 | -3.391347 | -0.244423 |
| 6 | 0.728216 | -3.770554 | -1.865191 |
| 1 | 1.608647 | -3.582156 | -1.265705 |
| 6 | 0.872186 | -4.101506 | -3.233332 |
| 1 | 1.866611 | -4.160881 | -3.666175 |
| 6 | -0.241734 | -4.340976 | -4.005109 |
| 1 | -0.144541 | -4.586755 | -5.059757 |
| 6 | -1.542386 | -4.282879 | -3.435888 |
| 6 | -2.707555 | -4.551756 | -4.198629 |
| 1 | -2.601174 | -4.793140 | -5.253440 |
| 6 | -3.952344 | -4.512999 | -3.618070 |
| 1 | -4.827408 | -4.717689 | -4.226327 |
| 6 | -4.123782 | -4.204306 | -2.240280 |
| 8 | -1.707406 | 0.655939 | -1.062390 |
| 8 | -1.007857 | -1.015303 | 0.854913 |
| 8 | -2.871542 | 0.663220 | 1.224976 |
| 8 | -3.246161 | -1.270994 | -0.439888 |
| 15 | -2.045206 | -0.240801 | 0.094974 |
| 6 | -5.524267 | -4.117350 | -1.651230 |
| 6 | -6.148868 | -2.723547 | -1.904778 |
| 6 | -6.487413 | -5.225915 | -2.119410 |
| 1 | -5.437469 | -4.225981 | -0.565700 |
| 6 | -7.515429 | -2.590811 | -1.218281 |
| 1 | -6.262343 | -2.580661 | -2.989817 |
| 1 | -5.468449 | -1.942418 | -1.551553 |
| 6 | -7.846391 | -5.099073 | -1.413269 |
| 1 | -6.648000 | -5.158296 | -3.203683 |
| 1 | -6.042720 | -6.209397 | -1.925746 |
| 6 | -8.471421 | -3.714656 | -1.643606 |
| 1 | -7.953883 | -1.609693 | -1.437389 |
| 1 | -7.368062 | -2.628548 | -0.129716 |
| 1 | -8.526142 | -5.886331 | -1.761571 |
| 1 | -7.707405 | -5.258489 | -0.334534 |
| 1 | -9.422937 | -3.632062 | -1.104132 |
| 1 | -8.705348 | -3.601269 | -2.712251 |
| 6 | -3.792825 | 2.879122 | 4.432628 |
| 6 | -2.387980 | 2.501982 | 4.962197 |
| 6 | -4.582315 | 3.668403 | 5.498383 |
| 1 | -4.326048 | 1.936851 | 4.279366 |
| 6 | -2.483691 | 1.725733 | 6.283731 |
| 1 | -1.799410 | 3.417113 | 5.118528 |
| 1 | -1.854200 | 1.902970 | 4.215702 |
| 6 | -4.674923 | 2.883334 | 6.815042 |
| 1 | -4.090493 | 4.632129 | 5.688168 |
| 1 | -5.584907 | 3.893376 | 5.116654 |
| 6 | -3.282242 | 2.503385 | 7.339727 |
| 1 | -1.477872 | 1.493826 | 6.654276 |
| 1 | -2.976812 | 0.762028 | 6.094394 |
| 1 | -5.219968 | 3.469553 | 7.565241 |
| 1 | -5.260180 | 1.968480 | 6.644836 |
| 1 | -3.369001 | 1.916295 | 8.262409 |
| 1 | -2.734870 | 3.420946 | 7.600713 |
| 1 | -0.018521 | -0.221858 | 2.139083 |
| 8 | 0.591661 | 0.174715 | 2.797944 |
| 1 | 0.778514 | 1.051563 | 2.440674 |

---

(*S*,*S*,*S*)-**[2a-3a]**‡endo-*re*

---

Number of imaginary frequencies : 1
The smallest frequencies are : -332.8803 9.2868 14.3953 cm(-1)

Electronic energy : HF=-6613.9950609
Zero-point correction= 1.888483 (Hartree/Particle)
Thermal correction to Energy= 1.999409
Thermal correction to Enthalpy= 2.000353
Thermal correction to Gibbs Free Energy= 1.735395
Sum of electronic and zero-point Energies= -6612.106578
Sum of electronic and thermal Energies= -6611.995652
Sum of electronic and thermal Enthalpies= -6611.994708
Sum of electronic and thermal Free Energies= -6612.259666

Cartesian Coordinates

| | | | |
|---|---|---|---|
| 6 | -6.915164 | -0.068334 | -1.468871 |
| 6 | -6.916781 | 0.672026 | -0.274724 |
| 6 | -8.125130 | 0.828133 | 0.418062 |
| 6 | -9.301242 | 0.259337 | -0.074258 |
| 6 | -9.289828 | -0.469521 | -1.266166 |
| 6 | -8.092085 | -0.629746 | -1.965751 |
| 15 | -5.280200 | 1.325594 | 0.295808 |
| 6 | -5.440142 | 1.669540 | 2.094336 |
| 6 | -6.332462 | 2.611483 | 2.637849 |
| 6 | -6.379586 | 2.827164 | 4.014673 |
| 6 | -5.531041 | 2.111818 | 4.865640 |
| 6 | -4.634688 | 1.184276 | 4.334741 |
| 6 | -4.587235 | 0.963243 | 2.956494 |
| 46 | -3.495203 | -0.127487 | -0.288710 |
| 6 | -1.587216 | -0.567541 | -1.119435 |
| 6 | -1.575404 | 0.820125 | -0.814787 |
| 6 | -1.600563 | 1.786493 | -1.876414 |
| 15 | -4.306682 | -2.296273 | 0.225641 |
| 6 | -5.483731 | -2.349040 | 1.648375 |
| 6 | -6.855568 | -2.099987 | 1.484647 |
| 6 | -7.697167 | -2.012423 | 2.594771 |
| 6 | -7.183433 | -2.163084 | 3.883555 |
| 6 | -5.819311 | -2.408032 | 4.055942 |
| 6 | -4.975422 | -2.501432 | 2.949682 |
| 6 | -5.275652 | 3.008087 | -0.487503 |
| 6 | -4.889991 | 4.163860 | 0.209159 |
| 6 | -4.814747 | 5.398359 | -0.442282 |
| 6 | -5.112576 | 5.497729 | -1.801818 |
| 6 | -5.479876 | 4.349929 | -2.509807 |
| 6 | -5.560560 | 3.119091 | -1.859669 |
| 6 | -3.062464 | -3.564557 | 0.743538 |
| 6 | -1.825163 | -3.120437 | 1.224375 |
| 6 | -0.859127 | -4.028483 | 1.661828 |
| 6 | -1.129811 | -5.396482 | 1.642927 |
| 6 | -2.371114 | -5.849398 | 1.180325 |
| 6 | -3.330185 | -4.943420 | 0.725789 |
| 6 | -5.182901 | -3.114641 | -1.175179 |
| 6 | -4.769586 | -2.770543 | -2.472538 |
| 6 | -5.349435 | -3.375434 | -3.588257 |
| 6 | -6.360605 | -4.325440 | -3.421789 |
| 6 | -6.782450 | -4.670869 | -2.135854 |
| 6 | -6.195148 | -4.073782 | -1.018625 |
| 1 | -1.968468 | 1.386243 | -2.817852 |
| 1 | -1.005618 | -1.243192 | -0.509092 |

| | | | |
|---|---|---|---|
| 1 | -1.676989 | -0.854970 | -2.164179 |
| 1 | -4.641237 | 4.106556 | 1.262355 |
| 1 | -5.854238 | 2.241497 | -2.427145 |
| 1 | -4.514054 | 6.280372 | 0.116190 |
| 1 | -5.709636 | 4.412345 | -3.569881 |
| 1 | -5.054865 | 6.457951 | -2.306349 |
| 1 | -6.973258 | 3.194833 | 1.983700 |
| 1 | -3.876859 | 0.248947 | 2.552252 |
| 1 | -7.072588 | 3.557784 | 4.422586 |
| 1 | -3.965756 | 0.629615 | 4.985987 |
| 1 | -5.566406 | 2.284666 | 5.937783 |
| 1 | -8.152657 | 1.360295 | 1.361424 |
| 1 | -5.979919 | -0.240006 | -1.992817 |
| 1 | -10.227321 | 0.379733 | 0.481302 |
| 1 | -8.063180 | -1.211667 | -2.882167 |
| 1 | -10.205807 | -0.917153 | -1.641312 |
| 1 | -1.615186 | -2.063858 | 1.266705 |
| 1 | -4.278482 | -5.314943 | 0.351160 |
| 1 | 0.099750 | -3.652208 | 1.999438 |
| 1 | -2.587346 | -6.914205 | 1.157483 |
| 1 | -0.375122 | -6.103229 | 1.974881 |
| 1 | -7.274043 | -1.971852 | 0.493632 |
| 1 | -3.916727 | -2.685634 | 3.101835 |
| 1 | -8.755114 | -1.817491 | 2.444560 |
| 1 | -5.406708 | -2.524180 | 5.054209 |
| 1 | -7.839197 | -2.088144 | 4.746416 |
| 1 | -3.990378 | -2.025100 | -2.600876 |
| 1 | -6.534758 | -4.348502 | -0.025311 |
| 1 | -5.012732 | -3.102800 | -4.584499 |
| 1 | -7.568647 | -5.408356 | -2.000151 |
| 1 | -6.817809 | -4.793024 | -4.289294 |
| 1 | -1.158727 | 1.134605 | 0.132908 |
| 1 | 0.780604 | 1.233067 | -1.752079 |
| 6 | 0.636182 | 3.342502 | -1.912488 |
| 6 | -0.535450 | 3.927069 | -1.065004 |
| 1 | 0.721331 | 3.965306 | -2.800108 |
| 1 | 1.596450 | 3.343745 | -1.394363 |
| 7 | 0.287832 | 1.967062 | -2.336487 |
| 16 | 0.542689 | 1.546479 | -4.015488 |
| 8 | -0.192658 | 0.290351 | -4.182271 |
| 8 | 0.156882 | 2.729258 | -4.794626 |
| 6 | -1.783219 | 3.279197 | -1.721854 |
| 1 | -1.917394 | 3.698665 | -2.722770 |
| 1 | -2.689427 | 3.480713 | -1.149421 |
| 6 | -0.505780 | 3.593919 | 0.435176 |
| 6 | -1.543130 | 4.102410 | 1.233275 |
| 6 | 0.462577 | 2.787309 | 1.035946 |
| 6 | -1.637392 | 3.781526 | 2.584833 |
| 1 | -2.285309 | 4.752882 | 0.784603 |
| 6 | 0.368996 | 2.460817 | 2.391821 |
| 1 | 1.279546 | 2.368278 | 0.462694 |
| 6 | -0.678150 | 2.950905 | 3.171194 |
| 1 | -2.459135 | 4.172425 | 3.178729 |
| 1 | 1.115320 | 1.808201 | 2.823915 |
| 1 | -0.746662 | 2.685912 | 4.221843 |
| 6 | -0.475801 | 5.454531 | -1.207072 |
| 6 | -1.481855 | 6.214542 | -1.812184 |
| 6 | 0.657577 | 6.117440 | -0.706908 |
| 6 | -1.356576 | 7.603670 | -1.920751 |
| 1 | -2.378680 | 5.738809 | -2.190238 |
| 6 | 0.788281 | 7.498865 | -0.825943 |
| 1 | 1.430882 | 5.544720 | -0.204041 |

| | | | |
|---|---|---|---|
| 6 | -0.221343 | 8.250659 | -1.434790 |
| 1 | -2.152957 | 8.176689 | -2.388220 |
| 1 | 1.675155 | 7.991615 | -0.436362 |
| 1 | -0.124705 | 9.329160 | -1.522346 |
| 6 | 2.296304 | 1.201127 | -4.158932 |
| 6 | 3.288044 | 2.226976 | -4.312804 |
| 6 | 2.628420 | -0.136533 | -4.055034 |
| 6 | 3.034035 | 3.622874 | -4.417751 |
| 6 | 4.656849 | 1.788022 | -4.360425 |
| 6 | 3.974866 | -0.541042 | -4.141426 |
| 1 | 1.853487 | -0.878342 | -3.908736 |
| 6 | 4.070158 | 4.527569 | -4.502009 |
| 1 | 2.015942 | 3.981651 | -4.456512 |
| 6 | 5.697905 | 2.750820 | -4.455125 |
| 6 | 4.961396 | 0.403004 | -4.295757 |
| 1 | 4.216043 | -1.594935 | -4.060905 |
| 6 | 5.416256 | 4.096150 | -4.509148 |
| 1 | 3.844537 | 5.587719 | -4.571502 |
| 1 | 6.725501 | 2.397849 | -4.475009 |
| 1 | 6.003379 | 0.100873 | -4.348078 |
| 1 | 6.219330 | 4.824444 | -4.571119 |
| 6 | 3.455896 | 0.130343 | 2.456694 |
| 6 | 4.222412 | 1.185298 | 2.953897 |
| 6 | 5.033750 | 0.998656 | 4.109152 |
| 6 | 5.843265 | 2.032166 | 4.660690 |
| 1 | 5.844483 | 3.008074 | 4.188475 |
| 6 | 6.607962 | 1.803692 | 5.782812 |
| 1 | 7.215372 | 2.605649 | 6.193099 |
| 6 | 6.614642 | 0.531321 | 6.408377 |
| 1 | 7.222581 | 0.367961 | 7.293571 |
| 6 | 5.858398 | -0.493032 | 5.887811 |
| 1 | 5.866074 | -1.476739 | 6.350705 |
| 6 | 5.055295 | -0.289528 | 4.731069 |
| 6 | 4.334200 | -1.347016 | 4.135561 |
| 1 | 4.464142 | -2.338843 | 4.555579 |
| 6 | 3.534921 | -1.197258 | 3.007099 |
| 6 | 2.994559 | -2.462227 | 2.409689 |
| 6 | 2.661998 | -3.541799 | 3.211101 |
| 1 | 2.547753 | -3.401135 | 4.281644 |
| 6 | 2.498370 | -4.848641 | 2.683779 |
| 6 | 2.146819 | -5.947620 | 3.514298 |
| 1 | 1.940119 | -5.760417 | 4.564941 |
| 6 | 2.076153 | -7.225889 | 3.004763 |
| 1 | 1.806348 | -8.056828 | 3.650188 |
| 6 | 2.366380 | -7.461181 | 1.638434 |
| 1 | 2.323528 | -8.473162 | 1.245958 |
| 6 | 2.704026 | -6.416358 | 0.804959 |
| 1 | 2.928411 | -6.598279 | -0.240205 |
| 6 | 2.754272 | -5.082942 | 1.291841 |
| 6 | 3.012378 | -3.963706 | 0.442566 |
| 6 | 3.025103 | -2.698361 | 0.998539 |
| 6 | 4.254180 | 2.468556 | 2.175164 |
| 6 | 3.561215 | 3.629753 | 2.649562 |
| 6 | 2.911234 | 3.670073 | 3.915025 |
| 1 | 2.937156 | 2.788346 | 4.545311 |
| 6 | 2.247446 | 4.798739 | 4.340953 |
| 1 | 1.754820 | 4.801595 | 5.309106 |
| 6 | 2.187316 | 5.950734 | 3.520757 |
| 1 | 1.648838 | 6.830515 | 3.861083 |
| 6 | 2.811970 | 5.951071 | 2.294580 |
| 1 | 2.777051 | 6.832579 | 1.659377 |
| 6 | 3.517431 | 4.807269 | 1.831636 |

| | | | |
|---|---|---|---|
| 6 | 4.176817 | 4.793527 | 0.574912 |
| 1 | 4.130577 | 5.679313 | -0.054377 |
| 6 | 4.880700 | 3.685327 | 0.168508 |
| 1 | 5.395840 | 3.699224 | -0.787866 |
| 6 | 4.945899 | 2.509270 | 0.965057 |
| 6 | 3.443209 | -4.116484 | -0.986219 |
| 6 | 4.865404 | -4.071588 | -1.224695 |
| 6 | 5.818693 | -3.968296 | -0.171649 |
| 1 | 5.472508 | -3.924971 | 0.854048 |
| 6 | 7.171548 | -3.919738 | -0.431331 |
| 1 | 7.874819 | -3.838812 | 0.392678 |
| 6 | 7.652883 | -3.964132 | -1.760578 |
| 1 | 8.721017 | -3.918223 | -1.952840 |
| 6 | 6.761641 | -4.073615 | -2.804315 |
| 1 | 7.115886 | -4.117521 | -3.831455 |
| 6 | 5.362968 | -4.140697 | -2.566004 |
| 6 | 4.432989 | -4.278200 | -3.629205 |
| 1 | 4.802153 | -4.335502 | -4.650554 |
| 6 | 3.088574 | -4.350476 | -3.370451 |
| 1 | 2.399651 | -4.470342 | -4.200341 |
| 6 | 2.560536 | -4.271221 | -2.047324 |
| 8 | 1.824636 | 0.278649 | -0.961804 |
| 8 | 0.699991 | -1.124161 | 0.968973 |
| 8 | 2.654767 | 0.491447 | 1.404898 |
| 8 | 3.117498 | -1.621366 | 0.142648 |
| 15 | 1.901416 | -0.500765 | 0.321482 |
| 6 | 1.054851 | -4.401877 | -1.876265 |
| 6 | 0.597577 | -5.822063 | -2.299773 |
| 6 | 0.281997 | -3.319355 | -2.668265 |
| 1 | 0.805409 | -4.272280 | -0.817099 |
| 6 | -0.923205 | -5.999902 | -2.224001 |
| 1 | 0.926562 | -6.014230 | -3.330385 |
| 1 | 1.098341 | -6.564843 | -1.668719 |
| 6 | -1.238066 | -3.530350 | -2.612207 |
| 1 | 0.596494 | -3.338372 | -3.720252 |
| 1 | 0.542237 | -2.328274 | -2.279011 |
| 6 | -1.647029 | -4.938317 | -3.058923 |
| 1 | -1.196096 | -7.008834 | -2.559365 |
| 1 | -1.245477 | -5.913874 | -1.180944 |
| 1 | -1.732173 | -2.780272 | -3.239856 |
| 1 | -1.589878 | -3.367255 | -1.588143 |
| 1 | -2.733958 | -5.055261 | -2.972474 |
| 1 | -1.395104 | -5.074367 | -4.121383 |
| 6 | 5.804830 | 1.349980 | 0.498521 |
| 6 | 7.306789 | 1.718257 | 0.512523 |
| 6 | 5.413162 | 0.782660 | -0.880264 |
| 1 | 5.684877 | 0.533126 | 1.214968 |
| 6 | 8.174941 | 0.498483 | 0.167575 |
| 1 | 7.492013 | 2.522041 | -0.214210 |
| 1 | 7.582187 | 2.114009 | 1.497560 |
| 6 | 6.255780 | -0.462685 | -1.180128 |
| 1 | 5.585643 | 1.540970 | -1.655197 |
| 1 | 4.347994 | 0.544869 | -0.906834 |
| 6 | 7.755632 | -0.139392 | -1.165776 |
| 1 | 9.234475 | 0.783582 | 0.143268 |
| 1 | 8.068545 | -0.248275 | 0.967443 |
| 1 | 5.967353 | -0.903308 | -2.137517 |
| 1 | 6.038994 | -1.225935 | -0.424461 |
| 1 | 8.341519 | -1.047071 | -1.354088 |
| 1 | 7.982838 | 0.559744 | -1.985478 |
| 1 | -0.628399 | -0.445948 | 2.048200 |
| 8 | -1.422494 | -0.181720 | 2.558260 |

| | | | |
|---|---|---|---|
| 1 | -1.503813 | 0.764800 | 2.389167 |

---

(*S*,*S*,*S*)-**[2a-3a]**‡exo-*si*

---

Number of imaginary frequencies : 1
The smallest frequencies are : -291.9826 5.5770 11.6518 cm(-1)

Electronic energy : HF=-6614.0020383
Zero-point correction= 1.888791 (Hartree/Particle)
Thermal correction to Energy= 1.999496
Thermal correction to Enthalpy= 2.000441
Thermal correction to Gibbs Free Energy= 1.735028
Sum of electronic and zero-point Energies= -6612.113247
Sum of electronic and thermal Energies= -6612.002542
Sum of electronic and thermal Enthalpies= -6612.001598
Sum of electronic and thermal Free Energies= -6612.267010

.............................................................

Cartesian Coordinates

.............................................................

| | | | |
|---|---|---|---|
| 6 | 6.991845 | 0.718249 | -1.149491 |
| 6 | 6.828635 | -0.238117 | -0.134341 |
| 6 | 7.901601 | -0.490270 | 0.732931 |
| 6 | 9.103821 | 0.204640 | 0.589106 |
| 6 | 9.254190 | 1.156587 | -0.422897 |
| 6 | 8.195467 | 1.408569 | -1.297314 |
| 15 | 5.161671 | -1.030350 | 0.022056 |
| 6 | 5.103154 | -1.698341 | 1.736374 |
| 6 | 5.883720 | -2.787534 | 2.166296 |
| 6 | 5.792111 | -3.248808 | 3.478794 |
| 6 | 4.921573 | -2.629742 | 4.381114 |
| 6 | 4.147037 | -1.547152 | 3.965847 |
| 6 | 4.237114 | -1.084185 | 2.651766 |
| 46 | 3.423191 | 0.470429 | -0.653454 |
| 6 | 1.704288 | 1.017382 | -1.814812 |
| 6 | 1.949477 | -0.311357 | -2.231532 |
| 6 | 1.295336 | -1.444441 | -1.618792 |
| 15 | 3.894286 | 2.555706 | 0.393740 |
| 6 | 4.656341 | 2.404960 | 2.068501 |
| 6 | 6.005862 | 2.038407 | 2.207350 |
| 6 | 6.541804 | 1.769989 | 3.466472 |
| 6 | 5.739439 | 1.853606 | 4.606152 |
| 6 | 4.397913 | 2.218176 | 4.477732 |
| 6 | 3.858595 | 2.494257 | 3.220222 |
| 6 | 5.363334 | -2.535593 | -1.041349 |
| 6 | 4.837176 | -3.781527 | -0.664453 |
| 6 | 4.922532 | -4.880159 | -1.523595 |
| 6 | 5.517319 | -4.749489 | -2.778878 |
| 6 | 6.026820 | -3.508515 | -3.171258 |
| 6 | 5.953558 | -2.413373 | -2.310873 |
| 6 | 2.525204 | 3.768237 | 0.676035 |
| 6 | 2.715383 | 5.158176 | 0.598693 |
| 6 | 1.645174 | 6.027537 | 0.816546 |
| 6 | 0.373647 | 5.525643 | 1.113187 |
| 6 | 0.178616 | 4.146244 | 1.195372 |
| 6 | 1.248359 | 3.275596 | 0.978277 |
| 6 | 5.095551 | 3.538436 | -0.603605 |
| 6 | 4.995835 | 3.452550 | -2.002207 |
| 6 | 5.853208 | 4.185204 | -2.822977 |
| 6 | 6.829534 | 5.009270 | -2.256545 |
| 6 | 6.932496 | 5.106295 | -0.867424 |
| 6 | 6.067614 | 4.381375 | -0.044879 |

| | | | |
|---|---|---|---|
| 1 | 1.029930 | -1.309093 | -0.574472 |
| 1 | 1.937774 | 1.816620 | -2.508985 |
| 1 | 0.877198 | 1.224876 | -1.142113 |
| 1 | 4.346645 | -3.896955 | 0.294610 |
| 1 | 6.365481 | -1.461482 | -2.631347 |
| 1 | 4.509630 | -5.835540 | -1.213269 |
| 1 | 6.491045 | -3.393322 | -4.146850 |
| 1 | 5.579871 | -5.603618 | -3.447184 |
| 1 | 6.552214 | -3.286345 | 1.471514 |
| 1 | 3.629035 | -0.241623 | 2.349483 |
| 1 | 6.397871 | -4.093412 | 3.795417 |
| 1 | 3.463292 | -1.060828 | 4.654291 |
| 1 | 4.846930 | -2.994756 | 5.401777 |
| 1 | 7.795201 | -1.202843 | 1.542987 |
| 1 | 6.158787 | 0.952923 | -1.804011 |
| 1 | 9.921804 | 0.007199 | 1.276622 |
| 1 | 8.288388 | 2.161366 | -2.074318 |
| 1 | 10.187538 | 1.703420 | -0.522894 |
| 1 | 3.690613 | 5.567071 | 0.356691 |
| 1 | 1.078177 | 2.212961 | 1.051261 |
| 1 | 1.803910 | 7.100049 | 0.744196 |
| 1 | -0.801916 | 3.733774 | 1.407865 |
| 1 | -0.460895 | 6.203438 | 1.269841 |
| 1 | 6.642512 | 1.960103 | 1.334703 |
| 1 | 2.812102 | 2.768371 | 3.140047 |
| 1 | 7.586566 | 1.484803 | 3.550115 |
| 1 | 3.763481 | 2.287026 | 5.356926 |
| 1 | 6.155026 | 1.633588 | 5.585353 |
| 1 | 4.248674 | 2.800369 | -2.445428 |
| 1 | 6.156293 | 4.470410 | 1.032664 |
| 1 | 5.762434 | 4.107502 | -3.902805 |
| 1 | 7.685436 | 5.749047 | -0.419893 |
| 1 | 7.503795 | 5.574049 | -2.894211 |
| 1 | 2.431346 | -0.491623 | -3.190565 |
| 1 | -1.146156 | -1.233072 | -1.387690 |
| 6 | -0.705168 | -3.173185 | -2.026497 |
| 6 | 0.612500 | -3.823655 | -1.506764 |
| 1 | -0.894693 | -3.567401 | -3.022507 |
| 1 | -1.573955 | -3.385766 | -1.403876 |
| 7 | -0.523565 | -1.697555 | -2.108397 |
| 16 | -0.959754 | -0.990163 | -3.658940 |
| 8 | -0.842250 | 0.451641 | -3.464473 |
| 8 | -0.134608 | -1.682681 | -4.654710 |
| 6 | 1.695429 | -2.840742 | -2.011549 |
| 1 | 1.783828 | -2.881760 | -3.102049 |
| 1 | 2.669065 | -3.077684 | -1.581339 |
| 6 | 0.726387 | -3.899540 | 0.026164 |
| 6 | 1.723940 | -4.714025 | 0.589847 |
| 6 | -0.023336 | -3.085435 | 0.881372 |
| 6 | 1.984054 | -4.686619 | 1.958777 |
| 1 | 2.305690 | -5.365010 | -0.054485 |
| 6 | 0.241966 | -3.050755 | 2.252297 |
| 1 | -0.810724 | -2.445515 | 0.502273 |
| 6 | 1.247370 | -3.843781 | 2.796553 |
| 1 | 2.770035 | -5.314807 | 2.370038 |
| 1 | -0.334001 | -2.384268 | 2.880406 |
| 1 | 1.453456 | -3.804835 | 3.861732 |
| 6 | 0.709118 | -5.224995 | -2.114047 |
| 6 | 1.766484 | -5.628150 | -2.936804 |
| 6 | -0.312673 | -6.148574 | -1.837975 |
| 6 | 1.800511 | -6.918913 | -3.474612 |
| 1 | 2.579907 | -4.947331 | -3.157332 |

| | | | |
|---|---|---|---|
| 6 | -0.286710 | -7.430750 | -2.382405 |
| 1 | -1.129820 | -5.860125 | -1.182064 |
| 6 | 0.773956 | -7.822822 | -3.204861 |
| 1 | 2.634866 | -7.211932 | -4.106399 |
| 1 | -1.090765 | -8.126641 | -2.159493 |
| 1 | 0.799211 | -8.823875 | -3.626052 |
| 6 | -2.703199 | -1.407322 | -3.803085 |
| 6 | -3.199748 | -2.587381 | -4.454381 |
| 6 | -3.537684 | -0.527734 | -3.136192 |
| 6 | -2.415494 | -3.539941 | -5.164517 |
| 6 | -4.619536 | -2.811806 | -4.371326 |
| 6 | -4.923320 | -0.772611 | -3.072845 |
| 1 | -3.136429 | 0.350629 | -2.648984 |
| 6 | -2.995292 | -4.658759 | -5.723023 |
| 1 | -1.351953 | -3.380387 | -5.275222 |
| 6 | -5.178853 | -3.974595 | -4.966672 |
| 6 | -5.447918 | -1.886591 | -3.682004 |
| 1 | -5.554136 | -0.083739 | -2.526645 |
| 6 | -4.386482 | -4.887027 | -5.623066 |
| 1 | -2.369654 | -5.372484 | -6.251040 |
| 1 | -6.251282 | -4.130002 | -4.885097 |
| 1 | -6.515472 | -2.082939 | -3.634520 |
| 1 | -4.824422 | -5.775619 | -6.067891 |
| 6 | -3.368569 | 0.314768 | 2.826321 |
| 6 | -3.754961 | -0.694689 | 3.694067 |
| 6 | -4.847108 | -0.464776 | 4.597677 |
| 6 | -5.324662 | -1.465975 | 5.490459 |
| 1 | -4.849384 | -2.439817 | 5.494380 |
| 6 | -6.384291 | -1.215319 | 6.334792 |
| 1 | -6.734340 | -1.996585 | 7.003626 |
| 6 | -7.024172 | 0.047877 | 6.338944 |
| 1 | -7.857787 | 0.230389 | 7.010964 |
| 6 | -6.588175 | 1.037114 | 5.487728 |
| 1 | -7.070881 | 2.011136 | 5.476191 |
| 6 | -5.498605 | 0.807978 | 4.604103 |
| 6 | -5.038690 | 1.812809 | 3.722982 |
| 1 | -5.544230 | 2.773815 | 3.721295 |
| 6 | -3.989965 | 1.603861 | 2.845351 |
| 6 | -3.613434 | 2.735664 | 1.957391 |
| 6 | -3.536757 | 4.019952 | 2.461128 |
| 1 | -3.523554 | 4.171563 | 3.536448 |
| 6 | -3.552402 | 5.149373 | 1.607112 |
| 6 | -3.494437 | 6.472000 | 2.126103 |
| 1 | -3.340226 | 6.604548 | 3.193911 |
| 6 | -3.654020 | 7.562114 | 1.300183 |
| 1 | -3.610839 | 8.568369 | 1.706614 |
| 6 | -3.909992 | 7.367643 | -0.078309 |
| 1 | -4.080727 | 8.226906 | -0.720536 |
| 6 | -3.951942 | 6.097483 | -0.611340 |
| 1 | -4.164616 | 5.957735 | -1.663651 |
| 6 | -3.725259 | 4.951792 | 0.197518 |
| 6 | -3.663806 | 3.622815 | -0.343521 |
| 6 | -3.545414 | 2.564129 | 0.542380 |
| 6 | -3.023629 | -1.998335 | 3.810183 |
| 6 | -2.109853 | -2.144796 | 4.908902 |
| 6 | -1.717069 | -1.046106 | 5.725278 |
| 1 | -2.128098 | -0.064076 | 5.519064 |
| 6 | -0.811275 | -1.208979 | 6.749766 |
| 1 | -0.517180 | -0.352830 | 7.350449 |
| 6 | -0.255005 | -2.481961 | 7.024748 |
| 1 | 0.458800 | -2.596090 | 7.835825 |
| 6 | -0.619462 | -3.568382 | 6.262203 |

| | | | |
|---|---|---|---|
| 1 | -0.198170 | -4.550906 | 6.461187 |
| 6 | -1.545492 | -3.431260 | 5.192500 |
| 6 | -1.917613 | -4.528633 | 4.377473 |
| 1 | -1.505764 | -5.510976 | 4.594244 |
| 6 | -2.750045 | -4.348461 | 3.300598 |
| 1 | -2.983385 | -5.200102 | 2.671939 |
| 6 | -3.303145 | -3.078763 | 2.976545 |
| 6 | -3.860512 | 3.376888 | -1.810256 |
| 6 | -5.115301 | 2.792795 | -2.223028 |
| 6 | -6.139634 | 2.426671 | -1.303100 |
| 1 | -5.977626 | 2.571522 | -0.242083 |
| 6 | -7.340123 | 1.901789 | -1.733605 |
| 1 | -8.100246 | 1.634408 | -1.004957 |
| 6 | -7.592098 | 1.711058 | -3.112806 |
| 1 | -8.542678 | 1.299519 | -3.439994 |
| 6 | -6.623866 | 2.046652 | -4.031821 |
| 1 | -6.795883 | 1.899807 | -5.095129 |
| 6 | -5.376897 | 2.586671 | -3.617333 |
| 6 | -4.373685 | 2.931746 | -4.558387 |
| 1 | -4.556202 | 2.750288 | -5.614542 |
| 6 | -3.196768 | 3.495039 | -4.142198 |
| 1 | -2.446671 | 3.758320 | -4.880719 |
| 6 | -2.920135 | 3.755844 | -2.768138 |
| 8 | -2.362990 | -0.940802 | -0.342426 |
| 8 | -0.874694 | 1.130502 | 0.201419 |
| 8 | -2.274462 | 0.092417 | 2.025224 |
| 8 | -3.489152 | 1.268667 | 0.058096 |
| 15 | -2.147348 | 0.359412 | 0.397344 |
| 6 | -1.613346 | 4.464853 | -2.442536 |
| 6 | -1.558334 | 5.876332 | -3.081448 |
| 6 | -0.379487 | 3.648147 | -2.887683 |
| 1 | -1.540503 | 4.588797 | -1.357404 |
| 6 | -0.263389 | 6.609776 | -2.710946 |
| 1 | -1.614954 | 5.780621 | -4.174468 |
| 1 | -2.424663 | 6.468716 | -2.775574 |
| 6 | 0.927816 | 4.387689 | -2.568726 |
| 1 | -0.434884 | 3.454348 | -3.966977 |
| 1 | -0.401805 | 2.673524 | -2.397537 |
| 6 | 0.965082 | 5.805597 | -3.152090 |
| 1 | -0.255231 | 7.608662 | -3.165866 |
| 1 | -0.231061 | 6.754021 | -1.622491 |
| 1 | 1.784621 | 3.815931 | -2.943431 |
| 1 | 1.045124 | 4.446620 | -1.484054 |
| 1 | 1.888309 | 6.312824 | -2.845177 |
| 1 | 0.982805 | 5.751967 | -4.250891 |
| 6 | -4.159803 | -2.909115 | 1.730682 |
| 6 | -5.679202 | -2.976008 | 2.000316 |
| 6 | -3.797983 | -3.888990 | 0.597071 |
| 1 | -3.947486 | -1.910248 | 1.340040 |
| 6 | -6.462416 | -2.627254 | 0.723932 |
| 1 | -5.941062 | -3.988907 | 2.339443 |
| 1 | -5.952655 | -2.292380 | 2.809255 |
| 6 | -4.556275 | -3.533476 | -0.686877 |
| 1 | -4.056651 | -4.916607 | 0.888771 |
| 1 | -2.716761 | -3.868538 | 0.429552 |
| 6 | -6.073781 | -3.538843 | -0.450216 |
| 1 | -7.541488 | -2.687700 | 0.913442 |
| 1 | -6.245536 | -1.583667 | 0.454200 |
| 1 | -4.297459 | -4.235556 | -1.489661 |
| 1 | -4.235205 | -2.539493 | -1.014407 |
| 1 | -6.599725 | -3.227981 | -1.361184 |
| 1 | -6.402411 | -4.565689 | -0.231921 |

| | | | |
|---|---|---|---|
| 1 | 0.435788 | 0.343414 | 1.225791 |
| 8 | 1.198761 | -0.049310 | 1.696248 |
| 1 | 1.918454 | -0.021058 | 1.042833 |

----------------------------------------------------------------------------------------------------------------

(*S*,*S*,*S*)-**[2a-3a]**‡exo-*re*

----------------------------------------------------------------------------------------------------------------

Number of imaginary frequencies : 1
The smallest frequencies are : -311.5051 13.3659 14.9728 cm(-1)

Electronic energy : HF=-6614.0020918
Zero-point correction= 1.888316 (Hartree/Particle)
Thermal correction to Energy= 1.999302
Thermal correction to Enthalpy= 2.000246
Thermal correction to Gibbs Free Energy= 1.736840
Sum of electronic and zero-point Energies= -6612.113776
Sum of electronic and thermal Energies= -6612.002790
Sum of electronic and thermal Enthalpies= -6612.001846
Sum of electronic and thermal Free Energies= -6612.265252

.............................................................

Cartesian Coordinates

.............................................................

| | | | |
|---|---|---|---|
| 6 | 3.395069 | -0.256113 | 2.675051 |
| 6 | 4.667888 | 0.145506 | 2.237350 |
| 6 | 5.753812 | 0.034855 | 3.117782 |
| 6 | 5.565635 | -0.467354 | 4.406557 |
| 6 | 4.295054 | -0.867173 | 4.830037 |
| 6 | 3.205412 | -0.760845 | 3.962645 |
| 15 | 4.779031 | 0.775051 | 0.508966 |
| 6 | 6.551672 | 0.617265 | -0.001293 |
| 6 | 7.573474 | 1.371597 | 0.602476 |
| 6 | 8.896062 | 1.239783 | 0.182268 |
| 6 | 9.217244 | 0.368844 | -0.863005 |
| 6 | 8.207944 | -0.362365 | -1.488628 |
| 6 | 6.884486 | -0.237801 | -1.060516 |
| 46 | 3.275884 | -0.422994 | -0.894861 |
| 6 | 1.716437 | -0.723413 | -2.349779 |
| 6 | 1.821914 | 0.677415 | -2.142084 |
| 6 | 0.956298 | 1.333424 | -1.192242 |
| 15 | 3.906912 | -2.687179 | -0.540969 |
| 6 | 5.479223 | -3.117133 | 0.338483 |
| 6 | 5.534907 | -3.025552 | 1.739161 |
| 6 | 6.726539 | -3.276187 | 2.418725 |
| 6 | 7.883615 | -3.614554 | 1.714074 |
| 6 | 7.838596 | -3.708214 | 0.322490 |
| 6 | 6.646405 | -3.463299 | -0.360362 |
| 6 | 4.732687 | 2.613499 | 0.742280 |
| 6 | 4.876726 | 3.418164 | -0.401427 |
| 6 | 4.957179 | 4.805346 | -0.292009 |
| 6 | 4.898041 | 5.412451 | 0.967077 |
| 6 | 4.732786 | 4.623893 | 2.106427 |
| 6 | 4.643224 | 3.232141 | 1.996855 |
| 6 | 4.151782 | -3.592080 | -2.137215 |
| 6 | 4.144550 | -4.990814 | -2.248733 |
| 6 | 4.415416 | -5.607658 | -3.470216 |
| 6 | 4.716836 | -4.837383 | -4.597209 |
| 6 | 4.736973 | -3.445493 | -4.496595 |
| 6 | 4.450937 | -2.828723 | -3.276194 |
| 6 | 2.667413 | -3.613772 | 0.462323 |
| 6 | 1.468004 | -2.975527 | 0.808327 |
| 6 | 0.535456 | -3.613583 | 1.628828 |
| 6 | 0.774931 | -4.907914 | 2.085464 |

| | | | |
|---|---|---|---|
| 6 | 1.967444 | -5.556485 | 1.747273 |
| 6 | 2.916401 | -4.906740 | 0.959909 |
| 1 | 0.446182 | 0.646951 | -0.521151 |
| 1 | 0.883975 | -1.272389 | -1.914664 |
| 1 | 2.097134 | -1.160863 | -3.267688 |
| 1 | 4.943840 | 2.952132 | -1.381585 |
| 1 | 4.524425 | 2.632663 | 2.892980 |
| 1 | 5.060044 | 5.411652 | -1.187083 |
| 1 | 4.672740 | 5.086913 | 3.087315 |
| 1 | 4.962078 | 6.493053 | 1.054112 |
| 1 | 7.332992 | 2.077340 | 1.391575 |
| 1 | 6.102930 | -0.803929 | -1.554967 |
| 1 | 9.674783 | 1.824670 | 0.663790 |
| 1 | 8.446093 | -1.033412 | -2.309085 |
| 1 | 10.247772 | 0.271006 | -1.192809 |
| 1 | 6.751587 | 0.310679 | 2.796638 |
| 1 | 2.543575 | -0.187731 | 2.004378 |
| 1 | 6.416815 | -0.555640 | 5.076453 |
| 1 | 2.204919 | -1.060571 | 4.257703 |
| 1 | 4.156149 | -1.264027 | 5.832040 |
| 1 | 3.918931 | -5.607038 | -1.386418 |
| 1 | 4.451742 | -1.744527 | -3.207802 |
| 1 | 4.391725 | -6.691362 | -3.541384 |
| 1 | 4.963936 | -2.836979 | -5.367465 |
| 1 | 4.928518 | -5.320636 | -5.546713 |
| 1 | 4.648494 | -2.758462 | 2.304161 |
| 1 | 6.629834 | -3.546360 | -1.441810 |
| 1 | 6.746057 | -3.195555 | 3.501322 |
| 1 | 8.731942 | -3.971349 | -0.237257 |
| 1 | 8.811773 | -3.805217 | 2.245700 |
| 1 | 1.257133 | -1.972551 | 0.453297 |
| 1 | 3.865197 | -5.394457 | 0.760710 |
| 1 | -0.360496 | -3.080381 | 1.912398 |
| 1 | 2.168430 | -6.559088 | 2.114718 |
| 1 | 0.042049 | -5.406281 | 2.714197 |
| 1 | 2.347627 | 1.297842 | -2.860409 |
| 1 | -1.370412 | 1.143376 | -1.626996 |
| 6 | -0.951846 | 3.154253 | -1.219264 |
| 6 | 0.401420 | 3.836731 | -0.932202 |
| 1 | -1.416240 | 2.841672 | -0.279700 |
| 1 | -1.654959 | 3.802462 | -1.738966 |
| 7 | -0.712854 | 1.902571 | -1.970026 |
| 16 | -0.846365 | 1.864352 | -3.735623 |
| 8 | 0.444853 | 2.257787 | -4.299290 |
| 8 | -1.399624 | 0.538214 | -4.010158 |
| 6 | 1.315141 | 2.650022 | -0.518668 |
| 1 | 1.193458 | 2.495080 | 0.554098 |
| 1 | 2.364541 | 2.876338 | -0.680502 |
| 6 | 0.910220 | 4.618548 | -2.165253 |
| 6 | 2.123816 | 4.349993 | -2.807610 |
| 6 | 0.140134 | 5.689326 | -2.646772 |
| 6 | 2.552520 | 5.119498 | -3.892067 |
| 1 | 2.749914 | 3.530450 | -2.481284 |
| 6 | 0.562974 | 6.462142 | -3.725866 |
| 1 | -0.801903 | 5.932964 | -2.165690 |
| 6 | 1.776670 | 6.180240 | -4.357207 |
| 1 | 3.496014 | 4.880674 | -4.375252 |
| 1 | -0.058679 | 7.282863 | -4.072543 |
| 1 | 2.111866 | 6.780781 | -5.198111 |
| 6 | 0.268000 | 4.844061 | 0.220148 |
| 6 | -0.947159 | 5.463779 | 0.542035 |
| 6 | 1.404391 | 5.186142 | 0.965464 |

| | | | |
|---|---|---|---|
| 6 | -1.021835 | 6.392845 | 1.583724 |
| 1 | -1.855102 | 5.222131 | -0.000209 |
| 6 | 1.329556 | 6.100382 | 2.014030 |
| 1 | 2.357600 | 4.731493 | 0.730387 |
| 6 | 0.112852 | 6.710220 | 2.330323 |
| 1 | -1.974907 | 6.860563 | 1.814882 |
| 1 | 2.225836 | 6.333088 | 2.581485 |
| 1 | 0.050806 | 7.424328 | 3.146444 |
| 6 | -2.040340 | 3.151578 | -4.143882 |
| 6 | -3.358605 | 3.187914 | -3.582225 |
| 6 | -1.583125 | 4.122495 | -5.010120 |
| 6 | -3.876027 | 2.228907 | -2.671853 |
| 6 | -4.168388 | 4.330321 | -3.910631 |
| 6 | -2.427589 | 5.191374 | -5.382845 |
| 1 | -0.565689 | 4.078569 | -5.378259 |
| 6 | -5.091093 | 2.436279 | -2.054100 |
| 1 | -3.328380 | 1.326979 | -2.437634 |
| 6 | -5.435288 | 4.484297 | -3.285986 |
| 6 | -3.682438 | 5.298213 | -4.831061 |
| 1 | -2.060445 | 5.940183 | -6.077023 |
| 6 | -5.878887 | 3.569867 | -2.357881 |
| 1 | -5.439580 | 1.718923 | -1.320455 |
| 1 | -6.038019 | 5.351105 | -3.544140 |
| 1 | -4.321917 | 6.140725 | -5.081786 |
| 1 | -6.833304 | 3.709029 | -1.859530 |
| 6 | -2.807493 | -3.131348 | 0.438308 |
| 6 | -3.192209 | -3.932458 | -0.621533 |
| 6 | -3.974297 | -5.099710 | -0.363302 |
| 6 | -4.362936 | -5.995582 | -1.396534 |
| 1 | -4.044862 | -5.788994 | -2.412441 |
| 6 | -5.120271 | -7.111495 | -1.114122 |
| 1 | -5.403586 | -7.788501 | -1.915107 |
| 6 | -5.533552 | -7.385452 | 0.213092 |
| 1 | -6.131136 | -8.268612 | 0.420280 |
| 6 | -5.179534 | -6.533517 | 1.234801 |
| 1 | -5.497324 | -6.732378 | 2.255320 |
| 6 | -4.397008 | -5.374371 | 0.977790 |
| 6 | -4.076299 | -4.451257 | 2.002441 |
| 1 | -4.496635 | -4.608613 | 2.991447 |
| 6 | -3.300963 | -3.327963 | 1.765316 |
| 6 | -3.132806 | -2.321960 | 2.846593 |
| 6 | -2.969189 | -2.703596 | 4.168207 |
| 1 | -2.784916 | -3.748028 | 4.403405 |
| 6 | -3.079087 | -1.773323 | 5.232704 |
| 6 | -2.932588 | -2.179027 | 6.588442 |
| 1 | -2.681312 | -3.216016 | 6.796156 |
| 6 | -3.115904 | -1.283048 | 7.616284 |
| 1 | -3.002705 | -1.603845 | 8.647793 |
| 6 | -3.471323 | 0.058151 | 7.331465 |
| 1 | -3.639239 | 0.754715 | 8.147850 |
| 6 | -3.608303 | 0.485385 | 6.029050 |
| 1 | -3.888343 | 1.510083 | 5.815210 |
| 6 | -3.390965 | -0.403026 | 4.941685 |
| 6 | -3.459511 | 0.021096 | 3.576387 |
| 6 | -3.304925 | -0.927021 | 2.579716 |
| 6 | -2.869956 | -3.488801 | -2.013061 |
| 6 | -1.566823 | -3.713456 | -2.556582 |
| 6 | -0.563516 | -4.425187 | -1.845364 |
| 1 | -0.786474 | -4.802246 | -0.853380 |
| 6 | 0.682506 | -4.627911 | -2.394107 |
| 1 | 1.434497 | -5.170141 | -1.832163 |
| 6 | 0.996650 | -4.119784 | -3.677680 |

| | | | |
|---|---|---|---|
| 1 | 1.986479 | -4.279713 | -4.090560 |
| 6 | 0.048384 | -3.419174 | -4.388926 |
| 1 | 0.280811 | -3.017879 | -5.372528 |
| 6 | -1.250612 | -3.202499 | -3.856495 |
| 6 | -2.248826 | -2.491768 | -4.571374 |
| 1 | -2.004514 | -2.080823 | -5.546872 |
| 6 | -3.497775 | -2.309147 | -4.033366 |
| 1 | -4.244184 | -1.753302 | -4.592848 |
| 6 | -3.836030 | -2.802105 | -2.743065 |
| 6 | -3.745868 | 1.440494 | 3.189682 |
| 6 | -5.021135 | 1.732872 | 2.585008 |
| 6 | -6.026410 | 0.744449 | 2.388658 |
| 1 | -5.845523 | -0.269171 | 2.727025 |
| 6 | -7.221837 | 1.048074 | 1.774256 |
| 1 | -7.966513 | 0.270276 | 1.633022 |
| 6 | -7.486721 | 2.362935 | 1.322382 |
| 1 | -8.431192 | 2.588894 | 0.835113 |
| 6 | -6.545497 | 3.350033 | 1.511677 |
| 1 | -6.735935 | 4.367704 | 1.179278 |
| 6 | -5.305600 | 3.066266 | 2.145278 |
| 6 | -4.329174 | 4.074726 | 2.351510 |
| 1 | -4.543661 | 5.089836 | 2.025220 |
| 6 | -3.133854 | 3.774736 | 2.951732 |
| 1 | -2.398410 | 4.558974 | 3.090549 |
| 6 | -2.804991 | 2.452054 | 3.369019 |
| 8 | -2.396265 | 0.085715 | -0.967192 |
| 8 | -0.825284 | -0.055292 | 1.100606 |
| 8 | -1.849520 | -2.172721 | 0.198753 |
| 8 | -3.407449 | -0.506465 | 1.267401 |
| 15 | -2.034957 | -0.545743 | 0.349095 |
| 6 | -1.418038 | 2.215594 | 3.945852 |
| 6 | -1.190792 | 2.977143 | 5.272541 |
| 6 | -0.317297 | 2.606307 | 2.932804 |
| 1 | -1.292029 | 1.147876 | 4.145418 |
| 6 | 0.210946 | 2.703103 | 5.839088 |
| 1 | -1.304487 | 4.056289 | 5.096763 |
| 1 | -1.952167 | 2.694881 | 6.006813 |
| 6 | 1.081724 | 2.331536 | 3.496991 |
| 1 | -0.403828 | 3.669738 | 2.685337 |
| 1 | -0.471322 | 2.035976 | 2.013931 |
| 6 | 1.307238 | 3.062700 | 4.825675 |
| 1 | 0.352369 | 3.260645 | 6.774000 |
| 1 | 0.289552 | 1.636232 | 6.089967 |
| 1 | 1.845607 | 2.631445 | 2.769381 |
| 1 | 1.189528 | 1.253956 | 3.654569 |
| 1 | 2.296534 | 2.815922 | 5.232260 |
| 1 | 1.296104 | 4.148263 | 4.648321 |
| 6 | -5.230850 | -2.547906 | -2.196842 |
| 6 | -6.324982 | -3.229993 | -3.047797 |
| 6 | -5.538135 | -1.045295 | -2.019334 |
| 1 | -5.291350 | -2.991774 | -1.198509 |
| 6 | -7.715943 | -3.022274 | -2.430726 |
| 1 | -6.310828 | -2.817144 | -4.065974 |
| 1 | -6.106910 | -4.300613 | -3.136967 |
| 6 | -6.927760 | -0.845216 | -1.397679 |
| 1 | -5.495439 | -0.538415 | -2.992663 |
| 1 | -4.763323 | -0.588868 | -1.397069 |
| 6 | -8.023516 | -1.531528 | -2.225964 |
| 1 | -8.482983 | -3.488745 | -3.061592 |
| 1 | -7.753396 | -3.535744 | -1.459170 |
| 1 | -7.146866 | 0.223608 | -1.291400 |
| 1 | -6.923571 | -1.259485 | -0.379800 |

| | | | |
|---|---|---|---|
| 1 | -9.002265 | -1.407370 | -1.745100 |
| 1 | -8.090509 | -1.040396 | -3.207796 |
| 1 | -0.255362 | -0.622666 | 2.689055 |
| 8 | 0.020925 | -0.957133 | 3.569170 |
| 1 | -0.759848 | -1.411751 | 3.906086 |

---

(*S*,*S*,*S*)-**[2a-3a]**$^{‡}$endo-*si*

---

Number of imaginary frequencies : 1
The smallest frequencies are : -350.7991 13.1557 14.1348 cm(-1)

Electronic energy : HF=-6614.0032626
Zero-point correction= 1.888472 (Hartree/Particle)
Thermal correction to Energy= 1.999392
Thermal correction to Enthalpy= 2.000336
Thermal correction to Gibbs Free Energy= 1.736352
Sum of electronic and zero-point Energies= -6612.114791
Sum of electronic and thermal Energies= -6612.003870
Sum of electronic and thermal Enthalpies= -6612.002926
Sum of electronic and thermal Free Energies= -6612.266911

............................................................

Cartesian Coordinates

............................................................

| | | | |
|---|---|---|---|
| 6 | -3.318040 | -0.016264 | 2.811784 |
| 6 | -4.571834 | -0.476985 | 2.375876 |
| 6 | -5.661348 | -0.410466 | 3.255628 |
| 6 | -5.494542 | 0.104249 | 4.542856 |
| 6 | -4.242565 | 0.559101 | 4.965602 |
| 6 | -3.149077 | 0.497646 | 4.098232 |
| 15 | -4.655162 | -1.123708 | 0.650881 |
| 6 | -6.443503 | -1.133265 | 0.170996 |
| 6 | -7.382876 | -1.972324 | 0.796955 |
| 6 | -8.717097 | -1.968943 | 0.393225 |
| 6 | -9.131042 | -1.143590 | -0.656725 |
| 6 | -8.202633 | -0.327515 | -1.302043 |
| 6 | -6.868082 | -0.323485 | -0.890410 |
| 46 | -3.263217 | 0.227484 | -0.717267 |
| 6 | -1.530266 | 0.817484 | -1.925740 |
| 6 | -1.128682 | -0.280606 | -1.139192 |
| 6 | -1.006758 | -1.636316 | -1.614823 |
| 15 | -4.209698 | 2.411603 | -0.532202 |
| 6 | -5.787584 | 2.717137 | 0.382154 |
| 6 | -5.781886 | 2.634516 | 1.785269 |
| 6 | -6.966101 | 2.775830 | 2.507558 |
| 6 | -8.175348 | 2.996677 | 1.844502 |
| 6 | -8.191766 | 3.076195 | 0.451419 |
| 6 | -7.008150 | 2.936832 | -0.275149 |
| 6 | -4.440024 | -2.948663 | 0.895372 |
| 6 | -4.543513 | -3.772926 | -0.238912 |
| 6 | -4.495992 | -5.161186 | -0.119522 |
| 6 | -4.340912 | -5.749546 | 1.140178 |
| 6 | -4.212203 | -4.939405 | 2.269281 |
| 6 | -4.255454 | -3.546347 | 2.149751 |
| 6 | -4.561946 | 3.187216 | -2.174057 |
| 6 | -4.912653 | 4.534397 | -2.359163 |
| 6 | -5.247610 | 5.013825 | -3.625602 |
| 6 | -5.252100 | 4.151928 | -4.727033 |
| 6 | -4.908687 | 2.810452 | -4.555753 |
| 6 | -4.561966 | 2.334949 | -3.288922 |
| 6 | -3.048455 | 3.496457 | 0.418103 |
| 6 | -1.858608 | 2.939438 | 0.908365 |

| | | | |
|---|---|---|---|
| 6 | -0.975025 | 3.693854 | 1.682887 |
| 6 | -1.258870 | 5.031295 | 1.953465 |
| 6 | -2.448071 | 5.598925 | 1.481665 |
| 6 | -3.346704 | 4.832388 | 0.740724 |
| 1 | -1.456293 | -1.852676 | -2.579793 |
| 1 | -1.768268 | 0.669311 | -2.978301 |
| 1 | -1.187633 | 1.816586 | -1.678085 |
| 1 | -4.682102 | -3.320822 | -1.218460 |
| 1 | -4.165956 | -2.930575 | 3.038049 |
| 1 | -4.572414 | -5.782482 | -1.006884 |
| 1 | -4.078973 | -5.387127 | 3.250077 |
| 1 | -4.303048 | -6.830649 | 1.235215 |
| 1 | -7.067639 | -2.643329 | 1.590342 |
| 1 | -6.148450 | 0.310076 | -1.397033 |
| 1 | -9.431952 | -2.618423 | 0.890902 |
| 1 | -8.512522 | 0.309786 | -2.125352 |
| 1 | -10.170184 | -1.145908 | -0.973824 |
| 1 | -6.645863 | -0.731919 | 2.935880 |
| 1 | -2.465988 | -0.049686 | 2.140359 |
| 1 | -6.348652 | 0.157452 | 5.212766 |
| 1 | -2.163001 | 0.844977 | 4.390361 |
| 1 | -4.121273 | 0.964018 | 5.966675 |
| 1 | -4.935427 | 5.215871 | -1.517269 |
| 1 | -4.278789 | 1.293584 | -3.160506 |
| 1 | -5.510295 | 6.060335 | -3.751630 |
| 1 | -4.900508 | 2.133864 | -5.405512 |
| 1 | -5.517756 | 4.527080 | -5.711229 |
| 1 | -4.853537 | 2.453354 | 2.317583 |
| 1 | -7.040767 | 3.003285 | -1.357339 |
| 1 | -6.939124 | 2.704542 | 3.590720 |
| 1 | -9.126803 | 3.245586 | -0.075611 |
| 1 | -9.097190 | 3.105200 | 2.409170 |
| 1 | -1.619736 | 1.903416 | 0.702602 |
| 1 | -4.292732 | 5.271369 | 0.442407 |
| 1 | -0.090296 | 3.212537 | 2.081169 |
| 1 | -2.686215 | 6.634349 | 1.709594 |
| 1 | -0.566829 | 5.627235 | 2.542192 |
| 1 | -0.639119 | -0.091291 | -0.188209 |
| 1 | 1.315023 | -1.050426 | -1.719799 |
| 6 | 1.205834 | -3.098587 | -1.343725 |
| 6 | -0.070201 | -3.894710 | -0.995535 |
| 1 | 1.674884 | -2.721606 | -0.431557 |
| 1 | 1.944785 | -3.685714 | -1.882356 |
| 7 | 0.805451 | -1.893459 | -2.114560 |
| 16 | 1.060606 | -1.826091 | -3.874091 |
| 8 | -0.181843 | -2.288857 | -4.499071 |
| 8 | 1.539016 | -0.464713 | -4.111734 |
| 6 | -1.038833 | -2.759410 | -0.601302 |
| 1 | -0.737864 | -2.367120 | 0.373003 |
| 1 | -2.066692 | -3.104137 | -0.496654 |
| 6 | -0.549028 | -4.732961 | -2.200624 |
| 6 | -1.839609 | -4.633404 | -2.733861 |
| 6 | 0.321442 | -5.678791 | -2.764437 |
| 6 | -2.241737 | -5.435917 | -3.804308 |
| 1 | -2.551549 | -3.929269 | -2.322491 |
| 6 | -0.075155 | -6.483823 | -3.830407 |
| 1 | 1.320955 | -5.800979 | -2.358887 |
| 6 | -1.361745 | -6.363186 | -4.360638 |
| 1 | -3.247307 | -5.330028 | -4.202137 |
| 1 | 0.623651 | -7.204410 | -4.245907 |
| 1 | -1.674066 | -6.987460 | -5.192911 |
| 6 | 0.182288 | -4.852425 | 0.174897 |

| | | | |
|---|---|---|---|
| 6 | 1.450981 | -5.387335 | 0.438418 |
| 6 | -0.886412 | -5.245601 | 0.991095 |
| 6 | 1.641529 | -6.292040 | 1.486105 |
| 1 | 2.309419 | -5.097253 | -0.158495 |
| 6 | -0.696250 | -6.137295 | 2.044954 |
| 1 | -1.878077 | -4.853891 | 0.809279 |
| 6 | 0.570907 | -6.667378 | 2.298434 |
| 1 | 2.633267 | -6.695692 | 1.670359 |
| 1 | -1.542897 | -6.412669 | 2.666876 |
| 1 | 0.721881 | -7.363452 | 3.118508 |
| 6 | 2.351644 | -3.025806 | -4.230158 |
| 6 | 3.646645 | -2.957357 | -3.619305 |
| 6 | 2.005720 | -4.023161 | -5.118590 |
| 6 | 4.042528 | -1.975231 | -2.672815 |
| 6 | 4.564647 | -4.018042 | -3.935655 |
| 6 | 2.949922 | -5.013057 | -5.468101 |
| 1 | 1.003590 | -4.056299 | -5.527645 |
| 6 | 5.253639 | -2.080455 | -2.022813 |
| 1 | 3.401375 | -1.138382 | -2.432852 |
| 6 | 5.822617 | -4.065254 | -3.276081 |
| 6 | 4.193104 | -5.013583 | -4.879508 |
| 1 | 2.672531 | -5.783533 | -6.180002 |
| 6 | 6.153169 | -3.128811 | -2.323121 |
| 1 | 5.513334 | -1.349668 | -1.265840 |
| 1 | 6.509620 | -4.869092 | -3.527174 |
| 1 | 4.911852 | -5.792834 | -5.119204 |
| 1 | 7.101121 | -3.185570 | -1.797487 |
| 6 | 2.404238 | 3.286843 | 0.457673 |
| 6 | 2.599235 | 4.141117 | -0.612755 |
| 6 | 3.237383 | 5.400600 | -0.397648 |
| 6 | 3.410464 | 6.350137 | -1.441987 |
| 1 | 3.033842 | 6.112093 | -2.430834 |
| 6 | 4.034410 | 7.555067 | -1.202546 |
| 1 | 4.152049 | 8.272139 | -2.010110 |
| 6 | 4.523263 | 7.869469 | 0.089655 |
| 1 | 5.017045 | 8.821501 | 0.262435 |
| 6 | 4.376436 | 6.968569 | 1.120051 |
| 1 | 4.753520 | 7.199070 | 2.113507 |
| 6 | 3.730913 | 5.719251 | 0.908296 |
| 6 | 3.622331 | 4.751388 | 1.935550 |
| 1 | 4.102376 | 4.951897 | 2.888879 |
| 6 | 2.980626 | 3.538877 | 1.742161 |
| 6 | 3.038814 | 2.512206 | 2.813596 |
| 6 | 2.922467 | 2.856753 | 4.149574 |
| 1 | 2.628015 | 3.867691 | 4.416280 |
| 6 | 3.207710 | 1.932812 | 5.186070 |
| 6 | 3.118429 | 2.306346 | 6.556044 |
| 1 | 2.776884 | 3.309084 | 6.800026 |
| 6 | 3.473126 | 1.425631 | 7.551633 |
| 1 | 3.405800 | 1.722884 | 8.594132 |
| 6 | 3.943352 | 0.132016 | 7.217579 |
| 1 | 4.244812 | -0.550422 | 8.007121 |
| 6 | 4.023793 | -0.266603 | 5.901344 |
| 1 | 4.389042 | -1.255744 | 5.650986 |
| 6 | 3.637593 | 0.605963 | 4.847758 |
| 6 | 3.663518 | 0.211040 | 3.471989 |
| 6 | 3.365379 | 1.154921 | 2.504713 |
| 6 | 2.274172 | 3.666359 | -1.992831 |
| 6 | 0.965691 | 3.844908 | -2.537120 |
| 6 | -0.063770 | 4.520996 | -1.826842 |
| 1 | 0.137917 | 4.887664 | -0.826386 |
| 6 | -1.304758 | 4.710330 | -2.393652 |

| | | | |
|---|---|---|---|
| 1 | -2.077822 | 5.228058 | -1.836630 |
| 6 | -1.587397 | 4.216904 | -3.689846 |
| 1 | -2.571160 | 4.368373 | -4.118155 |
| 6 | -0.617735 | 3.541008 | -4.395404 |
| 1 | -0.828621 | 3.152558 | -5.389143 |
| 6 | 0.678241 | 3.344753 | -3.848589 |
| 6 | 1.705766 | 2.680535 | -4.565943 |
| 1 | 1.483966 | 2.276052 | -5.549566 |
| 6 | 2.959329 | 2.542345 | -4.025671 |
| 1 | 3.730483 | 2.029033 | -4.591758 |
| 6 | 3.273440 | 3.036819 | -2.730545 |
| 6 | 4.041789 | -1.177364 | 3.054360 |
| 6 | 5.319073 | -1.374475 | 2.420801 |
| 6 | 6.236948 | -0.310934 | 2.191462 |
| 1 | 5.984005 | 0.687528 | 2.529042 |
| 6 | 7.433722 | -0.524163 | 1.542540 |
| 1 | 8.110074 | 0.308570 | 1.373490 |
| 6 | 7.789189 | -1.818519 | 1.092671 |
| 1 | 8.734745 | -1.973294 | 0.580424 |
| 6 | 6.935605 | -2.875728 | 1.315821 |
| 1 | 7.198092 | -3.877875 | 0.985351 |
| 6 | 5.694360 | -2.685732 | 1.981095 |
| 6 | 4.805058 | -3.764689 | 2.220127 |
| 1 | 5.092147 | -4.763860 | 1.900495 |
| 6 | 3.600923 | -3.551328 | 2.840324 |
| 1 | 2.932126 | -4.388620 | 3.003189 |
| 6 | 3.178475 | -2.253676 | 3.250009 |
| 8 | 2.288590 | 0.026945 | -0.959021 |
| 8 | 0.957217 | -0.026831 | 1.280190 |
| 8 | 1.571130 | 2.206751 | 0.272490 |
| 8 | 3.439904 | 0.775795 | 1.175959 |
| 15 | 1.989749 | 0.614650 | 0.393502 |
| 6 | 1.782893 | -2.108029 | 3.835208 |
| 6 | 1.632460 | -2.776961 | 5.221127 |
| 6 | 0.708599 | -2.681061 | 2.883337 |
| 1 | 1.564557 | -1.042843 | 3.952244 |
| 6 | 0.220779 | -2.556046 | 5.788388 |
| 1 | 1.822316 | -3.855541 | 5.122487 |
| 1 | 2.378814 | -2.385445 | 5.919011 |
| 6 | -0.700434 | -2.439442 | 3.436533 |
| 1 | 0.862680 | -3.757839 | 2.754106 |
| 1 | 0.817468 | -2.205874 | 1.908209 |
| 6 | -0.865228 | -3.064151 | 4.827863 |
| 1 | 0.129580 | -3.046363 | 6.766241 |
| 1 | 0.073788 | -1.480486 | 5.957531 |
| 1 | -1.448908 | -2.848192 | 2.747017 |
| 1 | -0.873722 | -1.360183 | 3.503267 |
| 1 | -1.861362 | -2.841999 | 5.231611 |
| 1 | -0.790851 | -4.158522 | 4.742861 |
| 6 | 4.694805 | 2.900164 | -2.209759 |
| 6 | 5.673562 | 3.792559 | -3.007080 |
| 6 | 5.199997 | 1.443140 | -2.166311 |
| 1 | 4.714707 | 3.258371 | -1.175929 |
| 6 | 7.092183 | 3.720009 | -2.424310 |
| 1 | 5.688689 | 3.466960 | -4.056840 |
| 1 | 5.314069 | 4.827949 | -3.001628 |
| 6 | 6.618600 | 1.376200 | -1.581557 |
| 1 | 5.202804 | 1.014033 | -3.177165 |
| 1 | 4.505773 | 0.844275 | -1.572098 |
| 6 | 7.596640 | 2.271340 | -2.355814 |
| 1 | 7.777619 | 4.337276 | -3.018690 |
| 1 | 7.081406 | 4.148452 | -1.411705 |

| | | | |
|---|---|---|---|
| 1 | 6.977799 | 0.340718 | -1.578065 |
| 1 | 6.584553 | 1.691869 | -0.529568 |
| 1 | 8.593505 | 2.235722 | -1.897954 |
| 1 | 7.705850 | 1.881593 | -3.378452 |
| 1 | 0.320431 | 0.661613 | 2.757399 |
| 8 | 0.003366 | 1.054642 | 3.600897 |
| 1 | 0.806587 | 1.383128 | 4.021755 |

---

(*S*,*R*,*S*)-**3a**endo-*re*

---

Number of imaginary frequencies : 0
The smallest frequencies are : 12.2949 18.5649 19.5637 cm(-1)

Electronic energy : HF=-6614.0135074
Zero-point correction= 1.889295 (Hartree/Particle)
Thermal correction to Energy= 2.000050
Thermal correction to Enthalpy= 2.000994
Thermal correction to Gibbs Free Energy= 1.738816
Sum of electronic and zero-point Energies= -6612.124212
Sum of electronic and thermal Energies= -6612.013458
Sum of electronic and thermal Enthalpies= -6612.012513
Sum of electronic and thermal Free Energies= -6612.274692

Cartesian Coordinates

| | | | |
|---|---|---|---|
| 6 | -3.600509 | 0.203016 | 2.770591 |
| 6 | -4.860086 | 0.403553 | 2.178211 |
| 6 | -6.013080 | 0.097282 | 2.916810 |
| 6 | -5.907383 | -0.407092 | 4.215339 |
| 6 | -4.652140 | -0.604382 | 4.794844 |
| 6 | -3.500186 | -0.289857 | 4.071651 |
| 15 | -4.859976 | 0.977259 | 0.419672 |
| 6 | -6.614427 | 0.847074 | -0.147555 |
| 6 | -7.649633 | 1.600645 | 0.433604 |
| 6 | -8.964095 | 1.459429 | -0.009865 |
| 6 | -9.261080 | 0.576105 | -1.052277 |
| 6 | -8.237510 | -0.155649 | -1.654547 |
| 6 | -6.922157 | -0.018446 | -1.205758 |
| 46 | -3.273148 | -0.292751 | -0.800048 |
| 6 | -1.345477 | -0.550456 | -1.833593 |
| 6 | -1.255986 | 0.585475 | -1.016578 |
| 6 | -1.085599 | 1.979719 | -1.522617 |
| 15 | -3.931249 | -2.566169 | -0.538991 |
| 6 | -5.710775 | -3.035959 | -0.350973 |
| 6 | -6.396578 | -2.651731 | 0.814744 |
| 6 | -7.768351 | -2.862036 | 0.937359 |
| 6 | -8.486889 | -3.449016 | -0.107196 |
| 6 | -7.817138 | -3.831552 | -1.270405 |
| 6 | -6.439797 | -3.630181 | -1.392246 |
| 6 | -4.709018 | 2.817656 | 0.585089 |
| 6 | -5.131314 | 3.625896 | -0.486108 |
| 6 | -4.921502 | 5.004950 | -0.464446 |
| 6 | -4.274284 | 5.599312 | 0.621932 |
| 6 | -3.847809 | 4.805457 | 1.687369 |
| 6 | -4.067740 | 3.426684 | 1.673469 |
| 6 | -3.378542 | -3.778130 | -1.820231 |
| 6 | -3.091133 | -5.121123 | -1.533460 |
| 6 | -2.759811 | -6.007490 | -2.559789 |
| 6 | -2.729752 | -5.567314 | -3.886363 |
| 6 | -3.015441 | -4.232182 | -4.180811 |
| 6 | -3.322037 | -3.339224 | -3.151167 |

| | | | |
|---|---|---|---|
| 6 | -3.167646 | -3.170411 | 1.034764 |
| 6 | -1.975663 | -2.565329 | 1.466271 |
| 6 | -1.379888 | -2.935396 | 2.672000 |
| 6 | -1.971671 | -3.916718 | 3.468463 |
| 6 | -3.146485 | -4.542152 | 3.042800 |
| 6 | -3.739575 | -4.175751 | 1.833140 |
| 1 | -1.512911 | 2.128789 | -2.514233 |
| 1 | -1.461116 | -0.438233 | -2.910712 |
| 1 | -0.890948 | -1.487032 | -1.526989 |
| 1 | -5.628636 | 3.175559 | -1.340829 |
| 1 | -3.714542 | 2.825745 | 2.504340 |
| 1 | -5.252829 | 5.613199 | -1.301776 |
| 1 | -3.322586 | 5.257141 | 2.523284 |
| 1 | -4.089496 | 6.668938 | 0.632248 |
| 1 | -7.424791 | 2.303413 | 1.230411 |
| 1 | -6.128023 | -0.591950 | -1.670980 |
| 1 | -9.755499 | 2.042042 | 0.453792 |
| 1 | -8.458596 | -0.842833 | -2.465511 |
| 1 | -10.285963 | 0.466120 | -1.396147 |
| 1 | -6.995856 | 0.223331 | 2.477056 |
| 1 | -2.688923 | 0.433450 | 2.226313 |
| 1 | -6.809636 | -0.651587 | 4.769902 |
| 1 | -2.517643 | -0.446383 | 4.503974 |
| 1 | -4.572630 | -1.005851 | 5.801681 |
| 1 | -3.119900 | -5.473940 | -0.507351 |
| 1 | -3.514964 | -2.292937 | -3.374125 |
| 1 | -2.527890 | -7.042358 | -2.323906 |
| 1 | -2.987360 | -3.882541 | -5.208982 |
| 1 | -2.474512 | -6.258816 | -4.684246 |
| 1 | -5.855465 | -2.185366 | 1.629906 |
| 1 | -5.936927 | -3.939731 | -2.302843 |
| 1 | -8.276807 | -2.556322 | 1.847608 |
| 1 | -8.364105 | -4.294745 | -2.087430 |
| 1 | -9.558005 | -3.605261 | -0.015702 |
| 1 | -1.500411 | -1.791441 | 0.875957 |
| 1 | -4.660664 | -4.659495 | 1.524933 |
| 1 | -0.460525 | -2.443999 | 2.969755 |
| 1 | -3.607699 | -5.313767 | 3.653595 |
| 1 | -1.518043 | -4.196627 | 4.415279 |
| 1 | -0.842526 | 0.450110 | -0.022066 |
| 1 | 0.994730 | 1.412778 | -1.270105 |
| 6 | 0.812228 | 3.469334 | -0.856927 |
| 6 | -0.509382 | 4.257413 | -0.685392 |
| 1 | 1.158720 | 3.071401 | 0.095938 |
| 1 | 1.629122 | 4.029236 | -1.308508 |
| 7 | 0.478640 | 2.261130 | -1.686354 |
| 16 | 0.980693 | 2.249641 | -3.468003 |
| 8 | -0.059900 | 2.985722 | -4.183726 |
| 8 | 1.203764 | 0.833692 | -3.720411 |
| 6 | -1.498138 | 3.075585 | -0.538475 |
| 1 | -1.425432 | 2.670865 | 0.471614 |
| 1 | -2.531359 | 3.371657 | -0.688149 |
| 6 | -0.764648 | 5.123767 | -1.929353 |
| 6 | -1.952393 | 5.060456 | -2.666007 |
| 6 | 0.204778 | 6.059510 | -2.327142 |
| 6 | -2.160153 | 5.888674 | -3.771129 |
| 1 | -2.731580 | 4.361305 | -2.389181 |
| 6 | -0.001719 | 6.896821 | -3.422704 |
| 1 | 1.125856 | 6.153654 | -1.758672 |
| 6 | -1.186975 | 6.809511 | -4.157039 |
| 1 | -3.088206 | 5.808643 | -4.330255 |
| 1 | 0.763688 | 7.615985 | -3.701647 |

| | | | |
|---|---|---|---|
| 1 | -1.350254 | 7.454631 | -5.015649 |
| 6 | -0.504014 | 5.109629 | 0.603270 |
| 6 | -0.194510 | 4.510218 | 1.837425 |
| 6 | -0.890132 | 6.456718 | 0.608260 |
| 6 | -0.267873 | 5.238031 | 3.025043 |
| 1 | 0.060462 | 3.456453 | 1.891017 |
| 6 | -0.954317 | 7.187903 | 1.797577 |
| 1 | -1.162000 | 6.946735 | -0.318258 |
| 6 | -0.643586 | 6.583242 | 3.014363 |
| 1 | -0.044120 | 4.742101 | 3.964225 |
| 1 | -1.259057 | 8.230525 | 1.766191 |
| 1 | -0.699831 | 7.146785 | 3.941566 |
| 6 | 2.501911 | 3.201613 | -3.521654 |
| 6 | 3.741828 | 2.725742 | -2.976558 |
| 6 | 2.392268 | 4.426123 | -4.154403 |
| 6 | 3.924467 | 1.476536 | -2.332115 |
| 6 | 4.873039 | 3.609172 | -3.092788 |
| 6 | 3.521395 | 5.263689 | -4.267841 |
| 1 | 1.437866 | 4.745480 | -4.551637 |
| 6 | 5.154405 | 1.126263 | -1.815533 |
| 1 | 3.103906 | 0.784846 | -2.229456 |
| 6 | 6.126859 | 3.205792 | -2.560884 |
| 6 | 4.727791 | 4.864361 | -3.741168 |
| 1 | 3.423114 | 6.221556 | -4.767924 |
| 6 | 6.266491 | 1.989583 | -1.931264 |
| 1 | 5.259809 | 0.170732 | -1.311582 |
| 1 | 6.972589 | 3.880816 | -2.659406 |
| 1 | 5.598449 | 5.510449 | -3.817116 |
| 1 | 7.227134 | 1.690981 | -1.522418 |
| 6 | 3.783936 | -2.336026 | -0.485971 |
| 6 | 3.856672 | -2.825388 | -1.784386 |
| 6 | 5.128492 | -3.268061 | -2.281174 |
| 6 | 5.275507 | -3.904077 | -3.546202 |
| 1 | 4.393282 | -4.084697 | -4.148646 |
| 6 | 6.514302 | -4.292493 | -4.005437 |
| 1 | 6.600675 | -4.781105 | -4.971798 |
| 6 | 7.677703 | -4.060612 | -3.231302 |
| 1 | 8.649273 | -4.364275 | -3.610466 |
| 6 | 7.570097 | -3.466559 | -1.995299 |
| 1 | 8.452716 | -3.300841 | -1.382408 |
| 6 | 6.301909 | -3.074124 | -1.483274 |
| 6 | 6.169009 | -2.532728 | -0.183420 |
| 1 | 7.063111 | -2.397104 | 0.418240 |
| 6 | 4.934631 | -2.216223 | 0.357886 |
| 6 | 4.835017 | -1.859604 | 1.796201 |
| 6 | 5.610674 | -2.528838 | 2.726772 |
| 1 | 6.251857 | -3.342384 | 2.400903 |
| 6 | 5.588053 | -2.195543 | 4.102329 |
| 6 | 6.423134 | -2.863111 | 5.040241 |
| 1 | 7.085510 | -3.646682 | 4.681013 |
| 6 | 6.400541 | -2.521807 | 6.373074 |
| 1 | 7.045299 | -3.035088 | 7.080682 |
| 6 | 5.537196 | -1.493680 | 6.824212 |
| 1 | 5.530091 | -1.221509 | 7.875868 |
| 6 | 4.706317 | -0.837187 | 5.943172 |
| 1 | 4.049664 | -0.051638 | 6.299263 |
| 6 | 4.699922 | -1.168798 | 4.559979 |
| 6 | 3.842405 | -0.516755 | 3.614194 |
| 6 | 3.959129 | -0.838805 | 2.274134 |
| 6 | 2.632420 | -2.898426 | -2.645184 |
| 6 | 2.578314 | -2.083922 | -3.834139 |
| 6 | 3.630377 | -1.208274 | -4.224284 |

| | | | |
|---|---|---|---|
| 1 | 4.494541 | -1.096839 | -3.583095 |
| 6 | 3.572474 | -0.493354 | -5.399811 |
| 1 | 4.393404 | 0.167658 | -5.664240 |
| 6 | 2.451307 | -0.599859 | -6.254156 |
| 1 | 2.416522 | -0.031315 | -7.179394 |
| 6 | 1.399592 | -1.410443 | -5.895062 |
| 1 | 0.519449 | -1.488228 | -6.528946 |
| 6 | 1.432381 | -2.162182 | -4.690527 |
| 6 | 0.353092 | -2.998662 | -4.310683 |
| 1 | -0.531783 | -3.040592 | -4.939512 |
| 6 | 0.423635 | -3.756627 | -3.170515 |
| 1 | -0.413365 | -4.393592 | -2.907566 |
| 6 | 1.571922 | -3.744973 | -2.329885 |
| 6 | 2.804044 | 0.451210 | 4.088202 |
| 6 | 1.686848 | -0.084204 | 4.821622 |
| 6 | 1.478413 | -1.483265 | 4.982617 |
| 1 | 2.161212 | -2.176992 | 4.507606 |
| 6 | 0.415368 | -1.965804 | 5.713210 |
| 1 | 0.280232 | -3.037995 | 5.820400 |
| 6 | -0.506037 | -1.078755 | 6.319952 |
| 1 | -1.340066 | -1.473372 | 6.893152 |
| 6 | -0.351429 | 0.280237 | 6.163948 |
| 1 | -1.064148 | 0.972334 | 6.605224 |
| 6 | 0.734312 | 0.808189 | 5.413172 |
| 6 | 0.896710 | 2.205805 | 5.214008 |
| 1 | 0.178640 | 2.889055 | 5.660824 |
| 6 | 1.935852 | 2.683410 | 4.453949 |
| 1 | 2.026959 | 3.752174 | 4.292428 |
| 6 | 2.911309 | 1.821492 | 3.877247 |
| 8 | 1.803186 | 0.305539 | -0.602155 |
| 8 | 0.816527 | -0.964438 | 1.470180 |
| 8 | 2.535965 | -2.112785 | 0.056355 |
| 8 | 3.280511 | -0.074806 | 1.350284 |
| 15 | 1.958343 | -0.653293 | 0.547427 |
| 6 | 4.012710 | 2.443184 | 3.037265 |
| 6 | 3.426150 | 3.107321 | 1.769685 |
| 6 | 4.879865 | 3.446634 | 3.828359 |
| 1 | 4.680107 | 1.645533 | 2.697738 |
| 6 | 4.523540 | 3.725120 | 0.895057 |
| 1 | 2.709093 | 3.886439 | 2.061461 |
| 1 | 2.876895 | 2.350839 | 1.204197 |
| 6 | 5.979242 | 4.051336 | 2.941197 |
| 1 | 4.249382 | 4.257263 | 4.218028 |
| 1 | 5.320976 | 2.945251 | 4.698498 |
| 6 | 5.388268 | 4.714179 | 1.687722 |
| 1 | 4.075442 | 4.220480 | 0.024620 |
| 1 | 5.156377 | 2.922953 | 0.496399 |
| 1 | 6.568518 | 4.776476 | 3.515956 |
| 1 | 6.672920 | 3.255108 | 2.634800 |
| 1 | 6.190024 | 5.112556 | 1.053445 |
| 1 | 4.770999 | 5.571869 | 1.992329 |
| 6 | 1.606240 | -4.721104 | -1.166809 |
| 6 | 0.442638 | -4.515724 | -0.174147 |
| 6 | 1.631259 | -6.182576 | -1.674917 |
| 1 | 2.534894 | -4.570013 | -0.609120 |
| 6 | 0.519826 | -5.514710 | 0.988678 |
| 1 | -0.513643 | -4.648857 | -0.693604 |
| 1 | 0.462026 | -3.492497 | 0.206431 |
| 6 | 1.710033 | -7.178231 | -0.508322 |
| 1 | 0.722964 | -6.380180 | -2.260362 |
| 1 | 2.480697 | -6.319928 | -2.355680 |
| 6 | 0.560375 | -6.964774 | 0.486577 |

| | | | |
|---|---|---|---|
| 1 | -0.331743 | -5.366329 | 1.660483 |
| 1 | 1.424996 | -5.308274 | 1.578471 |
| 1 | 1.703860 | -8.207338 | -0.889470 |
| 1 | 2.667880 | -7.042427 | 0.014342 |
| 1 | 0.651388 | -7.661768 | 1.329450 |
| 1 | -0.392184 | -7.197626 | -0.011370 |
| 1 | -0.025346 | 0.435607 | 2.022847 |
| 8 | -0.488588 | 1.272627 | 2.258215 |
| 1 | -0.244749 | 1.433546 | 3.178266 |

---

(*S*,*R*,*S*)-**3a**exo-*si*

---

Number of imaginary frequencies : 0
The smallest frequencies are : 16.0168 16.3943 19.0440 cm(-1)

Electronic energy : HF=-6614.0223715
Zero-point correction= 1.889967 (Hartree/Particle)
Thermal correction to Energy= 2.000353
Thermal correction to Enthalpy= 2.001297
Thermal correction to Gibbs Free Energy= 1.740013
Sum of electronic and zero-point Energies= -6612.132405
Sum of electronic and thermal Energies= -6612.022018
Sum of electronic and thermal Enthalpies= -6612.021074
Sum of electronic and thermal Free Energies= -6612.282359

Cartesian Coordinates

| | | | |
|---|---|---|---|
| 6 | -3.585013 | 0.884513 | 2.354974 |
| 6 | -4.899314 | 0.968520 | 1.862381 |
| 6 | -5.964353 | 0.965977 | 2.775486 |
| 6 | -5.716005 | 0.879827 | 4.148299 |
| 6 | -4.405139 | 0.804920 | 4.626927 |
| 6 | -3.338100 | 0.811141 | 3.725781 |
| 15 | -5.066461 | 1.029030 | 0.021434 |
| 6 | -6.842447 | 0.702364 | -0.367261 |
| 6 | -7.879355 | 1.552828 | 0.053870 |
| 6 | -9.204196 | 1.270692 | -0.277939 |
| 6 | -9.509448 | 0.147313 | -1.052384 |
| 6 | -8.483890 | -0.684833 | -1.502096 |
| 6 | -7.158262 | -0.405991 | -1.163974 |
| 46 | -3.425533 | -0.400050 | -0.917901 |
| 6 | -1.564139 | -0.664562 | -2.018755 |
| 6 | -1.886486 | 0.704954 | -2.095437 |
| 6 | -1.161371 | 1.714397 | -1.255359 |
| 15 | -3.881416 | -2.570749 | -0.070293 |
| 6 | -5.643138 | -2.980989 | 0.329310 |
| 6 | -6.241015 | -2.337819 | 1.427381 |
| 6 | -7.588249 | -2.533596 | 1.720731 |
| 6 | -8.368637 | -3.369610 | 0.917441 |
| 6 | -7.785193 | -4.013775 | -0.174052 |
| 6 | -6.431039 | -3.824506 | -0.466122 |
| 6 | -5.029197 | 2.848424 | -0.324100 |
| 6 | -5.142769 | 3.250962 | -1.666562 |
| 6 | -5.100371 | 4.600316 | -2.008569 |
| 6 | -4.925302 | 5.571403 | -1.014890 |
| 6 | -4.799875 | 5.180956 | 0.317920 |
| 6 | -4.861910 | 3.827232 | 0.663964 |
| 6 | -3.430367 | -3.978789 | -1.182018 |
| 6 | -3.085265 | -5.253349 | -0.707548 |
| 6 | -2.825532 | -6.294748 | -1.600717 |
| 6 | -2.918741 | -6.080361 | -2.978547 |

| | | | |
|---|---|---|---|
| 6 | -3.263499 | -4.815418 | -3.460317 |
| 6 | -3.506138 | -3.769224 | -2.567506 |
| 6 | -3.044798 | -2.948509 | 1.531440 |
| 6 | -1.859890 | -2.268327 | 1.846649 |
| 6 | -1.213712 | -2.496647 | 3.063308 |
| 6 | -1.749073 | -3.401791 | 3.979730 |
| 6 | -2.921668 | -4.097037 | 3.671824 |
| 6 | -3.565662 | -3.872970 | 2.455033 |
| 1 | -1.061413 | 1.367389 | -0.223953 |
| 1 | -0.763778 | -1.005247 | -1.366048 |
| 1 | -1.730383 | -1.300613 | -2.883098 |
| 1 | -5.250447 | 2.499301 | -2.444637 |
| 1 | -4.765471 | 3.537878 | 1.704846 |
| 1 | -5.189533 | 4.895954 | -3.050357 |
| 1 | -4.647815 | 5.925495 | 1.094080 |
| 1 | -4.876309 | 6.623136 | -1.282652 |
| 1 | -7.649440 | 2.445161 | 0.628951 |
| 1 | -6.361650 | -1.053971 | -1.511781 |
| 1 | -9.997407 | 1.932337 | 0.059477 |
| 1 | -8.710604 | -1.557125 | -2.107502 |
| 1 | -10.541932 | -0.070365 | -1.311577 |
| 1 | -6.988661 | 1.006319 | 2.421484 |
| 1 | -2.734389 | 0.854782 | 1.681848 |
| 1 | -6.550797 | 0.866156 | 4.844038 |
| 1 | -2.311482 | 0.733076 | 4.069029 |
| 1 | -4.218220 | 0.730589 | 5.694913 |
| 1 | -3.011601 | -5.434248 | 0.359684 |
| 1 | -3.753173 | -2.780483 | -2.945044 |
| 1 | -2.549470 | -7.274128 | -1.220016 |
| 1 | -3.331621 | -4.638496 | -4.530120 |
| 1 | -2.714940 | -6.891530 | -3.671569 |
| 1 | -5.647763 | -1.684504 | 2.057657 |
| 1 | -5.993856 | -4.339150 | -1.315519 |
| 1 | -8.030209 | -2.024044 | 2.572501 |
| 1 | -8.381037 | -4.671290 | -0.801643 |
| 1 | -9.421544 | -3.516147 | 1.141018 |
| 1 | -1.433370 | -1.555861 | 1.148963 |
| 1 | -4.484328 | -4.406222 | 2.232587 |
| 1 | -0.294112 | -1.967511 | 3.274241 |
| 1 | -3.338948 | -4.809953 | 4.378116 |
| 1 | -1.249261 | -3.567730 | 4.930175 |
| 1 | -2.322434 | 1.096967 | -3.012036 |
| 1 | 0.880635 | 1.019654 | -1.239792 |
| 6 | 0.737406 | 3.086020 | -0.785100 |
| 6 | -0.480493 | 4.067075 | -0.781150 |
| 1 | 0.886958 | 2.679191 | 0.215151 |
| 1 | 1.677440 | 3.503153 | -1.135236 |
| 7 | 0.362504 | 1.893641 | -1.605778 |
| 16 | 0.714492 | 1.941793 | -3.416179 |
| 8 | -0.371718 | 2.721480 | -4.010912 |
| 8 | 0.927487 | 0.543549 | -3.758172 |
| 6 | -1.646724 | 3.160026 | -1.299943 |
| 1 | -2.560290 | 3.277857 | -0.726582 |
| 1 | -1.881264 | 3.413816 | -2.331967 |
| 6 | -0.288299 | 5.292522 | -1.691595 |
| 6 | -1.416102 | 5.946757 | -2.212742 |
| 6 | 0.968691 | 5.853476 | -1.954989 |
| 6 | -1.290415 | 7.097221 | -2.989245 |
| 1 | -2.406800 | 5.559220 | -2.003097 |
| 6 | 1.099454 | 7.012704 | -2.725276 |
| 1 | 1.870422 | 5.397984 | -1.560678 |
| 6 | -0.029018 | 7.636652 | -3.253997 |

| | | | |
|---|---|---|---|
| 1 | -2.182376 | 7.574942 | -3.385471 |
| 1 | 2.089745 | 7.417691 | -2.913145 |
| 1 | 0.070460 | 8.533963 | -3.858076 |
| 6 | -0.691066 | 4.559077 | 0.675111 |
| 6 | -1.562268 | 3.896850 | 1.551012 |
| 6 | 0.030564 | 5.653241 | 1.178076 |
| 6 | -1.730007 | 4.324662 | 2.868506 |
| 1 | -2.112778 | 3.027456 | 1.225229 |
| 6 | -0.128543 | 6.081393 | 2.498233 |
| 1 | 0.711632 | 6.196830 | 0.533371 |
| 6 | -1.019100 | 5.425072 | 3.349356 |
| 1 | -2.414810 | 3.782998 | 3.515276 |
| 1 | 0.438579 | 6.937303 | 2.853986 |
| 1 | -1.152166 | 5.763086 | 4.373316 |
| 6 | 2.240296 | 2.875931 | -3.592798 |
| 6 | 3.514242 | 2.374431 | -3.163066 |
| 6 | 2.108790 | 4.065641 | -4.282469 |
| 6 | 3.714151 | 1.170745 | -2.441375 |
| 6 | 4.662908 | 3.180396 | -3.484569 |
| 6 | 3.252442 | 4.832674 | -4.590901 |
| 1 | 1.128820 | 4.411255 | -4.584939 |
| 6 | 4.980675 | 0.792994 | -2.045796 |
| 1 | 2.883860 | 0.525556 | -2.195490 |
| 6 | 5.952225 | 2.746842 | -3.076810 |
| 6 | 4.496165 | 4.397954 | -4.197848 |
| 1 | 3.133189 | 5.764002 | -5.133991 |
| 6 | 6.110318 | 1.576866 | -2.369017 |
| 1 | 5.103630 | -0.124007 | -1.480198 |
| 1 | 6.810578 | 3.362879 | -3.330632 |
| 1 | 5.380454 | 4.985267 | -4.430887 |
| 1 | 7.098585 | 1.254555 | -2.054968 |
| 6 | 3.873131 | -2.537911 | -0.357895 |
| 6 | 3.828297 | -3.161676 | -1.596591 |
| 6 | 5.062296 | -3.614129 | -2.174326 |
| 6 | 5.108701 | -4.370636 | -3.378900 |
| 1 | 4.178842 | -4.628097 | -3.872257 |
| 6 | 6.309814 | -4.779531 | -3.914334 |
| 1 | 6.319866 | -5.360516 | -4.832158 |
| 6 | 7.532913 | -4.451118 | -3.279890 |
| 1 | 8.472862 | -4.776633 | -3.716382 |
| 6 | 7.524658 | -3.731272 | -2.107757 |
| 1 | 8.455523 | -3.484179 | -1.603288 |
| 6 | 6.300267 | -3.305693 | -1.521567 |
| 6 | 6.281237 | -2.594164 | -0.298435 |
| 1 | 7.227075 | -2.341126 | 0.171742 |
| 6 | 5.096778 | -2.234430 | 0.319622 |
| 6 | 5.114466 | -1.605030 | 1.665089 |
| 6 | 6.013587 | -2.037637 | 2.624019 |
| 1 | 6.684189 | -2.860327 | 2.394116 |
| 6 | 6.072080 | -1.456510 | 3.913738 |
| 6 | 7.024723 | -1.885151 | 4.878712 |
| 1 | 7.727326 | -2.667976 | 4.604141 |
| 6 | 7.061804 | -1.319646 | 6.132813 |
| 1 | 7.795729 | -1.652398 | 6.861159 |
| 6 | 6.140649 | -0.300172 | 6.476792 |
| 1 | 6.177048 | 0.144510 | 7.467284 |
| 6 | 5.198693 | 0.130265 | 5.568308 |
| 1 | 4.494940 | 0.908881 | 5.840944 |
| 6 | 5.134511 | -0.430879 | 4.262980 |
| 6 | 4.165491 | -0.011657 | 3.295166 |
| 6 | 4.213704 | -0.555147 | 2.023653 |
| 6 | 2.528770 | -3.321296 | -2.327487 |

| | | | |
|---|---|---|---|
| 6 | 2.363109 | -2.603751 | -3.569353 |
| 6 | 3.356610 | -1.736260 | -4.105303 |
| 1 | 4.265886 | -1.558420 | -3.547166 |
| 6 | 3.185518 | -1.108337 | -5.319072 |
| 1 | 3.962314 | -0.446451 | -5.692324 |
| 6 | 2.004615 | -1.301228 | -6.070774 |
| 1 | 1.879708 | -0.799216 | -7.026196 |
| 6 | 1.006721 | -2.104217 | -5.570158 |
| 1 | 0.080049 | -2.243987 | -6.121822 |
| 6 | 1.153813 | -2.762482 | -4.320457 |
| 6 | 0.123820 | -3.579191 | -3.790649 |
| 1 | -0.804776 | -3.689235 | -4.343632 |
| 6 | 0.291698 | -4.225734 | -2.594134 |
| 1 | -0.515279 | -4.838937 | -2.209832 |
| 6 | 1.499998 | -4.128813 | -1.847276 |
| 6 | 3.065644 | 0.928770 | 3.679265 |
| 6 | 2.007009 | 0.406344 | 4.503080 |
| 6 | 1.948691 | -0.957701 | 4.904515 |
| 1 | 2.707974 | -1.645021 | 4.553214 |
| 6 | 0.933044 | -1.419961 | 5.711568 |
| 1 | 0.912448 | -2.466848 | 6.000379 |
| 6 | -0.092903 | -0.550117 | 6.153098 |
| 1 | -0.892575 | -0.930930 | 6.781655 |
| 6 | -0.079270 | 0.771356 | 5.768763 |
| 1 | -0.864954 | 1.451005 | 6.089724 |
| 6 | 0.958356 | 1.277491 | 4.939377 |
| 6 | 0.970348 | 2.628872 | 4.504470 |
| 1 | 0.166851 | 3.291372 | 4.810433 |
| 6 | 1.971705 | 3.091235 | 3.686062 |
| 1 | 1.938305 | 4.122668 | 3.349650 |
| 6 | 3.051301 | 2.257492 | 3.273622 |
| 8 | 1.583890 | -0.199728 | -0.690280 |
| 8 | 1.093722 | -1.028967 | 1.745319 |
| 8 | 2.686345 | -2.324087 | 0.309239 |
| 8 | 3.400278 | -0.025129 | 1.037549 |
| 15 | 2.034449 | -0.847495 | 0.594527 |
| 6 | 4.149380 | 2.862668 | 2.416339 |
| 6 | 3.613128 | 3.307420 | 1.038448 |
| 6 | 4.858373 | 4.034822 | 3.131395 |
| 1 | 4.906386 | 2.092368 | 2.232473 |
| 6 | 4.709585 | 3.941803 | 0.173417 |
| 1 | 2.797954 | 4.029955 | 1.183110 |
| 1 | 3.192013 | 2.436141 | 0.531772 |
| 6 | 5.957499 | 4.651785 | 2.253835 |
| 1 | 4.122789 | 4.811941 | 3.378942 |
| 1 | 5.277197 | 3.684825 | 4.082639 |
| 6 | 5.400289 | 5.104987 | 0.896282 |
| 1 | 4.288132 | 4.276715 | -0.782598 |
| 1 | 5.453809 | 3.175774 | -0.076921 |
| 1 | 6.426126 | 5.494292 | 2.777260 |
| 1 | 6.747817 | 3.906034 | 2.087242 |
| 1 | 6.202495 | 5.519508 | 0.273213 |
| 1 | 4.674644 | 5.916013 | 1.056454 |
| 6 | 1.610170 | -4.962679 | -0.582000 |
| 6 | 0.520226 | -4.607253 | 0.454542 |
| 6 | 1.573039 | -6.477027 | -0.898643 |
| 1 | 2.578414 | -4.765193 | -0.113124 |
| 6 | 0.660374 | -5.449903 | 1.728371 |
| 1 | -0.470984 | -4.782353 | 0.019881 |
| 1 | 0.575068 | -3.545875 | 0.701433 |
| 6 | 1.712291 | -7.318750 | 0.378286 |
| 1 | 0.624100 | -6.724045 | -1.394031 |

| | | | |
|---|---|---|---|
| 1 | 2.371458 | -6.725426 | -1.608901 |
| 6 | 0.639659 | -6.952449 | 1.414755 |
| 1 | -0.140595 | -5.191502 | 2.429176 |
| 1 | 1.607039 | -5.194270 | 2.226331 |
| 1 | 1.660365 | -8.387143 | 0.132488 |
| 1 | 2.706563 | -7.145675 | 0.814788 |
| 1 | 0.779467 | -7.540177 | 2.330943 |
| 1 | -0.350163 | -7.221309 | 1.017064 |
| 1 | 0.101476 | 0.350039 | 1.869849 |
| 8 | -0.405516 | 1.192297 | 1.800359 |
| 1 | -0.046246 | 1.770307 | 2.485100 |

------------------------------------------------------------

(*S*,*R*,*S*)-**3a**exo-*re*

------------------------------------------------------------

Number of imaginary frequencies : 0
The smallest frequencies are : 6.9100 13.5049 14.7911 cm(-1)

Electronic energy : HF=-6614.0124975
Zero-point correction= 1.889355 (Hartree/Particle)
Thermal correction to Energy= 2.000324
Thermal correction to Enthalpy= 2.001268
Thermal correction to Gibbs Free Energy= 1.735672
Sum of electronic and zero-point Energies= -6612.123143
Sum of electronic and thermal Energies= -6612.012174
Sum of electronic and thermal Enthalpies= -6612.011230
Sum of electronic and thermal Free Energies= -6612.276825

..........................................................

Cartesian Coordinates

..........................................................

| | | | |
|---|---|---|---|
| 6 | 6.918279 | -0.417622 | -1.087712 |
| 6 | 6.824315 | 0.356102 | 0.081157 |
| 6 | 7.952808 | 0.456995 | 0.907864 |
| 6 | 9.136825 | -0.205143 | 0.577772 |
| 6 | 9.215499 | -0.976218 | -0.584683 |
| 6 | 8.102572 | -1.076384 | -1.421112 |
| 15 | 5.160904 | 1.093460 | 0.463955 |
| 6 | 5.159407 | 1.397842 | 2.282917 |
| 6 | 6.038329 | 2.276608 | 2.944021 |
| 6 | 5.965074 | 2.445635 | 4.326171 |
| 6 | 5.009614 | 1.744196 | 5.068793 |
| 6 | 4.126273 | 0.880962 | 4.421265 |
| 6 | 4.198192 | 0.708763 | 3.036600 |
| 46 | 3.447324 | -0.314479 | -0.387801 |
| 6 | 1.781593 | -0.669130 | -1.753350 |
| 6 | 2.037670 | 0.713474 | -1.787844 |
| 6 | 1.203722 | 1.680935 | -1.016982 |
| 15 | 4.008233 | -2.489950 | 0.401638 |
| 6 | 4.915807 | -2.568182 | 2.012302 |
| 6 | 6.289146 | -2.277925 | 2.072283 |
| 6 | 6.944051 | -2.187036 | 3.300391 |
| 6 | 6.239978 | -2.380906 | 4.490421 |
| 6 | 4.875569 | -2.672261 | 4.441202 |
| 6 | 4.217323 | -2.765983 | 3.214222 |
| 6 | 5.360563 | 2.793488 | -0.260005 |
| 6 | 5.020224 | 3.968319 | 0.429415 |
| 6 | 5.101304 | 5.214799 | -0.197412 |
| 6 | 5.508977 | 5.309605 | -1.528335 |
| 6 | 5.832930 | 4.145045 | -2.231077 |
| 6 | 5.759626 | 2.902343 | -1.603830 |
| 6 | 2.622869 | -3.675107 | 0.722078 |
| 6 | 2.771778 | -5.071549 | 0.704505 |

| | | | |
|---|---|---|---|
| 6 | 1.686535 | -5.896650 | 1.002845 |
| 6 | 0.448168 | -5.342219 | 1.343622 |
| 6 | 0.295982 | -3.955337 | 1.369699 |
| 6 | 1.375375 | -3.129434 | 1.047021 |
| 6 | 5.100281 | -3.421628 | -0.765755 |
| 6 | 4.992307 | -3.119141 | -2.133061 |
| 6 | 5.773170 | -3.788703 | -3.076237 |
| 6 | 6.681854 | -4.767408 | -2.665532 |
| 6 | 6.799185 | -5.075323 | -1.308442 |
| 6 | 6.012789 | -4.410223 | -0.365367 |
| 1 | 0.877330 | 1.230632 | -0.079078 |
| 1 | 2.096992 | -1.285710 | -2.588923 |
| 1 | 0.937007 | -1.054078 | -1.191837 |
| 1 | 4.687681 | 3.918975 | 1.460126 |
| 1 | 6.016292 | 2.009281 | -2.166104 |
| 1 | 4.832589 | 6.109755 | 0.356523 |
| 1 | 6.147091 | 4.204093 | -3.269649 |
| 1 | 5.569290 | 6.278646 | -2.015862 |
| 1 | 6.766798 | 2.848042 | 2.377193 |
| 1 | 3.491184 | 0.051816 | 2.542309 |
| 1 | 6.649946 | 3.127707 | 4.822832 |
| 1 | 3.373862 | 0.337846 | 4.984924 |
| 1 | 4.953703 | 1.877571 | 6.145888 |
| 1 | 7.905742 | 1.014978 | 1.834807 |
| 1 | 6.041547 | -0.542516 | -1.715362 |
| 1 | 9.995734 | -0.127871 | 1.239160 |
| 1 | 8.138584 | -1.692441 | -2.314804 |
| 1 | 10.133451 | -1.503066 | -0.829995 |
| 1 | 3.726112 | -5.518919 | 0.444883 |
| 1 | 1.244264 | -2.059223 | 1.031362 |
| 1 | 1.805872 | -6.976252 | 0.966439 |
| 1 | -0.664318 | -3.505841 | 1.600349 |
| 1 | -0.396440 | -5.989068 | 1.560450 |
| 1 | 6.852862 | -2.124019 | 1.159905 |
| 1 | 3.154311 | -2.982232 | 3.196152 |
| 1 | 8.005566 | -1.956645 | 3.321621 |
| 1 | 4.316459 | -2.825510 | 5.360371 |
| 1 | 6.749115 | -2.302670 | 5.447015 |
| 1 | 4.294301 | -2.351514 | -2.452275 |
| 1 | 6.120832 | -4.653523 | 0.686564 |
| 1 | 5.677218 | -3.539044 | -4.129533 |
| 1 | 7.504228 | -5.834372 | -0.980700 |
| 1 | 7.296199 | -5.284528 | -3.397438 |
| 1 | 2.579953 | 1.139684 | -2.629887 |
| 1 | -1.020652 | 1.453263 | -1.233895 |
| 6 | -0.511683 | 3.491026 | -1.239807 |
| 6 | 0.612499 | 3.912151 | -0.261207 |
| 1 | -0.439221 | 4.135359 | -2.112155 |
| 1 | -1.526548 | 3.550652 | -0.847892 |
| 7 | -0.228635 | 2.070666 | -1.654260 |
| 16 | -0.361902 | 1.830378 | -3.447020 |
| 8 | -0.415194 | 0.388132 | -3.640929 |
| 8 | 0.704154 | 2.647016 | -4.031140 |
| 6 | 1.785471 | 3.070443 | -0.810707 |
| 1 | 2.132461 | 3.450320 | -1.776338 |
| 1 | 2.636095 | 3.033265 | -0.135429 |
| 6 | 0.373650 | 3.504977 | 1.200788 |
| 6 | 1.366493 | 3.823318 | 2.143272 |
| 6 | -0.742568 | 2.780713 | 1.628281 |
| 6 | 1.250495 | 3.420951 | 3.472410 |
| 1 | 2.238052 | 4.389023 | 1.826588 |
| 6 | -0.859260 | 2.370651 | 2.960293 |

| | | | |
|---|---|---|---|
| 1 | -1.530018 | 2.501982 | 0.940283 |
| 6 | 0.131870 | 2.688543 | 3.886562 |
| 1 | 2.039048 | 3.663431 | 4.178719 |
| 1 | -1.722934 | 1.787495 | 3.252133 |
| 1 | 0.044725 | 2.357092 | 4.917052 |
| 6 | 0.757135 | 5.432865 | -0.366288 |
| 6 | 1.823236 | 6.053505 | -1.025094 |
| 6 | -0.263942 | 6.237002 | 0.166322 |
| 6 | 1.871047 | 7.446072 | -1.145323 |
| 1 | 2.631728 | 5.463788 | -1.439100 |
| 6 | -0.222880 | 7.623705 | 0.036569 |
| 1 | -1.093979 | 5.766344 | 0.685548 |
| 6 | 0.848944 | 8.235997 | -0.620054 |
| 1 | 2.714220 | 7.908688 | -1.651536 |
| 1 | -1.024922 | 8.227639 | 0.452560 |
| 1 | 0.886602 | 9.317492 | -0.716412 |
| 6 | -1.994588 | 2.513689 | -3.775141 |
| 6 | -2.234710 | 3.868575 | -4.190231 |
| 6 | -3.020337 | 1.605697 | -3.577638 |
| 6 | -1.241958 | 4.864393 | -4.411940 |
| 6 | -3.612906 | 4.239592 | -4.380770 |
| 6 | -4.357808 | 1.994246 | -3.792648 |
| 1 | -2.800366 | 0.598384 | -3.248278 |
| 6 | -1.594889 | 6.148518 | -4.766699 |
| 1 | -0.195943 | 4.611837 | -4.309265 |
| 6 | -3.934148 | 5.571831 | -4.758179 |
| 6 | -4.642158 | 3.280843 | -4.185656 |
| 1 | -5.151111 | 1.273392 | -3.634580 |
| 6 | -2.949122 | 6.513532 | -4.940328 |
| 1 | -0.815029 | 6.889134 | -4.916659 |
| 1 | -4.980664 | 5.831166 | -4.893853 |
| 1 | -5.672126 | 3.589224 | -4.343763 |
| 1 | -3.205897 | 7.530533 | -5.220600 |
| 6 | -4.195107 | -0.738731 | 1.640702 |
| 6 | -5.055074 | 0.207003 | 2.168847 |
| 6 | -6.440713 | -0.126166 | 2.332150 |
| 6 | -7.394056 | 0.808037 | 2.822701 |
| 1 | -7.066852 | 1.810832 | 3.073516 |
| 6 | -8.717083 | 0.453336 | 2.976086 |
| 1 | -9.430793 | 1.183395 | 3.347264 |
| 6 | -9.156718 | -0.852919 | 2.652180 |
| 1 | -10.202440 | -1.118111 | 2.778609 |
| 6 | -8.258161 | -1.781700 | 2.177901 |
| 1 | -8.583050 | -2.787885 | 1.925282 |
| 6 | -6.887279 | -1.447363 | 2.008718 |
| 6 | -5.948240 | -2.392807 | 1.528208 |
| 1 | -6.293890 | -3.390744 | 1.274503 |
| 6 | -4.617749 | -2.070873 | 1.335588 |
| 6 | -3.711057 | -3.102354 | 0.764072 |
| 6 | -3.730818 | -4.412364 | 1.201537 |
| 1 | -4.273635 | -4.666675 | 2.107541 |
| 6 | -3.089161 | -5.442434 | 0.468330 |
| 6 | -3.083635 | -6.785175 | 0.936421 |
| 1 | -3.532905 | -7.000297 | 1.902734 |
| 6 | -2.528479 | -7.794384 | 0.182347 |
| 1 | -2.526361 | -8.815707 | 0.551989 |
| 6 | -1.970802 | -7.502570 | -1.085987 |
| 1 | -1.546296 | -8.304222 | -1.683469 |
| 6 | -1.961224 | -6.212533 | -1.569406 |
| 1 | -1.526988 | -6.001113 | -2.539304 |
| 6 | -2.490437 | -5.138899 | -0.801810 |
| 6 | -2.439424 | -3.776592 | -1.244761 |

| | | | |
|---|---|---|---|
| 6 | -2.958299 | -2.804510 | -0.407808 |
| 6 | -4.551038 | 1.519169 | 2.690165 |
| 6 | -4.377580 | 2.638340 | 1.812864 |
| 6 | -4.584451 | 2.548012 | 0.408997 |
| 1 | -4.882099 | 1.598878 | -0.019473 |
| 6 | -4.369682 | 3.632968 | -0.411699 |
| 1 | -4.512603 | 3.531917 | -1.481707 |
| 6 | -3.942229 | 4.871475 | 0.125180 |
| 1 | -3.766607 | 5.714464 | -0.537509 |
| 6 | -3.744821 | 4.999269 | 1.482655 |
| 1 | -3.417752 | 5.944766 | 1.908353 |
| 6 | -3.955167 | 3.897513 | 2.355557 |
| 6 | -3.734194 | 4.000372 | 3.751904 |
| 1 | -3.409909 | 4.952368 | 4.164129 |
| 6 | -3.893834 | 2.907948 | 4.568649 |
| 1 | -3.689070 | 3.005219 | 5.629792 |
| 6 | -4.298967 | 1.644814 | 4.056403 |
| 6 | -2.022950 | -3.385487 | -2.632259 |
| 6 | -0.653003 | -3.468675 | -3.055232 |
| 6 | 0.381213 | -3.962937 | -2.212767 |
| 1 | 0.144784 | -4.250516 | -1.197806 |
| 6 | 1.673517 | -4.091397 | -2.671959 |
| 1 | 2.438866 | -4.479419 | -2.010311 |
| 6 | 2.014685 | -3.700845 | -3.988095 |
| 1 | 3.040551 | -3.803727 | -4.329072 |
| 6 | 1.047753 | -3.185979 | -4.820975 |
| 1 | 1.295774 | -2.871837 | -5.831957 |
| 6 | -0.300051 | -3.068122 | -4.386541 |
| 6 | -1.313058 | -2.576253 | -5.246411 |
| 1 | -1.042393 | -2.250881 | -6.247784 |
| 6 | -2.618300 | -2.528594 | -4.827244 |
| 1 | -3.383381 | -2.168629 | -5.509010 |
| 6 | -3.004485 | -2.943345 | -3.523115 |
| 8 | -2.281208 | 0.910990 | -0.635066 |
| 8 | -0.650353 | -0.848190 | 0.387001 |
| 8 | -2.853878 | -0.427095 | 1.532240 |
| 8 | -2.903660 | -1.482458 | -0.807743 |
| 15 | -2.046299 | -0.400116 | 0.088626 |
| 6 | -4.485809 | -2.908640 | -3.176145 |
| 6 | -5.070741 | -1.479470 | -3.201502 |
| 6 | -5.320675 | -3.846869 | -4.076688 |
| 1 | -4.612882 | -3.277241 | -2.155606 |
| 6 | -6.547962 | -1.475867 | -2.781731 |
| 1 | -4.980011 | -1.066015 | -4.215832 |
| 1 | -4.485932 | -0.842446 | -2.533011 |
| 6 | -6.798626 | -3.842781 | -3.656060 |
| 1 | -5.239508 | -3.527053 | -5.124423 |
| 1 | -4.910935 | -4.862670 | -4.025653 |
| 6 | -7.382903 | -2.422149 | -3.656513 |
| 1 | -6.953502 | -0.456781 | -2.828033 |
| 1 | -6.620248 | -1.788624 | -1.730580 |
| 1 | -7.380928 | -4.495174 | -4.318360 |
| 1 | -6.883276 | -4.266003 | -2.644772 |
| 1 | -8.425356 | -2.439050 | -3.315323 |
| 1 | -7.395674 | -2.040084 | -4.687789 |
| 6 | -4.357834 | 0.438022 | 4.978100 |
| 6 | -2.949209 | -0.184389 | 5.139859 |
| 6 | -4.987506 | 0.703618 | 6.359718 |
| 1 | -4.979999 | -0.323610 | 4.498019 |
| 6 | -3.002686 | -1.470191 | 5.975703 |
| 1 | -2.290832 | 0.548941 | 5.627736 |
| 1 | -2.520062 | -0.388545 | 4.153195 |

| | | | |
|---|---|---|---|
| 6 | -5.054519 | -0.592298 | 7.182825 |
| 1 | -4.391522 | 1.440841 | 6.913744 |
| 1 | -5.989161 | 1.133010 | 6.236135 |
| 6 | -3.665381 | -1.229808 | 7.340280 |
| 1 | -1.992170 | -1.875945 | 6.104930 |
| 1 | -3.576489 | -2.227177 | 5.422128 |
| 1 | -5.495363 | -0.390383 | 8.166699 |
| 1 | -5.723917 | -1.303743 | 6.678437 |
| 1 | -3.739730 | -2.170207 | 7.899952 |
| 1 | -3.028685 | -0.559088 | 7.935394 |
| 1 | 0.480828 | -0.298121 | 1.685233 |
| 8 | 1.211593 | -0.081110 | 2.298253 |
| 1 | 1.178282 | 0.878563 | 2.388383 |

------------------------------------------------------------------------------------------------------------------------

(*S*,*R*,*S*)-**3a**endo-*si*

------------------------------------------------------------------------------------------------------------------------

Number of imaginary frequencies : 0
The smallest frequencies are : 9.0658 12.8686 13.2523 cm(-1)

Electronic energy : HF=-6614.0068614
Zero-point correction= 1.889139 (Hartree/Particle)
Thermal correction to Energy= 2.000638
Thermal correction to Enthalpy= 2.001583
Thermal correction to Gibbs Free Energy= 1.733817
Sum of electronic and zero-point Energies= -6612.117723
Sum of electronic and thermal Energies= -6612.006223
Sum of electronic and thermal Enthalpies= -6612.005279
Sum of electronic and thermal Free Energies= -6612.273044

............................................................

Cartesian Coordinates

............................................................

| | | | |
|---|---|---|---|
| 6 | 6.795961 | -0.925195 | -1.236535 |
| 6 | 6.955727 | -0.087145 | -0.120293 |
| 6 | 8.199499 | -0.057002 | 0.525353 |
| 6 | 9.252147 | -0.853845 | 0.070424 |
| 6 | 9.079892 | -1.689035 | -1.036349 |
| 6 | 7.848229 | -1.719053 | -1.694275 |
| 15 | 5.458622 | 0.852670 | 0.441876 |
| 6 | 5.770507 | 1.235691 | 2.218269 |
| 6 | 6.751603 | 2.140736 | 2.659981 |
| 6 | 6.944941 | 2.364928 | 4.022872 |
| 6 | 6.158210 | 1.693069 | 4.963279 |
| 6 | 5.172773 | 0.803099 | 4.535055 |
| 6 | 4.976642 | 0.579601 | 3.170853 |
| 46 | 3.512903 | -0.406304 | 0.008427 |
| 6 | 1.556627 | -0.743378 | -0.806813 |
| 6 | 1.755116 | 0.651193 | -0.764688 |
| 6 | 1.825302 | 1.410914 | -2.053526 |
| 15 | 3.996336 | -2.628993 | 0.729504 |
| 6 | 5.198962 | -2.858536 | 2.115822 |
| 6 | 6.581332 | -2.783125 | 1.874845 |
| 6 | 7.488981 | -2.806724 | 2.933546 |
| 6 | 7.034770 | -2.899282 | 4.250717 |
| 6 | 5.663453 | -2.977604 | 4.500336 |
| 6 | 4.752070 | -2.959331 | 3.443179 |
| 6 | 5.723232 | 2.494064 | -0.379712 |
| 6 | 5.261212 | 3.677241 | 0.221725 |
| 6 | 5.391488 | 4.905393 | -0.428619 |
| 6 | 5.968653 | 4.972974 | -1.699417 |
| 6 | 6.408642 | 3.800095 | -2.316466 |
| 6 | 6.289650 | 2.572462 | -1.662239 |

| | | | |
|---|---|---|---|
| 6 | 2.509271 | -3.544785 | 1.340807 |
| 6 | 2.316358 | -4.924068 | 1.159331 |
| 6 | 1.153594 | -5.539289 | 1.626223 |
| 6 | 0.173564 | -4.792366 | 2.286016 |
| 6 | 0.363558 | -3.423589 | 2.479531 |
| 6 | 1.521404 | -2.802556 | 2.006860 |
| 6 | 4.652182 | -3.706223 | -0.619181 |
| 6 | 4.332257 | -3.357617 | -1.941828 |
| 6 | 4.778489 | -4.134818 | -3.011924 |
| 6 | 5.557492 | -5.270099 | -2.774161 |
| 6 | 5.884195 | -5.623943 | -1.462968 |
| 6 | 5.434227 | -4.849231 | -0.391977 |
| 1 | 2.334231 | 0.807166 | -2.803980 |
| 1 | 0.931470 | -1.229488 | -0.071059 |
| 1 | 1.598503 | -1.245501 | -1.770832 |
| 1 | 4.799703 | 3.640347 | 1.202988 |
| 1 | 6.652670 | 1.674324 | -2.152063 |
| 1 | 5.036042 | 5.810266 | 0.056595 |
| 1 | 6.857048 | 3.838877 | -3.305461 |
| 1 | 6.072261 | 5.929899 | -2.202931 |
| 1 | 7.354620 | 2.682627 | 1.937788 |
| 1 | 4.202308 | -0.104731 | 2.840694 |
| 1 | 7.707346 | 3.066251 | 4.350896 |
| 1 | 4.552345 | 0.280728 | 5.257147 |
| 1 | 6.310071 | 1.869656 | 6.024570 |
| 1 | 8.345358 | 0.559853 | 1.404840 |
| 1 | 5.827957 | -0.983546 | -1.724331 |
| 1 | 10.205927 | -0.829835 | 0.590610 |
| 1 | 7.692526 | -2.377722 | -2.543679 |
| 1 | 9.897633 | -2.317221 | -1.378343 |
| 1 | 3.058079 | -5.516773 | 0.633881 |
| 1 | 1.634487 | -1.732607 | 2.147637 |
| 1 | 1.004264 | -6.602785 | 1.460309 |
| 1 | -0.402607 | -2.821328 | 2.955565 |
| 1 | -0.738788 | -5.271493 | 2.627824 |
| 1 | 6.953681 | -2.708118 | 0.859914 |
| 1 | 3.689720 | -3.019409 | 3.656546 |
| 1 | 8.552815 | -2.745271 | 2.722606 |
| 1 | 5.297619 | -3.054000 | 5.520679 |
| 1 | 7.743254 | -2.910469 | 5.074277 |
| 1 | 3.735658 | -2.468958 | -2.126239 |
| 1 | 5.699581 | -5.130533 | 0.622122 |
| 1 | 4.522877 | -3.848508 | -4.028454 |
| 1 | 6.490165 | -6.505257 | -1.271542 |
| 1 | 5.910218 | -5.874098 | -3.605516 |
| 1 | 1.347738 | 1.207272 | 0.073262 |
| 1 | -0.349214 | 1.093139 | -1.914217 |
| 6 | 0.015551 | 3.065016 | -2.594800 |
| 6 | 1.109762 | 3.758949 | -1.744940 |
| 1 | 0.112620 | 3.417855 | -3.619797 |
| 1 | -1.010068 | 3.214601 | -2.260698 |
| 7 | 0.331365 | 1.593551 | -2.572929 |
| 16 | 0.101115 | 0.771644 | -4.216895 |
| 8 | 0.752132 | -0.525155 | -4.051790 |
| 8 | 0.611838 | 1.734294 | -5.195149 |
| 6 | 2.329246 | 2.854211 | -2.063143 |
| 1 | 2.697932 | 3.087792 | -3.066449 |
| 1 | 3.151105 | 2.979115 | -1.359890 |
| 6 | 0.830246 | 3.753627 | -0.233664 |
| 6 | 1.799585 | 4.309108 | 0.616659 |
| 6 | -0.302727 | 3.171643 | 0.339078 |
| 6 | 1.663238 | 4.246310 | 2.001315 |

| | | | |
|---|---|---|---|
| 1 | 2.673997 | 4.782783 | 0.184375 |
| 6 | -0.437065 | 3.097333 | 1.727927 |
| 1 | -1.082780 | 2.725455 | -0.263198 |
| 6 | 0.543278 | 3.627032 | 2.565657 |
| 1 | 2.431386 | 4.675021 | 2.639500 |
| 1 | -1.314303 | 2.614855 | 2.136333 |
| 1 | 0.434108 | 3.556988 | 3.643564 |
| 6 | 1.263257 | 5.207442 | -2.220838 |
| 6 | 2.486261 | 5.742138 | -2.640289 |
| 6 | 0.132673 | 6.039138 | -2.219652 |
| 6 | 2.574563 | 7.074011 | -3.057055 |
| 1 | 3.383128 | 5.134280 | -2.632774 |
| 6 | 0.217518 | 7.363643 | -2.643689 |
| 1 | -0.815828 | 5.646373 | -1.868864 |
| 6 | 1.442426 | 7.888133 | -3.066734 |
| 1 | 3.534431 | 7.471134 | -3.376327 |
| 1 | -0.671711 | 7.988552 | -2.639605 |
| 1 | 1.512531 | 8.921585 | -3.394132 |
| 6 | -1.662739 | 0.559070 | -4.288221 |
| 6 | -2.566632 | 1.567967 | -4.767529 |
| 6 | -2.097337 | -0.633606 | -3.740797 |
| 6 | -2.202064 | 2.817576 | -5.339141 |
| 6 | -3.968200 | 1.261091 | -4.652175 |
| 6 | -3.474221 | -0.906334 | -3.651593 |
| 1 | -1.387350 | -1.361383 | -3.369615 |
| 6 | -3.165204 | 3.716200 | -5.748047 |
| 1 | -1.158710 | 3.063327 | -5.476931 |
| 6 | -4.928968 | 2.208958 | -5.095836 |
| 6 | -4.383593 | 0.023580 | -4.090813 |
| 1 | -3.790653 | -1.839366 | -3.211015 |
| 6 | -4.540892 | 3.418005 | -5.626054 |
| 1 | -2.856658 | 4.664491 | -6.178130 |
| 1 | -5.982019 | 1.959464 | -4.998941 |
| 1 | -5.448658 | -0.175155 | -4.005589 |
| 1 | -5.283893 | 4.137852 | -5.955286 |
| 6 | -4.121656 | 0.532347 | 1.671432 |
| 6 | -4.783429 | 1.701760 | 2.022031 |
| 6 | -6.111239 | 1.643485 | 2.549578 |
| 6 | -6.863503 | 2.809627 | 2.866807 |
| 1 | -6.419795 | 3.784163 | 2.699191 |
| 6 | -8.141354 | 2.710272 | 3.372679 |
| 1 | -8.700687 | 3.612991 | 3.602006 |
| 6 | -8.736512 | 1.443612 | 3.593023 |
| 1 | -9.744269 | 1.382266 | 3.993565 |
| 6 | -8.035965 | 0.297014 | 3.295926 |
| 1 | -8.480897 | -0.681696 | 3.458007 |
| 6 | -6.715808 | 0.366601 | 2.772669 |
| 6 | -5.972671 | -0.796841 | 2.471483 |
| 1 | -6.432289 | -1.765009 | 2.646342 |
| 6 | -4.693234 | -0.754503 | 1.940086 |
| 6 | -4.025795 | -2.058248 | 1.681699 |
| 6 | -4.094631 | -3.085971 | 2.603236 |
| 1 | -4.486382 | -2.888140 | 3.596788 |
| 6 | -3.666142 | -4.398432 | 2.280283 |
| 6 | -3.670318 | -5.445386 | 3.241229 |
| 1 | -3.974323 | -5.215966 | 4.259374 |
| 6 | -3.295587 | -6.725074 | 2.894340 |
| 1 | -3.297785 | -7.515375 | 3.639577 |
| 6 | -2.907762 | -7.013190 | 1.563182 |
| 1 | -2.611376 | -8.023595 | 1.296104 |
| 6 | -2.895429 | -6.020748 | 0.606766 |
| 1 | -2.583523 | -6.243577 | -0.407724 |

| | | | |
|---|---|---|---|
| 6 | -3.258773 | -4.687149 | 0.936519 |
| 6 | -3.195852 | -3.623361 | -0.019808 |
| 6 | -3.464113 | -2.339157 | 0.403939 |
| 6 | -4.077707 | 3.018616 | 1.945200 |
| 6 | -3.910161 | 3.677035 | 0.683282 |
| 6 | -4.290783 | 3.074274 | -0.547722 |
| 1 | -4.723048 | 2.079911 | -0.536086 |
| 6 | -4.094939 | 3.722718 | -1.747217 |
| 1 | -4.377917 | 3.239695 | -2.675667 |
| 6 | -3.523662 | 5.018164 | -1.779767 |
| 1 | -3.380418 | 5.519015 | -2.733186 |
| 6 | -3.147298 | 5.632658 | -0.605467 |
| 1 | -2.703615 | 6.625124 | -0.615811 |
| 6 | -3.313645 | 4.979436 | 0.644970 |
| 6 | -2.881373 | 5.571788 | 1.859224 |
| 1 | -2.418803 | 6.554959 | 1.829999 |
| 6 | -3.009293 | 4.897514 | 3.047290 |
| 1 | -2.635177 | 5.350979 | 3.960053 |
| 6 | -3.604771 | 3.607242 | 3.116596 |
| 6 | -2.996477 | -3.919757 | -1.472966 |
| 6 | -1.686281 | -3.966063 | -2.044019 |
| 6 | -0.516441 | -3.718352 | -1.273893 |
| 1 | -0.612693 | -3.437968 | -0.231069 |
| 6 | 0.731472 | -3.821363 | -1.848176 |
| 1 | 1.611352 | -3.647920 | -1.243078 |
| 6 | 0.877213 | -4.142788 | -3.218392 |
| 1 | 1.873018 | -4.211824 | -3.646520 |
| 6 | -0.236018 | -4.361702 | -3.997522 |
| 1 | -0.136704 | -4.601696 | -5.053333 |
| 6 | -1.538657 | -4.290685 | -3.434366 |
| 6 | -2.704187 | -4.537968 | -4.204172 |
| 1 | -2.596312 | -4.772899 | -5.260326 |
| 6 | -3.950880 | -4.490083 | -3.628320 |
| 1 | -4.825497 | -4.682036 | -4.241350 |
| 6 | -4.124699 | -4.190188 | -2.248656 |
| 8 | -1.666438 | 0.623196 | -1.095680 |
| 8 | -1.017617 | -1.019973 | 0.864778 |
| 8 | -2.862000 | 0.704625 | 1.158048 |
| 8 | -3.240898 | -1.269667 | -0.451579 |
| 15 | -2.033619 | -0.247696 | 0.078721 |
| 6 | -5.526182 | -4.104063 | -1.661136 |
| 6 | -6.150718 | -2.708309 | -1.903235 |
| 6 | -6.488207 | -5.208985 | -2.140906 |
| 1 | -5.440568 | -4.221627 | -0.576267 |
| 6 | -7.520574 | -2.582808 | -1.221694 |
| 1 | -6.259095 | -2.553596 | -2.987264 |
| 1 | -5.473235 | -1.930625 | -1.536670 |
| 6 | -7.849942 | -5.088451 | -1.439272 |
| 1 | -6.644814 | -5.132945 | -3.225212 |
| 1 | -6.043688 | -6.193711 | -1.953563 |
| 6 | -8.474376 | -3.702673 | -1.661756 |
| 1 | -7.958409 | -1.599678 | -1.433065 |
| 1 | -7.377743 | -2.631696 | -0.133027 |
| 1 | -8.527679 | -5.873387 | -1.796486 |
| 1 | -7.715463 | -5.255881 | -0.361232 |
| 1 | -9.428548 | -3.624461 | -1.126455 |
| 1 | -8.702711 | -3.580428 | -2.730640 |
| 6 | -3.653369 | 2.891838 | 4.457441 |
| 6 | -2.238826 | 2.508679 | 4.956476 |
| 6 | -4.412682 | 3.691801 | 5.537914 |
| 1 | -4.195805 | 1.951952 | 4.322582 |
| 6 | -2.308003 | 1.744275 | 6.286780 |

| | | | |
|---|---|---|---|
| 1 | -1.639943 | 3.420631 | 5.090344 |
| 1 | -1.724574 | 1.896298 | 4.207450 |
| 6 | -4.479764 | 2.915116 | 6.860832 |
| 1 | -3.910096 | 4.653214 | 5.710799 |
| 1 | -5.422436 | 3.921466 | 5.178602 |
| 6 | -3.077292 | 2.532402 | 7.356199 |
| 1 | -1.294613 | 1.509815 | 6.634102 |
| 1 | -2.809882 | 0.781424 | 6.116629 |
| 1 | -5.005768 | 3.508407 | 7.619040 |
| 1 | -5.072600 | 2.001865 | 6.708943 |
| 1 | -3.145272 | 1.951548 | 8.284391 |
| 1 | -2.520071 | 3.449175 | 7.598713 |
| 1 | -0.063299 | -0.219498 | 2.172598 |
| 8 | 0.514815 | 0.196971 | 2.846786 |
| 1 | 0.800340 | 1.017737 | 2.426585 |

-----------------------------------------------------------------------------------------------------------------------

(*S*,*S*,*S*)-**3a**endo-*re*

-----------------------------------------------------------------------------------------------------------------------

Number of imaginary frequencies : 0
The smallest frequencies are : 9.4263 13.7529 15.6752 cm(-1)

Electronic energy : HF=-6614.0009637
Zero-point correction= 1.889343 (Hartree/Particle)
Thermal correction to Energy= 2.000499
Thermal correction to Enthalpy= 2.001444
Thermal correction to Gibbs Free Energy= 1.735810
Sum of electronic and zero-point Energies= -6612.111620
Sum of electronic and thermal Energies= -6612.000464
Sum of electronic and thermal Enthalpies= -6611.999520
Sum of electronic and thermal Free Energies= -6612.265154

............................................................

Cartesian Coordinates

............................................................

| | | | |
|---|---|---|---|
| 6 | -6.837606 | 0.003321 | -1.472035 |
| 6 | -6.837823 | 0.744908 | -0.278645 |
| 6 | -8.049995 | 0.917594 | 0.403511 |
| 6 | -9.229801 | 0.363480 | -0.097139 |
| 6 | -9.218639 | -0.368169 | -1.287265 |
| 6 | -8.017362 | -0.544782 | -1.976970 |
| 15 | -5.195410 | 1.381471 | 0.306088 |
| 6 | -5.392824 | 1.709120 | 2.107084 |
| 6 | -6.261105 | 2.677285 | 2.642915 |
| 6 | -6.345349 | 2.866814 | 4.022045 |
| 6 | -5.561431 | 2.094769 | 4.884974 |
| 6 | -4.693021 | 1.135679 | 4.363552 |
| 6 | -4.606745 | 0.944456 | 2.982984 |
| 46 | -3.458728 | -0.107912 | -0.271323 |
| 6 | -1.548709 | -0.571292 | -1.130564 |
| 6 | -1.555283 | 0.805852 | -0.820802 |
| 6 | -1.394647 | 1.795578 | -1.934801 |
| 15 | -4.343124 | -2.275634 | 0.229022 |
| 6 | -5.540443 | -2.343567 | 1.636115 |
| 6 | -6.905942 | -2.070224 | 1.455938 |
| 6 | -7.763408 | -1.984658 | 2.553920 |
| 6 | -7.272927 | -2.162191 | 3.848481 |
| 6 | -5.915852 | -2.432018 | 4.037862 |
| 6 | -5.056375 | -2.523050 | 2.943211 |
| 6 | -5.196913 | 3.072361 | -0.462923 |
| 6 | -4.808293 | 4.224636 | 0.237712 |
| 6 | -4.740253 | 5.463518 | -0.405761 |
| 6 | -5.045945 | 5.571769 | -1.763229 |

| | | | |
|---|---|---|---|
| 6 | -5.414007 | 4.427386 | -2.476365 |
| 6 | -5.489508 | 3.192805 | -1.832979 |
| 6 | -3.111473 | -3.556922 | 0.753499 |
| 6 | -1.886513 | -3.116102 | 1.269684 |
| 6 | -0.926074 | -4.028087 | 1.712527 |
| 6 | -1.188513 | -5.397034 | 1.660804 |
| 6 | -2.416632 | -5.846862 | 1.161711 |
| 6 | -3.370208 | -4.936533 | 0.703860 |
| 6 | -5.208756 | -3.091842 | -1.182240 |
| 6 | -4.780797 | -2.743174 | -2.473727 |
| 6 | -5.351149 | -3.339478 | -3.599069 |
| 6 | -6.368497 | -4.285627 | -3.448677 |
| 6 | -6.805800 | -4.635256 | -2.169024 |
| 6 | -6.227495 | -4.046656 | -1.042488 |
| 1 | -1.888360 | 1.418035 | -2.830205 |
| 1 | -1.013319 | -1.267157 | -0.500657 |
| 1 | -1.634267 | -0.864872 | -2.173995 |
| 1 | -4.550358 | 4.160243 | 1.288170 |
| 1 | -5.784694 | 2.318202 | -2.404241 |
| 1 | -4.440329 | 6.342969 | 0.157547 |
| 1 | -5.649485 | 4.495823 | -3.534892 |
| 1 | -4.994416 | 6.535593 | -2.261808 |
| 1 | -6.857768 | 3.298117 | 1.981525 |
| 1 | -3.922840 | 0.201211 | 2.585582 |
| 1 | -7.019139 | 3.619293 | 4.422570 |
| 1 | -4.076665 | 0.533739 | 5.024538 |
| 1 | -5.626245 | 2.245942 | 5.959043 |
| 1 | -8.078208 | 1.452057 | 1.345767 |
| 1 | -5.900382 | -0.178352 | -1.989168 |
| 1 | -10.158500 | 0.496698 | 0.451244 |
| 1 | -7.988255 | -1.129246 | -2.891842 |
| 1 | -10.137244 | -0.805273 | -1.668557 |
| 1 | -1.686285 | -2.057989 | 1.343496 |
| 1 | -4.308745 | -5.305455 | 0.302792 |
| 1 | 0.023269 | -3.655597 | 2.081399 |
| 1 | -2.627027 | -6.912119 | 1.114353 |
| 1 | -0.437753 | -6.106485 | 1.996331 |
| 1 | -7.306650 | -1.921641 | 0.460233 |
| 1 | -4.003160 | -2.726470 | 3.109396 |
| 1 | -8.815638 | -1.769983 | 2.390337 |
| 1 | -5.520975 | -2.570042 | 5.040652 |
| 1 | -7.941064 | -2.088859 | 4.702018 |
| 1 | -3.998461 | -1.998594 | -2.588370 |
| 1 | -6.579275 | -4.324417 | -0.054184 |
| 1 | -5.003073 | -3.062592 | -4.590343 |
| 1 | -7.597059 | -5.369614 | -2.045612 |
| 1 | -6.818837 | -4.746457 | -4.323457 |
| 1 | -1.107383 | 1.112779 | 0.117329 |
| 1 | 0.685529 | 1.082517 | -1.704092 |
| 6 | 0.691873 | 3.185396 | -1.845139 |
| 6 | -0.437164 | 3.898626 | -1.052591 |
| 1 | 0.887882 | 3.768024 | -2.741547 |
| 1 | 1.629500 | 3.034681 | -1.312744 |
| 7 | 0.156956 | 1.839927 | -2.258769 |
| 16 | 0.441788 | 1.431670 | -4.025461 |
| 8 | -0.262552 | 0.162929 | -4.197221 |
| 8 | 0.013735 | 2.624984 | -4.758155 |
| 6 | -1.692311 | 3.280286 | -1.723964 |
| 1 | -1.840227 | 3.741652 | -2.705307 |
| 1 | -2.601386 | 3.414427 | -1.141635 |
| 6 | -0.460666 | 3.610573 | 0.458643 |
| 6 | -1.495900 | 4.178737 | 1.216264 |

| | | | |
|---|---|---|---|
| 6 | 0.448671 | 2.768325 | 1.101161 |
| 6 | -1.650834 | 3.872115 | 2.566200 |
| 1 | -2.192968 | 4.856606 | 0.737380 |
| 6 | 0.291138 | 2.451760 | 2.452410 |
| 1 | 1.266178 | 2.311465 | 0.561141 |
| 6 | -0.760379 | 2.994167 | 3.189338 |
| 1 | -2.470972 | 4.309091 | 3.129433 |
| 1 | 0.988871 | 1.765141 | 2.913982 |
| 1 | -0.885555 | 2.733007 | 4.235603 |
| 6 | -0.291612 | 5.411089 | -1.252233 |
| 6 | -1.342983 | 6.218541 | -1.698503 |
| 6 | 0.937392 | 6.016652 | -0.945321 |
| 6 | -1.169367 | 7.598590 | -1.842224 |
| 1 | -2.308841 | 5.782328 | -1.924906 |
| 6 | 1.114706 | 7.390077 | -1.100007 |
| 1 | 1.753507 | 5.410218 | -0.565008 |
| 6 | 0.059366 | 8.189373 | -1.549482 |
| 1 | -2.000454 | 8.208894 | -2.185571 |
| 1 | 2.075338 | 7.840021 | -0.863452 |
| 1 | 0.194375 | 9.261112 | -1.664524 |
| 6 | 2.199972 | 1.156793 | -4.112526 |
| 6 | 3.156400 | 2.205880 | -4.335141 |
| 6 | 2.572322 | -0.160470 | -3.904152 |
| 6 | 2.863590 | 3.579899 | -4.557606 |
| 6 | 4.538102 | 1.806282 | -4.327998 |
| 6 | 3.930601 | -0.524266 | -3.939822 |
| 1 | 1.822926 | -0.917836 | -3.713096 |
| 6 | 3.875491 | 4.505342 | -4.697970 |
| 1 | 1.836511 | 3.907006 | -4.635992 |
| 6 | 5.552504 | 2.789214 | -4.483736 |
| 6 | 4.885550 | 0.441618 | -4.150343 |
| 1 | 4.203651 | -1.560234 | -3.775271 |
| 6 | 5.233270 | 4.116546 | -4.649665 |
| 1 | 3.619848 | 5.549059 | -4.855122 |
| 1 | 6.589632 | 2.466148 | -4.461270 |
| 1 | 5.938032 | 0.174567 | -4.162683 |
| 1 | 6.015647 | 4.861622 | -4.757056 |
| 6 | 3.438223 | 0.133740 | 2.452628 |
| 6 | 4.195024 | 1.189363 | 2.961965 |
| 6 | 4.980651 | 1.005456 | 4.135252 |
| 6 | 5.771699 | 2.042275 | 4.706734 |
| 1 | 5.779053 | 3.018738 | 4.235745 |
| 6 | 6.509660 | 1.816513 | 5.847144 |
| 1 | 7.102158 | 2.621255 | 6.273431 |
| 6 | 6.507638 | 0.543464 | 6.471385 |
| 1 | 7.094524 | 0.382403 | 7.371078 |
| 6 | 5.769642 | -0.484231 | 5.931669 |
| 1 | 5.770720 | -1.468141 | 6.394093 |
| 6 | 4.993108 | -0.283210 | 4.756459 |
| 6 | 4.290439 | -1.343263 | 4.143724 |
| 1 | 4.416729 | -2.335233 | 4.564352 |
| 6 | 3.511950 | -1.194952 | 3.000500 |
| 6 | 2.986458 | -2.461391 | 2.392161 |
| 6 | 2.645350 | -3.541745 | 3.188783 |
| 1 | 2.517224 | -3.401392 | 4.257696 |
| 6 | 2.487826 | -4.848394 | 2.659529 |
| 6 | 2.127422 | -5.947287 | 3.486334 |
| 1 | 1.908879 | -5.760035 | 4.534535 |
| 6 | 2.062690 | -7.225480 | 2.975870 |
| 1 | 1.785772 | -8.056496 | 3.618138 |
| 6 | 2.368924 | -7.460682 | 1.613089 |
| 1 | 2.331387 | -8.472743 | 1.220332 |

| | | | |
|---|---|---|---|
| 6 | 2.714771 | -6.415686 | 0.783222 |
| 1 | 2.950705 | -6.597806 | -0.259349 |
| 6 | 2.757873 | -5.082309 | 1.270618 |
| 6 | 3.020279 | -3.962048 | 0.423829 |
| 6 | 3.030430 | -2.697420 | 0.980948 |
| 6 | 4.250779 | 2.466598 | 2.174615 |
| 6 | 3.548096 | 3.634177 | 2.617840 |
| 6 | 2.862808 | 3.688548 | 3.863508 |
| 1 | 2.871127 | 2.814542 | 4.504821 |
| 6 | 2.183959 | 4.820659 | 4.254899 |
| 1 | 1.662348 | 4.833844 | 5.207555 |
| 6 | 2.139968 | 5.959911 | 3.416252 |
| 1 | 1.582981 | 6.839252 | 3.726471 |
| 6 | 2.801173 | 5.948077 | 2.209554 |
| 1 | 2.773028 | 6.817147 | 1.557552 |
| 6 | 3.529299 | 4.803207 | 1.786758 |
| 6 | 4.233041 | 4.778305 | 0.554702 |
| 1 | 4.217950 | 5.661491 | -0.079622 |
| 6 | 4.943477 | 3.663819 | 0.178325 |
| 1 | 5.490188 | 3.669737 | -0.760558 |
| 6 | 4.975513 | 2.492793 | 0.983385 |
| 6 | 3.443592 | -4.113509 | -1.007161 |
| 6 | 4.863784 | -4.073987 | -1.256113 |
| 6 | 5.825177 | -3.990838 | -0.208811 |
| 1 | 5.485954 | -3.957429 | 0.819644 |
| 6 | 7.176530 | -3.952343 | -0.477744 |
| 1 | 7.886505 | -3.888683 | 0.342011 |
| 6 | 7.647935 | -3.984231 | -1.810970 |
| 1 | 8.715054 | -3.945753 | -2.010261 |
| 6 | 6.748498 | -4.073161 | -2.849649 |
| 1 | 7.095239 | -4.108739 | -3.879662 |
| 6 | 5.350920 | -4.132595 | -2.601978 |
| 6 | 4.412015 | -4.254140 | -3.659264 |
| 1 | 4.772522 | -4.299847 | -4.684217 |
| 6 | 3.069561 | -4.328665 | -3.390170 |
| 1 | 2.373559 | -4.440574 | -4.215262 |
| 6 | 2.552261 | -4.262755 | -2.062160 |
| 8 | 1.812900 | 0.278966 | -0.971489 |
| 8 | 0.704814 | -1.133774 | 0.961979 |
| 8 | 2.656891 | 0.494555 | 1.383876 |
| 8 | 3.120523 | -1.617620 | 0.124248 |
| 15 | 1.900577 | -0.505354 | 0.314402 |
| 6 | 1.049655 | -4.409033 | -1.877896 |
| 6 | 0.605911 | -5.832679 | -2.304535 |
| 6 | 0.253911 | -3.333839 | -2.656337 |
| 1 | 0.808971 | -4.287610 | -0.815806 |
| 6 | -0.910983 | -6.030897 | -2.206843 |
| 1 | 0.922161 | -6.012681 | -3.341382 |
| 1 | 1.126036 | -6.573014 | -1.686586 |
| 6 | -1.262708 | -3.563445 | -2.580292 |
| 1 | 0.555948 | -3.343502 | -3.712316 |
| 1 | 0.504564 | -2.341749 | -2.263652 |
| 6 | -1.660677 | -4.974896 | -3.025839 |
| 1 | -1.175559 | -7.041785 | -2.542939 |
| 1 | -1.218378 | -5.953515 | -1.158584 |
| 1 | -1.775422 | -2.818305 | -3.198188 |
| 1 | -1.602558 | -3.405962 | -1.551398 |
| 1 | -2.744606 | -5.104924 | -2.923098 |
| 1 | -1.423707 | -5.104091 | -4.092694 |
| 6 | 5.835615 | 1.322941 | 0.546306 |
| 6 | 7.340775 | 1.672677 | 0.606398 |
| 6 | 5.479581 | 0.757921 | -0.843090 |

| | | | |
|---|---|---|---|
| 1 | 5.682952 | 0.508394 | 1.258781 |
| 6 | 8.203944 | 0.441188 | 0.292388 |
| 1 | 7.558556 | 2.472145 | -0.116196 |
| 1 | 7.589924 | 2.068054 | 1.598332 |
| 6 | 6.317431 | -0.496641 | -1.116795 |
| 1 | 5.682477 | 1.513965 | -1.613497 |
| 1 | 4.412885 | 0.529922 | -0.900507 |
| 6 | 7.820057 | -0.193476 | -1.052777 |
| 1 | 9.267287 | 0.712174 | 0.301796 |
| 1 | 8.061571 | -0.302240 | 1.089627 |
| 1 | 6.057981 | -0.936228 | -2.083496 |
| 1 | 6.065597 | -1.256574 | -0.369079 |
| 1 | 8.398395 | -1.109956 | -1.220294 |
| 1 | 8.084167 | 0.500433 | -1.865722 |
| 1 | -0.592603 | -0.468353 | 2.091311 |
| 8 | -1.362406 | -0.214267 | 2.640759 |
| 1 | -1.607850 | 0.657018 | 2.306966 |

---

(*S*,*S*,*S*)-**3a**exo-*si*

---

Number of imaginary frequencies : 0
The smallest frequencies are : 10.3282 13.4759 16.1041 cm(-1)

Electronic energy : HF=-6614.0053512
Zero-point correction= 1.890573 (Hartree/Particle)
Thermal correction to Energy= 2.001229
Thermal correction to Enthalpy= 2.002174
Thermal correction to Gibbs Free Energy= 1.738714
Sum of electronic and zero-point Energies= -6612.114779
Sum of electronic and thermal Energies= -6612.004122
Sum of electronic and thermal Enthalpies= -6612.003178
Sum of electronic and thermal Free Energies= -6612.266637

........................................................

Cartesian Coordinates

........................................................

| | | | |
|---|---|---|---|
| 6 | 6.880079 | 0.800262 | -1.317229 |
| 6 | 6.760099 | -0.112151 | -0.256461 |
| 6 | 7.862089 | -0.311744 | 0.587840 |
| 6 | 9.049018 | 0.393055 | 0.378954 |
| 6 | 9.155339 | 1.302146 | -0.677076 |
| 6 | 8.068602 | 1.499824 | -1.530902 |
| 15 | 5.104437 | -0.914145 | -0.016433 |
| 6 | 5.091337 | -1.454245 | 1.746906 |
| 6 | 5.896412 | -2.495614 | 2.243986 |
| 6 | 5.837440 | -2.855931 | 3.589634 |
| 6 | 4.973486 | -2.183412 | 4.459209 |
| 6 | 4.171525 | -1.149341 | 3.977437 |
| 6 | 4.230305 | -0.788072 | 2.630239 |
| 46 | 3.364050 | 0.559632 | -0.712720 |
| 6 | 1.592609 | 0.997652 | -1.884929 |
| 6 | 1.884156 | -0.339506 | -2.194287 |
| 6 | 1.156254 | -1.489869 | -1.574125 |
| 15 | 3.847676 | 2.688687 | 0.272089 |
| 6 | 4.681027 | 2.625429 | 1.920488 |
| 6 | 6.038655 | 2.274167 | 2.012843 |
| 6 | 6.635185 | 2.062098 | 3.255146 |
| 6 | 5.885739 | 2.186893 | 4.426686 |
| 6 | 4.536334 | 2.535380 | 4.345308 |
| 6 | 3.937047 | 2.755908 | 3.103775 |
| 6 | 5.329735 | -2.499905 | -0.952635 |
| 6 | 4.804876 | -3.714822 | -0.482731 |

| | | | |
|---|---|---|---|
| 6 | 4.900420 | -4.876640 | -1.252207 |
| 6 | 5.505400 | -4.843768 | -2.509423 |
| 6 | 6.014843 | -3.635798 | -2.993823 |
| 6 | 5.930835 | -2.476552 | -2.222524 |
| 6 | 2.470972 | 3.890381 | 0.572644 |
| 6 | 2.623478 | 5.279999 | 0.436385 |
| 6 | 1.545486 | 6.133298 | 0.678211 |
| 6 | 0.303613 | 5.615332 | 1.060880 |
| 6 | 0.144515 | 4.235671 | 1.200105 |
| 6 | 1.221956 | 3.382227 | 0.954524 |
| 6 | 4.989301 | 3.659592 | -0.806362 |
| 6 | 4.830470 | 3.518810 | -2.195195 |
| 6 | 5.639357 | 4.230676 | -3.080743 |
| 6 | 6.626507 | 5.089694 | -2.590076 |
| 6 | 6.789378 | 5.240681 | -1.211439 |
| 6 | 5.972848 | 4.535813 | -0.324217 |
| 1 | 1.057111 | -1.345789 | -0.498628 |
| 1 | 1.825748 | 1.760150 | -2.620854 |
| 1 | 0.776460 | 1.245531 | -1.213202 |
| 1 | 4.305537 | -3.759506 | 0.478355 |
| 1 | 6.342990 | -1.550845 | -2.612130 |
| 1 | 4.490059 | -5.805934 | -0.868370 |
| 1 | 6.487453 | -3.596081 | -3.971470 |
| 1 | 5.577506 | -5.748386 | -3.106647 |
| 1 | 6.558829 | -3.037393 | 1.576108 |
| 1 | 3.602679 | 0.017127 | 2.271849 |
| 1 | 6.462972 | -3.664554 | 3.957899 |
| 1 | 3.491239 | -0.623531 | 4.639948 |
| 1 | 4.924144 | -2.469946 | 5.506208 |
| 1 | 7.789535 | -0.989047 | 1.431104 |
| 1 | 6.024143 | 0.992077 | -1.956600 |
| 1 | 9.889395 | 0.237815 | 1.050239 |
| 1 | 8.128346 | 2.218704 | -2.342622 |
| 1 | 10.076719 | 1.857736 | -0.827844 |
| 1 | 3.576218 | 5.699641 | 0.131212 |
| 1 | 1.080643 | 2.317820 | 1.060665 |
| 1 | 1.674101 | 7.205743 | 0.558995 |
| 1 | -0.814281 | 3.807054 | 1.472167 |
| 1 | -0.535988 | 6.282350 | 1.235408 |
| 1 | 6.634488 | 2.163924 | 1.115178 |
| 1 | 2.885609 | 3.020125 | 3.061206 |
| 1 | 7.685236 | 1.787816 | 3.301535 |
| 1 | 3.942953 | 2.636670 | 5.249891 |
| 1 | 6.348466 | 2.011168 | 5.393697 |
| 1 | 4.075467 | 2.838070 | -2.578000 |
| 1 | 6.109298 | 4.665256 | 0.744287 |
| 1 | 5.502807 | 4.109438 | -4.151778 |
| 1 | 7.552087 | 5.909789 | -0.822851 |
| 1 | 7.263257 | 5.638642 | -3.278235 |
| 1 | 2.378098 | -0.566113 | -3.137523 |
| 1 | -1.032905 | -1.163144 | -1.282402 |
| 6 | -0.682747 | -3.152135 | -1.766987 |
| 6 | 0.638550 | -3.864113 | -1.362130 |
| 1 | -1.033401 | -3.574396 | -2.703176 |
| 1 | -1.487445 | -3.223742 | -1.038919 |
| 7 | -0.383086 | -1.684047 | -1.963404 |
| 16 | -0.862704 | -1.092015 | -3.627596 |
| 8 | -0.845559 | 0.361515 | -3.537810 |
| 8 | 0.015541 | -1.810958 | -4.551639 |
| 6 | 1.694504 | -2.869832 | -1.906199 |
| 1 | 1.782930 | -2.955692 | -2.993850 |
| 1 | 2.676210 | -3.021499 | -1.463776 |

| | | | |
|---|---|---|---|
| 6 | 0.836287 | -3.984940 | 0.159967 |
| 6 | 1.754272 | -4.922886 | 0.661727 |
| 6 | 0.250231 | -3.087878 | 1.062471 |
| 6 | 2.101993 | -4.935088 | 2.012171 |
| 1 | 2.210208 | -5.639319 | -0.013360 |
| 6 | 0.613850 | -3.084685 | 2.410174 |
| 1 | -0.485325 | -2.359806 | 0.741564 |
| 6 | 1.544063 | -4.002274 | 2.890804 |
| 1 | 2.820434 | -5.666098 | 2.374127 |
| 1 | 0.167371 | -2.351489 | 3.069585 |
| 1 | 1.828096 | -3.990913 | 3.938664 |
| 6 | 0.665418 | -5.239857 | -2.030388 |
| 6 | 1.726817 | -5.668578 | -2.834898 |
| 6 | -0.418353 | -6.110741 | -1.832074 |
| 6 | 1.699007 | -6.929661 | -3.438891 |
| 1 | 2.588950 | -5.029664 | -2.988970 |
| 6 | -0.453128 | -7.363285 | -2.441513 |
| 1 | -1.240284 | -5.803340 | -1.190759 |
| 6 | 0.608445 | -7.778158 | -3.251258 |
| 1 | 2.534769 | -7.243405 | -4.058606 |
| 1 | -1.306206 | -8.016701 | -2.281214 |
| 1 | 0.586010 | -8.755277 | -3.725494 |
| 6 | -2.567493 | -1.643929 | -3.706237 |
| 6 | -2.987672 | -2.882086 | -4.304826 |
| 6 | -3.452249 | -0.787436 | -3.072640 |
| 6 | -2.143286 | -3.828348 | -4.950985 |
| 6 | -4.392115 | -3.184273 | -4.213402 |
| 6 | -4.818743 | -1.117966 | -2.991792 |
| 1 | -3.107758 | 0.136891 | -2.627942 |
| 6 | -2.654593 | -5.007704 | -5.449286 |
| 1 | -1.086312 | -3.625756 | -5.052995 |
| 6 | -4.881380 | -4.403181 | -4.755234 |
| 6 | -5.273729 | -2.283358 | -3.559253 |
| 1 | -5.491973 | -0.451041 | -2.470278 |
| 6 | -4.032977 | -5.303881 | -5.355557 |
| 1 | -1.981794 | -5.717693 | -5.920744 |
| 1 | -5.944541 | -4.612471 | -4.673624 |
| 1 | -6.327371 | -2.541414 | -3.500180 |
| 1 | -4.416278 | -6.237473 | -5.756031 |
| 6 | -3.309484 | 0.403922 | 2.880350 |
| 6 | -3.571320 | -0.569009 | 3.832941 |
| 6 | -4.625742 | -0.357547 | 4.785014 |
| 6 | -4.975982 | -1.324570 | 5.769924 |
| 1 | -4.428072 | -2.258054 | 5.813588 |
| 6 | -6.001907 | -1.090683 | 6.659356 |
| 1 | -6.252190 | -1.845328 | 7.399652 |
| 6 | -6.734681 | 0.120393 | 6.619257 |
| 1 | -7.539121 | 0.289979 | 7.329100 |
| 6 | -6.425648 | 1.073901 | 5.677047 |
| 1 | -6.981614 | 2.006906 | 5.629053 |
| 6 | -5.373427 | 0.860295 | 4.744982 |
| 6 | -5.049921 | 1.824873 | 3.765323 |
| 1 | -5.638618 | 2.736090 | 3.723835 |
| 6 | -4.035679 | 1.638274 | 2.841763 |
| 6 | -3.812502 | 2.731642 | 1.857605 |
| 6 | -3.861845 | 4.052044 | 2.266601 |
| 1 | -3.847093 | 4.282760 | 3.327638 |
| 6 | -4.003246 | 5.111764 | 1.338820 |
| 6 | -4.091902 | 6.465063 | 1.766979 |
| 1 | -3.967375 | 6.684575 | 2.824368 |
| 6 | -4.350777 | 7.474093 | 0.866971 |
| 1 | -4.418644 | 8.504551 | 1.203597 |

| | | | |
|---|---|---|---|
| 6 | -4.558848 | 7.163399 | -0.498420 |
| 1 | -4.801664 | 7.956487 | -1.199867 |
| 6 | -4.462140 | 5.863115 | -0.944233 |
| 1 | -4.637323 | 5.631909 | -1.987755 |
| 6 | -4.139813 | 4.803757 | -0.054319 |
| 6 | -3.948565 | 3.452545 | -0.498614 |
| 6 | -3.748571 | 2.470838 | 0.456510 |
| 6 | -2.763000 | -1.825094 | 3.969224 |
| 6 | -1.811736 | -1.890238 | 5.042782 |
| 6 | -1.440188 | -0.744561 | 5.802667 |
| 1 | -1.889619 | 0.213006 | 5.564054 |
| 6 | -0.508377 | -0.832319 | 6.812978 |
| 1 | -0.232450 | 0.058504 | 7.370332 |
| 6 | 0.098790 | -2.072383 | 7.128735 |
| 1 | 0.832972 | -2.126796 | 7.927760 |
| 6 | -0.242196 | -3.202118 | 6.420457 |
| 1 | 0.218063 | -4.160223 | 6.649989 |
| 6 | -1.195114 | -3.142801 | 5.367485 |
| 6 | -1.547493 | -4.287251 | 4.610966 |
| 1 | -1.094108 | -5.243692 | 4.857648 |
| 6 | -2.410185 | -4.182998 | 3.547970 |
| 1 | -2.623384 | -5.068186 | 2.960030 |
| 6 | -3.021006 | -2.950292 | 3.188379 |
| 6 | -4.075290 | 3.099157 | -1.949925 |
| 6 | -5.274002 | 2.415923 | -2.370074 |
| 6 | -6.308554 | 2.043573 | -1.464539 |
| 1 | -6.194741 | 2.264696 | -0.410056 |
| 6 | -7.456691 | 1.417933 | -1.903296 |
| 1 | -8.227859 | 1.148088 | -1.187314 |
| 6 | -7.640509 | 1.126879 | -3.276088 |
| 1 | -8.549961 | 0.635465 | -3.609913 |
| 6 | -6.659253 | 1.465826 | -4.180131 |
| 1 | -6.779525 | 1.240787 | -5.236838 |
| 6 | -5.465272 | 2.109369 | -3.757267 |
| 6 | -4.443914 | 2.454227 | -4.679075 |
| 1 | -4.571425 | 2.197931 | -5.727833 |
| 6 | -3.318142 | 3.109914 | -4.254783 |
| 1 | -2.553173 | 3.370035 | -4.978921 |
| 6 | -3.115754 | 3.471033 | -2.890597 |
| 8 | -2.200287 | -0.922021 | -0.275312 |
| 8 | -0.969198 | 1.327795 | 0.171950 |
| 8 | -2.237887 | 0.193856 | 2.045668 |
| 8 | -3.580749 | 1.154328 | 0.054545 |
| 15 | -2.142400 | 0.425776 | 0.415287 |
| 6 | -1.869249 | 4.276217 | -2.552743 |
| 6 | -1.887473 | 5.662248 | -3.246159 |
| 6 | -0.569179 | 3.526974 | -2.918501 |
| 1 | -1.844514 | 4.446155 | -1.471586 |
| 6 | -0.652948 | 6.492487 | -2.872039 |
| 1 | -1.907647 | 5.520717 | -4.335717 |
| 1 | -2.798253 | 6.207883 | -2.985174 |
| 6 | 0.674900 | 4.364579 | -2.589839 |
| 1 | -0.570855 | 3.280731 | -3.988436 |
| 1 | -0.546646 | 2.577989 | -2.381746 |
| 6 | 0.638202 | 5.752662 | -3.240999 |
| 1 | -0.694295 | 7.470221 | -3.368962 |
| 1 | -0.664491 | 6.684325 | -1.790277 |
| 1 | 1.580063 | 3.832233 | -2.903229 |
| 1 | 0.745637 | 4.482634 | -1.505892 |
| 1 | 1.516303 | 6.334692 | -2.934551 |
| 1 | 0.692509 | 5.649499 | -4.335179 |
| 6 | -3.914840 | -2.864077 | 1.960059 |

| | | | |
|---|---|---|---|
| 6 | -5.427010 | -2.927894 | 2.268660 |
| 6 | -3.574449 | -3.903252 | 0.874312 |
| 1 | -3.723775 | -1.887843 | 1.506112 |
| 6 | -6.239232 | -2.641759 | 0.994791 |
| 1 | -5.673997 | -3.926045 | 2.658243 |
| 1 | -5.687647 | -2.210137 | 3.051355 |
| 6 | -4.358759 | -3.611013 | -0.410396 |
| 1 | -3.828697 | -4.913652 | 1.224403 |
| 1 | -2.495699 | -3.896495 | 0.686125 |
| 6 | -5.871450 | -3.606341 | -0.143482 |
| 1 | -7.313743 | -2.698069 | 1.209464 |
| 1 | -6.033609 | -1.610709 | 0.672742 |
| 1 | -4.114666 | -4.349489 | -1.184735 |
| 1 | -4.048194 | -2.632401 | -0.791175 |
| 1 | -6.416045 | -3.339058 | -1.057458 |
| 1 | -6.193142 | -4.622412 | 0.128656 |
| 1 | 0.398536 | 0.485860 | 1.134104 |
| 8 | 1.122266 | 0.003746 | 1.579771 |
| 1 | 1.874963 | 0.094090 | 0.967127 |

---

(*S*,*S*,*S*)-**3a**exo-*re*

---

Number of imaginary frequencies : 0
The smallest frequencies are : 8.2173   15.0847   15.6970  cm(-1)

Electronic energy :        HF=-6614.0058322
Zero-point correction=                           1.889224 (Hartree/Particle)
Thermal correction to Energy=                    2.000404
Thermal correction to Enthalpy=                  2.001348
Thermal correction to Gibbs Free Energy=         1.736687
Sum of electronic and zero-point Energies=          -6612.116608
Sum of electronic and thermal Energies=             -6612.005429
Sum of electronic and thermal Enthalpies=           -6612.004484
Sum of electronic and thermal Free Energies=        -6612.269145

.......................................................

Cartesian Coordinates

.......................................................

| | | | |
|---|---|---|---|
| 6 | 3.422540 | -0.249393 | 2.627452 |
| 6 | 4.683375 | 0.157949 | 2.160979 |
| 6 | 5.783444 | 0.073393 | 3.026408 |
| 6 | 5.620842 | -0.406049 | 4.327611 |
| 6 | 4.361337 | -0.810166 | 4.779250 |
| 6 | 3.257315 | -0.732336 | 3.926796 |
| 15 | 4.759685 | 0.760258 | 0.416238 |
| 6 | 6.521079 | 0.547456 | -0.124690 |
| 6 | 7.576001 | 1.303167 | 0.416791 |
| 6 | 8.884646 | 1.116512 | -0.026882 |
| 6 | 9.158858 | 0.186171 | -1.033565 |
| 6 | 8.116765 | -0.549623 | -1.597200 |
| 6 | 6.807785 | -0.368260 | -1.145918 |
| 46 | 3.201630 | -0.476775 | -0.878507 |
| 6 | 1.508079 | -0.743065 | -2.248978 |
| 6 | 1.704278 | 0.641358 | -2.081717 |
| 6 | 0.802759 | 1.434041 | -1.192096 |
| 15 | 3.811531 | -2.746742 | -0.456876 |
| 6 | 5.380880 | -3.183565 | 0.430788 |
| 6 | 5.432932 | -3.090813 | 1.831753 |
| 6 | 6.621368 | -3.340932 | 2.516918 |
| 6 | 7.782345 | -3.678016 | 1.817866 |
| 6 | 7.742851 | -3.770750 | 0.426172 |
| 6 | 6.552736 | -3.527917 | -0.261452 |

| | | | |
|---|---|---|---|
| 6 | 4.778527 | 2.604476 | 0.634290 |
| 6 | 4.893196 | 3.393207 | -0.524039 |
| 6 | 5.010676 | 4.779299 | -0.436378 |
| 6 | 5.017319 | 5.403390 | 0.815912 |
| 6 | 4.881463 | 4.632242 | 1.970957 |
| 6 | 4.755113 | 3.241463 | 1.882839 |
| 6 | 4.067245 | -3.677077 | -2.040867 |
| 6 | 4.113227 | -5.076945 | -2.128653 |
| 6 | 4.404443 | -5.705485 | -3.339494 |
| 6 | 4.673842 | -4.944616 | -4.481305 |
| 6 | 4.636047 | -3.551543 | -4.406305 |
| 6 | 4.328527 | -2.924325 | -3.196125 |
| 6 | 2.571936 | -3.677007 | 0.549407 |
| 6 | 1.367599 | -3.040111 | 0.881172 |
| 6 | 0.431608 | -3.674680 | 1.700934 |
| 6 | 0.671348 | -4.963551 | 2.172584 |
| 6 | 1.868836 | -5.610383 | 1.849322 |
| 6 | 2.820433 | -4.964237 | 1.061893 |
| 1 | 0.409450 | 0.783146 | -0.410706 |
| 1 | 0.680815 | -1.244861 | -1.752349 |
| 1 | 1.848371 | -1.240753 | -3.152253 |
| 1 | 4.902979 | 2.913593 | -1.500176 |
| 1 | 4.659448 | 2.655571 | 2.790745 |
| 1 | 5.090425 | 5.372209 | -1.342931 |
| 1 | 4.873236 | 5.108400 | 2.947605 |
| 1 | 5.109588 | 6.483269 | 0.886392 |
| 1 | 7.372902 | 2.051649 | 1.176484 |
| 1 | 5.998355 | -0.937504 | -1.589513 |
| 1 | 9.689274 | 1.704038 | 0.406765 |
| 1 | 8.317998 | -1.267785 | -2.387241 |
| 1 | 10.178454 | 0.044828 | -1.381420 |
| 1 | 6.772942 | 0.354535 | 2.684253 |
| 1 | 2.562439 | -0.203612 | 1.965937 |
| 1 | 6.483560 | -0.473903 | 4.985064 |
| 1 | 2.265768 | -1.039465 | 4.245455 |
| 1 | 4.242424 | -1.189071 | 5.790806 |
| 1 | 3.916730 | -5.685319 | -1.253701 |
| 1 | 4.282731 | -1.839793 | -3.146577 |
| 1 | 4.424050 | -6.790531 | -3.390767 |
| 1 | 4.835068 | -2.950419 | -5.289216 |
| 1 | 4.903987 | -5.436418 | -5.422208 |
| 1 | 4.544865 | -2.821794 | 2.393194 |
| 1 | 6.541930 | -3.611251 | -1.342875 |
| 1 | 6.635965 | -3.258793 | 3.599582 |
| 1 | 8.639079 | -4.031124 | -0.130458 |
| 1 | 8.708577 | -3.867416 | 2.353458 |
| 1 | 1.155265 | -2.040884 | 0.516871 |
| 1 | 3.772767 | -5.450351 | 0.876009 |
| 1 | -0.471330 | -3.145139 | 1.970637 |
| 1 | 2.071414 | -6.608418 | 2.228445 |
| 1 | -0.064094 | -5.458513 | 2.801175 |
| 1 | 2.234121 | 1.200975 | -2.845571 |
| 1 | -1.288298 | 1.167560 | -1.521843 |
| 6 | -0.888388 | 3.208933 | -1.145061 |
| 6 | 0.458556 | 3.916640 | -0.895013 |
| 1 | -1.349658 | 2.916881 | -0.197153 |
| 1 | -1.608476 | 3.797260 | -1.705725 |
| 7 | -0.579265 | 1.922055 | -1.845578 |
| 16 | -0.756253 | 1.854885 | -3.694108 |
| 8 | 0.529031 | 2.209952 | -4.287238 |
| 8 | -1.354157 | 0.540885 | -3.906515 |
| 6 | 1.370402 | 2.711539 | -0.550665 |

| | | | |
|---|---|---|---|
| 1 | 1.371324 | 2.566882 | 0.529711 |
| 1 | 2.404746 | 2.867106 | -0.837399 |
| 6 | 0.909476 | 4.711516 | -2.143831 |
| 6 | 2.054332 | 4.400132 | -2.885523 |
| 6 | 0.153535 | 5.825289 | -2.546051 |
| 6 | 2.433986 | 5.173583 | -3.986148 |
| 1 | 2.660483 | 3.541752 | -2.627923 |
| 6 | 0.529297 | 6.602917 | -3.638696 |
| 1 | -0.738271 | 6.098793 | -1.991280 |
| 6 | 1.676801 | 6.279980 | -4.367633 |
| 1 | 3.324383 | 4.901513 | -4.546106 |
| 1 | -0.076661 | 7.459305 | -3.920840 |
| 1 | 1.975532 | 6.884440 | -5.219458 |
| 6 | 0.357980 | 4.902878 | 0.274188 |
| 6 | -0.843129 | 5.530269 | 0.630825 |
| 6 | 1.514298 | 5.217638 | 0.999725 |
| 6 | -0.884455 | 6.443570 | 1.688228 |
| 1 | -1.765042 | 5.304200 | 0.104403 |
| 6 | 1.472750 | 6.116018 | 2.063664 |
| 1 | 2.455053 | 4.751660 | 0.737197 |
| 6 | 0.270146 | 6.734906 | 2.414879 |
| 1 | -1.826424 | 6.918509 | 1.948388 |
| 1 | 2.383423 | 6.327883 | 2.616179 |
| 1 | 0.233982 | 7.436169 | 3.243586 |
| 6 | -1.931642 | 3.163125 | -4.063727 |
| 6 | -3.258072 | 3.191236 | -3.518714 |
| 6 | -1.453897 | 4.149105 | -4.903114 |
| 6 | -3.788023 | 2.226546 | -2.622981 |
| 6 | -4.057690 | 4.342363 | -3.841791 |
| 6 | -2.290528 | 5.224535 | -5.271046 |
| 1 | -0.431218 | 4.111943 | -5.257128 |
| 6 | -5.007788 | 2.435381 | -2.014462 |
| 1 | -3.246664 | 1.320185 | -2.390435 |
| 6 | -5.330935 | 4.495365 | -3.230451 |
| 6 | -3.554717 | 5.322630 | -4.739557 |
| 1 | -1.909123 | 5.984891 | -5.944079 |
| 6 | -5.788005 | 3.574176 | -2.315776 |
| 1 | -5.365831 | 1.714641 | -1.289168 |
| 1 | -5.926379 | 5.367856 | -3.485740 |
| 1 | -4.188418 | 6.170220 | -4.987376 |
| 1 | -6.745387 | 3.712658 | -1.823601 |
| 6 | -2.853596 | -3.118173 | 0.416955 |
| 6 | -3.242251 | -3.924040 | -0.638126 |
| 6 | -4.055664 | -5.068929 | -0.379529 |
| 6 | -4.452071 | -5.965297 | -1.409434 |
| 1 | -4.114654 | -5.777172 | -2.422793 |
| 6 | -5.240676 | -7.058915 | -1.125863 |
| 1 | -5.529794 | -7.737482 | -1.923426 |
| 6 | -5.678143 | -7.308583 | 0.198528 |
| 1 | -6.299201 | -8.175138 | 0.406664 |
| 6 | -5.317656 | -6.454676 | 1.216263 |
| 1 | -5.653812 | -6.635225 | 2.234286 |
| 6 | -4.503641 | -5.317598 | 0.957912 |
| 6 | -4.174267 | -4.390854 | 1.976072 |
| 1 | -4.611853 | -4.527654 | 2.960533 |
| 6 | -3.367499 | -3.288891 | 1.740386 |
| 6 | -3.190748 | -2.282304 | 2.820468 |
| 6 | -3.066775 | -2.661307 | 4.147243 |
| 1 | -2.918061 | -3.709180 | 4.392169 |
| 6 | -3.173538 | -1.722616 | 5.204759 |
| 6 | -3.068169 | -2.126269 | 6.564931 |
| 1 | -2.851225 | -3.169061 | 6.781957 |

| | | | |
|---|---|---|---|
| 6 | -3.247490 | -1.220548 | 7.584909 |
| 1 | -3.166107 | -1.539623 | 8.619917 |
| 6 | -3.555590 | 0.129378 | 7.286887 |
| 1 | -3.718820 | 0.834765 | 8.096612 |
| 6 | -3.651868 | 0.554503 | 5.980156 |
| 1 | -3.895406 | 1.586431 | 5.756620 |
| 6 | -3.439294 | -0.345476 | 4.901560 |
| 6 | -3.467900 | 0.072582 | 3.533165 |
| 6 | -3.318539 | -0.885040 | 2.546268 |
| 6 | -2.884247 | -3.506229 | -2.028390 |
| 6 | -1.578377 | -3.772629 | -2.545103 |
| 6 | -0.609676 | -4.506472 | -1.808503 |
| 1 | -0.860391 | -4.863418 | -0.815737 |
| 6 | 0.637616 | -4.754661 | -2.334425 |
| 1 | 1.365087 | -5.311172 | -1.754050 |
| 6 | 0.986775 | -4.273265 | -3.619194 |
| 1 | 1.975611 | -4.472171 | -4.015506 |
| 6 | 0.074924 | -3.548115 | -4.352650 |
| 1 | 0.336093 | -3.166062 | -5.336743 |
| 6 | -1.224052 | -3.282899 | -3.843115 |
| 6 | -2.185404 | -2.542672 | -4.578460 |
| 1 | -1.910206 | -2.146064 | -5.551941 |
| 6 | -3.438096 | -2.318223 | -4.064874 |
| 1 | -4.156493 | -1.742394 | -4.640761 |
| 6 | -3.817250 | -2.799705 | -2.781576 |
| 6 | -3.711310 | 1.496582 | 3.134755 |
| 6 | -4.970549 | 1.820176 | 2.512694 |
| 6 | -5.992891 | 0.854405 | 2.292515 |
| 1 | -5.838597 | -0.165488 | 2.624937 |
| 6 | -7.171376 | 1.187735 | 1.660792 |
| 1 | -7.929798 | 0.426973 | 1.501664 |
| 6 | -7.401971 | 2.511497 | 1.216114 |
| 1 | -8.334631 | 2.761020 | 0.717721 |
| 6 | -6.443389 | 3.477175 | 1.427953 |
| 1 | -6.607727 | 4.501270 | 1.101621 |
| 6 | -5.219686 | 3.162415 | 2.077913 |
| 6 | -4.225117 | 4.148393 | 2.305250 |
| 1 | -4.414588 | 5.170968 | 1.986555 |
| 6 | -3.043857 | 3.817330 | 2.916741 |
| 1 | -2.293715 | 4.583791 | 3.072311 |
| 6 | -2.748506 | 2.484630 | 3.326987 |
| 8 | -2.229559 | 0.124528 | -0.942366 |
| 8 | -0.765162 | -0.160714 | 1.188770 |
| 8 | -1.869489 | -2.188619 | 0.165202 |
| 8 | -3.370256 | -0.467011 | 1.227987 |
| 15 | -1.962072 | -0.565201 | 0.373824 |
| 6 | -1.370986 | 2.211962 | 3.909835 |
| 6 | -1.134542 | 2.960210 | 5.242497 |
| 6 | -0.256428 | 2.586219 | 2.904497 |
| 1 | -1.270262 | 1.140324 | 4.102977 |
| 6 | 0.258249 | 2.654233 | 5.814765 |
| 1 | -1.224992 | 4.042719 | 5.073385 |
| 1 | -1.905771 | 2.688757 | 5.970510 |
| 6 | 1.133442 | 2.280943 | 3.474696 |
| 1 | -0.320021 | 3.652368 | 2.661941 |
| 1 | -0.413195 | 2.022486 | 1.981567 |
| 6 | 1.366661 | 2.998521 | 4.809311 |
| 1 | 0.405539 | 3.203612 | 6.753609 |
| 1 | 0.314469 | 1.584823 | 6.059700 |
| 1 | 1.908200 | 2.568110 | 2.753329 |
| 1 | 1.219315 | 1.201464 | 3.627873 |
| 1 | 2.348740 | 2.727753 | 5.217646 |

| | | | |
|---|---|---|---|
| 1 | 1.378771 | 4.085277 | 4.639771 |
| 6 | -5.217657 | -2.514091 | -2.266368 |
| 6 | -6.308753 | -3.128176 | -3.170985 |
| 6 | -5.475853 | -1.008860 | -2.042705 |
| 1 | -5.323852 | -2.989134 | -1.286596 |
| 6 | -7.710459 | -2.889373 | -2.591317 |
| 1 | -6.249559 | -2.685485 | -4.174722 |
| 1 | -6.124212 | -4.202626 | -3.287421 |
| 6 | -6.877555 | -0.780393 | -1.458380 |
| 1 | -5.380960 | -0.469531 | -2.994660 |
| 1 | -4.706516 | -0.604426 | -1.377543 |
| 6 | -7.968524 | -1.395394 | -2.346572 |
| 1 | -8.472786 | -3.304995 | -3.262105 |
| 1 | -7.799878 | -3.432262 | -1.639390 |
| 1 | -7.063354 | 0.290482 | -1.317563 |
| 1 | -6.921773 | -1.233075 | -0.457864 |
| 1 | -8.958008 | -1.248406 | -1.895260 |
| 1 | -7.981826 | -0.869827 | -3.312773 |
| 1 | -0.235954 | -0.691944 | 2.819775 |
| 8 | 0.000728 | -0.991406 | 3.722282 |
| 1 | -0.774846 | -1.482961 | 4.017947 |

---

(*S*,*S*,*S*)-**3a**endo-*si*

---

Number of imaginary frequencies : 0
The smallest frequencies are : 8.9289 15.2325 18.5100 cm(-1)

Electronic energy : HF=-6614.0074376
Zero-point correction= 1.889962 (Hartree/Particle)
Thermal correction to Energy= 2.000838
Thermal correction to Enthalpy= 2.001782
Thermal correction to Gibbs Free Energy= 1.738684
Sum of electronic and zero-point Energies= -6612.117475
Sum of electronic and thermal Energies= -6612.006600
Sum of electronic and thermal Enthalpies= -6612.005655
Sum of electronic and thermal Free Energies= -6612.268754

Cartesian Coordinates

| | | | |
|---|---|---|---|
| 6 | -3.320463 | -0.143397 | 2.794568 |
| 6 | -4.566213 | -0.582696 | 2.314686 |
| 6 | -5.676388 | -0.523931 | 3.168374 |
| 6 | -5.536170 | -0.045634 | 4.472946 |
| 6 | -4.290921 | 0.382604 | 4.940074 |
| 6 | -3.177130 | 0.335146 | 4.097625 |
| 15 | -4.593281 | -1.204580 | 0.575184 |
| 6 | -6.365055 | -1.209185 | 0.028726 |
| 6 | -7.344051 | -2.011401 | 0.641321 |
| 6 | -8.658470 | -2.008043 | 0.176524 |
| 6 | -9.011552 | -1.224325 | -0.926178 |
| 6 | -8.041815 | -0.449569 | -1.562007 |
| 6 | -6.728999 | -0.441033 | -1.085140 |
| 46 | -3.201733 | 0.202244 | -0.721814 |
| 6 | -1.415793 | 0.777627 | -1.896679 |
| 6 | -1.103665 | -0.353705 | -1.129982 |
| 6 | -0.838060 | -1.709639 | -1.686449 |
| 15 | -4.198172 | 2.363467 | -0.436672 |
| 6 | -5.741810 | 2.606459 | 0.553473 |
| 6 | -5.672794 | 2.665326 | 1.955282 |
| 6 | -6.836113 | 2.750173 | 2.720728 |
| 6 | -8.088533 | 2.764461 | 2.104065 |

| | | | |
|---|---|---|---|
| 6 | -8.169379 | 2.693415 | 0.712139 |
| 6 | -7.007596 | 2.616912 | -0.056008 |
| 6 | -4.412931 | -3.038175 | 0.819374 |
| 6 | -4.524028 | -3.860003 | -0.316718 |
| 6 | -4.484789 | -5.248668 | -0.202397 |
| 6 | -4.332162 | -5.843128 | 1.055152 |
| 6 | -4.198544 | -5.036926 | 2.186245 |
| 6 | -4.232533 | -3.642831 | 2.070864 |
| 6 | -4.615318 | 3.244614 | -2.012427 |
| 6 | -5.237538 | 4.503170 | -2.069788 |
| 6 | -5.570292 | 5.077328 | -3.296791 |
| 6 | -5.303974 | 4.395057 | -4.488462 |
| 6 | -4.698092 | 3.138975 | -4.444680 |
| 6 | -4.354260 | 2.571497 | -3.215373 |
| 6 | -3.014426 | 3.429808 | 0.513818 |
| 6 | -1.850298 | 2.842287 | 1.029069 |
| 6 | -0.960146 | 3.575890 | 1.817151 |
| 6 | -1.213484 | 4.920749 | 2.080630 |
| 6 | -2.372644 | 5.521196 | 1.575773 |
| 6 | -3.273144 | 4.778116 | 0.813846 |
| 1 | -1.351096 | -1.886871 | -2.630394 |
| 1 | -1.601003 | 0.677322 | -2.965595 |
| 1 | -1.091996 | 1.765331 | -1.585037 |
| 1 | -4.662795 | -3.404761 | -1.294864 |
| 1 | -4.139577 | -3.031244 | 2.961727 |
| 1 | -4.568148 | -5.866029 | -1.092248 |
| 1 | -4.069478 | -5.488294 | 3.166078 |
| 1 | -4.301864 | -6.924851 | 1.146781 |
| 1 | -7.075091 | -2.657247 | 1.471705 |
| 1 | -5.975065 | 0.162802 | -1.580367 |
| 1 | -9.404676 | -2.627211 | 0.666869 |
| 1 | -8.303110 | 0.153259 | -2.427429 |
| 1 | -10.034609 | -1.228423 | -1.292006 |
| 1 | -6.657257 | -0.821064 | 2.816400 |
| 1 | -2.454390 | -0.169315 | 2.141611 |
| 1 | -6.407401 | 0.002331 | 5.120816 |
| 1 | -2.196546 | 0.667791 | 4.425062 |
| 1 | -4.190639 | 0.758190 | 5.954912 |
| 1 | -5.494618 | 5.027339 | -1.155703 |
| 1 | -3.873892 | 1.598176 | -3.181617 |
| 1 | -6.048547 | 6.052578 | -3.322668 |
| 1 | -4.485713 | 2.600663 | -5.364118 |
| 1 | -5.570993 | 4.841227 | -5.442421 |
| 1 | -4.710425 | 2.635285 | 2.455681 |
| 1 | -7.091038 | 2.567636 | -1.136307 |
| 1 | -6.757650 | 2.792446 | 3.802983 |
| 1 | -9.137837 | 2.695617 | 0.219338 |
| 1 | -8.993456 | 2.827416 | 2.702196 |
| 1 | -1.635599 | 1.799540 | 0.826172 |
| 1 | -4.182245 | 5.253127 | 0.462700 |
| 1 | -0.093140 | 3.076591 | 2.231928 |
| 1 | -2.582344 | 6.566262 | 1.787730 |
| 1 | -0.518223 | 5.498945 | 2.683294 |
| 1 | -0.640952 | -0.200623 | -0.158466 |
| 1 | 1.240555 | -1.026244 | -1.576625 |
| 6 | 1.195182 | -3.117550 | -1.353983 |
| 6 | -0.057311 | -3.969119 | -1.070260 |
| 1 | 1.664453 | -2.791140 | -0.423215 |
| 1 | 1.946008 | -3.615979 | -1.957013 |
| 7 | 0.711956 | -1.860129 | -2.026000 |
| 16 | 1.076120 | -1.640700 | -3.842468 |
| 8 | -0.156192 | -2.010427 | -4.539556 |

| | | | |
|---|---|---|---|
| 8 | 1.606344 | -0.283968 | -3.917571 |
| 6 | -1.046852 | -2.851199 | -0.692534 |
| 1 | -0.808505 | -2.497507 | 0.313790 |
| 1 | -2.089915 | -3.154724 | -0.679853 |
| 6 | -0.475448 | -4.768346 | -2.323732 |
| 6 | -1.749871 | -4.668634 | -2.895217 |
| 6 | 0.435388 | -5.666385 | -2.901807 |
| 6 | -2.098509 | -5.428332 | -4.014812 |
| 1 | -2.489147 | -3.997912 | -2.475166 |
| 6 | 0.091307 | -6.429362 | -4.015907 |
| 1 | 1.425640 | -5.782116 | -2.471594 |
| 6 | -1.180384 | -6.311054 | -4.582089 |
| 1 | -3.092332 | -5.324774 | -4.441378 |
| 1 | 0.818665 | -7.114844 | -4.441442 |
| 1 | -1.451606 | -6.902710 | -5.451759 |
| 6 | 0.189626 | -4.945849 | 0.081097 |
| 6 | 1.458194 | -5.482635 | 0.339749 |
| 6 | -0.880066 | -5.339081 | 0.894745 |
| 6 | 1.649326 | -6.391523 | 1.383358 |
| 1 | 2.314923 | -5.185335 | -0.256784 |
| 6 | -0.689548 | -6.236824 | 1.943825 |
| 1 | -1.869435 | -4.938938 | 0.717442 |
| 6 | 0.577511 | -6.768877 | 2.193835 |
| 1 | 2.640863 | -6.795948 | 1.567037 |
| 1 | -1.535537 | -6.513352 | 2.565956 |
| 1 | 0.728159 | -7.467910 | 3.011427 |
| 6 | 2.347504 | -2.844650 | -4.229001 |
| 6 | 3.637900 | -2.822681 | -3.604075 |
| 6 | 2.002714 | -3.772578 | -5.191690 |
| 6 | 4.017048 | -1.934572 | -2.564448 |
| 6 | 4.563989 | -3.843829 | -4.010916 |
| 6 | 2.951583 | -4.728442 | -5.613197 |
| 1 | 1.004659 | -3.773679 | -5.611726 |
| 6 | 5.228616 | -2.083876 | -1.923819 |
| 1 | 3.361200 | -1.137818 | -2.243149 |
| 6 | 5.823720 | -3.933337 | -3.359432 |
| 6 | 4.197788 | -4.762627 | -5.031046 |
| 1 | 2.677724 | -5.442671 | -6.382412 |
| 6 | 6.144273 | -3.083341 | -2.325405 |
| 1 | 5.476153 | -1.425514 | -1.098480 |
| 1 | 6.519850 | -4.702531 | -3.682857 |
| 1 | 4.922875 | -5.512704 | -5.335550 |
| 1 | 7.094513 | -3.174073 | -1.809384 |
| 6 | 2.402938 | 3.294894 | 0.523747 |
| 6 | 2.556457 | 4.202853 | -0.509902 |
| 6 | 3.218741 | 5.444439 | -0.264609 |
| 6 | 3.370545 | 6.435443 | -1.273578 |
| 1 | 2.955452 | 6.247055 | -2.257563 |
| 6 | 4.025671 | 7.617758 | -1.006703 |
| 1 | 4.128117 | 8.365995 | -1.787641 |
| 6 | 4.567073 | 7.868296 | 0.278562 |
| 1 | 5.084147 | 8.803631 | 0.473085 |
| 6 | 4.439738 | 6.927257 | 1.275016 |
| 1 | 4.855822 | 7.108322 | 2.263076 |
| 6 | 3.765316 | 5.698726 | 1.033703 |
| 6 | 3.669648 | 4.693449 | 2.024645 |
| 1 | 4.173967 | 4.853719 | 2.972983 |
| 6 | 3.010057 | 3.494768 | 1.804751 |
| 6 | 3.075742 | 2.444691 | 2.854277 |
| 6 | 2.985440 | 2.761639 | 4.198798 |
| 1 | 2.701825 | 3.767927 | 4.492931 |
| 6 | 3.281076 | 1.812843 | 5.209992 |

| | | | |
|---|---|---|---|
| 6 | 3.212871 | 2.156230 | 6.589206 |
| 1 | 2.879572 | 3.154898 | 6.859750 |
| 6 | 3.575965 | 1.251638 | 7.560051 |
| 1 | 3.524061 | 1.526027 | 8.609650 |
| 6 | 4.033741 | -0.037063 | 7.191174 |
| 1 | 4.340695 | -0.738817 | 7.961415 |
| 6 | 4.094085 | -0.407088 | 5.865536 |
| 1 | 4.448049 | -1.393218 | 5.588234 |
| 6 | 3.699166 | 0.491048 | 4.837186 |
| 6 | 3.699734 | 0.126431 | 3.453284 |
| 6 | 3.384350 | 1.092219 | 2.515318 |
| 6 | 2.130713 | 3.823411 | -1.892412 |
| 6 | 0.813813 | 4.137604 | -2.351728 |
| 6 | -0.114829 | 4.858087 | -1.551998 |
| 1 | 0.164175 | 5.141789 | -0.543427 |
| 6 | -1.354183 | 5.199879 | -2.045433 |
| 1 | -2.046791 | 5.753335 | -1.421854 |
| 6 | -1.742091 | 4.813268 | -3.349769 |
| 1 | -2.728790 | 5.075516 | -3.714899 |
| 6 | -0.874762 | 4.094426 | -4.141506 |
| 1 | -1.168432 | 3.785646 | -5.142104 |
| 6 | 0.422173 | 3.750866 | -3.675147 |
| 6 | 1.355253 | 3.053163 | -4.484959 |
| 1 | 1.053311 | 2.733418 | -5.479065 |
| 6 | 2.623104 | 2.794713 | -4.028154 |
| 1 | 3.324840 | 2.270322 | -4.669005 |
| 6 | 3.044079 | 3.187554 | -2.727877 |
| 6 | 4.073118 | -1.250797 | 2.994878 |
| 6 | 5.349621 | -1.432160 | 2.354957 |
| 6 | 6.262225 | -0.360805 | 2.140197 |
| 1 | 6.004857 | 0.631252 | 2.492999 |
| 6 | 7.459653 | -0.558815 | 1.487260 |
| 1 | 8.132131 | 0.279547 | 1.329967 |
| 6 | 7.822429 | -1.845473 | 1.021628 |
| 1 | 8.769868 | -1.989188 | 0.509704 |
| 6 | 6.973360 | -2.909536 | 1.228597 |
| 1 | 7.241618 | -3.905850 | 0.885463 |
| 6 | 5.728882 | -2.734392 | 1.891999 |
| 6 | 4.838558 | -3.818634 | 2.102811 |
| 1 | 5.127935 | -4.810776 | 1.763813 |
| 6 | 3.630893 | -3.618746 | 2.720782 |
| 1 | 2.960926 | -4.459127 | 2.862327 |
| 6 | 3.208431 | -2.330701 | 3.160118 |
| 8 | 2.168206 | -0.008030 | -0.891076 |
| 8 | 0.902861 | 0.033958 | 1.383927 |
| 8 | 1.568577 | 2.222865 | 0.297587 |
| 8 | 3.411121 | 0.736653 | 1.177949 |
| 15 | 1.927783 | 0.625305 | 0.458577 |
| 6 | 1.812749 | -2.195385 | 3.745582 |
| 6 | 1.662726 | -2.884362 | 5.121506 |
| 6 | 0.738021 | -2.750298 | 2.783330 |
| 1 | 1.595211 | -1.132715 | 3.880453 |
| 6 | 0.251276 | -2.669100 | 5.690807 |
| 1 | 1.852605 | -3.961613 | 5.008337 |
| 1 | 2.410345 | -2.501778 | 5.823287 |
| 6 | -0.670984 | -2.522780 | 3.342461 |
| 1 | 0.893422 | -3.823841 | 2.629766 |
| 1 | 0.842177 | -2.252572 | 1.818113 |
| 6 | -0.832245 | -3.169619 | 4.723996 |
| 1 | 0.159744 | -3.167718 | 6.664351 |
| 1 | 0.103146 | -1.594898 | 5.866564 |
| 1 | -1.419596 | -2.922437 | 2.648067 |

| | | | |
|---|---|---|---|
| 1 | -0.847233 | -1.445722 | 3.427671 |
| 1 | -1.829348 | -2.958065 | 5.130435 |
| 1 | -0.752630 | -4.262298 | 4.622965 |
| 6 | 4.490821 | 2.967570 | -2.317354 |
| 6 | 5.441145 | 3.862325 | -3.146853 |
| 6 | 4.942498 | 1.493818 | -2.375595 |
| 1 | 4.598561 | 3.278283 | -1.273465 |
| 6 | 6.895302 | 3.715954 | -2.679269 |
| 1 | 5.366545 | 3.584345 | -4.207693 |
| 1 | 5.120475 | 4.907668 | -3.070460 |
| 6 | 6.399909 | 1.350633 | -1.912034 |
| 1 | 4.845037 | 1.110457 | -3.400298 |
| 1 | 4.276711 | 0.894220 | -1.750663 |
| 6 | 7.348769 | 2.249726 | -2.717391 |
| 1 | 7.555894 | 4.338659 | -3.295676 |
| 1 | 6.977257 | 4.093523 | -1.649997 |
| 1 | 6.719155 | 0.305555 | -1.989913 |
| 1 | 6.460854 | 1.614145 | -0.846500 |
| 1 | 8.375375 | 2.157143 | -2.340502 |
| 1 | 7.365952 | 1.906836 | -3.762423 |
| 1 | 0.320928 | 0.640508 | 2.927202 |
| 8 | 0.036589 | 0.970780 | 3.807155 |
| 1 | 0.859516 | 1.232691 | 4.236896 |

---

(*S*,*R*,*S*)-**3a'**endo-*re*

---

Number of imaginary frequencies : 0
The smallest frequencies are : 11.5838 12.8185 16.8506 cm(-1)

Electronic energy : HF=-6614.0107841
Zero-point correction= 1.888206 (Hartree/Particle)
Thermal correction to Energy= 1.999324
Thermal correction to Enthalpy= 2.000268
Thermal correction to Gibbs Free Energy= 1.734464
Sum of electronic and zero-point Energies= -6612.122578
Sum of electronic and thermal Energies= -6612.011460
Sum of electronic and thermal Enthalpies= -6612.010516
Sum of electronic and thermal Free Energies= -6612.276320

........................................................

Cartesian Coordinates

........................................................

| | | | |
|---|---|---|---|
| 6 | -3.440044 | -1.244952 | 2.492093 |
| 6 | -4.734147 | -0.971887 | 2.014195 |
| 6 | -5.803847 | -1.752967 | 2.475938 |
| 6 | -5.580663 | -2.791786 | 3.384033 |
| 6 | -4.290634 | -3.058412 | 3.847122 |
| 6 | -3.222803 | -2.274974 | 3.405049 |
| 15 | -4.861540 | 0.318799 | 0.695457 |
| 6 | -6.602322 | 0.236086 | 0.081685 |
| 6 | -7.709248 | 0.488797 | 0.911246 |
| 6 | -9.006151 | 0.412484 | 0.404492 |
| 6 | -9.214847 | 0.098618 | -0.942226 |
| 6 | -8.122706 | -0.130112 | -1.779265 |
| 6 | -6.824222 | -0.059434 | -1.269649 |
| 46 | -3.154346 | -0.151833 | -0.874103 |
| 6 | -1.199687 | 0.298748 | -1.777467 |
| 6 | -1.358016 | 1.135106 | -0.663784 |
| 6 | -1.547634 | 2.632260 | -0.753213 |
| 15 | -3.572498 | -2.323828 | -1.740065 |
| 6 | -5.292112 | -2.994472 | -1.849472 |
| 6 | -5.987662 | -3.267917 | -0.658584 |

| | | | |
|---|---|---|---|
| 6 | -7.326535 | -3.651795 | -0.688418 |
| 6 | -8.001845 | -3.757418 | -1.907331 |
| 6 | -7.322577 | -3.482943 | -3.095260 |
| 6 | -5.977069 | -3.106366 | -3.068577 |
| 6 | -4.927105 | 1.908369 | 1.648396 |
| 6 | -5.545685 | 3.026868 | 1.061418 |
| 6 | -5.489500 | 4.278189 | 1.676589 |
| 6 | -4.800892 | 4.438533 | 2.881697 |
| 6 | -4.184527 | 3.334758 | 3.473110 |
| 6 | -4.252674 | 2.078983 | 2.866405 |
| 6 | -2.920016 | -2.705893 | -3.430237 |
| 6 | -2.692152 | -4.015908 | -3.880831 |
| 6 | -2.230269 | -4.248582 | -5.176310 |
| 6 | -1.992743 | -3.175752 | -6.041587 |
| 6 | -2.223012 | -1.869928 | -5.604889 |
| 6 | -2.681235 | -1.637207 | -4.305197 |
| 6 | -2.727146 | -3.564449 | -0.655642 |
| 6 | -1.608253 | -3.134526 | 0.073777 |
| 6 | -0.927570 | -4.009299 | 0.920649 |
| 6 | -1.359900 | -5.329096 | 1.054211 |
| 6 | -2.469605 | -5.772187 | 0.329234 |
| 6 | -3.149997 | -4.897496 | -0.520391 |
| 1 | -2.181294 | 2.911557 | -1.596779 |
| 1 | -1.325232 | 0.700129 | -2.779308 |
| 1 | -0.566623 | -0.580960 | -1.711600 |
| 1 | -6.073570 | 2.920684 | 0.117888 |
| 1 | -3.753711 | 1.238705 | 3.336838 |
| 1 | -5.973609 | 5.130163 | 1.206513 |
| 1 | -3.628360 | 3.453765 | 4.398005 |
| 1 | -4.735421 | 5.415450 | 3.351146 |
| 1 | -7.553655 | 0.755205 | 1.952706 |
| 1 | -5.973807 | -0.243528 | -1.917280 |
| 1 | -9.853312 | 0.604663 | 1.057318 |
| 1 | -8.275490 | -0.375762 | -2.825670 |
| 1 | -10.225817 | 0.039397 | -1.336112 |
| 1 | -6.808928 | -1.575561 | 2.110502 |
| 1 | -2.591407 | -0.658672 | 2.153869 |
| 1 | -6.418258 | -3.397042 | 3.721222 |
| 1 | -2.214915 | -2.474622 | 3.749611 |
| 1 | -4.116815 | -3.874300 | 4.543583 |
| 1 | -2.864497 | -4.856069 | -3.216227 |
| 1 | -2.844940 | -0.621428 | -3.958833 |
| 1 | -2.055200 | -5.267142 | -5.511031 |
| 1 | -2.039738 | -1.030524 | -6.269762 |
| 1 | -1.629678 | -3.358848 | -7.049096 |
| 1 | -5.481028 | -3.175700 | 0.296080 |
| 1 | -5.462676 | -2.903089 | -4.002755 |
| 1 | -7.845799 | -3.857147 | 0.243747 |
| 1 | -7.837075 | -3.565085 | -4.049163 |
| 1 | -9.048453 | -4.047805 | -1.929769 |
| 1 | -1.261411 | -2.113022 | -0.010089 |
| 1 | -4.022070 | -5.251002 | -1.061529 |
| 1 | -0.070075 | -3.643195 | 1.475095 |
| 1 | -2.810803 | -6.799410 | 0.426768 |
| 1 | -0.836544 | -6.010956 | 1.718750 |
| 1 | -0.884319 | 0.823416 | 0.264799 |
| 6 | 0.305304 | 3.728573 | 0.373189 |
| 6 | -0.981077 | 4.345648 | 0.994137 |
| 1 | 0.658306 | 2.858942 | 0.938486 |
| 1 | 1.123577 | 4.439564 | 0.277370 |
| 7 | -0.227816 | 3.339619 | -0.933720 |
| 16 | 0.688612 | 3.101673 | -2.267804 |

| | | | |
|---|---|---|---|
| 8 | -0.231257 | 2.925459 | -3.397830 |
| 8 | 1.716632 | 2.045482 | -2.070405 |
| 6 | -2.008779 | 3.246724 | 0.593162 |
| 1 | -1.987867 | 2.466087 | 1.351377 |
| 1 | -3.029758 | 3.617539 | 0.562340 |
| 6 | -1.208687 | 5.685551 | 0.272162 |
| 6 | -2.325758 | 5.943953 | -0.529119 |
| 6 | -0.228549 | 6.688876 | 0.371447 |
| 6 | -2.459745 | 7.157214 | -1.212005 |
| 1 | -3.103313 | 5.197456 | -0.639250 |
| 6 | -0.355793 | 7.898634 | -0.306818 |
| 1 | 0.644755 | 6.521661 | 0.995806 |
| 6 | -1.476577 | 8.139503 | -1.106087 |
| 1 | -3.336748 | 7.327481 | -1.830795 |
| 1 | 0.423241 | 8.650493 | -0.215875 |
| 1 | -1.578420 | 9.080249 | -1.640068 |
| 6 | -0.921875 | 4.490956 | 2.520130 |
| 6 | -0.510925 | 3.402480 | 3.309287 |
| 6 | -1.356232 | 5.650686 | 3.176500 |
| 6 | -0.523561 | 3.481772 | 4.701559 |
| 1 | -0.222961 | 2.462399 | 2.850132 |
| 6 | -1.366447 | 5.731272 | 4.571882 |
| 1 | -1.703436 | 6.499116 | 2.598325 |
| 6 | -0.947087 | 4.648098 | 5.343976 |
| 1 | -0.216345 | 2.616622 | 5.282353 |
| 1 | -1.711354 | 6.643568 | 5.051434 |
| 1 | -0.957631 | 4.707720 | 6.428960 |
| 6 | 1.547762 | 4.679786 | -2.452072 |
| 6 | 2.780533 | 5.009745 | -1.798163 |
| 6 | 0.888091 | 5.584212 | -3.259850 |
| 6 | 3.524097 | 4.142826 | -0.946653 |
| 6 | 3.297729 | 6.334162 | -2.026969 |
| 6 | 1.415070 | 6.874897 | -3.470682 |
| 1 | -0.042918 | 5.296541 | -3.733739 |
| 6 | 4.697989 | 4.562668 | -0.359885 |
| 1 | 3.177141 | 3.137241 | -0.765798 |
| 6 | 4.514152 | 6.727312 | -1.404753 |
| 6 | 2.594711 | 7.238225 | -2.866212 |
| 1 | 0.879019 | 7.572088 | -4.106296 |
| 6 | 5.203356 | 5.863681 | -0.586750 |
| 1 | 5.241551 | 3.877424 | 0.282620 |
| 1 | 4.887437 | 7.731080 | -1.590020 |
| 1 | 3.008839 | 8.231300 | -3.019120 |
| 1 | 6.131146 | 6.174729 | -0.115875 |
| 6 | 4.143081 | -1.923812 | -1.093222 |
| 6 | 4.429877 | -1.619451 | -2.411182 |
| 6 | 5.780034 | -1.791207 | -2.864928 |
| 6 | 6.161151 | -1.587707 | -4.220129 |
| 1 | 5.405114 | -1.301842 | -4.941845 |
| 6 | 7.469148 | -1.753539 | -4.618182 |
| 1 | 7.738842 | -1.598112 | -5.658853 |
| 6 | 8.467539 | -2.125334 | -3.684604 |
| 1 | 9.495667 | -2.247403 | -4.013057 |
| 6 | 8.131730 | -2.335520 | -2.367490 |
| 1 | 8.887117 | -2.629116 | -1.643114 |
| 6 | 6.787637 | -2.185461 | -1.926211 |
| 6 | 6.429339 | -2.423175 | -0.578720 |
| 1 | 7.212872 | -2.684562 | 0.125615 |
| 6 | 5.118737 | -2.335743 | -0.139432 |
| 6 | 4.783223 | -2.712594 | 1.259448 |
| 6 | 5.453923 | -3.769077 | 1.851661 |
| 1 | 6.144383 | -4.358888 | 1.256923 |

| | | | |
|---|---|---|---|
| 6 | 5.285322 | -4.104247 | 3.214802 |
| 6 | 6.036597 | -5.153570 | 3.813099 |
| 1 | 6.735859 | -5.710031 | 3.194552 |
| 6 | 5.891864 | -5.449631 | 5.148342 |
| 1 | 6.473380 | -6.248630 | 5.598884 |
| 6 | 4.987284 | -4.702867 | 5.941814 |
| 1 | 4.887295 | -4.930057 | 6.999273 |
| 6 | 4.232931 | -3.692639 | 5.387470 |
| 1 | 3.545551 | -3.130089 | 6.007886 |
| 6 | 4.348263 | -3.367265 | 4.007233 |
| 6 | 3.564068 | -2.336849 | 3.384494 |
| 6 | 3.824682 | -2.018983 | 2.062592 |
| 6 | 3.371631 | -1.091805 | -3.330924 |
| 6 | 3.484307 | 0.276231 | -3.767621 |
| 6 | 4.493566 | 1.159806 | -3.290126 |
| 1 | 5.202207 | 0.807717 | -2.549774 |
| 6 | 4.572196 | 2.461063 | -3.733376 |
| 1 | 5.342944 | 3.117324 | -3.340540 |
| 6 | 3.640605 | 2.960945 | -4.673306 |
| 1 | 3.705863 | 3.993511 | -5.003653 |
| 6 | 2.639030 | 2.142057 | -5.140735 |
| 1 | 1.895681 | 2.519627 | -5.837565 |
| 6 | 2.531891 | 0.796062 | -4.701597 |
| 6 | 1.476345 | -0.042400 | -5.138387 |
| 1 | 0.739234 | 0.357868 | -5.829704 |
| 6 | 1.369682 | -1.330406 | -4.679154 |
| 1 | 0.541074 | -1.945467 | -5.013863 |
| 6 | 2.315523 | -1.885937 | -3.771851 |
| 6 | 2.462104 | -1.683936 | 4.165754 |
| 6 | 1.351457 | -2.521732 | 4.552496 |
| 6 | 1.193543 | -3.856095 | 4.079248 |
| 1 | 1.904075 | -4.252103 | 3.363731 |
| 6 | 0.154751 | -4.650744 | 4.512884 |
| 1 | 0.061500 | -5.665209 | 4.137149 |
| 6 | -0.790177 | -4.159336 | 5.443413 |
| 1 | -1.598042 | -4.799749 | 5.784817 |
| 6 | -0.692227 | -2.862374 | 5.894189 |
| 1 | -1.428301 | -2.460281 | 6.585206 |
| 6 | 0.361490 | -2.015555 | 5.456418 |
| 6 | 0.465292 | -0.670088 | 5.897569 |
| 1 | -0.284548 | -0.278429 | 6.579916 |
| 6 | 1.493041 | 0.127333 | 5.458421 |
| 1 | 1.546423 | 1.157353 | 5.795350 |
| 6 | 2.512480 | -0.357050 | 4.586492 |
| 8 | 2.560411 | 0.612613 | -0.185479 |
| 8 | 0.784361 | -1.175653 | 0.594192 |
| 8 | 2.801269 | -1.932921 | -0.709186 |
| 8 | 3.215117 | -0.877381 | 1.558594 |
| 15 | 2.199889 | -0.832959 | 0.303212 |
| 6 | 3.570764 | 0.627893 | 4.126731 |
| 6 | 2.919432 | 1.765168 | 3.302421 |
| 6 | 4.418005 | 1.224489 | 5.271889 |
| 1 | 4.267074 | 0.105723 | 3.466243 |
| 6 | 3.978170 | 2.717259 | 2.737831 |
| 1 | 2.232922 | 2.334154 | 3.942408 |
| 1 | 2.313564 | 1.341930 | 2.498407 |
| 6 | 5.480974 | 2.186390 | 4.714492 |
| 1 | 3.770340 | 1.766528 | 5.974179 |
| 1 | 4.894231 | 0.416586 | 5.840680 |
| 6 | 4.853365 | 3.297594 | 3.858080 |
| 1 | 3.495745 | 3.524100 | 2.174688 |
| 1 | 4.606497 | 2.168023 | 2.022728 |

| | | | |
|---|---|---|---|
| 1 | 6.061253 | 2.621210 | 5.537488 |
| 1 | 6.189799 | 1.614780 | 4.098443 |
| 1 | 5.637211 | 3.941233 | 3.439597 |
| 1 | 4.231350 | 3.938118 | 4.499632 |
| 6 | 2.129458 | -3.331518 | -3.346313 |
| 6 | 0.773231 | -3.553550 | -2.639627 |
| 6 | 2.283420 | -4.307647 | -4.535423 |
| 1 | 2.915274 | -3.590706 | -2.630292 |
| 6 | 0.598696 | -5.011507 | -2.195502 |
| 1 | -0.043060 | -3.290155 | -3.321201 |
| 1 | 0.693925 | -2.887076 | -1.776819 |
| 6 | 2.124965 | -5.764939 | -4.077336 |
| 1 | 1.524275 | -4.081080 | -5.296105 |
| 1 | 3.260975 | -4.158385 | -5.010732 |
| 6 | 0.781148 | -5.985457 | -3.367970 |
| 1 | -0.387927 | -5.141641 | -1.739472 |
| 1 | 1.336054 | -5.240359 | -1.412534 |
| 1 | 2.222496 | -6.442351 | -4.935060 |
| 1 | 2.943576 | -6.013576 | -3.386355 |
| 1 | 0.699041 | -7.023123 | -3.020369 |
| 1 | -0.032068 | -5.828316 | -4.089801 |
| 1 | -0.036420 | -0.337668 | 1.951393 |
| 8 | -0.443105 | 0.240040 | 2.625287 |
| 1 | -0.036438 | -0.020022 | 3.461547 |
| 1 | 2.056548 | 1.049794 | -0.940792 |

---

(*S*,*R*,*S*)-**3a'**exo-*si*

---

Number of imaginary frequencies : 0
The smallest frequencies are : 12.7228 13.4452 14.1620 cm(-1)

Electronic energy : HF=-6614.010006
Zero-point correction= 1.887746 (Hartree/Particle)
Thermal correction to Energy= 1.999213
Thermal correction to Enthalpy= 2.000157
Thermal correction to Gibbs Free Energy= 1.733067
Sum of electronic and zero-point Energies= -6612.122260
Sum of electronic and thermal Energies= -6612.010793
Sum of electronic and thermal Enthalpies= -6612.009849
Sum of electronic and thermal Free Energies= -6612.276939

Cartesian Coordinates

| | | | |
|---|---|---|---|
| 6 | -3.839398 | -1.248299 | 2.496449 |
| 6 | -5.048302 | -0.738519 | 1.992724 |
| 6 | -6.256637 | -1.170378 | 2.559750 |
| 6 | -6.256562 | -2.109281 | 3.593559 |
| 6 | -5.050731 | -2.625927 | 4.075018 |
| 6 | -3.842123 | -2.185083 | 3.530988 |
| 15 | -4.931080 | 0.391349 | 0.530696 |
| 6 | -6.634719 | 0.364507 | -0.192903 |
| 6 | -7.727047 | 1.014767 | 0.406922 |
| 6 | -8.999411 | 0.933929 | -0.159682 |
| 6 | -9.199985 | 0.205466 | -1.335588 |
| 6 | -8.120145 | -0.433520 | -1.946272 |
| 6 | -6.846146 | -0.349114 | -1.381433 |
| 46 | -3.225075 | -0.302658 | -0.967092 |
| 6 | -1.571160 | 0.118128 | -2.316228 |
| 6 | -1.962780 | 1.350250 | -1.766891 |
| 6 | -1.040001 | 2.118139 | -0.857578 |
| 15 | -3.472760 | -2.655512 | -1.228860 |

| | | | |
|---|---|---|---|
| 6 | -5.172190 | -3.382522 | -1.130074 |
| 6 | -5.800418 | -3.481848 | 0.122982 |
| 6 | -7.133923 | -3.875627 | 0.221641 |
| 6 | -7.867348 | -4.174865 | -0.928863 |
| 6 | -7.250688 | -4.088089 | -2.178158 |
| 6 | -5.913464 | -3.697394 | -2.279711 |
| 6 | -4.923621 | 2.066824 | 1.318170 |
| 6 | -5.179173 | 3.188374 | 0.508625 |
| 6 | -5.110640 | 4.477536 | 1.032605 |
| 6 | -4.785263 | 4.669433 | 2.378586 |
| 6 | -4.532050 | 3.564205 | 3.190816 |
| 6 | -4.597587 | 2.269870 | 2.665610 |
| 6 | -2.877418 | -3.355832 | -2.837664 |
| 6 | -2.314850 | -4.634549 | -2.961790 |
| 6 | -1.894120 | -5.107189 | -4.206656 |
| 6 | -2.033198 | -4.311715 | -5.346707 |
| 6 | -2.608341 | -3.043244 | -5.237613 |
| 6 | -3.025114 | -2.570142 | -3.991852 |
| 6 | -2.528283 | -3.599732 | 0.042129 |
| 6 | -1.404810 | -2.968092 | 0.596401 |
| 6 | -0.646360 | -3.600831 | 1.580595 |
| 6 | -1.006129 | -4.874140 | 2.025823 |
| 6 | -2.119597 | -5.515957 | 1.476835 |
| 6 | -2.876294 | -4.885003 | 0.487648 |
| 1 | -0.286227 | 1.438322 | -0.436733 |
| 1 | -0.647784 | -0.350692 | -1.981887 |
| 1 | -1.877361 | -0.163325 | -3.318487 |
| 1 | -5.419233 | 3.050920 | -0.542579 |
| 1 | -4.390119 | 1.420691 | 3.308152 |
| 1 | -5.282333 | 5.333300 | 0.387156 |
| 1 | -4.275553 | 3.705168 | 4.237645 |
| 1 | -4.716986 | 5.674651 | 2.782944 |
| 1 | -7.580994 | 1.591343 | 1.315222 |
| 1 | -6.008822 | -0.842246 | -1.861733 |
| 1 | -9.834233 | 1.441028 | 0.316697 |
| 1 | -8.264305 | -1.002447 | -2.860071 |
| 1 | -10.191902 | 0.142967 | -1.774907 |
| 1 | -7.202803 | -0.804123 | 2.176908 |
| 1 | -2.892574 | -0.912192 | 2.086039 |
| 1 | -7.201212 | -2.446229 | 4.012663 |
| 1 | -2.897065 | -2.577685 | 3.890885 |
| 1 | -5.054000 | -3.369595 | 4.867836 |
| 1 | -2.188467 | -5.260594 | -2.084902 |
| 1 | -3.466005 | -1.581361 | -3.908729 |
| 1 | -1.452385 | -6.096869 | -4.283333 |
| 1 | -2.725726 | -2.418256 | -6.118550 |
| 1 | -1.697469 | -4.678405 | -6.312674 |
| 1 | -5.246040 | -3.250162 | 1.024599 |
| 1 | -5.451353 | -3.636964 | -3.259834 |
| 1 | -7.598615 | -3.937161 | 1.201581 |
| 1 | -7.808905 | -4.326421 | -3.079781 |
| 1 | -8.909304 | -4.472744 | -0.852260 |
| 1 | -1.121383 | -1.976358 | 0.264657 |
| 1 | -3.746827 | -5.386735 | 0.077410 |
| 1 | 0.211831 | -3.086514 | 1.998826 |
| 1 | -2.401728 | -6.508584 | 1.817773 |
| 1 | -0.420746 | -5.365062 | 2.796821 |
| 1 | -2.634585 | 1.991869 | -2.336250 |
| 6 | 0.266913 | 4.095723 | -0.592631 |
| 6 | -0.900532 | 4.273725 | 0.427995 |
| 1 | 1.122105 | 3.619960 | -0.094839 |
| 1 | 0.589982 | 5.038345 | -1.033735 |

| | | | |
|---|---|---|---|
| 7 | -0.324222 | 3.217479 | -1.613987 |
| 16 | 0.611546 | 2.728120 | -2.918649 |
| 8 | -0.273008 | 2.463489 | -4.055833 |
| 8 | 1.520339 | 1.618536 | -2.524989 |
| 6 | -1.687265 | 2.923011 | 0.280815 |
| 1 | -1.654513 | 2.307869 | 1.178209 |
| 1 | -2.728970 | 3.121479 | 0.046079 |
| 6 | -1.806838 | 5.453003 | -0.002056 |
| 6 | -2.149038 | 6.493987 | 0.871321 |
| 6 | -2.349563 | 5.485142 | -1.300327 |
| 6 | -2.996161 | 7.530443 | 0.467415 |
| 1 | -1.764104 | 6.499133 | 1.883809 |
| 6 | -3.190521 | 6.520354 | -1.706158 |
| 1 | -2.108363 | 4.689010 | -1.995369 |
| 6 | -3.522065 | 7.551863 | -0.823423 |
| 1 | -3.244471 | 8.320421 | 1.171510 |
| 1 | -3.591806 | 6.516535 | -2.716341 |
| 1 | -4.180866 | 8.356611 | -1.138090 |
| 6 | -0.330484 | 4.528263 | 1.824885 |
| 6 | -0.932958 | 4.010973 | 2.978880 |
| 6 | 0.766309 | 5.392672 | 1.985637 |
| 6 | -0.458378 | 4.347946 | 4.249414 |
| 1 | -1.793748 | 3.359454 | 2.893025 |
| 6 | 1.244621 | 5.728930 | 3.252349 |
| 1 | 1.243729 | 5.828641 | 1.112880 |
| 6 | 0.631982 | 5.207154 | 4.394635 |
| 1 | -0.951819 | 3.940478 | 5.127832 |
| 1 | 2.092999 | 6.400937 | 3.345633 |
| 1 | 0.998706 | 5.469691 | 5.383021 |
| 6 | 1.579592 | 4.202671 | -3.320453 |
| 6 | 2.843959 | 4.530643 | -2.730374 |
| 6 | 0.997925 | 5.001549 | -4.285204 |
| 6 | 3.508009 | 3.770956 | -1.728697 |
| 6 | 3.490567 | 5.724414 | -3.209070 |
| 6 | 1.641797 | 6.177058 | -4.726200 |
| 1 | 0.047733 | 4.710712 | -4.716874 |
| 6 | 4.737147 | 4.159323 | -1.242070 |
| 1 | 3.059930 | 2.868223 | -1.349281 |
| 6 | 4.758257 | 6.092813 | -2.681934 |
| 6 | 2.863572 | 6.524200 | -4.201246 |
| 1 | 1.167947 | 6.792268 | -5.484248 |
| 6 | 5.374275 | 5.327295 | -1.719938 |
| 1 | 5.219938 | 3.552388 | -0.483496 |
| 1 | 5.231183 | 6.996140 | -3.057816 |
| 1 | 3.372874 | 7.422214 | -4.540637 |
| 1 | 6.344323 | 5.616017 | -1.326412 |
| 6 | 4.244115 | -1.965896 | -0.771772 |
| 6 | 4.496684 | -1.942293 | -2.130663 |
| 6 | 5.850205 | -2.123203 | -2.567303 |
| 6 | 6.204545 | -2.187954 | -3.943070 |
| 1 | 5.423610 | -2.111547 | -4.690642 |
| 6 | 7.517671 | -2.345195 | -4.327084 |
| 1 | 7.767631 | -2.394469 | -5.382993 |
| 6 | 8.546964 | -2.443341 | -3.358617 |
| 1 | 9.578571 | -2.561887 | -3.677310 |
| 6 | 8.237661 | -2.392999 | -2.019321 |
| 1 | 9.018600 | -2.473314 | -1.267454 |
| 6 | 6.890098 | -2.242213 | -1.588126 |
| 6 | 6.560743 | -2.209873 | -0.212553 |
| 1 | 7.364982 | -2.269141 | 0.514238 |
| 6 | 5.251598 | -2.102741 | 0.226250 |
| 6 | 4.938794 | -2.184156 | 1.677109 |

| | | | |
|---|---|---|---|
| 6 | 5.656381 | -3.053302 | 2.481648 |
| 1 | 6.394402 | -3.708363 | 2.029198 |
| 6 | 5.465150 | -3.119120 | 3.881480 |
| 6 | 6.259293 | -3.972876 | 4.696462 |
| 1 | 7.020879 | -4.585589 | 4.221070 |
| 6 | 6.076380 | -4.015003 | 6.059113 |
| 1 | 6.691595 | -4.665590 | 6.673798 |
| 6 | 5.086653 | -3.202753 | 6.665046 |
| 1 | 4.954233 | -3.231779 | 7.742742 |
| 6 | 4.290516 | -2.378011 | 5.901648 |
| 1 | 3.534271 | -1.762480 | 6.374981 |
| 6 | 4.448858 | -2.314469 | 4.489499 |
| 6 | 3.631918 | -1.476418 | 3.660239 |
| 6 | 3.931496 | -1.394040 | 2.310867 |
| 6 | 3.385049 | -1.703999 | -3.106340 |
| 6 | 3.375761 | -0.462549 | -3.835600 |
| 6 | 4.341949 | 0.562945 | -3.630278 |
| 1 | 5.106160 | 0.429827 | -2.873909 |
| 6 | 4.312863 | 1.728257 | -4.363369 |
| 1 | 5.057135 | 2.497327 | -4.179863 |
| 6 | 3.308724 | 1.943379 | -5.336387 |
| 1 | 3.291421 | 2.871745 | -5.899755 |
| 6 | 2.341907 | 0.986345 | -5.541482 |
| 1 | 1.542376 | 1.153238 | -6.257537 |
| 6 | 2.345433 | -0.225816 | -4.802261 |
| 6 | 1.332319 | -1.202170 | -4.975731 |
| 1 | 0.530530 | -1.010975 | -5.684373 |
| 6 | 1.350628 | -2.366605 | -4.250859 |
| 1 | 0.557691 | -3.093357 | -4.396335 |
| 6 | 2.382563 | -2.649294 | -3.311672 |
| 6 | 2.446892 | -0.760764 | 4.234906 |
| 6 | 1.341630 | -1.568095 | 4.694071 |
| 6 | 1.325746 | -2.989188 | 4.595012 |
| 1 | 2.154551 | -3.500132 | 4.121036 |
| 6 | 0.278110 | -3.729333 | 5.098144 |
| 1 | 0.303758 | -4.812461 | 5.026947 |
| 6 | -0.832261 | -3.095117 | 5.701505 |
| 1 | -1.654276 | -3.691452 | 6.086656 |
| 6 | -0.869036 | -1.721834 | 5.783464 |
| 1 | -1.724548 | -1.216909 | 6.224213 |
| 6 | 0.205894 | -0.933277 | 5.294544 |
| 6 | 0.186203 | 0.482375 | 5.392110 |
| 1 | -0.679346 | 0.967611 | 5.836016 |
| 6 | 1.241603 | 1.227604 | 4.929286 |
| 1 | 1.200805 | 2.308256 | 5.004477 |
| 6 | 2.394536 | 0.625149 | 4.347517 |
| 8 | 2.728254 | 0.652144 | -0.503351 |
| 8 | 0.870739 | -0.804184 | 0.640048 |
| 8 | 2.908058 | -1.937651 | -0.361571 |
| 8 | 3.302276 | -0.387447 | 1.586004 |
| 15 | 2.308806 | -0.611013 | 0.334731 |
| 6 | 3.512401 | 1.542505 | 3.888302 |
| 6 | 3.023156 | 2.534928 | 2.809338 |
| 6 | 4.159972 | 2.309628 | 5.064751 |
| 1 | 4.302268 | 0.934036 | 3.438901 |
| 6 | 4.164595 | 3.434397 | 2.321530 |
| 1 | 2.225647 | 3.162772 | 3.219428 |
| 1 | 2.593742 | 1.984842 | 1.969047 |
| 6 | 5.304340 | 3.209747 | 4.574332 |
| 1 | 3.401566 | 2.929100 | 5.562446 |
| 1 | 4.525849 | 1.596101 | 5.813165 |
| 6 | 4.825997 | 4.182803 | 3.487039 |

| | | | |
|---|---|---|---|
| 1 | 3.787429 | 4.143784 | 1.576364 |
| 1 | 4.916297 | 2.813342 | 1.813021 |
| 1 | 5.736653 | 3.760825 | 5.418609 |
| 1 | 6.107346 | 2.578603 | 4.167321 |
| 1 | 5.663116 | 4.793395 | 3.126292 |
| 1 | 4.093692 | 4.876350 | 3.923675 |
| 6 | 2.346446 | -3.985880 | -2.592100 |
| 6 | 1.060910 | -4.154197 | -1.753247 |
| 6 | 2.508959 | -5.168565 | -3.575305 |
| 1 | 3.193867 | -4.036487 | -1.901167 |
| 6 | 1.036050 | -5.502053 | -1.021852 |
| 1 | 0.187497 | -4.090902 | -2.410092 |
| 1 | 0.976564 | -3.332060 | -1.039664 |
| 6 | 2.489638 | -6.514741 | -2.836495 |
| 1 | 1.693373 | -5.146890 | -4.310305 |
| 1 | 3.443469 | -5.053184 | -4.138226 |
| 6 | 1.213621 | -6.674478 | -1.997114 |
| 1 | 0.097937 | -5.603008 | -0.466712 |
| 1 | 1.843454 | -5.524398 | -0.275214 |
| 1 | 2.583152 | -7.339220 | -3.554480 |
| 1 | 3.366079 | -6.574271 | -2.174920 |
| 1 | 1.230303 | -7.628019 | -1.454324 |
| 1 | 0.346162 | -6.711455 | -2.671602 |
| 1 | -0.378485 | 0.029127 | 1.709917 |
| 8 | -0.990992 | 0.490326 | 2.309557 |
| 1 | -0.445788 | 0.730880 | 3.069570 |
| 1 | 2.140184 | 0.928710 | -1.267499 |

---

**1b**

---

Number of imaginary frequencies : 0
The smallest frequencies are : 11.8833 15.5756 16.4986 cm(-1)

Electronic energy : HF=-5577.5984873
Zero-point correction= 1.613385 (Hartree/Particle)
Thermal correction to Energy= 1.706957
Thermal correction to Enthalpy= 1.707901
Thermal correction to Gibbs Free Energy= 1.478460
Sum of electronic and zero-point Energies= -5575.985103
Sum of electronic and thermal Energies= -5575.891530
Sum of electronic and thermal Enthalpies= -5575.890586
Sum of electronic and thermal Free Energies= -5576.120027

............................................................
Cartesian Coordinates
............................................................

| | | | |
|---|---|---|---|
| 46 | 0.547203 | -1.350229 | -0.669007 |
| 6 | 2.599580 | -0.813605 | -0.822407 |
| 6 | 1.829089 | 0.325763 | -1.171149 |
| 15 | 0.044262 | -3.507582 | 0.096397 |
| 6 | 1.425196 | -4.404336 | 0.939490 |
| 6 | 1.977374 | -3.832448 | 2.100198 |
| 6 | 3.089848 | -4.401883 | 2.717976 |
| 6 | 3.668021 | -5.558646 | 2.188287 |
| 6 | 3.118399 | -6.145055 | 1.048047 |
| 6 | 2.004828 | -5.573471 | 0.425681 |
| 6 | -1.310495 | -3.632980 | 1.349071 |
| 6 | -1.345056 | -4.608273 | 2.358728 |
| 6 | -2.400072 | -4.640999 | 3.273512 |
| 6 | -3.432160 | -3.703508 | 3.189663 |
| 6 | -3.404685 | -2.729153 | 2.189470 |
| 6 | -2.348265 | -2.689721 | 1.278215 |

| | | | |
|---|---|---|---|
| 6 | -0.467440 | -4.699559 | -1.216761 |
| 6 | -0.079483 | -4.424300 | -2.536360 |
| 6 | -0.407675 | -5.304755 | -3.569069 |
| 6 | -1.138175 | -6.462799 | -3.294556 |
| 6 | -1.536731 | -6.740329 | -1.983350 |
| 6 | -1.199175 | -5.866826 | -0.948674 |
| 1 | 3.045849 | -1.368774 | -1.645829 |
| 1 | -0.543426 | -5.335834 | 2.439532 |
| 1 | -2.321625 | -1.925264 | 0.509976 |
| 1 | -2.412827 | -5.398614 | 4.052461 |
| 1 | -4.198703 | -1.992483 | 2.122720 |
| 1 | -4.251649 | -3.728934 | 3.902720 |
| 1 | 1.543424 | -2.927687 | 2.513180 |
| 1 | 1.597279 | -6.035540 | -0.466995 |
| 1 | 3.520723 | -3.927586 | 3.594314 |
| 1 | 3.561467 | -7.045004 | 0.630572 |
| 1 | 4.547245 | -5.990039 | 2.657509 |
| 1 | 0.473594 | -3.512644 | -2.747587 |
| 1 | -1.513528 | -6.089042 | 0.066471 |
| 1 | -0.103923 | -5.078628 | -4.587217 |
| 1 | -2.109788 | -7.637595 | -1.766435 |
| 1 | -1.401078 | -7.144837 | -4.098433 |
| 1 | 1.663560 | 1.099151 | -0.419671 |
| 6 | 1.704967 | 0.773598 | -2.605836 |
| 8 | 0.329179 | 0.776212 | -3.109692 |
| 6 | 3.301142 | -0.972249 | 0.517401 |
| 6 | 4.866623 | -1.108064 | 0.501534 |
| 1 | 3.022080 | -0.124343 | 1.145750 |
| 1 | 2.920980 | -1.856643 | 1.026072 |
| 6 | 5.503064 | 0.307202 | 0.321314 |
| 1 | 6.589204 | 0.254037 | 0.231955 |
| 1 | 5.296087 | 0.867664 | 1.238129 |
| 6 | 5.305774 | -2.145491 | -0.546270 |
| 6 | 4.554259 | -3.323611 | -0.698134 |
| 6 | 6.478206 | -2.021983 | -1.301313 |
| 6 | 4.939239 | -4.320392 | -1.591392 |
| 1 | 3.658078 | -3.471853 | -0.111196 |
| 6 | 6.867443 | -3.019290 | -2.201148 |
| 1 | 7.105053 | -1.145172 | -1.213336 |
| 6 | 6.099037 | -4.171018 | -2.356100 |
| 1 | 4.330254 | -5.215461 | -1.681068 |
| 1 | 7.775006 | -2.883099 | -2.782745 |
| 1 | 6.399763 | -4.943921 | -3.058155 |
| 6 | 5.322224 | -1.585497 | 1.906877 |
| 6 | 6.395475 | -2.472437 | 2.074109 |
| 6 | 4.679606 | -1.121304 | 3.067945 |
| 6 | 6.798376 | -2.892198 | 3.343944 |
| 1 | 6.915061 | -2.856088 | 1.203912 |
| 6 | 5.077991 | -1.537267 | 4.338919 |
| 1 | 3.843822 | -0.437813 | 2.988626 |
| 6 | 6.140188 | -2.431693 | 4.484676 |
| 1 | 7.628921 | -3.586854 | 3.436533 |
| 1 | 4.554188 | -1.162445 | 5.214406 |
| 1 | 6.450325 | -2.762495 | 5.471896 |
| 7 | 4.991732 | 1.147150 | -0.774440 |
| 1 | 3.976741 | 1.134987 | -0.858077 |
| 16 | 5.687160 | 1.149955 | -2.297720 |
| 8 | 7.140519 | 1.026790 | -2.094943 |
| 1 | -0.016445 | -0.121489 | -2.942763 |
| 1 | -0.598787 | 1.578538 | -2.219813 |
| 1 | 2.308094 | 0.140321 | -3.263539 |
| 1 | 2.031458 | 1.810334 | -2.734181 |

| | | | |
|---|---|---|---|
| 8 | 5.006002 | 0.230330 | -3.223338 |
| 6 | 5.297239 | 2.823363 | -2.838049 |
| 6 | 5.825038 | 3.970305 | -2.162827 |
| 6 | 4.488708 | 2.941099 | -3.948712 |
| 6 | 6.649136 | 3.918263 | -1.004537 |
| 6 | 5.458024 | 5.259466 | -2.681236 |
| 6 | 4.148179 | 4.217520 | -4.450808 |
| 1 | 4.115015 | 2.046207 | -4.432124 |
| 6 | 7.068077 | 5.076781 | -0.387416 |
| 1 | 6.956890 | 2.959987 | -0.609981 |
| 6 | 5.915481 | 6.431256 | -2.019922 |
| 6 | 4.623740 | 5.347610 | -3.829155 |
| 1 | 3.507572 | 4.292956 | -5.323512 |
| 6 | 6.698984 | 6.345457 | -0.892753 |
| 1 | 7.693660 | 5.014000 | 0.498219 |
| 1 | 5.626502 | 7.399170 | -2.420646 |
| 1 | 4.363138 | 6.333240 | -4.205900 |
| 1 | 7.037154 | 7.246817 | -0.390374 |
| 6 | -4.516086 | 0.742745 | -0.030352 |
| 6 | -5.482879 | -0.045990 | -0.616102 |
| 6 | -6.453035 | -0.674751 | 0.230572 |
| 6 | -7.446065 | -1.551331 | -0.282513 |
| 1 | -7.467973 | -1.751746 | -1.347740 |
| 6 | -8.361479 | -2.146435 | 0.557777 |
| 1 | -9.109894 | -2.819631 | 0.149957 |
| 6 | -8.335094 | -1.890791 | 1.950467 |
| 1 | -9.063012 | -2.368277 | 2.599912 |
| 6 | -7.391297 | -1.039119 | 2.477677 |
| 1 | -7.365217 | -0.833402 | 3.544669 |
| 6 | -6.432786 | -0.407937 | 1.638225 |
| 6 | -5.465138 | 0.486195 | 2.161654 |
| 1 | -5.468351 | 0.701140 | 3.226179 |
| 6 | -4.508542 | 1.074769 | 1.355525 |
| 6 | -3.565969 | 2.081653 | 1.899838 |
| 6 | -4.025479 | 3.073565 | 2.745545 |
| 1 | -5.085703 | 3.125554 | 2.975354 |
| 6 | -3.154957 | 4.038975 | 3.307071 |
| 6 | -3.635728 | 5.054112 | 4.178349 |
| 1 | -4.697914 | 5.084151 | 4.406983 |
| 6 | -2.774666 | 5.978466 | 4.724438 |
| 1 | -3.151254 | 6.749536 | 5.390235 |
| 6 | -1.392754 | 5.925734 | 4.420024 |
| 1 | -0.718982 | 6.657849 | 4.855779 |
| 6 | -0.895694 | 4.956858 | 3.575835 |
| 1 | 0.163470 | 4.923854 | 3.346871 |
| 6 | -1.758456 | 3.989658 | 2.991496 |
| 6 | -1.274317 | 2.969772 | 2.107630 |
| 6 | -2.175081 | 2.066661 | 1.584463 |
| 6 | -5.513262 | -0.217967 | -2.102414 |
| 6 | -4.903326 | -1.397890 | -2.650200 |
| 6 | -4.324160 | -2.405951 | -1.829352 |
| 1 | -4.329779 | -2.284099 | -0.753715 |
| 6 | -3.752897 | -3.530980 | -2.378988 |
| 1 | -3.311341 | -4.283043 | -1.734589 |
| 6 | -3.720947 | -3.709653 | -3.783060 |
| 1 | -3.257842 | -4.599191 | -4.197490 |
| 6 | -4.268789 | -2.754880 | -4.608610 |
| 1 | -4.251874 | -2.879516 | -5.688671 |
| 6 | -4.872836 | -1.586922 | -4.069484 |
| 6 | -5.457507 | -0.596057 | -4.896728 |
| 1 | -5.436849 | -0.728387 | -5.975691 |
| 6 | -6.052536 | 0.509266 | -4.343643 |

| | | | |
|---|---|---|---|
| 1 | -6.504069 | 1.252410 | -4.996037 |
| 6 | -6.104904 | 0.730800 | -2.936285 |
| 6 | 0.190314 | 2.882005 | 1.811923 |
| 6 | 0.734805 | 3.691377 | 0.761752 |
| 6 | -0.079555 | 4.487472 | -0.090605 |
| 1 | -1.153899 | 4.483560 | 0.052975 |
| 6 | 0.476028 | 5.233810 | -1.106089 |
| 1 | -0.166642 | 5.821934 | -1.755027 |
| 6 | 1.876364 | 5.236129 | -1.316219 |
| 1 | 2.304647 | 5.823935 | -2.122179 |
| 6 | 2.695198 | 4.488401 | -0.500741 |
| 1 | 3.769456 | 4.486900 | -0.645293 |
| 6 | 2.151341 | 3.700267 | 0.547610 |
| 6 | 2.976380 | 2.914441 | 1.393320 |
| 1 | 4.051506 | 2.933191 | 1.238121 |
| 6 | 2.421723 | 2.133838 | 2.378196 |
| 1 | 3.072103 | 1.544073 | 3.014725 |
| 6 | 1.016872 | 2.087975 | 2.600555 |
| 8 | -1.744402 | -0.549131 | -1.184872 |
| 8 | -1.296644 | 1.986075 | -1.576511 |
| 8 | -3.560542 | 1.346097 | -0.854747 |
| 8 | -1.681918 | 1.015447 | 0.811033 |
| 15 | -2.018263 | 0.857155 | -0.768127 |
| 6 | -6.789694 | 2.028949 | -2.502438 |
| 6 | -7.783842 | 1.943935 | -1.325010 |
| 6 | -5.787219 | 3.184650 | -2.265479 |
| 1 | -7.393325 | 2.332403 | -3.370092 |
| 6 | -8.526349 | 3.276790 | -1.145281 |
| 1 | -7.252561 | 1.723871 | -0.395711 |
| 1 | -8.491198 | 1.122342 | -1.485088 |
| 6 | -6.516986 | 4.520307 | -2.059423 |
| 1 | -5.178409 | 2.965663 | -1.384224 |
| 1 | -5.093300 | 3.251268 | -3.111311 |
| 6 | -7.538835 | 4.430846 | -0.916383 |
| 1 | -9.226892 | 3.201884 | -0.304509 |
| 1 | -9.130014 | 3.487497 | -2.039901 |
| 1 | -5.788689 | 5.315705 | -1.859119 |
| 1 | -7.037956 | 4.797376 | -2.987404 |
| 1 | -8.076720 | 5.380744 | -0.808209 |
| 1 | -7.003606 | 4.260742 | 0.029344 |
| 6 | 0.449120 | 1.144670 | 3.645590 |
| 6 | 0.448497 | -0.314068 | 3.127678 |
| 6 | 1.149502 | 1.223963 | 5.017882 |
| 1 | -0.598062 | 1.419461 | 3.812791 |
| 6 | -0.226312 | -1.254303 | 4.134225 |
| 1 | 1.487735 | -0.631269 | 2.965450 |
| 1 | -0.048185 | -0.363740 | 2.154008 |
| 6 | 0.487656 | 0.273964 | 6.028390 |
| 1 | 2.208332 | 0.950165 | 4.915309 |
| 1 | 1.125650 | 2.256198 | 5.387804 |
| 6 | 0.452682 | -1.171550 | 5.508544 |
| 1 | -0.219240 | -2.283601 | 3.760716 |
| 1 | -1.284524 | -0.974205 | 4.229493 |
| 1 | 1.014149 | 0.322989 | 6.989488 |
| 1 | -0.541237 | 0.612407 | 6.216371 |
| 1 | -0.058109 | -1.821911 | 6.229090 |
| 1 | 1.482592 | -1.548885 | 5.420614 |

---

**[1b-2b]‡**

---

Number of imaginary frequencies : 1

The smallest frequencies are : -279.1165 10.6050 12.7001 cm(-1)

Electronic energy : HF=-5577.5838725
Zero-point correction= 1.611575 (Hartree/Particle)
Thermal correction to Energy= 1.705111
Thermal correction to Enthalpy= 1.706055
Thermal correction to Gibbs Free Energy= 1.476134
Sum of electronic and zero-point Energies= -5575.972298
Sum of electronic and thermal Energies= -5575.878762
Sum of electronic and thermal Enthalpies= -5575.877817
Sum of electronic and thermal Free Energies= -5576.107738
............................................................
Cartesian Coordinates
............................................................

| | | | |
|---|---|---|---|
| 46 | 0.057438 | 1.507109 | -0.884776 |
| 6 | -1.879763 | 1.405578 | -1.616974 |
| 6 | -1.210204 | 0.314535 | -2.282811 |
| 15 | 0.178297 | 3.165180 | 0.750624 |
| 6 | -1.407850 | 3.799722 | 1.452431 |
| 6 | -2.187328 | 2.934775 | 2.242289 |
| 6 | -3.423053 | 3.344097 | 2.741745 |
| 6 | -3.898450 | 4.629299 | 2.467203 |
| 6 | -3.130532 | 5.498122 | 1.690351 |
| 6 | -1.896613 | 5.085483 | 1.179077 |
| 6 | 1.104690 | 2.680744 | 2.274444 |
| 6 | 0.829941 | 3.222945 | 3.541407 |
| 6 | 1.572848 | 2.826048 | 4.654790 |
| 6 | 2.597288 | 1.886737 | 4.516206 |
| 6 | 2.875769 | 1.344585 | 3.260446 |
| 6 | 2.133072 | 1.734084 | 2.144677 |
| 6 | 1.009291 | 4.710635 | 0.184844 |
| 6 | 1.128177 | 4.944147 | -1.192072 |
| 6 | 1.698503 | 6.128781 | -1.662387 |
| 6 | 2.161719 | 7.087595 | -0.760111 |
| 6 | 2.058315 | 6.857535 | 0.615368 |
| 6 | 1.483313 | 5.677330 | 1.086178 |
| 1 | -2.066833 | 2.297236 | -2.217058 |
| 1 | 0.030933 | 3.946799 | 3.665092 |
| 1 | 2.348956 | 1.306084 | 1.172359 |
| 1 | 1.348458 | 3.250845 | 5.629423 |
| 1 | 3.665001 | 0.610327 | 3.144995 |
| 1 | 3.174676 | 1.576732 | 5.382856 |
| 1 | -1.826595 | 1.934295 | 2.462547 |
| 1 | -1.317019 | 5.770936 | 0.570353 |
| 1 | -4.023576 | 2.653127 | 3.324688 |
| 1 | -3.492609 | 6.498849 | 1.471486 |
| 1 | -4.865452 | 4.943561 | 2.848395 |
| 1 | 0.778783 | 4.191002 | -1.892817 |
| 1 | 1.409707 | 5.506902 | 2.155643 |
| 1 | 1.790939 | 6.294840 | -2.731721 |
| 1 | 2.425836 | 7.597171 | 1.321083 |
| 1 | 2.609258 | 8.007831 | -1.125061 |
| 1 | -1.266506 | -0.680220 | -1.851336 |
| 6 | -0.773410 | 0.401656 | -3.628867 |
| 8 | 1.102128 | 0.707788 | -3.872015 |
| 6 | -2.913041 | 1.144071 | -0.545645 |
| 6 | -4.431234 | 1.217314 | -0.959287 |
| 1 | -2.710115 | 0.168969 | -0.101051 |
| 1 | -2.788788 | 1.879736 | 0.246249 |
| 6 | -4.774299 | -0.096529 | -1.693946 |
| 1 | -4.492618 | -0.917103 | -1.029726 |
| 1 | -4.180025 | -0.198104 | -2.604167 |

| | | | |
|---|---|---|---|
| 6 | -4.624651 | 2.470413 | -1.822118 |
| 6 | -4.285283 | 3.720552 | -1.274762 |
| 6 | -5.031238 | 2.426767 | -3.161165 |
| 6 | -4.341954 | 4.883717 | -2.037910 |
| 1 | -3.971367 | 3.782534 | -0.239564 |
| 6 | -5.090653 | 3.594949 | -3.930001 |
| 1 | -5.319136 | 1.486362 | -3.616332 |
| 6 | -4.743592 | 4.826175 | -3.376176 |
| 1 | -4.068705 | 5.833289 | -1.585840 |
| 1 | -5.411649 | 3.533992 | -4.966283 |
| 1 | -4.788005 | 5.730836 | -3.976093 |
| 6 | -5.268477 | 1.256810 | 0.339169 |
| 6 | -6.339811 | 2.142782 | 0.518846 |
| 6 | -4.993251 | 0.347103 | 1.375860 |
| 6 | -7.086828 | 2.143842 | 1.700546 |
| 1 | -6.588700 | 2.851456 | -0.262934 |
| 6 | -5.736500 | 0.344729 | 2.554468 |
| 1 | -4.190771 | -0.374782 | 1.271300 |
| 6 | -6.784857 | 1.251819 | 2.727527 |
| 1 | -7.909736 | 2.844636 | 1.810656 |
| 1 | -5.501195 | -0.374277 | 3.333716 |
| 1 | -7.368309 | 1.247448 | 3.642978 |
| 7 | -6.204653 | -0.182879 | -2.050127 |
| 1 | -6.810654 | 0.367934 | -1.443500 |
| 16 | -6.831253 | -1.722971 | -2.258179 |
| 8 | -8.205050 | -1.557481 | -2.744016 |
| 1 | 1.295102 | 1.419085 | -3.232657 |
| 1 | 1.532222 | -0.106912 | -3.419300 |
| 1 | -1.037867 | 1.285643 | -4.202313 |
| 1 | -0.698105 | -0.513857 | -4.206980 |
| 8 | -5.838265 | -2.460174 | -3.050973 |
| 6 | -6.867899 | -2.457509 | -0.606716 |
| 6 | -7.755263 | -1.977349 | 0.412616 |
| 6 | -5.948283 | -3.453401 | -0.354142 |
| 6 | -8.742369 | -0.971074 | 0.223575 |
| 6 | -7.626696 | -2.563670 | 1.719227 |
| 6 | -5.834798 | -4.014858 | 0.938195 |
| 1 | -5.307658 | -3.805944 | -1.151860 |
| 6 | -9.551478 | -0.570956 | 1.264516 |
| 1 | -8.889133 | -0.537228 | -0.757335 |
| 6 | -8.480513 | -2.125557 | 2.766260 |
| 6 | -6.650587 | -3.570924 | 1.950919 |
| 1 | -5.096392 | -4.789475 | 1.117189 |
| 6 | -9.424985 | -1.149843 | 2.547627 |
| 1 | -10.299849 | 0.197007 | 1.093110 |
| 1 | -8.372663 | -2.581217 | 3.747012 |
| 1 | -6.565331 | -3.991077 | 2.949600 |
| 1 | -10.074119 | -0.823946 | 3.354906 |
| 6 | 4.714685 | -0.874573 | 0.285528 |
| 6 | 5.770732 | 0.007966 | 0.400710 |
| 6 | 6.423592 | 0.144966 | 1.668358 |
| 6 | 7.473903 | 1.078060 | 1.883670 |
| 1 | 7.790257 | 1.710802 | 1.062226 |
| 6 | 8.075949 | 1.190333 | 3.117978 |
| 1 | 8.871579 | 1.914946 | 3.265779 |
| 6 | 7.666424 | 0.370717 | 4.197999 |
| 1 | 8.150245 | 0.471359 | 5.165297 |
| 6 | 6.660662 | -0.551509 | 4.019024 |
| 1 | 6.341910 | -1.189484 | 4.839552 |
| 6 | 6.018991 | -0.692092 | 2.757895 |
| 6 | 4.995476 | -1.649665 | 2.549423 |
| 1 | 4.710363 | -2.299483 | 3.371776 |

| | | | |
|---|---|---|---|
| 6 | 4.339533 | -1.765223 | 1.337211 |
| 6 | 3.347622 | -2.846186 | 1.120168 |
| 6 | 3.608138 | -4.125415 | 1.575308 |
| 1 | 4.561618 | -4.336823 | 2.050709 |
| 6 | 2.672621 | -5.177600 | 1.424423 |
| 6 | 2.946513 | -6.489648 | 1.898147 |
| 1 | 3.901023 | -6.679465 | 2.382554 |
| 6 | 2.023124 | -7.499572 | 1.749984 |
| 1 | 2.241849 | -8.498509 | 2.116170 |
| 6 | 0.783721 | -7.238051 | 1.116721 |
| 1 | 0.060319 | -8.040242 | 1.001295 |
| 6 | 0.489495 | -5.978152 | 0.642714 |
| 1 | -0.459735 | -5.785902 | 0.155101 |
| 6 | 1.421556 | -4.912888 | 0.778421 |
| 6 | 1.150633 | -3.592726 | 0.291790 |
| 6 | 2.104783 | -2.609008 | 0.459026 |
| 6 | 6.251725 | 0.765754 | -0.796128 |
| 6 | 5.822425 | 2.128757 | -0.945750 |
| 6 | 4.974642 | 2.764171 | 0.004424 |
| 1 | 4.617344 | 2.200574 | 0.856938 |
| 6 | 4.597543 | 4.079554 | -0.145300 |
| 1 | 3.947302 | 4.542442 | 0.588862 |
| 6 | 5.040121 | 4.832868 | -1.259189 |
| 1 | 4.729077 | 5.867141 | -1.363176 |
| 6 | 5.850945 | 4.248045 | -2.204565 |
| 1 | 6.195504 | 4.814221 | -3.066725 |
| 6 | 6.261888 | 2.894264 | -2.073128 |
| 6 | 7.109775 | 2.272551 | -3.023475 |
| 1 | 7.445215 | 2.842198 | -3.886742 |
| 6 | 7.512621 | 0.973502 | -2.848743 |
| 1 | 8.169954 | 0.517469 | -3.585038 |
| 6 | 7.108118 | 0.185267 | -1.730981 |
| 6 | -0.155287 | -3.301912 | -0.379102 |
| 6 | -0.271742 | -3.552018 | -1.786362 |
| 6 | 0.830219 | -3.970699 | -2.581856 |
| 1 | 1.799295 | -4.096509 | -2.114715 |
| 6 | 0.687193 | -4.179431 | -3.934848 |
| 1 | 1.546293 | -4.484021 | -4.525450 |
| 6 | -0.564217 | -3.982832 | -4.568845 |
| 1 | -0.659288 | -4.147838 | -5.638473 |
| 6 | -1.654884 | -3.583784 | -3.829005 |
| 1 | -2.623478 | -3.431816 | -4.298953 |
| 6 | -1.537455 | -3.363845 | -2.429227 |
| 6 | -2.644049 | -2.947553 | -1.647907 |
| 1 | -3.601370 | -2.806580 | -2.140175 |
| 6 | -2.497497 | -2.697910 | -0.303633 |
| 1 | -3.362162 | -2.380665 | 0.269442 |
| 6 | -1.249496 | -2.859546 | 0.359937 |
| 8 | 2.306065 | 0.899468 | -0.984931 |
| 8 | 2.163389 | -1.195282 | -2.529976 |
| 8 | 4.102799 | -1.011074 | -0.948951 |
| 8 | 1.801964 | -1.314743 | 0.074275 |
| 15 | 2.538817 | -0.581639 | -1.204975 |
| 6 | 7.643726 | -1.248835 | -1.726099 |
| 6 | 8.074745 | -1.860836 | -0.376930 |
| 6 | 6.693422 | -2.219090 | -2.471865 |
| 1 | 8.563103 | -1.206752 | -2.329168 |
| 6 | 8.750082 | -3.223988 | -0.592636 |
| 1 | 7.206507 | -2.015925 | 0.267990 |
| 1 | 8.743518 | -1.173755 | 0.153658 |
| 6 | 7.341380 | -3.598350 | -2.663146 |
| 1 | 5.763393 | -2.322199 | -1.905944 |

| | | | |
|---|---|---|---|
| 1 | 6.416603 | -1.789100 | -3.441632 |
| 6 | 7.809066 | -4.191396 | -1.326214 |
| 1 | 9.053154 | -3.644836 | 0.374072 |
| 1 | 9.669614 | -3.093848 | -1.181732 |
| 1 | 6.634017 | -4.276968 | -3.155353 |
| 1 | 8.206694 | -3.503380 | -3.335472 |
| 1 | 8.301292 | -5.158539 | -1.487471 |
| 1 | 6.930646 | -4.384266 | -0.693289 |
| 6 | -1.117089 | -2.504915 | 1.832537 |
| 6 | -0.897288 | -0.983419 | 2.024598 |
| 6 | -2.291823 | -2.978226 | 2.713048 |
| 1 | -0.217259 | -3.002331 | 2.212748 |
| 6 | -0.641235 | -0.639993 | 3.497349 |
| 1 | -1.793074 | -0.450809 | 1.678331 |
| 1 | -0.068105 | -0.644816 | 1.398853 |
| 6 | -2.049888 | -2.630417 | 4.190802 |
| 1 | -3.224916 | -2.498522 | 2.387668 |
| 1 | -2.436551 | -4.058911 | 2.592593 |
| 6 | -1.792723 | -1.128006 | 4.387457 |
| 1 | -0.489042 | 0.439125 | 3.614686 |
| 1 | 0.296202 | -1.116341 | 3.816669 |
| 1 | -2.905773 | -2.953992 | 4.796314 |
| 1 | -1.178536 | -3.193915 | 4.553045 |
| 1 | -1.582204 | -0.913892 | 5.442402 |
| 1 | -2.705096 | -0.569277 | 4.129885 |

---

**2b**

---

Number of imaginary frequencies : 0
The smallest frequencies are : 9.1508 14.7852 16.0671 cm(-1)

Electronic energy : HF=-5577.6044487
Zero-point correction= 1.611084 (Hartree/Particle)
Thermal correction to Energy= 1.706276
Thermal correction to Enthalpy= 1.707221
Thermal correction to Gibbs Free Energy= 1.473772
Sum of electronic and zero-point Energies= -5575.993365
Sum of electronic and thermal Energies= -5575.898172
Sum of electronic and thermal Enthalpies= -5575.897228
Sum of electronic and thermal Free Energies= -5576.130677

Cartesian Coordinates

| | | | |
|---|---|---|---|
| 46 | 0.509133 | -1.021709 | -0.804044 |
| 6 | 2.671063 | -0.798879 | -0.641823 |
| 6 | 2.082691 | 0.480289 | -0.884564 |
| 15 | 0.086404 | -3.198661 | 0.048416 |
| 6 | 1.445381 | -4.131197 | 0.874262 |
| 6 | 1.866952 | -3.704707 | 2.147224 |
| 6 | 2.919989 | -4.343205 | 2.799314 |
| 6 | 3.562754 | -5.425940 | 2.193785 |
| 6 | 3.143776 | -5.865846 | 0.938376 |
| 6 | 2.093989 | -5.221739 | 0.277570 |
| 6 | -1.271004 | -3.373856 | 1.281556 |
| 6 | -1.337255 | -4.471067 | 2.158794 |
| 6 | -2.385633 | -4.580777 | 3.072609 |
| 6 | -3.375748 | -3.596715 | 3.124570 |
| 6 | -3.311219 | -2.502836 | 2.261169 |
| 6 | -2.265484 | -2.387246 | 1.343903 |
| 6 | -0.377297 | -4.288128 | -1.361973 |
| 6 | -0.147880 | -3.843236 | -2.671833 |

| | | | |
|---|---|---|---|
| 6 | -0.419147 | -4.677179 | -3.758365 |
| 6 | -0.919098 | -5.962096 | -3.545059 |
| 6 | -1.163693 | -6.408783 | -2.242497 |
| 6 | -0.896642 | -5.576572 | -1.156328 |
| 1 | 3.039035 | -1.339452 | -1.510059 |
| 1 | -0.564369 | -5.232390 | 2.144542 |
| 1 | -2.222649 | -1.539160 | 0.672937 |
| 1 | -2.424245 | -5.432927 | 3.745434 |
| 1 | -4.070068 | -1.731041 | 2.299476 |
| 1 | -4.192269 | -3.679532 | 3.836418 |
| 1 | 1.370584 | -2.868570 | 2.628307 |
| 1 | 1.788873 | -5.569457 | -0.702957 |
| 1 | 3.252266 | -3.981326 | 3.767217 |
| 1 | 3.640620 | -6.705195 | 0.460663 |
| 1 | 4.391665 | -5.914979 | 2.696182 |
| 1 | 0.236413 | -2.841815 | -2.847064 |
| 1 | -1.090432 | -5.930877 | -0.148924 |
| 1 | -0.246262 | -4.315666 | -4.767418 |
| 1 | -1.563350 | -7.404535 | -2.073035 |
| 1 | -1.128217 | -6.611899 | -4.390130 |
| 1 | 2.002530 | 1.195431 | -0.069071 |
| 6 | 1.354708 | 0.696186 | -2.043865 |
| 8 | -1.003875 | -0.204096 | -3.977127 |
| 6 | 3.363935 | -1.067943 | 0.672458 |
| 6 | 4.935977 | -1.134508 | 0.704925 |
| 1 | 3.039229 | -0.304586 | 1.382134 |
| 1 | 3.012991 | -2.014326 | 1.078398 |
| 6 | 5.537509 | 0.305007 | 0.581698 |
| 1 | 6.627802 | 0.270439 | 0.577521 |
| 1 | 5.254757 | 0.866265 | 1.475123 |
| 6 | 5.490832 | -2.104945 | -0.350610 |
| 6 | 4.724567 | -3.196565 | -0.784473 |
| 6 | 6.803763 | -2.005860 | -0.833123 |
| 6 | 5.228530 | -4.127168 | -1.692265 |
| 1 | 3.723039 | -3.343988 | -0.406055 |
| 6 | 7.312909 | -2.931689 | -1.745182 |
| 1 | 7.445901 | -1.191511 | -0.524748 |
| 6 | 6.526902 | -3.995802 | -2.185683 |
| 1 | 4.601333 | -4.956680 | -2.007663 |
| 1 | 8.328002 | -2.811895 | -2.112883 |
| 1 | 6.921181 | -4.713346 | -2.899784 |
| 6 | 5.303287 | -1.643152 | 2.128643 |
| 6 | 6.129139 | -2.754983 | 2.343164 |
| 6 | 4.767836 | -1.007569 | 3.264138 |
| 6 | 6.403046 | -3.216367 | 3.633725 |
| 1 | 6.555494 | -3.280980 | 1.498281 |
| 6 | 5.034125 | -1.466470 | 4.553696 |
| 1 | 4.118045 | -0.147450 | 3.150126 |
| 6 | 5.854760 | -2.580162 | 4.746677 |
| 1 | 7.045429 | -4.083301 | 3.762526 |
| 1 | 4.599041 | -0.951703 | 5.406177 |
| 1 | 6.064565 | -2.942269 | 5.749095 |
| 7 | 5.113107 | 1.159764 | -0.534903 |
| 1 | 4.125286 | 1.391037 | -0.529894 |
| 16 | 5.647509 | 0.904569 | -2.109812 |
| 8 | 7.114918 | 0.835258 | -2.027691 |
| 1 | -1.373793 | -0.719279 | -3.243122 |
| 1 | -1.034281 | 0.688919 | -3.599570 |
| 1 | 1.525931 | 0.103693 | -2.938447 |
| 1 | 0.743822 | 1.584844 | -2.146578 |
| 8 | 4.925097 | -0.174674 | -2.802560 |
| 6 | 5.120337 | 2.456218 | -2.855806 |

| | | | |
|---|---|---|---|
| 6 | 5.669991 | 3.713960 | -2.449725 |
| 6 | 4.178365 | 2.361009 | -3.859037 |
| 6 | 6.622335 | 3.881564 | -1.406687 |
| 6 | 5.190981 | 4.884066 | -3.132510 |
| 6 | 3.727488 | 3.522335 | -4.524688 |
| 1 | 3.789679 | 1.387770 | -4.134074 |
| 6 | 7.056185 | 5.139364 | -1.047872 |
| 1 | 7.010160 | 3.011666 | -0.894769 |
| 6 | 5.667920 | 6.163232 | -2.737654 |
| 6 | 4.227438 | 4.752311 | -4.169048 |
| 1 | 2.984274 | 3.431558 | -5.310138 |
| 6 | 6.575982 | 6.292490 | -1.712332 |
| 1 | 7.780123 | 5.246104 | -0.245198 |
| 1 | 5.293599 | 7.040364 | -3.258741 |
| 1 | 3.882913 | 5.650944 | -4.673910 |
| 1 | 6.928147 | 7.275116 | -1.412957 |
| 6 | -4.463932 | 0.612770 | 0.037747 |
| 6 | -5.465180 | -0.219825 | -0.422986 |
| 6 | -6.392385 | -0.775537 | 0.514604 |
| 6 | -7.405227 | -1.693745 | 0.126653 |
| 1 | -7.476601 | -1.985555 | -0.915108 |
| 6 | -8.276527 | -2.216582 | 1.057191 |
| 1 | -9.039295 | -2.923714 | 0.744077 |
| 6 | -8.187192 | -1.841631 | 2.420277 |
| 1 | -8.881203 | -2.262628 | 3.142082 |
| 6 | -7.226243 | -0.943628 | 2.825772 |
| 1 | -7.152459 | -0.644607 | 3.868374 |
| 6 | -6.312047 | -0.385588 | 1.890378 |
| 6 | -5.326594 | 0.553228 | 2.284783 |
| 1 | -5.287089 | 0.863122 | 3.324967 |
| 6 | -4.404394 | 1.067785 | 1.389594 |
| 6 | -3.458982 | 2.126745 | 1.823393 |
| 6 | -3.909884 | 3.151593 | 2.634006 |
| 1 | -4.966200 | 3.208149 | 2.880261 |
| 6 | -3.036551 | 4.139244 | 3.148819 |
| 6 | -3.512420 | 5.187863 | 3.982115 |
| 1 | -4.574790 | 5.230682 | 4.208430 |
| 6 | -2.647738 | 6.127153 | 4.495976 |
| 1 | -3.020597 | 6.924287 | 5.132606 |
| 6 | -1.266367 | 6.054403 | 4.194434 |
| 1 | -0.587940 | 6.798133 | 4.602750 |
| 6 | -0.774551 | 5.052605 | 3.386066 |
| 1 | 0.284669 | 5.010150 | 3.159688 |
| 6 | -1.641127 | 4.068893 | 2.833980 |
| 6 | -1.167080 | 3.018687 | 1.978589 |
| 6 | -2.073261 | 2.107370 | 1.471630 |
| 6 | -5.594441 | -0.479617 | -1.890569 |
| 6 | -5.036914 | -1.698198 | -2.406892 |
| 6 | -4.411035 | -2.658779 | -1.564471 |
| 1 | -4.328355 | -2.460667 | -0.503277 |
| 6 | -3.908501 | -3.833409 | -2.076747 |
| 1 | -3.435755 | -4.550971 | -1.415219 |
| 6 | -3.991370 | -4.108783 | -3.462537 |
| 1 | -3.582639 | -5.035877 | -3.850115 |
| 6 | -4.577144 | -3.195792 | -4.309073 |
| 1 | -4.641821 | -3.391510 | -5.376661 |
| 6 | -5.114385 | -1.980016 | -3.808265 |
| 6 | -5.742514 | -1.032345 | -4.654419 |
| 1 | -5.800322 | -1.231756 | -5.721596 |
| 6 | -6.280681 | 0.114886 | -4.131041 |
| 1 | -6.765175 | 0.826044 | -4.795609 |
| 6 | -6.232029 | 0.423945 | -2.739405 |

| | | | |
|---|---|---|---|
| 6 | 0.304090 | 2.932623 | 1.703137 |
| 6 | 0.855323 | 3.722273 | 0.641043 |
| 6 | 0.035486 | 4.418792 | -0.289761 |
| 1 | -1.040321 | 4.316798 | -0.223138 |
| 6 | 0.595490 | 5.178418 | -1.292270 |
| 1 | -0.048485 | 5.687923 | -2.003112 |
| 6 | 2.001705 | 5.293130 | -1.411818 |
| 1 | 2.430843 | 5.897010 | -2.204803 |
| 6 | 2.825859 | 4.630175 | -0.531717 |
| 1 | 3.904814 | 4.709064 | -0.616189 |
| 6 | 2.279133 | 3.826388 | 0.503951 |
| 6 | 3.104570 | 3.116060 | 1.413626 |
| 1 | 4.184183 | 3.202471 | 1.319466 |
| 6 | 2.546554 | 2.323764 | 2.390252 |
| 1 | 3.201303 | 1.800088 | 3.077389 |
| 6 | 1.137246 | 2.213423 | 2.559084 |
| 8 | -1.657958 | -0.552417 | -1.185187 |
| 8 | -1.413541 | 2.000543 | -1.773542 |
| 8 | -3.602701 | 1.162589 | -0.889704 |
| 8 | -1.607407 | 1.063600 | 0.695181 |
| 15 | -1.979807 | 0.921870 | -0.913242 |
| 6 | -6.854951 | 1.769306 | -2.357661 |
| 6 | -7.623711 | 1.865972 | -1.023771 |
| 6 | -5.825338 | 2.922329 | -2.464353 |
| 1 | -7.606062 | 1.962889 | -3.138252 |
| 6 | -8.317514 | 3.230799 | -0.895431 |
| 1 | -6.938692 | 1.757801 | -0.179452 |
| 1 | -8.350990 | 1.050005 | -0.945806 |
| 6 | -6.499293 | 4.292451 | -2.302105 |
| 1 | -5.057503 | 2.794025 | -1.696199 |
| 1 | -5.306837 | 2.862970 | -3.428359 |
| 6 | -7.298035 | 4.375531 | -0.993344 |
| 1 | -8.860026 | 3.282786 | 0.056688 |
| 1 | -9.067885 | 3.342902 | -1.691603 |
| 1 | -5.742698 | 5.085790 | -2.338639 |
| 1 | -7.179180 | 4.466788 | -3.149032 |
| 1 | -7.801743 | 5.346856 | -0.912413 |
| 1 | -6.602029 | 4.307726 | -0.144288 |
| 6 | 0.561532 | 1.305829 | 3.635591 |
| 6 | 0.368016 | -0.135030 | 3.099790 |
| 6 | 1.366381 | 1.270927 | 4.949834 |
| 1 | -0.436697 | 1.683578 | 3.885918 |
| 6 | -0.321834 | -1.026598 | 4.140523 |
| 1 | 1.354497 | -0.551462 | 2.853660 |
| 1 | -0.207508 | -0.110495 | 2.171512 |
| 6 | 0.676439 | 0.381395 | 5.995543 |
| 1 | 2.371105 | 0.867606 | 4.763374 |
| 1 | 1.500197 | 2.288687 | 5.335632 |
| 6 | 0.456487 | -1.042917 | 5.463093 |
| 1 | -0.444470 | -2.042978 | 3.750634 |
| 1 | -1.336986 | -0.644623 | 4.316704 |
| 1 | 1.270238 | 0.358193 | 6.917585 |
| 1 | -0.295264 | 0.822748 | 6.258656 |
| 1 | -0.066787 | -1.652560 | 6.209683 |
| 1 | 1.434829 | -1.518768 | 5.297587 |

---

**1c**

---

Number of imaginary frequencies : 0
The smallest frequencies are : 6.6730 14.4410 14.6791 cm(-1)

Electronic energy : HF=-5577.5854032
Zero-point correction= 1.613035 (Hartree/Particle)
Thermal correction to Energy= 1.706992
Thermal correction to Enthalpy= 1.707936
Thermal correction to Gibbs Free Energy= 1.475948
Sum of electronic and zero-point Energies= -5575.972368
Sum of electronic and thermal Energies= -5575.878411
Sum of electronic and thermal Enthalpies= -5575.877467
Sum of electronic and thermal Free Energies= -5576.109455

............................................................

Cartesian Coordinates

............................................................

| | | | |
|---|---|---|---|
| 46 | -3.620467 | -0.270326 | -1.903117 |
| 6 | -2.720692 | -2.189764 | -1.300957 |
| 6 | -1.743531 | -1.382537 | -1.874502 |
| 15 | -5.560308 | 0.796970 | -1.262349 |
| 6 | -7.151903 | 0.305169 | -2.055402 |
| 6 | -7.271711 | -1.023001 | -2.495203 |
| 6 | -8.458868 | -1.472568 | -3.073613 |
| 6 | -9.535269 | -0.596138 | -3.233424 |
| 6 | -9.421121 | 0.729231 | -2.808156 |
| 6 | -8.237359 | 1.178748 | -2.218927 |
| 6 | -5.875428 | 0.532014 | 0.540418 |
| 6 | -7.148013 | 0.277670 | 1.072715 |
| 6 | -7.306427 | 0.055827 | 2.443683 |
| 6 | -6.200156 | 0.085305 | 3.294905 |
| 6 | -4.927716 | 0.342789 | 2.775177 |
| 6 | -4.769264 | 0.563657 | 1.406605 |
| 6 | -5.553284 | 2.635341 | -1.427348 |
| 6 | -4.974637 | 3.183678 | -2.582908 |
| 6 | -4.947545 | 4.565326 | -2.775284 |
| 6 | -5.483439 | 5.417724 | -1.805653 |
| 6 | -6.051686 | 4.881552 | -0.647712 |
| 6 | -6.090201 | 3.497725 | -0.459338 |
| 1 | -3.121430 | -2.992982 | -1.920054 |
| 1 | -8.015842 | 0.246258 | 0.421602 |
| 1 | -3.777162 | 0.756204 | 1.009817 |
| 1 | -8.296779 | -0.145431 | 2.843142 |
| 1 | -4.052363 | 0.335461 | 3.417843 |
| 1 | -6.325621 | -0.098782 | 4.358376 |
| 1 | -6.425966 | -1.697385 | -2.385094 |
| 1 | -8.160558 | 2.209832 | -1.887871 |
| 1 | -8.539717 | -2.503125 | -3.408055 |
| 1 | -10.254069 | 1.415614 | -2.933622 |
| 1 | -10.456971 | -0.943193 | -3.692096 |
| 1 | -4.538988 | 2.523414 | -3.329144 |
| 1 | -6.531802 | 3.089880 | 0.444771 |
| 1 | -4.495782 | 4.976344 | -3.673732 |
| 1 | -6.464753 | 5.539671 | 0.111625 |
| 1 | -5.452152 | 6.494035 | -1.949094 |
| 1 | -1.124859 | -0.749512 | -1.242197 |
| 6 | -1.168022 | -1.677610 | -3.227806 |
| 8 | 0.167545 | -2.246934 | -3.010662 |
| 6 | -2.995562 | -2.347470 | 0.178705 |
| 6 | -2.046352 | -3.356097 | 0.915822 |
| 1 | -2.926020 | -1.390264 | 0.695319 |
| 1 | -4.026029 | -2.695401 | 0.291692 |
| 6 | -0.678363 | -2.650226 | 1.195488 |
| 1 | -0.179918 | -2.390024 | 0.260658 |
| 1 | -0.027140 | -3.327575 | 1.753990 |
| 6 | -1.884446 | -4.584474 | 0.005884 |
| 6 | -2.967184 | -5.475183 | -0.110476 |

| | | | |
|---|---|---|---|
| 6 | -0.766589 | -4.804693 | -0.809617 |
| 6 | -2.938812 | -6.543234 | -1.003597 |
| 1 | -3.842643 | -5.324219 | 0.513532 |
| 6 | -0.735922 | -5.878051 | -1.706215 |
| 1 | 0.084301 | -4.135114 | -0.786322 |
| 6 | -1.816086 | -6.751589 | -1.810113 |
| 1 | -3.792211 | -7.212929 | -1.069084 |
| 1 | 0.139764 | -6.018321 | -2.330867 |
| 1 | -1.787195 | -7.583156 | -2.508993 |
| 6 | -2.636739 | -3.769967 | 2.275329 |
| 6 | -2.132391 | -4.905412 | 2.930070 |
| 6 | -3.616683 | -3.016107 | 2.929523 |
| 6 | -2.600883 | -5.283061 | 4.184979 |
| 1 | -1.362425 | -5.500112 | 2.447130 |
| 6 | -4.089334 | -3.391343 | 4.190449 |
| 1 | -4.016529 | -2.119279 | 2.476120 |
| 6 | -3.588221 | -4.526360 | 4.822833 |
| 1 | -2.192678 | -6.166771 | 4.668358 |
| 1 | -4.848371 | -2.782959 | 4.674689 |
| 1 | -3.954910 | -4.817758 | 5.803417 |
| 7 | -0.840755 | -1.386296 | 1.917428 |
| 1 | -0.640180 | -0.544395 | 1.381781 |
| 16 | -0.653649 | -1.206456 | 3.554467 |
| 8 | -1.779879 | -0.401517 | 4.059631 |
| 1 | 0.493511 | -2.663565 | -3.826545 |
| 1 | 1.042074 | -1.069801 | -2.702105 |
| 1 | -1.781901 | -2.403101 | -3.771545 |
| 1 | -1.056611 | -0.774893 | -3.841666 |
| 8 | -0.397517 | -2.533409 | 4.130086 |
| 6 | 0.847495 | -0.205288 | 3.731820 |
| 6 | 1.070940 | 0.638774 | 4.870608 |
| 6 | 1.802404 | -0.347884 | 2.749825 |
| 6 | 0.164041 | 0.807461 | 5.955707 |
| 6 | 2.302684 | 1.382860 | 4.901306 |
| 6 | 3.020885 | 0.359101 | 2.818510 |
| 1 | 1.611813 | -0.973890 | 1.890371 |
| 6 | 0.450149 | 1.674163 | 6.988168 |
| 1 | -0.767715 | 0.258770 | 5.960508 |
| 6 | 2.554258 | 2.277522 | 5.976204 |
| 6 | 3.258177 | 1.217150 | 3.864054 |
| 1 | 3.746763 | 0.233108 | 2.026828 |
| 6 | 1.648285 | 2.425294 | 6.999956 |
| 1 | -0.260038 | 1.784111 | 7.802747 |
| 1 | 3.480582 | 2.845813 | 5.967001 |
| 1 | 4.177508 | 1.792407 | 3.906318 |
| 1 | 1.849405 | 3.112529 | 7.816654 |
| 6 | 4.289824 | -0.407831 | -0.905882 |
| 6 | 4.887501 | -1.490257 | -1.518265 |
| 6 | 6.244981 | -1.366848 | -1.963166 |
| 6 | 6.927857 | -2.429356 | -2.614872 |
| 1 | 6.406322 | -3.364691 | -2.783038 |
| 6 | 8.234162 | -2.280550 | -3.027059 |
| 1 | 8.739623 | -3.103365 | -3.524252 |
| 6 | 8.925076 | -1.063864 | -2.809464 |
| 1 | 9.954248 | -0.961225 | -3.140837 |
| 6 | 8.292793 | -0.016663 | -2.179126 |
| 1 | 8.814544 | 0.920914 | -2.005825 |
| 6 | 6.946486 | -0.138880 | -1.738865 |
| 6 | 6.283459 | 0.928026 | -1.085221 |
| 1 | 6.817070 | 1.861783 | -0.933746 |
| 6 | 4.972977 | 0.823982 | -0.658489 |
| 6 | 4.326802 | 1.951509 | 0.058971 |

| | | | |
|---|---|---|---|
| 6 | 4.998847 | 2.651548 | 1.044235 |
| 1 | 6.005860 | 2.351741 | 1.319803 |
| 6 | 4.390356 | 3.726331 | 1.738008 |
| 6 | 5.073673 | 4.413846 | 2.779165 |
| 1 | 6.087645 | 4.109577 | 3.026628 |
| 6 | 4.464298 | 5.440750 | 3.463523 |
| 1 | 4.993252 | 5.955588 | 4.260369 |
| 6 | 3.145826 | 5.833869 | 3.126637 |
| 1 | 2.672962 | 6.646973 | 3.669531 |
| 6 | 2.458447 | 5.193063 | 2.119746 |
| 1 | 1.448004 | 5.495204 | 1.869186 |
| 6 | 3.050760 | 4.116164 | 1.405892 |
| 6 | 2.350936 | 3.406891 | 0.377787 |
| 6 | 2.995895 | 2.356266 | -0.241007 |
| 6 | 4.139225 | -2.766052 | -1.763046 |
| 6 | 3.520768 | -2.915912 | -3.055648 |
| 6 | 3.542838 | -1.880766 | -4.033553 |
| 1 | 4.044803 | -0.949112 | -3.804267 |
| 6 | 2.908556 | -2.028169 | -5.247385 |
| 1 | 2.927208 | -1.214738 | -5.966442 |
| 6 | 2.222062 | -3.227317 | -5.561131 |
| 1 | 1.725308 | -3.332927 | -6.521337 |
| 6 | 2.199797 | -4.261437 | -4.648088 |
| 1 | 1.685579 | -5.191167 | -4.878134 |
| 6 | 2.843608 | -4.133394 | -3.386668 |
| 6 | 2.824070 | -5.179462 | -2.430883 |
| 1 | 2.343401 | -6.120450 | -2.682699 |
| 6 | 3.408808 | -5.005633 | -1.204172 |
| 1 | 3.369665 | -5.813476 | -0.478280 |
| 6 | 4.079235 | -3.801680 | -0.832543 |
| 6 | 0.978543 | 3.800652 | -0.073763 |
| 6 | -0.153343 | 3.475195 | 0.746636 |
| 6 | -0.032559 | 2.799189 | 1.993659 |
| 1 | 0.945593 | 2.482417 | 2.336465 |
| 6 | -1.141986 | 2.540332 | 2.768632 |
| 1 | -1.034870 | 2.021504 | 3.712891 |
| 6 | -2.431737 | 2.927968 | 2.334180 |
| 1 | -3.295616 | 2.710063 | 2.953344 |
| 6 | -2.590033 | 3.558329 | 1.121625 |
| 1 | -3.576676 | 3.844551 | 0.767838 |
| 6 | -1.465097 | 3.847880 | 0.304011 |
| 6 | -1.608476 | 4.508493 | -0.943099 |
| 1 | -2.604718 | 4.779936 | -1.280449 |
| 6 | -0.506943 | 4.803187 | -1.706758 |
| 1 | -0.637278 | 5.314998 | -2.655813 |
| 6 | 0.808646 | 4.460849 | -1.288041 |
| 8 | 1.555526 | -0.191289 | -2.548270 |
| 8 | 0.576640 | 0.129282 | -0.132051 |
| 8 | 2.993855 | -0.562809 | -0.409224 |
| 8 | 2.350544 | 1.717555 | -1.302105 |
| 15 | 1.729810 | 0.245173 | -1.056249 |
| 6 | 4.586328 | -3.776389 | 0.608058 |
| 6 | 5.919570 | -3.066489 | 0.912948 |
| 6 | 3.474246 | -3.242717 | 1.543842 |
| 1 | 4.743933 | -4.829860 | 0.880660 |
| 6 | 6.304676 | -3.263098 | 2.387773 |
| 1 | 5.832014 | -1.993507 | 0.723665 |
| 1 | 6.708102 | -3.439388 | 0.249825 |
| 6 | 3.852199 | -3.378990 | 3.024533 |
| 1 | 3.299067 | -2.193260 | 1.295070 |
| 1 | 2.534965 | -3.772153 | 1.342227 |
| 6 | 5.209549 | -2.727156 | 3.322701 |

| | | | |
|---|---|---|---|
| 1 | 7.258680 | -2.761289 | 2.590953 |
| 1 | 6.463854 | -4.332633 | 2.587707 |
| 1 | 3.067094 | -2.935077 | 3.647198 |
| 1 | 3.897802 | -4.445533 | 3.287215 |
| 1 | 5.487028 | -2.889963 | 4.371159 |
| 1 | 5.120676 | -1.640343 | 3.187385 |
| 6 | 1.976408 | 4.844214 | -2.180219 |
| 6 | 1.906152 | 4.168290 | -3.568071 |
| 6 | 2.113354 | 6.376518 | -2.329199 |
| 1 | 2.900596 | 4.496440 | -1.708178 |
| 6 | 3.126143 | 4.537651 | -4.424191 |
| 1 | 0.992101 | 4.488337 | -4.086854 |
| 1 | 1.837948 | 3.083575 | -3.439033 |
| 6 | 3.329747 | 6.744519 | -3.191526 |
| 1 | 1.203295 | 6.783951 | -2.790306 |
| 1 | 2.194378 | 6.834800 | -1.336007 |
| 6 | 3.273043 | 6.060124 | -4.565368 |
| 1 | 3.048795 | 4.068021 | -5.412560 |
| 1 | 4.031590 | 4.128356 | -3.953075 |
| 1 | 3.393278 | 7.833360 | -3.308806 |
| 1 | 4.245670 | 6.430732 | -2.670312 |
| 1 | 4.167963 | 6.305790 | -5.150419 |
| 1 | 2.412728 | 6.452726 | -5.126716 |

---

**[1c-2c]‡**

---

Number of imaginary frequencies : 1
The smallest frequencies are : -249.8466 7.6185 12.0562 cm(-1)

Electronic energy : HF=-5577.5673186
Zero-point correction= 1.611170 (Hartree/Particle)
Thermal correction to Energy= 1.704896
Thermal correction to Enthalpy= 1.705841
Thermal correction to Gibbs Free Energy= 1.474395
Sum of electronic and zero-point Energies= -5575.956149
Sum of electronic and thermal Energies= -5575.862422
Sum of electronic and thermal Enthalpies= -5575.861478
Sum of electronic and thermal Free Energies= -5576.092924

Cartesian Coordinates

| | | | |
|---|---|---|---|
| 46 | -3.443865 | 0.006171 | -1.920699 |
| 6 | -2.635421 | -1.895881 | -1.352179 |
| 6 | -1.577416 | -1.105484 | -1.869491 |
| 15 | -5.526961 | 0.654914 | -1.116859 |
| 6 | -7.004372 | -0.055610 | -1.948812 |
| 6 | -6.917729 | -1.373399 | -2.425989 |
| 6 | -8.018912 | -1.978013 | -3.032075 |
| 6 | -9.213186 | -1.267963 | -3.179758 |
| 6 | -9.304285 | 0.045872 | -2.714786 |
| 6 | -8.206983 | 0.650935 | -2.098665 |
| 6 | -5.776214 | 0.269248 | 0.664646 |
| 6 | -6.998266 | -0.182953 | 1.184373 |
| 6 | -7.122311 | -0.450243 | 2.550026 |
| 6 | -6.032797 | -0.269091 | 3.404756 |
| 6 | -4.810979 | 0.179795 | 2.894623 |
| 6 | -4.684720 | 0.444796 | 1.530706 |
| 6 | -5.772939 | 2.477984 | -1.216976 |
| 6 | -5.343453 | 3.132185 | -2.383537 |
| 6 | -5.477003 | 4.514852 | -2.509730 |
| 6 | -6.027597 | 5.262030 | -1.464459 |

| | | | |
|---|---|---|---|
| 6 | -6.452890 | 4.619824 | -0.299752 |
| 6 | -6.330352 | 3.233639 | -0.175192 |
| 1 | -3.064371 | -2.635282 | -2.029853 |
| 1 | -7.851453 | -0.328247 | 0.529447 |
| 1 | -3.730165 | 0.785263 | 1.142486 |
| 1 | -8.071445 | -0.803959 | 2.942972 |
| 1 | -3.942422 | 0.279107 | 3.536979 |
| 1 | -6.129990 | -0.487442 | 4.464496 |
| 1 | -5.984073 | -1.920236 | -2.323071 |
| 1 | -8.287588 | 1.671074 | -1.735830 |
| 1 | -7.941725 | -2.998582 | -3.396097 |
| 1 | -10.230511 | 0.601722 | -2.830106 |
| 1 | -10.068341 | -1.735604 | -3.659332 |
| 1 | -4.896868 | 2.557252 | -3.191918 |
| 1 | -6.660118 | 2.743188 | 0.735088 |
| 1 | -5.138832 | 5.009709 | -3.415623 |
| 1 | -6.878839 | 5.196688 | 0.516112 |
| 1 | -6.119151 | 6.340370 | -1.556016 |
| 1 | -0.930074 | -0.539764 | -1.204985 |
| 6 | -1.236980 | -1.145713 | -3.244582 |
| 8 | 0.327858 | -2.299065 | -3.311255 |
| 6 | -2.861589 | -2.182200 | 0.111390 |
| 6 | -1.884176 | -3.233455 | 0.744719 |
| 1 | -2.782970 | -1.268983 | 0.700979 |
| 1 | -3.884558 | -2.553031 | 0.216584 |
| 6 | -0.518052 | -2.539118 | 1.067003 |
| 1 | -0.004364 | -2.245667 | 0.151375 |
| 1 | 0.122461 | -3.242591 | 1.603390 |
| 6 | -1.724991 | -4.384666 | -0.263459 |
| 6 | -2.816241 | -5.251445 | -0.460846 |
| 6 | -0.598330 | -4.554941 | -1.076690 |
| 6 | -2.787037 | -6.245309 | -1.435402 |
| 1 | -3.696111 | -5.142567 | 0.166301 |
| 6 | -0.567498 | -5.552254 | -2.057265 |
| 1 | 0.262174 | -3.904687 | -0.988895 |
| 6 | -1.655943 | -6.400689 | -2.243545 |
| 1 | -3.645082 | -6.900101 | -1.562725 |
| 1 | 0.314658 | -5.645885 | -2.681331 |
| 1 | -1.627859 | -7.173856 | -3.006610 |
| 6 | -2.454530 | -3.769385 | 2.070153 |
| 6 | -1.906170 | -4.936333 | 2.627230 |
| 6 | -3.460931 | -3.111259 | 2.783287 |
| 6 | -2.357833 | -5.434757 | 3.845212 |
| 1 | -1.116784 | -5.460354 | 2.096148 |
| 6 | -3.917581 | -3.608534 | 4.007440 |
| 1 | -3.897287 | -2.195814 | 2.407920 |
| 6 | -3.372690 | -4.772721 | 4.542705 |
| 1 | -1.915873 | -6.339883 | 4.253248 |
| 1 | -4.698595 | -3.071268 | 4.538613 |
| 1 | -3.726397 | -5.159821 | 5.494481 |
| 7 | -0.678776 | -1.299745 | 1.828450 |
| 1 | -0.412315 | -0.452400 | 1.322370 |
| 16 | -0.543240 | -1.201848 | 3.478369 |
| 8 | -1.715333 | -0.474153 | 3.997815 |
| 1 | 0.667200 | -2.464698 | -4.207806 |
| 1 | 0.954126 | -1.591806 | -2.919424 |
| 1 | -1.845632 | -1.730623 | -3.928028 |
| 1 | -0.694984 | -0.309274 | -3.670583 |
| 8 | -0.253478 | -2.549402 | 3.992846 |
| 6 | 0.899551 | -0.147850 | 3.771136 |
| 6 | 1.065589 | 0.551949 | 5.014105 |
| 6 | 1.861508 | -0.097628 | 2.788304 |

| | | | |
|---|---|---|---|
| 6 | 0.145654 | 0.519319 | 6.100394 |
| 6 | 2.250726 | 1.355579 | 5.159186 |
| 6 | 3.028080 | 0.677696 | 2.960328 |
| 1 | 1.719386 | -0.608897 | 1.848527 |
| 6 | 0.380506 | 1.243965 | 7.248970 |
| 1 | -0.755613 | -0.072336 | 6.017526 |
| 6 | 2.451542 | 2.095848 | 6.355103 |
| 6 | 3.210437 | 1.400978 | 4.112649 |
| 1 | 3.754994 | 0.708906 | 2.160953 |
| 6 | 1.538009 | 2.044934 | 7.381818 |
| 1 | -0.337837 | 1.199335 | 8.062644 |
| 1 | 3.347777 | 2.704836 | 6.439779 |
| 1 | 4.089473 | 2.026695 | 4.237555 |
| 1 | 1.702284 | 2.614436 | 8.292021 |
| 6 | 4.338483 | -0.256092 | -0.776623 |
| 6 | 4.986825 | -1.338488 | -1.339665 |
| 6 | 6.366552 | -1.208342 | -1.704974 |
| 6 | 7.104309 | -2.279580 | -2.279544 |
| 1 | 6.606435 | -3.227442 | -2.450015 |
| 6 | 8.432226 | -2.125277 | -2.613139 |
| 1 | 8.978481 | -2.956572 | -3.049718 |
| 6 | 9.092409 | -0.891935 | -2.393492 |
| 1 | 10.139280 | -0.784234 | -2.662343 |
| 6 | 8.406621 | 0.165241 | -1.840169 |
| 1 | 8.903467 | 1.116486 | -1.666807 |
| 6 | 7.036876 | 0.037527 | -1.480885 |
| 6 | 6.316601 | 1.118015 | -0.916160 |
| 1 | 6.822448 | 2.069747 | -0.780909 |
| 6 | 4.987126 | 1.002986 | -0.556821 |
| 6 | 4.274746 | 2.149154 | 0.063077 |
| 6 | 4.876971 | 2.931305 | 1.031198 |
| 1 | 5.883066 | 2.692072 | 1.364285 |
| 6 | 4.196937 | 4.016184 | 1.637756 |
| 6 | 4.817728 | 4.802679 | 2.647374 |
| 1 | 5.836830 | 4.561853 | 2.940025 |
| 6 | 4.146671 | 5.846417 | 3.242373 |
| 1 | 4.629877 | 6.438234 | 4.014500 |
| 6 | 2.823248 | 6.154923 | 2.843545 |
| 1 | 2.300770 | 6.984005 | 3.312134 |
| 6 | 2.192484 | 5.412429 | 1.869844 |
| 1 | 1.178045 | 5.652926 | 1.572861 |
| 6 | 2.849826 | 4.316942 | 1.245382 |
| 6 | 2.214190 | 3.506710 | 0.249140 |
| 6 | 2.938804 | 2.473229 | -0.314606 |
| 6 | 4.263527 | -2.617396 | -1.642767 |
| 6 | 3.767745 | -2.762440 | -2.990265 |
| 6 | 3.845846 | -1.709153 | -3.945311 |
| 1 | 4.281103 | -0.762405 | -3.652443 |
| 6 | 3.345736 | -1.858635 | -5.220009 |
| 1 | 3.407656 | -1.031666 | -5.921370 |
| 6 | 2.741479 | -3.075897 | -5.620514 |
| 1 | 2.357642 | -3.185307 | -6.631185 |
| 6 | 2.653625 | -4.120593 | -4.724638 |
| 1 | 2.194304 | -5.062193 | -5.015431 |
| 6 | 3.155992 | -3.989678 | -3.401477 |
| 6 | 3.056153 | -5.045266 | -2.460413 |
| 1 | 2.617458 | -5.991281 | -2.765942 |
| 6 | 3.507486 | -4.871230 | -1.179241 |
| 1 | 3.404700 | -5.684121 | -0.464963 |
| 6 | 4.122190 | -3.661542 | -0.732549 |
| 6 | 0.802436 | 3.751862 | -0.191353 |
| 6 | -0.269103 | 3.441110 | 0.716158 |

| | | | |
|---|---|---|---|
| 6 | -0.047802 | 2.875370 | 2.004065 |
| 1 | 0.961971 | 2.635437 | 2.312910 |
| 6 | -1.097456 | 2.614557 | 2.857647 |
| 1 | -0.908656 | 2.169214 | 3.827493 |
| 6 | -2.427805 | 2.902898 | 2.471574 |
| 1 | -3.244773 | 2.691113 | 3.154144 |
| 6 | -2.683866 | 3.423995 | 1.223895 |
| 1 | -3.702359 | 3.631786 | 0.906097 |
| 6 | -1.622639 | 3.695788 | 0.318723 |
| 6 | -1.872482 | 4.216681 | -0.977212 |
| 1 | -2.899243 | 4.407099 | -1.278429 |
| 6 | -0.831479 | 4.477867 | -1.832851 |
| 1 | -1.041952 | 4.877234 | -2.820877 |
| 6 | 0.523976 | 4.253726 | -1.460662 |
| 8 | 1.603733 | -0.299831 | -2.487433 |
| 8 | 0.636009 | 0.283618 | -0.114603 |
| 8 | 3.044674 | -0.424789 | -0.311643 |
| 8 | 2.386709 | 1.770147 | -1.367745 |
| 15 | 1.762793 | 0.273669 | -1.101195 |
| 6 | 4.473762 | -3.654832 | 0.753944 |
| 6 | 5.683449 | -2.833958 | 1.236946 |
| 6 | 3.213531 | -3.275209 | 1.571319 |
| 1 | 4.705994 | -4.700602 | 1.005226 |
| 6 | 5.917713 | -3.062896 | 2.739053 |
| 1 | 5.507511 | -1.767268 | 1.076983 |
| 1 | 6.576191 | -3.096028 | 0.658065 |
| 6 | 3.430097 | -3.429575 | 3.082467 |
| 1 | 2.956610 | -2.241064 | 1.327060 |
| 1 | 2.367580 | -3.893389 | 1.248654 |
| 6 | 4.677913 | -2.672491 | 3.558189 |
| 1 | 6.789778 | -2.485219 | 3.069514 |
| 1 | 6.156570 | -4.121644 | 2.917382 |
| 1 | 2.538948 | -3.082308 | 3.618160 |
| 1 | 3.544795 | -4.497380 | 3.319424 |
| 1 | 4.849151 | -2.857856 | 4.625693 |
| 1 | 4.503687 | -1.593753 | 3.452325 |
| 6 | 1.609876 | 4.574917 | -2.472180 |
| 6 | 1.478605 | 3.726291 | -3.757585 |
| 6 | 1.664608 | 6.078395 | -2.824335 |
| 1 | 2.577597 | 4.325426 | -2.027619 |
| 6 | 2.630709 | 4.019040 | -4.729196 |
| 1 | 0.524497 | 3.956235 | -4.252818 |
| 1 | 1.460400 | 2.665578 | -3.491625 |
| 6 | 2.810389 | 6.374726 | -3.804346 |
| 1 | 0.710983 | 6.386824 | -3.274764 |
| 1 | 1.784274 | 6.667062 | -1.906691 |
| 6 | 2.708577 | 5.514231 | -5.072564 |
| 1 | 2.514714 | 3.422773 | -5.642813 |
| 1 | 3.575651 | 3.701433 | -4.265687 |
| 1 | 2.817157 | 7.440295 | -4.065452 |
| 1 | 3.768053 | 6.168208 | -3.305301 |
| 1 | 3.560694 | 5.712043 | -5.734661 |
| 1 | 1.804753 | 5.801348 | -5.629935 |

**2c**

Number of imaginary frequencies : 0
The smallest frequencies are : 13.9726 16.5940 17.9045 cm(-1)

Electronic energy : HF=-5577.5825694
Zero-point correction= 1.610562 (Hartree/Particle)

Thermal correction to Energy= 1.705909
Thermal correction to Enthalpy= 1.706853
Thermal correction to Gibbs Free Energy= 1.473962
Sum of electronic and zero-point Energies= -5575.972008
Sum of electronic and thermal Energies= -5575.876661
Sum of electronic and thermal Enthalpies= -5575.875717
Sum of electronic and thermal Free Energies= -5576.108607
............................................................
Cartesian Coordinates
............................................................

| | | | |
|---|---|---|---|
| 46 | -3.477694 | 1.408374 | -0.896591 |
| 6 | -2.556025 | -0.249500 | -1.939774 |
| 6 | -1.538456 | 0.681389 | -1.565003 |
| 15 | -5.592205 | 0.653314 | -0.213080 |
| 6 | -6.790116 | 0.504778 | -1.594950 |
| 6 | -6.339285 | -0.019122 | -2.817370 |
| 6 | -7.225783 | -0.187534 | -3.880628 |
| 6 | -8.567427 | 0.174296 | -3.736221 |
| 6 | -9.021615 | 0.699689 | -2.524501 |
| 6 | -8.139397 | 0.863390 | -1.454961 |
| 6 | -5.675066 | -0.946885 | 0.672720 |
| 6 | -6.746798 | -1.838047 | 0.510204 |
| 6 | -6.770803 | -3.036202 | 1.226754 |
| 6 | -5.731996 | -3.351684 | 2.105910 |
| 6 | -4.658602 | -2.471475 | 2.268245 |
| 6 | -4.630334 | -1.274734 | 1.551990 |
| 6 | -6.337156 | 1.884639 | 0.928210 |
| 6 | -6.307281 | 3.235535 | 0.537780 |
| 6 | -6.785429 | 4.227167 | 1.392601 |
| 6 | -7.290050 | 3.881216 | 2.649871 |
| 6 | -7.324866 | 2.542050 | 3.041736 |
| 6 | -6.853431 | 1.543667 | 2.185288 |
| 1 | -2.952208 | -0.150186 | -2.950418 |
| 1 | -7.557973 | -1.602978 | -0.171422 |
| 1 | -3.787324 | -0.602656 | 1.672947 |
| 1 | -7.600676 | -3.724188 | 1.092874 |
| 1 | -3.819362 | -2.725719 | 2.906252 |
| 1 | -5.749459 | -4.289300 | 2.653922 |
| 1 | -5.296419 | -0.296094 | -2.936256 |
| 1 | -8.500431 | 1.268986 | -0.514970 |
| 1 | -6.866940 | -0.593646 | -4.821796 |
| 1 | -10.064071 | 0.982133 | -2.409296 |
| 1 | -9.256318 | 0.049954 | -4.566645 |
| 1 | -5.911814 | 3.511364 | -0.437758 |
| 1 | -6.878967 | 0.505740 | 2.500643 |
| 1 | -6.757742 | 5.267460 | 1.081922 |
| 1 | -7.717785 | 2.269869 | 4.016958 |
| 1 | -7.652769 | 4.654034 | 3.321058 |
| 1 | -0.865316 | 0.468879 | -0.740419 |
| 6 | -1.605134 | 1.970635 | -2.066470 |
| 8 | 0.564414 | 2.241232 | -4.127198 |
| 6 | -2.711439 | -1.607586 | -1.303561 |
| 6 | -1.562351 | -2.642253 | -1.590421 |
| 1 | -2.798007 | -1.510827 | -0.221443 |
| 1 | -3.648833 | -2.031835 | -1.670482 |
| 6 | -0.329603 | -2.384275 | -0.650220 |
| 1 | 0.248161 | -1.526895 | -0.979477 |
| 1 | 0.321726 | -3.255870 | -0.696117 |
| 6 | -1.180283 | -2.477515 | -3.077391 |
| 6 | -1.944927 | -3.122251 | -4.065697 |
| 6 | -0.215903 | -1.548940 | -3.499911 |
| 6 | -1.751605 | -2.851929 | -5.420577 |

| | | | |
|---|---|---|---|
| 1 | -2.700994 | -3.842766 | -3.770166 |
| 6 | -0.036899 | -1.258957 | -4.855345 |
| 1 | 0.394726 | -0.996837 | -2.796655 |
| 6 | -0.799540 | -1.910554 | -5.823156 |
| 1 | -2.354581 | -3.370871 | -6.161412 |
| 1 | 0.678093 | -0.489898 | -5.128003 |
| 1 | -0.661688 | -1.685180 | -6.877328 |
| 6 | -2.052954 | -4.065181 | -1.271139 |
| 6 | -1.300309 | -5.162393 | -1.721945 |
| 6 | -3.167398 | -4.321919 | -0.466114 |
| 6 | -1.655902 | -6.466818 | -1.391788 |
| 1 | -0.425516 | -4.988444 | -2.342150 |
| 6 | -3.526064 | -5.630103 | -0.129472 |
| 1 | -3.763434 | -3.511090 | -0.069054 |
| 6 | -2.776469 | -6.708655 | -0.591761 |
| 1 | -1.055169 | -7.295994 | -1.755834 |
| 1 | -4.393671 | -5.794434 | 0.503849 |
| 1 | -3.054218 | -7.725879 | -0.329588 |
| 7 | -0.709928 | -2.105922 | 0.740481 |
| 1 | -0.544799 | -1.136155 | 1.011753 |
| 16 | -0.475272 | -3.181162 | 1.985234 |
| 8 | -1.504697 | -2.892219 | 3.000478 |
| 1 | 1.368516 | 2.330390 | -4.653955 |
| 1 | 0.879746 | 1.740839 | -3.347510 |
| 1 | -2.099624 | 2.194563 | -3.009119 |
| 1 | -0.960840 | 2.739871 | -1.660098 |
| 8 | -0.392695 | -4.523852 | 1.394444 |
| 6 | 1.099449 | -2.805582 | 2.800937 |
| 6 | 1.538406 | -3.661553 | 3.865174 |
| 6 | 1.784501 | -1.660210 | 2.467623 |
| 6 | 0.899167 | -4.876577 | 4.237181 |
| 6 | 2.711331 | -3.259376 | 4.591643 |
| 6 | 2.940411 | -1.283858 | 3.193261 |
| 1 | 1.453974 | -1.019204 | 1.660724 |
| 6 | 1.390142 | -5.644779 | 5.270026 |
| 1 | 0.026374 | -5.212798 | 3.692406 |
| 6 | 3.186789 | -4.078157 | 5.650933 |
| 6 | 3.383706 | -2.057071 | 4.237537 |
| 1 | 3.459550 | -0.375477 | 2.908608 |
| 6 | 2.542452 | -5.246589 | 5.986622 |
| 1 | 0.887286 | -6.570989 | 5.533008 |
| 1 | 4.076501 | -3.762663 | 6.189799 |
| 1 | 4.266038 | -1.768396 | 4.802795 |
| 1 | 2.916989 | -5.865736 | 6.796705 |
| 6 | 4.393640 | 0.457034 | -0.253439 |
| 6 | 5.177753 | -0.049844 | -1.270873 |
| 6 | 6.565602 | 0.298055 | -1.311229 |
| 6 | 7.447331 | -0.220572 | -2.298459 |
| 1 | 7.051836 | -0.896241 | -3.049047 |
| 6 | 8.783196 | 0.116949 | -2.300491 |
| 1 | 9.442765 | -0.294928 | -3.059096 |
| 6 | 9.305148 | 0.998827 | -1.322875 |
| 1 | 10.359947 | 1.257909 | -1.337694 |
| 6 | 8.475502 | 1.527133 | -0.359891 |
| 1 | 8.865567 | 2.209676 | 0.391099 |
| 6 | 7.093693 | 1.193997 | -0.325146 |
| 6 | 6.217160 | 1.762040 | 0.632292 |
| 1 | 6.607086 | 2.508193 | 1.318994 |
| 6 | 4.877886 | 1.422872 | 0.686025 |
| 6 | 3.967124 | 2.076192 | 1.659664 |
| 6 | 4.352517 | 2.329492 | 2.962841 |
| 1 | 5.331766 | 2.005682 | 3.304264 |

| | | | |
|---|---|---|---|
| 6 | 3.493783 | 2.987602 | 3.878435 |
| 6 | 3.910911 | 3.267937 | 5.208723 |
| 1 | 4.911995 | 2.971750 | 5.512171 |
| 6 | 3.071226 | 3.903411 | 6.094429 |
| 1 | 3.402191 | 4.113593 | 7.107398 |
| 6 | 1.772088 | 4.288522 | 5.683600 |
| 1 | 1.115439 | 4.796476 | 6.384187 |
| 6 | 1.333601 | 4.024116 | 4.404823 |
| 1 | 0.337419 | 4.324315 | 4.100118 |
| 6 | 2.172375 | 3.364346 | 3.464863 |
| 6 | 1.745860 | 3.062846 | 2.128726 |
| 6 | 2.657497 | 2.487881 | 1.263196 |
| 6 | 4.552001 | -0.802515 | -2.406289 |
| 6 | 4.209979 | 0.009338 | -3.553014 |
| 6 | 4.377568 | 1.422648 | -3.564377 |
| 1 | 4.760959 | 1.919686 | -2.682118 |
| 6 | 4.035616 | 2.178426 | -4.665303 |
| 1 | 4.166269 | 3.256669 | -4.637412 |
| 6 | 3.506674 | 1.563152 | -5.825480 |
| 1 | 3.247373 | 2.166248 | -6.691469 |
| 6 | 3.320125 | 0.197914 | -5.845955 |
| 1 | 2.904990 | -0.290345 | -6.724256 |
| 6 | 3.656283 | -0.601817 | -4.721683 |
| 6 | 3.437379 | -2.003464 | -4.715550 |
| 1 | 3.009789 | -2.477678 | -5.594871 |
| 6 | 3.746423 | -2.743040 | -3.606851 |
| 1 | 3.559546 | -3.814044 | -3.613735 |
| 6 | 4.308832 | -2.169803 | -2.423938 |
| 6 | 0.327202 | 3.295413 | 1.701390 |
| 6 | -0.690671 | 2.478618 | 2.310973 |
| 6 | -0.381157 | 1.369703 | 3.149454 |
| 1 | 0.655053 | 1.097831 | 3.303006 |
| 6 | -1.373840 | 0.613596 | 3.732117 |
| 1 | -1.112316 | -0.249841 | 4.335462 |
| 6 | -2.736566 | 0.941998 | 3.531067 |
| 1 | -3.511201 | 0.352632 | 4.012624 |
| 6 | -3.075958 | 1.986553 | 2.700865 |
| 1 | -4.117822 | 2.233082 | 2.519573 |
| 6 | -2.071072 | 2.754806 | 2.050325 |
| 6 | -2.399624 | 3.785824 | 1.127190 |
| 1 | -3.447355 | 4.023849 | 0.953155 |
| 6 | -1.403491 | 4.486279 | 0.486895 |
| 1 | -1.672446 | 5.253626 | -0.233065 |
| 6 | -0.023201 | 4.261250 | 0.759638 |
| 8 | 1.557646 | 0.852023 | -1.988600 |
| 8 | 0.693333 | 0.369631 | 0.432451 |
| 8 | 3.116638 | -0.045518 | -0.088984 |
| 8 | 2.305249 | 2.328523 | -0.062923 |
| 15 | 1.774514 | 0.828758 | -0.510123 |
| 6 | 4.505337 | -3.168712 | -1.286103 |
| 6 | 5.554431 | -2.873517 | -0.201136 |
| 6 | 3.140656 | -3.480187 | -0.620378 |
| 1 | 4.831317 | -4.101340 | -1.772722 |
| 6 | 5.685323 | -4.073491 | 0.752379 |
| 1 | 5.255190 | -1.998890 | 0.383153 |
| 1 | 6.521578 | -2.634995 | -0.657817 |
| 6 | 3.243554 | -4.653944 | 0.363266 |
| 1 | 2.798693 | -2.577773 | -0.100988 |
| 1 | 2.397439 | -3.701515 | -1.394697 |
| 6 | 4.338600 | -4.411257 | 1.409986 |
| 1 | 6.437452 | -3.855175 | 1.520810 |
| 1 | 6.052256 | -4.947015 | 0.193435 |

| | | | |
|---|---|---|---|
| 1 | 2.275600 | -4.816780 | 0.850759 |
| 1 | 3.473852 | -5.570888 | -0.199298 |
| 1 | 4.438460 | -5.283914 | 2.067097 |
| 1 | 4.037339 | -3.572834 | 2.046999 |
| 6 | 1.005546 | 5.082783 | 0.001884 |
| 6 | 0.950735 | 4.802708 | -1.518687 |
| 6 | 0.886657 | 6.599330 | 0.270876 |
| 1 | 1.999664 | 4.783178 | 0.343957 |
| 6 | 2.042869 | 5.578368 | -2.265695 |
| 1 | -0.031475 | 5.104198 | -1.910343 |
| 1 | 1.061576 | 3.734261 | -1.714243 |
| 6 | 1.979709 | 7.377137 | -0.478923 |
| 1 | -0.100302 | 6.959025 | -0.051861 |
| 1 | 0.953656 | 6.789718 | 1.349067 |
| 6 | 1.951050 | 7.085662 | -1.987501 |
| 1 | 1.966808 | 5.374920 | -3.340637 |
| 1 | 3.025476 | 5.204719 | -1.943933 |
| 1 | 1.869221 | 8.453056 | -0.295226 |
| 1 | 2.961185 | 7.088781 | -0.076184 |
| 1 | 2.764816 | 7.621028 | -2.492235 |
| 1 | 1.011503 | 7.471501 | -2.409662 |

---

**1d**

---

Number of imaginary frequencies : 0
The smallest frequencies are : 10.9565 12.5503 15.4252 cm(-1)

Electronic energy : HF=-4039.5429545
Zero-point correction= 1.080706 (Hartree/Particle)
Thermal correction to Energy= 1.147408
Thermal correction to Enthalpy= 1.148352
Thermal correction to Gibbs Free Energy= 0.973210
Sum of electronic and zero-point Energies= -4038.462248
Sum of electronic and thermal Energies= -4038.395547
Sum of electronic and thermal Enthalpies= -4038.394603
Sum of electronic and thermal Free Energies= -4038.569745

Cartesian Coordinates

| | | | |
|---|---|---|---|
| 6 | 4.049167 | -3.284622 | 2.543211 |
| 6 | 3.773440 | -2.079741 | 1.873896 |
| 6 | 4.133512 | -0.867597 | 2.480977 |
| 6 | 4.771898 | -0.857454 | 3.721978 |
| 6 | 5.045959 | -2.058801 | 4.377691 |
| 6 | 4.681006 | -3.271808 | 3.787230 |
| 15 | 2.974609 | -2.025168 | 0.212355 |
| 6 | 4.326722 | -2.425876 | -0.975679 |
| 6 | 5.501226 | -3.103312 | -0.617605 |
| 6 | 6.492348 | -3.346678 | -1.570636 |
| 6 | 6.320083 | -2.922993 | -2.890475 |
| 6 | 5.152457 | -2.249258 | -3.257502 |
| 6 | 4.164782 | -1.997463 | -2.304546 |
| 46 | 1.609948 | -0.194073 | -0.359840 |
| 6 | -0.473141 | 0.224753 | -0.895718 |
| 6 | -0.199457 | -1.155915 | -1.021750 |
| 15 | 2.640218 | 1.912548 | -0.148581 |
| 6 | 2.578926 | 2.878390 | -1.722042 |
| 6 | 1.314808 | 3.216853 | -2.237417 |
| 6 | 1.203368 | 3.893372 | -3.450982 |
| 6 | 2.350006 | 4.229521 | -4.176897 |
| 6 | 3.607756 | 3.898531 | -3.672009 |

| | | | |
|---|---|---|---|
| 6 | 3.724589 | 3.231996 | -2.448867 |
| 6 | 1.960168 | -3.574351 | 0.253615 |
| 6 | 2.137384 | -4.645945 | -0.634135 |
| 6 | 1.286527 | -5.754635 | -0.591018 |
| 6 | 0.253804 | -5.815988 | 0.350379 |
| 6 | 0.065200 | -4.745497 | 1.239998 |
| 6 | 0.906046 | -3.632327 | 1.182349 |
| 6 | 1.839681 | 3.028289 | 1.078936 |
| 6 | 1.792730 | 4.422455 | 0.931112 |
| 6 | 1.161917 | 5.212454 | 1.894036 |
| 6 | 0.568623 | 4.620371 | 3.012083 |
| 6 | 0.617526 | 3.233145 | 3.171926 |
| 6 | 1.248819 | 2.442202 | 2.210545 |
| 6 | 4.425422 | 1.948453 | 0.318141 |
| 6 | 5.276877 | 1.020880 | -0.307269 |
| 6 | 6.621458 | 0.936930 | 0.048763 |
| 6 | 7.131721 | 1.767612 | 1.050606 |
| 6 | 6.294106 | 2.690761 | 1.678603 |
| 6 | 4.948876 | 2.787932 | 1.311306 |
| 1 | -0.503401 | 0.835295 | -1.798480 |
| 1 | 2.949189 | -4.623329 | -1.354235 |
| 1 | 0.746371 | -2.804606 | 1.868224 |
| 1 | 1.441457 | -6.579127 | -1.280844 |
| 1 | -0.730528 | -4.782553 | 1.979348 |
| 1 | -0.391285 | -6.688594 | 0.400405 |
| 1 | 5.650898 | -3.430114 | 0.406139 |
| 1 | 3.271493 | -1.442006 | -2.581126 |
| 1 | 7.401193 | -3.865869 | -1.279953 |
| 1 | 5.017849 | -1.906412 | -4.279503 |
| 1 | 7.095652 | -3.109752 | -3.627552 |
| 1 | 3.924161 | 0.068437 | 1.979388 |
| 1 | 3.767230 | -4.233254 | 2.096757 |
| 1 | 5.051848 | 0.090871 | 4.171444 |
| 1 | 4.888080 | -4.209386 | 4.295421 |
| 1 | 5.538627 | -2.052228 | 5.345740 |
| 1 | 2.240381 | 4.890542 | 0.060094 |
| 1 | 1.272190 | 1.361575 | 2.326658 |
| 1 | 1.131537 | 6.291118 | 1.768815 |
| 1 | 0.154117 | 2.766492 | 4.036391 |
| 1 | 0.070129 | 5.236719 | 3.754508 |
| 1 | 0.417768 | 2.973085 | -1.678787 |
| 1 | 4.710956 | 2.994562 | -2.065000 |
| 1 | 0.218302 | 4.160240 | -3.820852 |
| 1 | 4.505011 | 4.165468 | -4.223540 |
| 1 | 2.262303 | 4.751929 | -5.125337 |
| 1 | 4.881048 | 0.349034 | -1.061899 |
| 1 | 4.309509 | 3.510633 | 1.807420 |
| 1 | 7.261902 | 0.209583 | -0.441782 |
| 1 | 6.685742 | 3.339948 | 2.456734 |
| 1 | 8.175266 | 1.692230 | 1.342401 |
| 1 | -0.543040 | -1.821087 | -0.231287 |
| 6 | 0.010275 | -1.828624 | -2.331485 |
| 8 | -1.294350 | -2.461342 | -2.749029 |
| 6 | -1.268335 | 0.728102 | 0.296387 |
| 6 | -2.645903 | 1.358491 | -0.063479 |
| 1 | -1.431659 | -0.117193 | 0.968180 |
| 1 | -0.707677 | 1.475403 | 0.858900 |
| 6 | -3.425995 | 0.395905 | -0.998869 |
| 1 | -2.998529 | 0.456804 | -1.999390 |
| 1 | -4.479210 | 0.686427 | -1.072115 |
| 6 | -2.397819 | 2.711477 | -0.778008 |
| 6 | -1.745493 | 3.729177 | -0.057387 |

| | | | |
|---|---|---|---|
| 6 | -2.790667 | 2.996309 | -2.092270 |
| 6 | -1.484702 | 4.969731 | -0.631226 |
| 1 | -1.441449 | 3.552535 | 0.969449 |
| 6 | -2.533904 | 4.245626 | -2.670771 |
| 1 | -3.317715 | 2.262853 | -2.691387 |
| 6 | -1.877405 | 5.236804 | -1.945906 |
| 1 | -0.968715 | 5.725935 | -0.048496 |
| 1 | -2.855703 | 4.436223 | -3.690946 |
| 1 | -1.674901 | 6.204741 | -2.394649 |
| 6 | -3.481670 | 1.676527 | 1.199090 |
| 6 | -4.708742 | 2.343967 | 1.044174 |
| 6 | -3.057297 | 1.382508 | 2.500053 |
| 6 | -5.487529 | 2.697101 | 2.143072 |
| 1 | -5.048585 | 2.613368 | 0.047854 |
| 6 | -3.834801 | 1.736536 | 3.607438 |
| 1 | -2.121751 | 0.866320 | 2.669883 |
| 6 | -5.052299 | 2.393944 | 3.436304 |
| 1 | -6.431136 | 3.212894 | 1.989908 |
| 1 | -3.480132 | 1.496360 | 4.605987 |
| 1 | -5.652182 | 2.673098 | 4.297884 |
| 7 | -3.298607 | -1.040659 | -0.702772 |
| 1 | -3.256784 | -1.611553 | -1.537839 |
| 16 | -3.978509 | -1.868758 | 0.554836 |
| 8 | -3.944047 | -3.291460 | 0.064507 |
| 1 | -1.197128 | -2.817973 | -3.648036 |
| 1 | -1.623765 | -3.471806 | -1.853392 |
| 1 | 0.289055 | -1.125160 | -3.119919 |
| 1 | 0.738015 | -2.644976 | -2.283077 |
| 8 | -3.273331 | -1.578433 | 1.801407 |
| 6 | -5.680211 | -1.360329 | 0.710262 |
| 6 | -6.605416 | -1.510306 | -0.375321 |
| 6 | -6.021865 | -0.742656 | 1.895922 |
| 6 | -6.320951 | -2.138583 | -1.618047 |
| 6 | -7.921199 | -0.968769 | -0.173483 |
| 6 | -7.329344 | -0.244055 | 2.077998 |
| 1 | -5.278798 | -0.616225 | 2.673909 |
| 6 | -7.276412 | -2.205805 | -2.609549 |
| 1 | -5.357661 | -2.602337 | -1.783832 |
| 6 | -8.879629 | -1.060680 | -1.217651 |
| 6 | -8.250586 | -0.350730 | 1.063837 |
| 1 | -7.584727 | 0.240635 | 3.013650 |
| 6 | -8.565528 | -1.659928 | -2.415614 |
| 1 | -7.038031 | -2.695570 | -3.549145 |
| 1 | -9.870399 | -0.648071 | -1.049692 |
| 1 | -9.254041 | 0.045948 | 1.191297 |
| 1 | -9.305045 | -1.724071 | -3.207750 |
| 8 | -1.944026 | -4.219228 | -1.148036 |
| 1 | -2.738346 | -3.863229 | -0.588131 |
| 1 | -1.210030 | -4.503988 | -0.557395 |

---

**[1d-2d]‡**

---

Number of imaginary frequencies : 1
The smallest frequencies are : -252.0992 12.9497 14.0899 cm(-1)

Electronic energy : HF=-4039.5409235
Zero-point correction= 1.081190 (Hartree/Particle)
Thermal correction to Energy= 1.147748
Thermal correction to Enthalpy= 1.148693
Thermal correction to Gibbs Free Energy= 0.973996
Sum of electronic and zero-point Energies= -4038.459733

Sum of electronic and thermal Energies= -4038.393175
Sum of electronic and thermal Enthalpies= -4038.392231
Sum of electronic and thermal Free Energies= -4038.566927

........................................................

Cartesian Coordinates

........................................................

| | | | |
|---|---|---|---|
| 6 | 4.107027 | -3.312420 | 2.508554 |
| 6 | 3.787635 | -2.092923 | 1.886694 |
| 6 | 4.057522 | -0.893403 | 2.561274 |
| 6 | 4.651248 | -0.908657 | 3.824181 |
| 6 | 4.970396 | -2.123580 | 4.432548 |
| 6 | 4.695365 | -3.324980 | 3.773447 |
| 15 | 3.043368 | -2.012944 | 0.203759 |
| 6 | 4.439410 | -2.306032 | -0.960180 |
| 6 | 5.635641 | -2.938338 | -0.592415 |
| 6 | 6.658442 | -3.106956 | -1.527990 |
| 6 | 6.496391 | -2.652769 | -2.838905 |
| 6 | 5.306598 | -2.024336 | -3.215290 |
| 6 | 4.286690 | -1.847311 | -2.279805 |
| 46 | 1.598282 | -0.212600 | -0.343830 |
| 6 | -0.460012 | 0.240688 | -0.886600 |
| 6 | -0.232639 | -1.159738 | -0.966218 |
| 15 | 2.595204 | 1.904088 | -0.086913 |
| 6 | 2.621382 | 2.837663 | -1.679147 |
| 6 | 1.388675 | 3.116541 | -2.295359 |
| 6 | 1.343324 | 3.775073 | -3.522839 |
| 6 | 2.528607 | 4.150532 | -4.162055 |
| 6 | 3.755890 | 3.879771 | -3.555980 |
| 6 | 3.805171 | 3.232379 | -2.318176 |
| 6 | 2.088843 | -3.598630 | 0.146386 |
| 6 | 2.345172 | -4.625740 | -0.773649 |
| 6 | 1.535603 | -5.765873 | -0.807661 |
| 6 | 0.471184 | -5.902672 | 0.087133 |
| 6 | 0.208238 | -4.880580 | 1.010942 |
| 6 | 1.004377 | -3.734730 | 1.030766 |
| 6 | 1.699800 | 3.015770 | 1.074424 |
| 6 | 1.576518 | 4.397685 | 0.868928 |
| 6 | 0.867125 | 5.184436 | 1.778554 |
| 6 | 0.274619 | 4.601476 | 2.901430 |
| 6 | 0.403226 | 3.227426 | 3.120862 |
| 6 | 1.109915 | 2.439019 | 2.211768 |
| 6 | 4.341493 | 1.966423 | 0.497947 |
| 6 | 5.264133 | 1.090085 | -0.099429 |
| 6 | 6.581927 | 1.032203 | 0.349574 |
| 6 | 6.993248 | 1.838097 | 1.415116 |
| 6 | 6.084347 | 2.711552 | 2.014172 |
| 6 | 4.765746 | 2.782562 | 1.555873 |
| 1 | -0.468518 | 0.810799 | -1.815765 |
| 1 | 3.183562 | -4.543603 | -1.458523 |
| 1 | 0.784783 | -2.943019 | 1.741881 |
| 1 | 1.749618 | -6.555651 | -1.521978 |
| 1 | -0.613461 | -4.977760 | 1.715528 |
| 1 | -0.144715 | -6.797246 | 0.073043 |
| 1 | 5.776297 | -3.289962 | 0.424314 |
| 1 | 3.374096 | -1.327634 | -2.562762 |
| 1 | 7.583667 | -3.592149 | -1.230562 |
| 1 | 5.179090 | -1.659241 | -4.230396 |
| 1 | 7.296392 | -2.781391 | -3.562163 |
| 1 | 3.815250 | 0.052960 | 2.095655 |
| 1 | 3.892452 | -4.252033 | 2.008750 |
| 1 | 4.861606 | 0.030374 | 4.327773 |
| 1 | 4.937041 | -4.273113 | 4.245227 |

| | | | |
|---|---|---|---|
| 1 | 5.427706 | -2.136869 | 5.417686 |
| 1 | 2.025237 | 4.859014 | -0.004759 |
| 1 | 1.188723 | 1.366724 | 2.372886 |
| 1 | 0.775248 | 6.253276 | 1.607918 |
| 1 | -0.057389 | 2.768186 | 3.990534 |
| 1 | -0.286160 | 5.213998 | 3.601167 |
| 1 | 0.463117 | 2.844849 | -1.800649 |
| 1 | 4.767337 | 3.042155 | -1.855409 |
| 1 | 0.379836 | 3.997737 | -3.970552 |
| 1 | 4.681263 | 4.178941 | -4.040158 |
| 1 | 2.494170 | 4.658434 | -5.121639 |
| 1 | 4.947430 | 0.440311 | -0.908456 |
| 1 | 4.070507 | 3.466666 | 2.031048 |
| 1 | 7.278963 | 0.344894 | -0.120806 |
| 1 | 6.398792 | 3.342204 | 2.840951 |
| 1 | 8.015494 | 1.782559 | 1.777755 |
| 1 | -0.585747 | -1.800799 | -0.163244 |
| 6 | 0.105845 | -1.827645 | -2.200845 |
| 8 | -1.356136 | -2.432164 | -2.863623 |
| 6 | -1.286199 | 0.799805 | 0.253154 |
| 6 | -2.669409 | 1.374430 | -0.172231 |
| 1 | -1.442790 | 0.006209 | 0.986232 |
| 1 | -0.745918 | 1.598210 | 0.761408 |
| 6 | -3.440240 | 0.317736 | -1.008849 |
| 1 | -3.010944 | 0.293352 | -2.011106 |
| 1 | -4.495247 | 0.596356 | -1.106519 |
| 6 | -2.429464 | 2.651315 | -1.019470 |
| 6 | -1.764478 | 3.731159 | -0.408966 |
| 6 | -2.847833 | 2.812057 | -2.346828 |
| 6 | -1.512807 | 4.911605 | -1.101032 |
| 1 | -1.445019 | 3.653359 | 0.625405 |
| 6 | -2.600988 | 4.001543 | -3.044039 |
| 1 | -3.386874 | 2.026534 | -2.864209 |
| 6 | -1.930299 | 5.055048 | -2.427614 |
| 1 | -0.986471 | 5.718653 | -0.600838 |
| 1 | -2.943128 | 4.097395 | -4.070866 |
| 1 | -1.737355 | 5.976866 | -2.968380 |
| 6 | -3.505313 | 1.823842 | 1.048908 |
| 6 | -4.744140 | 2.446577 | 0.823812 |
| 6 | -3.065065 | 1.702461 | 2.371825 |
| 6 | -5.522854 | 2.916905 | 1.878509 |
| 1 | -5.096008 | 2.586802 | -0.194539 |
| 6 | -3.842585 | 2.172883 | 3.434381 |
| 1 | -2.117817 | 1.230100 | 2.594319 |
| 6 | -5.074866 | 2.780320 | 3.194979 |
| 1 | -6.476685 | 3.393656 | 1.671560 |
| 1 | -3.477229 | 2.062204 | 4.451627 |
| 1 | -5.675708 | 3.149482 | 4.021241 |
| 7 | -3.298319 | -1.089977 | -0.588147 |
| 1 | -3.458814 | -1.712863 | -1.372314 |
| 16 | -3.955064 | -1.782966 | 0.781618 |
| 8 | -3.849154 | -3.240120 | 0.492909 |
| 1 | -1.178031 | -2.860641 | -3.720137 |
| 1 | -1.654204 | -3.191482 | -2.181396 |
| 1 | 0.432546 | -1.193014 | -3.022143 |
| 1 | 0.664583 | -2.757780 | -2.118096 |
| 8 | -3.271787 | -1.259644 | 1.963474 |
| 6 | -5.680946 | -1.316565 | 0.855082 |
| 6 | -6.598603 | -1.694070 | -0.181275 |
| 6 | -6.047587 | -0.498153 | 1.902727 |
| 6 | -6.292760 | -2.540581 | -1.282218 |
| 6 | -7.929028 | -1.158290 | -0.087874 |

| | | | |
|---|---|---|---|
| 6 | -7.371340 | -0.014375 | 1.990724 |
| 1 | -5.312921 | -0.206024 | 2.643467 |
| 6 | -7.237802 | -2.813052 | -2.248229 |
| 1 | -5.322538 | -3.015304 | -1.347307 |
| 6 | -8.876429 | -1.465794 | -1.100481 |
| 6 | -8.283386 | -0.330975 | 1.013225 |
| 1 | -7.645844 | 0.625366 | 2.822086 |
| 6 | -8.539448 | -2.268743 | -2.165727 |
| 1 | -6.981636 | -3.466399 | -3.077132 |
| 1 | -9.877868 | -1.053146 | -1.014664 |
| 1 | -9.298307 | 0.053727 | 1.064592 |
| 1 | -9.270897 | -2.495901 | -2.935021 |
| 8 | -2.010681 | -4.189905 | -1.242202 |
| 1 | -2.655193 | -3.884354 | -0.556219 |
| 1 | -1.245153 | -4.545501 | -0.758316 |

---

**2d**

---

Number of imaginary frequencies : 0
The smallest frequencies are : 7.7916 12.0438 16.8572 cm(-1)

Electronic energy : HF=-4039.5845284
Zero-point correction= 1.080823 (Hartree/Particle)
Thermal correction to Energy= 1.149177
Thermal correction to Enthalpy= 1.150121
Thermal correction to Gibbs Free Energy= 0.971046
Sum of electronic and zero-point Energies= -4038.503706
Sum of electronic and thermal Energies= -4038.435351
Sum of electronic and thermal Enthalpies= -4038.434407
Sum of electronic and thermal Free Energies= -4038.613483

Cartesian Coordinates

| | | | |
|---|---|---|---|
| 6 | 5.695442 | 2.979614 | -1.056004 |
| 6 | 4.580852 | 2.123483 | -1.097219 |
| 6 | 4.067266 | 1.724386 | -2.338647 |
| 6 | 4.671568 | 2.151050 | -3.522014 |
| 6 | 5.786555 | 2.988530 | -3.474245 |
| 6 | 6.294829 | 3.405460 | -2.240409 |
| 15 | 3.788599 | 1.509474 | 0.433580 |
| 6 | 4.930609 | 0.282787 | 1.188997 |
| 6 | 6.302231 | 0.236925 | 0.904222 |
| 6 | 7.102951 | -0.751791 | 1.477808 |
| 6 | 6.546171 | -1.699134 | 2.341004 |
| 6 | 5.182833 | -1.651187 | 2.641001 |
| 6 | 4.380054 | -0.665564 | 2.066750 |
| 46 | 1.637829 | 0.576786 | 0.231982 |
| 6 | -0.540080 | 0.188770 | 0.939133 |
| 6 | -0.359443 | 1.508772 | 0.508405 |
| 15 | 2.044942 | -1.532405 | -0.800540 |
| 6 | 2.151849 | -2.936691 | 0.378286 |
| 6 | 1.519387 | -2.820024 | 1.626073 |
| 6 | 1.585046 | -3.860413 | 2.553138 |
| 6 | 2.283507 | -5.029732 | 2.244759 |
| 6 | 2.908389 | -5.158397 | 1.002352 |
| 6 | 2.844158 | -4.119730 | 0.072552 |
| 6 | 3.859726 | 2.949390 | 1.579312 |
| 6 | 4.190436 | 2.788356 | 2.932133 |
| 6 | 4.150953 | 3.882097 | 3.800346 |
| 6 | 3.784749 | 5.142956 | 3.328166 |
| 6 | 3.456007 | 5.310195 | 1.979942 |

| | | | |
|---|---|---|---|
| 6 | 3.487892 | 4.221031 | 1.110473 |
| 6 | 0.670301 | -1.941278 | -1.953009 |
| 6 | 0.255476 | -3.259365 | -2.188682 |
| 6 | -0.775508 | -3.517054 | -3.094801 |
| 6 | -1.400365 | -2.465618 | -3.768832 |
| 6 | -0.991379 | -1.149793 | -3.539955 |
| 6 | 0.038481 | -0.888706 | -2.636844 |
| 6 | 3.538820 | -1.701286 | -1.866025 |
| 6 | 4.802576 | -1.886200 | -1.281454 |
| 6 | 5.948688 | -1.920408 | -2.074962 |
| 6 | 5.852032 | -1.772823 | -3.459994 |
| 6 | 4.598327 | -1.604926 | -4.049856 |
| 6 | 3.447530 | -1.571184 | -3.260962 |
| 1 | -0.222550 | -0.042925 | 1.956787 |
| 1 | 4.488241 | 1.816205 | 3.311545 |
| 1 | 3.234102 | 4.363147 | 0.063754 |
| 1 | 4.414789 | 3.746090 | 4.845135 |
| 1 | 3.175096 | 6.289661 | 1.603885 |
| 1 | 3.758638 | 5.992108 | 4.004647 |
| 1 | 6.744793 | 0.946966 | 0.214751 |
| 1 | 3.314477 | -0.653448 | 2.275592 |
| 1 | 8.162542 | -0.786853 | 1.241943 |
| 1 | 4.738664 | -2.388826 | 3.302387 |
| 1 | 7.171902 | -2.473120 | 2.775441 |
| 1 | 3.202053 | 1.073789 | -2.379496 |
| 1 | 6.086441 | 3.320561 | -0.102522 |
| 1 | 4.270361 | 1.826443 | -4.477208 |
| 1 | 7.156187 | 4.065642 | -2.200504 |
| 1 | 6.256712 | 3.321994 | -4.394739 |
| 1 | 0.727216 | -4.084741 | -1.665576 |
| 1 | 0.334596 | 0.139723 | -2.445006 |
| 1 | -1.095653 | -4.540865 | -3.265163 |
| 1 | -1.495119 | -0.328296 | -4.037335 |
| 1 | -2.214939 | -2.667243 | -4.457587 |
| 1 | 0.977356 | -1.915861 | 1.879081 |
| 1 | 3.344268 | -4.226927 | -0.884132 |
| 1 | 1.093896 | -3.750301 | 3.514429 |
| 1 | 3.450360 | -6.066543 | 0.755339 |
| 1 | 2.340746 | -5.837056 | 2.969042 |
| 1 | 4.895831 | -2.020809 | -0.211115 |
| 1 | 2.482029 | -1.443992 | -3.738526 |
| 1 | 6.916755 | -2.062411 | -1.604155 |
| 1 | 4.510854 | -1.504004 | -5.127770 |
| 1 | 6.746532 | -1.796234 | -4.075425 |
| 1 | -0.818959 | 1.834579 | -0.420010 |
| 6 | 0.649968 | 2.303433 | 1.106893 |
| 8 | -2.564685 | 3.939140 | 0.137741 |
| 6 | -1.504915 | -0.785862 | 0.327984 |
| 6 | -2.854762 | -0.865172 | 1.108030 |
| 1 | -1.709904 | -0.518810 | -0.706884 |
| 1 | -1.049115 | -1.778647 | 0.323730 |
| 6 | -3.549952 | 0.529627 | 1.047346 |
| 1 | -3.072196 | 1.196666 | 1.765550 |
| 1 | -4.602170 | 0.437812 | 1.338868 |
| 6 | -2.557533 | -1.268871 | 2.573251 |
| 6 | -1.960640 | -2.521713 | 2.804533 |
| 6 | -2.844966 | -0.470080 | 3.687714 |
| 6 | -1.648505 | -2.949624 | 4.092519 |
| 1 | -1.757568 | -3.179667 | 1.965243 |
| 6 | -2.531293 | -0.896979 | 4.983716 |
| 1 | -3.327951 | 0.493295 | 3.575030 |
| 6 | -1.927714 | -2.134328 | 5.193617 |

| | | | |
|---|---|---|---|
| 1 | -1.201145 | -3.928747 | 4.236987 |
| 1 | -2.770320 | -0.255774 | 5.827527 |
| 1 | -1.690449 | -2.467523 | 6.199705 |
| 6 | -3.782827 | -1.952039 | 0.523267 |
| 6 | -4.993752 | -2.223901 | 1.181199 |
| 6 | -3.467705 | -2.704723 | -0.612354 |
| 6 | -5.865139 | -3.206732 | 0.717164 |
| 1 | -5.251042 | -1.670440 | 2.080107 |
| 6 | -4.338555 | -3.692248 | -1.081256 |
| 1 | -2.558121 | -2.516651 | -1.165693 |
| 6 | -5.540389 | -3.948724 | -0.421870 |
| 1 | -6.795189 | -3.393997 | 1.246277 |
| 1 | -4.071230 | -4.257213 | -1.970030 |
| 1 | -6.214794 | -4.718332 | -0.786627 |
| 7 | -3.400265 | 1.268271 | -0.211172 |
| 1 | -3.250212 | 2.279405 | -0.076476 |
| 16 | -4.219227 | 0.966193 | -1.614383 |
| 8 | -4.038199 | 2.213012 | -2.405316 |
| 1 | -3.175531 | 4.684385 | 0.073758 |
| 1 | -2.094429 | 3.902594 | -0.726277 |
| 1 | 0.879728 | 2.191886 | 2.165777 |
| 1 | 0.870770 | 3.281163 | 0.691493 |
| 8 | -3.758944 | -0.289902 | -2.211807 |
| 6 | -5.947365 | 0.774708 | -1.178308 |
| 6 | -6.689041 | 1.845956 | -0.580285 |
| 6 | -6.491366 | -0.477579 | -1.371270 |
| 6 | -6.183898 | 3.153755 | -0.341003 |
| 6 | -8.041197 | 1.556224 | -0.189416 |
| 6 | -7.828906 | -0.733447 | -0.996237 |
| 1 | -5.882561 | -1.269149 | -1.791853 |
| 6 | -6.969169 | 4.111135 | 0.264398 |
| 1 | -5.180573 | 3.410466 | -0.653117 |
| 6 | -8.820652 | 2.572044 | 0.426025 |
| 6 | -8.581112 | 0.260753 | -0.418269 |
| 1 | -8.243271 | -1.723300 | -1.154679 |
| 6 | -8.297463 | 3.823825 | 0.654373 |
| 1 | -6.565130 | 5.104635 | 0.436955 |
| 1 | -9.842291 | 2.339047 | 0.713576 |
| 1 | -9.607710 | 0.068475 | -0.118478 |
| 1 | -8.900963 | 4.592885 | 1.126750 |
| 8 | -1.475488 | 3.225180 | -2.254759 |
| 1 | -2.336663 | 2.817714 | -2.492806 |
| 1 | -1.263830 | 3.835154 | -2.973417 |

---

**2a'**exo-*re*

---

Number of imaginary frequencies : 0
The smallest frequencies are : 9.0630 12.6580 13.9010 cm(-1)

Electronic energy : HF=-6614.0110187
Zero-point correction= 1.889150 (Hartree/Particle)
Thermal correction to Energy= 2.000741
Thermal correction to Enthalpy= 2.001686
Thermal correction to Gibbs Free Energy= 1.733896
Sum of electronic and zero-point Energies= -6612.121869
Sum of electronic and thermal Energies= -6612.010277
Sum of electronic and thermal Enthalpies= -6612.009333
Sum of electronic and thermal Free Energies= -6612.277123

............................................................

Cartesian Coordinates

............................................................

| | | | |
|---|---|---|---|
| 6 | -5.852521 | -3.762180 | -0.955186 |
| 6 | -4.844488 | -4.280486 | -0.127480 |
| 6 | -5.052652 | -5.512228 | 0.514913 |
| 6 | -6.246687 | -6.208369 | 0.326539 |
| 6 | -7.245831 | -5.682908 | -0.497854 |
| 6 | -7.047599 | -4.458509 | -1.137887 |
| 15 | -3.286433 | -3.325602 | 0.072496 |
| 6 | -2.053832 | -4.137805 | -1.016180 |
| 6 | -2.292543 | -5.374325 | -1.637713 |
| 6 | -1.292594 | -5.973866 | -2.406323 |
| 6 | -0.047856 | -5.356192 | -2.553970 |
| 6 | 0.188546 | -4.122656 | -1.944978 |
| 6 | -0.810420 | -3.509625 | -1.190543 |
| 46 | -3.783835 | -1.066147 | -0.337756 |
| 6 | -1.551331 | 2.016523 | 0.137301 |
| 6 | -1.880902 | 0.879974 | 0.793295 |
| 15 | -4.915888 | 0.905956 | -1.033765 |
| 6 | -2.744881 | -3.724725 | 1.785064 |
| 6 | -1.460280 | -4.198995 | 2.080855 |
| 6 | -1.099963 | -4.467811 | 3.403720 |
| 6 | -2.013609 | -4.271654 | 4.438742 |
| 6 | -3.296897 | -3.796879 | 4.149520 |
| 6 | -3.656851 | -3.511901 | 2.833826 |
| 6 | -6.648610 | 0.289468 | -1.194508 |
| 6 | -6.977218 | -0.502918 | -2.309542 |
| 6 | -8.238759 | -1.089116 | -2.411979 |
| 6 | -9.183874 | -0.903526 | -1.399084 |
| 6 | -8.861812 | -0.123019 | -0.286884 |
| 6 | -7.603003 | 0.472173 | -0.183024 |
| 6 | -4.522925 | 1.555538 | -2.698264 |
| 6 | -5.468445 | 2.251667 | -3.473554 |
| 6 | -5.112619 | 2.745847 | -4.728050 |
| 6 | -3.816231 | 2.550746 | -5.218927 |
| 6 | -2.878974 | 1.855010 | -4.454753 |
| 6 | -3.231037 | 1.351778 | -3.200216 |
| 1 | -0.737619 | -4.359706 | 1.288782 |
| 1 | -4.653299 | -3.135938 | 2.617679 |
| 1 | -0.099025 | -4.820738 | 3.621791 |
| 1 | -4.013676 | -3.638861 | 4.950204 |
| 1 | -1.722372 | -4.473847 | 5.465163 |
| 1 | -3.249765 | -5.872282 | -1.523775 |
| 1 | -0.580469 | -2.556131 | -0.735511 |
| 1 | -1.487627 | -6.929478 | -2.885312 |
| 1 | 1.140952 | -3.616281 | -2.036614 |
| 1 | 0.736369 | -5.833855 | -3.133946 |
| 1 | -4.285515 | -5.924050 | 1.163057 |
| 1 | -5.711391 | -2.808099 | -1.456307 |
| 1 | -6.397910 | -7.160719 | 0.826680 |
| 1 | -7.821574 | -4.034384 | -1.770084 |
| 1 | -8.176602 | -6.225437 | -0.636795 |
| 1 | -6.249852 | -0.651270 | -3.102547 |
| 1 | -7.369261 | 1.085212 | 0.681780 |
| 1 | -8.482806 | -1.687116 | -3.285471 |
| 1 | -9.594143 | 0.031554 | 0.500375 |
| 1 | -10.165407 | -1.361556 | -1.478400 |
| 1 | -6.479308 | 2.396395 | -3.103927 |
| 1 | -2.476111 | 0.811414 | -2.640419 |
| 1 | -5.846923 | 3.280865 | -5.323716 |
| 1 | -1.869385 | 1.691357 | -4.817385 |
| 1 | -3.542208 | 2.939281 | -6.195666 |
| 6 | -1.841457 | -0.429593 | 0.181431 |
| 6 | -1.577735 | 3.346959 | 0.830912 |

| | | | |
|---|---|---|---|
| 6 | -0.623033 | 4.492789 | 0.364931 |
| 1 | -1.389609 | 3.170237 | 1.892557 |
| 1 | -2.603429 | 3.732244 | 0.780613 |
| 6 | 0.813105 | 3.950515 | 0.110298 |
| 1 | 1.489984 | 4.797624 | -0.014094 |
| 1 | 1.148542 | 3.388396 | 0.986619 |
| 6 | -1.114721 | 5.219209 | -0.896550 |
| 6 | -0.621679 | 6.501673 | -1.184910 |
| 6 | -2.009842 | 4.641807 | -1.803107 |
| 6 | -1.008275 | 7.183713 | -2.335326 |
| 1 | 0.074133 | 6.971108 | -0.495499 |
| 6 | -2.401758 | 5.321769 | -2.959941 |
| 1 | -2.414776 | 3.653948 | -1.629082 |
| 6 | -1.904406 | 6.594865 | -3.231004 |
| 1 | -0.608266 | 8.174474 | -2.534146 |
| 1 | -3.089737 | 4.840832 | -3.649109 |
| 1 | -2.206071 | 7.122897 | -4.131557 |
| 6 | -0.602413 | 5.490366 | 1.544723 |
| 6 | -1.796504 | 6.148306 | 1.888474 |
| 6 | 0.530762 | 5.746027 | 2.328324 |
| 6 | -1.861795 | 7.011941 | 2.978843 |
| 1 | -2.683292 | 5.983492 | 1.285177 |
| 6 | 0.471717 | 6.620492 | 3.419732 |
| 1 | 1.477473 | 5.268332 | 2.108351 |
| 6 | -0.723473 | 7.254154 | 3.754206 |
| 1 | -2.800405 | 7.504926 | 3.218853 |
| 1 | 1.368956 | 6.800958 | 4.005658 |
| 1 | -0.769016 | 7.931517 | 4.602372 |
| 7 | 0.907826 | 3.036033 | -1.036889 |
| 1 | 1.237482 | 2.100045 | -0.737951 |
| 16 | 1.774936 | 3.468036 | -2.388619 |
| 8 | 1.292239 | 4.755709 | -2.894693 |
| 8 | 1.708295 | 2.282110 | -3.266769 |
| 6 | -5.018608 | 2.362618 | 0.077821 |
| 6 | -5.159956 | 3.673266 | -0.402376 |
| 6 | -4.934774 | 2.144721 | 1.462900 |
| 6 | -5.241340 | 4.744556 | 0.489124 |
| 1 | -5.186628 | 3.868131 | -1.467971 |
| 6 | -5.023417 | 3.217119 | 2.349691 |
| 1 | -4.774098 | 1.140365 | 1.844640 |
| 6 | -5.179553 | 4.518234 | 1.865477 |
| 1 | -5.337265 | 5.755076 | 0.103354 |
| 1 | -4.944957 | 3.037093 | 3.416022 |
| 1 | -5.232430 | 5.352697 | 2.558575 |
| 6 | 3.498096 | 3.634260 | -1.859222 |
| 6 | 4.041958 | 4.852280 | -1.327461 |
| 6 | 4.235275 | 2.466742 | -1.895143 |
| 6 | 3.358097 | 6.102005 | -1.257195 |
| 6 | 5.391438 | 4.797548 | -0.828162 |
| 6 | 5.562769 | 2.443531 | -1.419696 |
| 1 | 3.791367 | 1.559983 | -2.286152 |
| 6 | 3.956604 | 7.207637 | -0.693351 |
| 1 | 2.360634 | 6.183568 | -1.666803 |
| 6 | 5.975184 | 5.963218 | -0.260511 |
| 6 | 6.124078 | 3.583456 | -0.896617 |
| 1 | 6.119722 | 1.515777 | -1.452539 |
| 6 | 5.274450 | 7.143721 | -0.184021 |
| 1 | 3.408118 | 8.144422 | -0.649075 |
| 1 | 6.992551 | 5.898218 | 0.116914 |
| 1 | 7.141297 | 3.567460 | -0.513990 |
| 1 | 5.730048 | 8.025672 | 0.256974 |
| 6 | 1.480878 | -1.881150 | 2.291397 |

| | | | |
|---|---|---|---|
| 6 | 1.242051 | -1.337120 | 3.543333 |
| 6 | 1.496108 | -2.127340 | 4.709541 |
| 6 | 1.192384 | -1.672905 | 6.023772 |
| 1 | 0.752746 | -0.690453 | 6.151487 |
| 6 | 1.440250 | -2.466802 | 7.122155 |
| 1 | 1.192518 | -2.103956 | 8.115711 |
| 6 | 2.018583 | -3.750947 | 6.970441 |
| 1 | 2.212101 | -4.363434 | 7.846376 |
| 6 | 2.340667 | -4.213601 | 5.714737 |
| 1 | 2.793982 | -5.193462 | 5.586029 |
| 6 | 2.087572 | -3.423591 | 4.560172 |
| 6 | 2.434295 | -3.873167 | 3.263272 |
| 1 | 2.970005 | -4.812706 | 3.163751 |
| 6 | 2.141765 | -3.139997 | 2.126757 |
| 6 | 2.619546 | -3.638076 | 0.812136 |
| 6 | 2.546046 | -4.977518 | 0.479969 |
| 1 | 1.981338 | -5.656079 | 1.113082 |
| 6 | 3.209548 | -5.497749 | -0.657911 |
| 6 | 3.116445 | -6.873074 | -1.005861 |
| 1 | 2.480000 | -7.518337 | -0.405600 |
| 6 | 3.819981 | -7.380988 | -2.074746 |
| 1 | 3.740667 | -8.433325 | -2.332082 |
| 6 | 4.664682 | -6.532466 | -2.831047 |
| 1 | 5.238560 | -6.942522 | -3.657273 |
| 6 | 4.766829 | -5.192414 | -2.526009 |
| 1 | 5.419637 | -4.548838 | -3.103811 |
| 6 | 4.022108 | -4.625677 | -1.456283 |
| 6 | 4.043306 | -3.223570 | -1.161961 |
| 6 | 3.318109 | -2.765481 | -0.077637 |
| 6 | 0.812395 | 0.091596 | 3.678317 |
| 6 | -0.545835 | 0.400420 | 4.003217 |
| 6 | -1.551390 | -0.603597 | 4.094273 |
| 1 | -1.284208 | -1.637110 | 3.905658 |
| 6 | -2.852711 | -0.276172 | 4.407754 |
| 1 | -3.600845 | -1.059267 | 4.470453 |
| 6 | -3.221173 | 1.070445 | 4.648215 |
| 1 | -4.250005 | 1.309514 | 4.903372 |
| 6 | -2.276189 | 2.067847 | 4.558742 |
| 1 | -2.547388 | 3.107784 | 4.722891 |
| 6 | -0.925379 | 1.763167 | 4.238329 |
| 6 | 0.060656 | 2.773783 | 4.121487 |
| 1 | -0.219035 | 3.810159 | 4.286955 |
| 6 | 1.351691 | 2.451336 | 3.777239 |
| 1 | 2.082010 | 3.247107 | 3.681332 |
| 6 | 1.760996 | 1.109447 | 3.546951 |
| 6 | 4.892625 | -2.268712 | -1.945946 |
| 6 | 6.104575 | -1.792071 | -1.332507 |
| 6 | 6.490017 | -2.145982 | -0.008367 |
| 1 | 5.861144 | -2.816902 | 0.564829 |
| 6 | 7.639236 | -1.642952 | 0.562348 |
| 1 | 7.901250 | -1.922551 | 1.578852 |
| 6 | 8.478941 | -0.763956 | -0.162086 |
| 1 | 9.380450 | -0.371429 | 0.299791 |
| 6 | 8.148394 | -0.413821 | -1.451763 |
| 1 | 8.784535 | 0.258600 | -2.022530 |
| 6 | 6.965253 | -0.910187 | -2.064153 |
| 6 | 6.596026 | -0.530464 | -3.381332 |
| 1 | 7.245254 | 0.140289 | -3.938647 |
| 6 | 5.426565 | -0.985172 | -3.934371 |
| 1 | 5.152702 | -0.668774 | -4.935891 |
| 6 | 4.546456 | -1.856283 | -3.230185 |
| 8 | 2.141434 | 0.841074 | 0.082757 |

| | | | |
|---|---|---|---|
| 8 | 1.228564 | -1.187368 | -1.319474 |
| 8 | 0.998186 | -1.204619 | 1.200724 |
| 8 | 3.322004 | -1.411580 | 0.199680 |
| 15 | 1.883666 | -0.624746 | -0.088616 |
| 6 | 3.252902 | -2.272288 | -3.905361 |
| 6 | 3.486402 | -3.159298 | -5.150385 |
| 6 | 2.376477 | -1.059146 | -4.290958 |
| 1 | 2.676873 | -2.858744 | -3.184862 |
| 6 | 2.153790 | -3.621122 | -5.758433 |
| 1 | 4.050547 | -2.587192 | -5.900596 |
| 1 | 4.098647 | -4.028483 | -4.890470 |
| 6 | 1.037688 | -1.522615 | -4.880584 |
| 1 | 2.903305 | -0.442088 | -5.031861 |
| 1 | 2.205673 | -0.435150 | -3.413425 |
| 6 | 1.245570 | -2.431044 | -6.100387 |
| 1 | 2.340444 | -4.232699 | -6.650606 |
| 1 | 1.639056 | -4.269287 | -5.034960 |
| 1 | 0.423113 | -0.653124 | -5.132684 |
| 1 | 0.482718 | -2.068278 | -4.106909 |
| 1 | 0.280623 | -2.788546 | -6.481970 |
| 1 | 1.708951 | -1.849340 | -6.910904 |
| 6 | 3.213187 | 0.800189 | 3.209812 |
| 6 | 4.025326 | 0.386748 | 4.461549 |
| 6 | 3.968181 | 1.911216 | 2.455236 |
| 1 | 3.213133 | -0.062058 | 2.538875 |
| 6 | 5.416301 | -0.122788 | 4.055039 |
| 1 | 4.121898 | 1.256730 | 5.127423 |
| 1 | 3.494133 | -0.383497 | 5.027064 |
| 6 | 5.356234 | 1.406250 | 2.031251 |
| 1 | 4.092825 | 2.794503 | 3.098426 |
| 1 | 3.395959 | 2.208129 | 1.573361 |
| 6 | 6.181430 | 0.923434 | 3.232409 |
| 1 | 5.990726 | -0.408088 | 4.945620 |
| 1 | 5.290983 | -1.034106 | 3.452654 |
| 1 | 5.892590 | 2.196788 | 1.496616 |
| 1 | 5.227968 | 0.581025 | 1.319245 |
| 1 | 7.139730 | 0.512588 | 2.890176 |
| 1 | 6.418147 | 1.782255 | 3.878315 |
| 1 | -1.358708 | -0.467479 | -0.794016 |
| 1 | -1.514133 | -1.210424 | 0.863412 |
| 1 | -2.148215 | 0.928807 | 1.844470 |
| 1 | -1.263230 | 1.950705 | -0.906660 |
| 1 | 0.179843 | -0.213767 | -2.322550 |
| 8 | -0.403335 | 0.427109 | -2.781388 |
| 1 | 0.199633 | 1.157972 | -2.996256 |

---

**[*re-si*]$^{\ddagger}$**

---

Number of imaginary frequencies : 1
The smallest frequencies are : -99.7907 11.6406 16.9793 cm(-1)

Electronic energy : HF=-6613.9935096
Zero-point correction= 1.888202 (Hartree/Particle)
Thermal correction to Energy= 1.999358
Thermal correction to Enthalpy= 2.000302
Thermal correction to Gibbs Free Energy= 1.734531
Sum of electronic and zero-point Energies= -6612.105308
Sum of electronic and thermal Energies= -6611.994152
Sum of electronic and thermal Enthalpies= -6611.993208
Sum of electronic and thermal Free Energies= -6612.258978
............................................................

Cartesian Coordinates

.............................................................

| | | | |
|---|---|---|---|
| 6 | -5.121981 | -4.408552 | -0.701665 |
| 6 | -4.147580 | -4.638545 | 0.280900 |
| 6 | -4.344417 | -5.681788 | 1.202045 |
| 6 | -5.494379 | -6.469203 | 1.143083 |
| 6 | -6.455096 | -6.236359 | 0.156138 |
| 6 | -6.264385 | -5.207844 | -0.767582 |
| 15 | -2.599291 | -3.640867 | 0.245381 |
| 6 | -1.433594 | -4.707884 | -0.699741 |
| 6 | -1.694876 | -6.064569 | -0.955293 |
| 6 | -0.754074 | -6.847903 | -1.624481 |
| 6 | 0.459194 | -6.291183 | -2.034215 |
| 6 | 0.717757 | -4.942291 | -1.788648 |
| 6 | -0.225876 | -4.143247 | -1.140119 |
| 46 | -3.071279 | -1.394726 | -0.508638 |
| 6 | -2.031058 | 1.937335 | 0.678823 |
| 6 | -1.719197 | 0.806254 | 1.313585 |
| 15 | -4.752674 | 0.225718 | -0.696892 |
| 6 | -1.992220 | -3.707175 | 1.982733 |
| 6 | -0.638823 | -3.899852 | 2.290817 |
| 6 | -0.206782 | -3.848460 | 3.618872 |
| 6 | -1.115882 | -3.607920 | 4.649259 |
| 6 | -2.464959 | -3.398174 | 4.347762 |
| 6 | -2.898297 | -3.434455 | 3.023750 |
| 6 | -6.173313 | -0.845065 | -1.191337 |
| 6 | -6.184194 | -1.358579 | -2.501339 |
| 6 | -7.214559 | -2.199937 | -2.920535 |
| 6 | -8.243673 | -2.540472 | -2.038182 |
| 6 | -8.229213 | -2.046523 | -0.732014 |
| 6 | -7.198935 | -1.206292 | -0.306677 |
| 6 | -4.758277 | 1.459656 | -2.058192 |
| 6 | -5.969208 | 1.963236 | -2.570031 |
| 6 | -5.961818 | 2.851875 | -3.643035 |
| 6 | -4.749268 | 3.244558 | -4.218337 |
| 6 | -3.545405 | 2.747529 | -3.721608 |
| 6 | -3.548970 | 1.853904 | -2.648542 |
| 1 | 0.084538 | -4.083834 | 1.503568 |
| 1 | -3.946705 | -3.262742 | 2.796702 |
| 1 | 0.845573 | -3.985033 | 3.843382 |
| 1 | -3.179556 | -3.204812 | 5.142839 |
| 1 | -0.773168 | -3.568106 | 5.679059 |
| 1 | -2.627079 | -6.515405 | -0.634036 |
| 1 | 0.001554 | -3.095444 | -0.967878 |
| 1 | -0.969823 | -7.895007 | -1.818517 |
| 1 | 1.659616 | -4.508411 | -2.097800 |
| 1 | 1.202393 | -6.901291 | -2.538907 |
| 1 | -3.598048 | -5.883668 | 1.962795 |
| 1 | -4.999245 | -3.601722 | -1.417428 |
| 1 | -5.633857 | -7.269312 | 1.864493 |
| 1 | -7.006181 | -5.013518 | -1.534329 |
| 1 | -7.347857 | -6.853380 | 0.108695 |
| 1 | -5.400040 | -1.079520 | -3.200070 |
| 1 | -7.204973 | -0.821379 | 0.707934 |
| 1 | -7.216246 | -2.581736 | -3.937401 |
| 1 | -9.025053 | -2.310370 | -0.041405 |
| 1 | -9.051231 | -3.188224 | -2.367136 |
| 1 | -6.915651 | 1.644900 | -2.143252 |
| 1 | -2.608889 | 1.468703 | -2.273632 |
| 1 | -6.901407 | 3.235743 | -4.030767 |
| 1 | -2.599609 | 3.067341 | -4.142694 |
| 1 | -4.744032 | 3.943252 | -5.049928 |

| | | | |
|---|---|---|---|
| 6 | -1.910844 | -0.631154 | 1.016869 |
| 6 | -1.708327 | 3.279798 | 1.284892 |
| 6 | -1.153676 | 4.404646 | 0.356973 |
| 1 | -0.966679 | 3.127797 | 2.070378 |
| 1 | -2.599855 | 3.664474 | 1.795403 |
| 6 | 0.067440 | 3.869432 | -0.429580 |
| 1 | 0.538083 | 4.691027 | -0.971108 |
| 1 | 0.807347 | 3.476621 | 0.271326 |
| 6 | -2.201184 | 4.997833 | -0.599123 |
| 6 | -1.820462 | 6.006407 | -1.498615 |
| 6 | -3.551725 | 4.634635 | -0.559802 |
| 6 | -2.750314 | 6.618501 | -2.334812 |
| 1 | -0.785440 | 6.330261 | -1.535527 |
| 6 | -4.491391 | 5.253435 | -1.388387 |
| 1 | -3.889883 | 3.866460 | 0.122284 |
| 6 | -4.095681 | 6.244404 | -2.283778 |
| 1 | -2.424507 | 7.392184 | -3.024853 |
| 1 | -5.531675 | 4.944590 | -1.342163 |
| 1 | -4.823421 | 6.719864 | -2.935573 |
| 6 | -0.695021 | 5.534114 | 1.312662 |
| 6 | -1.664737 | 6.209250 | 2.073909 |
| 6 | 0.647817 | 5.885784 | 1.508817 |
| 6 | -1.308341 | 7.174851 | 3.012864 |
| 1 | -2.714732 | 5.979728 | 1.919424 |
| 6 | 1.008796 | 6.862316 | 2.443069 |
| 1 | 1.438054 | 5.401658 | 0.948551 |
| 6 | 0.036306 | 7.505341 | 3.206781 |
| 1 | -2.081931 | 7.675934 | 3.588747 |
| 1 | 2.058545 | 7.112627 | 2.571411 |
| 1 | 0.318178 | 8.259786 | 3.935889 |
| 7 | -0.258738 | 2.767272 | -1.357970 |
| 1 | 0.165655 | 1.903135 | -0.995780 |
| 16 | 0.229081 | 2.904742 | -2.949280 |
| 8 | -0.490071 | 4.020407 | -3.578341 |
| 8 | 0.052406 | 1.548612 | -3.507840 |
| 6 | -5.304052 | 1.071016 | 0.835203 |
| 6 | -5.905847 | 2.336804 | 0.836918 |
| 6 | -5.078610 | 0.417369 | 2.057203 |
| 6 | -6.256182 | 2.947023 | 2.043328 |
| 1 | -6.085378 | 2.856694 | -0.097461 |
| 6 | -5.443674 | 1.022485 | 3.258389 |
| 1 | -4.596969 | -0.555754 | 2.064269 |
| 6 | -6.024455 | 2.293239 | 3.254727 |
| 1 | -6.705124 | 3.936049 | 2.032813 |
| 1 | -5.254461 | 0.509949 | 4.196557 |
| 1 | -6.290126 | 2.773783 | 4.192003 |
| 6 | 1.999679 | 3.267709 | -2.901529 |
| 6 | 2.542803 | 4.597835 | -2.876021 |
| 6 | 2.795193 | 2.158988 | -2.696282 |
| 6 | 1.803536 | 5.798496 | -3.085945 |
| 6 | 3.955319 | 4.716094 | -2.620388 |
| 6 | 4.173959 | 2.299764 | -2.445550 |
| 1 | 2.357126 | 1.171576 | -2.704244 |
| 6 | 2.417822 | 7.030771 | -3.022672 |
| 1 | 0.747778 | 5.735033 | -3.308461 |
| 6 | 4.550765 | 6.005746 | -2.567989 |
| 6 | 4.738774 | 3.550794 | -2.408505 |
| 1 | 4.768048 | 1.416596 | -2.259397 |
| 6 | 3.802334 | 7.143398 | -2.759033 |
| 1 | 1.827316 | 7.928001 | -3.185725 |
| 1 | 5.617290 | 6.072218 | -2.368460 |
| 1 | 5.800406 | 3.664367 | -2.205656 |

| | | | |
|---|---|---|---|
| 1 | 4.269338 | 8.122928 | -2.713212 |
| 6 | 2.073913 | -1.230734 | 2.578149 |
| 6 | 2.057345 | -0.540514 | 3.779634 |
| 6 | 2.670135 | -1.123464 | 4.932512 |
| 6 | 2.640954 | -0.501677 | 6.212515 |
| 1 | 2.138805 | 0.453029 | 6.320218 |
| 6 | 3.233839 | -1.100788 | 7.302281 |
| 1 | 3.196246 | -0.612469 | 8.272011 |
| 6 | 3.896026 | -2.346548 | 7.171362 |
| 1 | 4.359989 | -2.805375 | 8.039736 |
| 6 | 3.955570 | -2.966639 | 5.944295 |
| 1 | 4.471296 | -3.916812 | 5.828995 |
| 6 | 3.349746 | -2.377447 | 4.800506 |
| 6 | 3.451074 | -2.969593 | 3.519020 |
| 1 | 4.067776 | -3.856416 | 3.407180 |
| 6 | 2.839871 | -2.425185 | 2.400965 |
| 6 | 3.117588 | -3.035580 | 1.075308 |
| 6 | 3.214256 | -4.407380 | 0.930792 |
| 1 | 2.887179 | -5.055621 | 1.738671 |
| 6 | 3.772548 | -4.997180 | -0.228467 |
| 6 | 3.857296 | -6.408477 | -0.374135 |
| 1 | 3.433244 | -7.037220 | 0.404620 |
| 6 | 4.464891 | -6.970252 | -1.474303 |
| 1 | 4.522819 | -8.050111 | -1.576082 |
| 6 | 5.031775 | -6.136381 | -2.468570 |
| 1 | 5.532154 | -6.582492 | -3.323374 |
| 6 | 4.957975 | -4.764273 | -2.359707 |
| 1 | 5.402473 | -4.134423 | -3.121114 |
| 6 | 4.301255 | -4.150922 | -1.257997 |
| 6 | 4.126119 | -2.731454 | -1.156759 |
| 6 | 3.497891 | -2.217824 | -0.036530 |
| 6 | 1.489649 | 0.843058 | 3.846317 |
| 6 | 0.189399 | 1.056606 | 4.402603 |
| 6 | -0.659849 | -0.024529 | 4.771287 |
| 1 | -0.304048 | -1.039927 | 4.638820 |
| 6 | -1.931255 | 0.204397 | 5.248382 |
| 1 | -2.567293 | -0.638638 | 5.502901 |
| 6 | -2.419620 | 1.526927 | 5.392735 |
| 1 | -3.427444 | 1.694222 | 5.761495 |
| 6 | -1.620483 | 2.595727 | 5.054223 |
| 1 | -1.988581 | 3.614653 | 5.144536 |
| 6 | -0.305291 | 2.393205 | 4.555438 |
| 6 | 0.519568 | 3.473224 | 4.150819 |
| 1 | 0.152862 | 4.491143 | 4.251277 |
| 6 | 1.750232 | 3.236232 | 3.587220 |
| 1 | 2.344408 | 4.081370 | 3.259081 |
| 6 | 2.259722 | 1.921261 | 3.407497 |
| 6 | 4.706024 | -1.811813 | -2.189241 |
| 6 | 5.928274 | -1.125189 | -1.856992 |
| 6 | 6.544274 | -1.223522 | -0.577021 |
| 1 | 6.093110 | -1.852746 | 0.181019 |
| 6 | 7.698053 | -0.529363 | -0.282373 |
| 1 | 8.141963 | -0.619249 | 0.704919 |
| 6 | 8.307302 | 0.302230 | -1.251630 |
| 1 | 9.212235 | 0.849868 | -1.004236 |
| 6 | 7.748973 | 0.409878 | -2.505394 |
| 1 | 8.206361 | 1.042397 | -3.262354 |
| 6 | 6.561442 | -0.295197 | -2.839655 |
| 6 | 5.959028 | -0.169882 | -4.118962 |
| 1 | 6.429930 | 0.467508 | -4.862956 |
| 6 | 4.783857 | -0.816852 | -4.400675 |
| 1 | 4.327756 | -0.688096 | -5.377189 |

| | | | |
|---|---|---|---|
| 6 | 4.133614 | -1.654998 | -3.448676 |
| 8 | 1.713250 | 1.155794 | -0.105652 |
| 8 | 0.924225 | -1.210912 | -0.895297 |
| 8 | 1.271562 | -0.755615 | 1.574845 |
| 8 | 3.307259 | -0.850154 | 0.051803 |
| 15 | 1.723953 | -0.333139 | 0.033729 |
| 6 | 2.843312 | -2.344050 | -3.861882 |
| 6 | 3.060320 | -3.305328 | -5.055226 |
| 6 | 1.717159 | -1.333069 | -4.177155 |
| 1 | 2.495503 | -2.939748 | -3.014416 |
| 6 | 1.758797 | -4.004629 | -5.471367 |
| 1 | 3.450411 | -2.739081 | -5.911991 |
| 1 | 3.821264 | -4.049616 | -4.798381 |
| 6 | 0.425966 | -2.040268 | -4.611898 |
| 1 | 2.042573 | -0.649087 | -4.972390 |
| 1 | 1.516976 | -0.728932 | -3.290524 |
| 6 | 0.659714 | -2.984654 | -5.796980 |
| 1 | 1.947378 | -4.659957 | -6.331224 |
| 1 | 1.417044 | -4.650094 | -4.650785 |
| 1 | -0.334841 | -1.291774 | -4.855115 |
| 1 | 0.028246 | -2.611083 | -3.764436 |
| 1 | -0.271582 | -3.500520 | -6.062910 |
| 1 | 0.959726 | -2.399954 | -6.679441 |
| 6 | 3.634813 | 1.716155 | 2.788628 |
| 6 | 4.746829 | 1.702041 | 3.864118 |
| 6 | 3.997604 | 2.710384 | 1.667527 |
| 1 | 3.643521 | 0.728103 | 2.320286 |
| 6 | 6.101356 | 1.330602 | 3.242359 |
| 1 | 4.806446 | 2.697048 | 4.329111 |
| 1 | 4.490701 | 0.996139 | 4.660724 |
| 6 | 5.345552 | 2.329962 | 1.036112 |
| 1 | 4.070439 | 3.730442 | 2.070464 |
| 1 | 3.213014 | 2.698389 | 0.906857 |
| 6 | 6.467240 | 2.267609 | 2.082431 |
| 1 | 6.886225 | 1.343348 | 4.009526 |
| 1 | 6.042065 | 0.298688 | 2.867661 |
| 1 | 5.601923 | 3.046255 | 0.248004 |
| 1 | 5.246485 | 1.351989 | 0.547413 |
| 1 | 7.403859 | 1.943167 | 1.612521 |
| 1 | 6.646599 | 3.277261 | 2.481024 |
| 1 | -0.981723 | -1.085240 | 0.659459 |
| 1 | -2.244161 | -1.148997 | 1.917788 |
| 1 | -1.191796 | 0.905515 | 2.264812 |
| 1 | -2.565173 | 1.917042 | -0.260814 |
| 1 | -0.481854 | -0.897396 | -1.807049 |
| 8 | -1.313292 | -0.708856 | -2.300827 |
| 1 | -1.084171 | 0.081333 | -2.816559 |

---

**2a'**endo-*si*

---

Number of imaginary frequencies : 0
The smallest frequencies are : 9.3585   10.6687   12.9399  cm(-1)

Electronic energy :        HF=-6614.0185107
Zero-point correction=                            1.891148 (Hartree/Particle)
Thermal correction to Energy=                 2.001841
Thermal correction to Enthalpy=               2.002786
Thermal correction to Gibbs Free Energy=        1.738313
Sum of electronic and zero-point Energies=          -6612.127363
Sum of electronic and thermal Energies=           -6612.016669
Sum of electronic and thermal Enthalpies=          -6612.015725

Sum of electronic and thermal Free Energies= -6612.280197

........................................................

Cartesian Coordinates

........................................................

| | | | |
|---|---|---|---|
| 6 | -4.529979 | -3.851687 | -0.460702 |
| 6 | -3.972477 | -3.774534 | 0.827289 |
| 6 | -4.681115 | -4.335231 | 1.898174 |
| 6 | -5.937385 | -4.914400 | 1.689112 |
| 6 | -6.493383 | -4.958213 | 0.410344 |
| 6 | -5.776342 | -4.436805 | -0.670105 |
| 15 | -2.302614 | -2.966131 | 0.913344 |
| 6 | -1.322162 | -4.300611 | 0.085223 |
| 6 | -1.637243 | -5.647140 | 0.349212 |
| 6 | -0.930675 | -6.675017 | -0.269705 |
| 6 | 0.099075 | -6.373182 | -1.165305 |
| 6 | 0.423084 | -5.042527 | -1.419332 |
| 6 | -0.276966 | -4.003452 | -0.796929 |
| 46 | -2.668975 | -0.777102 | -0.076694 |
| 6 | -1.875215 | 2.317572 | 1.252175 |
| 6 | -1.771094 | 1.033724 | 1.672110 |
| 15 | -4.779890 | 0.078250 | -0.884747 |
| 6 | -1.762965 | -3.114766 | 2.668819 |
| 6 | -0.460426 | -3.556231 | 2.953821 |
| 6 | -0.013264 | -3.646797 | 4.271821 |
| 6 | -0.861197 | -3.312633 | 5.329650 |
| 6 | -2.153405 | -2.859694 | 5.058460 |
| 6 | -2.594430 | -2.737542 | 3.739367 |
| 6 | -5.551145 | -1.074110 | -2.104031 |
| 6 | -4.730134 | -1.635431 | -3.095673 |
| 6 | -5.275634 | -2.466352 | -4.075074 |
| 6 | -6.644613 | -2.746757 | -4.077613 |
| 6 | -7.466352 | -2.192158 | -3.093873 |
| 6 | -6.924414 | -1.361185 | -2.111561 |
| 6 | -4.974439 | 1.692214 | -1.741323 |
| 6 | -6.214669 | 2.090985 | -2.272104 |
| 6 | -6.337449 | 3.320006 | -2.915056 |
| 6 | -5.223160 | 4.156771 | -3.043484 |
| 6 | -3.989488 | 3.760682 | -2.531874 |
| 6 | -3.865355 | 2.532680 | -1.876953 |
| 1 | 0.208523 | -3.828037 | 2.144938 |
| 1 | -3.595563 | -2.363386 | 3.552329 |
| 1 | 1.003365 | -3.969955 | 4.468694 |
| 1 | -2.819688 | -2.589949 | 5.872700 |
| 1 | -0.511646 | -3.389355 | 6.354881 |
| 1 | -2.442805 | -5.894488 | 1.033010 |
| 1 | 0.016671 | -2.977030 | -0.978025 |
| 1 | -1.187293 | -7.709089 | -0.056218 |
| 1 | 1.239125 | -4.808608 | -2.091138 |
| 1 | 0.657360 | -7.168502 | -1.650006 |
| 1 | -4.261807 | -4.342447 | 2.896388 |
| 1 | -3.988813 | -3.446598 | -1.308028 |
| 1 | -6.474832 | -5.338973 | 2.532752 |
| 1 | -6.188288 | -4.464867 | -1.673411 |
| 1 | -7.471561 | -5.404121 | 0.254414 |
| 1 | -3.661700 | -1.448013 | -3.088905 |
| 1 | -7.573225 | -0.946231 | -1.347450 |
| 1 | -4.626852 | -2.898539 | -4.831609 |
| 1 | -8.531228 | -2.407724 | -3.086631 |
| 1 | -7.067158 | -3.395297 | -4.839853 |
| 1 | -7.079573 | 1.440705 | -2.184407 |
| 1 | -2.900775 | 2.243260 | -1.476606 |
| 1 | -7.299300 | 3.623518 | -3.319545 |

| | | | |
|---|---|---|---|
| 1 | -3.116792 | 4.390604 | -2.638624 |
| 1 | -5.317754 | 5.115169 | -3.546234 |
| 6 | -2.922135 | 0.126642 | 1.763639 |
| 6 | -0.811013 | 3.351414 | 1.493092 |
| 6 | -0.516281 | 4.469415 | 0.442000 |
| 1 | 0.136222 | 2.850017 | 1.707762 |
| 1 | -1.093753 | 3.844896 | 2.431069 |
| 6 | 0.220912 | 3.832486 | -0.752075 |
| 1 | 0.454367 | 4.599147 | -1.488944 |
| 1 | 1.160771 | 3.400561 | -0.410544 |
| 6 | -1.753002 | 5.267330 | -0.018651 |
| 6 | -1.655544 | 6.130927 | -1.123365 |
| 6 | -2.962104 | 5.266292 | 0.692372 |
| 6 | -2.726704 | 6.930206 | -1.519081 |
| 1 | -0.729181 | 6.197900 | -1.683117 |
| 6 | -4.041254 | 6.060966 | 0.295938 |
| 1 | -3.075942 | 4.653825 | 1.578776 |
| 6 | -3.932874 | 6.893410 | -0.816046 |
| 1 | -2.616786 | 7.581614 | -2.381711 |
| 1 | -4.966999 | 6.024765 | 0.863757 |
| 1 | -4.771822 | 7.508670 | -1.128048 |
| 6 | 0.441574 | 5.480663 | 1.133127 |
| 6 | -0.053876 | 6.280428 | 2.176937 |
| 6 | 1.792008 | 5.628852 | 0.788201 |
| 6 | 0.765040 | 7.174212 | 2.863150 |
| 1 | -1.101392 | 6.208130 | 2.452387 |
| 6 | 2.616832 | 6.529716 | 1.470562 |
| 1 | 2.235111 | 5.045551 | -0.008420 |
| 6 | 2.112885 | 7.301975 | 2.514882 |
| 1 | 0.348445 | 7.776071 | 3.666451 |
| 1 | 3.659434 | 6.618711 | 1.177839 |
| 1 | 2.755400 | 7.998845 | 3.045549 |
| 7 | -0.544314 | 2.748243 | -1.402780 |
| 1 | -0.258735 | 1.856253 | -0.989834 |
| 16 | -0.309140 | 2.630476 | -3.065934 |
| 8 | -0.989701 | 3.766019 | -3.697982 |
| 8 | -0.722285 | 1.258054 | -3.423196 |
| 6 | -5.996771 | 0.158790 | 0.494785 |
| 6 | -6.463558 | 1.379490 | 1.003131 |
| 6 | -6.346257 | -1.032702 | 1.154811 |
| 6 | -7.272811 | 1.406001 | 2.143041 |
| 1 | -6.198769 | 2.311164 | 0.513651 |
| 6 | -7.158286 | -1.001847 | 2.286522 |
| 1 | -5.984367 | -1.984454 | 0.782971 |
| 6 | -7.622379 | 0.218918 | 2.787055 |
| 1 | -7.628079 | 2.359104 | 2.524865 |
| 1 | -7.423890 | -1.933731 | 2.777686 |
| 1 | -8.251083 | 0.242873 | 3.672675 |
| 6 | 1.478918 | 2.772456 | -3.277765 |
| 6 | 2.162906 | 4.006097 | -3.549893 |
| 6 | 2.161012 | 1.638068 | -2.892855 |
| 6 | 1.544467 | 5.226973 | -3.945266 |
| 6 | 3.595124 | 3.997046 | -3.392255 |
| 6 | 3.560046 | 1.653910 | -2.740539 |
| 1 | 1.615705 | 0.733761 | -2.663540 |
| 6 | 2.292617 | 6.365824 | -4.153959 |
| 1 | 0.473424 | 5.252269 | -4.086139 |
| 6 | 4.329826 | 5.191096 | -3.626187 |
| 6 | 4.259804 | 2.809532 | -2.983067 |
| 1 | 4.057619 | 0.759282 | -2.396760 |
| 6 | 3.696948 | 6.355693 | -3.993426 |
| 1 | 1.792648 | 7.283134 | -4.451868 |

| | | | |
|---|---|---|---|
| 1 | 5.409256 | 5.163480 | -3.501277 |
| 1 | 5.337905 | 2.831685 | -2.848909 |
| 1 | 4.269710 | 7.262676 | -4.163643 |
| 6 | 2.008705 | -1.213431 | 2.517646 |
| 6 | 2.103786 | -0.458691 | 3.674231 |
| 6 | 2.783810 | -1.015447 | 4.809406 |
| 6 | 2.852587 | -0.346735 | 6.064361 |
| 1 | 2.385002 | 0.624681 | 6.170572 |
| 6 | 3.492348 | -0.922955 | 7.140309 |
| 1 | 3.523145 | -0.398294 | 8.091081 |
| 6 | 4.113791 | -2.189644 | 7.019858 |
| 1 | 4.615567 | -2.630030 | 7.876679 |
| 6 | 4.086451 | -2.852833 | 5.814524 |
| 1 | 4.571522 | -3.819426 | 5.702649 |
| 6 | 3.426964 | -2.288790 | 4.688462 |
| 6 | 3.438702 | -2.930345 | 3.427964 |
| 1 | 4.039693 | -3.826581 | 3.307491 |
| 6 | 2.752101 | -2.420602 | 2.341065 |
| 6 | 2.927732 | -3.073198 | 1.020461 |
| 6 | 3.003780 | -4.450208 | 0.928365 |
| 1 | 2.721493 | -5.058151 | 1.783120 |
| 6 | 3.492023 | -5.093637 | -0.231537 |
| 6 | 3.584924 | -6.509734 | -0.309833 |
| 1 | 3.217067 | -7.100486 | 0.525036 |
| 6 | 4.131183 | -7.121252 | -1.415166 |
| 1 | 4.198180 | -8.204219 | -1.465550 |
| 6 | 4.624733 | -6.333236 | -2.483072 |
| 1 | 5.080694 | -6.816397 | -3.342519 |
| 6 | 4.537908 | -4.958382 | -2.439720 |
| 1 | 4.931340 | -4.365348 | -3.256187 |
| 6 | 3.942128 | -4.294899 | -1.331932 |
| 6 | 3.768835 | -2.870510 | -1.288031 |
| 6 | 3.238037 | -2.296965 | -0.143700 |
| 6 | 1.638818 | 0.963950 | 3.753852 |
| 6 | 0.419183 | 1.285026 | 4.429912 |
| 6 | -0.503358 | 0.282107 | 4.837552 |
| 1 | -0.275710 | -0.757014 | 4.629471 |
| 6 | -1.683088 | 0.619106 | 5.462906 |
| 1 | -2.378150 | -0.162035 | 5.753392 |
| 6 | -2.001416 | 1.974643 | 5.723393 |
| 1 | -2.937555 | 2.224551 | 6.215117 |
| 6 | -1.122958 | 2.968721 | 5.354847 |
| 1 | -1.355042 | 4.013127 | 5.549823 |
| 6 | 0.102314 | 2.654619 | 4.706882 |
| 6 | 1.017518 | 3.657618 | 4.302690 |
| 1 | 0.791742 | 4.699205 | 4.509677 |
| 6 | 2.155153 | 3.327101 | 3.607538 |
| 1 | 2.813242 | 4.121468 | 3.275711 |
| 6 | 2.488594 | 1.978128 | 3.304675 |
| 6 | 4.279253 | -2.025266 | -2.416199 |
| 6 | 5.535381 | -1.346803 | -2.220580 |
| 6 | 6.246920 | -1.383607 | -0.987801 |
| 1 | 5.844756 | -1.956440 | -0.160795 |
| 6 | 7.434247 | -0.703195 | -0.825964 |
| 1 | 7.951813 | -0.746676 | 0.127751 |
| 6 | 7.982353 | 0.054415 | -1.888031 |
| 1 | 8.914939 | 0.592586 | -1.744349 |
| 6 | 7.330913 | 0.100383 | -3.099989 |
| 1 | 7.742542 | 0.672686 | -3.927886 |
| 6 | 6.107561 | -0.594853 | -3.299066 |
| 6 | 5.415579 | -0.542137 | -4.536559 |
| 1 | 5.839714 | 0.036487 | -5.353107 |

| | | | |
|---|---|---|---|
| 6 | 4.218791 | -1.192276 | -4.694054 |
| 1 | 3.700510 | -1.124804 | -5.645437 |
| 6 | 3.626362 | -1.953645 | -3.645457 |
| 8 | 1.645440 | 1.205321 | 0.025393 |
| 8 | 0.708024 | -1.001608 | -0.987659 |
| 8 | 1.128124 | -0.833063 | 1.528088 |
| 8 | 3.109905 | -0.917360 | -0.074897 |
| 15 | 1.568719 | -0.285530 | 0.024476 |
| 6 | 2.315460 | -2.670835 | -3.932232 |
| 6 | 2.464369 | -3.710498 | -5.069713 |
| 6 | 1.163193 | -1.691717 | -4.252700 |
| 1 | 2.023731 | -3.209009 | -3.026769 |
| 6 | 1.145677 | -4.445446 | -5.350273 |
| 1 | 2.789131 | -3.200345 | -5.986612 |
| 1 | 3.248915 | -4.430549 | -4.816817 |
| 6 | -0.148495 | -2.433613 | -4.546824 |
| 1 | 1.432389 | -1.070455 | -5.117389 |
| 1 | 1.019766 | -1.021372 | -3.405650 |
| 6 | 0.015492 | -3.459096 | -5.674327 |
| 1 | 1.285423 | -5.158520 | -6.172447 |
| 1 | 0.864648 | -5.035470 | -4.466945 |
| 1 | -0.930490 | -1.706891 | -4.792449 |
| 1 | -0.482822 | -2.944333 | -3.635611 |
| 1 | -0.925232 | -3.998962 | -5.840946 |
| 1 | 0.250595 | -2.936288 | -6.613122 |
| 6 | 3.783504 | 1.674050 | 2.566292 |
| 6 | 4.987507 | 1.561000 | 3.531328 |
| 6 | 4.117685 | 2.663543 | 1.431516 |
| 1 | 3.673425 | 0.697960 | 2.087328 |
| 6 | 6.246274 | 1.104633 | 2.777530 |
| 1 | 5.163839 | 2.540978 | 3.998221 |
| 1 | 4.759195 | 0.862057 | 4.342180 |
| 6 | 5.360441 | 2.191162 | 0.663451 |
| 1 | 4.312885 | 3.663187 | 1.842899 |
| 1 | 3.262484 | 2.736557 | 0.757938 |
| 6 | 6.569774 | 2.031325 | 1.596211 |
| 1 | 7.099307 | 1.048249 | 3.465670 |
| 1 | 6.076476 | 0.085835 | 2.400794 |
| 1 | 5.592797 | 2.895980 | -0.144474 |
| 1 | 5.139971 | 1.229282 | 0.183246 |
| 1 | 7.432004 | 1.647903 | 1.037176 |
| 1 | 6.859099 | 3.018919 | 1.985428 |
| 1 | -2.823560 | -0.594480 | 2.569902 |
| 1 | -3.887426 | 0.627557 | 1.815928 |
| 1 | -0.810929 | 0.669192 | 2.024369 |
| 1 | -2.837441 | 2.652596 | 0.873921 |
| 1 | -0.717874 | -1.081958 | -1.718764 |
| 8 | -1.642822 | -1.048538 | -2.089754 |
| 1 | -1.624939 | -0.215878 | -2.597563 |

---

**2a''*si*(*trans*)**

---

Number of imaginary frequencies : 0
The smallest frequencies are : 9.8889 10.7457 15.5075 cm(-1)

Electronic energy : HF=-6613.9777176
Zero-point correction= 1.888225 (Hartree/Particle)
Thermal correction to Energy= 1.999942
Thermal correction to Enthalpy= 2.000886
Thermal correction to Gibbs Free Energy= 1.733146
Sum of electronic and zero-point Energies= -6612.089492

Sum of electronic and thermal Energies= -6611.977776
Sum of electronic and thermal Enthalpies= -6611.976832
Sum of electronic and thermal Free Energies= -6612.244571
..............................................................
Cartesian Coordinates
..............................................................

| | | | |
|---|---|---|---|
| 6 | 3.019278 | -1.040655 | 1.805634 |
| 6 | 3.906239 | -2.155867 | 2.144462 |
| 6 | 4.530963 | -2.289587 | 3.332425 |
| 1 | 5.195615 | -3.125652 | 3.526592 |
| 1 | 4.376326 | -1.584621 | 4.142778 |
| 1 | 4.053049 | -2.929188 | 1.394061 |
| 15 | 4.872997 | 1.490577 | 1.690920 |
| 15 | 5.986332 | -0.660317 | -1.382023 |
| 6 | 5.541294 | 0.904202 | 3.287544 |
| 6 | 4.923203 | 1.202058 | 4.510715 |
| 6 | 6.658951 | 0.051375 | 3.266883 |
| 6 | 5.431685 | 0.668261 | 5.697052 |
| 1 | 4.052840 | 1.848920 | 4.542224 |
| 6 | 7.175501 | -0.458335 | 4.457194 |
| 1 | 7.127198 | -0.205628 | 2.323696 |
| 6 | 6.559988 | -0.155217 | 5.674150 |
| 1 | 4.945762 | 0.901977 | 6.639802 |
| 1 | 8.046748 | -1.105712 | 4.426425 |
| 1 | 6.954040 | -0.562891 | 6.600530 |
| 6 | 3.606113 | 2.739266 | 2.135590 |
| 6 | 4.011707 | 4.015984 | 2.565638 |
| 6 | 2.241754 | 2.418987 | 2.122871 |
| 6 | 3.062016 | 4.945055 | 2.986243 |
| 1 | 5.064264 | 4.281660 | 2.574926 |
| 6 | 1.290493 | 3.350265 | 2.549978 |
| 1 | 1.903115 | 1.454015 | 1.767698 |
| 6 | 1.703838 | 4.611476 | 2.981682 |
| 1 | 3.383200 | 5.929153 | 3.316011 |
| 1 | 0.238327 | 3.083537 | 2.491519 |
| 1 | 0.966358 | 5.339491 | 3.308451 |
| 6 | 5.555804 | -2.258906 | -2.188902 |
| 6 | 4.196385 | -2.513579 | -2.423911 |
| 6 | 6.512547 | -3.196480 | -2.610437 |
| 6 | 3.791801 | -3.693323 | -3.051851 |
| 1 | 3.435197 | -1.795717 | -2.139153 |
| 6 | 6.105887 | -4.378249 | -3.229630 |
| 1 | 7.569663 | -3.010256 | -2.447179 |
| 6 | 4.746650 | -4.631114 | -3.447034 |
| 1 | 2.732597 | -3.860250 | -3.219612 |
| 1 | 6.850892 | -5.102347 | -3.547300 |
| 1 | 4.437395 | -5.553801 | -3.929750 |
| 6 | 6.591202 | 0.371751 | -2.779064 |
| 6 | 7.893871 | 0.241859 | -3.287697 |
| 6 | 5.707074 | 1.277577 | -3.381476 |
| 6 | 8.313968 | 1.029258 | -4.358832 |
| 1 | 8.587349 | -0.463622 | -2.841545 |
| 6 | 6.130869 | 2.061045 | -4.456796 |
| 1 | 4.690062 | 1.378343 | -3.013597 |
| 6 | 7.434720 | 1.945261 | -4.941093 |
| 1 | 9.326358 | 0.925586 | -4.738685 |
| 1 | 5.436796 | 2.757938 | -4.911916 |
| 1 | 7.762654 | 2.561885 | -5.773084 |
| 1 | 2.785294 | -0.399637 | 2.652299 |
| 6 | 6.219369 | 2.470548 | 0.918176 |
| 6 | 6.049158 | 2.865492 | -0.418230 |
| 6 | 7.390851 | 2.833918 | 1.598241 |

| | | | |
|---|---|---|---|
| 6 | 7.043204 | 3.592087 | -1.071422 |
| 1 | 5.146316 | 2.586826 | -0.952014 |
| 6 | 8.382912 | 3.565161 | 0.941625 |
| 1 | 7.536159 | 2.542511 | 2.632352 |
| 6 | 8.215667 | 3.937877 | -0.393800 |
| 1 | 6.910107 | 3.864198 | -2.113664 |
| 1 | 9.288755 | 3.837683 | 1.475534 |
| 1 | 8.995096 | 4.495149 | -0.905225 |
| 6 | 7.483717 | -1.002430 | -0.364306 |
| 6 | 8.419305 | 0.015766 | -0.115898 |
| 6 | 7.609529 | -2.222038 | 0.322630 |
| 6 | 9.468737 | -0.192742 | 0.780785 |
| 1 | 8.328058 | 0.975107 | -0.613260 |
| 6 | 8.658261 | -2.424765 | 1.220111 |
| 1 | 6.888161 | -3.015883 | 0.158103 |
| 6 | 9.593831 | -1.412300 | 1.449427 |
| 1 | 10.181558 | 0.606327 | 0.961418 |
| 1 | 8.742802 | -3.375112 | 1.739207 |
| 1 | 10.410832 | -1.571624 | 2.147095 |
| 46 | 4.287017 | -0.251565 | 0.319713 |
| 6 | 1.880935 | -1.259044 | 0.838685 |
| 1 | 1.382855 | -0.309692 | 0.649577 |
| 1 | 2.270460 | -1.606784 | -0.133330 |
| 6 | 0.719959 | -2.249739 | 1.259699 |
| 6 | 0.102173 | -1.666853 | 2.542349 |
| 6 | 0.770982 | -1.783253 | 3.776372 |
| 6 | -1.086619 | -0.928097 | 2.516517 |
| 6 | 0.262006 | -1.194698 | 4.933622 |
| 1 | 1.691032 | -2.354625 | 3.837237 |
| 6 | -1.600750 | -0.336102 | 3.673768 |
| 1 | -1.647857 | -0.805313 | 1.603396 |
| 6 | -0.930001 | -0.465723 | 4.887693 |
| 1 | 0.796844 | -1.312964 | 5.872913 |
| 1 | -2.527340 | 0.223002 | 3.594156 |
| 1 | -1.330403 | -0.009997 | 5.789321 |
| 6 | 1.206558 | -3.704450 | 1.407633 |
| 6 | 2.033816 | -4.263572 | 0.414963 |
| 6 | 0.762108 | -4.552366 | 2.430410 |
| 6 | 2.439478 | -5.595518 | 0.469277 |
| 1 | 2.356166 | -3.659894 | -0.427426 |
| 6 | 1.161191 | -5.891494 | 2.486076 |
| 1 | 0.096496 | -4.168485 | 3.193644 |
| 6 | 2.009538 | -6.418802 | 1.513468 |
| 1 | 3.083069 | -5.989125 | -0.313150 |
| 1 | 0.800379 | -6.521102 | 3.295083 |
| 1 | 2.320605 | -7.458701 | 1.558909 |
| 6 | -0.295827 | -2.312813 | 0.061295 |
| 1 | 0.065382 | -3.059778 | -0.642708 |
| 1 | -1.262182 | -2.660299 | 0.430154 |
| 7 | -0.538493 | -1.048994 | -0.652728 |
| 1 | -1.535860 | -0.757874 | -0.595571 |
| 16 | 0.079059 | -0.870096 | -2.175455 |
| 8 | 1.173281 | -1.857557 | -2.340124 |
| 8 | -0.983116 | -0.844570 | -3.192855 |
| 6 | 0.834839 | 0.770283 | -2.172207 |
| 6 | 1.539301 | 1.196979 | -3.345890 |
| 6 | 0.654001 | 1.604965 | -1.093057 |
| 6 | 1.728437 | 0.394759 | -4.506083 |
| 6 | 2.057182 | 2.537234 | -3.353407 |
| 6 | 1.209837 | 2.907735 | -1.105314 |
| 1 | 0.054632 | 1.306289 | -0.239436 |
| 6 | 2.352139 | 0.905487 | -5.623553 |

| | | | |
|---|---|---|---|
| 1 | 1.369966 | -0.626299 | -4.517330 |
| 6 | 2.691154 | 3.029857 | -4.525587 |
| 6 | 1.898101 | 3.360673 | -2.204064 |
| 1 | 1.065778 | 3.541716 | -0.238529 |
| 6 | 2.826380 | 2.237454 | -5.642987 |
| 1 | 2.475530 | 0.278388 | -6.501412 |
| 1 | 3.058996 | 4.052860 | -4.524049 |
| 1 | 2.307530 | 4.367656 | -2.223508 |
| 1 | 3.302349 | 2.628250 | -6.537552 |
| 6 | -5.943342 | 1.635224 | -0.655313 |
| 6 | -5.836526 | 3.006264 | -0.833874 |
| 6 | -7.034461 | 3.789682 | -0.870973 |
| 6 | -7.028655 | 5.183570 | -1.151791 |
| 1 | -6.084318 | 5.663779 | -1.381517 |
| 6 | -8.196314 | 5.915138 | -1.144885 |
| 1 | -8.167581 | 6.978854 | -1.364195 |
| 6 | -9.435379 | 5.290610 | -0.861350 |
| 1 | -10.348384 | 5.879171 | -0.852412 |
| 6 | -9.480247 | 3.937460 | -0.613645 |
| 1 | -10.427868 | 3.443616 | -0.413106 |
| 6 | -8.294808 | 3.151710 | -0.624009 |
| 6 | -8.337194 | 1.750985 | -0.418130 |
| 1 | -9.298805 | 1.281176 | -0.231602 |
| 6 | -7.196069 | 0.970837 | -0.460274 |
| 6 | -7.282062 | -0.512107 | -0.392576 |
| 6 | -8.287364 | -1.185961 | -1.062139 |
| 1 | -9.005274 | -0.624735 | -1.653230 |
| 6 | -8.398183 | -2.597347 | -1.018066 |
| 6 | -9.460730 | -3.278699 | -1.673217 |
| 1 | -10.198499 | -2.691556 | -2.214652 |
| 6 | -9.561064 | -4.650438 | -1.620765 |
| 1 | -10.379081 | -5.158805 | -2.123177 |
| 6 | -8.599939 | -5.401742 | -0.901474 |
| 1 | -8.691981 | -6.483007 | -0.848682 |
| 6 | -7.551650 | -4.772457 | -0.265891 |
| 1 | -6.822844 | -5.350832 | 0.290707 |
| 6 | -7.408910 | -3.358706 | -0.312681 |
| 6 | -6.322598 | -2.676252 | 0.323886 |
| 6 | -6.324793 | -1.290380 | 0.329156 |
| 6 | -4.478010 | 3.621026 | -0.990898 |
| 6 | -3.707642 | 3.314132 | -2.168544 |
| 6 | -4.204633 | 2.492944 | -3.219170 |
| 1 | -5.215888 | 2.109204 | -3.154974 |
| 6 | -3.419000 | 2.159594 | -4.299473 |
| 1 | -3.817576 | 1.510550 | -5.073775 |
| 6 | -2.091836 | 2.639258 | -4.402850 |
| 1 | -1.472029 | 2.346862 | -5.245479 |
| 6 | -1.588085 | 3.464638 | -3.424862 |
| 1 | -0.571356 | 3.839860 | -3.487986 |
| 6 | -2.375179 | 3.822264 | -2.297242 |
| 6 | -1.863554 | 4.660658 | -1.273237 |
| 1 | -0.856000 | 5.055201 | -1.371934 |
| 6 | -2.625044 | 4.957463 | -0.169132 |
| 1 | -2.209332 | 5.593775 | 0.606448 |
| 6 | -3.939562 | 4.433971 | 0.007173 |
| 6 | -5.170176 | -3.415733 | 0.934185 |
| 6 | -4.990996 | -3.399897 | 2.359183 |
| 6 | -5.913823 | -2.769284 | 3.239665 |
| 1 | -6.799977 | -2.302096 | 2.825020 |
| 6 | -5.694209 | -2.733785 | 4.598565 |
| 1 | -6.411076 | -2.237557 | 5.247024 |
| 6 | -4.537791 | -3.328696 | 5.158213 |

| | | | |
|---|---|---|---|
| 1 | -4.369083 | -3.281315 | 6.230479 |
| 6 | -3.633553 | -3.965247 | 4.339484 |
| 1 | -2.741163 | -4.428005 | 4.754351 |
| 6 | -3.837292 | -4.024188 | 2.934767 |
| 6 | -2.919109 | -4.681376 | 2.077336 |
| 1 | -2.043931 | -5.161614 | 2.505137 |
| 6 | -3.123395 | -4.700179 | 0.720927 |
| 1 | -2.397833 | -5.197992 | 0.084596 |
| 6 | -4.240798 | -4.059279 | 0.115089 |
| 8 | -3.669073 | 1.098342 | 1.565150 |
| 8 | -3.142806 | -0.853773 | -0.104987 |
| 8 | -4.807193 | 0.874916 | -0.790029 |
| 8 | -5.424858 | -0.628488 | 1.132239 |
| 15 | -4.101487 | 0.127005 | 0.504571 |
| 6 | -4.339006 | -4.087661 | -1.403315 |
| 6 | -4.507174 | -5.523695 | -1.952454 |
| 6 | -3.133628 | -3.401843 | -2.087270 |
| 1 | -5.227607 | -3.523593 | -1.701649 |
| 6 | -4.659375 | -5.522989 | -3.480942 |
| 1 | -3.628606 | -6.124049 | -1.677352 |
| 1 | -5.375153 | -6.004781 | -1.489371 |
| 6 | -3.283651 | -3.400987 | -3.613932 |
| 1 | -2.208719 | -3.932236 | -1.818208 |
| 1 | -3.039014 | -2.382398 | -1.713921 |
| 6 | -3.477861 | -4.819923 | -4.166086 |
| 1 | -4.759812 | -6.552669 | -3.847708 |
| 1 | -5.592445 | -5.004361 | -3.743878 |
| 1 | -2.409832 | -2.916316 | -4.062421 |
| 1 | -4.153945 | -2.785542 | -3.884301 |
| 1 | -3.627783 | -4.795230 | -5.253190 |
| 1 | -2.562307 | -5.403973 | -3.988665 |
| 6 | -4.666237 | 4.768851 | 1.301620 |
| 6 | -4.910133 | 6.287644 | 1.462525 |
| 6 | -3.929760 | 4.216853 | 2.545264 |
| 1 | -5.647383 | 4.285664 | 1.280661 |
| 6 | -5.692287 | 6.594301 | 2.747678 |
| 1 | -3.944880 | 6.811912 | 1.493855 |
| 1 | -5.450836 | 6.676458 | 0.593752 |
| 6 | -4.708068 | 4.529033 | 3.831337 |
| 1 | -2.928205 | 4.664841 | 2.608947 |
| 1 | -3.792344 | 3.140356 | 2.434133 |
| 6 | -4.980070 | 6.032062 | 3.986215 |
| 1 | -5.840619 | 7.677033 | 2.848189 |
| 1 | -6.692774 | 6.145891 | 2.668934 |
| 1 | -4.158780 | 4.147414 | 4.701066 |
| 1 | -5.665668 | 3.989657 | 3.806896 |
| 1 | -5.574109 | 6.225161 | 4.888412 |
| 1 | -4.023853 | 6.559416 | 4.120731 |
| 1 | -2.102552 | 1.762561 | 1.431009 |
| 8 | -1.272839 | 2.261470 | 1.244286 |
| 1 | -1.563545 | 2.948462 | 0.629050 |

---

***si*[*trans-cis*]‡**

---

Number of imaginary frequencies : 1
The smallest frequencies are : -36.6155 9.5574 14.4221 cm(-1)

Electronic energy : HF=-6613.9623605
Zero-point correction= 1.887490 (Hartree/Particle)
Thermal correction to Energy= 1.998276
Thermal correction to Enthalpy= 1.999220

Thermal correction to Gibbs Free Energy= 1.735277
Sum of electronic and zero-point Energies= -6612.074871
Sum of electronic and thermal Energies= -6611.964085
Sum of electronic and thermal Enthalpies= -6611.963141
Sum of electronic and thermal Free Energies= -6612.227083

............................................................

Cartesian Coordinates

............................................................

| | | | |
|---|---|---|---|
| 6 | 2.798662 | 0.898205 | -2.079452 |
| 6 | 3.363751 | 1.939626 | -2.988819 |
| 6 | 4.642457 | 2.209275 | -3.260229 |
| 1 | 4.903095 | 2.990154 | -3.969268 |
| 1 | 5.464916 | 1.675560 | -2.797318 |
| 1 | 2.591883 | 2.523032 | -3.492605 |
| 15 | 4.935737 | -1.451687 | -1.802137 |
| 15 | 5.774418 | 0.763848 | 1.187499 |
| 6 | 5.910530 | -0.960447 | -3.272148 |
| 6 | 5.325543 | -0.939581 | -4.546976 |
| 6 | 7.203635 | -0.441458 | -3.099332 |
| 6 | 6.029842 | -0.415175 | -5.631398 |
| 1 | 4.321430 | -1.321575 | -4.696940 |
| 6 | 7.903777 | 0.076781 | -4.187380 |
| 1 | 7.663402 | -0.436503 | -2.119129 |
| 6 | 7.317817 | 0.094163 | -5.455456 |
| 1 | 5.566196 | -0.401014 | -6.613298 |
| 1 | 8.903650 | 0.472676 | -4.037090 |
| 1 | 7.861062 | 0.504415 | -6.301794 |
| 6 | 3.685156 | -2.637091 | -2.440707 |
| 6 | 4.125407 | -3.722776 | -3.219819 |
| 6 | 2.321750 | -2.510915 | -2.150755 |
| 6 | 3.208708 | -4.645551 | -3.717306 |
| 1 | 5.182060 | -3.842086 | -3.440017 |
| 6 | 1.400396 | -3.435345 | -2.654857 |
| 1 | 1.961801 | -1.703071 | -1.524673 |
| 6 | 1.845240 | -4.498395 | -3.440016 |
| 1 | 3.558358 | -5.477526 | -4.322056 |
| 1 | 0.350557 | -3.294069 | -2.412621 |
| 1 | 1.131017 | -5.215247 | -3.835588 |
| 6 | 5.590666 | 2.473178 | 1.861010 |
| 6 | 4.289325 | 2.917743 | 2.133638 |
| 6 | 6.680426 | 3.294151 | 2.195305 |
| 6 | 4.075934 | 4.168633 | 2.714445 |
| 1 | 3.433027 | 2.287561 | 1.923809 |
| 6 | 6.461826 | 4.547550 | 2.767387 |
| 1 | 7.697145 | 2.963205 | 2.011033 |
| 6 | 5.160572 | 4.989010 | 3.025343 |
| 1 | 3.059792 | 4.495709 | 2.910804 |
| 1 | 7.311015 | 5.177627 | 3.016991 |
| 1 | 4.995821 | 5.966378 | 3.469738 |
| 6 | 5.879996 | -0.227503 | 2.727143 |
| 6 | 7.031737 | -0.247491 | 3.529415 |
| 6 | 4.726591 | -0.896666 | 3.159231 |
| 6 | 7.037652 | -0.957749 | 4.728933 |
| 1 | 7.923628 | 0.287715 | 3.219253 |
| 6 | 4.731456 | -1.593758 | 4.368024 |
| 1 | 3.821167 | -0.869399 | 2.559072 |
| 6 | 5.888636 | -1.633879 | 5.147556 |
| 1 | 7.935984 | -0.975912 | 5.339438 |
| 1 | 3.834539 | -2.101623 | 4.698169 |
| 1 | 5.890997 | -2.181889 | 6.085532 |
| 1 | 2.370541 | 0.074158 | -2.649297 |
| 6 | 6.018595 | -2.542400 | -0.790895 |

| | | | |
|---|---|---|---|
| 6 | 5.598586 | -2.822230 | 0.518191 |
| 6 | 7.195212 | -3.131987 | -1.274808 |
| 6 | 6.358395 | -3.648363 | 1.344980 |
| 1 | 4.681700 | -2.384776 | 0.897154 |
| 6 | 7.951304 | -3.963436 | -0.446599 |
| 1 | 7.531706 | -2.938606 | -2.287155 |
| 6 | 7.541881 | -4.214871 | 0.865151 |
| 1 | 6.031784 | -3.830986 | 2.364127 |
| 1 | 8.865031 | -4.410241 | -0.828015 |
| 1 | 8.140625 | -4.852432 | 1.509147 |
| 6 | 7.447648 | 0.737667 | 0.426215 |
| 6 | 8.300244 | -0.370519 | 0.548233 |
| 6 | 7.832060 | 1.802016 | -0.409339 |
| 6 | 9.528104 | -0.392001 | -0.117620 |
| 1 | 8.009766 | -1.221002 | 1.154656 |
| 6 | 9.060958 | 1.778499 | -1.067882 |
| 1 | 7.174612 | 2.656312 | -0.538601 |
| 6 | 9.916954 | 0.683752 | -0.918341 |
| 1 | 10.175764 | -1.257024 | -0.011109 |
| 1 | 9.345773 | 2.613929 | -1.700633 |
| 1 | 10.874888 | 0.666084 | -1.429773 |
| 46 | 4.160168 | 0.342753 | -0.586988 |
| 6 | 1.924090 | 1.297173 | -0.914580 |
| 1 | 1.342104 | 0.428647 | -0.616672 |
| 1 | 2.570464 | 1.530023 | -0.005767 |
| 6 | 0.899024 | 2.478354 | -0.979491 |
| 6 | 0.020828 | 2.239532 | -2.224395 |
| 6 | 0.096227 | 3.054361 | -3.365539 |
| 6 | -0.855691 | 1.144280 | -2.257426 |
| 6 | -0.681320 | 2.787019 | -4.494501 |
| 1 | 0.755556 | 3.914956 | -3.375026 |
| 6 | -1.643872 | 0.882787 | -3.378839 |
| 1 | -0.941020 | 0.481638 | -1.407300 |
| 6 | -1.559726 | 1.703975 | -4.503697 |
| 1 | -0.607140 | 3.439461 | -5.360561 |
| 1 | -2.336245 | 0.049167 | -3.341174 |
| 1 | -2.185511 | 1.511310 | -5.370039 |
| 6 | 1.512984 | 3.884075 | -0.939872 |
| 6 | 2.870743 | 4.131900 | -0.716131 |
| 6 | 0.648501 | 4.993277 | -0.998135 |
| 6 | 3.356871 | 5.434788 | -0.570944 |
| 1 | 3.574312 | 3.315270 | -0.647178 |
| 6 | 1.126442 | 6.293568 | -0.852757 |
| 1 | -0.409418 | 4.828208 | -1.164573 |
| 6 | 2.489004 | 6.522446 | -0.639339 |
| 1 | 4.416762 | 5.588176 | -0.388816 |
| 1 | 0.432132 | 7.128148 | -0.902832 |
| 1 | 2.865169 | 7.534995 | -0.523587 |
| 6 | 0.041026 | 2.423546 | 0.355650 |
| 1 | 0.580441 | 3.002712 | 1.104290 |
| 1 | -0.909753 | 2.922648 | 0.163429 |
| 7 | -0.252499 | 1.087692 | 0.900564 |
| 1 | -1.250347 | 0.810806 | 0.770035 |
| 16 | 0.274793 | 0.765871 | 2.444719 |
| 8 | 1.525303 | 1.529015 | 2.655281 |
| 8 | -0.803376 | 0.931012 | 3.433558 |
| 6 | 0.728230 | -0.983418 | 2.418833 |
| 6 | 1.072480 | -1.631597 | 3.653834 |
| 6 | 0.767346 | -1.656058 | 1.219686 |
| 6 | 1.023708 | -1.014993 | 4.934912 |
| 6 | 1.476186 | -3.009302 | 3.581543 |
| 6 | 1.187516 | -3.006863 | 1.168756 |

| | | | |
|---|---|---|---|
| 1 | 0.415032 | -1.189719 | 0.310757 |
| 6 | 1.319625 | -1.728994 | 6.076092 |
| 1 | 0.724540 | 0.020963 | 5.020724 |
| 6 | 1.772770 | -3.713807 | 4.779906 |
| 6 | 1.548258 | -3.661246 | 2.320218 |
| 1 | 1.205344 | -3.512678 | 0.210362 |
| 6 | 1.688023 | -3.092718 | 6.005774 |
| 1 | 1.264116 | -1.236424 | 7.042223 |
| 1 | 2.064712 | -4.758347 | 4.707392 |
| 1 | 1.865344 | -4.700523 | 2.288932 |
| 1 | 1.908340 | -3.642803 | 6.915952 |
| 6 | -5.748334 | -1.736709 | 0.437777 |
| 6 | -5.664411 | -3.119434 | 0.481951 |
| 6 | -6.865811 | -3.887491 | 0.349474 |
| 6 | -6.892659 | -5.299297 | 0.514940 |
| 1 | -5.974660 | -5.809406 | 0.783114 |
| 6 | -8.061276 | -6.011758 | 0.354232 |
| 1 | -8.058799 | -7.089712 | 0.489877 |
| 6 | -9.267787 | -5.349857 | 0.020001 |
| 1 | -10.180802 | -5.923458 | -0.112210 |
| 6 | -9.282298 | -3.980852 | -0.121327 |
| 1 | -10.206480 | -3.459493 | -0.358283 |
| 6 | -8.097018 | -3.215246 | 0.054448 |
| 6 | -8.111156 | -1.802214 | -0.031386 |
| 1 | -9.047220 | -1.302953 | -0.264374 |
| 6 | -6.975299 | -1.045796 | 0.192950 |
| 6 | -7.056793 | 0.436138 | 0.247953 |
| 6 | -8.133038 | 1.040281 | 0.872755 |
| 1 | -8.877943 | 0.421907 | 1.365360 |
| 6 | -8.298075 | 2.445721 | 0.893123 |
| 6 | -9.432450 | 3.052974 | 1.498390 |
| 1 | -10.173603 | 2.413885 | 1.972050 |
| 6 | -9.597717 | 4.419211 | 1.478044 |
| 1 | -10.470306 | 4.872247 | 1.940051 |
| 6 | -8.633223 | 5.237079 | 0.840717 |
| 1 | -8.777626 | 6.313393 | 0.808539 |
| 6 | -7.514914 | 4.679517 | 0.259639 |
| 1 | -6.784434 | 5.310863 | -0.233033 |
| 6 | -7.302098 | 3.273492 | 0.280858 |
| 6 | -6.140744 | 2.662640 | -0.293665 |
| 6 | -6.059993 | 1.280413 | -0.337601 |
| 6 | -4.335511 | -3.780239 | 0.689212 |
| 6 | -3.715795 | -3.697623 | 1.984394 |
| 6 | -4.325404 | -3.029456 | 3.082956 |
| 1 | -5.295740 | -2.566986 | 2.945577 |
| 6 | -3.700410 | -2.952088 | 4.307637 |
| 1 | -4.181808 | -2.422171 | 5.124569 |
| 6 | -2.433715 | -3.549450 | 4.510182 |
| 1 | -1.943166 | -3.470998 | 5.476059 |
| 6 | -1.822593 | -4.219789 | 3.475965 |
| 1 | -0.850998 | -4.682188 | 3.618049 |
| 6 | -2.437767 | -4.307354 | 2.198800 |
| 6 | -1.815940 | -4.983891 | 1.117392 |
| 1 | -0.843639 | -5.443320 | 1.273601 |
| 6 | -2.432514 | -5.056647 | -0.108044 |
| 1 | -1.937861 | -5.579392 | -0.921064 |
| 6 | -3.702933 | -4.458518 | -0.352977 |
| 6 | -5.030297 | 3.507226 | -0.838200 |
| 6 | -4.888403 | 3.649287 | -2.259872 |
| 6 | -5.745039 | 2.988826 | -3.184236 |
| 1 | -6.532988 | 2.347532 | -2.806165 |
| 6 | -5.586171 | 3.148198 | -4.543107 |

| | | | |
|---|---|---|---|
| 1 | -6.251070 | 2.628472 | -5.227539 |
| 6 | -4.563504 | 3.981230 | -5.056998 |
| 1 | -4.447582 | 4.096601 | -6.131202 |
| 6 | -3.718248 | 4.637479 | -4.191752 |
| 1 | -2.924488 | 5.276392 | -4.570169 |
| 6 | -3.856472 | 4.491070 | -2.785990 |
| 6 | -2.998945 | 5.164753 | -1.879808 |
| 1 | -2.219095 | 5.809400 | -2.277902 |
| 6 | -3.149246 | 5.004360 | -0.524675 |
| 1 | -2.477645 | 5.526074 | 0.150924 |
| 6 | -4.165260 | 4.173852 | 0.028593 |
| 8 | -3.342276 | -1.063941 | -1.509052 |
| 8 | -2.862211 | 0.728083 | 0.350834 |
| 8 | -4.634421 | -0.996094 | 0.755066 |
| 8 | -5.051182 | 0.707184 | -1.075958 |
| 15 | -3.808602 | -0.153517 | -0.407125 |
| 6 | -4.264642 | 4.066088 | 1.543112 |
| 6 | -4.619331 | 5.421745 | 2.198569 |
| 6 | -2.984772 | 3.487165 | 2.187785 |
| 1 | -5.074637 | 3.370170 | 1.782817 |
| 6 | -4.779336 | 5.280675 | 3.719074 |
| 1 | -3.822751 | 6.148156 | 1.983442 |
| 1 | -5.539065 | 5.822238 | 1.760234 |
| 6 | -3.138541 | 3.351388 | 3.709124 |
| 1 | -2.132772 | 4.147968 | 1.970893 |
| 1 | -2.768874 | 2.513624 | 1.745156 |
| 6 | -3.519267 | 4.686555 | 4.364256 |
| 1 | -5.017857 | 6.255333 | 4.163893 |
| 1 | -5.636336 | 4.623392 | 3.924502 |
| 1 | -2.213092 | 2.949730 | 4.134003 |
| 1 | -3.921484 | 2.608493 | 3.918268 |
| 1 | -3.668358 | 4.557386 | 5.443885 |
| 1 | -2.687634 | 5.397405 | 4.246078 |
| 6 | -4.305202 | -4.601880 | -1.743189 |
| 6 | -4.657464 | -6.073318 | -2.069566 |
| 6 | -3.400888 | -4.029876 | -2.859198 |
| 1 | -5.236710 | -4.028245 | -1.770021 |
| 6 | -5.340447 | -6.192891 | -3.439429 |
| 1 | -3.735638 | -6.672227 | -2.068496 |
| 1 | -5.304693 | -6.490435 | -1.292820 |
| 6 | -4.081014 | -4.144069 | -4.230869 |
| 1 | -2.450989 | -4.582238 | -2.883868 |
| 1 | -3.166511 | -2.990203 | -2.635327 |
| 6 | -4.473319 | -5.592399 | -4.555150 |
| 1 | -5.569319 | -7.244293 | -3.655260 |
| 1 | -6.303167 | -5.663480 | -3.402028 |
| 1 | -3.419455 | -3.743751 | -5.009288 |
| 1 | -4.981964 | -3.514690 | -4.234610 |
| 1 | -4.998961 | -5.643750 | -5.517055 |
| 1 | -3.560260 | -6.196910 | -4.663018 |
| 1 | -1.829817 | -1.762100 | -1.328403 |
| 8 | -0.986393 | -2.240796 | -1.134495 |
| 1 | -1.254495 | -2.896013 | -0.476069 |

---

**2a''*si*(*cis*)**

---

Number of imaginary frequencies : 0
The smallest frequencies are : 7.7638 10.4742 14.1633 cm(-1)

Electronic energy : HF=-6613.9972362
Zero-point correction= 1.889405 (Hartree/Particle)

Thermal correction to Energy= 2.000944
Thermal correction to Enthalpy= 2.001889
Thermal correction to Gibbs Free Energy= 1.733455
Sum of electronic and zero-point Energies= -6612.107832
Sum of electronic and thermal Energies= -6611.996292
Sum of electronic and thermal Enthalpies= -6611.995348
Sum of electronic and thermal Free Energies= -6612.263781
............................................................

Cartesian Coordinates

............................................................

| | | | |
|---|---|---|---|
| 6 | -3.259058 | 1.744136 | 2.001182 |
| 6 | -4.417424 | 2.530591 | 1.887752 |
| 6 | -4.898130 | 2.953409 | 0.625163 |
| 1 | -5.871779 | 3.431256 | 0.571259 |
| 1 | -4.221317 | 3.221863 | -0.175805 |
| 1 | -5.083240 | 2.582579 | 2.747560 |
| 15 | -4.849479 | -1.439525 | 1.562155 |
| 15 | -6.303365 | 0.657447 | -1.206694 |
| 6 | -5.869078 | -1.206151 | 3.084493 |
| 6 | -5.455273 | -1.680098 | 4.337563 |
| 6 | -7.072475 | -0.482082 | 2.991149 |
| 6 | -6.228031 | -1.431095 | 5.474620 |
| 1 | -4.530327 | -2.238510 | 4.432050 |
| 6 | -7.847845 | -0.250638 | 4.126586 |
| 1 | -7.409380 | -0.101926 | 2.032666 |
| 6 | -7.424464 | -0.719987 | 5.373046 |
| 1 | -5.892021 | -1.798563 | 6.439964 |
| 1 | -8.778920 | 0.300902 | 4.034136 |
| 1 | -8.022552 | -0.530118 | 6.259643 |
| 6 | -3.352048 | -2.322198 | 2.158229 |
| 6 | -3.340268 | -3.720918 | 2.293804 |
| 6 | -2.223109 | -1.591759 | 2.556954 |
| 6 | -2.211746 | -4.365026 | 2.805257 |
| 1 | -4.203979 | -4.308022 | 2.000490 |
| 6 | -1.095960 | -2.236294 | 3.061991 |
| 1 | -2.208903 | -0.517472 | 2.461364 |
| 6 | -1.086913 | -3.626931 | 3.180435 |
| 1 | -2.210708 | -5.447635 | 2.897934 |
| 1 | -0.214552 | -1.661597 | 3.315830 |
| 1 | -0.193556 | -4.127087 | 3.539699 |
| 6 | -6.220157 | 2.150070 | -2.284635 |
| 6 | -4.939898 | 2.580962 | -2.674251 |
| 6 | -7.351804 | 2.791200 | -2.804800 |
| 6 | -4.799656 | 3.650787 | -3.556957 |
| 1 | -4.048888 | 2.079130 | -2.305792 |
| 6 | -7.203742 | 3.869440 | -3.680984 |
| 1 | -8.347667 | 2.454368 | -2.536111 |
| 6 | -5.930930 | 4.302233 | -4.056279 |
| 1 | -3.804505 | 3.969961 | -3.852588 |
| 1 | -8.086678 | 4.364589 | -4.075186 |
| 1 | -5.820167 | 5.138900 | -4.739941 |
| 6 | -6.102825 | -0.642768 | -2.481247 |
| 6 | -7.174612 | -1.144909 | -3.234219 |
| 6 | -4.790740 | -1.003476 | -2.819737 |
| 6 | -6.937114 | -2.034124 | -4.282526 |
| 1 | -8.192334 | -0.840112 | -3.015912 |
| 6 | -4.558036 | -1.867848 | -3.887766 |
| 1 | -3.948768 | -0.603526 | -2.261227 |
| 6 | -5.628933 | -2.396144 | -4.610162 |
| 1 | -7.774079 | -2.429014 | -4.851347 |
| 1 | -3.543523 | -2.130470 | -4.150785 |
| 1 | -5.440541 | -3.078750 | -5.433981 |

| | | | |
|---|---|---|---|
| 1 | -3.088370 | 1.292900 | 2.972434 |
| 6 | -5.713068 | -2.743747 | 0.596042 |
| 6 | -5.066542 | -3.227103 | -0.553843 |
| 6 | -6.963002 | -3.267822 | 0.951078 |
| 6 | -5.669382 | -4.202305 | -1.344596 |
| 1 | -4.086844 | -2.847759 | -0.826436 |
| 6 | -7.566032 | -4.244406 | 0.153190 |
| 1 | -7.470807 | -2.917203 | 1.842815 |
| 6 | -6.925385 | -4.707836 | -0.997057 |
| 1 | -5.162968 | -4.553449 | -2.237976 |
| 1 | -8.536612 | -4.642304 | 0.436030 |
| 1 | -7.398891 | -5.462972 | -1.617833 |
| 6 | -8.000871 | 0.556171 | -0.523064 |
| 6 | -8.656699 | -0.676824 | -0.381760 |
| 6 | -8.565310 | 1.699575 | 0.070851 |
| 6 | -9.861341 | -0.758329 | 0.319370 |
| 1 | -8.223870 | -1.578397 | -0.796414 |
| 6 | -9.770613 | 1.613420 | 0.766104 |
| 1 | -8.062077 | 2.657827 | -0.008866 |
| 6 | -10.423077 | 0.383270 | 0.892254 |
| 1 | -10.353854 | -1.720871 | 0.421928 |
| 1 | -10.197995 | 2.506739 | 1.212092 |
| 1 | -11.360171 | 0.316654 | 1.437010 |
| 46 | -4.839307 | 0.760171 | 0.620525 |
| 6 | -2.106113 | 1.749505 | 1.033620 |
| 1 | -1.770541 | 0.724267 | 0.869184 |
| 1 | -2.430606 | 2.102931 | 0.058022 |
| 6 | -0.807256 | 2.552086 | 1.418710 |
| 6 | -0.140854 | 1.885602 | 2.634971 |
| 6 | -0.758985 | 1.940306 | 3.900868 |
| 6 | 1.066935 | 1.181190 | 2.541101 |
| 6 | -0.202934 | 1.309321 | 5.013235 |
| 1 | -1.666103 | 2.521557 | 4.030919 |
| 6 | 1.631901 | 0.549462 | 3.653742 |
| 1 | 1.610010 | 1.115518 | 1.610886 |
| 6 | 0.997695 | 0.604556 | 4.893233 |
| 1 | -0.704995 | 1.379282 | 5.974855 |
| 1 | 2.567368 | 0.017106 | 3.517561 |
| 1 | 1.436247 | 0.114172 | 5.757959 |
| 6 | -1.115373 | 4.047964 | 1.646273 |
| 6 | -1.885349 | 4.751056 | 0.701800 |
| 6 | -0.592844 | 4.781072 | 2.721038 |
| 6 | -2.147444 | 6.113444 | 0.838932 |
| 1 | -2.272663 | 4.236314 | -0.170617 |
| 6 | -0.851352 | 6.147499 | 2.863677 |
| 1 | 0.031867 | 4.287183 | 3.454135 |
| 6 | -1.635028 | 6.821322 | 1.927967 |
| 1 | -2.746335 | 6.621567 | 0.087918 |
| 1 | -0.428779 | 6.682876 | 3.709672 |
| 1 | -1.836187 | 7.883079 | 2.038507 |
| 6 | 0.090777 | 2.510288 | 0.131034 |
| 1 | -0.347563 | 3.184970 | -0.604869 |
| 1 | 1.080620 | 2.903584 | 0.369232 |
| 7 | 0.271937 | 1.171924 | -0.461226 |
| 1 | 1.270922 | 0.873829 | -0.452196 |
| 16 | -0.395575 | 0.854182 | -1.948064 |
| 8 | -1.719300 | 1.519996 | -2.014785 |
| 8 | 0.543332 | 1.139903 | -3.047259 |
| 6 | -0.663194 | -0.934374 | -1.878642 |
| 6 | -0.951408 | -1.663027 | -3.084905 |
| 6 | -0.649012 | -1.558465 | -0.650296 |
| 6 | -0.925884 | -1.111912 | -4.397186 |

| | | | |
|---|---|---|---|
| 6 | -1.268456 | -3.058727 | -2.947708 |
| 6 | -0.968479 | -2.932269 | -0.538794 |
| 1 | -0.357571 | -1.024029 | 0.241981 |
| 6 | -1.175024 | -1.897273 | -5.502937 |
| 1 | -0.677280 | -0.068163 | -4.533031 |
| 6 | -1.529887 | -3.833686 | -4.109584 |
| 6 | -1.293096 | -3.656767 | -1.657588 |
| 1 | -0.936377 | -3.389229 | 0.442107 |
| 6 | -1.476180 | -3.272088 | -5.365741 |
| 1 | -1.136776 | -1.450927 | -6.492112 |
| 1 | -1.765971 | -4.887515 | -3.985155 |
| 1 | -1.545795 | -4.711166 | -1.577454 |
| 1 | -1.669059 | -3.876777 | -6.247110 |
| 6 | 5.893498 | -1.568814 | -0.656194 |
| 6 | 5.908866 | -2.955149 | -0.661891 |
| 6 | 7.169141 | -3.635457 | -0.679694 |
| 6 | 7.273025 | -5.049187 | -0.794123 |
| 1 | 6.365200 | -5.631890 | -0.898216 |
| 6 | 8.500876 | -5.674573 | -0.786983 |
| 1 | 8.555686 | -6.755886 | -0.877844 |
| 6 | 9.692881 | -4.919404 | -0.667955 |
| 1 | 10.654525 | -5.424612 | -0.656654 |
| 6 | 9.629136 | -3.547343 | -0.579950 |
| 1 | 10.538354 | -2.956025 | -0.503410 |
| 6 | 8.378126 | -2.870992 | -0.597997 |
| 6 | 8.300565 | -1.458723 | -0.550904 |
| 1 | 9.222804 | -0.890402 | -0.471048 |
| 6 | 7.092297 | -0.787337 | -0.610512 |
| 6 | 7.069255 | 0.696440 | -0.681371 |
| 6 | 8.008180 | 1.371664 | -1.440861 |
| 1 | 8.720520 | 0.807343 | -2.035546 |
| 6 | 8.071929 | 2.785519 | -1.469386 |
| 6 | 9.070698 | 3.468852 | -2.216548 |
| 1 | 9.786610 | 2.880828 | -2.785610 |
| 6 | 9.139408 | 4.843404 | -2.214140 |
| 1 | 9.908655 | 5.354198 | -2.786336 |
| 6 | 8.211163 | 5.594965 | -1.452787 |
| 1 | 8.280534 | 6.679112 | -1.437676 |
| 6 | 7.224205 | 4.962722 | -0.728425 |
| 1 | 6.520598 | 5.541978 | -0.141370 |
| 6 | 7.110735 | 3.545315 | -0.726195 |
| 6 | 6.080534 | 2.858416 | -0.006580 |
| 6 | 6.109384 | 1.472932 | 0.038932 |
| 6 | 4.622060 | -3.722742 | -0.683872 |
| 6 | 3.839949 | -3.732410 | -1.891171 |
| 6 | 4.226955 | -3.022863 | -3.062459 |
| 1 | 5.151381 | -2.457935 | -3.054951 |
| 6 | 3.442757 | -3.030898 | -4.194679 |
| 1 | 3.753431 | -2.464252 | -5.067835 |
| 6 | 2.231631 | -3.761333 | -4.228424 |
| 1 | 1.611532 | -3.748406 | -5.119582 |
| 6 | 1.836356 | -4.475159 | -3.121067 |
| 1 | 0.909258 | -5.039432 | -3.133008 |
| 6 | 2.615206 | -4.473294 | -1.934174 |
| 6 | 2.205196 | -5.183021 | -0.775771 |
| 1 | 1.272635 | -5.740976 | -0.802298 |
| 6 | 2.968864 | -5.156585 | 0.364928 |
| 1 | 2.630930 | -5.695800 | 1.244315 |
| 6 | 4.190956 | -4.427642 | 0.439574 |
| 6 | 4.970750 | 3.601996 | 0.671009 |
| 6 | 4.904963 | 3.617796 | 2.106405 |
| 6 | 5.887228 | 2.994354 | 2.925577 |

| | | | |
|---|---|---|---|
| 1 | 6.730668 | 2.503995 | 2.453368 |
| 6 | 5.778161 | 2.995298 | 4.298240 |
| 1 | 6.537859 | 2.502988 | 4.899165 |
| 6 | 4.680898 | 3.624712 | 4.934092 |
| 1 | 4.600372 | 3.608262 | 6.017507 |
| 6 | 3.723445 | 4.257186 | 4.174990 |
| 1 | 2.878251 | 4.750067 | 4.649559 |
| 6 | 3.811979 | 4.276384 | 2.757074 |
| 6 | 2.837474 | 4.928534 | 1.960428 |
| 1 | 2.009708 | 5.436198 | 2.444816 |
| 6 | 2.929181 | 4.909474 | 0.591764 |
| 1 | 2.161426 | 5.403464 | 0.003895 |
| 6 | 3.988743 | 4.242678 | -0.085758 |
| 8 | 3.612152 | -0.930152 | 1.562259 |
| 8 | 2.942806 | 0.789303 | -0.303028 |
| 8 | 4.689301 | -0.927838 | -0.829618 |
| 8 | 5.258698 | 0.824312 | 0.901342 |
| 15 | 3.966330 | -0.066368 | 0.387399 |
| 6 | 3.978406 | 4.250847 | -1.607113 |
| 6 | 4.062673 | 5.682604 | -2.186403 |
| 6 | 2.752363 | 3.513400 | -2.191677 |
| 1 | 4.861212 | 3.711095 | -1.961047 |
| 6 | 4.110657 | 5.659857 | -3.721219 |
| 1 | 3.186566 | 6.261592 | -1.862203 |
| 1 | 4.944364 | 6.197116 | -1.789486 |
| 6 | 2.795403 | 3.493889 | -3.725107 |
| 1 | 1.831680 | 4.018399 | -1.866012 |
| 1 | 2.718313 | 2.495678 | -1.800397 |
| 6 | 2.906476 | 4.908973 | -4.308936 |
| 1 | 4.153202 | 6.684971 | -4.111186 |
| 1 | 5.038587 | 5.163566 | -4.039574 |
| 1 | 1.906602 | 2.979674 | -4.103232 |
| 1 | 3.662953 | 2.900434 | -4.048198 |
| 1 | 2.979213 | 4.870664 | -5.403597 |
| 1 | 1.987897 | 5.468382 | -4.075350 |
| 6 | 4.957619 | -4.452780 | 1.753420 |
| 6 | 5.435649 | -5.876654 | 2.124080 |
| 6 | 4.147843 | -3.847846 | 2.923816 |
| 1 | 5.851185 | -3.831665 | 1.637852 |
| 6 | 6.263185 | -5.867472 | 3.417645 |
| 1 | 4.562386 | -6.530411 | 2.258564 |
| 1 | 6.025616 | -6.301570 | 1.305829 |
| 6 | 4.974356 | -3.841145 | 4.217661 |
| 1 | 3.230145 | -4.431519 | 3.079562 |
| 1 | 3.840958 | -2.835722 | 2.662062 |
| 6 | 5.480197 | -5.244149 | 4.581964 |
| 1 | 6.578311 | -6.888035 | 3.669783 |
| 1 | 7.181621 | -5.288313 | 3.246337 |
| 1 | 4.378305 | -3.423721 | 5.039032 |
| 1 | 5.835005 | -3.170036 | 4.085044 |
| 1 | 6.103549 | -5.207392 | 5.484432 |
| 1 | 4.619672 | -5.886914 | 4.820309 |
| 1 | 2.134355 | -1.850386 | 1.524446 |
| 8 | 1.378477 | -2.473822 | 1.457061 |
| 1 | 1.663229 | -3.090835 | 0.771483 |

---

**2a''***re*(*trans*)

---

Number of imaginary frequencies : 0
The smallest frequencies are : 8.5679 14.0350 16.3570 cm(-1)

Electronic energy : HF=-6613.9763349
Zero-point correction= 1.887943 (Hartree/Particle)
Thermal correction to Energy= 1.999823
Thermal correction to Enthalpy= 2.000767
Thermal correction to Gibbs Free Energy= 1.733772
Sum of electronic and zero-point Energies= -6612.088392
Sum of electronic and thermal Energies= -6611.976512
Sum of electronic and thermal Enthalpies= -6611.975568
Sum of electronic and thermal Free Energies= -6612.242563

..............................................................

Cartesian Coordinates

..............................................................

| | | | |
|---|---|---|---|
| 6 | -3.128136 | 1.586833 | 2.014916 |
| 6 | -2.673475 | 1.153353 | 3.332699 |
| 6 | -3.324742 | 1.434548 | 4.474421 |
| 1 | -2.944178 | 1.101873 | 5.434858 |
| 1 | -4.239921 | 2.022855 | 4.484559 |
| 1 | -1.744953 | 0.594222 | 3.380089 |
| 15 | -4.332331 | -1.496068 | 1.794926 |
| 15 | -5.939058 | 0.367181 | -1.136146 |
| 6 | -5.601074 | -1.653882 | 3.106218 |
| 6 | -6.146923 | -0.493340 | 3.672445 |
| 6 | -6.021867 | -2.910847 | 3.568827 |
| 6 | -7.100986 | -0.585572 | 4.685965 |
| 1 | -5.822644 | 0.477823 | 3.314982 |
| 6 | -6.980656 | -2.999734 | 4.578594 |
| 1 | -5.609892 | -3.817286 | 3.136324 |
| 6 | -7.521531 | -1.838882 | 5.137047 |
| 1 | -7.518237 | 0.319300 | 5.118195 |
| 1 | -7.306052 | -3.975659 | 4.926499 |
| 1 | -8.269377 | -1.911286 | 5.921440 |
| 6 | -2.716130 | -1.841282 | 2.575483 |
| 6 | -2.584633 | -2.150684 | 3.935469 |
| 6 | -1.563225 | -1.601811 | 1.806329 |
| 6 | -1.314406 | -2.192618 | 4.516916 |
| 1 | -3.462568 | -2.326368 | 4.547625 |
| 6 | -0.298448 | -1.642998 | 2.387371 |
| 1 | -1.659903 | -1.337441 | 0.758629 |
| 6 | -0.178381 | -1.922164 | 3.753082 |
| 1 | -1.218139 | -2.413990 | 5.576057 |
| 1 | 0.588032 | -1.415780 | 1.802584 |
| 1 | 0.803014 | -1.910078 | 4.214138 |
| 6 | -6.226230 | 2.123767 | -1.609335 |
| 6 | -5.133725 | 2.821796 | -2.158370 |
| 6 | -7.438558 | 2.794872 | -1.395111 |
| 6 | -5.263716 | 4.167706 | -2.497025 |
| 1 | -4.185800 | 2.317707 | -2.324152 |
| 6 | -7.557595 | 4.146337 | -1.730374 |
| 1 | -8.289733 | 2.267241 | -0.976723 |
| 6 | -6.474655 | 4.833441 | -2.282681 |
| 1 | -4.412688 | 4.696689 | -2.914937 |
| 1 | -8.501517 | 4.658527 | -1.565971 |
| 1 | -6.571555 | 5.883398 | -2.543832 |
| 6 | -5.574877 | -0.467702 | -2.719313 |
| 6 | -6.215865 | -0.101740 | -3.914492 |
| 6 | -4.659393 | -1.528025 | -2.720736 |
| 6 | -5.956139 | -0.807605 | -5.088927 |
| 1 | -6.912002 | 0.731784 | -3.926571 |
| 6 | -4.407248 | -2.233618 | -3.895864 |
| 1 | -4.139377 | -1.798145 | -1.809470 |
| 6 | -5.056808 | -1.877446 | -5.078031 |
| 1 | -6.451938 | -0.519745 | -6.011641 |

| | | | |
|---|---|---|---|
| 1 | -3.685410 | -3.042254 | -3.891418 |
| 1 | -4.847358 | -2.420582 | -5.994892 |
| 1 | -3.894591 | 2.372290 | 2.091326 |
| 6 | -4.661843 | -2.890848 | 0.645436 |
| 6 | -3.618196 | -3.640391 | 0.079731 |
| 6 | -5.984215 | -3.140294 | 0.239423 |
| 6 | -3.894439 | -4.609412 | -0.886143 |
| 1 | -2.590785 | -3.473739 | 0.380690 |
| 6 | -6.252825 | -4.113820 | -0.720930 |
| 1 | -6.803020 | -2.577481 | 0.672463 |
| 6 | -5.208870 | -4.844977 | -1.292864 |
| 1 | -3.075995 | -5.174746 | -1.321759 |
| 1 | -7.278988 | -4.290827 | -1.028152 |
| 1 | -5.419308 | -5.593999 | -2.050522 |
| 6 | -7.579167 | -0.268342 | -0.602156 |
| 6 | -8.394047 | -1.070880 | -1.412717 |
| 6 | -7.984269 | 0.004657 | 0.715058 |
| 6 | -9.587419 | -1.593828 | -0.909514 |
| 1 | -8.092077 | -1.305042 | -2.427764 |
| 6 | -9.177249 | -0.515922 | 1.214789 |
| 1 | -7.349068 | 0.605123 | 1.359080 |
| 6 | -9.979909 | -1.321772 | 0.402891 |
| 1 | -10.207367 | -2.220709 | -1.544165 |
| 1 | -9.465520 | -0.309070 | 2.241155 |
| 1 | -10.903633 | -1.739176 | 0.793092 |
| 46 | -4.350136 | 0.507475 | 0.666544 |
| 6 | -2.123479 | 1.777110 | 0.904218 |
| 1 | -1.459386 | 0.914389 | 0.873615 |
| 1 | -2.632942 | 1.815304 | -0.080638 |
| 6 | -1.159453 | 3.018527 | 0.946132 |
| 6 | -0.284834 | 2.832976 | 2.197730 |
| 6 | -0.586439 | 3.479524 | 3.406037 |
| 6 | 0.755504 | 1.889716 | 2.201125 |
| 6 | 0.123633 | 3.195274 | 4.573912 |
| 1 | -1.397384 | 4.198124 | 3.444893 |
| 6 | 1.465457 | 1.598579 | 3.365626 |
| 1 | 1.015830 | 1.347127 | 1.302406 |
| 6 | 1.147694 | 2.249105 | 4.559034 |
| 1 | -0.135812 | 3.709635 | 5.495838 |
| 1 | 2.268800 | 0.870553 | 3.340355 |
| 1 | 1.705980 | 2.026199 | 5.463428 |
| 6 | -1.872058 | 4.374344 | 0.901394 |
| 6 | -3.195383 | 4.519849 | 0.464087 |
| 6 | -1.137233 | 5.544777 | 1.158515 |
| 6 | -3.778264 | 5.782787 | 0.327872 |
| 1 | -3.786267 | 3.653527 | 0.194087 |
| 6 | -1.712023 | 6.807132 | 1.018978 |
| 1 | -0.104294 | 5.456567 | 1.476857 |
| 6 | -3.042279 | 6.932871 | 0.610660 |
| 1 | -4.805845 | 5.856801 | -0.015927 |
| 1 | -1.118565 | 7.693060 | 1.228312 |
| 1 | -3.494488 | 7.915013 | 0.505141 |
| 6 | -0.319606 | 3.003200 | -0.390675 |
| 1 | -0.902101 | 3.541639 | -1.140286 |
| 1 | 0.599867 | 3.567265 | -0.224813 |
| 7 | 0.039995 | 1.675377 | -0.908688 |
| 1 | 1.052947 | 1.434260 | -0.904400 |
| 16 | -0.645697 | 1.233673 | -2.356774 |
| 8 | -2.085790 | 1.575462 | -2.281247 |
| 8 | 0.083844 | 1.765039 | -3.521868 |
| 6 | -0.468182 | -0.558314 | -2.347869 |
| 6 | -0.851612 | -1.289404 | -3.520610 |

| | | | |
|---|---|---|---|
| 6 | 0.037158 | -1.192797 | -1.233442 |
| 6 | -1.332907 | -0.700530 | -4.723077 |
| 6 | -0.723253 | -2.718787 | -3.469525 |
| 6 | 0.160329 | -2.600357 | -1.205721 |
| 1 | 0.364574 | -0.622442 | -0.377273 |
| 6 | -1.638053 | -1.479527 | -5.817184 |
| 1 | -1.442768 | 0.373638 | -4.790023 |
| 6 | -1.055180 | -3.490021 | -4.616354 |
| 6 | -0.234232 | -3.342733 | -2.291280 |
| 1 | 0.589460 | -3.077075 | -0.331139 |
| 6 | -1.494685 | -2.885869 | -5.771896 |
| 1 | -1.995874 | -1.004872 | -6.725937 |
| 1 | -0.932148 | -4.568472 | -4.566004 |
| 1 | -0.139088 | -4.422110 | -2.277524 |
| 1 | -1.731470 | -3.483081 | -6.647917 |
| 6 | 5.329930 | -1.848303 | -0.534299 |
| 6 | 5.323598 | -3.230035 | -0.426795 |
| 6 | 6.557555 | -3.919941 | -0.180034 |
| 6 | 6.655012 | -5.339566 | -0.175435 |
| 1 | 5.771374 | -5.927554 | -0.394104 |
| 6 | 7.851810 | -5.969064 | 0.091053 |
| 1 | 7.901447 | -7.054383 | 0.085552 |
| 6 | 9.017661 | -5.215697 | 0.369832 |
| 1 | 9.951932 | -5.725160 | 0.587899 |
| 6 | 8.964534 | -3.840841 | 0.346512 |
| 1 | 9.855850 | -3.248777 | 0.539107 |
| 6 | 7.748915 | -3.163366 | 0.055722 |
| 6 | 7.692633 | -1.753755 | -0.036458 |
| 1 | 8.598973 | -1.184408 | 0.147177 |
| 6 | 6.528431 | -1.083422 | -0.364685 |
| 6 | 6.580695 | 0.386052 | -0.561874 |
| 6 | 7.651894 | 0.953364 | -1.228228 |
| 1 | 8.387566 | 0.309170 | -1.700702 |
| 6 | 7.834645 | 2.354734 | -1.292196 |
| 6 | 8.965356 | 2.928772 | -1.935881 |
| 1 | 9.675603 | 2.267373 | -2.425804 |
| 6 | 9.166966 | 4.290018 | -1.928187 |
| 1 | 10.036231 | 4.717965 | -2.419341 |
| 6 | 8.246932 | 5.135180 | -1.261370 |
| 1 | 8.423090 | 6.206838 | -1.234112 |
| 6 | 7.133429 | 4.609625 | -0.643071 |
| 1 | 6.439568 | 5.261209 | -0.125033 |
| 6 | 6.877212 | 3.211051 | -0.658768 |
| 6 | 5.708961 | 2.636404 | -0.058283 |
| 6 | 5.593488 | 1.256573 | -0.009690 |
| 6 | 4.075894 | -4.038318 | -0.609101 |
| 6 | 3.630372 | -4.328260 | -1.943119 |
| 6 | 4.227889 | -3.740052 | -3.092853 |
| 1 | 5.035297 | -3.029183 | -2.960728 |
| 6 | 3.789747 | -4.049767 | -4.361251 |
| 1 | 4.252954 | -3.576428 | -5.222171 |
| 6 | 2.739837 | -4.979292 | -4.553699 |
| 1 | 2.406211 | -5.217991 | -5.559589 |
| 6 | 2.142956 | -5.571612 | -3.464302 |
| 1 | 1.335936 | -6.288595 | -3.598641 |
| 6 | 2.558638 | -5.257029 | -2.142661 |
| 6 | 1.931419 | -5.828498 | -1.005399 |
| 1 | 1.116354 | -6.534240 | -1.150663 |
| 6 | 2.345203 | -5.496542 | 0.260614 |
| 1 | 1.848871 | -5.943697 | 1.116718 |
| 6 | 3.437072 | -4.610418 | 0.489011 |
| 6 | 4.663065 | 3.513933 | 0.554563 |

| | | | |
|---|---|---|---|
| 6 | 4.585657 | 3.585308 | 1.987259 |
| 6 | 5.453182 | 2.843645 | 2.839366 |
| 1 | 6.204245 | 2.199227 | 2.395989 |
| 6 | 5.362853 | 2.941776 | 4.211090 |
| 1 | 6.034429 | 2.361600 | 4.837749 |
| 6 | 4.406153 | 3.795610 | 4.810102 |
| 1 | 4.343247 | 3.862486 | 5.892515 |
| 6 | 3.555677 | 4.531069 | 4.017043 |
| 1 | 2.807154 | 5.180356 | 4.462968 |
| 6 | 3.619255 | 4.444778 | 2.600913 |
| 6 | 2.753499 | 5.199037 | 1.768706 |
| 1 | 2.018915 | 5.851993 | 2.232882 |
| 6 | 2.850007 | 5.116722 | 0.401795 |
| 1 | 2.178823 | 5.706923 | -0.215035 |
| 6 | 3.813574 | 4.285077 | -0.238450 |
| 8 | 2.592941 | -0.823355 | 1.087313 |
| 8 | 2.604767 | 0.748480 | -1.009191 |
| 8 | 4.185266 | -1.208984 | -0.956869 |
| 8 | 4.560822 | 0.692182 | 0.711985 |
| 15 | 3.325034 | -0.134348 | -0.032842 |
| 6 | 3.887680 | 4.302477 | -1.758434 |
| 6 | 4.221067 | 5.710883 | -2.306253 |
| 6 | 2.604250 | 3.769278 | -2.431191 |
| 1 | 4.699022 | 3.637237 | -2.068069 |
| 6 | 4.366125 | 5.692536 | -3.834717 |
| 1 | 3.418829 | 6.409174 | -2.029570 |
| 1 | 5.139646 | 6.088825 | -1.845973 |
| 6 | 2.735077 | 3.759517 | -3.960284 |
| 1 | 1.750000 | 4.399861 | -2.147523 |
| 1 | 2.405139 | 2.759441 | -2.074313 |
| 6 | 3.100831 | 5.144737 | -4.510537 |
| 1 | 4.593954 | 6.701467 | -4.201820 |
| 1 | 5.224215 | 5.059312 | -4.101564 |
| 1 | 1.801081 | 3.393893 | -4.397751 |
| 1 | 3.516671 | 3.039638 | -4.242672 |
| 1 | 3.236415 | 5.103579 | -5.598794 |
| 1 | 2.267310 | 5.838288 | -4.324310 |
| 6 | 3.889311 | -4.374666 | 1.922851 |
| 6 | 4.445393 | -5.668509 | 2.564521 |
| 6 | 2.772127 | -3.779692 | 2.810493 |
| 1 | 4.701102 | -3.639980 | 1.909388 |
| 6 | 4.945747 | -5.418115 | 3.993380 |
| 1 | 3.653157 | -6.430697 | 2.585634 |
| 1 | 5.255078 | -6.071251 | 1.948999 |
| 6 | 3.268483 | -3.543248 | 4.245932 |
| 1 | 1.911445 | -4.463054 | 2.832325 |
| 1 | 2.430601 | -2.843619 | 2.363084 |
| 6 | 3.838906 | -4.822135 | 4.872880 |
| 1 | 5.324630 | -6.352106 | 4.427867 |
| 1 | 5.793657 | -4.720075 | 3.955819 |
| 1 | 2.447754 | -3.160361 | 4.865077 |
| 1 | 4.032809 | -2.757797 | 4.230663 |
| 1 | 4.220830 | -4.615047 | 5.880533 |
| 1 | 3.034448 | -5.564286 | 4.987400 |
| 1 | 3.597005 | -0.755824 | 2.647950 |
| 8 | 4.127248 | -0.495975 | 3.427318 |
| 1 | 4.572515 | 0.299691 | 3.105570 |

---

***re*[*trans-cis*]‡**

---

Number of imaginary frequencies : 1

The smallest frequencies are : -46.1826 9.5492 12.0606 cm(-1)

Electronic energy : HF=-6613.9668731
Zero-point correction= 1.887109 (Hartree/Particle)
Thermal correction to Energy= 1.997986
Thermal correction to Enthalpy= 1.998931
Thermal correction to Gibbs Free Energy= 1.733765
Sum of electronic and zero-point Energies= -6612.079764
Sum of electronic and thermal Energies= -6611.968887
Sum of electronic and thermal Enthalpies= -6611.967942
Sum of electronic and thermal Free Energies= -6612.233108

.............................................................

Cartesian Coordinates

.............................................................

| | | | |
|---|---|---|---|
| 6 | 2.815167 | 0.363701 | -2.139448 |
| 6 | 2.064346 | -0.531381 | -3.072233 |
| 6 | 2.207588 | -1.822999 | -3.375010 |
| 1 | 1.561524 | -2.280106 | -4.119993 |
| 1 | 2.938061 | -2.470191 | -2.913292 |
| 1 | 1.277223 | 0.011272 | -3.599603 |
| 15 | 5.190095 | -1.919421 | -1.191453 |
| 15 | 5.644779 | 0.790673 | 1.239378 |
| 6 | 5.562754 | -2.083649 | -2.983401 |
| 6 | 5.831400 | -0.912987 | -3.707479 |
| 6 | 5.617937 | -3.323956 | -3.634687 |
| 6 | 6.158464 | -0.980935 | -5.062093 |
| 1 | 5.768695 | 0.051817 | -3.212532 |
| 6 | 5.929939 | -3.386768 | -4.992840 |
| 1 | 5.399935 | -4.238569 | -3.094515 |
| 6 | 6.203573 | -2.218089 | -5.707522 |
| 1 | 6.362721 | -0.068155 | -5.614185 |
| 1 | 5.956885 | -4.350444 | -5.493027 |
| 1 | 6.444717 | -2.271544 | -6.765153 |
| 6 | 4.091653 | -3.300675 | -0.668675 |
| 6 | 4.556946 | -4.627456 | -0.610424 |
| 6 | 2.763454 | -3.025466 | -0.307605 |
| 6 | 3.696234 | -5.654190 | -0.223867 |
| 1 | 5.591518 | -4.856464 | -0.845430 |
| 6 | 1.904376 | -4.056204 | 0.076171 |
| 1 | 2.380248 | -2.012480 | -0.332431 |
| 6 | 2.369320 | -5.370683 | 0.116100 |
| 1 | 4.063938 | -6.675507 | -0.184488 |
| 1 | 0.877215 | -3.810618 | 0.321028 |
| 1 | 1.702245 | -6.174648 | 0.414642 |
| 6 | 5.298807 | 2.553255 | 1.659747 |
| 6 | 4.026059 | 2.836492 | 2.182018 |
| 6 | 6.203083 | 3.602363 | 1.444149 |
| 6 | 3.655490 | 4.148092 | 2.467046 |
| 1 | 3.323820 | 2.033016 | 2.364044 |
| 6 | 5.826547 | 4.916726 | 1.735508 |
| 1 | 7.195702 | 3.405456 | 1.053752 |
| 6 | 4.554075 | 5.193715 | 2.237878 |
| 1 | 2.661837 | 4.335544 | 2.862394 |
| 1 | 6.532694 | 5.724741 | 1.564806 |
| 1 | 4.264396 | 6.218939 | 2.449655 |
| 6 | 5.474790 | -0.063944 | 2.847277 |
| 6 | 6.140699 | 0.379482 | 4.002227 |
| 6 | 4.582653 | -1.141531 | 2.937453 |
| 6 | 5.954239 | -0.282664 | 5.214819 |
| 1 | 6.789097 | 1.249734 | 3.956681 |
| 6 | 4.390569 | -1.795748 | 4.155119 |
| 1 | 4.012877 | -1.446006 | 2.066611 |

| | | | |
|---|---|---|---|
| 6 | 5.084233 | -1.374843 | 5.290473 |
| 1 | 6.475417 | 0.062259 | 6.103209 |
| 1 | 3.687497 | -2.621170 | 4.215291 |
| 1 | 4.932619 | -1.882387 | 6.238855 |
| 1 | 3.399071 | 1.079907 | -2.734168 |
| 6 | 6.791305 | -2.342365 | -0.388836 |
| 6 | 6.797922 | -2.836695 | 0.925617 |
| 6 | 8.010930 | -2.158148 | -1.056723 |
| 6 | 8.001449 | -3.151357 | 1.554808 |
| 1 | 5.866108 | -2.989640 | 1.456520 |
| 6 | 9.212194 | -2.477679 | -0.423841 |
| 1 | 8.027382 | -1.773556 | -2.070579 |
| 6 | 9.211090 | -2.976820 | 0.879824 |
| 1 | 7.989185 | -3.533577 | 2.571273 |
| 1 | 10.149594 | -2.329259 | -0.951101 |
| 1 | 10.148812 | -3.222885 | 1.369686 |
| 6 | 7.414451 | 0.812605 | 0.745303 |
| 6 | 8.476686 | 0.440327 | 1.576736 |
| 6 | 7.694904 | 1.276997 | -0.552856 |
| 6 | 9.794257 | 0.550899 | 1.125776 |
| 1 | 8.283988 | 0.049322 | 2.568668 |
| 6 | 9.010595 | 1.395045 | -0.996863 |
| 1 | 6.877136 | 1.546657 | -1.216950 |
| 6 | 10.065563 | 1.035272 | -0.153920 |
| 1 | 10.609007 | 0.251440 | 1.778634 |
| 1 | 9.211223 | 1.760250 | -2.000082 |
| 1 | 11.092149 | 1.122670 | -0.497780 |
| 46 | 4.225386 | 0.062217 | -0.581755 |
| 6 | 2.006179 | 1.006337 | -1.035473 |
| 1 | 1.274622 | 0.286719 | -0.669200 |
| 1 | 2.657713 | 1.218750 | -0.123009 |
| 6 | 1.246942 | 2.360662 | -1.227904 |
| 6 | 0.319304 | 2.159966 | -2.435803 |
| 6 | 0.713291 | 2.553064 | -3.725521 |
| 6 | -0.878542 | 1.451106 | -2.299080 |
| 6 | -0.064565 | 2.232317 | -4.839039 |
| 1 | 1.639036 | 3.102355 | -3.863387 |
| 6 | -1.662657 | 1.131352 | -3.408508 |
| 1 | -1.209344 | 1.123200 | -1.326445 |
| 6 | -1.253308 | 1.514012 | -4.685221 |
| 1 | 0.260293 | 2.545406 | -5.827951 |
| 1 | -2.583387 | 0.581630 | -3.252349 |
| 1 | -1.863186 | 1.271781 | -5.549848 |
| 6 | 2.124708 | 3.602907 | -1.414494 |
| 6 | 3.520877 | 3.607642 | -1.325728 |
| 6 | 1.479861 | 4.835952 | -1.622751 |
| 6 | 4.250860 | 4.794626 | -1.443210 |
| 1 | 4.069459 | 2.690913 | -1.152027 |
| 6 | 2.201260 | 6.022159 | -1.733328 |
| 1 | 0.398283 | 4.855758 | -1.707745 |
| 6 | 3.596198 | 6.007437 | -1.646066 |
| 1 | 5.332692 | 4.763850 | -1.357129 |
| 1 | 1.673374 | 6.959093 | -1.890166 |
| 1 | 4.162530 | 6.930449 | -1.732185 |
| 6 | 0.447409 | 2.622927 | 0.121672 |
| 1 | 1.099134 | 3.199672 | 0.783182 |
| 1 | -0.414768 | 3.253881 | -0.103580 |
| 7 | -0.022911 | 1.441968 | 0.841473 |
| 1 | -1.024695 | 1.176240 | 0.794451 |
| 16 | 0.728797 | 1.033039 | 2.267367 |
| 8 | 1.974097 | 0.285236 | 1.963298 |
| 8 | 0.926997 | 2.236524 | 3.107474 |

| | | | |
|---|---|---|---|
| 6 | -0.459029 | -0.123697 | 2.950839 |
| 6 | -1.169861 | 0.069597 | 4.178506 |
| 6 | -0.667338 | -1.225041 | 2.143615 |
| 6 | -0.964091 | 1.133238 | 5.101843 |
| 6 | -2.159708 | -0.924601 | 4.501505 |
| 6 | -1.639439 | -2.184676 | 2.486717 |
| 1 | -0.114325 | -1.337031 | 1.218162 |
| 6 | -1.711899 | 1.219662 | 6.256110 |
| 1 | -0.209058 | 1.876133 | 4.888815 |
| 6 | -2.914192 | -0.794518 | 5.699112 |
| 6 | -2.374625 | -2.028786 | 3.636952 |
| 1 | -1.817662 | -3.022200 | 1.823563 |
| 6 | -2.702293 | 0.256623 | 6.559865 |
| 1 | -1.537298 | 2.044142 | 6.941795 |
| 1 | -3.663506 | -1.550948 | 5.917194 |
| 1 | -3.139182 | -2.750326 | 3.900622 |
| 1 | -3.285544 | 0.347088 | 7.471837 |
| 6 | -5.490368 | -1.793823 | 0.064865 |
| 6 | -5.523383 | -3.179485 | 0.047114 |
| 6 | -6.712121 | -3.844312 | -0.403758 |
| 6 | -6.848475 | -5.261354 | -0.384662 |
| 1 | -6.028951 | -5.862999 | -0.008560 |
| 6 | -7.996425 | -5.870580 | -0.842467 |
| 1 | -8.075950 | -6.953868 | -0.820932 |
| 6 | -9.073808 | -5.098810 | -1.341267 |
| 1 | -9.970869 | -5.592422 | -1.703992 |
| 6 | -8.981636 | -3.726005 | -1.358441 |
| 1 | -9.805259 | -3.120579 | -1.728724 |
| 6 | -7.814344 | -3.067407 | -0.883635 |
| 6 | -7.723403 | -1.656375 | -0.851182 |
| 1 | -8.561821 | -1.070321 | -1.216387 |
| 6 | -6.605561 | -1.005415 | -0.364377 |
| 6 | -6.619188 | 0.474728 | -0.255359 |
| 6 | -7.747705 | 1.121040 | 0.212231 |
| 1 | -8.578153 | 0.534637 | 0.594910 |
| 6 | -7.863049 | 2.531193 | 0.186479 |
| 6 | -9.044681 | 3.187206 | 0.628013 |
| 1 | -9.853737 | 2.583249 | 1.031397 |
| 6 | -9.170468 | 4.554656 | 0.535507 |
| 1 | -10.078906 | 5.045386 | 0.872956 |
| 6 | -8.117237 | 5.322652 | -0.017047 |
| 1 | -8.228751 | 6.399187 | -0.111885 |
| 6 | -6.953556 | 4.716771 | -0.438981 |
| 1 | -6.156865 | 5.311280 | -0.870255 |
| 6 | -6.776888 | 3.309766 | -0.329732 |
| 6 | -5.566912 | 2.652633 | -0.732010 |
| 6 | -5.516808 | 1.268522 | -0.698443 |
| 6 | -4.397085 | -4.007524 | 0.588670 |
| 6 | -4.450626 | -4.372082 | 1.975963 |
| 6 | -5.460250 | -3.883530 | 2.853578 |
| 1 | -6.215322 | -3.210600 | 2.463438 |
| 6 | -5.484361 | -4.242479 | 4.183752 |
| 1 | -6.263558 | -3.852877 | 4.832862 |
| 6 | -4.497499 | -5.109175 | 4.714411 |
| 1 | -4.524325 | -5.380679 | 5.765909 |
| 6 | -3.506027 | -5.600229 | 3.894845 |
| 1 | -2.739720 | -6.262215 | 4.290718 |
| 6 | -3.457964 | -5.252849 | 2.517561 |
| 6 | -2.445358 | -5.743238 | 1.654668 |
| 1 | -1.689179 | -6.413555 | 2.056273 |
| 6 | -2.413253 | -5.371856 | 0.332221 |
| 1 | -1.621539 | -5.756662 | -0.301501 |

| | | | |
|---|---|---|---|
| 6 | -3.381695 | -4.493916 | -0.233556 |
| 6 | -4.410542 | 3.459368 | -1.239200 |
| 6 | -4.197486 | 3.530587 | -2.659057 |
| 6 | -5.005182 | 2.820648 | -3.591139 |
| 1 | -5.797039 | 2.179598 | -3.221439 |
| 6 | -4.803604 | 2.942566 | -4.948774 |
| 1 | -5.437275 | 2.391705 | -5.638472 |
| 6 | -3.780373 | 3.779521 | -5.453527 |
| 1 | -3.630967 | 3.866365 | -6.526150 |
| 6 | -2.971317 | 4.470370 | -4.580750 |
| 1 | -2.170836 | 5.104908 | -4.952293 |
| 6 | -3.155414 | 4.365654 | -3.177064 |
| 6 | -2.341074 | 5.084014 | -2.265004 |
| 1 | -1.548021 | 5.716136 | -2.656961 |
| 6 | -2.560738 | 4.994615 | -0.913113 |
| 1 | -1.931911 | 5.561992 | -0.233410 |
| 6 | -3.607579 | 4.194820 | -0.369009 |
| 8 | -2.577235 | -1.070792 | -1.228401 |
| 8 | -2.685484 | 0.697193 | 0.705553 |
| 8 | -4.411806 | -1.149788 | 0.630175 |
| 8 | -4.425881 | 0.615976 | -1.228687 |
| 15 | -3.362945 | -0.224224 | -0.266383 |
| 6 | -3.823468 | 4.220229 | 1.137121 |
| 6 | -4.244625 | 5.623843 | 1.635553 |
| 6 | -2.586734 | 3.736256 | 1.925197 |
| 1 | -4.639032 | 3.529724 | 1.375105 |
| 6 | -4.514762 | 5.623484 | 3.147260 |
| 1 | -3.444094 | 6.342670 | 1.410258 |
| 1 | -5.134282 | 5.964208 | 1.097459 |
| 6 | -2.842596 | 3.745214 | 3.437744 |
| 1 | -1.727617 | 4.385194 | 1.705150 |
| 1 | -2.335337 | 2.728281 | 1.599491 |
| 6 | -3.295163 | 5.124144 | 3.934483 |
| 1 | -4.801132 | 6.630394 | 3.477278 |
| 1 | -5.373289 | 4.968741 | 3.353822 |
| 1 | -1.936226 | 3.428513 | 3.962201 |
| 1 | -3.612071 | 2.997605 | 3.674860 |
| 1 | -3.520265 | 5.090468 | 5.008092 |
| 1 | -2.470239 | 5.841172 | 3.808414 |
| 6 | -3.288499 | -4.103355 | -1.700884 |
| 6 | -4.112743 | -5.014899 | -2.639387 |
| 6 | -1.843275 | -4.023681 | -2.228432 |
| 1 | -3.696674 | -3.092107 | -1.781095 |
| 6 | -4.103235 | -4.455096 | -4.071711 |
| 1 | -3.683266 | -6.027803 | -2.628067 |
| 1 | -5.142730 | -5.105018 | -2.286309 |
| 6 | -1.823335 | -3.420902 | -3.639062 |
| 1 | -1.402085 | -5.030496 | -2.273748 |
| 1 | -1.228382 | -3.428519 | -1.552893 |
| 6 | -2.676439 | -4.255408 | -4.606830 |
| 1 | -4.670573 | -5.116357 | -4.739049 |
| 1 | -4.624975 | -3.487917 | -4.068841 |
| 1 | -0.790388 | -3.354312 | -4.006165 |
| 1 | -2.206630 | -2.394297 | -3.582843 |
| 1 | -2.706531 | -3.786103 | -5.598473 |
| 1 | -2.204150 | -5.240174 | -4.739791 |
| 1 | -0.914929 | -1.367291 | -0.995867 |
| 8 | 0.027125 | -1.632631 | -0.852965 |
| 1 | 0.427947 | -1.652491 | -1.731997 |

---

**2a''***re*(*cis*)

---

Number of imaginary frequencies : 0
The smallest frequencies are : 9.4803 12.2525 15.9655 cm(-1)

Electronic energy : HF=-6613.9714874
Zero-point correction= 1.887815 (Hartree/Particle)
Thermal correction to Energy= 1.999673
Thermal correction to Enthalpy= 2.000617
Thermal correction to Gibbs Free Energy= 1.733210
Sum of electronic and zero-point Energies= -6612.083672
Sum of electronic and thermal Energies= -6611.971815
Sum of electronic and thermal Enthalpies= -6611.970871
Sum of electronic and thermal Free Energies= -6612.238277
............................................................
Cartesian Coordinates
............................................................

| | | | |
|---|---|---|---|
| 6 | -3.246051 | 1.577165 | 2.168483 |
| 6 | -3.097229 | 1.180783 | 3.560492 |
| 6 | -2.005433 | 0.676113 | 4.161874 |
| 1 | -2.028244 | 0.418200 | 5.215577 |
| 1 | -1.068853 | 0.515918 | 3.644311 |
| 1 | -3.995943 | 1.309865 | 4.162201 |
| 15 | -4.364646 | -1.550095 | 1.763464 |
| 15 | -5.963093 | 0.451238 | -1.108043 |
| 6 | -5.656746 | -1.812207 | 3.037165 |
| 6 | -6.251593 | -0.701709 | 3.652555 |
| 6 | -6.060869 | -3.103865 | 3.413849 |
| 6 | -7.229423 | -0.875933 | 4.632700 |
| 1 | -5.954876 | 0.298281 | 3.353920 |
| 6 | -7.041833 | -3.275090 | 4.390604 |
| 1 | -5.615364 | -3.973099 | 2.940349 |
| 6 | -7.627353 | -2.162693 | 5.000892 |
| 1 | -7.683933 | -0.007812 | 5.101248 |
| 1 | -7.349503 | -4.277578 | 4.673136 |
| 1 | -8.392851 | -2.299007 | 5.759275 |
| 6 | -2.765930 | -1.984675 | 2.536728 |
| 6 | -2.661841 | -2.506987 | 3.832799 |
| 6 | -1.594361 | -1.703786 | 1.812114 |
| 6 | -1.401746 | -2.752814 | 4.383908 |
| 1 | -3.552139 | -2.711360 | 4.417768 |
| 6 | -0.337762 | -1.943923 | 2.365070 |
| 1 | -1.669443 | -1.279058 | 0.817212 |
| 6 | -0.244782 | -2.466481 | 3.658641 |
| 1 | -1.327658 | -3.150427 | 5.392112 |
| 1 | 0.564334 | -1.690274 | 1.815055 |
| 1 | 0.730571 | -2.631567 | 4.101638 |
| 6 | -6.260221 | 2.219919 | -1.532089 |
| 6 | -5.166027 | 2.943627 | -2.043351 |
| 6 | -7.486691 | 2.869872 | -1.334248 |
| 6 | -5.309741 | 4.292588 | -2.364254 |
| 1 | -4.207429 | 2.454881 | -2.194980 |
| 6 | -7.618979 | 4.225015 | -1.649219 |
| 1 | -8.339405 | 2.322454 | -0.945538 |
| 6 | -6.535114 | 4.937023 | -2.166605 |
| 1 | -4.458609 | 4.841207 | -2.755913 |
| 1 | -8.573984 | 4.720303 | -1.497468 |
| 1 | -6.642489 | 5.989257 | -2.414191 |
| 6 | -5.596613 | -0.319699 | -2.723647 |
| 6 | -6.264952 | 0.069752 | -3.896476 |
| 6 | -4.650352 | -1.350369 | -2.776061 |
| 6 | -6.003793 | -0.587107 | -5.098511 |
| 1 | -6.984303 | 0.883055 | -3.869574 |

| | | | |
|---|---|---|---|
| 6 | -4.394817 | -2.006473 | -3.979212 |
| 1 | -4.109070 | -1.635676 | -1.882247 |
| 6 | -5.073816 | -1.629988 | -5.138281 |
| 1 | -6.522267 | -0.282143 | -6.003155 |
| 1 | -3.649692 | -2.793174 | -4.013730 |
| 1 | -4.864341 | -2.135748 | -6.076384 |
| 1 | -4.018857 | 2.356918 | 2.073974 |
| 6 | -4.671571 | -2.867367 | 0.515609 |
| 6 | -3.619029 | -3.562589 | -0.101556 |
| 6 | -5.990441 | -3.104714 | 0.091682 |
| 6 | -3.884302 | -4.471562 | -1.126691 |
| 1 | -2.591882 | -3.399719 | 0.202092 |
| 6 | -6.248124 | -4.018300 | -0.928911 |
| 1 | -6.815835 | -2.578015 | 0.557016 |
| 6 | -5.196320 | -4.700435 | -1.544895 |
| 1 | -3.057867 | -4.992049 | -1.601373 |
| 1 | -7.272338 | -4.185166 | -1.248286 |
| 1 | -5.398302 | -5.402606 | -2.348353 |
| 6 | -7.605509 | -0.209608 | -0.606753 |
| 6 | -8.402238 | -1.008680 | -1.438686 |
| 6 | -8.029128 | 0.036318 | 0.710183 |
| 6 | -9.594692 | -1.554129 | -0.957401 |
| 1 | -8.086914 | -1.223824 | -2.453739 |
| 6 | -9.221932 | -0.505193 | 1.187495 |
| 1 | -7.408034 | 0.634068 | 1.370973 |
| 6 | -10.005869 | -1.307469 | 0.354121 |
| 1 | -10.199524 | -2.178381 | -1.609053 |
| 1 | -9.525332 | -0.317600 | 2.213159 |
| 1 | -10.929017 | -1.741535 | 0.727142 |
| 46 | -4.414227 | 0.511426 | 0.739558 |
| 6 | -2.115615 | 1.705206 | 1.177984 |
| 1 | -1.458666 | 0.842172 | 1.260232 |
| 1 | -2.545099 | 1.667952 | 0.156951 |
| 6 | -1.151003 | 2.952927 | 1.169796 |
| 6 | -0.229534 | 2.794231 | 2.387626 |
| 6 | -0.516969 | 3.418263 | 3.611150 |
| 6 | 0.849133 | 1.895442 | 2.347538 |
| 6 | 0.247382 | 3.159881 | 4.750859 |
| 1 | -1.356232 | 4.101312 | 3.683046 |
| 6 | 1.607245 | 1.622903 | 3.486234 |
| 1 | 1.098857 | 1.366212 | 1.437770 |
| 6 | 1.307061 | 2.255591 | 4.693935 |
| 1 | 0.000176 | 3.658387 | 5.684779 |
| 1 | 2.433770 | 0.922695 | 3.429565 |
| 1 | 1.904517 | 2.049597 | 5.577037 |
| 6 | -1.856701 | 4.313895 | 1.112111 |
| 6 | -3.154135 | 4.464158 | 0.601588 |
| 6 | -1.136687 | 5.483735 | 1.409526 |
| 6 | -3.732992 | 5.726626 | 0.450048 |
| 1 | -3.723683 | 3.601332 | 0.280357 |
| 6 | -1.705992 | 6.747022 | 1.250780 |
| 1 | -0.118449 | 5.398383 | 1.771216 |
| 6 | -3.014912 | 6.875396 | 0.781491 |
| 1 | -4.741024 | 5.802178 | 0.053391 |
| 1 | -1.122661 | 7.631428 | 1.492589 |
| 1 | -3.463439 | 7.857801 | 0.663398 |
| 6 | -0.359135 | 2.907587 | -0.197489 |
| 1 | -0.961018 | 3.447218 | -0.931056 |
| 1 | 0.571859 | 3.463476 | -0.074136 |
| 7 | -0.035550 | 1.567715 | -0.717600 |
| 1 | 0.982235 | 1.344077 | -0.769097 |
| 16 | -0.756087 | 1.168170 | -2.166250 |

| | | | |
|---|---|---|---|
| 8 | -2.211349 | 1.412711 | -2.018331 |
| 8 | -0.111558 | 1.819348 | -3.321220 |
| 6 | -0.479899 | -0.608017 | -2.264189 |
| 6 | -0.833588 | -1.296546 | -3.472105 |
| 6 | 0.029825 | -1.278078 | -1.172639 |
| 6 | -1.290048 | -0.668899 | -4.664775 |
| 6 | -0.696515 | -2.726853 | -3.469904 |
| 6 | 0.168445 | -2.683784 | -1.196598 |
| 1 | 0.344122 | -0.735553 | -0.293476 |
| 6 | -1.570751 | -1.411487 | -5.790522 |
| 1 | -1.396224 | 0.407002 | -4.700145 |
| 6 | -1.010374 | -3.460250 | -4.646237 |
| 6 | -0.216274 | -3.389808 | -2.309385 |
| 1 | 0.593479 | -3.189987 | -0.337113 |
| 6 | -1.430090 | -2.818439 | -5.788913 |
| 1 | -1.907694 | -0.906020 | -6.690619 |
| 1 | -0.885108 | -4.539471 | -4.629657 |
| 1 | -0.116163 | -4.468668 | -2.332366 |
| 1 | -1.650877 | -3.386559 | -6.688117 |
| 6 | 5.336641 | -1.798026 | -0.611069 |
| 6 | 5.372737 | -3.179413 | -0.501854 |
| 6 | 6.632543 | -3.836333 | -0.301442 |
| 6 | 6.763948 | -5.253165 | -0.284426 |
| 1 | 5.886715 | -5.864595 | -0.460814 |
| 6 | 7.985065 | -5.850513 | -0.057334 |
| 1 | 8.060685 | -6.934311 | -0.051201 |
| 6 | 9.142577 | -5.066264 | 0.165768 |
| 1 | 10.096400 | -5.550901 | 0.353611 |
| 6 | 9.055675 | -3.693388 | 0.127880 |
| 1 | 9.939137 | -3.078074 | 0.279175 |
| 6 | 7.813308 | -3.048860 | -0.121865 |
| 6 | 7.717255 | -1.642151 | -0.223267 |
| 1 | 8.615836 | -1.048460 | -0.084123 |
| 6 | 6.522890 | -1.003498 | -0.502718 |
| 6 | 6.532642 | 0.465404 | -0.708737 |
| 6 | 7.558455 | 1.051529 | -1.428198 |
| 1 | 8.280246 | 0.420330 | -1.938313 |
| 6 | 7.715553 | 2.455808 | -1.495269 |
| 6 | 8.802501 | 3.050326 | -2.193456 |
| 1 | 9.495841 | 2.402420 | -2.723979 |
| 6 | 8.984555 | 4.414353 | -2.186935 |
| 1 | 9.820388 | 4.858081 | -2.720302 |
| 6 | 8.089338 | 5.241740 | -1.466227 |
| 1 | 8.251919 | 6.315576 | -1.439960 |
| 6 | 7.017886 | 4.695985 | -0.793477 |
| 1 | 6.344249 | 5.333478 | -0.232936 |
| 6 | 6.780041 | 3.294184 | -0.807683 |
| 6 | 5.652332 | 2.698989 | -0.151984 |
| 6 | 5.558869 | 1.317345 | -0.106920 |
| 6 | 4.140656 | -4.020174 | -0.632648 |
| 6 | 3.663006 | -4.341685 | -1.947753 |
| 6 | 4.222860 | -3.769152 | -3.124009 |
| 1 | 5.023147 | -3.044566 | -3.026503 |
| 6 | 3.759983 | -4.114153 | -4.374329 |
| 1 | 4.195985 | -3.654090 | -5.256418 |
| 6 | 2.720453 | -5.063670 | -4.520973 |
| 1 | 2.368476 | -5.331362 | -5.513238 |
| 6 | 2.158245 | -5.639792 | -3.404584 |
| 1 | 1.360166 | -6.372456 | -3.503139 |
| 6 | 2.600120 | -5.289173 | -2.100326 |
| 6 | 2.011349 | -5.845510 | -0.935251 |
| 1 | 1.199459 | -6.561254 | -1.044462 |

| | | | |
|---|---|---|---|
| 6 | 2.461326 | -5.490760 | 0.312453 |
| 1 | 1.996917 | -5.930557 | 1.190081 |
| 6 | 3.549090 | -4.588630 | 0.492870 |
| 6 | 4.628701 | 3.556628 | 0.523360 |
| 6 | 4.631615 | 3.619448 | 1.958549 |
| 6 | 5.557825 | 2.888101 | 2.755851 |
| 1 | 6.292593 | 2.257981 | 2.266995 |
| 6 | 5.544499 | 2.978285 | 4.130989 |
| 1 | 6.258862 | 2.404811 | 4.714969 |
| 6 | 4.611051 | 3.814562 | 4.788546 |
| 1 | 4.609324 | 3.875791 | 5.873081 |
| 6 | 3.705505 | 4.540097 | 4.049212 |
| 1 | 2.973559 | 5.175447 | 4.540467 |
| 6 | 3.688490 | 4.460405 | 2.631177 |
| 6 | 2.760938 | 5.199819 | 1.854091 |
| 1 | 2.039770 | 5.833511 | 2.363639 |
| 6 | 2.780646 | 5.125877 | 0.483415 |
| 1 | 2.061671 | 5.702930 | -0.090483 |
| 6 | 3.723710 | 4.318195 | -0.215389 |
| 8 | 2.636695 | -0.804444 | 1.126414 |
| 8 | 2.548413 | 0.746065 | -0.983292 |
| 8 | 4.156264 | -1.189957 | -0.977665 |
| 8 | 4.570281 | 0.733738 | 0.659528 |
| 15 | 3.317745 | -0.119304 | -0.027667 |
| 6 | 3.719600 | 4.351427 | -1.736488 |
| 6 | 4.008731 | 5.770453 | -2.282809 |
| 6 | 2.412042 | 3.810508 | -2.353621 |
| 1 | 4.521924 | 3.699205 | -2.093290 |
| 6 | 4.083604 | 5.771668 | -3.816160 |
| 1 | 3.210460 | 6.454070 | -1.961223 |
| 1 | 4.942328 | 6.156354 | -1.861196 |
| 6 | 2.474220 | 3.817932 | -3.887317 |
| 1 | 1.565187 | 4.428939 | -2.024817 |
| 1 | 2.239088 | 2.794780 | -1.998083 |
| 6 | 2.796428 | 5.213429 | -4.439287 |
| 1 | 4.279850 | 6.788083 | -4.180824 |
| 1 | 4.937526 | 5.154789 | -4.130229 |
| 1 | 1.526955 | 3.443842 | -4.287362 |
| 1 | 3.252472 | 3.112013 | -4.211433 |
| 1 | 2.882827 | 5.184414 | -5.532985 |
| 1 | 1.963156 | 5.893712 | -4.208283 |
| 6 | 4.051967 | -4.328119 | 1.904718 |
| 6 | 4.646716 | -5.605762 | 2.543643 |
| 6 | 2.964589 | -3.732315 | 2.825697 |
| 1 | 4.854347 | -3.585401 | 1.849291 |
| 6 | 5.198567 | -5.327210 | 3.948457 |
| 1 | 3.864543 | -6.375961 | 2.605998 |
| 1 | 5.437165 | -6.008904 | 1.903486 |
| 6 | 3.518631 | -3.459979 | 4.233397 |
| 1 | 2.117850 | -4.429788 | 2.896459 |
| 1 | 2.590001 | -2.810305 | 2.375047 |
| 6 | 4.122983 | -4.722655 | 4.861245 |
| 1 | 5.600700 | -6.250976 | 4.383916 |
| 1 | 6.039440 | -4.624411 | 3.866383 |
| 1 | 2.726465 | -3.059876 | 4.879538 |
| 1 | 4.277617 | -2.672281 | 4.167757 |
| 1 | 4.544688 | -4.493949 | 5.848079 |
| 1 | 3.329933 | -5.468412 | 5.023033 |
| 1 | 3.720562 | -0.702661 | 2.643894 |
| 8 | 4.299375 | -0.426817 | 3.381912 |
| 1 | 4.720225 | 0.363332 | 3.017168 |

---

**2a***

---

Number of imaginary frequencies : 0
The smallest frequencies are : 15.9208 17.5442 18.1516 cm(-1)

Electronic energy : HF=-6613.9991659
Zero-point correction= 1.888458 (Hartree/Particle)
Thermal correction to Energy= 1.999647
Thermal correction to Enthalpy= 2.000591
Thermal correction to Gibbs Free Energy= 1.736896
Sum of electronic and zero-point Energies= -6612.110707
Sum of electronic and thermal Energies= -6611.999519
Sum of electronic and thermal Enthalpies= -6611.998575
Sum of electronic and thermal Free Energies= -6612.262270

Cartesian Coordinates

| | | | |
|---|---|---|---|
| 6 | -3.501850 | -0.153068 | 2.764967 |
| 6 | -4.722868 | -0.604800 | 2.236511 |
| 6 | -5.873424 | -0.550941 | 3.034303 |
| 6 | -5.793830 | -0.070823 | 4.342393 |
| 6 | -4.571594 | 0.355704 | 4.868345 |
| 6 | -3.423949 | 0.319407 | 4.074422 |
| 15 | -4.643260 | -1.316935 | 0.543574 |
| 6 | -6.323987 | -1.347793 | -0.206466 |
| 6 | -7.396419 | -2.033972 | 0.388582 |
| 6 | -8.649607 | -2.049417 | -0.222173 |
| 6 | -8.842023 | -1.409734 | -1.450495 |
| 6 | -7.772011 | -0.765233 | -2.072368 |
| 6 | -6.520867 | -0.735590 | -1.452804 |
| 46 | -3.120322 | -0.035388 | -0.728947 |
| 6 | -1.533326 | 0.710355 | -2.076628 |
| 6 | -1.001182 | -0.323410 | -1.290652 |
| 6 | -1.560060 | -1.613013 | -1.327891 |
| 15 | -4.158642 | 2.108013 | -0.536202 |
| 6 | -5.516732 | 2.355067 | 0.693292 |
| 6 | -5.237579 | 2.819593 | 1.987469 |
| 6 | -6.263858 | 2.965919 | 2.920703 |
| 6 | -7.579182 | 2.646019 | 2.580425 |
| 6 | -7.865033 | 2.178465 | 1.296403 |
| 6 | -6.842641 | 2.035956 | 0.358530 |
| 6 | -4.393370 | -3.117238 | 0.871471 |
| 6 | -4.678999 | -4.039637 | -0.149651 |
| 6 | -4.516217 | -5.407438 | 0.066494 |
| 6 | -4.064017 | -5.873245 | 1.304365 |
| 6 | -3.764290 | -4.962433 | 2.317972 |
| 6 | -3.926709 | -3.591353 | 2.105629 |
| 6 | -4.898204 | 2.745476 | -2.096416 |
| 6 | -5.758101 | 3.858214 | -2.108763 |
| 6 | -6.279532 | 4.335369 | -3.310899 |
| 6 | -5.963563 | 3.700287 | -4.515157 |
| 6 | -5.126062 | 2.583998 | -4.511908 |
| 6 | -4.596729 | 2.109582 | -3.309755 |
| 6 | -2.916151 | 3.349545 | 0.012190 |
| 6 | -1.889659 | 2.902312 | 0.855918 |
| 6 | -0.969645 | 3.805347 | 1.385878 |
| 6 | -1.043746 | 5.158894 | 1.059101 |
| 6 | -2.060400 | 5.611403 | 0.212657 |
| 6 | -3.000037 | 4.715332 | -0.299086 |
| 1 | -2.042554 | -1.929399 | -2.250984 |
| 1 | -1.960700 | 0.477952 | -3.049883 |

| | | | |
|---|---|---|---|
| 1 | -1.166449 | 1.723088 | -1.955156 |
| 1 | -5.042312 | -3.691595 | -1.112712 |
| 1 | -3.694223 | -2.899466 | 2.906639 |
| 1 | -4.738212 | -6.107936 | -0.732819 |
| 1 | -3.398822 | -5.312956 | 3.278494 |
| 1 | -3.938723 | -6.938902 | 1.471911 |
| 1 | -7.249005 | -2.574729 | 1.317943 |
| 1 | -5.694716 | -0.225891 | -1.938587 |
| 1 | -9.473438 | -2.572713 | 0.254514 |
| 1 | -7.905462 | -0.280566 | -3.035269 |
| 1 | -9.818202 | -1.429287 | -1.926445 |
| 1 | -6.834829 | -0.856134 | 2.639870 |
| 1 | -2.598371 | -0.165706 | 2.165272 |
| 1 | -6.694018 | -0.023070 | 4.948506 |
| 1 | -2.461040 | 0.655020 | 4.444427 |
| 1 | -4.518165 | 0.724122 | 5.889024 |
| 1 | -6.032123 | 4.342938 | -1.177409 |
| 1 | -3.948879 | 1.241019 | -3.316826 |
| 1 | -6.938654 | 5.198705 | -3.305918 |
| 1 | -4.881874 | 2.078784 | -5.441805 |
| 1 | -6.373783 | 4.071338 | -5.449901 |
| 1 | -4.221938 | 3.068061 | 2.272714 |
| 1 | -7.085748 | 1.688923 | -0.638319 |
| 1 | -6.028670 | 3.323715 | 3.918416 |
| 1 | -8.884125 | 1.925767 | 1.017460 |
| 1 | -8.374998 | 2.758285 | 3.311305 |
| 1 | -1.792017 | 1.856632 | 1.124637 |
| 1 | -3.784904 | 5.084284 | -0.949882 |
| 1 | -0.193808 | 3.427272 | 2.037905 |
| 1 | -2.120344 | 6.662461 | -0.054907 |
| 1 | -0.304289 | 5.853558 | 1.445906 |
| 1 | -0.308646 | -0.090721 | -0.487488 |
| 1 | 2.699494 | -0.221662 | -2.482438 |
| 6 | 1.213673 | -2.859606 | -1.506005 |
| 6 | -0.003145 | -3.680890 | -1.009441 |
| 1 | 1.509879 | -2.162227 | -0.715060 |
| 1 | 2.051641 | -3.543215 | -1.659078 |
| 7 | 0.874587 | -2.158278 | -2.737156 |
| 16 | 1.913421 | -1.804936 | -3.803074 |
| 8 | 1.341063 | -1.539190 | -5.129864 |
| 8 | 2.810290 | -0.511019 | -3.447824 |
| 6 | -1.070271 | -2.699226 | -0.401496 |
| 1 | -0.609329 | -2.233918 | 0.472870 |
| 1 | -1.918003 | -3.279902 | -0.033971 |
| 6 | -0.548150 | -4.547251 | -2.159908 |
| 6 | -1.914565 | -4.683354 | -2.429139 |
| 6 | 0.348128 | -5.278700 | -2.956904 |
| 6 | -2.373201 | -5.505505 | -3.463905 |
| 1 | -2.643216 | -4.158991 | -1.825065 |
| 6 | -0.101652 | -6.095172 | -3.991111 |
| 1 | 1.414202 | -5.211792 | -2.769799 |
| 6 | -1.469607 | -6.212116 | -4.254637 |
| 1 | -3.441746 | -5.587625 | -3.647904 |
| 1 | 0.620349 | -6.639222 | -4.594261 |
| 1 | -1.822113 | -6.846709 | -5.063051 |
| 6 | 0.435363 | -4.642421 | 0.121031 |
| 6 | 1.756152 | -4.740085 | 0.581767 |
| 6 | -0.525386 | -5.481289 | 0.710058 |
| 6 | 2.103889 | -5.655059 | 1.580331 |
| 1 | 2.536914 | -4.101363 | 0.187570 |
| 6 | -0.186766 | -6.375195 | 1.723297 |
| 1 | -1.550886 | -5.447434 | 0.359892 |

| | | | |
|---|---|---|---|
| 6 | 1.136888 | -6.472454 | 2.162181 |
| 1 | 3.138063 | -5.709945 | 1.907763 |
| 1 | -0.956122 | -7.005605 | 2.161151 |
| 1 | 1.407921 | -7.177095 | 2.943652 |
| 6 | 3.196962 | -3.084600 | -3.997488 |
| 6 | 4.249157 | -3.331940 | -3.054002 |
| 6 | 3.014512 | -3.897740 | -5.098415 |
| 6 | 4.501657 | -2.553160 | -1.888052 |
| 6 | 5.108792 | -4.458745 | -3.309350 |
| 6 | 3.868688 | -4.997265 | -5.331997 |
| 1 | 2.207010 | -3.679286 | -5.787685 |
| 6 | 5.534673 | -2.865409 | -1.031254 |
| 1 | 3.883459 | -1.703368 | -1.645339 |
| 6 | 6.165000 | -4.749619 | -2.401736 |
| 6 | 4.891461 | -5.270391 | -4.454026 |
| 1 | 3.710416 | -5.618714 | -6.208215 |
| 6 | 6.377635 | -3.972363 | -1.287224 |
| 1 | 5.698363 | -2.249709 | -0.151999 |
| 1 | 6.802425 | -5.605449 | -2.609528 |
| 1 | 5.552711 | -6.116285 | -4.624210 |
| 1 | 7.189118 | -4.204370 | -0.603351 |
| 6 | 2.091589 | 3.269759 | 0.114680 |
| 6 | 2.267686 | 4.048474 | -1.014617 |
| 6 | 2.604840 | 5.430115 | -0.856413 |
| 6 | 2.700593 | 6.319273 | -1.963143 |
| 1 | 2.516353 | 5.934513 | -2.960037 |
| 6 | 3.015623 | 7.647322 | -1.776531 |
| 1 | 3.077755 | 8.312203 | -2.633321 |
| 6 | 3.263371 | 8.155241 | -0.477312 |
| 1 | 3.516905 | 9.203389 | -0.346945 |
| 6 | 3.187431 | 7.319427 | 0.613393 |
| 1 | 3.381868 | 7.697125 | 1.614159 |
| 6 | 2.852446 | 5.946295 | 0.456530 |
| 6 | 2.797055 | 5.064160 | 1.562626 |
| 1 | 3.085263 | 5.437166 | 2.541165 |
| 6 | 2.425895 | 3.738317 | 1.424160 |
| 6 | 2.449048 | 2.847319 | 2.611841 |
| 6 | 2.020440 | 3.295374 | 3.849863 |
| 1 | 1.543866 | 4.268184 | 3.930201 |
| 6 | 2.219856 | 2.532836 | 5.027476 |
| 6 | 1.811522 | 3.017814 | 6.301066 |
| 1 | 1.298769 | 3.974981 | 6.353090 |
| 6 | 2.075344 | 2.302440 | 7.446241 |
| 1 | 1.763088 | 2.684298 | 8.413912 |
| 6 | 2.773213 | 1.072821 | 7.366024 |
| 1 | 3.004450 | 0.525403 | 8.275238 |
| 6 | 3.165200 | 0.567662 | 6.145725 |
| 1 | 3.708194 | -0.368231 | 6.091288 |
| 6 | 2.877937 | 1.260856 | 4.938633 |
| 6 | 3.224945 | 0.738189 | 3.649211 |
| 6 | 3.011936 | 1.535580 | 2.536562 |
| 6 | 2.182596 | 3.438981 | -2.379709 |
| 6 | 0.962265 | 3.518743 | -3.121653 |
| 6 | -0.214965 | 4.120409 | -2.592119 |
| 1 | -0.193354 | 4.532018 | -1.589109 |
| 6 | -1.378551 | 4.167542 | -3.328892 |
| 1 | -2.265478 | 4.624949 | -2.904671 |
| 6 | -1.436180 | 3.605514 | -4.627880 |
| 1 | -2.367523 | 3.639063 | -5.185578 |
| 6 | -0.315704 | 3.017741 | -5.169951 |
| 1 | -0.344817 | 2.579137 | -6.164351 |
| 6 | 0.906203 | 2.969051 | -4.444785 |

| | | | |
|---|---|---|---|
| 6 | 2.078412 | 2.391609 | -4.990223 |
| 1 | 2.041026 | 1.964252 | -5.988517 |
| 6 | 3.237121 | 2.332426 | -4.256243 |
| 1 | 4.107431 | 1.853913 | -4.688953 |
| 6 | 3.315095 | 2.830699 | -2.928247 |
| 6 | 3.838107 | -0.620096 | 3.489841 |
| 6 | 5.208199 | -0.707920 | 3.053878 |
| 6 | 6.006070 | 0.438594 | 2.779104 |
| 1 | 5.584933 | 1.425707 | 2.927700 |
| 6 | 7.298451 | 0.320080 | 2.316510 |
| 1 | 7.877588 | 1.214487 | 2.105328 |
| 6 | 7.876756 | -0.954871 | 2.110415 |
| 1 | 8.894263 | -1.035079 | 1.738859 |
| 6 | 7.145319 | -2.086668 | 2.390983 |
| 1 | 7.579370 | -3.074134 | 2.252675 |
| 6 | 5.808448 | -1.995128 | 2.865557 |
| 6 | 5.044739 | -3.154209 | 3.158240 |
| 1 | 5.501524 | -4.132501 | 3.028529 |
| 6 | 3.747449 | -3.042575 | 3.586813 |
| 1 | 3.176247 | -3.942394 | 3.788773 |
| 6 | 3.105100 | -1.780310 | 3.743198 |
| 8 | 2.945238 | 0.101678 | -0.977615 |
| 8 | 1.219404 | -0.266288 | 0.956598 |
| 8 | 1.494481 | 2.034984 | -0.027056 |
| 8 | 3.423440 | 1.073809 | 1.299460 |
| 15 | 2.240204 | 0.588387 | 0.255640 |
| 6 | 1.630197 | -1.782229 | 4.119473 |
| 6 | 1.361698 | -2.336968 | 5.537690 |
| 6 | 0.796426 | -2.589714 | 3.099067 |
| 1 | 1.258547 | -0.754365 | 4.085660 |
| 6 | -0.136348 | -2.282726 | 5.878276 |
| 1 | 1.707539 | -3.379070 | 5.589075 |
| 1 | 1.931977 | -1.777881 | 6.284956 |
| 6 | -0.699346 | -2.530536 | 3.424245 |
| 1 | 1.116041 | -3.636507 | 3.106963 |
| 1 | 0.989881 | -2.201537 | 2.099078 |
| 6 | -0.983716 | -3.038654 | 4.843489 |
| 1 | -0.306210 | -2.688035 | 6.884101 |
| 1 | -0.454244 | -1.230913 | 5.905124 |
| 1 | -1.253914 | -3.125022 | 2.690772 |
| 1 | -1.039704 | -1.493627 | 3.335274 |
| 1 | -2.050867 | -2.939241 | 5.083671 |
| 1 | -0.745004 | -4.111341 | 4.896293 |
| 6 | 4.612675 | 2.694552 | -2.137139 |
| 6 | 5.517401 | 3.945198 | -2.245485 |
| 6 | 5.452999 | 1.445993 | -2.476177 |
| 1 | 4.335887 | 2.596869 | -1.082736 |
| 6 | 6.708506 | 3.837040 | -1.280208 |
| 1 | 5.878861 | 4.032986 | -3.280320 |
| 1 | 4.948234 | 4.853796 | -2.033604 |
| 6 | 6.623033 | 1.307363 | -1.491400 |
| 1 | 5.858166 | 1.532401 | -3.494148 |
| 1 | 4.831981 | 0.551828 | -2.451425 |
| 6 | 7.520718 | 2.553461 | -1.512986 |
| 1 | 7.353508 | 4.719759 | -1.375756 |
| 1 | 6.322927 | 3.839669 | -0.250377 |
| 1 | 7.208647 | 0.410532 | -1.729404 |
| 1 | 6.223269 | 1.154849 | -0.480026 |
| 1 | 8.318071 | 2.467072 | -0.763423 |
| 1 | 8.017354 | 2.619098 | -2.492283 |
| 1 | 0.166689 | 0.418278 | 2.192515 |
| 8 | -0.411041 | 0.845083 | 2.865891 |

| | | | |
|---|---|---|---|
| 1 | 0.193079 | 1.402028 | 3.371851 |

---

**[2a*-3a*]‡**

---

Number of imaginary frequencies : 1
The smallest frequencies are : -176.3516 12.9522 14.5959 cm(-1)

Electronic energy : HF=-6613.9863114
Zero-point correction= 1.886031 (Hartree/Particle)
Thermal correction to Energy= 1.996810
Thermal correction to Enthalpy= 1.997755
Thermal correction to Gibbs Free Energy= 1.733935
Sum of electronic and zero-point Energies= -6612.100281
Sum of electronic and thermal Energies= -6611.989501
Sum of electronic and thermal Enthalpies= -6611.988557
Sum of electronic and thermal Free Energies= -6612.252376

Cartesian Coordinates

| | | | |
|---|---|---|---|
| 6 | -3.554047 | -0.405304 | 2.708459 |
| 6 | -4.759221 | -0.835798 | 2.126595 |
| 6 | -5.930587 | -0.817505 | 2.894686 |
| 6 | -5.889450 | -0.395108 | 4.225308 |
| 6 | -4.683977 | 0.011617 | 4.802236 |
| 6 | -3.514358 | 0.010047 | 4.038491 |
| 15 | -4.638525 | -1.421959 | 0.384927 |
| 6 | -6.347916 | -1.538384 | -0.303574 |
| 6 | -7.324745 | -2.387766 | 0.243729 |
| 6 | -8.595034 | -2.464808 | -0.326397 |
| 6 | -8.899420 | -1.717122 | -1.468008 |
| 6 | -7.925542 | -0.898299 | -2.040866 |
| 6 | -6.657748 | -0.810704 | -1.461172 |
| 46 | -3.196567 | 0.040634 | -0.792773 |
| 6 | -1.415383 | 0.769050 | -1.819055 |
| 6 | -0.989481 | -0.330244 | -1.043667 |
| 6 | -0.922630 | -1.668474 | -1.510454 |
| 15 | -4.242661 | 2.153236 | -0.557132 |
| 6 | -5.644609 | 2.311613 | 0.637408 |
| 6 | -5.411514 | 2.655195 | 1.978169 |
| 6 | -6.464393 | 2.690187 | 2.892684 |
| 6 | -7.763155 | 2.377951 | 2.487641 |
| 6 | -8.004690 | 2.029747 | 1.157144 |
| 6 | -6.954912 | 1.998017 | 0.239578 |
| 6 | -4.289441 | -3.228384 | 0.593353 |
| 6 | -4.250713 | -4.025746 | -0.564115 |
| 6 | -4.051970 | -5.402660 | -0.474485 |
| 6 | -3.884554 | -6.005808 | 0.776482 |
| 6 | -3.899931 | -5.219437 | 1.929126 |
| 6 | -4.097655 | -3.837948 | 1.840765 |
| 6 | -4.898429 | 2.924127 | -2.096160 |
| 6 | -5.818917 | 3.986539 | -2.090232 |
| 6 | -6.271651 | 4.536115 | -3.289950 |
| 6 | -5.816356 | 4.029891 | -4.510646 |
| 6 | -4.908175 | 2.969911 | -4.527222 |
| 6 | -4.450105 | 2.421155 | -3.327357 |
| 6 | -3.022192 | 3.359699 | 0.124328 |
| 6 | -2.016680 | 2.859539 | 0.965683 |
| 6 | -1.084744 | 3.721111 | 1.541959 |
| 6 | -1.129317 | 5.089270 | 1.272675 |
| 6 | -2.128701 | 5.596226 | 0.437067 |
| 6 | -3.076516 | 4.739132 | -0.125957 |

| | | | |
|---|---|---|---|
| 1 | -1.330816 | -1.883462 | -2.492255 |
| 1 | -1.571997 | 0.639846 | -2.889424 |
| 1 | -1.121261 | 1.767916 | -1.518191 |
| 1 | -4.390169 | -3.565670 | -1.539626 |
| 1 | -4.115635 | -3.242937 | 2.747069 |
| 1 | -4.016690 | -6.002493 | -1.378966 |
| 1 | -3.757809 | -5.676426 | 2.904337 |
| 1 | -3.726021 | -7.077848 | 0.847297 |
| 1 | -7.087080 | -3.002993 | 1.106418 |
| 1 | -5.901227 | -0.171941 | -1.907400 |
| 1 | -9.343495 | -3.118727 | 0.112278 |
| 1 | -8.147811 | -0.326550 | -2.937652 |
| 1 | -9.887184 | -1.784980 | -1.915192 |
| 1 | -6.879472 | -1.106543 | 2.458057 |
| 1 | -2.636205 | -0.387113 | 2.130786 |
| 1 | -6.806288 | -0.375848 | 4.807955 |
| 1 | -2.564598 | 0.332536 | 4.453066 |
| 1 | -4.659380 | 0.337352 | 5.838588 |
| 1 | -6.192611 | 4.374648 | -1.148088 |
| 1 | -3.738915 | 1.601331 | -3.342827 |
| 1 | -6.982413 | 5.357580 | -3.271278 |
| 1 | -4.552422 | 2.568217 | -5.471577 |
| 1 | -6.174077 | 4.456010 | -5.443668 |
| 1 | -4.408569 | 2.892646 | 2.314208 |
| 1 | -7.162687 | 1.735553 | -0.790898 |
| 1 | -6.262997 | 2.953135 | 3.926804 |
| 1 | -9.009836 | 1.780746 | 0.828302 |
| 1 | -8.579649 | 2.401997 | 3.203967 |
| 1 | -1.940218 | 1.799488 | 1.182643 |
| 1 | -3.845519 | 5.149007 | -0.772232 |
| 1 | -0.318399 | 3.301350 | 2.180615 |
| 1 | -2.166724 | 6.658920 | 0.214798 |
| 1 | -0.379605 | 5.752230 | 1.693934 |
| 1 | -0.534549 | -0.140514 | -0.078553 |
| 1 | 2.731237 | 0.055022 | -2.171952 |
| 6 | 1.563916 | -2.902926 | -1.258034 |
| 6 | 0.362852 | -3.821920 | -0.933324 |
| 1 | 1.925357 | -2.416696 | -0.347153 |
| 1 | 2.384221 | -3.467606 | -1.700727 |
| 7 | 1.017480 | -1.913211 | -2.163789 |
| 16 | 1.625557 | -1.353671 | -3.472121 |
| 8 | 0.598471 | -0.964664 | -4.446637 |
| 8 | 2.558806 | -0.108551 | -3.209306 |
| 6 | -0.756262 | -2.813116 | -0.535098 |
| 1 | -0.501593 | -2.400431 | 0.442780 |
| 1 | -1.717628 | -3.318780 | -0.425058 |
| 6 | 0.015259 | -4.659039 | -2.183034 |
| 6 | -1.254197 | -4.664103 | -2.773260 |
| 6 | 1.007323 | -5.466657 | -2.763069 |
| 6 | -1.518079 | -5.424422 | -3.916736 |
| 1 | -2.058177 | -4.078285 | -2.346332 |
| 6 | 0.751280 | -6.224399 | -3.903780 |
| 1 | 1.995417 | -5.510026 | -2.315237 |
| 6 | -0.515729 | -6.203181 | -4.492641 |
| 1 | -2.512076 | -5.400630 | -4.355986 |
| 1 | 1.545138 | -6.827808 | -4.334669 |
| 1 | -0.717989 | -6.789559 | -5.384611 |
| 6 | 0.675449 | -4.779938 | 0.221870 |
| 6 | 1.990785 | -5.077315 | 0.606081 |
| 6 | -0.371508 | -5.418103 | 0.901870 |
| 6 | 2.247489 | -5.985478 | 1.636934 |
| 1 | 2.832483 | -4.596716 | 0.119670 |

| | | | |
|---|---|---|---|
| 6 | -0.119051 | -6.313264 | 1.939679 |
| 1 | -1.397032 | -5.219078 | 0.617179 |
| 6 | 1.195667 | -6.604080 | 2.313226 |
| 1 | 3.275665 | -6.199168 | 1.915523 |
| 1 | -0.952330 | -6.783472 | 2.454488 |
| 1 | 1.396680 | -7.303308 | 3.120063 |
| 6 | 2.752462 | -2.548171 | -4.225830 |
| 6 | 4.026659 | -2.894981 | -3.665116 |
| 6 | 2.253830 | -3.177951 | -5.347090 |
| 6 | 4.591071 | -2.319995 | -2.490463 |
| 6 | 4.771585 | -3.925683 | -4.338350 |
| 6 | 3.004945 | -4.182495 | -5.994552 |
| 1 | 1.279289 | -2.895355 | -5.728111 |
| 6 | 5.814338 | -2.738965 | -2.013155 |
| 1 | 4.066092 | -1.555163 | -1.937667 |
| 6 | 6.035244 | -4.322325 | -3.821176 |
| 6 | 4.235860 | -4.543924 | -5.500178 |
| 1 | 2.599025 | -4.663323 | -6.878851 |
| 6 | 6.548914 | -3.744867 | -2.683194 |
| 1 | 6.210883 | -2.287437 | -1.108329 |
| 1 | 6.585765 | -5.100313 | -4.343726 |
| 1 | 4.819835 | -5.318112 | -5.991130 |
| 1 | 7.513455 | -4.060708 | -2.296433 |
| 6 | 2.073781 | 3.222524 | 0.312441 |
| 6 | 2.245393 | 4.064944 | -0.770334 |
| 6 | 2.513923 | 5.452432 | -0.534841 |
| 6 | 2.620774 | 6.397087 | -1.593495 |
| 1 | 2.499087 | 6.056004 | -2.615073 |
| 6 | 2.869657 | 7.726515 | -1.331617 |
| 1 | 2.941377 | 8.433161 | -2.153584 |
| 6 | 3.035400 | 8.182596 | -0.000827 |
| 1 | 3.234507 | 9.233319 | 0.189352 |
| 6 | 2.947843 | 7.293045 | 1.045461 |
| 1 | 3.079794 | 7.629682 | 2.070817 |
| 6 | 2.683885 | 5.916025 | 0.809347 |
| 6 | 2.623329 | 4.980857 | 1.869880 |
| 1 | 2.849027 | 5.321428 | 2.876136 |
| 6 | 2.327936 | 3.646327 | 1.656917 |
| 6 | 2.350441 | 2.710140 | 2.809132 |
| 6 | 1.856249 | 3.081099 | 4.048407 |
| 1 | 1.330173 | 4.025611 | 4.153666 |
| 6 | 2.051419 | 2.274728 | 5.197500 |
| 6 | 1.588465 | 2.687488 | 6.477864 |
| 1 | 1.036288 | 3.620642 | 6.555224 |
| 6 | 1.849981 | 1.934111 | 7.599028 |
| 1 | 1.497279 | 2.262086 | 8.572541 |
| 6 | 2.598689 | 0.737085 | 7.487938 |
| 1 | 2.828096 | 0.161763 | 8.380209 |
| 6 | 3.042937 | 0.300166 | 6.259364 |
| 1 | 3.627116 | -0.608892 | 6.180062 |
| 6 | 2.761647 | 1.033806 | 5.075428 |
| 6 | 3.172945 | 0.584653 | 3.777692 |
| 6 | 2.980519 | 1.434032 | 2.700780 |
| 6 | 2.206129 | 3.545695 | -2.175060 |
| 6 | 1.003449 | 3.674590 | -2.940295 |
| 6 | -0.203046 | 4.187255 | -2.383849 |
| 1 | -0.223381 | 4.480975 | -1.340022 |
| 6 | -1.344072 | 4.295422 | -3.148100 |
| 1 | -2.255423 | 4.678045 | -2.702205 |
| 6 | -1.345017 | 3.895704 | -4.506695 |
| 1 | -2.256669 | 3.984330 | -5.089475 |
| 6 | -0.196838 | 3.393406 | -5.074785 |

| | | | |
|---|---|---|---|
| 1 | -0.184617 | 3.078743 | -6.115538 |
| 6 | 0.999812 | 3.276322 | -4.317216 |
| 6 | 2.200450 | 2.785667 | -4.885082 |
| 1 | 2.205194 | 2.487198 | -5.930370 |
| 6 | 3.336260 | 2.662858 | -4.124710 |
| 1 | 4.230957 | 2.256219 | -4.581593 |
| 6 | 3.365307 | 3.017903 | -2.748978 |
| 6 | 3.813074 | -0.752958 | 3.562638 |
| 6 | 5.185096 | -0.795243 | 3.126281 |
| 6 | 5.972466 | 0.376701 | 2.943963 |
| 1 | 5.543430 | 1.344208 | 3.176509 |
| 6 | 7.263106 | 0.308134 | 2.467663 |
| 1 | 7.833927 | 1.221907 | 2.328617 |
| 6 | 7.851079 | -0.941196 | 2.156597 |
| 1 | 8.867468 | -0.981789 | 1.775308 |
| 6 | 7.132763 | -2.098968 | 2.352599 |
| 1 | 7.575501 | -3.068089 | 2.134677 |
| 6 | 5.798169 | -2.057847 | 2.840211 |
| 6 | 5.046798 | -3.242499 | 3.049602 |
| 1 | 5.512924 | -4.204313 | 2.848476 |
| 6 | 3.749105 | -3.176582 | 3.486707 |
| 1 | 3.187218 | -4.094771 | 3.619940 |
| 6 | 3.091653 | -1.936669 | 3.732375 |
| 8 | 3.162322 | 0.148440 | -0.847681 |
| 8 | 1.373926 | -0.395023 | 0.993648 |
| 8 | 1.554692 | 1.962697 | 0.080534 |
| 8 | 3.470654 | 1.061634 | 1.460373 |
| 15 | 2.353531 | 0.550466 | 0.366613 |
| 6 | 1.619213 | -1.988530 | 4.117498 |
| 6 | 1.382789 | -2.646338 | 5.497966 |
| 6 | 0.776622 | -2.737131 | 3.058130 |
| 1 | 1.230452 | -0.967080 | 4.160479 |
| 6 | -0.108041 | -2.634043 | 5.870014 |
| 1 | 1.739861 | -3.685359 | 5.466138 |
| 1 | 1.962376 | -2.137828 | 6.273590 |
| 6 | -0.714098 | -2.709369 | 3.416028 |
| 1 | 1.107349 | -3.778902 | 2.990063 |
| 1 | 0.949313 | -2.280302 | 2.084451 |
| 6 | -0.967187 | -3.322584 | 4.798954 |
| 1 | -0.253582 | -3.113502 | 6.846730 |
| 1 | -0.436043 | -1.590350 | 5.979530 |
| 1 | -1.288663 | -3.247725 | 2.653657 |
| 1 | -1.058010 | -1.669793 | 3.408119 |
| 1 | -2.030152 | -3.251408 | 5.063930 |
| 1 | -0.717370 | -4.393511 | 4.769578 |
| 6 | 4.634556 | 2.796680 | -1.932064 |
| 6 | 5.525361 | 4.054764 | -1.808027 |
| 6 | 5.505659 | 1.619990 | -2.416789 |
| 1 | 4.317938 | 2.537702 | -0.917792 |
| 6 | 6.666764 | 3.803231 | -0.808335 |
| 1 | 5.936111 | 4.302119 | -2.797742 |
| 1 | 4.933789 | 4.916514 | -1.489253 |
| 6 | 6.624109 | 1.332866 | -1.405713 |
| 1 | 5.962916 | 1.868145 | -3.385064 |
| 1 | 4.894313 | 0.730007 | -2.569861 |
| 6 | 7.504736 | 2.573120 | -1.189551 |
| 1 | 7.306547 | 4.691740 | -0.733105 |
| 1 | 6.226840 | 3.646537 | 0.187116 |
| 1 | 7.236112 | 0.490123 | -1.751528 |
| 1 | 6.174720 | 1.023279 | -0.452849 |
| 1 | 8.262876 | 2.376869 | -0.420667 |
| 1 | 8.051024 | 2.788843 | -2.119570 |

| | | | |
|---|---|---|---|
| 1 | 0.195014 | 0.238139 | 2.182549 |
| 8 | -0.448480 | 0.635455 | 2.809422 |
| 1 | 0.092023 | 1.208640 | 3.366650 |

---

**3a***

---

Number of imaginary frequencies : 0
The smallest frequencies are : 6.9644 12.2695 14.1572 cm(-1)

Electronic energy : HF=-6614.012095
Zero-point correction= 1.888319 (Hartree/Particle)
Thermal correction to Energy= 1.999629
Thermal correction to Enthalpy= 2.000573
Thermal correction to Gibbs Free Energy= 1.732672
Sum of electronic and zero-point Energies= -6612.123776
Sum of electronic and thermal Energies= -6612.012466
Sum of electronic and thermal Enthalpies= -6612.011522
Sum of electronic and thermal Free Energies= -6612.279423

.............................................................

Cartesian Coordinates

.............................................................

| | | | |
|---|---|---|---|
| 6 | 3.547140 | 0.036625 | 2.861124 |
| 6 | 4.714218 | 0.576026 | 2.292191 |
| 6 | 5.911547 | 0.511019 | 3.017088 |
| 6 | 5.933781 | -0.060553 | 4.291557 |
| 6 | 4.763654 | -0.569362 | 4.859675 |
| 6 | 3.568719 | -0.523755 | 4.137678 |
| 15 | 4.514860 | 1.339776 | 0.621144 |
| 6 | 6.223486 | 1.542562 | -0.071152 |
| 6 | 7.185112 | 2.383272 | 0.516022 |
| 6 | 8.454735 | 2.513176 | -0.047074 |
| 6 | 8.776600 | 1.823742 | -1.219960 |
| 6 | 7.820031 | 1.011039 | -1.829407 |
| 6 | 6.552863 | 0.872879 | -1.257929 |
| 46 | 3.105892 | -0.020984 | -0.670832 |
| 6 | 1.255608 | -0.519055 | -1.759413 |
| 6 | 0.979370 | 0.581657 | -0.942846 |
| 6 | 0.658525 | 1.967592 | -1.439241 |
| 15 | 4.183683 | -2.142604 | -0.721678 |
| 6 | 5.580956 | -2.472748 | 0.447274 |
| 6 | 5.330786 | -2.921127 | 1.754227 |
| 6 | 6.372885 | -3.057104 | 2.671267 |
| 6 | 7.682849 | -2.744066 | 2.304358 |
| 6 | 7.942819 | -2.289370 | 1.010224 |
| 6 | 6.902360 | -2.154442 | 0.090779 |
| 6 | 4.186645 | 3.116217 | 1.051069 |
| 6 | 4.177656 | 4.054338 | 0.002693 |
| 6 | 3.957759 | 5.407126 | 0.255882 |
| 6 | 3.734778 | 5.847985 | 1.565308 |
| 6 | 3.720574 | 4.922886 | 2.609698 |
| 6 | 3.944779 | 3.565823 | 2.356035 |
| 6 | 4.852978 | -2.715430 | -2.344418 |
| 6 | 5.757843 | -3.781730 | -2.486978 |
| 6 | 6.215188 | -4.159979 | -3.749905 |
| 6 | 5.784294 | -3.472246 | -4.887940 |
| 6 | 4.892731 | -2.405840 | -4.757461 |
| 6 | 4.429097 | -2.030392 | -3.494299 |
| 6 | 2.971236 | -3.450281 | -0.220802 |
| 6 | 1.962426 | -3.073783 | 0.680146 |
| 6 | 1.021917 | -4.003711 | 1.121459 |
| 6 | 1.056210 | -5.318105 | 0.653452 |

| | | | |
|---|---|---|---|
| 6 | 2.055787 | -5.702092 | -0.244859 |
| 6 | 3.013452 | -4.778453 | -0.670524 |
| 1 | 1.261963 | 2.229569 | -2.310821 |
| 1 | 1.376003 | -0.388862 | -2.831819 |
| 1 | 0.953075 | -1.513057 | -1.448741 |
| 1 | 4.353339 | 3.720782 | -1.017552 |
| 1 | 3.939200 | 2.861830 | 3.181054 |
| 1 | 3.952482 | 6.117098 | -0.566408 |
| 1 | 3.535775 | 5.251315 | 3.628876 |
| 1 | 3.559785 | 6.901438 | 1.764021 |
| 1 | 6.936431 | 2.949785 | 1.408626 |
| 1 | 5.808886 | 0.241809 | -1.734706 |
| 1 | 9.189442 | 3.160712 | 0.423693 |
| 1 | 8.054923 | 0.482258 | -2.749299 |
| 1 | 9.763782 | 1.930537 | -1.661239 |
| 1 | 6.834298 | 0.882558 | 2.586370 |
| 1 | 2.614689 | 0.039799 | 2.308077 |
| 1 | 6.872246 | -0.114742 | 4.836693 |
| 1 | 2.647298 | -0.925778 | 4.547947 |
| 1 | 4.786813 | -1.010415 | 5.852664 |
| 1 | 6.117991 | -4.306707 | -1.607618 |
| 1 | 3.730746 | -1.204650 | -3.393313 |
| 1 | 6.913138 | -4.987348 | -3.844585 |
| 1 | 4.556529 | -1.862288 | -5.635942 |
| 1 | 6.146214 | -3.763974 | -5.869963 |
| 1 | 4.319253 | -3.159669 | 2.063913 |
| 1 | 7.126425 | -1.805545 | -0.910613 |
| 1 | 6.154564 | -3.398696 | 3.678783 |
| 1 | 8.956421 | -2.036308 | 0.710898 |
| 1 | 8.492016 | -2.847113 | 3.022110 |
| 1 | 1.892555 | -2.053437 | 1.042761 |
| 1 | 3.781842 | -5.095499 | -1.367934 |
| 1 | 0.255959 | -3.677270 | 1.813723 |
| 1 | 2.087495 | -6.720915 | -0.621216 |
| 1 | 0.301266 | -6.032126 | 0.969633 |
| 1 | 0.533428 | 0.378873 | 0.024898 |
| 1 | -2.875713 | 0.144728 | -1.629520 |
| 6 | -1.540839 | 2.997981 | -0.965890 |
| 6 | -0.448560 | 4.008382 | -0.543415 |
| 1 | -1.924334 | 2.459290 | -0.095292 |
| 1 | -2.373078 | 3.461901 | -1.491220 |
| 7 | -0.801617 | 2.073277 | -1.833283 |
| 16 | -1.356916 | 1.558832 | -3.283942 |
| 8 | -0.205108 | 1.113105 | -4.073247 |
| 8 | -2.458747 | 0.576220 | -3.054910 |
| 6 | 0.730643 | 3.030713 | -0.322041 |
| 1 | 0.584803 | 2.536851 | 0.641059 |
| 1 | 1.706401 | 3.508715 | -0.284474 |
| 6 | -0.196922 | 5.012926 | -1.687051 |
| 6 | 1.034423 | 5.130163 | -2.342207 |
| 6 | -1.237999 | 5.866683 | -2.087998 |
| 6 | 1.219794 | 6.064912 | -3.366483 |
| 1 | 1.868232 | 4.498875 | -2.059883 |
| 6 | -1.057826 | 6.799888 | -3.105157 |
| 1 | -2.199830 | 5.808985 | -1.587099 |
| 6 | 0.176540 | 6.903723 | -3.752842 |
| 1 | 2.186269 | 6.132292 | -3.859017 |
| 1 | -1.883578 | 7.442967 | -3.395656 |
| 1 | 0.319708 | 7.630070 | -4.547944 |
| 6 | -0.824207 | 4.780460 | 0.720218 |
| 6 | -2.157768 | 4.961254 | 1.112565 |
| 6 | 0.183014 | 5.365108 | 1.499974 |

| | | | |
|---|---|---|---|
| 6 | -2.473959 | 5.706505 | 2.251621 |
| 1 | -2.965445 | 4.514451 | 0.541602 |
| 6 | -0.128805 | 6.100285 | 2.642484 |
| 1 | 1.220717 | 5.243653 | 1.213643 |
| 6 | -1.461620 | 6.277142 | 3.024046 |
| 1 | -3.514836 | 5.831174 | 2.536472 |
| 1 | 0.672539 | 6.532738 | 3.235256 |
| 1 | -1.706821 | 6.851168 | 3.913259 |
| 6 | -2.120475 | 2.961259 | -4.134962 |
| 6 | -3.466623 | 3.405183 | -3.905384 |
| 6 | -1.296005 | 3.585675 | -5.049753 |
| 6 | -4.380332 | 2.838911 | -2.969498 |
| 6 | -3.915016 | 4.532716 | -4.680508 |
| 6 | -1.760491 | 4.684337 | -5.801246 |
| 1 | -0.286944 | 3.219576 | -5.195998 |
| 6 | -5.647696 | 3.354831 | -2.810254 |
| 1 | -4.083567 | 1.992774 | -2.368753 |
| 6 | -5.232781 | 5.031860 | -4.491262 |
| 6 | -3.043048 | 5.142958 | -5.620247 |
| 1 | -1.095233 | 5.160857 | -6.513462 |
| 6 | -6.085022 | 4.459712 | -3.576613 |
| 1 | -6.317034 | 2.899835 | -2.085998 |
| 1 | -5.551960 | 5.883175 | -5.086497 |
| 1 | -3.409940 | 5.990377 | -6.193218 |
| 1 | -7.088386 | 4.851970 | -3.440335 |
| 6 | -2.145002 | -3.207144 | -0.045222 |
| 6 | -2.279289 | -3.906308 | -1.227637 |
| 6 | -2.520529 | -5.317712 | -1.164418 |
| 6 | -2.590955 | -6.128181 | -2.331011 |
| 1 | -2.464040 | -5.662383 | -3.301175 |
| 6 | -2.811649 | -7.484574 | -2.235008 |
| 1 | -2.854457 | -8.087919 | -3.137171 |
| 6 | -2.986488 | -8.100862 | -0.971786 |
| 1 | -3.162821 | -9.170972 | -0.913056 |
| 6 | -2.937921 | -7.342649 | 0.175454 |
| 1 | -3.077463 | -7.803818 | 1.149894 |
| 6 | -2.701325 | -5.941920 | 0.111210 |
| 6 | -2.670936 | -5.143913 | 1.279188 |
| 1 | -2.893613 | -5.611099 | 2.233822 |
| 6 | -2.394065 | -3.789202 | 1.236502 |
| 6 | -2.403360 | -3.010401 | 2.501520 |
| 6 | -1.872723 | -3.536863 | 3.667727 |
| 1 | -1.342756 | -4.484004 | 3.633603 |
| 6 | -2.023281 | -2.883051 | 4.915940 |
| 6 | -1.507003 | -3.450829 | 6.114027 |
| 1 | -0.952259 | -4.383246 | 6.050927 |
| 6 | -1.719564 | -2.842013 | 7.329238 |
| 1 | -1.324304 | -3.285067 | 8.238569 |
| 6 | -2.472839 | -1.644842 | 7.399687 |
| 1 | -2.663968 | -1.185698 | 8.365166 |
| 6 | -2.967343 | -1.059315 | 6.255104 |
| 1 | -3.552115 | -0.149169 | 6.314243 |
| 6 | -2.733109 | -1.638207 | 4.979221 |
| 6 | -3.186649 | -1.025877 | 3.764810 |
| 6 | -3.031383 | -1.733324 | 2.587202 |
| 6 | -2.238005 | -3.215045 | -2.556390 |
| 6 | -1.032341 | -3.236307 | -3.327778 |
| 6 | 0.182813 | -3.786802 | -2.830583 |
| 1 | 0.211162 | -4.196122 | -1.826566 |
| 6 | 1.324332 | -3.786219 | -3.601161 |
| 1 | 2.244090 | -4.195725 | -3.199403 |
| 6 | 1.316247 | -3.237043 | -4.906237 |

| | | | |
|---|---|---|---|
| 1 | 2.229707 | -3.240931 | -5.492721 |
| 6 | 0.159283 | -2.694979 | -5.416072 |
| 1 | 0.141754 | -2.261321 | -6.412912 |
| 6 | -1.036901 | -2.681656 | -4.649160 |
| 6 | -2.241945 | -2.134748 | -5.153545 |
| 1 | -2.249783 | -1.714573 | -6.156153 |
| 6 | -3.377277 | -2.106902 | -4.382699 |
| 1 | -4.276209 | -1.653586 | -4.784874 |
| 6 | -3.401654 | -2.631411 | -3.062812 |
| 6 | -3.838253 | 0.322989 | 3.756600 |
| 6 | -5.242323 | 0.397544 | 3.446788 |
| 6 | -6.032814 | -0.752404 | 3.164103 |
| 1 | -5.575288 | -1.734172 | 3.202970 |
| 6 | -7.366763 | -0.642759 | 2.837225 |
| 1 | -7.941914 | -1.537871 | 2.617272 |
| 6 | -7.994342 | 0.624583 | 2.787144 |
| 1 | -9.046102 | 0.698469 | 2.525575 |
| 6 | -7.267468 | 1.756699 | 3.076844 |
| 1 | -7.737842 | 2.736717 | 3.051090 |
| 6 | -5.888976 | 1.675116 | 3.412205 |
| 6 | -5.126976 | 2.832952 | 3.713889 |
| 1 | -5.616704 | 3.803760 | 3.698479 |
| 6 | -3.792649 | 2.733161 | 4.013367 |
| 1 | -3.227676 | 3.634571 | 4.224455 |
| 6 | -3.109577 | 1.482976 | 4.028837 |
| 8 | -3.326143 | -0.021377 | -0.736171 |
| 8 | -1.453803 | 0.334388 | 1.066171 |
| 8 | -1.655306 | -1.904869 | -0.123680 |
| 8 | -3.548704 | -1.187836 | 1.406439 |
| 15 | -2.420995 | -0.583642 | 0.411951 |
| 6 | -1.614057 | 1.498508 | 4.316345 |
| 6 | -1.290812 | 1.924329 | 5.768727 |
| 6 | -0.842699 | 2.426883 | 3.348023 |
| 1 | -1.219164 | 0.488984 | 4.168167 |
| 6 | 0.220498 | 1.864485 | 6.040991 |
| 1 | -1.649742 | 2.950992 | 5.927037 |
| 1 | -1.821392 | 1.290906 | 6.484752 |
| 6 | 0.668301 | 2.357136 | 3.601559 |
| 1 | -1.175726 | 3.461485 | 3.483059 |
| 1 | -1.071403 | 2.146282 | 2.320831 |
| 6 | 1.011783 | 2.729691 | 5.049213 |
| 1 | 0.426629 | 2.174558 | 7.073471 |
| 1 | 0.553486 | 0.820910 | 5.953023 |
| 1 | 1.191230 | 3.022405 | 2.906786 |
| 1 | 1.015859 | 1.339226 | 3.398416 |
| 1 | 2.089230 | 2.620715 | 5.227859 |
| 1 | 0.766048 | 3.788564 | 5.218258 |
| 6 | -4.679528 | -2.549134 | -2.235713 |
| 6 | -5.546549 | -3.827462 | -2.317080 |
| 6 | -5.570731 | -1.328654 | -2.547222 |
| 1 | -4.379292 | -2.446979 | -1.188057 |
| 6 | -6.702414 | -3.757339 | -1.306795 |
| 1 | -5.941635 | -3.925047 | -3.338301 |
| 1 | -4.939479 | -4.716433 | -2.128940 |
| 6 | -6.703530 | -1.222894 | -1.515934 |
| 1 | -6.013898 | -1.437259 | -3.546758 |
| 1 | -4.975345 | -0.413142 | -2.565086 |
| 6 | -7.561331 | -2.497953 | -1.502952 |
| 1 | -7.324795 | -4.657832 | -1.380866 |
| 1 | -6.278049 | -3.750884 | -0.292444 |
| 1 | -7.329955 | -0.348270 | -1.732569 |
| 1 | -6.269062 | -1.058334 | -0.521026 |

| | | | |
|---|---|---|---|
| 1 | -8.326931 | -2.434760 | -0.719332 |
| 1 | -8.096727 | -2.577619 | -2.460312 |
| 1 | -0.176012 | -0.480960 | 2.182605 |
| 8 | 0.432898 | -0.986361 | 2.754085 |
| 1 | -0.140945 | -1.596194 | 3.233648 |
